# Analysis of rare Parkinson’s disease variants in millions of people

**DOI:** 10.1038/s41531-023-00608-8

**Published:** 2024-01-08

**Authors:** Vanessa Pitz, Mary B. Makarious, Sara Bandres-Ciga, Hirotaka Iwaki, 23andMe Research Team, Stella Aslibekyan, Adam Auton, Elizabeth Babalola, Robert K. Bell, Jessica Bielenberg, Katarzyna Bryc, Emily Bullis, Daniella Coker, Gabriel Cuellar Partida, Devika Dhamija, Sayantan Das, Sarah L. Elson, Nicholas Eriksson, Teresa Filshtein, Alison Fitch, Kipper Fletez-Brant, Pierre Fontanillas, Will Freyman, Julie M. Granka, Alejandro Hernandez, Barry Hicks, David A. Hinds, Ethan M. Jewett, Yunxuan Jiang, Katelyn Kukar, Alan Kwong, Keng-Han Lin, Bianca A. Llamas, Maya Lowe, Jey C. McCreight, Matthew H. McIntyre, Steven J. Micheletti, Meghan E. Moreno, Priyanka Nandakumar, Dominique T. Nguyen, Elizabeth S. Noblin, Jared O’Connell, Aaron A. Petrakovitz, G. David Poznik, Alexandra Reynoso, Morgan Schumacher, Anjali J. Shastri, Janie F. Shelton, Jingchunzi Shi, Suyash Shringarpure, Qiaojuan Jane Su, Susana A. Tat, Christophe Toukam Tchakouté, Vinh Tran, Joyce Y. Tung, Xin Wang, Wei Wang, Catherine H. Weldon, Peter Wilton, Corinna D. Wong, Andrew B. Singleton, Mike Nalls, Karl Heilbron, Cornelis Blauwendraat

**Affiliations:** 1 Integrative Neurogenomics Unit, Laboratory of Neurogenetics, National Institute on Aging, National Institutes of Health, Bethesda, MD USA; 2 Molecular Genetics Section, Laboratory of Neurogenetics, National Institute on Aging, National Institutes of Health, Bethesda, MD USA; 3 Department of Clinical and Movement Neurosciences, UCL Queen Square Institute of Neurology, London, UK; 4 UCL Movement Disorders Centre, University College London, London, UK; 5 Center for Alzheimer’s and Related Dementias (CARD), National Institute on Aging and National Institute of Neurological Disorders and Stroke, National Institutes of Health, Bethesda, MD USA; 6 Data Tecnica International, Washington, DC, USA; 7 23andMe, Inc., Sunnyvale, CA USA

**Keywords:** Parkinson's disease, Risk factors

## Abstract

Although many rare variants have been reportedly associated with Parkinson’s disease (PD), many have not been replicated or have failed to replicate. Here, we conduct a large-scale replication of rare PD variants. We assessed a total of 27,590 PD cases, 6701 PD proxies, and 3,106,080 controls from three data sets: 23andMe, Inc., UK Biobank, and AMP-PD. Based on well-known PD genes, 834 variants of interest were selected from the ClinVar annotated 23andMe dataset. We performed a meta-analysis using summary statistics of all three studies. The meta-analysis resulted in five significant variants after Bonferroni correction, including variants in *GBA1* and *LRRK2*. Another eight variants are strong candidate variants for their association with PD. Here, we provide the largest rare variant meta-analysis to date, providing information on confirmed and newly identified variants for their association with PD using several large databases. Additionally we also show the complexities of studying rare variants in large-scale cohorts.

## Introduction

Over the past several decades, common and rare variants in multiple genes have been associated with Parkinson’s disease (PD). PD is a neurodegenerative disorder primarily affecting dopaminergic neurons in the substantia nigra, and is caused by a combination of aging, environmental factors, and genetics. Different angles allow us to better understand the interplay between genetics and disease: genetics account for a large proportion of PD risk^1,2^, and by looking at an individual’s genetic makeup, we can understand the likelihood that someone develops the disease. In a wider-cast net, we can look at the impact of genetic factors on a population and estimate the proportion of cases that are caused by genetic factors. We can then estimate the penetrance of a specific genetic variant: the percentage of variant carriers who have the disease. High penetrance indicates a strong correlation between variant and disease status, and results in high odds ratios (ORs), whereas low penetrance indicates that other factors may contribute more to disease development and result in lower ORs.

There are two primary approaches to studying genetics in PD: monogenic PD and genome-wide association studies (GWAS). Monogenic PD involves mutations in a single gene, which are rare and account for a small percentage of PD cases, with most studies only including a handful of cases to be studied. This can be differentiated into autosomal dominant inheritance, which means that a mutation in one copy of the gene (one allele) from one parent is sufficient to express the disease, whereas autosomal recessive inheritance requires both copies of the gene (two alleles), one from each parent. Genes such as *SNCA, LRRK2* (both autosomal dominant), *PRKN, PINK1*, and *DJ-1* (all autosomal recessive) have been identified through this approach^3–7^. GWAS, on the other hand, aims to identify common genetic variations, like single nucleotide polymorphisms (SNPs), that are associated with PD risk and provide insights into the broader genetic landscape of PD in the population and mostly focus on common variants with a minor allele frequency (MAF) of >5%. Several large-scale case–control GWAS have been performed and have identified 90 risk variants^8^. Many variants occur less frequently in the general population (rare, MAF < 1%) and have been considered either a strong risk factor (OR > 5–10) or a monogenic cause of PD if they are highly penetrant^9,10^.

Pathogenic variants in *GBA1* and *LRRK2* are the most common high-risk genetic factors for PD, and typically present in 1–10% of the PD population depending on genetic ancestry. Prior studies suggest a lifetime penetrance of ~9% for *GBA1* mutations, and ~25% for *LRRK2* p.G2019S^11,12^, although estimates vary with age, ethnicity, and methodology^13,14^. Most other known pathogenic variants are very rare (allele frequency <0.1%, e.g., damaging variants in *SNCA* and *PRKN*).

Typically, these rare variants are also associated with a slightly different PD phenotype, highlighting the importance of their research even more. Especially the genotype–phenotype relationship of *GBA1* carriers in PD is of great interest in the field: Whilst overall more severe motor and non-motor symptoms are seen in *GBA1* variant carriers^15–17^, pathogenic variants resulting from recombinant alleles cause Gaucher’s disease (GD), and risk variants are not necessarily associated with GD but increase the risk of developing PD^18^. While most *GBA1* carriers will never develop PD, PD patients who are *GBA1* carriers typically have more severe motor/cognitive phenotypes than idiopathic PD patients^19^.

Similarly, pathogenic *SNCA* variant carriers exhibit a higher prevalence of dementia (around 50%) in comparison to idiopathic PD, manifesting 5–22 years after motor onset^20,21^. 30% of idiopathic PD cases had dementia at any point in their disease progression (point prevalence), but it is estimated that over 75% of PD patients present with dementia within 10 years after the onset of motor symptoms^22^. *PRKN* carriers present mostly with a very early onset and an akinesia-related phenotype^23,24^.

It is important to note that the status of some reported PD genes is debated, since most associations are reported without a replication cohort or in small, often biased single-case or family studies^1^. Previously, we investigated the *SNCA* p.H50Q mutation, which was identified in a pathologically-proven PD case without family segregation^25^. Other data do not support a pathogenic role for *SNCA* p.H50Q despite functional evidence supporting a potential role in disease^26,27^. This potentially also applies to variants in other genes including *DNAJC13, EIF4G1, GIGYF2, HTRA2, LRP10, TMEM230,* and *UCLH1*, all of which have been categorized previously as potentially low-confidence PD genes^1^. A robust assessment of high-risk and causal PD variants will be incredibly valuable for the global PD community from laboratory researchers to genetic counselors.

Here we tested PD mutations from the ClinVar database for association with PD in three large case-control cohorts (23andMe, Inc., UK Biobank, and AMP-PD) totaling over 3 million individuals (27,590 cases, 6701 proxies, and 3,106,080 controls). The large number of participants in this study is an opportunity to assess rare variants in PD more reliably, which has not previously been possible to this extent. Our goal was to create lists of high-confidence and low-confidence PD variants. This work improves our understanding of the clinical relevance of these variants, which can then be used to improve genetic testing in patients.

## Results

### Variant selection criteria and included dataset overview

**Fig. 1:**
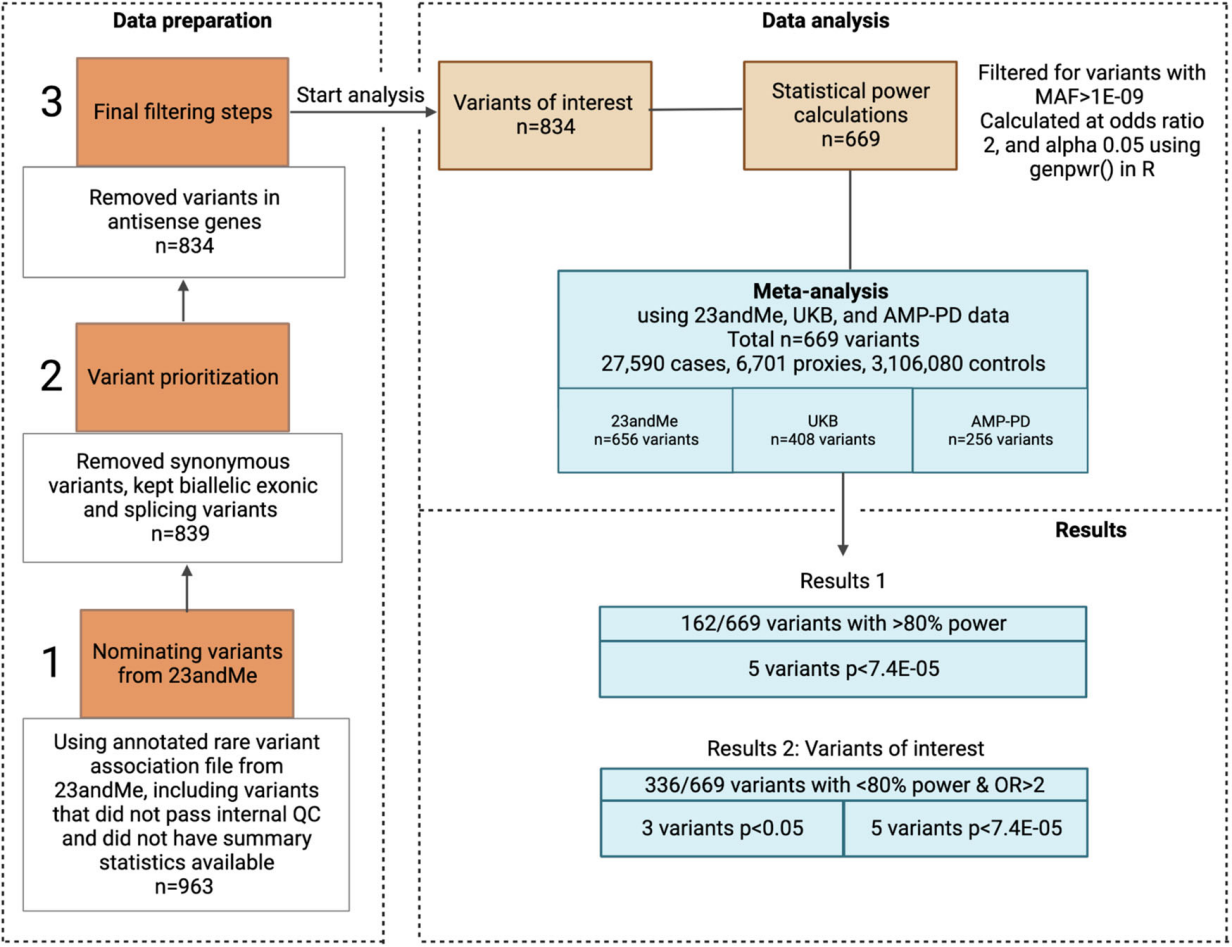
Analysis flowchart. *Data preparation:* Variant selection was based on an annotated 23andMe rare variant association file, including variants that passed or failed internal quality control steps, resulting in some variants not having summary statistics available but could still be used for variant selection in other data sets. Data were filtered to remove synonymous variants and to keep biallelic exonic, and splicing variants. *Data analysis:* 669 variants of interest were used to calculate statistical power for each variant at odds ratio of 2, and alpha 0.05. 656 variants had summary statistics available in 23andMe data and were analyzed individually but also as part of a meta-analysis, using 23andMe variants found in UK Biobank and AMP-PD data. *Results:* The results were organized into categories depending on whether the variants reached a statistical power of more or less than 80% at an odds ratio (OR) of 2 and successfully passed Bonferroni correction. Subsequently, we examined the clinical significance of these variants and their previous association with Parkinson’s disease (PD), considering information from the ClinVar database. For each variant, we calculated the odds ratio (OR) and estimated the corresponding 95% confidence intervals, providing a range of values to indicate the precision of our findings. Figure created with BioRender.com.

In total, we selected 669 genetic variants for further investigation into their relationship with PD (Fig. 1, see Methods). 471 (70.4%) of the annotated variants had either no reported clinical significance, or had conflicting or uncertain interpretations in ClinVar. Of the remaining 198 variants, 109 variants (16.3%) were classified as pathogenic and/or likely pathogenic, followed by 82 variants (12.3%) classified as benign or likely benign variants. Five variants (0.7%) were reported to be risk factors for PD, and 2 (0.3%) variants were reported to be either pathogenic/risk factors (Supplementary Table 1A). The 669 variants were located in 32 genes (Supplementary Table 2). Three genes accounted for 256/669 (28%) of all variants: GBA1 (*n* = 87), LRRK2 (*n* = 86), and VPS13C (*n* = 83).

The list of variants include variants that did not pass 23andMe-internal QC and, therefore had no summary statistics, but were still useful for variant selection in other studies. 256 (38.3%) variants were available in the AMP-PD dataset, 608 (90.9%) in UK Biobank, and 656 (98.0%) variants in 23andMe, resulting in a total of 679 unique variants. 249 (36.7%) variants were solely contributed by 23andMe, whereas 18 (2.7%) variants were only found in UK Biobank, and 1 (0.1%) variant was only found in AMP-PD (Supplementary Fig. 1).

Variants from the AMP-PD and UK Biobank datasets were derived from whole exomes (UKB) and the exome regions of whole genome sequencing (AMP-PD). Of the 679 variants included in the meta-analysis, 264 (39%) were genotyped and 405 (61%) were imputed in the 23andMe dataset (Supplementary Table 8).

Figure 1 provides a visual overview of our analysis approach. Next, we categorized our results based on variants where we had statistical power exceeding 80% at an odds ratio (OR) of 2. We then examined the variants that passed Bonferroni correction, considering their previous association with PD and their documented clinical significance as reported in the ClinVar database. Finally, we focused on variants that did not reach >80% statistical power at OR = 2 but had OR greater than 2 in the meta-analysis, and again considered variants passing Bonferroni correction. The results were presented using OR and estimated penetrance (based on lifetime risk) values along with corresponding 95% confidence intervals.

### Analysis of large-scale datasets

We conducted a meta-analysis using summary statistics generated by the single-variant association testing data from 23andMe, UK Biobank, and AMP-PD (Table 1). 23andMe data comprised 25,034 PD cases and 3,065,473 controls. Cases were 59.9% male with a mean age of 72.3 (±10.9) years, controls were 43.5% male with a mean age of 50.1 (±17.5) years. The UK Biobank dataset comprised 1,105 cases and 6701 proxies. Cases and proxies were 45.6% male with an average age of 59.1 (±7.1) years, 38,051 were controls (48.6% male) with a mean age of 64.1 (±2.8) years. AMP-PD had 1451 cases (63.7% male) with a mean age of 61.3 (±10.2) years, and 2556 controls (49.8% male) with a mean age of 70.7 (±13.2) years. A summary of cohort demographics can be found in Table 1.

**Table 1:** Cohort demographics.

	23andMe	AMP-PD	UKB	Total
Cases, *n*	25,034	1451	1105 (+6701 proxies)^a^	27,590 (+6701 proxies)
Age, mean ± SD	72.3 (±10.9)^b^	61.3 (±10.2)^c^	59.1 (±7.1)^d^	
Male %	59.9	63.7	45.6	
Controls	3,065,473	2,556	38,051	3,106,080
Age, mean ± SD	50.1 (±17.5)	70.7 (±13.2)^c^	64.1 (±2.8)^d^	
Male %	43.5	49.8	48.6	
Nominated variants	692	301	644	
Type of data	Imputed genotype data	Whole genome sequencing	Whole exome sequencing	

*UKB* UK Biobank, *AMP-PD* Accelerating Medicines Partnership Parkinson’s disease

^a^Proxies in UK Biobank data include: 6033 parents and 668 siblings.

^b^Current age at time of analysis.

^c^Age at analysis for AMP-PD.

^d^Age at recruitment for UK Biobank data.

We conducted power calculations for all variants in the meta-analysis, using the R package genpwr and accounted for the tool’s MAF limit of >1E-09 (*n* = 669, Supplementary Table 8) with a special focus on variants with a *p* value > 0.05 (*n* = 601) to assess whether we had >80% statistical power to detect an association assuming OR = 2, alpha = 0.05, and an additive model. The OR and alpha values set for this calculation are generous, since an OR of two is insufficient to cause monogenic disease in affected individuals and we are not correcting for multiple testing. We selected these lenient values in order to offset other limitations to our experimental design (see “Discussion”). Therefore, under these parameters, any association that had >80% power but was not statistically significant in our analysis is very likely to be a spurious association if the true inheritance mode of the variant is either additive or dominant.

Based on the parameters set for our power calculation, we had 80% or more power for 162/669 (23.9%) variants at OR 2 and alpha 0.05. One hundred sixty-two variants were located in 25 genes. Three genes accounted for 45% of all variants: VPS13C (*n* = 30), POLG (*n* = 24), and DNAJC13 (*n* = 19). Of the 162 variants with sufficient power, 105 (64.8%) were reported in ClinVar to be of conflicting or unknown clinical significance, 47 (29.0%) were benign, 8 (4.9%) pathogenic, and 2 (0.6%) were risk factors.

**Fig. 2:**
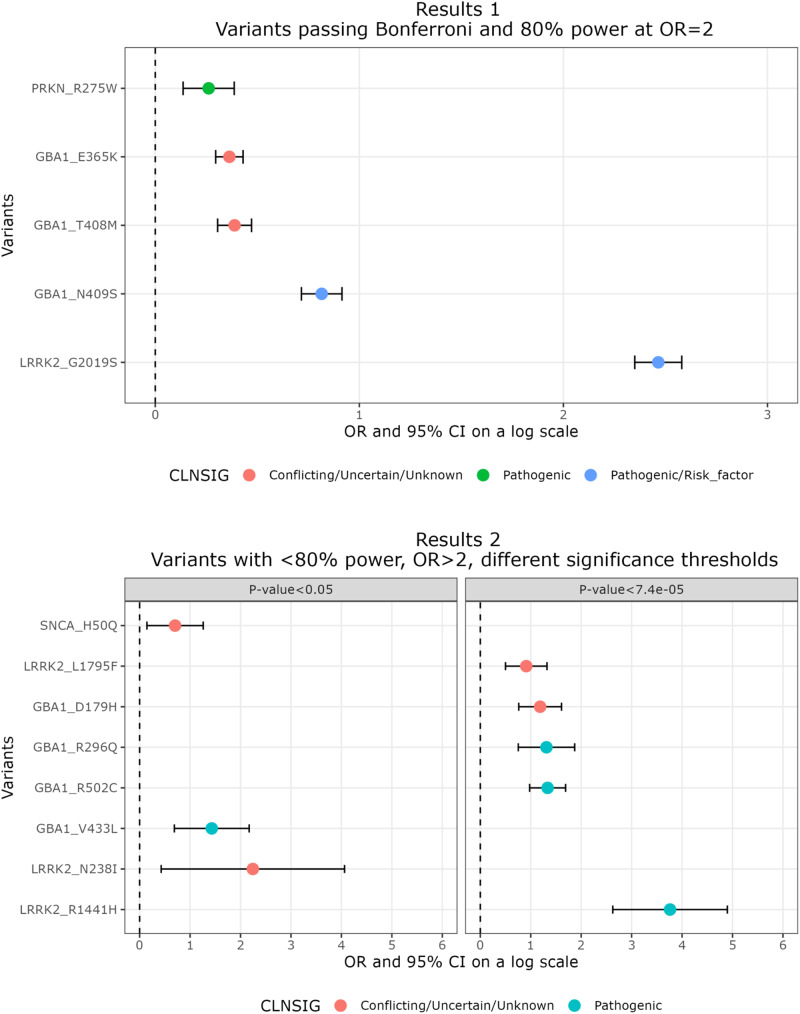
Variants passing Bonferroni correction and 80% power. *Results 1:* Forest plot showing five variants passing Bonferroni correction at *p* < 7.4E-05 in the meta-analysis and reaching 80% statistical power at OR = 2 and alpha = 0.05. *Results 2:* Forest plot showing five strong candidate variants passing Bonferroni correction at *p* < 7.4E-05 but did not reach 80% power at OR = 2 (right). Three variants passed *p* < 0.05, have previously been linked to PD, and are located in dominantly inherited genes. All variants had calculated ORs greater than 2 in the meta-analysis, suggesting that they are good candidates to reach 80% power at higher OR calculations. Data is based on OR (dot) and 95% CI (error bar) and for visualization purposes the *x*-axis is on a log scale. Colors indicate clinical significance of the variant: Conflicting/uncertain/unknown (red), pathogenic (green), and pathogenic/risk factor (blue) based on ClinVar annotations.

13/162 (8.0%) variants passed a significant threshold of *p* < 0.05 and 5 variants passed multiple test correction at *p* < 7.4E-05 (Fig. 2 top, Table 2). Among those five variants, three were pathogenic: *LRRK2* p.G2019S (rs34637584, OR = 11.8, 95%CI: 10.5–13.2), *GBA1* p.N409S (rs76763715, OR = 2.3, 95%CI: 2.0-2.5), *PRKN* p.R275W (rs34424986, OR = 1.3 95%CI: 1.1–1.5). Two *GBA1* variants were of conflicting or unknown clinical significance: p.T408M (rs75548401, OR = 1.5, 95%CI: 1.4–1.6) and p.E365K (rs2230288, OR = 1.4, 95%CI: 1.3–1.5). Based on the ClinVar database, all variants have previously been associated with PD. Based on the penetrance calculator described in^28^, we estimated a penetrance range of 2.7% for the *GBA1* variants, whereas *LRRK2* p.G2019S had a penetrance estimate of 25.1% (95%CI: 0.22–0.28). Penetrance was not calculated for *PRKN* since it is an autosomal recessive gene.

**Table 2:** List of five variants identified and eight strong candidate variants.

	rsID	Variant Name	Ref	Alt	CLNSIG	Power	OR	L95	U95	Penetrance	PL95	PU95	MAF (MAC) cases	MAF (MAC) controls
**R1**	rs34637584	**LRRK2 p.G2019S**	G	A	P/R	0.97	11.8	10.5	13.2	0.3	0.2	0.3	0.01 (470)	0.001 (3,385)
	rs76763715	**GBA1 p.N409S**	T	C	P/R	1.00	2.3	2.0	2.5	0.0	0.0	0.1	0.01 (487)	0.006 (18,190)
	rs75548401	**GBA1 p.T408M**	G	A	C/U/U	1.00	1.5	1.4	1.6	0.0	0.0	0.0	0.02 (206)	0.009 (1,013)
	rs2230288	**GBA1 p.E365K**	C	T	C/U/U	1.00	1.4	1.3	1.5	0.0	0.0	0.0	0.03 (1,099)	0.02 (74,759)
	rs34424986	**PRKN p.R275W**	G	A	P	1.00	1.3	1.1	1.5	NA	NA	NA	0.004 (138)	0.003 (8,532)
**R2.1**	rs34995376	LRRK2 p.R1441H	G	A	P	0.06	43.0	13.8	133.9	0.8	0.2	1.0	2e-04 (6)	0 (14)
	rs80356771	GBA1 p.R502C	G	A	P	0.60	3.8	2.7	5.4	0.1	0.0	0.1	0.001 (45)	4e-04 (1,271)
	rs78973108	GBA1 p.R296Q	C	T	P	0.10	3.7	2.1	6.5	0.9	0.2	1.0	1e-04 (4)	0 (10)
	rs147138516	GBA1 p.D179H	C	G	C/U/U	0.41	3.3	2.1	5.0	0.0	0.0	0.1	2e-04 (6)	1e-04 (272)
	rs111910483	LRRK2 p.L1795F	G	T	C/U/U	0.30	2.5	1.6	3.8	0.1	0.0	0.4	4e-04 (2)	1e-04 (53)
**R2.2**	rs28365216	LRRK2 p.N238I	A	T	C/U/U	0.05	9.4	1.5	58.1	0.6	0.1	1	1e-04 (2)	0 (3)
	rs80356769	GBA1 p.V433L	C	A	P	0.13	4.18	1.99	8.77	0.11	0.05	0.2	3e-04 (9)	0 (149)
	rs201106962	SNCA p.H50Q	A	C	C/U/U	0.64	2.0	1.2	3.5	0.05	0.02	0.1	0.001 (10)	4e-04 (18)

R1 are variants in our main results, having passed 80% and Bonferroni correction. R2 describes variants not passing 80% power with ORs greater than 2. R2.1 are variants passing Bonferroni correction, R2.2 are pathological variants passing *p* value < 0.05, dominantly inherited genes, and previously linked to PD. Variants in the R2 group are strong candidates for a potential association with PD, based on their high ORs and *p* value status. More information can be found in Supplementary Table 8. Variants with wide confidence intervals should be interpreted carefully.

*Ref* reference allele, *Alt* alternate allele, *CLNSIG* clinical significance based on ClinVar, Power statistical power at OR = 2 and alpha=0.05, *OR* odds ratio, *L95* Lower boundary 95% confidence interval, *U95* upper boundary 95% confidence interval, *PL95* Lower boundary 95% confidence interval penetrance, *PU95* Upper boundary 95% confidence interval penetrance, *MAF* minor allele frequency, *MAC* minor allele count, *P* pathogenic, *P/R* pathogenic/risk factor, *C/U/U* conflicting, uncertain, unknown.

149/162 (92.0%) variants did not pass *p* < 0.05. 33 (22.1%) of those have previously been associated with PD: 30 (90.9%) were of conflicting or unknown clinical significance, 1 (3.0%) variant was pathogenic, and 2 (6.0%) were risk factors. The pathogenic variant was *GLUD2* p.S498A (rs9697983, OR = 1.0, 95%CI: 0.97–1.07). The two risk factors were both located in the *GIGYF2* gene: p.N56S (rs72554080, OR = 1.7, 95%CI:0.98–2.90) and p.N457T (rs116074753, OR = 1.2, 95%CI: 076–1.90). The 30 conflicting or uncertain variants comprised 3 *LRRK2* variants: p.D2175H (rs72547981, OR = 1.73, 95%CI: 0.64–4.7), p.M1869T (rs35602796, OR = 1.16, 95%CI: 0.75–1.78), and p.L119P (rs33995463, OR = 1.02, 95%CI: 0.86–1.20).

Based on the parameters set for our power calculation, we had less than 80% power for 507/669 (75.8%). 507 variants were located in 28 genes, with the most variants being located in *LRRK2* (*n* = 79), *POLG* (*n* = 63), and *GBA1* (*n* = 54). 366 (72.2%) variants were of conflicting or unknown clinical significance, followed by 103 (20.3%) pathogenic, 35 (6.9%) benign variants, and 3 (0.6%) risk factors.

The power calculation was set to an OR of 2, since with the small MAFs in our data set, this was the limit of our calculation tool. There were 336/507 variants that did not reach 80% power at OR = 2 but had ORs greater than 2 in our analysis. 43/336 variants passed a significance threshold of *p* < 0.05 (not Bonferroni), 3 of which have previously been linked to PD, and are located in dominantly inherited genes that our models could generate reliable statistical outputs for: LRRK2 p.N238I (rs28365216, OR = 9.4, 95%CI:1.5–58.1), *GBA1* p.V433L (rs80356769, OR = 4.2, 95%CI: 2.0–8.8), and *SNCA* p.H50Q (rs201106962, OR = 2.0, 95%CI: 1.2–3.5). These variants were estimated to have a penetrance of 60% (95%CI: 0.1–1.0), 10% (95%CI: 0.0–0.2), and 5% (95%CI: 0.02–0.1), respectively.

Six variants had an OR > 2, power of less than 80% and passed multiple test correction at *p* < 7.4E-05. Of these six variants, three variants were of conflicting and uncertain significance, whereas three were pathogenic. The pathogenic variants were *LRRK2* p.R1441H (rs34995376, OR = 43.0, 95%CI: 13.8–133.9), and the *GBA1* variants p.R502C (rs80356771, OR = 3.8, 95%CI: 2.7–5.4) and p.R296Q (rs78973108, OR = 3.7, 95%CI: 2.1–6.5). All had a minor allele count greater than four in cases and controls, and MAFs were <0.01%. For *LRRK2* p.R1441H, we estimated a penetrance of 77.6% (95%CI: 0.21–1.0), whereas the *GBA1* variants had an estimated penetrance of 6.8% (95%CI: 0.05–0.1) and 6.8% (95%CI: 0.02–0.21) respectively. Among the variants with conflicting or uncertain clinical significance, of interest were the *LRRK2* variant p.L1795F (OR = 2.5, 95%CI: 1.6–3.8) with a penetrance of 4.3% (95%: 0.01–0.19) and the *GBA1* variant p.D179H (OR = 3.3, 95%CI: 2.1–5.0) with an estimated penetrance of 4.1% (95%CI: 0.02–0.07). Based on the ClinVar database, *GBA1* p.D179H was not previously reported to be associated with PD and identified in 4 cases, and eight controls in UK Biobank, 12 cases and 0 controls in 23andMe (OR = 3.4, 95%CI: 2.2–5.2, *p* = 4.3E-06). In AMP-PD, no effect allele carriers were identified. Interestingly, *LRRK2* p.L1795F has been identified previously in a family with PD^29^, however, no segregation was shown and further reports are lacking in the literature. This variant was identified in 2 cases and 0 controls in AMP-PD and showed a significant association with PD in the 23andMe cohort (OR: 2.3, 95% CI: 1.57–3.61, *P* = 0.0003), but was not present in UKB.

All variants and their respective statistics can be found in Supplementary Table 8.

## Discussion

Here, we assessed the role of rare variants and their relationship with PD using several large case-control datasets of European ancestry. We conducted a single rare variant association analysis of PD vs controls, including 23andMe, UK Biobank and AMP-PD; totaling over 3 million individuals comprising 27,590 cases, 6,701 PD proxy cases, and 3,106,080 controls. We provide robust evidence of five high-risk and causal (*LRRK2* p.G2019S, *GBA1* p.N409S, p.T408M, p.E365K, PRKN p.R275W) and five variants potentially strongly involved in PD disease development (*LRRK2* p.R1441H, p.L1795F, *GBA1* p.R502C, p.R296Q, p.D179H).

We provide evidence that a large number of variants that have previously been associated with PD are unlikely to be highly penetrant causes of PD with an additive or dominant mode of inheritance. However, these variants may be weakly penetrant or may have a recessive mode of inheritance. We also clearly showcase how complicated rare variants are to study, even though we include a very large number of individuals.

Our findings confirm the prominent role of variants in *LRRK2* and *GBA1* on increased PD risk, in particular the pathogenic variants: *LRRK2* p.G2019S and *GBA1* p.N409S, p.T408M, and p.E365K. These variants are well-known for PD and have been robustly replicated in this piece of work. Despite identifying variants associated with PD, this work also aims to update the risk estimates for these variants with a more reliable confidence interval than previously reported, which has now been made available in our Supplementary tables.

In addition to this replication, we looked into variants that did not reach 80% at OR = 2 (which was the limit of our power calculation tool), whilst generating ORs > 2 in our results. We included this list of variants, since it is very likely that these variants would pass 80% power at their respective ORs. Whilst many variants fit these criteria, five variants passed Bonferroni correction and are of greater interest to us: *LRRK2* p.R1441H (OR = 43.0), *GBA1* variants p.R502C (OR = 3.8) and p.R296Q (OR = 3.7), *LRRK2* variant p.L1795F (OR = 2.5) and *GBA1* variant p.D179H (OR = 3.3). All variants have previously been linked to PD.

*LRRK2* p.L1795F is a lesser-known and studied variant compared to p.R1441H and p.G2019S, which are both well-known damaging variants. *LRRK2* p.L1795F is located in the C-terminal of ROC B region and has been reported in a family with PD, however segregation was not shown^30^. We provide evidence for *LRRK2* p.L1795F as a genetic risk factor for PD with an estimated OR of 2.5. Interestingly, this variant was recently shown to have a functional effect providing more evidence for its pathogenicity^31^. *GBA1*, p.E365K, p.T408M, and p.N409S all have been previously identified via GWAS or similar approaches^8^, with p.E365K and p.T408M being risk factors for PD, and p.N409S being a risk factor for PD with an additional association in homozygous state with Gaucher disease. The other *GBA1* variants (p.D179H, p.R296Q, and p.R502C) are all associated with Gaucher disease in a bi-allelic state and were robustly associated with PD in this study. In the case of *GBA1* p.D179H, the association with PD was statistically evident with higher ORs and narrow 95% CIs: 23andMe reporting OR = 3.4 (95% CI: 2.2–5.2) and OR = 3.3 (95% CI:2.1–5.0, MAF = 0.0001) in the meta-analysis. However, it is worth noting that this variant is often on the same haplotype as p.E365K (MAF = 0.012)^32,33^ and indeed, when exploring their relationship in the UK Biobank data, we identified strong linkage disequilibrium (LD) with a perfect *D*’ value of 1 and a low *r*2 value 0.009 indicating significant associations between the alleles.

In our results section, we highlighted variants that meet the <80% power and OR > 2 criteria but only achieved *p* value < 0.05 significance. While we do not assert an immediate association of these variants with PD in our findings, their potential significance is underscored by substantial ORs and wide confidence intervals, indicative of their rare nature. These variants could potentially be regarded as points of interest for further investigation in future studies.

Very little is known about LRRK2 p.N238I and ClinVar categorizes this variant to be a variant of unknown clinical significance. Just like many other *GBA1* variants, the pathogenic variant *GBA1* p.V433L is commonly associated with PD and Gaucher’s disease, although reports on the National Center for Biotechnology Information dbSNP database are outdated and limited. And lastly, *SNCA* p.H50Q is a heavily discussed variant that is frequently associated with PD, however, its clinical significance is unclear. Many reports show an association with PD but if the variant is truly pathogenic remains questionable^25,27,34^.

Many variants, common or rare, are reported to be associated with PD, in fact the ClinVar database shows 4,320 results for the condition when searching for “Parkinson” as of January 1, 2023. Most well-powered variants with meta-analysis *p* value > 0.05 were of uncertain, unknown or unclear clinical significance, but quite a few were also previously associated with Parkinson’s (Supplementary Table 3). For example, six variants that were previously reported to be “pathogenic” or “likely pathogenic” did not show evidence of association with PD in our results and therefore these variants should be treated with caution. Those variants were: *POLG* p.467T (rs113994095), p.G737R (rs121918054), and p.G848S (rs113994098); *GLUD2* p.S498A (rs9697983), *PINK1* p.T313M (rs74315359), and *SNCB* p.P123H (rs104893937). However, due to our study design, results regarding autosomal recessive genes should be interpreted carefully.

We acknowledge that our analysis comes with some limitations. First, very large sample sizes are required to study rare variants. Investigating variants with low frequencies often results in unreliable high odds ratios with wide confidence intervals, suggesting that the population parameter is not confidently predicted. For example, *SNCA* p.A53T which is known to be causal for PD^35^, but given the extreme low frequency of this allele, it was not found in AMP-PD or UK Biobank data, and had a *p* value of 0.03 with a very wide risk estimate (OR = 38.0, 95% CI = 2.82–510.12) in the 23andMe dataset. This clearly shows a connection but not enough carriers to pass multiple test correction. Some pathogenic variants that passed significance at *p* < 0.05 but failed the multiple test correction were identified. *GBA1* p.V433L (rs80356769, meta-analysis: *P* = 1.6E-04, OR = 4.2, 95% CI = 1.98–8.8) was present in ClinVar as a pathogenic PD variant, but also the previously linked autosomal dominant *LRRK2* variant p.I2020T (rs35870237, *P* = 0.0004, OR = 21.4, 95% CI = 3.97–115.5) is considered pathogenic based on biological evidence^36^. A genome-wide linkage study in the Japanese Sagamihara family linked the PARK8 locus for the first time to familial parkinsonism on chromosome 12p11.2-q.13.1^37^. Subsequently, it was discovered that *LRRK2* mutations in this locus were associated with both familial and sporadic forms of PD^4,38^, propelling *LRRK2* into the spotlight as a key player in PD research. These variants showcase the complexities of rare variant analysis and although we used the largest sample size for PD yet, it still shows the limited power we have to identify robust risk estimates for very rare variants. We acknowledge that our dataset may not be representative of the general population, as it does not include all pathogenic PD variants. However, we generated updated statistics for many pathogenic variants in causal PD genes in our data set, which could be of value to the wider research community.

Second, an important aspect of rare and causal variants is the allele-dosage effect, since recessive genes are only causal in a homozygous or compound heterozygous state, which is especially important for PD since several genes are known to only be causal in a recessive state. Most of our statistical models used here are based on additive effects and since only summary-level data was available from 23andMe, we cannot accurately report results on autosomal recessive genes such as *PRKN* and *PINK1*. To highlight this further the *PRKN* p.R275W (rs34424986) variant is known to increase the risk for PD in a homozygous or compound heterozygous state^39^. In our analysis, this particular variant showed robust association with an overall OR of 1.3 in the meta-analysis. A preliminary analysis by 23andMe using a recessive model for *PRKN* p.R275W, showed that in a heterozygous state OR is at 1.4, whereas in a homozygous alternative state the OR is 6. This variant is under heavy debate for its association with PD risk in a heterozygous state and is likely only disease-causal if another damaging *PRKN* variant is on the other haplotype^40^.

Third, our analysis started with the selection of variants based on array genotype data of 23andMe and therefore, we missed variants that cannot be reliably imputed or are hard to genotype (such as *GBA1* p.L444P). This is a limitation that will be resolved with the availability of more sequencing data in the coming years and we would like to highlight that missing support for some variants in this analysis should not be interpreted as evidence against their role in PD but that this could simply be due to technical reasons such as variant selection process but also the lack of sufficient carrier numbers in our data used.

Finally, the lack of diversity is a critical challenge in genetic research and limits our insights and understanding of the disease and variants alone can have different effects in different populations. For example current sample sizes in 23andMe for African and Asian ancestry is under 400 cases^41^. However, initiatives such as the Genomic Aggregation Database (gnomAD) and the Global Parkinson’s Genetics Program (GP2) are making an active effort to increase the number of non-European underrepresented populations in genetic datasets^42^, so that in the near future, based on genetics, we can hopefully create a more representative picture of the disease.

In summary, we provide a robust list of 5 variants associated with PD and more reliable risk estimates. Additionally we provide a list of 8 variants that are strong candidates for their association with PD, based on their statistical output. A robust assignment of extremely rare high-risk and causal variants is crucial to better inform genetic counseling efforts, but several complications arise when working with very low allele frequency variants. Larger data sets and more suitable tools are a critical requirement to further our understanding of rare variants, and we hope this work can be a useful tool for the wider PD community.

## Methods

### Gene and variant selection

For variant selection, we annotated PD GWAS summary statistics from 23andMe using ANNOVAR^43^ including gene-based and ClinVar (version clinvar_20220320) annotation. We investigated variants with a minor allele frequency (MAF) of less than 5% to restrict our focus to rare variants (MAF < 1%) while retaining known risk variants such as *GBA1* p.E365K (MAF = 1.4% in gnomAD for non-finnish Europeans). Only biallelic exonic or splicing variants related to “Parkinson’s disease” and/or “Lewy body dementia” were kept. We selected all coding variants from monogenic PD genes confirmed by the MDS Task Force^9,10^, such as *LRRK2, SNCA*, and *VPS35* (classical parkinsonism, dominant), *DJ1, PRKN, PINK1* (early-onset parkinsonism, recessive) and genes previously published in the literature: *ATP13A2*, *DNAJC13*, *DNAJC6*, *EIF4G1*, *FBXO7*, *GBA1*, *GIGYF2*, *HTRA2*, *LRP10*, *PARK7*, *PLA2G6*, *POLG*, *SYNJ1*, *TMEM230*, *UCHL1*, and *VPS13C*^1^. We removed “benign” and “likely benign” variants in the *GBA1* gene but also in genes identified in the keyword search “Parkinson’s disease” and “Lewy body dementia”. Synonymous, *PINK1*-antisense (AS), and *UCHL*-AS variants were also excluded from the dataset. The process of variant selection based on 23andMe data is summarized in Fig. 1.

### 23andMe data

A rare variant association analysis was conducted using 3,090,507 unrelated people (3,065,473 controls, 25,034 PD cases). Participants provided informed consent and volunteered to participate in the research online, under a protocol approved by the external AAHRPP-accredited IRB, Ethical & Independent (E&I) Review Services. As of 2022, E&I Review Services is part of Salus IRB (https://www.versiticlinicaltrials.org/salusirb). Related individuals were removed, defined as >700 cM that are identical-by-descent (~20% of the genome or approximately first cousins in an outbred population)^44^. Ancestry composition was performed as previously reported^45^, and to minimize confounding by ancestry, only individuals with predominantly European ancestry were used.

DNA extraction and genotyping were performed on saliva samples by the National Genetics Institute. Five Illumina-based genotyping platforms (v1, v2, v3, v4, and v5) were employed, each with varying numbers of custom SNPs and total SNPs. The samples had a minimum call rate of 98.5%.

The imputation panel combines two independent reference panels: the publicly available Human Reference Consortium (HRC) panel and a 23andMe reference panel that combines both internal and external cohorts. HRC reference panel: The publicly available HRC data were downloaded from the European Genome-Phenome Archive at the European Bioinformatics Institute (accession EGAD00001002729). The HRC data includes 27,165 samples. Variants were lifted to hg38 and excluded if their new positions were on a different chromosome. Variants were then re-phased using SHAPEIT4^46^. Finally, singletons were excluded. 23andMe reference panel: We selected 12,217 samples from multiple internal and external WGS datasets (see Supplementary files for more information).

All samples were aligned and duplicate marked using one of two very similar pipelines. Data was aligned to the GRCh38 reference genome. For recent sequencing datasets (after 01/01/2019), we did not re-process received CRAMS from the Broad Institute since they use a well-known public pipeline. For older datasets (before 01/01/2019), we re-aligned the data using an in-house pipeline which consisted of bwa mem 0.7.15-r1140 alignment, duplicate marking with samblaster v0.1.24, and no BQSR.

Variants were called in each individual sample using DeepVariant-0.8.0 9 to produce GVCFs. The GVCFs were then joint-called using GLnexus-1.2.3 10. The following quality controls were applied to variants: singletons were removed, genotypes with GQ < 20 were set to missing, variants with >20% missingness (after the GQ filter) were removed, variants with >30% excess heterozygosity were removed.

Finally, variants were phased using SHAPEIT4. It is worth noting that SHAPEIT4 imputed missing genotypes and produced a final panel without missingness. The final 23andMe reference panel included 12,217 samples and 82,078,539 variants (73,852,355 SNPs + 8,226,184 indels).

Using Beagle 5^47^, variant imputation was performed separately for the three sets of variants: 1) HRC only, 2) 23andMe only, and 3) both HRC and 23andMe). Variants found only in the HRC panel were imputed using the 17,165 HRC panel individuals. Variants found only in the 23andMe panel were imputed using the 12,217 23andMe panel individuals. Variants found on both panels were imputed using the 36,898 individuals from the union of both panels. Imputation was performed independently for each genotyped platform.

Association test results were computed by logistic regression assuming additive allelic effects. Covariates for age, sex, and the top five genetic principal components (PCs) were included to account for residual population structure, and indicators for genotype platforms to account for genotype batch effects. The association test *p* value reported was computed using a likelihood ratio test.

Genotyped SNPs were excluded that: 1) had a genotyping rate <90%, 2) were only genotyped on the “v1” or “v2” 23andMe genotyping array, 3) were found on the mitochondrial chromosome or the Y-chromosome, 4) failed a test for parent-offspring transmission (*p* < 10^−20^), 5) had an association with genotype date (*p* < 10^–50^ by ANOVA of SNP genotypes against a factor dividing genotyping date into 20 roughly equal-sized buckets), 6) had a large sex effect (ANOVA of SNP genotypes, *r*2 > 0.1), or 7) had probes matching multiple genomic positions in the reference genome. For tests using imputed data, we used the imputed dosages rather than best-guess genotypes. Imputed SNPs were excluded that: 1) had imputation *r*2 < 0.5, or 2) had a significant batch effect between the “v4” and “v5” genotyping arrays (*p* < 10^−50^ by ANOVA of SNP dosage against genotyping array). Both genotyped and imputed SNPs were removed if: 1) available sample size was less than 20% of the total GWAS sample size, or 2) the logistic regression failed to converge (absolute value of the estimated log odds ratio or standard error >10). For more information on the 23andMe data set and its preparation, please refer to the supplementary files.

### AMP-PD and UK Biobank Data

Association results for the variants selected from the 23andMe dataset were generated from whole-genome sequencing data made available by the Accelerating Medicines Partnership—Parkinson’s disease Initiative (AMP-PD, https://amp-pd.org/) and the whole-exome sequencing data made available by the UK Biobank (https://www.ukbiobank.ac.uk/).

Whole genome sequencing data from multiple datasets, including the Parkinson’s Progression Markers Initiative (PPMI), the Parkinson’s Disease Biomarkers Program (PDBP), the Harvard Biomarker Study (HBS), BioFIND, SURE-PD3, and STEADY-PD3, were obtained as part of the Accelerating Medicines Partnership in Parkinson’s Disease (AMP-PD) initiative. The AMP-PD cohorts (PPMI, PDBP, HBS, BioFIND, SURE-PD3, and STEADY-PD3) followed the GATK Best Practices guidelines established by the Broad Institute’s joint discovery pipeline, as well as additional details provided elsewhere^48^. Data processing and quality control (QC) procedures have been described previously^48,49^. All individuals included in the analysis were of European ancestry through principal component analysis using HapMap3 European ancestry populations.

Exome sequencing data from a total of 200,643 individuals (OQFE dataset, field codes: 23151 and 23155) were obtained from the UK Biobank^50^. Data was filtered and processed as reported previously^51^. In brief, standard quality control measures were implemented to exclude non-European outliers. Individuals with close relationships (PI_HAT > 0.125) were excluded by randomly selecting one sample using PLINK (v1.9^52^). The exome sequencing data underwent standard filtering procedures based on suggested parameters outlined in previous UK Biobank studies^53^. Phenotype data from the UK Biobank included ICD10 codes (field code: 41270), PD (field code: 131023), parental and maternal illnesses (field codes: 20107 and 20110), parkinsonism (field code: 42031), dementia (field code: 42018), genetic ethnic grouping (field code: 22006), year of birth (field code: 34), and age of recruitment (field code: 21022). Cases were defined as individuals identified with PD based on the provided field code, while proxy-cases were defined as individuals with a parent or sibling with PD, as previously reported^8^. Controls were filtered to exclude individuals with an age of recruitment less than 59 years, reported nervous system disorders (Category 2406), a parent with PD or dementia (field codes: 20107 and 20110), and any reported neurological disorder (field codes: Dementia/42018, Vascular dementia/42022, FTD/42024, ALS/42028, Parkinsonism/42030, PD/42032, PSP/42034, MSA/42036).

Association results for the variants selected from the 23andMe dataset were generated from whole-genome sequencing data made available by the Accelerating Medicines Partnership - Parkinson’s disease Initiative (AMP-PD, https://amp-pd.org/) and the whole-exome sequencing data made available by the UK Biobank (https://www.ukbiobank.ac.uk/).

### Statistical analyses

We used three different data sets, including summary statistics from 23andMe and sequencing data from AMP-PD and UK Biobank. All data used genome build GRCh38. PLINK (v1.9^52^) was used to extract the variants identified in 23andMe data from AMP-PD and UK Biobank. To generate the association files for AMP-PD and UK Biobank, we then used RVTests^54^ for single variant association testing, using sex, and PC 1 to 5 as covariates for AMP-PD, excluding age since this dataset is a combination of several cohorts and therefore co-linearity exists between cohorts and reported ages. Genetic sex, age at recruitment, Townsend score, and PC 1 to 5 were used as covariates for UK Biobank. We conducted a fixed-effect inverse variance-weighted meta-analysis with the summary statistics, using METAL (version 2020-05-05^55^). Results were annotated using ANNOVAR, refGene, avsnp150, and clinvar_20220320^43^. Forest plots were generated using the rmeta (version 3.0) and metafor (version 3.8-1) packages in R. Power calculations were conducted using the R (v. 3.6) package genpwr (version 1.0.4), a power and sample size calculator for genetic association studies which allows for misspecification of the model of genetic susceptibility^56^. This package allows the assessment of allele frequencies as low as 1E-9 at OR = 2, which reduces the number of variants we worked with in the actual analysis. We used an additive model with an alpha value of 0.05. Since we used an additive model, it is important to note that we had less power to detect recessive associations in our analysis.

### Ethics statement

Each contributing study abided by the ethics guidelines set out by their institutional review boards, and all participants gave written informed consent to participate in both their initial cohorts and subsequent studies. The research used was deemed “not human subjects research” by the NIH Office of IRB Operations and stated that no IRB approval is required. Studies that are conducted on de-identified human genetics are waived ethical approval by the NIH Intramural IRB, as they are considered non-human subjects research. VP and CB take final responsibility for the decision to submit the paper for publication.

### Reporting summary

Further information on research design is available in the Nature Research Reporting Summary linked to this article.

### Supplementary information

Supplementary figures and tables

reporting summary

## Data Availability

All AMP-PD (https://amp-pd.org/) and UK Biobank (https://www.ukbiobank.ac.uk/) data is available via application on their websites, and 23andMe summary statistics are available via application at https://research.23andme.com/dataset-access/.

## Code availability

All code used for this analysis is available in the GitHub repository that can be found here: https://github.com/neurogenetics/23andme_rare_variants. All authors and the public can access the statistical programming code used in this project for the analyses and results generation.
