# Supplementary material for: Analysis of rare Parkinson’s disease variants in millions of people
Source: NPJ Parkinsons Dis. 2024 Jan 8;10:11. doi: 10.1038/s41531-023-00608-8 (PMC10774311; doi:10.1038/s41531-023-00608-8)
Supplement: Supplementary file 1 — Supplementary figures and tables [file 41531_2023_608_MOESM1_ESM.pdf]

Content overview of supplementary figures and tables					
**Name**	**Abbreviation**	**Content**	**Status**	**Note**	**Last update**
Supplementary Figure 1	SF1	Venn diagram showcasing variant overlap across all three studies	Done		Jul-23
Supplementary Table 1	ST1	ClinVar annotations of 669 variants	Done		Jul-23
Supplementary Table 2	ST2	List of 669 variants in 32 genes	Done		Jul-23
Supplementary Table 3	ST3	149 variants with sufficient power at OR=2 and not significant (p-value>0.05)	Done		Aug-23
Supplementary Table 4	ST4	23andMe summary statistics	Done		Jul-23
Supplementary Table 5	ST5	UKBiobank summary statistics	Done		Jul-23
Supplementary Table 6	ST6	AMP-PD summary statistics	Done		Jul-23
Supplementary Table 7	ST7	Meta-analysis sumary statistics	Done		Jul-23
Supplementary Table 8	ST8	Meta-analysis with annotations, statistics, penetrance, power		Edited for revision. NOTE: Wide CIs and large ORs for variants with little enrichment in data need to be interpreted/viewed carefully.	Aug-23
Supplementary Table 9	ST9	All meta-analysis variants with gnomAD and meta-analysis frequencies	Done		Aug-23
Supplementary Table 10	ST10	Meta-analysis variants and their counts, and MAC counts	Done	Added for revision. Caution: MACs are estimates, since for 23andMe only dosage information was available for imputed variants.	Aug-23

Supplementary Figure 1: Venn diagram showcasing variant overlap across all three studies.

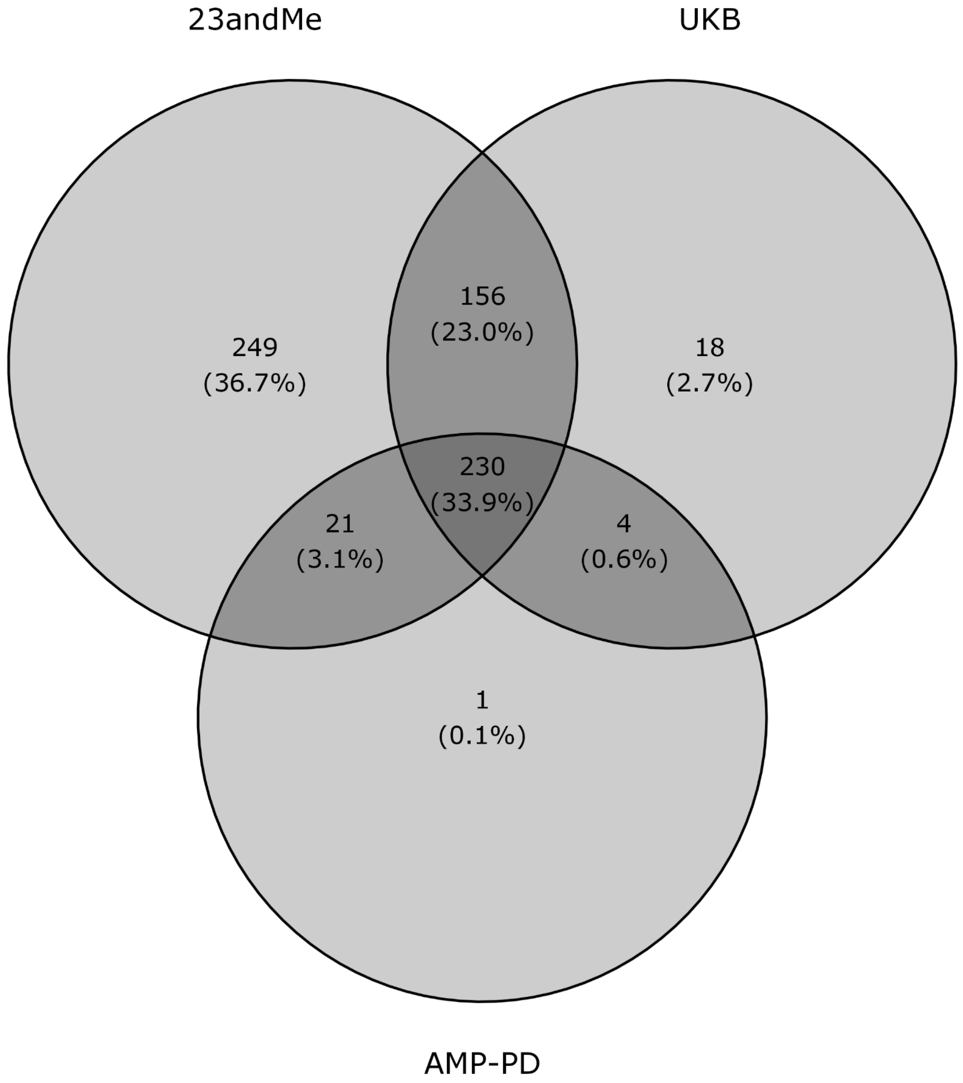

Supplementary Table 1: ClinVar annotations of 669 variants.

A) Overview clinical significance according to ClinVar for all variants used in this analysis.

	Number of variants	Percentage
**No information**	227	33.9
**Uncertain significance**	149	22.3
**Conflicting interpretations of pathogenicity**	94	14.1
**Pathogenic**	62	9.3
**Benign**	48	7.1
**Likely pathogenic**	30	4.5
**Likely benign**	21	3.1
**Pathogenic/Likely pathogenic**	17	2.5
**Benign/Likely benign**	13	1.9
**Risk factor**	5	0.7
**Conflicting interpretations of pathogenicity/risk factor**	1	0.2
**Pathogenic/likely pathogenic/risk factor**	1	0.2
**Pathogenic/risk factor**	1	0.2
Total	669	100

Supplementary Table 2: List of 669 variants in 32 genes.

Gene	n	perc	
POLG	87	13	
LRRK2	86	12.9	
VPS13C	83	12.4	
GBA1	57	8.5	
PLA2G6	55	8.2	
PINK1	38	5.7	
DNAJC13	37	5.5	
PRKN	35	5.2	
ATP13A2	34	5.1	
EIF4G1	33	4.9	
GIGYF2	20	3	
LRP10	19	2.8	
SYNJ1	14	2.1	
DNAJC6	11	1.6	
FBXO7	10	1.5	
TMEM230	9	1.3	
PARK7	6	0.9	
HTRA2	5	0.7	
SLC6A3	5	0.7	
VPS35	5	0.7	
TNR	4	0.6	
UCHL1	3	0.4	
MAPT	2	0.3	
SNCA	2	0.3	
TNK2	2	0.3	
FBXO7;FBXO7	1	0.1	
GLUD2	1	0.1	
MRE11	1	0.1	
RAB39B	1	0.1	

SNCAIP	1	0.1	
SNCB	1	0.1	
TRPM7	1	0.1	

Supplementary Table 3: 149 variants with sufficient power at OR=2 and not significant (p-value>0.05).

MarkerName	avsnp150	VariantName	Ref	Alt	CLNDN	PD_known	CLNSIG	MAF_23	MAF_NFE	MAF_case	MAF_ctrls	Power_at_alpha(	Effect	StdErr	P-value	OR	L95	U95
chr3:195867623	rs112384084	TNK2_R877H	C	T	Parkinson_disease|not_s	PD_known	Conflicting_interpretations_of_pathogenicity	0.017196233	0.017	0.024832333	0.017155231	1	-0.0111	0.0313	0.7225	1.011161834	0.950993549	1.075136898
chr2:232791124	rs148277228	GIGYF2_D349E	T	A	.	Not_PD_known	.	0.00057051	0.001	0.000756266	0.000985052	0.96133828	-0.1359	0.1863	0.4657	1.145567331	0.795131316	1.650450036
chr6:161785877	rs150562946	PRKN_R256C	G	A	Neoplasm_of_ovary|Parl	PD_known	Uncertain_significance	0.000815874	9.00E-04	0.001266101	0.000584162	0.993708479	-0.2652	0.164	0.1059	1.303691688	0.945312234	1.797937186
chr6:162443371	rs148990138	PRKN_P37L	G	A	Parkinson_disease_2|nc	PD_known	Conflicting_interpretations_of_pathogenicity	0.000341303	2.00E-04	0.000579119	0.000268485	0.821938061	0.0896	0.2011	0.6559	1.093736702	0.73745073	1.622155791
chr1:65392454	rs145329294	DNAJC6_C441S	T	A	Parkinson_disease_19a	PD_known	Conflicting_interpretations_of_pathogenicity	0.000659876	6.00E-04	0.001296316	0.001157464	0.979681835	-0.0207	0.167	0.9012	1.020915731	0.735930201	1.416260575
chr3:184328741	rs144059151	EIF4G1_M1356T	T	C	.	Not_PD_known	.	0.000413179	5.00E-04	0.001512696	0.001453059	0.887157546	0.0198	0.2206	0.9283	1.01999732	0.661942887	1.571728548
chr15:89318595	rs2307441	POLG_E1143G	T	C	Association_with_valpro	Not_PD_known	Benign	0.043663397	0.0434	0.055697768	0.042580272	1	0.0318	0.0202	0.1154	1.032311022	0.992238279	1.074002152
chr3:184322040	rs112545306	EIF4G1_P486S	C	T	.	Not_PD_known	.	0.001075387	0.0015	0.003457217	0.002881419	0.999193846	0.0993	0.1388	0.4745	1.104397569	0.841349608	1.449687477
chr22:38132952	rs149653983	PLA2G6_T319M	G	A	Parkinson_disease_14|F	PD_known	Uncertain_significance	0.000466984	3.00E-04	0.001296316	0.000911173	0.92094843	-0.1515	0.2058	0.4617	1.163578302	0.777347341	1.741711064
chr3:132502299	rs139620588	DNAJC13_R1516H	G	A	.	Not_PD_known	.	0.003634315	0.0037	0.007995678	0.008325328	1	0.0448	0.0687	0.5147	1.045818675	0.914066457	1.196561468
chr2:232794835	rs116074753	GIGYF2_N457T	A	C	Parkinson_disease_11	PD_known	risk_factor	0.000618382	6.00E-04	0.001306076	0.001241644	0.9725334	0.1876	0.2307	0.4161	1.206350878	0.767534384	1.896048531
chr1:20644665	rs139226733	PINK1_M318L	A	T	Parkinson_disease_6,_a	PD_known	Uncertain_significance	0.001067776	0.0016	0.001100711	0.000722864	0.99914329	-0.0308	0.1432	0.8297	1.031279227	0.778900476	1.365433554
chr12:40323256	rs35602796	LRRK2_M1869T	T	C	Parkinson_disease_8,_a	PD_known	Uncertain_significance	0.000414089	3.00E-04	0.000400465	0.000372365	0.887823842	0.1448	0.2193	0.5092	1.155808385	0.751993198	1.776469557
chr15:89330250	rs202088074	POLG_V229G	A	C	.	Not_PD_known	.	0.000714481	NA	NA	NA	0.986435862	-0.0306	0.2941	0.917	1.031072992	0.579357238	1.834984437
chr15:89330184	rs113994094	POLG_T251I	G	A	Abnormality_of_the_ner	Not_PD_known	Conflicting_interpretations_of_pathogenicity	0.002281261	0.0023	0.003196052	0.002331736	0.999999979	0.0513	0.0846	0.5441	1.052638638	0.89179767	1.242488222
chr4:41261759	rs150601238	UCHL1_M124L	A	C	Parkinson_disease_5|nc	PD_known	Conflicting_interpretations_of_pathogenicity	0.001436651	0.0014	0.002918288	0.003595793	0.999960757	0.0146	0.1175	0.9013	1.014707101	0.805977059	1.277493557
chr15:61915712	rs72747885	VPS13C_I2789T	A	G	not_provided	Not_PD_known	Benign	0.010065019	0.0108	0.020633034	0.019235979	1	0.0592	0.0412	0.1505	1.060987417	0.978678585	1.150218587
chr5:1432618	rs71653633	SLC6A3_L167F	G	A	Tobacco_addiction,_sus	PD_known	Conflicting_interpretations_of_pathogenicity	0.001277995	0.0019	0.002592913	0.002684266	0.999849142	0.0132	0.1226	0.9142	1.013287505	0.796844282	1.288522225
chr20:5069196	rs570474631	TMEM230_P126T	G	T	.	Not_PD_known	.	0.001894236	0.0019	0.005725397	0.005664327	0.999999291	0.0643	0.1152	0.5766	1.066412274	0.850873338	1.336550445
chr3:132522838	rs145242123	DNAJC13_T1895M	C	T	not_provided	Not_PD_known	Likely_benign	0.002448976	0.0036	0.008210003	0.006132703	0.999999995	-0.047	0.0859	0.5838	1.048122009	0.885711502	1.240313289
chr15:89327201	rs113994095	POLG_A467T	C	T	POLG-related_condition	Not_PD_known	Pathogenic	0.001178722	7.00E-04	0.001568425	0.000956035	0.999655031	0.0332	0.14	0.8127	1.03375727	0.785684474	1.360156817
chr22:38169326	rs147948449	PLA2G6_S34L	G	A	Neurodegeneration_with	PD_known	Uncertain_significance	0.000489464	6.00E-04	0.001944474	0.001650002	0.932095525	0.0197	0.1934	0.9187	1.019895326	0.698120189	1.489981941
chr2:232747740	rs72554080	GIGYF2_N56S	A	G	Parkinson_disease_11	PD_known	risk_factor	0.000532225	4.00E-04	0.000597222	0.000454292	0.949406264	-0.5189	0.2776	0.06159	1.680178437	0.97511877	2.895031524
chr1:17000494	rs145515028	ATP13A2_A244V	G	A	Kufor-Rakeb_syndrome|	Not_PD_known	Conflicting_interpretations_of_pathogenicity	0.000769239	3.00E-04	0.000756185	0.000221637	0.991015393	-0.1577	0.142	0.2666	1.170814898	0.886370716	1.546539725
chr3:132475009	rs149121829	DNAJC13_S790Y	C	A	not_provided	Not_PD_known	Likely_benign	0.007230685	0.0068	0.021501891	0.021234665	1	0.0574	0.054	0.2875	1.059079357	0.9527145	1.177319212
chr22:38120886	rs143826762	PLA2G6_G539S	C	T	Iron_accumulation_in_br	PD_known	Conflicting_interpretations_of_pathogenicity	0.001222253	0.0017	0.003348817	0.003768009	0.999759631	-0.1113	0.1163	0.3387	1.117730176	0.889898382	1.403891467
chr14:22875924	rs138170865	LRP10_G326S	G	A	.	Not_PD_known	.	0.001105133	0.0012	0.001296316	0.001206718	0.99936738	-0.0251	0.1501	0.8674	1.025417657	0.764069901	1.376158608
chr15:89330106	rs138929605	POLG_H277L	T	A	Progressive_sclerosing_	Not_PD_known	Conflicting_interpretations_of_pathogenicity	0.000606196	3.00E-04	0.001204217	0.000620175	0.970016439	-0.0613	0.1773	0.7297	1.063217832	0.75110637	1.505022728
chr15:89333627	rs28567406	POLG_Q43R	T	C	Seizures|Progressive_sc	Not_PD_known	Benign	0.003384898	0.0025	0.005837838	0.006162341	1	-0.0574	0.0754	0.4463	1.059079357	0.913580303	1.22775095
chr1:16986335	rs377703085	ATP13A2_R1043C	G	A	Kufor-Rakeb_syndrome|	Not_PD_known	Conflicting_interpretations_of_pathogenicity	0.000437842	8.00E-04	0.00162057	0.00219174	0.904006672	0.1176	0.194	0.5445	1.124794104	0.769018694	1.645163876
chr1:16986101	rs41273151	ATP13A2_T1121S	T	A	Kufor-Rakeb_syndrome|	Not_PD_known	Benign/Likely_benign	0.005611755	0.0056	0.012859304	0.013796162	1	0.0023	0.0574	0.9678	1.002302647	0.895651404	1.121653572
chr2:232847564	rs143181034	GIGYF2_Q1226P	A	C	.	Not_PD_known	.	0.002822578	NA	NA	NA	1	-0.174	0.1306	0.1827	1.190055566	0.921294069	1.53722063
chr15:89326947	rs61752783	POLG_G517V	C	A	Seizures|Toe_walking|Id	Not_PD_known	Conflicting_interpretations_of_pathogenicity	0.007377997	0.0065	0.010729635	0.007525376	1	0.0309	0.0487	0.5254	1.03138236	0.937487364	1.134681506
chr12:40351680	rs72547981	LRRK2_D2175H	G	C	Parkinson_disease_8,_a	PD_known	Uncertain_significance	0.00079698	NA	0.000333444	0.001534281	0.992728201	0.5511	0.5085	0.2785	1.735160645	0.640465515	4.700928301
chr22:38169336	rs150024227	PLA2G6_D31N	C	T	Neurodegeneration_with	PD_known	Uncertain_significance	0.000546336	1.00E-04	0.000864211	0.001083584	0.95416274	-0.1128	0.198	0.5688	1.119408029	0.759359473	1.650172784
chr1:16985990	rs76298930	ATP13A2_V1158M	C	T	Kufor-Rakeb_syndrome	Not_PD_known	Benign	0.000496447	1.00E-04	0.000432105	0.000270889	0.935253998	-0.1097	0.1564	0.4831	1.115943237	0.821318749	1.51625579
chr15:61868702	rs116228685	VPS13C_R3607H	C	T	.	Not_PD_known	.	0.000466645	5.00E-04	0.002052501	0.001748553	0.920767982	-0.1913	0.1843	0.2993	1.210822644	0.843725563	1.737640225
chr2:232847399	rs72554081	GIGYF2_H1171R	A	G	.	Not_PD_known	.	0.00209862	0.0034	0.004969749	0.004063639	0.999999888	0.0346	0.1019	0.7345	1.035205544	0.847788572	1.264053979
chr15:61950998	rs11629598	VPS13C_I1495V	T	C	not_provided	Not_PD_known	Benign	0.077637793	0.0846	0.122192607	0.104706513	1	0.0168	0.0153	0.2721	1.016941914	0.986898581	1.047899831
chr2:74530221	rs150047108	HTRA2_L72P	T	C	Parkinson_disease_13|n	PD_known	Conflicting_interpretations_of_pathogenicity	0.003193612	0.0025	0.008534082	0.008398394	1	-0.1007	0.0773	0.1929	1.105944809	0.950461142	1.286863677
chr21:32665968	rs147929290	SYNJ1_I746T	A	G	Parkinson_disease_20,_	PD_known	Conflicting_interpretations_of_pathogenicity	0.001411014	0.0017	0.001653136	0.001363453	0.999951153	-0.0953	0.1125	0.3969	1.099988802	0.882320421	1.371355957
chr15:89328996	rs2307437	POLG_P324S	G	A	Progressive_sclerosing_	Not_PD_known	Conflicting_interpretations_of_pathogenicity	0.000545124	6.00E-04	0.001296316	0.001256034	0.953772427	0.1874	0.2113	0.3751	1.206109632	0.797121632	1.824941625
chr3:132525718	rs138693725	DNAJC13_A2057S	G	T	.	Not_PD_known	.	0.001096487	8.00E-04	0.002160527	0.002462751	0.999321112	0.0903	0.1349	0.5035	1.094502585	0.840209511	1.425758567
chr20:5106224	rs148033002	TMEM230_I62M	T	C	.	Not_PD_known	.	0.001344351	0.0011	0.001188418	0.002068609	0.999913837	-0.1566	0.1325	0.2371	1.169527709	0.902036765	1.516340703
chr3:195868341	rs201407161	TNK2_V638M	C	T	Parkinson_disease|Auto	PD_known	Conflicting_interpretations_of_pathogenicity	0.000969291	0.0014	0.002485143	0.001674629	0.998107638	-0.213	0.1385	0.1241	1.237384651	0.943215968	1.623298191
chr22:38126390	rs201801144	PLA2G6_M470V	T	C	Infantile_neuroaxonal_dy	Not_PD_known	Uncertain_significance	0.000525552	7.00E-04	0.001080264	0.000837294	0.947007339	0.0272	0.22	0.9016	1.027573297	0.667644121	1.581541493
chr1:16993627	rs74058364	NA	A	C	Kufor-Rakeb_syndrome	Not_PD_known	Uncertain_significance	0.014196694	1.00E-04	NA	NA	1	0.1403	0.1547	0.3646	1.150618933	0.849665956	1.558169913
chr22:38145447	rs141825182	PLA2G6_R139H	C	T	Neurodegeneration_with	PD_known	Conflicting_interpretations_of_pathogenicity	0.00067544	7.00E-04	0.000972447	0.001256034	0.981875318	0.1544	0.1693	0.3619	1.166957576	0.837421291	1.626170721
chr3:184327609	rs35629949	EIF4G1_P1230A	C	G	not_provided	Not_PD_known	Likely_benign	0.005021393	0.0037	0.009510429	0.009510669	1	0.0312	0.0605	0.6063	1.031691822	0.916328825	1.161578667
chr15:61962790	rs3784635	VPS13C_I1132V	T	C	not_provided	Not_PD_known	Benign	0.025377766	0.0409	0.026085672	0.025210965	1	-0.0558	0.0298	0.06099	1.057386185	0.997395398	1.120985266
chr3:184315839	rs759151568	EIF4G1_S15P	T	C	.	Not_PD_known	.	0.000594318	6.00E-04	0.00156041	0.001347332	0.967357528	-0.1479	0.2047	0.47	1.159396951	0.776225662	1.731714572
chr1:20645615	rs55831733	PINK1_A339T	G	A	Parkinson_disease_6,_a	PD_known	Conflicting_interpretations_of_pathogenicity	0.001025844	6.00E-04	0.002034539	0.000943203	0.998797301	0.0044	0.143	0.9757	1.004409694	0.758903994	1.329336572
chr15:89327198	rs145843073	POLG_N468D	T	C	Intellectual_disability|Sei	Not_PD_known	Conflicting_interpretations_of_pathogenicity	0.000524262	8.00E-04	0.001728608	0.001477651	0.946531514	-0.0805	0.2274	0.7235	1.083828847	0.694055049	1.692495387
chr1:175406219	rs147204644	TNR_T166A	T	C	Parkinson_disease|not_p	PD_known	Conflicting_interpretations_of_pathogenicity	0.00511309	0.0051	0.009507347	0.010323248	1	0.0744	0.0577	0.1975	1.077237614	0.962046974	1.2062206
chr1:16990276	rs200924194	ATP13A2_Q750E	G	C	Kufor-Rakeb_syndrome|	Not_PD_known	Uncertain_significance	0.000679567	6.00E-04	0.000834425	0.000495652	0.982417986	-0.2509	0.178	0.1585	1.28518156	0.906667037	1.821717978
chr15:89323426	rs113994097	POLG_W748S	C	G	.	Not_PD_known	.	0.000565724	6.00E-04	0.00112202	0.001140535	0.959992583	0.0719	0.1826	0.6936	1.074547884	0.751265621	1.536943955
chr1:65366050	rs61757223	DNAJC6_M76L	A	T	Parkinson_disease_19a	PD_known	Uncertain_significance	0.000724256	6.00E-04	0.001404343	0.001157521	0.98739111	0.2137	0.1567	0.1727	1.238251124	0.91079995	1.683427679
chr15:89319318	rs201204229	POLG_V1005G	A	C	not_provided	Not_PD_known	Uncertain_significance	0.000599086	4.00E-04	NA	NA	0.968449426	-0.2456	0.2997	0.4124	1.278388116	0.710481763	2.300236629

chr1:16996298	rs149372969	ATP13A2_L432V	G	C	Kufor-Rakeb_syndrome|	Not_PD_known	Conflicting_interpretations_of_p athogenicity	0.001209371	0.0015	0.00356487	0.003743381	0.999732437	-0.0636	0.1196	0.5945	1.065666047	0.842976667	1.347183343
chr14:22877070	rs142153001	LRP10_R562H	G	A	.	Not_PD_known	.	0.008213293	0.0095	0.018478496	0.017420658	1	-0.0521	0.0458	0.2545	1.053481085	0.963032615	1.152424518
chr3:184322583	rs111924994	EIF4G1_A550P	G	C	not_provided	Not_PD_known	Likely_benign	0.001455163	9.00E-04	0.003457217	0.003300167	0.999966526	-0.0846	0.1126	0.4522	1.088281667	0.872758844	1.357026623
chrX:121049176	rs9697983	GLUD2_S498A	T	G	Parkinson_disease,_late	PD_known	Pathogenic	0.022863518	0.0254	0.022435041	0.022435041	1	0.0182	0.0237	0.443	1.018366629	0.972143356	1.066787718
chr3:132499777	rs61748103	DNAJC13_R1462H	G	A	not_specified|not_provid	Not_PD_known	Benign	0.010368491	0.0124	0.025513514	0.022912047	1	-0.0477	0.0426	0.2626	1.048855951	0.9648371	1.140191237
chr15:61910283	rs10851704	VPS13C_S2913N	C	T	not_provided	Not_PD_known	Benign	0.474297271	0.4356	0.528360392	0.477434848	1	0.0076	0.0091	0.4049	1.007628953	0.98981621	1.025762255
chr15:89323460	rs121918054	POLG_G737R	C	G	Seizures|Progressive_sc	Not_PD_known	Pathogenic/Likely_pathogenic	0.001802528	0.0019	0.002505781	0.001802322	0.999998393	0.0135	0.0965	0.8891	1.013591536	0.83891994	1.224631522
chr3:132522950	rs147575919	DNAJC13_D1932E	T	G	.	Not_PD_known	.	0.000722064	7.00E-04	0.001836448	0.001748596	0.987182645	-0.1716	0.1748	0.3261	1.187202857	0.842814831	1.672313505
chr15:61920571	rs139993005	VPS13C_H2380R	T	C	not_provided	Not_PD_known	Benign	0.003944486	0.0045	0.00713051	0.005984632	1	0.068	0.0691	0.3253	1.070365308	0.934787545	1.225606716
chr22:32498414	rs141286570	FBXO7_V485I	G	A	Parkinson_disease_15|n	PD_known	Uncertain_significance	0.000722593	9.00E-04	0.002808685	0.001871737	0.987233278	-0.2085	0.1681	0.215	1.23182893	0.886055225	1.712537177
chr1:17000272	rs56367069	ATP13A2_R289Q	C	T	Kufor-Rakeb_syndrome|	Not_PD_known	Benign/Likely_benign	0.017024194	0.0196	0.020936069	0.017089812	1	0.0385	0.0318	0.2267	1.039250728	0.976453645	1.106086379
chr20:5069288	rs186368464	TMEM230_R95H	C	T	.	Not_PD_known	.	0.000907719	0.0012	0.002916712	0.002536508	0.996919474	-0.0268	0.1657	0.8717	1.02716235	0.742322124	1.421300077
chr15:61931104	rs78071599	VPS13C_E2008D	C	G	.	Not_PD_known	.	0.018998384	0.0194	0.042063149	0.043260451	1	0.0237	0.0303	0.4357	1.023983077	0.964941308	1.086637428
chr1:65386886	rs201840876	DNAJC6_M300T	T	C	not_provided	Not_PD_known	Uncertain_significance	0.000324073	1.00E-04	0.000864211	0.000541779	0.801879866	0.0375	0.256	0.8837	1.038211997	0.628600097	1.714737487
chr15:61991096	rs143639809	NA	T	C	not_provided	Not_PD_known	Benign	0.000996965	0.0021	0.0015152	0.000954011	0.998483124	-0.2371	0.1399	0.09005	1.267567868	0.963575918	1.667464151
chr1:65385740	rs146050826	DNAJC6_A220T	G	A	not_provided	Not_PD_known	Uncertain_significance	0.000621921	8.00E-04	0.000864211	0.00068957	0.973226462	-0.1236	0.1992	0.5349	1.131563155	0.765801715	1.672019204
chr3:184323468	rs111396765	EIF4G1_A718P	G	C	not_provided	Not_PD_known	Likely_benign	0.001287533	0.0012	0.004321054	0.003546449	0.999860756	-0.027	0.1193	0.8207	1.027367803	0.813159501	1.298004389
chr22:38113621	rs141777179	PLA2G6_V690I	C	T	Iron_accumulation_in_br	Not_PD_known	Uncertain_significance	0.000741389	0.0018	0.000869837	0.000538278	0.988911866	-0.1036	0.1845	0.5743	1.109156703	0.77257978	1.592364471
chr22:38132881	rs11570680	PLA2G6_A343T	C	T	PLA2G6-associated_neu	Not_PD_known	Benign/Likely_benign	0.012910068	0.0134	0.025832252	0.024675229	1	-0.0659	0.037	0.0749	1.0681199	0.993401864	1.148457801
chr21:32664963	rs145978776	SYNJ1_D791Y	C	A	Parkinson_disease_20,_	PD_known	Uncertain_significance	0.000331727	1.00E-04	0.001296456	0.001699256	0.811128034	-0.0503	0.1909	0.7922	1.051586525	0.723348611	1.528770779
chr15:89333723	rs765472726	POLG_G11D	C	T	.	Not_PD_known	.	0.000452995	2.00E-04	0.001080264	0.001452987	0.913190322	0.1363	0.197	0.489	1.14602565	0.77894098	1.686103086
chr6:161569358	rs72480423	PRKN_E310D	C	G	Parkinson_disease_2|nc	PD_known	Uncertain_significance	0.000337624	4.00E-04	0.000830607	0.000507778	0.817850264	-0.3665	0.2281	0.108	1.442676401	0.922584784	2.255960896
chr3:132528316	rs140537885	DNAJC13_L2170W	T	G	.	Not_PD_known	.	0.00454461	0.0058	0.00609738	0.004491301	1	-0.1108	0.0626	0.07663	1.11717145	0.988174478	1.263007776
chr1:16986554	rs201756175	ATP13A2_P1105L	G	A	Kufor-Rakeb_syndrome|	Not_PD_known	Conflicting_interpretations_of_p athogenicity	0.000653883	6.50E-05	0.000108026	0.000344768	0.978771643	0.14	0.1733	0.4191	1.150273799	0.819002617	1.615537955
chr14:22875363	rs28534929	LRP10_M139V	A	G	not_provided	Not_PD_known	Benign	0.000615478	7.00E-04	0.00137836	0.001173709	0.971951912	0.2157	0.1938	0.2657	1.240730104	0.848616417	1.814024758
chr1:17004708	rs376169767	ATP13A2_A154V	G	A	Kufor-Rakeb_syndrome|	Not_PD_known	Uncertain_significance	NA	5.00E-04	0	7.39E-05	0.936808247	1.1469	1.8134	0.5271	3.148417671	0.09005236	110.0752255
chr3:132466351	rs199541720	DNAJC13_D674A	A	C	.	Not_PD_known	.	0.000389278	8.00E-04	0.000756185	0.000788041	0.868341097	-0.0413	0.2186	0.8503	1.042164708	0.678985165	1.599603842
chr15:61922557	rs141890160	VPS13C_I2272T	A	G	.	Not_PD_known	.	0.000840724	0.001	0.001944474	0.002561135	0.99480579	0.091	0.144	0.5272	1.095269005	0.82593434	1.452432882
chr12:40363410	rs75695264	LRRK2_Y2346C	A	G	.	Not_PD_known	.	0.000384023	NA	0.000245278	0.000851464	0.863839958	-0.5517	0.7162	0.4411	1.736202054	0.426538776	7.067112637
chr15:89316763	rs3087374	POLG_Q1236H	C	A	Seizures|Progressive_sc	Not_PD_known	Benign/Likely_benign	0.080359167	0.0896	0.098482169	0.080946477	1	0.0085	0.0155	0.5824	1.008536228	0.978357631	1.039645719
chr15:61922408	rs12907567	VPS13C_V2322M	C	T	not_provided	Not_PD_known	Benign	0.079014133	0.084	0.098112166	0.079248642	1	-0.0153	0.0153	0.3156	1.015417644	0.985419342	1.046329159
chr15:61978646	rs140060073	VPS13C_Q757R	T	C	.	Not_PD_known	.	0.000758508	6.48E-05	0.000216053	0.000221637	0.990254585	-0.2008	0.1438	0.1625	1.222380271	0.922149427	1.620359438
chr15:61922527	rs138540886	VPS13C_S2282F	G	A	.	Not_PD_known	.	0.000402319	1.00E-04	0.000128107	0.000420521	0.878925938	0.3994	0.2984	0.1807	1.490929871	0.830718741	2.67584174
chr3:132499231	rs61748102	DNAJC13_A1421V	C	T	.	Not_PD_known	.	0.018825189	0.0193	0.024265861	0.018890982	1	0.0463	0.0308	0.1334	1.04738858	0.986030492	1.112564822
chr15:89330133	rs61752784	POLG_G268A	C	G	Seizures|Progressive_sc	Not_PD_known	Conflicting_interpretations_of_p athogenicity	0.004539149	0.004	0.007175409	0.004512395	1	0.0434	0.0635	0.4942	1.044355554	0.922138361	1.182771011
chr2:232794843	rs2289912	GIGYF2_P460T	C	A	not_provided	Not_PD_known	Benign	0.019302656	0.0216	0.02086171	0.019388477	1	0.0272	0.0332	0.4126	1.027573297	0.962848176	1.096663074
chr15:89328532	rs145289229	POLG_L392V	G	C	Seizures|Progressive_sc	Not_PD_known	Conflicting_interpretations_of_p athogenicity	0.001023628	0.0016	0.002273139	0.001166047	0.998775636	0.0971	0.103	0.3459	1.101970565	0.900522616	1.348482654
chr15:62023836	rs12595158	VPS13C_R153H	C	T	not_provided	Not_PD_known	Benign	0.022237398	0.0365	0.023136776	0.022099546	1	0.029	0.0313	0.3549	1.029424594	0.9681696	1.094555123
chr15:61934224	rs112236709	VPS13C_N1955H	T	G	not_provided	Not_PD_known	Benign	0.008076957	0.0069	0.008836191	0.008077726	1	-0.0258	0.0515	0.6173	1.026135701	0.927613611	1.135121848
chr15:61854543	rs115819951	VPS13C_I3726V	T	C	not_provided	Not_PD_known	Benign	0.005511863	0.0043	0.009940573	0.010469785	1	-0.0619	0.0573	0.2794	1.063855954	0.950841402	1.190303123
chr15:89319073	rs150233690	POLG_V1044A	A	G	Seizures|EEG_abnorma	Not_PD_known	Uncertain_significance	0.000782013	1.00E-04	0.001404343	0.001970104	0.991846973	-0.0055	0.1519	0.9712	1.005515153	0.746601279	1.354217775
chr1:175386035	rs140481433	TNR_T592A	T	C	Parkinson_disease	PD_known	Uncertain_significance	0.000586048	4.00E-04	0.001728422	0.002314986	0.96537483	-0.2244	0.1956	0.2513	1.251571548	0.853016831	1.836342828
chr15:61967438	rs3784634	VPS13C_R974K	C	T	not_provided	Not_PD_known	Benign	0.413990863	0.4316	0.444886208	0.420717094	1	-0.0098	0.0092	0.284	1.009848177	0.99180179	1.028222928
chr15:89330258	rs147282197	POLG_Q226H	C	G	Autistic_disorder|Seizure	Not_PD_known	Conflicting_interpretations_of_p athogenicity	0.000501454	7.00E-04	0.001512369	0.001379107	0.937434239	0.1244	0.1754	0.478	1.132468768	0.803013302	1.59709124
chr5:176621218	rs104893937	SNCB_P123H	G	T	Lewy_body_dementia	Not_PD_known	Likely_pathogenic	0.00049402	7.00E-04	0.000431224	0.000315116	0.934171925	-0.2231	0.3125	0.4753	1.249945562	0.677463231	2.306197352
chr20:5100832	rs143571424	TMEM230_R108C	G	A	.	Not_PD_known	.	0.002062707	0.0031	0.003999135	0.004015668	0.999999845	0.1481	0.1249	0.2358	1.159628853	0.907824682	1.48127618
chr1:7985019	rs71653622	PARK7_A179T	G	A	Parkinson_disease_7|nc	PD_known	Uncertain_significance	0.000641287	0.0013	0.001296316	0.000763528	0.976730487	-0.3631	0.189	0.05466	1.43777963	0.992686872	2.08243941
chr3:132484650	rs202127368	DNAJC13_T1082I	C	T	.	Not_PD_known	.	0.000566602	7.00E-04	0.00118829	0.001428431	0.9602397	-0.0645	0.1815	0.7221	1.066625578	0.747336296	1.52232687
chr12:40235634	rs33995463	LRRK2_L119P	T	C	Parkinson_disease_8,_a	PD_known	Conflicting_interpretations_of_p athogenicity	0.00249264	0.002	0.003111626	0.002425171	0.999999997	0.0165	0.0857	0.8474	1.016636877	0.859441946	1.202583308
chr6:161360169	rs55830907	PRKN_R402C	G	A	Parkinson_disease_2|nc	PD_known	Conflicting_interpretations_of_p athogenicity	0.001583714	0.0013	0.003349179	0.003029855	0.999988989	-0.1324	0.1134	0.2428	1.141564854	0.914055488	1.425701538
chr15:50586433	rs8042919	TRPM7_T1482I	G	A	Amyotrophic_lateral_scle	PD_known	Conflicting_interpretations_of_p athogenicity	0.105267067	0.1049	0.155800395	0.109962369	1	-0.0138	0.0134	0.3039	1.01389566	0.987613354	1.040877388
chr3:132494190	rs61748101	DNAJC13_E1291G	A	G	Parkinson_disease_21|n	PD_known	Conflicting_interpretations_of_p athogenicity	0.005826202	0.0047	0.011621053	0.011749855	1	-0.0699	0.0671	0.2976	1.072400936	0.940243871	1.22313349
chr15:61911844	rs115869241	VPS13C_S2904L	G	A	not_provided	Not_PD_known	Likely_benign	0.003339221	0.0032	0.006589608	0.005885975	1	-0.0585	0.074	0.4294	1.060244986	0.917098864	1.225734186
chr3:132478139	rs141952333	DNAJC13_R903K	G	A	.	Not_PD_known	.	0.002575526	0.0025	0.007672358	0.006502303	0.999999999	-0.0375	0.088	0.6701	1.038211997	0.873733386	1.233653387
chr3:132502295	rs55825559	DNAJC13_P1515S	C	T	not_specified|not_provid	Not_PD_known	Benign/Likely_benign	0.029150533	0.0262	0.038385496	0.028792441	1	0.0373	0.0253	0.1406	1.038004375	0.987787189	1.090774506
chr15:61854888	rs62007358	VPS13C_D3715Y	C	A	.	Not_PD_known	.	0.001256445	0.0016	0.003348817	0.003447851	0.999819345	-0.0011	0.1255	0.993	1.001100605	0.782798468	1.280281531
chr15:61915655	rs34060567	VPS13C_K2808R	T	C	not_provided	Not_PD_known	Benign	0.000673097	4.00E-04	0.00137836	0.000782473	0.981560189	-0.0552	0.1961	0.7783	1.056751944	0.719530761	1.55201797
chr1:16988161	rs55708915	ATP13A2_I902F	T	A	Kufor-Rakeb_syndrome|	Not_PD_known	Conflicting_interpretations_of_p athogenicity	0.001821771	9.00E-04	0.004429559	0.003447766	0.999998647	0.0082	0.1251	0.9476	1.008233712	0.788994444	1.28839338
chr3:184327622	rs2230570	EIF4G1_L1234P	T	C	not_specified|not_provid	Not_PD_known	Benign	0.023361123	0.0226	0.034403581	0.024301166	1	-0.0239	0.0275	0.3839	1.024187894	0.970445534	1.080906456
chr2:232844373	rs144086186	GIGYF2_S1035C	C	G	.	Not_PD_known	.	0.001950085	0.0021	0.002592633	0.004260559	0.999999569	-0.1218	0.1153	0.2909	1.129528174	0.901055883	1.41593204
chr15:89321842	rs41549716	POLG_Y831C	T	C	Seizures|Progressive_sc	Not_PD_known	Conflicting_interpretations_of_p athogenicity	0.009691849	0.0134	0.014147663	0.010094553	1	-0.0286	0.0434	0.5106	1.029012907	0.945100508	1.120375615
chr22:32475378	rs9621461	FBXO7_G6E	G	A	not_provided	Not_PD_known	Benign	0.099832363	0.1036	0.198721422	0.19886827	1	0.0137	0.0139	0.3252	1.013794275	0.986547307	1.041793763
chr15:61927138	rs75341202	VPS13C_L2157F	G	A	not_provided	Not_PD_known	Benign	0.01298976	0.0118	0.018695378	0.013247781	1	0.047	0.0375	0.2103	1.048122009	0.973848044	1.128060741
chr15:89325639	rs113994096	POLG_P587L	G	A	Abnormality_of_the_nerv	Not_PD_known	Conflicting_interpretations_of_p athogenicity	0.002495169	0.0023	0.003304681	0.002525879	0.999999997	0.0307	0.0849	0.7176	1.031176105	0.873101032	1.217870693

chr1:16986091	rs189334432	ATP13A2_P1124L	G	A	Kufor-Rakeb_syndrome|	Not_PD_known	Conflicting_interpretations_of_pathogenicity	0.004868161	0.0032	0.010916559	0.013383284	1	0.111	0.0666	0.09528	1.117394907	0.980653591	1.273203289
chr15:89321792	rs113994098	POLG_G848S	C	T	POLG-_Related_Disorde	Not_PD_known	Pathogenic	0.000342583	6.00E-04	0.000529617	0.000225159	0.823341966	-0.1062	0.3261	0.7446	1.112044263	0.586867622	2.107191462
chr3:132507256	rs79953286	DNAJC13_Y1673C	A	G	not_specified|not_provid	Not_PD_known	Benign	0.062294006	0.0645	0.116654042	0.109061273	1	0.0085	0.0173	0.625	1.008536228	0.974912067	1.043320067
chr15:61945803	rs114089496	VPS13C_A1687V	G	A	not_provided	Not_PD_known	Benign	0.002894355	0.0021	0.005401901	0.006328958	1	0.009	0.0807	0.9108	1.009040622	0.861420938	1.181957545
chr15:61954471	rs140338178	VPS13C_I1417L	T	G	not_provided	Not_PD_known	Likely_benign	0.000984163	8.00E-04	0.001944474	0.001847063	0.998319461	-0.0769	0.1472	0.6016	1.079934078	0.809278637	1.441107622
chr3:184321987	rs200221361	EIF4G1_A468E	C	A	.	Not_PD_known	.	0.001379765	NA	NA	NA	0.999936209	0.3329	0.2184	0.1275	1.395007791	0.90922381	2.140338513
chr15:61963904	rs200815172	VPS13C_A1088T	C	T	.	Not_PD_known	.	0.000393927	6.50E-05	0.000864304	0.000985149	0.872200377	-0.0909	0.2101	0.6654	1.095159484	0.725498699	1.65317222
chr15:61920268	rs143926369	VPS13C_V2426I	C	T	not_provided	Not_PD_known	Likely_benign	0.002035975	0.002	0.004321054	0.004900754	0.999999802	0.1301	0.0994	0.1908	1.138942272	0.93732613	1.383925464
chr15:62023783	rs150832196	VPS13C_K171E	T	C	not_provided	Not_PD_known	Likely_benign	0.000646956	8.00E-04	0.00108038	0.001748553	0.977671252	-0.1163	0.1745	0.505	1.123332821	0.797941494	1.581414975
chr15:89333267	rs752892262	POLG_P163L	G	A	Progressive_sclerosing_	Not_PD_known	Uncertain_significance	0.000528077	NA	0.000708307	0.001018633	0.947927292	0.2421	0.3077	0.4315	1.273921579	0.696984574	2.328424829
chr3:132511169	rs142160751	DNAJC13_E1740Q	G	C	.	Not_PD_known	.	0.001492668	0.0016	0.004539068	0.003670041	0.999975765	-0.15	0.1065	0.1591	1.161834243	0.942951905	1.431524557
chr15:89324193	rs2307450	POLG_E662K	C	T	Seizures|Progressive_sc	Not_PD_known	Benign	0.000371218	3.00E-04	0.000432105	0.000369394	0.852306394	0.0815	0.3702	0.8258	1.084913218	0.525139152	2.241380566
chr15:61969413	rs146460562	VPS13C_T933A	T	C	not_provided	Not_PD_known	Benign/Likely_benign	0.005021414	0.009	0.007254223	0.008865511	1	-0.0851	0.0641	0.1844	1.088825944	0.960274594	1.234586381
chr15:61961592	rs2303405	VPS13C_Y1302C	T	C	not_provided	Not_PD_known	Benign	0.078807765	0.0829	0.098759633	0.079383947	1	0.0142	0.0153	0.3517	1.014301299	0.984335977	1.04517883
chr3:132538225	rs138367039	DNAJC13_M2225I	G	A	not_provided	Not_PD_known	Benign	0.003394106	0.0036	0.006591744	0.007931034	1	0.1337	0.0769	0.082	1.143049853	0.983120089	1.328996306
chr15:89333364	rs562847013	POLG_Y131H	A	G	Intellectual_disability|Pri	Not_PD_known	Conflicting_interpretations_of_pathogenicity	0.000344634	4.00E-04	0.001296316	0.000566405	0.825570826	0.215	0.2382	0.3667	1.239861897	0.777344232	1.977576292
chr3:132523636	rs10935014	DNAJC13_V1995L	G	C	not_provided	Not_PD_known	Likely_benign	0.001135396	8.00E-04	0.001404343	0.002142541	0.999506735	-0.0828	0.1259	0.5107	1.086324522	0.848772577	1.390361799
chr15:61920099	rs115481870	VPS13C_R2482H	C	T	not_provided	Not_PD_known	Benign	0.007353783	0.006	0.012423031	0.012688479	1	0.0429	0.0496	0.3867	1.043833506	0.947132765	1.150407238
chr2:232839964	rs146430802	GIGYF2_R961Q	G	A	.	Not_PD_known	.	0.000607839	0.001	0.001728422	0.001625416	0.970368012	0.0282	0.2089	0.8926	1.028601384	0.683011215	1.549053346
chr1:16986097	rs15786	ATP13A2_P1122L	G	A	Kufor-Rakeb_syndrome|	Not_PD_known	Benign	0.058122213	0.0628	0.078073751	0.058741413	1	0.0096	0.0177	0.5877	1.009646228	0.975220188	1.045287534
chr14:22876816	rs74357167	LRP10_D518N	G	A	.	Not_PD_known	.	0.000790127	8.00E-04	0.000216053	0.00019701	0.992336113	0.1993	0.1562	0.202	1.220548075	0.898658665	1.657734647
chr6:161350187	rs149953814	PRKN_P437L	G	A	Parkinson_disease_2|nc	PD_known	Conflicting_interpretations_of_pathogenicity	0.002126524	0.0024	0.003704664	0.002432878	0.999999913	0.054	0.0763	0.4797	1.055484602	0.908874735	1.225743992
chr15:61856408	rs138846118	VPS13C_R3652X	G	A	Frontotemporal_dementi	Not_PD_known	Uncertain_significance	0.00037858	2.00E-04	0.001566778	0.000593522	0.859038903	-0.1508	0.222	0.497	1.162764082	0.752525798	1.796643137
chr3:132492497	rs201263331	DNAJC13_Y1236C	A	G	.	Not_PD_known	.	0.000475617	3.00E-04	0.000578332	0.000400257	0.925413284	0.0194	0.2525	0.9387	1.019589403	0.621574192	1.672467365
chr22:38126374	rs139184008	PLA2G6_R475Q	C	T	Infantile_neuroaxonal_dy	Not_PD_known	Uncertain_significance	0.000506163	4.00E-04	0.000756185	0.000640394	0.939422467	0.1753	0.2221	0.4301	1.191603644	0.771039249	1.841565454
chr20:5109429	rs141394228	TMEM230_M2del	A	G	.	Not_PD_known	.	0.001283467	0.0014	0.001944474	0.003275378	0.999855914	-0.1395	0.1603	0.3841	1.149698806	0.839718972	1.5741068
chr3:184319745	rs13319149	EIF4G1_T161A	A	G	not_specified	Not_PD_known	Benign	0.002502148	0.0031	0.008752026	0.008401084	0.999999997	-0.0395	0.0905	0.6626	1.040290499	0.87120323	1.24219503

Supplementary Table 4: 23andMe summary statistics							
Location	REF	ALT	FREQ	effect	stderr	pvalue	N_INFORMATIVE
chr1:155235057	C	T	6.81E-07	0	0	NA	3090507
chr1:155235195	C	T	7.03E-06	1.27652395	1.05224016	0.3141778	3090507
chr1:155235196	G	A	0.0001993	1.26221164	0.19902373	8.50E-08	3090507
chr1:155235231	T	C	6.58E-07	-1.0092804	17.434075	0.9104737	3090507
chr1:155235699	T	C	3.57E-06	0	0	NA	3090507
chr1:155235704	C	T	1.79E-06	0	0	NA	3090507
chr1:155235708	G	C	4.04E-05	0.32092984	0.49106919	0.5241418	3090507
chr1:155235725	G	C	0	0	0	NA	3090507
chr1:155235726	T	A	0	NA	NA	NA	3090507
chr1:155235750	G	A	9.86E-07	0	0	NA	3090507
chr1:155235765	T	G	9.89E-07	-1.0029091	31.0018209	0.9450107	3090507
chr1:155235777	T	A	1.95E-05	2.06049631	1.04920251	0.1319955	3090507
chr1:155235798	A	G	6.89E-06	-2.0177101	11.1887387	0.7473445	3090507
chr1:155235813	T	G	3.26E-06	-1.0010718	25.9130284	0.9429189	3090507
chr1:155235819	C	T	1.61E-06	-3.1188666	16.9107502	0.7022372	3090507
chr1:155235823	C	T	6.70E-05	-4.0418233	5.20148435	0.0433711	3090507
chr1:155235829	C	A	2.89E-06	-1.0115956	6.97838876	0.7885756	3090507
chr1:155235843	T	C	0.0031114	0.81029846	0.05240872	3.09E-44	3090507
chr1:155236269	C	T	1.97E-06	-2.4884927	18.8953748	0.716966	3090507
chr1:155236277	G	A	1.25E-06	-1.0175769	9.40336836	0.8415062	3090507
chr1:155236331	C	T	1.61E-06	-1.0012449	47.3355746	0.9687288	3090507
chr1:155236367	G	A	1.21E-06	-1.0117188	12.4412763	0.880427	3090507
chr1:155236376	C	T	0.0118229	0.36868728	0.03852363	1.02E-19	3090507
chr1:155236384	G	A	1.27E-05	2.10852387	0.62584382	0.0087029	3090507
chr1:155236399	G	T	5.63E-06	-2.0147294	11.393819	0.7520736	3090507
chr1:155236409	C	G	1.25E-06	-1.0047516	16.7741991	0.9116611	3090507
chr1:155236420	T	C	6.56E-07	0	0	NA	3090507
chr1:155236426	G	A	2.95E-06	-2.0350752	13.0021335	0.7777152	3090507

chr1:155236441	T	C	1.97E-06	-1.0014619	43.6282446	0.9565058	3090507		
chr1:155236444	T	A	1.61E-06	-1.0083751	13.273946	0.8881775	3090507		
chr1:155237357	G	A	6.57E-07	0	0	NA	3090507		
chr1:155237369	C	T	4.02E-07	0	0	NA	3090507		
chr1:155237370	G	A	5.27E-06	-2.0164675	12.9649328	0.7811275	3090507		
chr1:155237444	A	G	2.08E-07	-0.5033424	10.2471251	0.9275339	3090507		
chr1:155237576	A	T	5.58E-06	1.28026075	1.08581897	0.3162474	3090507		
chr1:155238144	A	G	4.75E-07	-1.0790105	6.33294952	0.7554763	3090507		
chr1:155238173	C	T	4.02E-07	0	0	NA	3090507		
chr1:155238186	T	C	0	NA	NA	NA	3090507		
chr1:155238194	C	T	1.43E-05	0	0	NA	3090507		
chr1:155238209	G	T	1.33E-05	-2.0121027	11.7979672	0.760587	3090507		
chr1:155238214	A	C	7.23E-06	-4.0349751	23.9945082	0.6608435	3090507		
chr1:155238215	T	C	3.27E-05	1.26404127	0.52197356	0.0417197	3090507		
chr1:155238233	G	A	1.79E-06	0	0	NA	3090507		
chr1:155238258	G	A	2.43E-06	-2.0300567	14.4002345	0.8012394	3090507		
chr1:155238264	C	T	5.33E-05	-6.3412753	4.86836429	0.0773457	3090507		
chr1:155238596	C	A	5.93E-06	-5.8218065	17.5244096	0.195627	3090507		
chr1:155238629	C	T	7.80E-06	-2.0194845	6.06278502	0.551843	3090507		
chr1:155239633	G	A	4.03E-07	-2.0365678	17.1270083	0.8318701	3090507		
chr1:155239639	A	C	4.96E-07	-0.8719552	17.1897751	0.9234288	3090507		
chr1:155239716	C	G	5.13E-06	2.32580285	1.04726406	0.0989682	3090507		
chr1:155239933	C	T	1.21E-06	-1.00182	29.1298236	0.9491824	3090507		
chr1:155239939	C	T	3.57E-06	0	0	NA	3090507		
chr1:155239948	G	A	6.63E-06	-2.0218211	9.65055865	0.7066723	3090507		
chr1:155240048	C	T	1.08E-05	-2.0143477	11.8600105	0.7615592	3090507		
chr1:155240078	C	T	4.02E-07	0	0	NA	3090507		
chr1:155241085	C	T	7.28E-07	-1.0016887	40.6027429	0.9635335	3090507		
chr1:16986208	C	T	0	-306.32403	991.809097	0.0952772	3090507		

chr1:16986291	C	T	7.39E-05	-1.8150027	1.99724295	0.270379	3090507	
chr1:16987157	C	T	0.000175	0.41820974	0.36281125	0.2844408	3090507	
chr1:16987187	G	A	5.72E-05	-3.1860909	2.81701154	0.1521852	3090507	
chr1:16988161	T	A	0.0018218	-0.1992043	0.15095628	0.1753031	3090507	
chr1:16988226	C	T	8.29E-05	-0.1199536	1.72605705	0.9408016	3090507	
chr1:16990191	C	T	3.08E-05	-0.8333348	2.27954461	0.6796899	3090507	
chr1:16992115	C	T	7.15E-07	-0.7870695	16.4239908	0.9415958	3090507	
chr1:16993627	A	C	0.0141967	0.14026735	0.15472269	0.372417	3090507	
chr1:16997107	G	A	5.52E-05	1.21168189	0.42584815	0.0160465	3090507	
chr1:17000429	T	C	1.78E-06	-5.9512829	26.5081176	0.6260961	3090507	
chr1:17000495	C	T	5.79E-05	0.57100972	1.45412123	0.7151673	3090507	
chr1:17011732	C	G	6.20E-06	-258.36311	476.856379	0.1288659	3090507	
chr1:20633840	C	T	1.05E-05	-2.0088982	13.0926225	0.7839576	3090507	
chr1:20637888	C	T	2.79E-05	-4.1496595	11.2521272	0.3324721	3090507	
chr1:20637894	G	A	3.05E-05	-5.0851494	17.993291	0.3257971	3090507	
chr1:20638104	C	A	3.28E-07	-1.0082875	18.4692972	0.9150608	3090507	
chr1:20639911	C	T	1.04E-07	-1.000712	62.4851037	0.9763182	3090507	
chr1:20639934	G	A	8.05E-07	-1.0011251	49.778135	0.9702654	3090507	
chr1:20639990	C	A	2.48E-07	0	0	NA	3090507	
chr1:20644526	C	A	1.17E-06	-1.0002808	72.2717091	0.9795313	3090507	
chr1:20644551	G	A	7.24E-06	-1.004416	10.8290332	0.8635916	3090507	
chr1:20644626	C	G	6.25E-07	-1.9449021	19.7908531	0.8185974	3090507	
chr1:20644627	C	T	8.20E-06	-3.0583659	16.3995835	0.6983977	3090507	
chr1:20644639	G	A	2.02E-05	-4.0203638	15.7454635	0.5077484	3090507	
chr1:20644651	C	T	0.0001749	0.13809897	0.36316473	0.7098136	3090507	
chr1:20645640	T	C	3.15E-07	0	0	NA	3090507	
chr1:20648534	T	C	2.81E-06	-2.0165706	14.2089792	0.7998563	3090507	
chr1:20648601	G	A	2.25E-05	-3.0137415	13.0793627	0.6348833	3090507	
chr1:20649062	G	A	2.37E-07	-1.0074434	19.1510221	0.9224192	3090507	

chr1:20649095	A	G	2.01E-05	-4.0486237	18.0847693	0.5602783	3090507	
chr1:20649224	C	T	0.000172	-1.9701811	2.29118765	0.3135582	3090507	
chr12:40225159	G	A	2.01E-06	-1.0073775	17.8846573	0.9169499	3090507	
chr12:40225558	C	T	6.56E-07	-1.0005301	72.3471869	0.9659541	3090507	
chr12:40232383	A	G	1.32E-05	-7.0022297	14.9510888	0.2578182	3090507	
chr12:40232385	T	C	8.04E-07	-7.0022296	14.9510885	0.2578182	3090507	
chr12:40240535	G	T	4.17E-07	-1.0007387	61.4355765	0.9759133	3090507	
chr12:40243556	A	T	2.96E-06	2.24439246	0.92741538	0.0226586	3090507	
chr12:40251361	A	G	1.61E-06	-1.0012264	47.6552653	0.9689389	3090507	
chr12:40251480	G	A	1.18E-06	-7.5454058	60.9370547	0.8154518	3090507	
chr12:40257264	A	G	4.95E-06	-3.0209301	16.0095211	0.6957765	3090507	
chr12:40257283	C	T	1.19E-06	-2.5301035	89.3332816	0.9231721	3090507	
chr12:40257348	T	G	1.64E-06	-1.0178615	12.6706064	0.8789441	3090507	
chr12:40259493	G	A	0	0	0	NA	3090507	
chr12:40263861	A	G	4.88E-05	-5.1254216	11.6121148	0.1232078	3090507	
chr12:40263875	A	G	1.25E-05	-4.0637025	12.5740289	0.3990873	3090507	
chr12:40274671	T	C	6.57E-07	-1.0183614	12.1835814	0.8743327	3090507	
chr12:40278154	A	G	9.65E-06	-2.0102769	14.5686539	0.8052566	3090507	
chr12:40284061	A	G	2.63E-06	-1.0142824	10.9580843	0.8614632	3090507	
chr12:40293626	G	A	0.0001931	0.70620539	0.51947558	0.2105061	3090507	
chr12:40293629	G	A	6.82E-06	-0.1349321	3.07488018	0.9632431	3090507	
chr12:40293644	A	G	1.61E-06	-1.0092364	17.4969799	0.9149894	3090507	
chr12:40294909	C	T	2.38E-07	-1.0402551	22.3005467	0.9298332	3090507	
chr12:40299255	T	C	0.0001146	-5.0383703	13.4721533	0.1965542	3090507	
chr12:40302860	G	A	2.33E-06	-1.5103936	10.5293475	0.7855547	3090507	
chr12:40304000	G	A	0.0001286	0.3490258	0.49183682	0.4990678	3090507	
chr12:40304004	A	G	2.30E-06	-1.011592	11.7553819	0.8706714	3090507	
chr12:40304079	C	T	1.79E-05	0	0	NA	3090507	
chr12:40305893	G	T	6.58E-07	-1.001365	45.1426497	0.957389	3090507	

chr12:40309225	A	C	2.08E-07	-1.0028124	31.4954012	0.9529565	3090507		
chr12:40310437	G	C	0	0	0	NA	3090507		
chr12:40310515	A	G	6.03E-06	-3.0429999	13.4438186	0.640363	3090507		
chr12:40310561	G	A	1.53E-05	-3.0278936	12.3968631	0.6144545	3090507		
chr12:40314101	C	A	0	0	0	NA	3090507		
chr12:40315266	T	A	4.03E-07	-1.0003876	84.7887603	0.9825512	3090507		
chr12:40319998	T	C	8.04E-07	-1.0056969	17.1926873	0.9137375	3090507		
chr12:40320042	C	T	4.43E-05	-13.897279	11.2849439	0.0271423	3090507		
chr12:40321114	A	G	8.71E-07	-1.0153004	10.8227369	0.8623185	3090507		
chr12:40322037	C	T	3.32E-05	-4.0454497	10.7821093	0.3286805	3090507		
chr12:40322038	G	A	1.64E-06	-1.0051998	23.2088524	0.9305352	3090507		
chr12:40323255	A	G	1.21E-06	-1.0044385	17.85015	0.9169873	3090507		
chr12:40323270	G	T	4.03E-06	-1.0042976	13.3421338	0.8891102	3090507		
chr12:40340380	T	C	3.96E-07	-1.0061425	13.6800181	0.8916706	3090507		
chr12:40340400	G	A	0.0006262	2.48010897	0.06001126	3.78E-250	3090507		
chr12:40340404	T	C	2.44E-06	3.06388647	0.85977255	0.0069103	3090507		
chr12:40340436	A	T	1.21E-06	0	0	NA	3090507		
chr12:40348484	C	T	1.79E-06	0	0	NA	3090507		
chr12:40351579	C	T	1.05E-05	-3.0164174	15.5501568	0.6892884	3090507		
chr12:40351680	G	C	0.000797	-0.5511453	0.5085493	0.2342084	3090507		
chr12:40363410	A	G	0.000384	-0.5516944	0.71617906	0.3962727	3090507		
chr12:40363541	G	A	1.02E-05	-3.0165002	16.1607763	0.6987584	3090507		
chr12:40364843	G	A	5.91E-06	-2.0320316	12.5390081	0.7701991	3090507		
chr12:40364975	C	A	2.92E-05	-4.0587211	12.1553009	0.3839149	3090507		
chr12:40367012	T	A	1.81E-05	-2.0064613	13.9921788	0.7977608	3090507		
chr12:40367050	A	G	2.30E-06	-1.004516	24.90282	0.9345348	3090507		
chr14:22873373	C	T	0.0001822	0	0	NA	3090507		
chr14:22875400	G	A	0.0002552	-2.4554556	0.92473034	0.0006128	3090507		
chr14:22875648	G	A	0.0001122	-0.131902	0.70592586	0.8427653	3090507		

chr14:22875772	C	A	1.40E-06	-1.3026987	14.284279	0.880215	3090507		
chr14:22876816	G	A	0.0007901	-0.1823382	0.15999139	0.241448	3090507		
chr14:22877045	C	T	0.0001741	-6.0410797	14.5278637	0.0476669	3090507		
chr14:22877128	G	T	6.06E-05	-0.4884047	2.52934111	0.8378083	3090507		
chr15:61875847	T	A	4.04E-07	NA	NA	NA	3090507		
chr15:61880845	G	A	2.42E-05	-0.0533438	1.79067141	0.9756583	3090507		
chr15:61907289	T	C	8.53E-06	-31.534202	41.3793988	0.194865	3090507		
chr15:61910215	G	A	6.05E-06	-2.0202475	11.0426547	0.7438251	3090507		
chr15:61915865	G	C	5.12E-07	-51.302462	346.20307	0.7708907	3090507		
chr15:61917535	C	T	5.84E-06	-14.12043	47.471856	0.5422235	3090507		
chr15:61919368	G	C	6.73E-07	-2.7571072	39.7987206	0.8634662	3090507		
chr15:61922396	C	T	1.21E-06	-30232177	0	NA	3090507		
chr15:61929659	G	C	1.44E-05	2.89638097	1.46609066	0.1172494	3090507		
chr15:61940726	G	A	6.31E-06	-0.4064204	5.62820114	0.9318704	3090507		
chr15:61961754	G	A	0.0001704	-0.3567998	0.56413781	0.5071897	3090507		
chr15:61962390	T	G	6.59E-05	-6.4009422	4.05062058	0.018637	3090507		
chr15:61962754	T	A	4.46E-06	-1.006483	12.1543763	0.87814	3090507		
chr15:61969298	C	A	4.03E-07	0	0	NA	3090507		
chr15:61977090	T	G	4.44E-06	-232.43949	924.928072	0.452051	3090507		
chr15:61978646	T	C	0.0007585	-0.180658	0.14696564	0.2068122	3090507		
chr15:61983818	A	G	1.61E-06	-1.0087373	15.7546568	0.9056673	3090507		
chr15:61983868	G	T	3.93E-06	-8.8134291	20.1500916	0.4449589	3090507		
chr15:61983969	G	A	2.01E-05	-3.0101859	14.8267667	0.6757071	3090507		
chr15:61991076	G	C	1.42E-06	-2.3240306	15.8724086	0.7348867	3090507		
chr15:62008659	G	A	7.23E-06	-2.0392983	11.7126773	0.7559255	3090507		
chr15:62012166	T	C	3.67E-05	-4.0225138	13.6886645	0.4457938	3090507		
chr15:62013943	A	G	4.01E-05	0.55552936	1.67507157	0.7576397	3090507		
chr15:62023788	C	T	2.41E-05	-10.415469	9.63708476	0.0530769	3090507		
chr15:62023837	G	A	7.06E-05	0.23832171	0.79492077	0.7703012	3090507		

chr15:62035002	C	A	2.20E-06	-4.4842548	21.2982158	0.6149649	3090507		
chr15:62044256	C	T	7.17E-05	0.50578447	1.02280979	0.6534452	3090507		
chr15:89317460	G	A	7.05E-05	-0.0380496	0.91744095	0.9662545	3090507		
chr15:89318617	C	T	2.29E-06	-1.007346	11.1403157	0.8670537	3090507		
chr15:89318962	C	T	1.41E-05	-3.0286507	8.22859361	0.4477919	3090507		
chr15:89318989	G	C	3.54E-06	-1.0051828	10.751481	0.8625324	3090507		
chr15:89319318	A	C	0.0005991	-0.2456412	0.29968425	0.3971739	3090507		
chr15:89320878	C	A	3.28E-07	0	0	NA	3090507		
chr15:89320890	G	A	6.56E-07	-1.0019717	37.5844238	0.9521203	3090507		
chr15:89320907	T	C	1.56E-06	-1.0017417	24.0870516	0.9385664	3090507		
chr15:89320953	G	A	6.28E-06	-4.3907834	15.2236158	0.4347742	3090507		
chr15:89320998	C	T	4.16E-07	-1.0034153	24.4712436	0.9394467	3090507		
chr15:89321242	C	A	9.94E-07	-1.0080088	14.2965439	0.8926886	3090507		
chr15:89321773	G	T	4.16E-07	-1.0034443	19.5642112	0.9242982	3090507		
chr15:89321780	G	A	5.21E-07	-1.0070206	20.0067718	0.9257532	3090507		
chr15:89322748	C	T	2.18E-05	-3.0313221	6.22207777	0.3149389	3090507		
chr15:89323403	C	G	1.04E-07	0	0	NA	3090507		
chr15:89325456	G	C	1.90E-05	0.63082809	1.01682886	0.587954	3090507		
chr15:89325466	C	A	2.32E-05	-0.3311402	2.51872461	0.8825679	3090507		
chr15:89325520	G	A	3.95E-05	0	0	NA	3090507		
chr15:89325683	C	A	0	NA	NA	NA	3090507		
chr15:89327006	C	G	7.77E-05	-0.3882372	1.01671676	0.6823921	3090507		
chr15:89330250	A	C	0.0007145	-0.0306425	0.29413796	0.9158075	3090507		
chr15:89333177	C	T	4.05E-06	2.0479639	2.95810914	0.6671316	3090507		
chr15:89333267	G	A	0.0005281	-0.2420522	0.30768285	0.412597	3090507		
chr15:89333347	G	C	6.88E-06	-11.761845	42.8445222	0.7104004	3090507		
chr15:89333427	G	A	5.32E-05	0.94483133	0.75661634	0.2729649	3090507		
chr16:46662405	A	C	2.38E-06	-25.259479	84.0319514	0.4332015	3090507		
chr16:46671766	T	C	1.04E-07	-1.0048445	24.0369099	0.9382831	3090507		

chr16:46682118	G	A	2.08E-07	-1.0098525	14.8247047	0.89969	3090507	
chr1:65385756	G	A	6.71E-05	-0.6428898	2.9480431	0.8161035	3090507	
chr1:65386820	G	A	0.0001697	0.57962702	1.02467972	0.6000578	3090507	
chr1:65392613	C	A	4.60E-06	-7.8558621	62.8723229	0.6715672	3090507	
chr1:65401812	C	T	9.30E-06	-11.402306	12.7293173	0.1696898	3090507	
chr17:46010324	T	G	2.08E-07	NA	NA	NA	3090507	
chr17:46024061	C	T	1.23E-05	1.55517561	0.75230447	0.0943505	3090507	
chr1:7962863	G	A	4.02E-07	NA	NA	NA	3090507	
chr1:7965348	G	T	0	0	0	NA	3090507	
chr1:7965425	G	C	8.06E-07	-1.0045643	24.78631	0.9401593	3090507	
chr1:7984930	A	C	1.16E-06	-102891562	0	NA	3090507	
chr1:7984981	T	C	6.62E-07	-1.0040375	24.2849129	0.9389522	3090507	
chr1:7985019	G	A	0.0006413	0.31832982	0.2087645	0.1449402	3090507	
chr20:5069205	C	T	1.65E-05	-315.90659	1079.11591	0.2617296	3090507	
chr20:5069330	C	T	0.0001057	-4.3065497	4.79207794	0.2436509	3090507	
chr20:5100832	G	A	0.0020627	-0.1636226	0.15946767	0.294959	3090507	
chr20:5109429	A	G	0.0012835	0.00427511	0.21753604	0.9843513	3090507	
chr20:5109435	C	T	0.0001082	-0.8642509	1.32153156	0.461526	3090507	
chr20:5111588	G	A	0.0001099	-1.2503238	1.17123065	0.2042105	3090507	
chr21:32641895	C	T	4.02E-07	-1.0117138	15.5560187	0.9042397	3090507	
chr21:32643457	C	T	1.43E-06	-289.69881	1156.64626	0.4438919	3090507	
chr21:32646598	A	T	0	0	0	NA	3090507	
chr21:32657057	G	A	5.39E-06	-33.960519	126.908077	0.5355701	3090507	
chr21:32666479	C	T	3.00E-05	3.12401063	1.10952403	0.0371834	3090507	
chr21:32678644	C	A	1.21E-06	-1.0053456	17.06117	0.9130995	3090507	
chr21:32688306	C	T	3.04E-06	-3.4156023	22.8545834	0.6781983	3090507	
chr21:32688348	C	T	4.29E-06	-2.8112322	8.73174587	0.6539023	3090507	
chr21:32726855	T	C	1.22E-05	-0.3547748	3.21385202	0.8956887	3090507	
chr22:32475067	C	T	9.42E-06	-4.1165266	14.4507615	0.4546226	3090507	

chr22:32483895	A	T	4.82E-06	6.40E+54	0	NA	3090507	
chr22:32493282	G	T	6.56E-07	0	0	NA	3090507	
chr22:32498466	A	G	8.58E-06	-5.5378194	30.8345352	0.6805339	3090507	
chr2:232790817	A	G	3.58E-06	0	0	NA	3090507	
chr2:232832882	G	A	0.0001345	0.6799521	0.41807405	0.1351799	3090507	
chr2:232839964	G	A	0.0006078	-0.1094614	0.27999287	0.6917026	3090507	
chr2:232844498	G	C	8.64E-07	-1.3152949	17.4126311	0.8750183	3090507	
chr2:232844499	G	C	8.64E-07	-1.3152949	17.4126311	0.8750183	3090507	
chr2:232847564	A	C	0.0028226	-0.1740289	0.13060382	0.1745985	3090507	
chr22:38112571	C	A	7.53E-05	0.11268056	0.79561589	0.8933919	3090507	
chr22:38115667	G	A	5.40E-06	0	0	NA	3090507	
chr22:38116119	C	A	0.0001619	1.00794054	0.41571505	0.0309984	3090507	
chr22:38116200	G	A	8.33E-07	-1.0005543	41.5474744	0.9643951	3090507	
chr22:38128349	G	A	9.67E-05	0.18074118	0.59692687	0.7669191	3090507	
chr22:38132917	C	T	9.54E-05	0.05507724	0.85080203	0.9494457	3090507	
chr22:38132979	A	T	3.97E-07	-1.0059992	18.4436833	0.9195477	3090507	
chr22:38133010	C	T	2.16E-05	-4.8624206	13.4507367	0.5781323	3090507	
chr22:38135061	A	C	1.04E-07	-1.0005278	72.5718905	0.9796121	3090507	
chr22:38140106	G	A	2.97E-05	-0.3599733	1.0238173	0.7084799	3090507	
chr22:38143236	A	G	2.08E-07	0	0	NA	3090507	
chr22:38145438	C	A	5.36E-06	0	0	NA	3090507	
chr22:38145597	G	T	1.50E-05	0.86193525	2.30927431	0.7288847	3090507	
chr22:38145625	C	T	8.94E-06	0	0	NA	3090507	
chr22:38169216	A	C	1.61E-06	0	0	NA	3090507	
chr2:74530208	C	T	1.10E-05	-0.411176	2.66172233	0.8525646	3090507	
chr2:74530433	C	G	4.16E-07	-1.0004654	57.1180005	0.9740989	3090507	
chr2:74532831	G	A	3.48E-05	-4.091354	3.75498503	0.1151441	3090507	
chr3:132450732	A	T	0.000145	0.08092138	0.59230845	0.895206	3090507	
chr3:132454135	C	T	3.23E-05	0.43369988	1.01732413	0.6941258	3090507	

chr3:132457295	T	C	1.15E-06	-1.2164478	18.3256349	0.8949049	3090507	
chr3:132503289	G	A	7.00E-06	0.47573228	3.21902274	0.9014056	3090507	
chr3:132505400	A	T	3.46E-06	-0.6923998	5.21980659	0.8437288	3090507	
chr3:184320964	C	T	4.74E-05	0.15184682	1.41801285	0.9199456	3090507	
chr3:184321315	G	A	1.27E-06	-1.1029521	21.0623855	0.9182847	3090507	
chr3:184321497	C	T	2.32E-06	-2.7574674	15.302928	0.641326	3090507	
chr3:184321987	C	A	0.0013798	-0.3328819	0.21843473	0.1129649	3090507	
chr3:184327694	A	G	4.59E-06	-35.323116	52.8749281	0.2821608	3090507	
chr3:184328662	A	G	9.76E-05	-5.9938561	3.03169199	0.0045018	3090507	
chr4:41263258	T	C	9.29E-07	1.92211667	6.37225837	0.8585832	3090507	
chr4:89828170	C	T	6.56E-07	0	0	NA	3090507	
chr5:176626472	C	T	0	0	0	NA	3090507	
chr6:161350139	C	T	8.50E-06	-2.0106489	11.0557334	0.7452217	3090507	
chr6:161360168	C	T	3.00E-05	-4.0390804	13.7845405	0.446325	3090507	
chr6:161360187	T	C	8.04E-07	-1.0158596	13.4151533	0.8886814	3090507	
chr6:161569357	G	A	0	2	374563.324	0.9999763	3090507	
chr6:161785778	A	C	3.31E-07	-1.0049398	21.7842109	0.9319105	3090507	
chr6:161785793	C	G	1.61E-06	-1.0137864	14.3636697	0.8961614	3090507	
chr6:161785825	T	C	4.08E-05	-4.0373528	12.8387238	0.4138217	3090507	
chr6:161785839	A	T	6.05E-06	-1.0069666	10.2001079	0.854974	3090507	
chr6:161785844	A	G	8.04E-06	-1.0034884	14.7395759	0.8996306	3090507	
chr6:161973347	G	A	1.28E-06	-1.3948107	10.766703	0.7818079	3090507	
chr6:162054109	G	C	6.57E-07	-1.0134906	14.5221822	0.8938041	3090507	
chr6:162201237	T	C	1.21E-06	0	0	NA	3090507	
chr6:162262627	G	A	1.53E-05	-3.0623659	11.9293776	0.5958641	3090507	
chr6:162262681	C	T	1.13E-05	-3.016814	14.6713332	0.6716851	3090507	
chr6:162443357	G	A	6.83E-06	-2.0097311	12.9839176	0.7821093	3090507	
chr6:162443429	C	T	4.02E-06	-12.480147	17.6616053	0.2935117	3090507	
chr6:162443438	C	T	1.21E-06	-0.9553581	15.213452	0.9065788	3090507	

chr1:155235006	G	A	5.83E-06	-2.4268208	6.46042411	0.4990213	3090507		
chr1:155235727	C	G	0.0001032	0.23006021	0.41964251	0.5953241	3090507		
chr1:155235772	C	A	2.62E-05	1.39262769	0.38511319	0.0022254	3090507		
chr1:155235780	G	A	1.64E-06	5.26746069	3.17892024	0.0311191	3090507		
chr1:155236246	G	A	0.0068502	0.40030395	0.0464187	3.18E-16	3090507		
chr1:155236276	C	T	4.92E-06	2.60543778	1.25362038	0.0890702	3090507		
chr1:155237423	G	A	0.0001574	0.00462539	0.4192363	0.9912203	3090507		
chr1:155237438	C	T	0.0001537	0.97765539	0.2734576	0.0015685	3090507		
chr1:155237453	C	T	1.81E-05	1.36422256	0.3066342	0.0001893	3090507		
chr1:155237458	A	C	8.28E-08	-0.1392236	0.61904417	0.8083862	3090507		
chr1:155238570	C	G	0.0001241	1.21310434	0.22523138	4.27E-06	3090507		
chr1:16985990	C	T	0.0004965	0.09892469	0.16099459	0.5446935	3090507		
chr1:16986065	C	T	0.0001851	0.23094144	0.43384725	0.6039422	3090507		
chr1:16986091	G	A	0.0048682	-0.0603329	0.08175851	0.4568988	3090507		
chr1:16986097	G	A	0.0581222	-0.004987	0.01997487	0.8023197	3090507		
chr1:16986101	T	A	0.0056118	-0.0180521	0.0666682	0.7843562	3090507		
chr1:16986248	C	T	0.476404	-0.0351455	0.0092878	0.0001536	3090507		
chr1:16986334	C	T	4.75E-05	-0.1637527	1.13731328	0.8759448	3090507		
chr1:16986335	G	A	0.0004378	0.04714944	0.26922299	0.864597	3090507		
chr1:16986554	G	A	0.0006539	-0.090349	0.17917718	0.6097132	3090507		
chr1:16990276	G	C	0.0006796	0.4847637	0.218346	0.0366331	3090507		
chr1:16991787	G	A	1.31E-05	-3.0237255	7.73504926	0.4202245	3090507		
chr1:16992042	A	G	4.83E-05	0.21909334	0.82928675	0.8130946	3090507		
chr1:16996298	G	C	0.0012094	0.17879127	0.1460452	0.2317274	3090507		
chr1:16997044	C	T	7.23E-05	-0.7649385	0.99528364	0.395375	3090507		
chr1:17000107	C	T	2.06E-05	-4.0924105	9.44431208	0.2576994	3090507		
chr1:17000272	C	T	0.0170242	-0.0274742	0.03547936	0.4368272	3090507		
chr1:17000494	G	A	0.0007692	0.11340264	0.14610326	0.444903	3090507		
chr1:17005517	C	T	0.0001809	0.25363585	0.32520817	0.4540628	3090507		

chr1:175386035	T	C	0.0005861	-0.3289023	0.26814035	0.197174	3090507	
chr1:175403578	T	G	0.0057561	0.18466182	0.05882291	0.0022746	3090507	
chr1:175406219	T	C	0.0051131	0.11825393	0.064564	0.0717712	3090507	
chr1:175406252	A	T	8.85E-05	-1.0083911	1.01434448	0.2344602	3090507	
chr11:94447275	C	T	0.0002703	-0.319323	0.33634477	0.3179484	3090507	
chr1:20633615	G	A	1.08E-05	-1.0413999	2.71470215	0.6336148	3090507	
chr1:20633766	C	T	8.40E-06	-0.5789038	2.59492336	0.7521207	3090507	
chr1:20637908	C	T	2.01E-05	0.74605799	0.90442587	0.4749217	3090507	
chr1:20637956	G	C	1.41E-05	-2.0134392	5.77896703	0.5336396	3090507	
chr1:20638080	C	T	5.50E-05	0.75314271	0.27375499	0.0128505	3090507	
chr1:20644515	C	G	7.80E-05	0.12013298	0.80622345	0.8888539	3090507	
chr1:20644549	G	A	0.0001574	1.18366192	0.39406584	0.0088444	3090507	
chr1:20644564	C	A	1.44E-05	-4.2013632	8.96924705	0.3555347	3090507	
chr1:20644570	C	T	1.98E-05	-4.0678406	8.33004919	0.2030701	3090507	
chr1:20644662	G	A	9.09E-05	-0.7835105	1.08248627	0.4067069	3090507	
chr1:20644665	A	T	0.0010678	-0.014286	0.15789997	0.9273473	3090507	
chr1:20645615	G	A	0.0010258	-0.125471	0.20720962	0.5382965	3090507	
chr1:20645675	G	A	1.85E-05	-3.0334209	6.57238366	0.3410498	3090507	
chr1:20648528	G	A	0.000251	-0.5742029	0.53024748	0.2339044	3090507	
chr1:20648577	C	T	9.50E-07	-1.0096012	9.46434152	0.84351	3090507	
chr1:20648612	G	A	0.0001388	-0.4488493	0.19778488	0.0155853	3090507	
chr1:20649034	T	C	9.93E-07	-1.0356637	8.89030462	0.828005	3090507	
chr1:20649054	G	A	7.04E-06	-2.0398897	6.3052047	0.5635716	3090507	
chr1:20649109	C	T	7.17E-05	-5.0426839	7.84233052	0.0263098	3090507	
chr1:20649134	G	A	3.66E-05	-0.0517362	1.01882099	0.9581401	3090507	
chr1:20649217	C	T	7.19E-05	0.02651569	0.68276268	0.9694501	3090507	
chr12:40235634	T	C	0.0024926	-0.0258786	0.09552234	0.7830943	3090507	
chr12:40240543	C	T	0.0001559	-0.1110389	0.33075684	0.7332349	3090507	
chr12:40240594	G	C	0.0001656	-1.1705431	0.75396823	0.0582123	3090507	

chr12:40249843	C	G	0.0001436	0.67085448	0.44655032	0.1579251	3090507		
chr12:40251273	G	A	0.0010785	0.3154777	0.13500038	0.0250127	3090507		
chr12:40251346	C	T	0.0002828	-0.0240513	0.31624332	0.938582	3090507		
chr12:40251369	G	A	1.24E-05	-2.0211789	5.48372026	0.5103159	3090507		
chr12:40263806	A	G	0.0001461	-0.3598332	0.30204852	0.2063056	3090507		
chr12:40274666	A	T	3.64E-06	-2.038894	6.44701224	0.5701255	3090507		
chr12:40278179	A	G	7.73E-05	-0.1338493	0.46059016	0.754831	3090507		
chr12:40284011	G	T	0.0006853	-0.4202578	0.26916028	0.0960325	3090507		
chr12:40293552	A	C	1.97E-05	-0.3988341	2.48849844	0.8393345	3090507		
chr12:40293624	G	C	0.0001154	-0.2791448	0.47928378	0.5435308	3090507		
chr12:40294866	G	T	9.98E-06	-3.0322869	6.63457112	0.3457942	3090507		
chr12:40295466	G	A	1.01E-05	1.20266255	1.03578852	0.3342336	3090507		
chr12:40298346	G	A	4.06E-06	-1.0126735	4.98466489	0.7071062	3090507		
chr12:40298433	C	G	6.23E-05	-0.1510085	1.11435372	0.8810775	3090507		
chr12:40299125	A	G	1.90E-06	-1.0110682	7.20899842	0.7952707	3090507		
chr12:40299212	G	A	0.0001217	0.05022266	0.76214562	0.9420802	3090507		
chr12:40302866	A	G	1.41E-05	0.415008	1.34148537	0.7725634	3090507		
chr12:40304040	G	C	0.0003076	0.00146197	0.58859504	0.99802	3090507		
chr12:40305946	T	A	5.47E-05	-0.5846001	1.014089	0.5195053	3090507		
chr12:40308481	G	A	0.0002266	0.37564968	0.24246812	0.1436589	3090507		
chr12:40308507	C	T	9.26E-06	2.70309687	1.11069244	0.0696963	3090507		
chr12:40309174	G	A	7.28E-07	-1.0538723	7.28830779	0.7906483	3090507		
chr12:40310435	G	A	3.47E-06	3.8309197	0.58341092	9.36E-08	3090507		
chr12:40320103	A	G	9.13E-05	0.61542705	0.47662242	0.2299976	3090507		
chr12:40322386	G	T	8.48E-05	0.86863099	0.21171909	0.0002754	3090507		
chr12:40323256	T	C	0.0004141	0.23209761	0.23464174	0.339394	3090507		
chr12:40323300	C	T	5.92E-06	2.03195189	1.25300085	0.1653542	3090507		
chr12:40335031	G	A	9.71E-05	0.18432648	0.33458351	0.5912857	3090507		
chr12:40351585	G	A	5.14E-05	0.69691426	0.35508345	0.0737643	3090507		

chr12:40351723	A	G	0.0002185	0.09163862	0.32438121	0.7895208	3090507	
chr12:40354486	A	T	0.0001326	0.39595418	0.47284403	0.4268318	3090507	
chr12:40356126	A	T	5.28E-06	1.86333852	1.39339686	0.2180341	3090507	
chr12:40359345	C	T	9.90E-05	-0.792473	0.81787532	0.2715308	3090507	
chr12:40363440	C	T	0.0001865	-0.3353	0.5798728	0.5442308	3090507	
chr12:40363526	G	A	2.43E-05	0.33093808	0.71577109	0.6589422	3090507	
chr12:40367045	G	A	0.0001203	0.04769005	0.4966685	0.9253595	3090507	
chr14:22875363	A	G	0.0006155	0.20651325	0.19750552	0.3100825	3090507	
chr14:22875639	C	T	0.0001342	-0.4681505	0.6692226	0.4521778	3090507	
chr14:22875924	G	A	0.0011051	0.03029751	0.16467194	0.8562907	3090507	
chr14:22876078	C	G	3.46E-05	-0.0016776	1.19231332	0.9988766	3090507	
chr14:22876113	G	A	0.0002377	0.42141315	0.30542085	0.1958913	3090507	
chr14:22876722	G	C	0.0002216	0.47955579	0.31400996	0.15039	3090507	
chr14:22876765	G	T	0.0001287	0.50785173	0.39131913	0.2313051	3090507	
chr14:22877070	G	A	0.0082133	0.06049829	0.05184118	0.2473785	3090507	
chr14:22877108	G	A	0.0001271	0.66778326	0.38116695	0.109484	3090507	
chr14:22877184	G	A	2.75E-05	0.86254423	1.15686181	0.5150867	3090507	
chr14:22877421	A	C	0.0002874	0.14996603	0.27753668	0.5964625	3090507	
chr15:50586433	G	A	0.1052671	0.01230758	0.01506783	0.4147339	3090507	
chr15:61854543	T	C	0.0055119	-0.049805	0.06435242	0.4354762	3090507	
chr15:61854888	C	A	0.0012564	-0.0294774	0.15017369	0.8419047	3090507	
chr15:61856407	C	T	0.0003455	-0.6334532	0.35859154	0.0531113	3090507	
chr15:61856408	G	A	0.0003786	-0.0478629	0.29777267	0.8687299	3090507	
chr15:61868675	C	T	0.0001192	0.0481113	0.46221102	0.919534	3090507	
chr15:61868702	C	T	0.0004666	0.08227245	0.22776434	0.7210345	3090507	
chr15:61880871	G	A	0.0001554	0.04143975	0.42352613	0.9239276	3090507	
chr15:61882609	A	G	0.0002199	-0.501466	0.4610159	0.2380741	3090507	
chr15:61890400	C	T	0.0001979	-0.948257	0.47105534	0.0189926	3090507	
chr15:61910283	C	T	0.4742973	-0.0116656	0.00923092	0.20627	3090507	

chr15:61911844	G	A	0.0033392	0.03674603	0.08236581	0.6572092	3090507	
chr15:61915655	T	C	0.0006731	-0.0828184	0.19938854	0.6740745	3090507	
chr15:61915712	A	G	0.010065	0.05926755	0.04581017	0.1996623	3090507	
chr15:61915725	G	A	4.03E-05	0.30968246	0.77482675	0.7033018	3090507	
chr15:61915890	G	A	0.0001543	-0.776492	0.62985259	0.1647166	3090507	
chr15:61916007	G	C	0.0001379	0.18420205	0.46404087	0.6991941	3090507	
chr15:61918208	T	C	0.0002396	0.23250953	0.31307792	0.4721949	3090507	
chr15:61920099	C	T	0.0073538	-0.0326266	0.05503678	0.5513264	3090507	
chr15:61920268	C	T	0.002036	-0.1390464	0.11668622	0.2235643	3090507	
chr15:61920571	T	C	0.0039445	0.04946014	0.07703913	0.5238476	3090507	
chr15:61922408	C	T	0.0790141	0.01922023	0.01708228	0.2617184	3090507	
chr15:61922527	G	A	0.0004023	-0.3579735	0.32396613	0.2459423	3090507	
chr15:61922557	A	G	0.0008407	0.3101611	0.17185027	0.0838661	3090507	
chr15:61922680	G	T	9.65E-05	0.03229187	0.63161713	0.9598761	3090507	
chr15:61927138	G	A	0.0129898	-0.0601001	0.0426889	0.1553597	3090507	
chr15:61929546	T	A	1.57E-06	4.36614746	1.35213181	0.0245137	3090507	
chr15:61931104	C	G	0.0189984	0.02160914	0.03459966	0.5335307	3090507	
chr15:61934224	T	G	0.008077	-0.028422	0.05253628	0.5868861	3090507	
chr15:61936649	C	G	4.66E-05	0.39290043	0.98046794	0.702908	3090507	
chr15:61936716	A	C	1.87E-05	-3.7122229	8.49652421	0.2843124	3090507	
chr15:61940651	A	G	2.92E-05	0.09890948	2.59909733	0.971274	3090507	
chr15:61941806	T	C	3.43E-05	1.77607421	0.62500939	0.0197059	3090507	
chr15:61945803	G	A	0.0028944	0.02338435	0.09234184	0.8026798	3090507	
chr15:61950998	T	C	0.0776378	0.02153826	0.01705558	0.2079263	3090507	
chr15:61951827	T	C	3.55E-05	1.09556665	0.49347616	0.0556593	3090507	
chr15:61954471	T	G	0.0009842	-0.0648494	0.1703106	0.7008406	3090507	
chr15:61961592	T	C	0.0788078	0.01897331	0.01709679	0.2682886	3090507	
chr15:61962790	T	C	0.0253778	-0.0584961	0.03024925	0.0511	3090507	
chr15:61963904	C	T	0.0003939	0.15082067	0.24018637	0.5386828	3090507	

chr15:61966088	C	T	5.63E-05	0.26054196	0.8617952	0.7689304	3090507		
chr15:61967438	C	T	0.4139909	0.01312346	0.00933362	0.1598475	3090507		
chr15:61969413	T	C	0.0050214	-0.0524441	0.0723493	0.4651643	3090507		
chr15:61977193	G	T	8.47E-05	-4.5824861	4.97213523	0.0096656	3090507		
chr15:61983908	T	A	6.01E-05	-0.1637154	0.85567482	0.8349671	3090507		
chr15:61983936	T	C	3.81E-05	-2.8022064	3.08912427	0.2109392	3090507		
chr15:61984885	A	T	0.0002004	-0.4183964	0.42849695	0.2998283	3090507		
chr15:61984938	T	C	5.35E-06	1.08802936	8.79330621	0.9395321	3090507		
chr15:61984953	G	A	7.98E-05	-0.5892673	0.85111623	0.4438224	3090507		
chr15:61984980	G	T	5.19E-06	1.80289236	1.03871579	0.1867404	3090507		
chr15:61991096	T	C	0.000997	-0.2479603	0.1651648	0.1194854	3090507		
chr15:62007466	T	C	2.95E-06	-0.8055396	5.12702777	0.8037764	3090507		
chr15:62008671	T	C	0.0003023	0.11244121	0.28718781	0.6997793	3090507		
chr15:62008730	A	G	8.95E-05	0.62048792	0.51640243	0.2650625	3090507		
chr15:62010533	G	A	0.0001575	0.11716427	0.47108539	0.8162334	3090507		
chr15:62010558	G	C	3.10E-05	1.53885471	0.59625596	0.0371955	3090507		
chr15:62023783	T	C	0.000647	-0.0141419	0.20415179	0.944355	3090507		
chr15:62023836	C	T	0.0222374	-0.0318011	0.03176576	0.3144904	3090507		
chr15:62034998	G	A	0.0002001	0.33554722	0.30747486	0.2992388	3090507		
chr15:89316763	C	A	0.0803592	-0.0083645	0.01708059	0.6239605	3090507		
chr15:89318553	T	C	6.78E-06	-2.0189124	7.36008664	0.6241398	3090507		
chr15:89318581	G	A	2.02E-05	1.10428741	1.01939181	0.3635853	3090507		
chr15:89318587	G	A	0.0001839	0.1337384	0.35766086	0.7138974	3090507		
chr15:89318595	T	C	0.0436634	0.02652365	0.02244382	0.239019	3090507		
chr15:89318599	G	A	7.80E-06	-3.0677629	7.6638296	0.4081332	3090507		
chr15:89318641	G	A	7.81E-06	-3.0001065	8.59723283	0.4723976	3090507		
chr15:89318676	A	G	3.12E-06	-1.0068624	8.49377575	0.8262793	3090507		
chr15:89318677	T	C	3.20E-05	0.09051053	1.02349862	0.9334688	3090507		
chr15:89318710	C	T	8.32E-07	-1.0098018	9.59126634	0.8455297	3090507		

chr15:89318736	C	T	1.19E-05	-3.0280338	8.60957717	0.4682527	3090507	
chr15:89318737	G	A	2.59E-05	-3.0263871	5.9932969	0.2977217	3090507	
chr15:89318986	G	A	1.40E-05	-2.0146667	6.17255551	0.5598204	3090507	
chr15:89319031	A	G	3.74E-06	-1.0102911	6.61204883	0.7773907	3090507	
chr15:89319053	C	G	5.40E-05	0.76392447	0.59710371	0.2530118	3090507	
chr15:89319065	G	A	5.32E-05	0.29749746	0.72719744	0.6968267	3090507	
chr15:89319073	A	G	0.000782	0.09687364	0.17556127	0.5863216	3090507	
chr15:89319234	G	A	0.0001525	-0.5467188	0.52075289	0.2543472	3090507	
chr15:89320832	C	T	1.05E-05	-3.0285972	6.99579324	0.3719291	3090507	
chr15:89320850	A	C	4.55E-05	0.41139458	0.72566373	0.5980296	3090507	
chr15:89320856	C	T	2.29E-06	-1.0103394	8.64769476	0.8288491	3090507	
chr15:89320857	G	A	7.59E-06	-2.0154291	7.54634581	0.6332735	3090507	
chr15:89320917	C	T	2.19E-06	-1.0105494	7.51031244	0.8033935	3090507	
chr15:89320944	T	G	1.56E-06	-1.0193684	8.34981308	0.821575	3090507	
chr15:89321217	G	A	0.0001118	-0.809605	0.43067415	0.0365529	3090507	
chr15:89321223	T	C	1.46E-06	-1.0056251	9.14138652	0.8385702	3090507	
chr15:89321776	C	T	9.37E-07	-1.012954	9.09215344	0.8367563	3090507	
chr15:89321777	G	A	2.34E-05	-4.0562362	6.83353279	0.1222551	3090507	
chr15:89321792	C	T	0.0003426	-0.2419989	0.47479764	0.5998144	3090507	
chr15:89321842	T	C	0.0096919	-0.0324742	0.04941727	0.5090216	3090507	
chr15:89322799	C	T	3.08E-05	-0.6677622	0.76736376	0.330315	3090507	
chr15:89322800	G	A	1.77E-05	-3.027289	6.20970611	0.3147083	3090507	
chr15:89323423	A	G	2.29E-06	-2.0817778	6.45828826	0.5654533	3090507	
chr15:89323426	C	G	0.0005657	0.06260334	0.20112492	0.7579123	3090507	
chr15:89323445	C	T	1.10E-05	1.54721531	0.81250428	0.1046322	3090507	
chr15:89323451	T	C	0.0001702	0.39954563	0.43292942	0.3792007	3090507	
chr15:89323460	C	G	0.0018025	0.04981349	0.10926929	0.6509563	3090507	
chr15:89323462	T	C	0.0002617	-0.1187858	0.37886121	0.7500525	3090507	
chr15:89323504	C	T	9.74E-05	0.10524307	0.5487726	0.8566767	3090507	

chr15:89324193	C	T	0.0003712	-0.3398637	0.44506573	0.4260552	3090507	
chr15:89325531	A	C	1.87E-06	-1.9607276	6.567591	0.5932879	3090507	
chr15:89325562	G	A	4.17E-05	0.22153269	0.72565229	0.7678326	3090507	
chr15:89325591	A	G	3.83E-05	-0.4449275	1.02747869	0.6401725	3090507	
chr15:89325639	G	A	0.0024952	-0.0542974	0.09546468	0.5662449	3090507	
chr15:89326688	G	A	4.58E-05	0.37840145	0.52538373	0.4970483	3090507	
chr15:89326947	C	A	0.007378	-0.0308218	0.05517665	0.5746369	3090507	
chr15:89327004	T	G	0.0001108	0.36826985	0.45816733	0.4508743	3090507	
chr15:89327198	T	C	0.0005243	-0.4931082	0.30492397	0.0840419	3090507	
chr15:89327201	C	T	0.0011787	-0.0119479	0.17164843	0.9441539	3090507	
chr15:89327300	A	G	1.40E-05	-3.0466549	5.97410547	0.2924609	3090507	
chr15:89328532	G	C	0.0010236	-0.0823364	0.12225403	0.4956179	3090507	
chr15:89328699	G	A	1.83E-05	-3.0492101	6.99693288	0.3683331	3090507	
chr15:89328795	T	C	2.71E-06	-1.0122861	6.67597117	0.7791213	3090507	
chr15:89328996	G	A	0.0005451	-0.1897497	0.27591709	0.4814867	3090507	
chr15:89330081	C	G	4.49E-05	-4.0505108	6.231909	0.0908104	3090507	
chr15:89330106	T	A	0.0006062	-0.109195	0.22531018	0.6226144	3090507	
chr15:89330133	C	G	0.0045392	-0.0367403	0.07194313	0.6075646	3090507	
chr15:89330184	G	A	0.0022813	-0.0731574	0.09546287	0.4382558	3090507	
chr15:89330257	G	A	1.25E-05	2.47641045	1.08647889	0.0868007	3090507	
chr15:89330258	C	G	0.0005015	0.13238223	0.20563298	0.5278269	3090507	
chr15:89333327	G	A	1.95E-05	0.64198511	1.04516285	0.5871009	3090507	
chr15:89333357	T	C	8.72E-05	-0.0114906	0.78226086	0.9882115	3090507	
chr15:89333364	A	G	0.0003446	0.07162762	0.27617633	0.8030003	3090507	
chr15:89333621	T	C	9.06E-05	-0.350758	0.47266046	0.4325548	3090507	
chr15:89333627	T	C	0.0033849	-0.0440307	0.08530298	0.603421	3090507	
chr15:89333723	C	T	0.000453	-0.0643827	0.24146902	0.7821106	3090507	
chr16:46674332	T	C	0.0001567	-0.7516981	0.5955734	0.1542053	3090507	
chr16:46683521	A	T	0.0001953	-0.1971899	0.43304584	0.6402435	3090507	

chr1:65366050	A	T	0.0007243	0.25192466	0.17462075	0.1636221	3090507		
chr1:65384226	G	A	2.33E-05	0.3248889	1.13205231	0.7851196	3090507		
chr1:65385740	G	A	0.0006219	0.14661946	0.22032123	0.5137349	3090507		
chr1:65386873	G	A	9.78E-06	1.56601037	1.00913604	0.2266291	3090507		
chr1:65386886	T	C	0.0003241	0.04515017	0.29242438	0.8802374	3090507		
chr1:65392454	T	A	0.0006599	-0.0203366	0.18600062	0.9119366	3090507		
chr1:65392779	A	C	0.0002974	0.0731542	0.31408645	0.8225974	3090507		
chr17:46010389	C	T	5.54E-06	-1.0076379	4.63183194	0.6871511	3090507		
chr1:7961737	T	A	6.85E-05	-1.0354177	0.96740804	0.215283	3090507		
chr1:7965399	G	A	6.05E-05	0.41690908	0.5649158	0.4902636	3090507		
chr1:7984971	G	A	1.66E-06	-1.012361	8.00649559	0.815085	3090507		
chr20:5069196	G	T	0.0018942	-0.1530464	0.15357393	0.3087567	3090507		
chr20:5069288	C	T	0.0009077	-0.1981618	0.21263711	0.3386831	3090507		
chr20:5106224	T	C	0.0013444	-0.0604684	0.14787045	0.6801014	3090507		
chr21:32631012	C	A	1.65E-05	1.17766713	1.07068522	0.3533894	3090507		
chr21:32656885	A	G	2.17E-05	0.81517726	0.95260892	0.4390154	3090507		
chr21:32664963	C	A	0.0003317	0.2898355	0.2428503	0.2515863	3090507		
chr21:32665968	A	G	0.001411	0.01743187	0.1290333	0.8936603	3090507		
chr21:32673463	C	T	9.78E-05	-3.3271818	1.92449228	0.0051399	3090507		
chr21:32673511	T	C	0.0002012	0.07510816	0.41308991	0.8613293	3090507		
chr22:32475369	G	A	1.37E-05	-2.92574	5.17913164	0.4350876	3090507		
chr22:32475378	G	A	0.0998324	-0.0214279	0.01568641	0.1707852	3090507		
chr22:32479132	G	C	0.0001387	0.37755229	0.7287645	0.6270289	3090507		
chr22:32479135	T	G	0.0001783	-0.5311482	0.5080974	0.2574479	3090507		
chr22:32484066	A	G	8.87E-05	-0.0713443	0.81611462	0.9276633	3090507		
chr22:32493191	G	A	0.0001003	0.33765075	0.54714102	0.5560053	3090507		
chr22:32498414	G	A	0.0007226	0.04446187	0.21249862	0.8378807	3090507		
chr22:32498453	C	T	1.97E-05	1.3092649	1.07953012	0.302071	3090507		
chr22:32498513	C	T	0.0001807	-0.6025904	0.55971862	0.2359561	3090507		

chr2:232747717	G	T	0.0001885	0.36394527	0.2566259	0.179125	3090507		
chr2:232747740	A	G	0.0005322	-0.7246152	0.40700493	0.0470567	3090507		
chr2:232790836	G	C	0.00019	0.08849969	0.39552765	0.8310587	3090507		
chr2:232791059	G	C	8.32E-05	-0.4282818	0.80404921	0.5710883	3090507		
chr2:232791124	T	A	0.0005705	0.17397503	0.20566624	0.4089073	3090507		
chr2:232791432	C	T	0.0001357	-0.3193396	0.51064224	0.5089105	3090507		
chr2:232794778	C	T	0.0001662	-0.1669675	0.4742199	0.7188712	3090507		
chr2:232794782	G	C	0.0001151	0.28936001	0.50552878	0.5819459	3090507		
chr2:232794835	A	C	0.0006184	0.46041809	0.30293466	0.1543129	3090507		
chr2:232794843	C	A	0.0193027	-0.0280181	0.03386646	0.4060926	3090507		
chr2:232794852	C	T	4.26E-06	-2.0315939	6.67501327	0.5868034	3090507		
chr2:232811255	C	T	3.13E-05	0.00478931	1.13193591	0.9966338	3090507		
chr2:232819840	G	A	0.0001346	-0.613509	0.57719301	0.2387292	3090507		
chr2:232844373	C	G	0.0019501	0.03063383	0.13882623	0.8280354	3090507		
chr2:232847399	A	G	0.0020986	-0.0630902	0.12002075	0.5958853	3090507		
chr22:38112165	G	C	5.11E-05	0.29522211	0.87677775	0.7468764	3090507		
chr22:38112186	A	T	3.13E-07	-2.1008015	9.46274796	0.692307	3090507		
chr22:38112534	C	G	1.02E-05	-2.0192702	6.14716227	0.5573567	3090507		
chr22:38112541	G	A	5.62E-06	-2.0142143	8.0174309	0.6535498	3090507		
chr22:38112547	G	A	2.81E-06	3.33509372	1.07837575	0.0337175	3090507		
chr22:38112558	C	T	1.04E-05	1.53612608	1.0297825	0.2387584	3090507		
chr22:38113561	G	A	4.58E-06	2.31429714	1.08788334	0.1041689	3090507		
chr22:38113591	G	A	1.98E-06	-1.0208612	7.75627782	0.8079505	3090507		
chr22:38113621	C	T	0.0007414	0.12536694	0.21288826	0.5626659	3090507		
chr22:38115583	G	A	2.19E-06	-1.0218034	6.25717386	0.7630088	3090507		
chr22:38115588	T	G	4.90E-06	-2.0244278	8.78994623	0.6808316	3090507		
chr22:38115619	C	T	1.14E-06	2.72281411	1.25734116	0.0798239	3090507		
chr22:38115658	G	A	4.94E-05	-4.0225965	7.4914571	0.1635617	3090507		
chr22:38116105	C	T	0.0001069	0.06858816	0.49727439	0.8945203	3090507		

chr22:38116155	C	T	2.60E-06	-1.0145734	6.03698986	0.756012	3090507		
chr22:38120802	C	T	2.71E-06	2.13724002	1.00382917	0.1203673	3090507		
chr22:38120867	T	G	5.63E-06	1.6160996	0.71151705	0.0708234	3090507		
chr22:38120886	C	T	0.0012223	0.19418251	0.13891711	0.173637	3090507		
chr22:38120889	G	A	2.49E-05	-3.0286602	5.63124802	0.26733	3090507		
chr22:38123197	G	A	1.46E-06	-1.0101417	8.34104886	0.8226883	3090507		
chr22:38123244	A	T	1.57E-06	-1.0088283	9.25628674	0.8401387	3090507		
chr22:38126371	G	A	0.0001539	-0.3554803	0.51333981	0.464569	3090507		
chr22:38126374	C	T	0.0005062	-0.204125	0.25002867	0.4006754	3090507		
chr22:38126390	T	C	0.0005256	-0.1029393	0.24919838	0.6754311	3090507		
chr22:38126417	G	A	0.0001636	-0.1588846	0.51003791	0.7491426	3090507		
chr22:38126451	T	C	1.24E-05	0.6158819	1.04721089	0.5996585	3090507		
chr22:38132850	G	A	8.59E-06	1.03114553	1.05165776	0.3983968	3090507		
chr22:38132881	C	T	0.0129101	0.0803234	0.04168662	0.0567939	3090507		
chr22:38132914	A	G	1.78E-06	-1.0136125	7.28487246	0.7969486	3090507		
chr22:38132922	C	T	1.97E-05	-3.0279908	6.35045135	0.3254341	3090507		
chr22:38132952	G	A	0.000467	-0.0326932	0.23598789	0.8878905	3090507		
chr22:38133007	G	A	0.00013	-0.7112785	0.70377061	0.2612465	3090507		
chr22:38135029	G	A	3.63E-05	-0.0491584	1.02147257	0.9604036	3090507		
chr22:38140006	G	A	0.0002901	0.13644416	0.2502634	0.5929498	3090507		
chr22:38140069	C	T	0.0001485	0.2229799	0.37779959	0.5678138	3090507		
chr22:38143150	G	A	0.0001422	-0.4469565	0.5836092	0.4117371	3090507		
chr22:38143219	C	G	0.0001615	0.27606809	0.34683848	0.4461185	3090507		
chr22:38145447	C	T	0.0006754	-0.0752615	0.19183763	0.691701	3090507		
chr22:38169240	T	C	6.65E-05	-0.7182033	0.80457412	0.3105178	3090507		
chr22:38169255	C	T	0.0001563	-0.3914547	0.4562904	0.3580547	3090507		
chr22:38169318	G	A	6.12E-05	-0.7623527	1.00219292	0.3734845	3090507		
chr22:38169326	G	A	0.0004895	-0.0357652	0.24909517	0.8836586	3090507		
chr22:38169336	C	T	0.0005463	0.23615398	0.22634475	0.3117081	3090507		

chr22:38169423	G	T	9.61E-06	-2.0281293	7.23464402	0.6166337	3090507	
chr2:74530221	T	C	0.0031936	-0.1334785	0.09185777	0.1381449	3090507	
chr2:74531688	G	A	5.20E-06	-2.0576785	6.53406251	0.574183	3090507	
chr3:132456751	C	T	0.0001701	0.64889352	0.3189446	0.0621823	3090507	
chr3:132466351	A	C	0.0003893	-0.0101092	0.25045499	0.9676127	3090507	
chr3:132467277	G	A	0.0001767	0.24133346	0.35047756	0.5053444	3090507	
chr3:132474976	A	G	3.02E-05	0.71205266	0.70507896	0.3569344	3090507	
chr3:132475009	C	A	0.0072307	-0.0854657	0.06765233	0.201431	3090507	
chr3:132478139	G	A	0.0025755	-0.0532493	0.10496299	0.6092461	3090507	
chr3:132480451	G	A	4.24E-05	0.20940911	0.35596772	0.5678782	3090507	
chr3:132484650	C	T	0.0005666	0.12624636	0.20864833	0.5523638	3090507	
chr3:132492416	G	A	4.78E-06	-1.7918264	6.40955133	0.6138694	3090507	
chr3:132492497	A	G	0.0004756	-0.1178705	0.2983083	0.6876326	3090507	
chr3:132494190	A	G	0.0058262	-0.1308908	0.08526711	0.1169095	3090507	
chr3:132496652	G	A	2.04E-05	-3.050064	5.87872583	0.2841065	3090507	
chr3:132499168	G	A	0.0002195	-0.4497232	0.47336198	0.3099523	3090507	
chr3:132499231	C	T	0.0188252	-0.0402057	0.03441755	0.2398901	3090507	
chr3:132499777	G	A	0.0103685	0.02719621	0.04954825	0.5845226	3090507	
chr3:132499779	G	T	0.4715526	-0.0167987	0.00921669	0.0683121	3090507	
chr3:132502295	C	T	0.0291505	-0.042265	0.02839105	0.1341578	3090507	
chr3:132502298	C	T	4.30E-05	-0.3286353	1.31110402	0.7608173	3090507	
chr3:132502299	G	A	0.0036343	-0.0541347	0.07834375	0.4860553	3090507	
chr3:132507256	A	G	0.062294	0.00151448	0.01926186	0.9373805	3090507	
chr3:132511169	G	C	0.0014927	0.06273496	0.12249566	0.6118547	3090507	
chr3:132516425	G	A	2.05E-05	1.73345469	0.69216971	0.0386218	3090507	
chr3:132516758	G	A	3.61E-05	-3.6261034	5.89304058	0.2215962	3090507	
chr3:132522838	C	T	0.002449	-0.0871381	0.10252428	0.389149	3090507	
chr3:132522861	G	A	3.18E-05	-0.766373	1.3016795	0.5036224	3090507	
chr3:132522950	T	G	0.0007221	-0.3194468	0.21118449	0.1112812	3090507	

chr3:132523168	A	C	6.87E-05	-0.0272387	0.68970192	0.9679893	3090507	
chr3:132523636	G	C	0.0011354	0.23728617	0.14199218	0.1064344	3090507	
chr3:132525718	G	T	0.0010965	-0.0913883	0.15823843	0.5584623	3090507	
chr3:132528231	G	A	2.55E-05	-4.0394606	9.11679238	0.249474	3090507	
chr3:132528316	T	G	0.0045446	-0.1253619	0.07073166	0.0706787	3090507	
chr3:132538225	G	A	0.0033941	-0.1339905	0.09029424	0.1299917	3090507	
chr3:184315839	T	C	0.0005943	-0.2629307	0.25535292	0.2853253	3090507	
chr3:184317393	C	T	2.60E-06	-0.839887	7.28964224	0.8264324	3090507	
chr3:184319745	A	G	0.0025022	-0.0162088	0.11781924	0.8896723	3090507	
chr3:184320694	G	A	0.0002503	0.10592747	0.33331027	0.7540024	3090507	
chr3:184321516	A	G	0.0002663	0.26328599	0.25445772	0.3209223	3090507	
chr3:184321599	G	C	0.0001463	-0.1863028	0.42138284	0.6496002	3090507	
chr3:184321882	C	T	6.11E-05	0.5898979	0.54527588	0.3179537	3090507	
chr3:184322040	C	T	0.0010754	-0.3334418	0.17181391	0.0405679	3090507	
chr3:184322551	C	T	2.35E-05	-0.3481707	1.17087344	0.7547149	3090507	
chr3:184322583	G	C	0.0014552	0.05632442	0.13018434	0.6678104	3090507	
chr3:184322631	C	T	6.17E-05	0.49374816	0.73898249	0.5316937	3090507	
chr3:184322835	C	G	9.36E-06	1.97637425	0.79647163	0.0491199	3090507	
chr3:184323209	G	T	0.0001503	0.17205383	0.42108989	0.6900906	3090507	
chr3:184323412	G	C	0.0001982	0.22576367	0.35831199	0.540107	3090507	
chr3:184323468	G	C	0.0012875	-0.0805128	0.14382624	0.571306	3090507	
chr3:184323541	C	T	3.86E-05	0.39100286	1.01323622	0.7162894	3090507	
chr3:184323921	A	G	0.000522	-0.5410434	0.30509247	0.0553102	3090507	
chr3:184325887	A	G	7.29E-07	-1.0146742	6.79215222	0.7823956	3090507	
chr3:184327401	G	A	0.0003158	-0.0435132	0.37672051	0.9060394	3090507	
chr3:184327433	C	T	0.0001361	0.21559889	0.45969232	0.6477446	3090507	
chr3:184327434	G	A	3.71E-05	-1.9821596	2.0804644	0.1717838	3090507	
chr3:184327609	C	G	0.0050214	0.05384109	0.06788286	0.4312452	3090507	
chr3:184327622	T	C	0.0233611	-0.0275417	0.03122459	0.3757902	3090507	

chr3:184328741	T	C	0.0004132	-0.067798	0.29123238	0.8090425	3090507	
chr3:184331523	C	G	2.09E-06	3.96439747	1.32396513	0.026557	3090507	
chr3:184331762	C	T	0.0001645	-0.7920651	0.59650876	0.1422812	3090507	
chr3:195867623	C	T	0.0171962	0.02663703	0.03561812	0.4563121	3090507	
chr3:195868341	C	T	0.0009693	0.1749797	0.15819633	0.2797899	3090507	
chr4:41261759	A	C	0.0014367	0.09594479	0.1382038	0.4933138	3090507	
chr4:89828149	C	T	9.12E-07	3.63695582	1.32605606	0.0321663	3090507	
chr4:89828156	A	C	0.0002185	0.60577975	0.32993417	0.0863054	3090507	
chr5:122450987	G	A	0.0001285	-0.1743436	0.39847814	0.6540141	3090507	
chr5:1394741	C	G	0.0003035	0.49171865	0.2537488	0.0686599	3090507	
chr5:1403013	G	A	0.0011252	0.11194693	0.18459801	0.5500923	3090507	
chr5:1414780	G	A	4.04E-05	-0.1760679	0.43334149	0.6762379	3090507	
chr5:1432618	G	A	0.001278	-0.0078889	0.13963976	0.9547431	3090507	
chr5:1443128	C	T	0.0003128	0.17940361	0.34199966	0.6069374	3090507	
chr5:176621218	G	T	0.000494	0.08712393	0.35916204	0.8169106	3090507	
chr6:161350125	T	G	6.84E-05	0.97133115	1.02337621	0.4161836	3090507	
chr6:161350187	G	A	0.0021265	0.00441129	0.088163	0.9602011	3090507	
chr6:161350208	C	T	0.000217	0.62131246	0.20650213	0.0054599	3090507	
chr6:161350211	C	T	3.90E-05	-0.1535473	1.01600148	0.8678789	3090507	
chr6:161360129	G	T	5.70E-06	-2.0234737	7.65302154	0.6367606	3090507	
chr6:161360169	G	A	0.0015837	0.12050425	0.1292849	0.3591881	3090507	
chr6:161386864	C	T	2.05E-05	1.79094712	1.0701651	0.1807539	3090507	
chr6:161548861	C	T	3.40E-06	-2.0652088	6.98573662	0.597937	3090507	
chr6:161548937	G	A	2.45E-05	1.32944237	0.89991346	0.2170563	3090507	
chr6:161569358	C	G	0.0003376	-0.3867475	0.32198152	0.2017345	3090507	
chr6:161785805	C	T	2.00E-05	0.54017236	1.08311254	0.644358	3090507	
chr6:161785820	G	A	0.0024927	0.34898922	0.07066524	2.76E-06	3090507	
chr6:161785877	G	A	0.0008159	0.39363785	0.19128192	0.0507228	3090507	
chr6:161973335	C	T	0.0001315	0.75539504	0.91895131	0.4526331	3090507	

chr6:161973401	C	T	4.91E-06	-2.0490152	5.35206232	0.4944276	3090507		
chr6:161973403	T	A	2.11E-05	1.43194726	0.58022492	0.0461347	3090507		
chr6:162443314	A	T	4.00E-05	0.47153141	0.57993281	0.4592275	3090507		
chr6:162443371	G	A	0.0003413	0.04385458	0.23935303	0.8578902	3090507		
chr6:162443383	C	T	1.87E-05	-3.0277358	6.31207686	0.3225375	3090507		
chr6:162443384	G	A	3.64E-06	-2.1015746	6.07886137	0.5378266	3090507		
chrX:121049176	T	G	0.0228635	0.01820622	0.02373192	0.445079	3090507		
chrX:155260942	G	T	2.67E-07	-1.0130378	8.00677621	0.8149924	3090507		

Supplementary Table 5: UKBiobank summary statistics													
**Location**	**REF**	**ALT**	**N_INFORMATIV**	**AF**	**U**	**V**	**STAT**	**DIRECTION**	**EFFECT**	**SE**	**PVALUE**	**minorAllele**	**majorAllele**
chr1:7965399	G	A	45857	0	NA	NA	NA	NA	NA	NA	NA	A	G
chr1:7984930	A	C	45857	0	NA	NA	NA	NA	NA	NA	NA	C	A
chr1:7984971	G	A	45857	0	NA	NA	NA	NA	NA	NA	NA	A	G
chr1:7985019	G	A	45857	0.00040343	1.65211	3.81673	0.715137	#ERROR!	0.432862	0.511864	0.397744	A	G
chr1:16985990	C	T	45857	9.81E-05	1.75147	0.977177	3.13931	#ERROR!	1.79238	1.01161	0.0764263	T	C
chr1:16986065	C	T	45857	0.00021807	-2.18282	2.37407	2.00699	-	-0.919445	0.649013	0.156576	T	C
chr1:16986091	G	A	45857	0.00666201	-14.5681	68.5895	3.0942	-	-0.212395	0.120746	0.0785719	A	G
chr1:16986097	G	A	45857	0.0615173	-16.4508	600.372	0.450767	-	-0.027401	0.0408122	0.501971	A	G
chr1:16986101	T	A	45857	0.0069237	4.77173	68.9193	0.330378	#ERROR!	0.0692365	0.120456	0.565437	A	T
chr1:16986235	C	T	45857	0	NA	NA	NA	NA	NA	NA	NA	T	C
chr1:16986246	G	A	45857	6.54E-05	0.180994	0.662239	0.0494668	#ERROR!	0.273306	1.22883	0.823994	A	G
chr1:16986291	C	T	45857	0.00019626	-0.690066	2.16143	0.220313	-	-0.319264	0.680189	0.638801	T	C
chr1:16986292	G	A	45857	0.00018536	0.540732	1.90365	0.153595	#ERROR!	0.28405	0.72478	0.695123	A	G
chr1:16986321	C	T	45857	1.09E-05	-0.26841	0.196339	0.366935	-	-1.36707	2.25682	0.544679	T	C
chr1:16986334	C	T	45857	2.18E-05	0.702443	0.241322	2.04468	#ERROR!	2.91082	2.03564	0.152739	T	C
chr1:16986335	G	A	45857	0.00110125	-2.85014	12.187	0.666557	-	-0.233868	0.286452	0.414255	A	G
chr1:16986554	G	A	45857	0.00011994	-0.617847	1.31544	0.290197	-	-0.46969	0.871897	0.590094	A	G
chr1:16987089	C	T	45857	0	NA	NA	NA	NA	NA	NA	NA	T	C
chr1:16987157	C	T	45857	1.09E-05	-0.0480824	0.0457648	0.0505174	-	-1.05064	4.67449	0.822165	T	C
chr1:16987187	G	A	45857	0	NA	NA	NA	NA	NA	NA	NA	A	G
chr1:16988161	T	A	45857	0.00186449	7.0786	17.8285	2.81048	#ERROR!	0.397038	0.236833	0.0936505	A	T
chr1:16988226	C	T	45857	0.00027259	5.98532	2.97205	12.0537	#ERROR!	2.01387	0.580059	0.0005169	T	C
chr1:16990191	C	T	45857	3.27E-05	-0.252345	0.227083	0.280419	-	-1.11125	2.0985	0.596427	T	C
chr1:16990276	G	C	45857	0.00091589	-1.1486	9.28379	0.142106	-	-0.123721	0.328199	0.706196	C	G
chr1:16991787	G	A	45857	0	NA	NA	NA	NA	NA	NA	NA	A	G
chr1:16992042	A	G	45857	0	NA	NA	NA	NA	NA	NA	NA	G	A
chr1:16992115	C	T	45857	0	NA	NA	NA	NA	NA	NA	NA	T	C
chr1:16993690	C	T	45857	0	NA	NA	NA	NA	NA	NA	NA	T	C
chr1:16996118	A	T	45857	0	NA	NA	NA	NA	NA	NA	NA	T	A
chr1:16996298	G	C	45857	0.00190811	-3.94555	20.8201	0.74771	-	-0.189507	0.219159	0.387202	C	G
chr1:16996484	G	T	45857	0	NA	NA	NA	NA	NA	NA	NA	T	G
chr1:16997044	C	T	45857	0.00028349	-1.20892	2.62118	0.557569	-	-0.461213	0.617663	0.455241	T	C
chr1:16997107	G	A	45857	0	NA	NA	NA	NA	NA	NA	NA	A	G
chr1:16997136	C	T	45857	0	NA	NA	NA	NA	NA	NA	NA	T	C
chr1:17000107	C	T	45857	5.45E-05	-0.793754	0.632942	0.995423	-	-1.25407	1.25695	0.31842	T	C
chr1:17000272	C	T	45857	0.0155047	-14.7562	160.152	1.35961	-	-0.0921385	0.0790193	0.243604	T	C
chr1:17000494	G	A	45857	8.72E-05	-0.126592	0.933204	0.0171726	-	-0.135653	1.03517	0.89574	A	G
chr1:17000495	C	T	45857	2.18E-05	1.80528	0.164792	19.7765	#ERROR!	10.9549	2.46338	8.70E-06	T	C
chr1:17004708	G	A	45857	3.27E-05	-0.348774	0.30411	0.399999	-	-1.14687	1.81336	0.52709	A	G
chr1:17005517	C	T	45857	2.18E-05	-0.344298	0.27158	0.436487	-	-1.26776	1.9189	0.508823	T	C
chr1:17005754	G	A	45857	0.00021807	1.03518	2.28277	0.469426	#ERROR!	0.453474	0.661864	0.493251	A	G
chr1:17011732	C	G	45857	0	NA	NA	NA	NA	NA	NA	NA	G	C
chr1:20633615	G	A	45857	0	NA	NA	NA	NA	NA	NA	NA	A	G

chr1:20633766	C	T	45857	0	NA	NA	NA	NA	NA	NA	NA	T	C
chr1:20633840	C	T	45857	0	NA	NA	NA	NA	NA	NA	NA	T	C
chr1:20637888	C	T	45857	2.18E-05	-0.389302	0.304841	0.497164	-	-1.27707	1.81119	0.480749	T	C
chr1:20637894	G	A	45857	1.09E-05	-0.25946	0.192091	0.350455	-	-1.35071	2.28163	0.553856	A	G
chr1:20637908	C	T	45857	1.09E-05	-0.203059	0.161804	0.254833	-	-1.25497	2.48602	0.613692	T	C
chr1:20638080	C	T	45857	0.00015265	-1.06203	1.21619	0.927411	-	-0.873245	0.906776	0.335537	T	C
chr1:20639911	C	T	45857	0	NA	NA	NA	NA	NA	NA	NA	T	C
chr1:20639934	G	A	45857	0	NA	NA	NA	NA	NA	NA	NA	A	G
chr1:20644515	C	G	45857	1.09E-05	0.932832	0.0626528	13.8888	#ERROR!	14.8889	3.99512	0.00019395	G	C
chr1:20644540	G	A	45857	0	NA	NA	NA	NA	NA	NA	NA	A	G
chr1:20644549	G	A	45857	7.63E-05	-0.929036	0.732132	1.1789	-	-1.26895	1.16871	0.277581	A	G
chr1:20644551	G	A	45857	1.09E-05	-0.117792	0.103908	0.133531	-	-1.13362	3.10224	0.714798	A	G
chr1:20644564	C	A	45857	0	NA	NA	NA	NA	NA	NA	NA	A	C
chr1:20644570	C	T	45857	1.09E-05	-0.0891238	0.0811698	0.0978573	-	-1.09799	3.50997	0.754416	T	C
chr1:20644626	C	G	45857	0	NA	NA	NA	NA	NA	NA	NA	G	C
chr1:20644651	C	T	45857	1.09E-05	0.955128	0.0428559	21.2869	#ERROR!	22.287	4.83053	3.95E-06	T	C
chr1:20644662	G	A	45857	4.36E-05	1.56349	0.378173	6.46402	#ERROR!	4.13434	1.62613	0.011008	A	G
chr1:20644665	A	T	45857	0.0006324	-0.933004	6.83605	0.127339	-	-0.136483	0.38247	0.721207	T	A
chr1:20645615	G	A	45857	0.00213708	1.79439	22.968	0.140189	#ERROR!	0.0781259	0.20866	0.708093	A	G
chr1:20645640	T	C	45857	0	NA	NA	NA	NA	NA	NA	NA	C	T
chr1:20645675	G	A	45857	0	NA	NA	NA	NA	NA	NA	NA	A	G
chr1:20648528	G	A	45857	0.00022897	2.08147	2.13651	2.02784	#ERROR!	0.974235	0.684144	0.15444	A	G
chr1:20648534	T	C	45857	0	NA	NA	NA	NA	NA	NA	NA	C	T
chr1:20648577	C	T	45857	0	NA	NA	NA	NA	NA	NA	NA	T	C
chr1:20648601	G	A	45857	0	NA	NA	NA	NA	NA	NA	NA	A	G
chr1:20648612	G	A	45857	0.00049066	-2.09734	5.24301	0.838988	-	-0.400025	0.436726	0.359686	A	G
chr1:20649054	G	A	45857	0	NA	NA	NA	NA	NA	NA	NA	A	G
chr1:20649095	A	G	45857	2.18E-05	0.877884	0.114458	6.73329	#ERROR!	7.66991	2.95581	0.00946297	G	A
chr1:20649109	C	T	45857	4.36E-05	-0.426598	0.372255	0.488874	-	-1.14598	1.639	0.48443	T	C
chr1:20649134	G	A	45857	5.45E-05	-0.011677	0.786395	0.00017339	-	-0.0148487	1.12766	0.989494	A	G
chr1:20649217	C	T	45857	8.72E-05	-0.0398376	0.875271	0.00181319	-	-0.0455145	1.06888	0.966035	T	C
chr1:20649224	C	T	45857	0	NA	NA	NA	NA	NA	NA	NA	T	C
chr1:65366050	A	T	45857	0.0006324	0.322407	7.42159	0.0140059	#ERROR!	0.0434418	0.367072	0.905793	T	A
chr1:65384226	G	A	45857	1.09E-05	0.0807867	0.07424	0.0879107	#ERROR!	1.08818	3.67013	0.76685	A	G
chr1:65385740	G	A	45857	0.0003053	-1.13414	2.96585	0.433699	-	-0.382402	0.580665	0.51018	A	G
chr1:65385756	G	A	45857	2.18E-05	-0.265753	0.211956	0.333203	-	-1.25381	2.17209	0.563779	A	G
chr1:65386820	G	A	45857	0.00011994	0.448378	1.17007	0.171821	#ERROR!	0.383206	0.924473	0.678498	A	G
chr1:65386873	G	A	45857	4.36E-05	0.548828	0.39354	0.76539	#ERROR!	1.39459	1.59406	0.381647	A	G
chr1:65386886	T	C	45857	0.0003053	-0.999082	3.06547	0.325616	-	-0.325915	0.571152	0.568252	C	T
chr1:65392454	T	A	45857	0.00056698	2.22539	5.75561	0.860438	#ERROR!	0.386646	0.416825	0.353616	A	T
chr1:65392613	C	A	45857	0	NA	NA	NA	NA	NA	NA	NA	A	C
chr1:65392779	A	C	45857	0.00013084	-1.85455	1.6069	2.14036	-	-1.15411	0.788871	0.143468	C	A
chr1:65394955	G	A	45857	0	NA	NA	NA	NA	NA	NA	NA	A	G
chr1:65401812	C	T	45857	2.18E-05	0.913367	0.0828722	10.0666	#ERROR!	11.0214	3.47373	0.00150983	T	C
chr1:155235195	C	T	45857	0	NA	NA	NA	NA	NA	NA	NA	T	C

chr1:155235196	G	A	45857	0.00050156	8.01728	4.74533	13.5453	#ERROR!	1.68951	0.459057	0.00023288	A	G
chr1:155235197	G	C	45857	1.09E-05	-0.0875091	0.07984	0.0959149	-	-1.09606	3.53908	0.756788	C	G
chr1:155235727	C	G	45857	5.45E-05	-0.101384	0.537707	0.019116	-	-0.18855	1.36373	0.890034	G	C
chr1:155235772	C	A	45857	0	NA	NA	NA	NA	NA	NA	NA	A	C
chr1:155235810	C	T	45857	0	NA	NA	NA	NA	NA	NA	NA	T	C
chr1:155235813	T	G	45857	2.18E-05	0.885764	0.107252	7.31529	#ERROR!	8.25874	3.0535	0.00683705	G	T
chr1:155235814	C	T	45857	1.09E-05	-0.146644	0.125123	0.171866	-	-1.172	2.82703	0.678459	T	C
chr1:155235823	C	T	45857	3.27E-05	0.553158	0.335658	0.911595	#ERROR!	1.64798	1.72604	0.339691	T	C
chr1:155235843	T	C	45857	0.00138474	11.9716	13.7388	10.4318	#ERROR!	0.871374	0.26979	0.00123866	C	T
chr1:155236246	G	A	45857	0.00746887	25.8021	76.7389	8.6755	#ERROR!	0.336232	0.114154	0.00322516	A	G
chr1:155236269	C	T	45857	0	NA	NA	NA	NA	NA	NA	NA	T	C
chr1:155236277	G	A	45857	1.09E-05	-0.146677	0.125148	0.171911	-	-1.17203	2.82676	0.678419	A	G
chr1:155236367	G	A	45857	0	NA	NA	NA	NA	NA	NA	NA	A	G
chr1:155236376	C	T	45857	0.0149159	44.2266	156.318	12.5129	#ERROR!	0.282927	0.0799825	0.00040415	T	C
chr1:155236384	G	A	45857	2.18E-05	-0.451423	0.343579	0.593117	-	-1.31388	1.70603	0.441216	A	G
chr1:155236409	C	G	45857	3.27E-05	1.59047	0.340377	7.43176	#ERROR!	4.67268	1.71404	0.00640828	G	C
chr1:155236415	A	G	45857	0	NA	NA	NA	NA	NA	NA	NA	G	A
chr1:155237357	G	A	45857	0	NA	NA	NA	NA	NA	NA	NA	A	G
chr1:155237369	C	T	45857	0	NA	NA	NA	NA	NA	NA	NA	T	C
chr1:155237370	G	A	45857	3.27E-05	-0.379447	0.330977	0.435016	-	-1.14645	1.73821	0.509538	A	G
chr1:155237423	G	A	45857	0.00011994	-0.535542	1.08797	0.263615	-	-0.49224	0.95872	0.607647	A	G
chr1:155237438	C	T	45857	0.0003053	1.53794	3.59795	0.657391	#ERROR!	0.427449	0.527196	0.417483	T	C
chr1:155237444	A	G	45857	0	NA	NA	NA	NA	NA	NA	NA	G	A
chr1:155237453	C	T	45857	0.00011994	0.914738	0.94036	0.889815	#ERROR!	0.972754	1.03122	0.345527	T	C
chr1:155237458	A	C	45857	9.81E-05	0.925062	0.90517	0.945391	#ERROR!	1.02198	1.05108	0.330895	C	A
chr1:155237576	A	T	45857	2.18E-05	-0.230105	0.195391	0.270986	-	-1.17766	2.26229	0.602671	T	A
chr1:155238194	C	T	45857	0	NA	NA	NA	NA	NA	NA	NA	T	C
chr1:155238215	T	C	45857	3.27E-05	0.307621	0.471417	0.200737	#ERROR!	0.652546	1.45646	0.654126	C	T
chr1:155238246	G	A	45857	1.09E-05	0.319442	0.217188	0.469838	#ERROR!	1.47081	2.14576	0.493062	A	G
chr1:155238251	G	T	45857	1.09E-05	-0.137083	0.118275	0.158881	-	-1.15902	2.90773	0.690189	T	G
chr1:155238264	C	T	45857	0	NA	NA	NA	NA	NA	NA	NA	T	C
chr1:155238570	C	G	45857	0.00013084	1.44434	1.66313	1.25433	#ERROR!	0.868446	0.77542	0.262727	G	C
chr1:155238629	C	T	45857	2.18E-05	-0.513996	0.379207	0.696696	-	-1.35545	1.62391	0.403896	T	C
chr1:155239633	G	A	45857	3.27E-05	2.57575	0.34405	19.2835	#ERROR!	7.48656	1.70486	1.13E-05	A	G
chr1:155239639	A	C	45857	2.18E-05	0.641708	0.330348	1.24653	#ERROR!	1.94252	1.73986	0.264217	C	A
chr1:155239685	C	T	45857	0	NA	NA	NA	NA	NA	NA	NA	T	C
chr1:155239736	G	A	45857	1.09E-05	-0.179216	0.14705	0.218418	-	-1.21874	2.60776	0.640248	A	G
chr1:155239933	C	T	45857	1.09E-05	-0.0669351	0.0624447	0.0717484	-	-1.07191	4.00177	0.788808	T	C
chr1:155239939	C	T	45857	0	NA	NA	NA	NA	NA	NA	NA	T	C
chr1:155240048	C	T	45857	0	NA	NA	NA	NA	NA	NA	NA	T	C
chr1:155240072	G	A	45857	0	NA	NA	NA	NA	NA	NA	NA	A	G
chr1:175386035	T	C	45857	0.00115577	-0.433286	11.2561	0.0166787	-	-0.0384935	0.298062	0.897242	C	T
chr1:175403578	T	G	45857	0.0059751	-3.2566	63.3313	0.16746	-	-0.0514216	0.125658	0.682379	G	T
chr1:175406219	T	C	45857	0.00493927	-6.29569	49.4875	0.800924	-	-0.127218	0.142152	0.370817	C	T
chr1:175406252	A	T	45857	5.45E-05	-0.722319	0.601086	0.868004	-	-1.20169	1.28983	0.351509	T	A

chr2:74530119	T	C	45857	0	NA	NA	NA	NA	NA	NA	NA	C	T
chr2:74530208	C	T	45857	0	NA	NA	NA	NA	NA	NA	NA	T	C
chr2:74530221	T	C	45857	0.00430687	-1.23779	43.507	0.0352155	-	-0.0284504	0.151607	0.851145	C	T
chr2:74530433	C	G	45857	1.09E-05	0.103315	0.0926056	0.115263	#ERROR!	1.11565	3.2861	0.73423	G	C
chr2:74531688	G	A	45857	0	NA	NA	NA	NA	NA	NA	NA	A	G
chr2:74532831	G	A	45857	0	NA	NA	NA	NA	NA	NA	NA	A	G
chr2:232747646	A	G	45857	0	NA	NA	NA	NA	NA	NA	NA	G	A
chr2:232747717	G	T	45857	1.09E-05	-0.211138	0.166523	0.267707	-	-1.26792	2.45054	0.604874	T	G
chr2:232747740	A	G	45857	0.00052337	-2.19196	5.54722	0.866141	-	-0.395145	0.424583	0.352026	G	A
chr2:232748987	A	G	45857	0	NA	NA	NA	NA	NA	NA	NA	G	A
chr2:232790817	A	G	45857	0	NA	NA	NA	NA	NA	NA	NA	G	A
chr2:232790836	G	C	45857	0.00014175	-0.101297	1.71012	0.00600017	-	-0.0592336	0.764692	0.938257	C	G
chr2:232791059	G	C	45857	4.36E-05	0.441238	0.466822	0.417056	#ERROR!	0.945196	1.46361	0.51841	C	G
chr2:232791124	T	A	45857	0.00047975	-0.431054	4.63279	0.040107	-	-0.0930441	0.464599	0.841272	A	T
chr2:232791432	C	T	45857	6.54E-05	0.437627	0.49905	0.383764	#ERROR!	0.876921	1.41556	0.535596	T	C
chr2:232794778	C	T	45857	8.72E-05	1.99533	0.812663	4.89914	#ERROR!	2.4553	1.10929	0.0268701	T	C
chr2:232794782	G	C	45857	0.00040343	2.15292	4.35665	1.0639	#ERROR!	0.494168	0.479097	0.302327	C	G
chr2:232794835	A	C	45857	0.00055608	-0.8046	6.25916	0.103429	-	-0.128548	0.399707	0.747752	C	A
chr2:232794852	C	T	45857	2.18E-05	-0.219336	0.186342	0.258172	-	-1.17706	2.31656	0.611379	T	C
chr2:232811255	C	T	45857	0	NA	NA	NA	NA	NA	NA	NA	T	C
chr2:232811303	A	G	45857	0	NA	NA	NA	NA	NA	NA	NA	G	A
chr2:232819840	G	A	45857	0.00014175	-1.89764	1.54325	2.33341	-	-1.22964	0.804974	0.126624	A	G
chr2:232832882	G	A	45857	0.0003053	-0.184502	2.76508	0.012311	-	-0.0667255	0.601376	0.911652	A	G
chr2:232839964	G	A	45857	0.00077415	0.805484	8.06156	0.0804813	#ERROR!	0.0999167	0.352201	0.776646	A	G
chr2:232844373	C	G	45857	0.00203895	-7.53058	20.9732	2.70391	-	-0.359057	0.218357	0.100103	G	C
chr2:232847399	A	G	45857	0.00214798	8.18276	23.7442	2.81995	#ERROR!	0.344621	0.205221	0.0930992	G	A
chr3:132450732	A	T	45857	0.00023988	2.97067	2.68904	3.28179	#ERROR!	1.10473	0.60982	0.0700526	T	A
chr3:132453603	C	G	45857	0	NA	NA	NA	NA	NA	NA	NA	G	C
chr3:132454135	C	T	45857	4.36E-05	-0.166272	0.436539	0.0633306	-	-0.380886	1.51352	0.801307	T	C
chr3:132456751	C	T	45857	8.72E-05	-0.192661	0.963661	0.0385178	-	-0.199926	1.01868	0.844407	T	C
chr3:132457295	T	C	45857	0	NA	NA	NA	NA	NA	NA	NA	C	T
chr3:132466351	A	C	45857	0.00041433	-0.202417	4.74007	0.00864389	-	-0.0427034	0.459312	0.925925	C	A
chr3:132467277	G	A	45857	0.00019626	-1.61277	2.15345	1.20784	-	-0.748923	0.681447	0.27176	A	G
chr3:132474976	A	G	45857	1.09E-05	-0.17844	0.146579	0.217227	-	-1.21737	2.61195	0.641161	G	A
chr3:132475009	C	A	45857	0.0107181	4.19506	108.336	0.162444	#ERROR!	0.0387228	0.0960759	0.686916	A	C
chr3:132477807	G	A	45857	0	NA	NA	NA	NA	NA	NA	NA	A	G
chr3:132478139	G	A	45857	0.00342369	9.61745	34.1953	2.70491	#ERROR!	0.28125	0.171008	0.10004	A	G
chr3:132480451	G	A	45857	6.54E-05	-0.218366	0.947901	0.0503044	-	-0.230368	1.02711	0.822534	A	G
chr3:132484650	C	T	45857	0.00066511	-0.39947	5.8608	0.0272278	-	-0.0681597	0.413068	0.868937	T	C
chr3:132492416	G	A	45857	1.09E-05	-0.14751	0.125725	0.17307	-	-1.17328	2.82026	0.677397	A	G
chr3:132492497	A	G	45857	0.00025078	2.04355	2.30903	1.80859	#ERROR!	0.885024	0.658089	0.178677	G	A
chr3:132494190	A	G	45857	0.0075561	3.24439	76.5736	0.137463	#ERROR!	0.0423696	0.114277	0.710816	G	A
chr3:132496652	G	A	45857	0	NA	NA	NA	NA	NA	NA	NA	A	G
chr3:132499168	G	A	45857	0.00022897	1.70324	2.41982	1.19886	#ERROR!	0.70387	0.642849	0.27355	A	G
chr3:132499231	C	T	45857	0.0178381	-9.03982	178.693	0.457311	-	-0.0505885	0.0748077	0.498883	T	C

chr3:132499777	G	A	45857	0.0116885	13.5569	125.058	1.46963	#ERROR!	0.108405	0.089422	0.225403	A	G
chr3:132502295	C	T	45857	0.0273459	-8.17331	271.587	0.245973	-	-0.0300946	0.06068	0.619925	T	C
chr3:132502298	C	T	45857	3.27E-05	-0.511317	0.408988	0.639249	-	-1.2502	1.56367	0.423983	T	C
chr3:132502299	G	A	45857	0.00424145	-0.876372	43.9178	0.0174878	-	-0.0199548	0.150897	0.894793	A	G
chr3:132503289	G	A	45857	0	NA	NA	NA	NA	NA	NA	NA	A	G
chr3:132505400	A	T	45857	1.09E-05	-0.0318989	0.0309712	0.0328543	-	-1.02995	5.68226	0.856165	T	A
chr3:132507256	A	G	45857	0.0541575	16.4237	531.622	0.507385	#ERROR!	0.0308935	0.0433709	0.476273	G	A
chr3:132511169	G	C	45857	0.00195172	6.86327	18.8079	2.5045	#ERROR!	0.364914	0.230584	0.113521	C	G
chr3:132516758	G	A	45857	0	NA	NA	NA	NA	NA	NA	NA	A	G
chr3:132516789	G	C	45857	0	NA	NA	NA	NA	NA	NA	NA	C	G
chr3:132522832	G	A	45857	3.27E-05	0.19718	0.429987	0.0904217	#ERROR!	0.458573	1.52501	0.763642	A	G
chr3:132522838	C	T	45857	0.00331465	13.3524	35.7551	4.98632	#ERROR!	0.37344	0.167236	0.0255485	T	C
chr3:132522861	G	A	45857	0	NA	NA	NA	NA	NA	NA	NA	A	G
chr3:132522950	T	G	45857	0.00090499	2.52387	9.14104	0.696848	#ERROR!	0.276103	0.330752	0.403845	G	T
chr3:132523168	A	C	45857	2.18E-05	-0.222256	0.18843	0.262155	-	-1.17952	2.3037	0.608643	C	A
chr3:132523636	G	C	45857	0.00095951	-5.7056	10.6697	3.05105	-	-0.534747	0.306142	0.080685	C	G
chr3:132525718	G	T	45857	0.00115577	-0.193157	11.8641	0.00314473	-	-0.0162807	0.290323	0.95528	T	G
chr3:132528231	G	A	45857	6.54E-05	-0.733064	0.601814	0.892939	-	-1.21809	1.28905	0.344682	A	G
chr3:132528316	T	G	45857	0.00465578	-2.32237	48.6323	0.110902	-	-0.0477537	0.143396	0.73912	G	T
chr3:132538225	G	A	45857	0.00383802	-5.25862	39.9859	0.691571	-	-0.131512	0.158142	0.405631	A	G
chr3:184315839	T	C	45857	0.00066511	1.58197	7.21664	0.346784	#ERROR!	0.219211	0.372248	0.555939	C	T
chr3:184319745	G	A	45857	0.00437229	1.09254	45.2252	0.0263934	#ERROR!	0.0241578	0.1487	0.870943	A	G
chr3:184320694	G	A	45857	0.00059969	-2.56525	6.1399	1.07176	-	-0.417799	0.403571	0.300549	A	G
chr3:184320964	C	T	45857	0	NA	NA	NA	NA	NA	NA	NA	T	C
chr3:184321315	G	A	45857	1.09E-05	-0.284917	0.203675	0.398566	-	-1.39888	2.21581	0.527831	A	G
chr3:184321345	C	G	45857	0	NA	NA	NA	NA	NA	NA	NA	G	C
chr3:184321454	G	A	45857	0	NA	NA	NA	NA	NA	NA	NA	A	G
chr3:184321497	C	T	45857	3.27E-05	0.821929	0.16584	4.07362	#ERROR!	4.95616	2.45559	0.0435579	T	C
chr3:184321498	G	A	45857	0	NA	NA	NA	NA	NA	NA	NA	A	G
chr3:184321599	G	C	45857	0.00019626	-1.76471	1.55272	2.00564	-	-1.13653	0.802516	0.156716	C	G
chr3:184321648	C	T	45857	0	NA	NA	NA	NA	NA	NA	NA	T	C
chr3:184321803	G	A	45857	0	NA	NA	NA	NA	NA	NA	NA	A	G
chr3:184321882	C	T	45857	4.36E-05	-0.161259	0.426368	0.0609909	-	-0.378216	1.53147	0.804937	T	C
chr3:184322040	C	T	45857	0.00143926	5.19647	14.4519	1.86849	#ERROR!	0.359569	0.263049	0.171648	T	C
chr3:184322551	C	T	45857	0	NA	NA	NA	NA	NA	NA	NA	T	C
chr3:184322583	G	C	45857	0.00167913	5.06662	17.3355	1.48081	#ERROR!	0.292269	0.240177	0.223647	C	G
chr3:184322631	C	T	45857	5.45E-05	-0.395215	0.346137	0.451253	-	-1.14179	1.69972	0.50174	T	C
chr3:184322835	C	G	45857	3.27E-05	-0.254909	0.21993	0.295452	-	-1.15905	2.13235	0.586748	G	C
chr3:184323209	G	T	45857	0.00040343	0.992524	3.5305	0.279027	#ERROR!	0.281128	0.532208	0.59734	T	G
chr3:184323412	G	C	45857	0.00038162	0.340209	4.33072	0.0267259	#ERROR!	0.0785571	0.480529	0.87014	C	G
chr3:184323468	G	C	45857	0.00185359	5.97764	18.7662	1.90407	#ERROR!	0.318532	0.23084	0.167623	C	G
chr3:184323541	C	T	45857	0	NA	NA	NA	NA	NA	NA	NA	T	C
chr3:184323921	A	G	45857	0.00090499	-5.26002	9.70992	2.84944	-	-0.541716	0.320917	0.091406	G	A
chr3:184327230	G	A	45857	1.09E-05	-0.265617	0.195014	0.361783	-	-1.36205	2.26448	0.547518	A	G
chr3:184327329	C	G	45857	0	NA	NA	NA	NA	NA	NA	NA	G	C

chr3:184327401	G	A	45857	0.00034891	2.45375	3.55543	1.69344	#ERROR!	0.690143	0.530339	0.193148	A	G
chr3:184327433	C	T	45857	5.45E-05	-0.0548261	0.797612	0.00376863	-	-0.0687379	1.11971	0.951049	T	C
chr3:184327434	G	A	45857	0	NA	NA	NA	NA	NA	NA	NA	A	G
chr3:184327609	C	G	45857	0.00459036	1.98097	45.4854	0.0862748	#ERROR!	0.0435518	0.148274	0.768967	G	C
chr3:184327622	T	C	45857	0.0262337	-10.5593	263.951	0.422419	-	-0.0400046	0.0615514	0.515733	C	T
chr3:184327628	C	T	45857	4.36E-05	-0.595668	0.471568	0.752426	-	-1.26316	1.45622	0.385709	T	C
chr3:184327694	A	G	45857	4.36E-05	-0.490811	0.396833	0.607044	-	-1.23682	1.58744	0.435903	G	A
chr3:184328662	A	G	45857	0.00014175	-1.73334	1.41856	2.11798	-	-1.22191	0.839608	0.145578	G	A
chr3:184328664	G	A	45857	0	NA	NA	NA	NA	NA	NA	NA	A	G
chr3:184328741	T	C	45857	0.00068692	-0.706096	6.85376	0.0727442	-	-0.103023	0.381975	0.787382	C	T
chr3:184331523	C	G	45857	0	NA	NA	NA	NA	NA	NA	NA	G	C
chr3:184331762	C	T	45857	0.00021807	0.191748	2.18265	0.0168453	#ERROR!	0.0878513	0.676875	0.896733	T	C
chr3:195867623	C	T	45857	0.0198116	-4.73527	204.658	0.109562	-	-0.0231375	0.0699014	0.740643	T	C
chr3:195868341	C	T	45857	0.00088318	3.5745	10.0392	1.27272	#ERROR!	0.356056	0.31561	0.259257	T	C
chr4:41261759	A	C	45857	0.00177726	-3.78937	17.6703	0.812628	-	-0.214449	0.237891	0.367344	C	A
chr4:41261921	C	T	45857	0	NA	NA	NA	NA	NA	NA	NA	T	C
chr4:41263258	T	C	45857	0	NA	NA	NA	NA	NA	NA	NA	C	T
chr4:89729235	G	A	45857	0	NA	NA	NA	NA	NA	NA	NA	A	G
chr4:89828156	A	C	45857	0.00028349	1.80124	2.69737	1.20282	#ERROR!	0.667776	0.608877	0.272758	C	A
chr5:1394741	C	G	45857	0.00051246	1.62556	5.63599	0.468852	#ERROR!	0.288425	0.421226	0.493516	G	C
chr5:1403013	G	A	45857	0.00204985	15.8755	20.2897	12.4217	#ERROR!	0.782443	0.222005	0.00042437	A	G
chr5:1414780	G	A	45857	2.18E-05	-0.247019	0.215658	0.28294	-	-1.14542	2.15336	0.594781	A	G
chr5:1432618	G	A	45857	0.00139564	0.426253	14.1096	0.0128772	#ERROR!	0.0302101	0.266221	0.909652	A	G
chr5:1443128	C	T	45857	0.00074144	-1.26546	7.41116	0.216079	-	-0.170751	0.36733	0.642044	T	C
chr5:122450987	G	A	45857	3.27E-05	-0.398759	0.320743	0.495752	-	-1.24324	1.76572	0.481373	A	G
chr5:176621218	G	T	45857	0.00017446	0.332135	1.42638	0.0773378	#ERROR!	0.232851	0.837301	0.780938	T	G
chr6:161350125	T	G	45857	0.00014175	-0.254182	1.25298	0.0515639	-	-0.202862	0.893364	0.820364	G	T
chr6:161350187	G	A	45857	0.00369627	-9.00428	39.13	2.07199	-	-0.230112	0.159862	0.150026	A	G
chr6:161350208	C	T	45857	0.0003162	2.91752	3.43384	2.47884	#ERROR!	0.849639	0.539647	0.115387	T	C
chr6:161350211	C	T	45857	0	NA	NA	NA	NA	NA	NA	NA	T	C
chr6:161360168	C	T	45857	3.27E-05	-0.38424	0.302375	0.48827	-	-1.27074	1.81856	0.4847	T	C
chr6:161360169	G	A	45857	0.00147197	4.17927	14.3533	1.21688	#ERROR!	0.291171	0.263951	0.269974	A	G
chr6:161360187	T	C	45857	0	NA	NA	NA	NA	NA	NA	NA	C	T
chr6:161386864	C	T	45857	3.27E-05	-0.390307	0.328478	0.463774	-	-1.18823	1.74481	0.495865	T	C
chr6:161548861	C	T	45857	0	NA	NA	NA	NA	NA	NA	NA	T	C
chr6:161548937	G	A	45857	0	NA	NA	NA	NA	NA	NA	NA	A	G
chr6:161569358	C	G	45857	0.00086137	-3.72923	8.63239	1.61104	-	-0.432004	0.340357	0.204345	G	C
chr6:161785793	C	G	45857	0	NA	NA	NA	NA	NA	NA	NA	G	C
chr6:161785805	C	T	45857	0.00013084	0.488111	1.42564	0.167119	#ERROR!	0.342379	0.837519	0.682685	T	C
chr6:161785820	G	A	45857	0.00387073	-2.2543	38.9966	0.130316	-	-0.0578077	0.160135	0.718105	A	G
chr6:161785825	T	C	45857	7.63E-05	0.720422	1.01316	0.512267	#ERROR!	0.711065	0.993484	0.474159	C	T
chr6:161785839	A	T	45857	0	NA	NA	NA	NA	NA	NA	NA	T	A
chr6:161785844	A	G	45857	0	NA	NA	NA	NA	NA	NA	NA	G	A
chr6:161785877	G	A	45857	0.00079595	-1.66494	7.82608	0.354205	-	-0.212743	0.35746	0.551742	A	G
chr6:161973335	C	T	45857	5.45E-05	-0.650495	0.551778	0.766875	-	-1.17891	1.34623	0.381186	T	C

chr6:161973347	G	A	45857	0	NA	NA	NA	NA	NA	NA	NA	A	G
chr6:161973401	C	T	45857	1.09E-05	-0.044885	0.0428841	0.0469793	-	-1.04666	4.82894	0.828405	T	C
chr6:161973403	T	A	45857	2.18E-05	0.63944	0.29269	1.39698	#ERROR!	2.1847	1.8484	0.237229	A	T
chr6:162201237	T	C	45857	0	NA	NA	NA	NA	NA	NA	NA	C	T
chr6:162262627	G	A	45857	0	NA	NA	NA	NA	NA	NA	NA	A	G
chr6:162262681	C	T	45857	3.27E-05	-0.278422	0.229749	0.337407	-	-1.21185	2.08628	0.56133	T	C
chr6:162443314	A	T	45857	1.09E-05	-0.0322742	0.0312308	0.0333524	-	-1.03341	5.65859	0.855091	T	A
chr6:162443357	G	A	45857	2.18E-05	-0.293294	0.239321	0.359439	-	-1.22553	2.04413	0.548818	A	G
chr6:162443371	G	A	45857	0.00059969	-2.51686	6.39703	0.990235	-	-0.393441	0.395376	0.319685	A	G
chr6:162443383	C	T	45857	0.00020717	-2.02291	1.87645	2.1808	-	-1.07805	0.730015	0.139741	T	C
chr6:162443384	G	A	45857	1.09E-05	0.967052	0.0318606	29.3526	#ERROR!	30.3526	5.60239	6.03E-08	A	G
chr6:162443429	C	T	45857	0	NA	NA	NA	NA	NA	NA	NA	T	C
chr11:94447275	C	T	45857	0.00013084	-0.171112	1.00301	0.0291914	-	-0.170598	0.998499	0.864338	T	C
chr12:40225558	C	T	45857	0	NA	NA	NA	NA	NA	NA	NA	T	C
chr12:40225561	A	G	45857	0	NA	NA	NA	NA	NA	NA	NA	G	A
chr12:40232383	A	G	45857	1.09E-05	-0.179398	0.147139	0.21873	-	-1.21924	2.60697	0.640009	G	A
chr12:40232385	T	C	45857	1.09E-05	-0.179466	0.147139	0.218897	-	-1.21971	2.60697	0.639882	C	T
chr12:40235634	T	C	45857	0.0022134	3.94293	22.9704	0.676814	#ERROR!	0.171652	0.208648	0.410686	C	T
chr12:40240535	G	T	45857	0	NA	NA	NA	NA	NA	NA	NA	T	G
chr12:40240543	C	T	45857	0	NA	NA	NA	NA	NA	NA	NA	T	C
chr12:40240594	G	C	45857	9.81E-05	1.87183	0.953356	3.67516	#ERROR!	1.96341	1.02417	0.055229	C	G
chr12:40243556	A	T	45857	0	NA	NA	NA	NA	NA	NA	NA	T	A
chr12:40249843	C	G	45857	0.00041433	1.7769	4.20374	0.751084	#ERROR!	0.422694	0.487733	0.386133	G	C
chr12:40251273	G	A	45857	0.00016355	1.51736	1.8034	1.27669	#ERROR!	0.841389	0.744652	0.258515	A	G
chr12:40251346	C	T	45857	0.00029439	0.915282	2.6573	0.31526	#ERROR!	0.34444	0.613451	0.57447	T	C
chr12:40251369	G	A	45857	1.09E-05	-0.0914455	0.0830757	0.100658	-	-1.10075	3.46947	0.751041	A	G
chr12:40251480	G	A	45857	0	NA	NA	NA	NA	NA	NA	NA	A	G
chr12:40257283	C	T	45857	0	NA	NA	NA	NA	NA	NA	NA	T	C
chr12:40263806	A	G	45857	0	NA	NA	NA	NA	NA	NA	NA	G	A
chr12:40263861	A	G	45857	0.00014175	0.322252	1.44643	0.0717949	#ERROR!	0.222791	0.831478	0.788741	G	A
chr12:40274666	A	T	45857	0	NA	NA	NA	NA	NA	NA	NA	T	A
chr12:40278179	A	G	45857	9.81E-05	-0.97775	1.09925	0.869676	-	-0.889467	0.953786	0.351045	G	A
chr12:40283944	G	T	45857	0	NA	NA	NA	NA	NA	NA	NA	T	G
chr12:40284011	G	T	45857	0.00086137	-5.50579	9.40169	3.22428	-	-0.585616	0.326135	0.0725538	T	G
chr12:40284061	A	G	45857	0	NA	NA	NA	NA	NA	NA	NA	G	A
chr12:40287443	T	C	45857	0	NA	NA	NA	NA	NA	NA	NA	C	T
chr12:40293552	A	C	45857	0	NA	NA	NA	NA	NA	NA	NA	C	A
chr12:40293624	G	C	45857	9.81E-05	-1.23236	1.01298	1.49924	-	-1.21656	0.993571	0.220788	C	G
chr12:40293626	G	A	45857	1.09E-05	-0.224759	0.174213	0.28997	-	-1.29014	2.39585	0.59024	A	G
chr12:40294866	G	T	45857	2.18E-05	-0.143171	0.13147	0.155913	-	-1.089	2.75796	0.692948	T	G
chr12:40294909	C	T	45857	0	NA	NA	NA	NA	NA	NA	NA	T	C
chr12:40298433	C	G	45857	1.09E-05	-0.0563089	0.0531321	0.0596757	-	-1.05979	4.33832	0.807009	G	C
chr12:40299212	G	A	45857	3.27E-05	-0.0350358	0.307126	0.00399676	-	-0.114077	1.80444	0.949591	A	G
chr12:40302866	A	G	45857	1.09E-05	-0.227616	0.175806	0.294696	-	-1.2947	2.38497	0.587227	G	A
chr12:40304000	G	A	45857	0.00011994	1.71485	1.04923	2.80272	#ERROR!	1.63438	0.976256	0.0941046	A	G

chr12:40304040	G	C	45857	0.00050156	-1.44705	5.51744	0.379515	-	-0.262268	0.425727	0.537863	C	G
chr12:40305946	T	A	45857	3.27E-05	0.517579	0.450059	0.595229	#ERROR!	1.15003	1.49061	0.440404	A	T
chr12:40308481	G	A	45857	0.00068692	1.88962	7.26062	0.491787	#ERROR!	0.260257	0.371119	0.483131	A	G
chr12:40308507	C	T	45857	0	NA	NA	NA	NA	NA	NA	NA	T	C
chr12:40310435	G	A	45857	1.09E-05	-0.0440817	0.0421354	0.0461178	-	-1.04619	4.87165	0.829962	A	G
chr12:40310515	A	G	45857	1.09E-05	-0.108545	0.0967446	0.121784	-	-1.12197	3.21504	0.727108	G	A
chr12:40310561	G	A	45857	0	NA	NA	NA	NA	NA	NA	NA	A	G
chr12:40320042	C	T	45857	0	NA	NA	NA	NA	NA	NA	NA	T	C
chr12:40320103	A	G	45857	0.00021807	-1.40315	1.9303	1.01995	-	-0.726904	0.719759	0.31253	G	A
chr12:40322037	C	T	45857	0	NA	NA	NA	NA	NA	NA	NA	T	C
chr12:40322038	G	A	45857	2.18E-05	-0.265413	0.229451	0.307011	-	-1.15673	2.08764	0.57952	A	G
chr12:40323256	T	C	45857	0.00025078	-0.735752	2.37292	0.228128	-	-0.310061	0.649169	0.632915	C	T
chr12:40335031	G	A	45857	0.00022897	-1.03595	2.49556	0.430045	-	-0.41512	0.633018	0.511967	A	G
chr12:40340380	T	C	45857	0	NA	NA	NA	NA	NA	NA	NA	C	T
chr12:40340400	G	A	45857	0.00045795	9.4569	5.24759	17.0427	#ERROR!	1.80214	0.436536	3.65E-05	A	G
chr12:40351579	C	T	45857	0	NA	NA	NA	NA	NA	NA	NA	T	C
chr12:40351585	G	A	45857	9.81E-05	-1.25538	1.07864	1.46108	-	-1.16386	0.962858	0.226758	A	G
chr12:40351680	G	C	45857	0	NA	NA	NA	NA	NA	NA	NA	C	G
chr12:40351723	A	G	45857	0.00018536	0.0792389	1.6712	0.00375705	#ERROR!	0.0474142	0.773544	0.951124	G	A
chr12:40354486	A	T	45857	5.45E-05	-0.627544	0.529598	0.743605	-	-1.18494	1.37413	0.388508	T	A
chr12:40359345	C	T	45857	3.27E-05	1.06986	0.361274	3.16822	#ERROR!	2.96135	1.66373	0.0750843	T	C
chr12:40363440	C	T	45857	0.00020717	-1.02262	2.36106	0.442916	-	-0.433119	0.650798	0.505718	T	C
chr12:40363526	G	A	45857	0	NA	NA	NA	NA	NA	NA	NA	A	G
chr12:40363541	G	A	45857	6.54E-05	-0.881355	0.733068	1.05964	-	-1.20228	1.16796	0.303298	A	G
chr12:40364843	G	A	45857	0	NA	NA	NA	NA	NA	NA	NA	A	G
chr12:40364975	C	A	45857	2.18E-05	-0.24196	0.212569	0.275415	-	-1.13827	2.16895	0.599722	A	C
chr12:40367012	T	A	45857	0	NA	NA	NA	NA	NA	NA	NA	A	T
chr12:40367045	G	A	45857	0.00021807	0.297855	1.82928	0.0484986	#ERROR!	0.162826	0.739367	0.825697	A	G
chr14:22873373	C	T	45857	4.36E-05	1.26161	0.57975	2.74544	#ERROR!	2.17613	1.31335	0.0975323	T	C
chr14:22875400	G	A	45857	2.18E-05	0.61142	0.312007	1.19816	#ERROR!	1.95964	1.79027	0.27369	A	G
chr14:22875562	C	T	45857	0	NA	NA	NA	NA	NA	NA	NA	T	C
chr14:22875591	T	C	45857	0	NA	NA	NA	NA	NA	NA	NA	C	T
chr14:22875639	C	T	45857	0.00010904	2.41091	1.19558	4.86165	#ERROR!	2.01652	0.914557	0.02746	T	C
chr14:22875648	G	A	45857	0.00029439	0.243232	3.07876	0.0192161	#ERROR!	0.0790031	0.569917	0.889749	A	G
chr14:22875661	C	T	45857	0	NA	NA	NA	NA	NA	NA	NA	T	C
chr14:22875844	A	G	45857	1.09E-05	-0.0871288	0.0795267	0.0954577	-	-1.09559	3.54604	0.75735	G	A
chr14:22875924	G	A	45857	0.00057788	0.2043	5.63707	0.00740431	#ERROR!	0.0362423	0.421185	0.931428	A	G
chr14:22876078	C	G	45857	0	NA	NA	NA	NA	NA	NA	NA	G	C
chr14:22876101	A	G	45857	0	NA	NA	NA	NA	NA	NA	NA	G	A
chr14:22876113	G	A	45857	0.00017446	1.80362	2.16487	1.50265	#ERROR!	0.833131	0.679647	0.220263	A	G
chr14:22876722	G	C	45857	0.00026168	0.841237	2.97209	0.238108	#ERROR!	0.283045	0.580055	0.625576	C	G
chr14:22876765	G	T	45857	2.18E-05	0.907555	0.0879039	9.36996	#ERROR!	10.3244	3.37284	0.00220571	T	G
chr14:22876816	G	A	45857	1.09E-05	-0.094235	0.0853489	0.104046	-	-1.10411	3.42295	0.747027	A	G
chr14:22877045	C	T	45857	1.09E-05	-0.112283	0.0996565	0.12651	-	-1.1267	3.16772	0.722078	T	C
chr14:22877070	G	A	45857	0.00904987	-0.063269	94.8103	4.22E-05	-	-0.0006673	0.1027	0.994816	A	G

chr14:22877106	G	A		45857	2.18E-05	-0.400505	0.319934	0.501366	-	-1.25184	1.76795	0.4789	A	G
chr14:22877108	G	A		45857	0.00015265	2.39125	1.70713	3.34952	#ERROR!	1.40074	0.765361	0.0672247	A	G
chr14:22877128	G	T		45857	0	NA	NA	NA	NA	NA	NA	NA	T	G
chr14:22877184	G	A		45857	2.18E-05	-0.205421	0.179747	0.234763	-	-1.14283	2.35868	0.628015	A	G
chr14:22877259	C	G		45857	0	NA	NA	NA	NA	NA	NA	NA	G	C
chr14:22877421	A	C		45857	0.00066511	-3.91034	6.09657	2.5081	-	-0.641401	0.405002	0.113263	C	A
chr15:50586433	G	A		45857	0.106091	22.1764	977.684	0.503018	#ERROR!	0.0226826	0.0319816	0.478177	A	G
chr15:61854543	T	C		45857	0.00514643	-7.77159	53.9738	1.11902	-	-0.143988	0.136116	0.29013	C	T
chr15:61854888	C	A		45857	0.00176636	-0.168233	17.0968	0.00165542	-	-0.00984	0.241848	0.967546	A	C
chr15:61854947	C	T		45857	0	NA	NA	NA	NA	NA	NA	NA	T	C
chr15:61856407	C	T		45857	0.00053427	-3.19121	5.92459	1.71891	-	-0.538638	0.410838	0.189834	T	C
chr15:61856408	G	A		45857	0.00086137	3.90112	8.80903	1.72763	#ERROR!	0.442855	0.336927	0.188714	A	G
chr15:61868675	C	T		45857	2.18E-05	-0.318744	0.267163	0.380284	-	-1.19307	1.93469	0.537452	T	C
chr15:61868702	C	T		45857	0.00091589	3.1944	8.92392	1.14346	#ERROR!	0.357959	0.334751	0.284922	T	C
chr15:61880845	G	A		45857	2.18E-05	0.419976	0.365404	0.482698	#ERROR!	1.14935	1.6543	0.487203	A	G
chr15:61880871	G	A		45857	0.00017446	0.750181	1.82767	0.307917	#ERROR!	0.410457	0.739692	0.578961	A	G
chr15:61880907	C	T		45857	0	NA	NA	NA	NA	NA	NA	NA	T	C
chr15:61882609	A	G		45857	0.00041433	-3.7379	4.58172	3.04948	-	-0.815828	0.467181	0.0807629	G	A
chr15:61890334	G	A		45857	1.09E-05	-0.212192	0.167134	0.269399	-	-1.2696	2.44606	0.603735	A	G
chr15:61890400	C	T		45857	6.54E-05	0.214461	0.674023	0.0682375	#ERROR!	0.318181	1.21804	0.79392	T	C
chr15:61907289	T	C		45857	0	NA	NA	NA	NA	NA	NA	NA	C	T
chr15:61910215	G	A		45857	0	NA	NA	NA	NA	NA	NA	NA	A	G
chr15:61911844	G	A		45857	0.00295484	8.57636	29.0056	2.53585	#ERROR!	0.295679	0.185677	0.111287	A	G
chr15:61915712	A	G		45857	0.00991125	4.82908	99.3809	0.234652	#ERROR!	0.0485916	0.100311	0.628095	G	A
chr15:61915725	G	A		45857	3.27E-05	-0.301289	0.261873	0.346638	-	-1.15051	1.95413	0.556023	A	G
chr15:61915865	G	C		45857	0	NA	NA	NA	NA	NA	NA	NA	C	G
chr15:61916007	G	C		45857	0.00010904	-1.5234	1.21965	1.90281	-	-1.24905	0.905489	0.167764	C	G
chr15:61917482	T	C		45857	0	NA	NA	NA	NA	NA	NA	NA	C	T
chr15:61917535	C	T		45857	0	NA	NA	NA	NA	NA	NA	NA	T	C
chr15:61918208	T	C		45857	0.00033801	-3.51071	3.65558	3.37158	-	-0.960371	0.523024	0.06633	C	T
chr15:61920099	C	T		45857	0.00617136	-7.10481	63.9192	0.789721	-	-0.111153	0.125079	0.374184	T	C
chr15:61920268	C	T		45857	0.00240966	-0.0527163	24.0176	0.00011571	-	-0.0021949	0.204049	0.991418	T	C
chr15:61920281	T	A		45857	0	NA	NA	NA	NA	NA	NA	NA	A	T
chr15:61920571	T	C		45857	0.00292213	5.59768	32.3527	0.968515	#ERROR!	0.173021	0.17581	0.325051	C	T
chr15:61922408	C	T		45857	0.0750376	-7.86602	720.759	0.085846	-	-0.0109135	0.0372482	0.769526	T	C
chr15:61922527	G	A		45857	0.00018536	-1.07545	1.7036	0.678913	-	-0.631281	0.766153	0.409961	A	G
chr15:61922557	A	G		45857	0.00124299	-7.40601	12.762	4.29785	-	-0.580319	0.279925	0.0381605	G	A
chr15:61922680	G	T		45857	8.72E-05	-0.895067	0.833716	0.960933	-	-1.07359	1.09519	0.326952	T	G
chr15:61927138	G	A		45857	0.0141091	-3.75631	138.534	0.101851	-	-0.0271147	0.0849614	0.749619	A	G
chr15:61929540	G	A		45857	0	NA	NA	NA	NA	NA	NA	NA	A	G
chr15:61929546	T	A		45857	0	NA	NA	NA	NA	NA	NA	NA	A	T
chr15:61929659	G	C		45857	0	NA	NA	NA	NA	NA	NA	NA	C	G
chr15:61931104	C	G		45857	0.0218178	-1.49577	218.371	0.0102456	-	-0.0068497	0.067671	0.919375	G	C
chr15:61936649	C	G		45857	2.18E-05	0.715253	0.244078	2.096	#ERROR!	2.93043	2.02412	0.147685	G	C
chr15:61936716	A	C		45857	0	NA	NA	NA	NA	NA	NA	NA	C	A

chr15:61940651	A	G	45857	0	NA	NA	NA	NA	NA	NA	NA	G	A
chr15:61940726	G	A	45857	1.09E-05	-0.042131	0.0403528	0.0439876	-	-1.04407	4.97809	0.833877	A	G
chr15:61941806	T	C	45857	0	NA	NA	NA	NA	NA	NA	NA	C	T
chr15:61945803	G	A	45857	0.00304207	-2.79954	30.9839	0.252952	-	-0.0903546	0.179652	0.615004	A	G
chr15:61950998	T	C	45857	0.0741108	-10.3839	713.593	0.151102	-	-0.0145516	0.0374347	0.697484	C	T
chr15:61951827	T	C	45857	0	NA	NA	NA	NA	NA	NA	NA	C	T
chr15:61954471	T	G	45857	0.00089408	-0.573265	9.01447	0.0364562	-	-0.0635939	0.333066	0.848577	G	T
chr15:61958653	C	A	45857	0	NA	NA	NA	NA	NA	NA	NA	A	C
chr15:61961592	T	C	45857	0.0766077	-10.6469	734.202	0.154395	-	-0.0145014	0.0369056	0.69437	C	T
chr15:61961728	T	C	45857	5.45E-05	-0.0403461	0.814696	0.00199805	-	-0.0495229	1.1079	0.964347	C	T
chr15:61961754	G	A	45857	0.00025078	-1.38511	2.75931	0.695295	-	-0.501978	0.602005	0.404369	A	G
chr15:61961776	T	G	45857	0	NA	NA	NA	NA	NA	NA	NA	G	T
chr15:61962390	T	G	45857	2.18E-05	0.603552	0.31738	1.14776	#ERROR!	1.90167	1.77505	0.284019	G	T
chr15:61963904	C	T	45857	0.00050156	-1.82543	4.86468	0.684974	-	-0.375241	0.453391	0.407879	T	C
chr15:61966088	C	T	45857	0	NA	NA	NA	NA	NA	NA	NA	T	C
chr15:61969413	T	C	45857	0.00423054	-11.1844	43.2721	2.89078	-	-0.258466	0.152018	0.089088	C	T
chr15:61977090	T	G	45857	0	NA	NA	NA	NA	NA	NA	NA	G	T
chr15:61977193	G	T	45857	1.09E-05	-0.221502	0.172421	0.284554	-	-1.28466	2.40827	0.593731	T	G
chr15:61978646	T	C	45857	2.18E-05	-0.0745901	0.0717208	0.0775742	-	-1.04001	3.73403	0.780612	C	T
chr15:61983868	G	T	45857	0	NA	NA	NA	NA	NA	NA	NA	T	G
chr15:61983908	T	A	45857	0	NA	NA	NA	NA	NA	NA	NA	A	T
chr15:61983936	T	C	45857	0	NA	NA	NA	NA	NA	NA	NA	C	T
chr15:61984004	G	A	45857	1.09E-05	-0.0905012	0.0822989	0.0995209	-	-1.09966	3.4858	0.752405	A	G
chr15:61984885	A	T	45857	0.00037072	1.85328	3.66799	0.936385	#ERROR!	0.505258	0.522139	0.333209	T	A
chr15:61984953	G	A	45857	5.45E-05	-0.579171	0.496852	0.675129	-	-1.16568	1.41869	0.411269	A	G
chr15:61984980	G	T	45857	0	NA	NA	NA	NA	NA	NA	NA	T	G
chr15:61991076	G	C	45857	0	NA	NA	NA	NA	NA	NA	NA	C	G
chr15:61991096	T	C	45857	0.00145016	-3.02971	14.4592	0.634828	-	-0.209535	0.262983	0.42559	C	T
chr15:62007466	T	C	45857	0	NA	NA	NA	NA	NA	NA	NA	C	T
chr15:62008659	G	A	45857	0	NA	NA	NA	NA	NA	NA	NA	A	G
chr15:62008667	T	C	45857	0	NA	NA	NA	NA	NA	NA	NA	C	T
chr15:62008671	T	C	45857	0.00074144	2.08132	7.7767	0.557037	#ERROR!	0.267636	0.358593	0.455457	C	T
chr15:62008730	A	G	45857	0.00059969	-3.3115	7.43044	1.47583	-	-0.445667	0.366854	0.224429	G	A
chr15:62010533	G	A	45857	1.09E-05	-0.173348	0.143284	0.209721	-	-1.20982	2.64181	0.646986	A	G
chr15:62010558	G	C	45857	1.09E-05	-0.184478	0.150364	0.226333	-	-1.22688	2.57886	0.634257	C	G
chr15:62012166	T	C	45857	3.27E-05	0.365124	0.450704	0.295794	#ERROR!	0.810119	1.48955	0.586531	C	T
chr15:62013055	G	A	45857	0	NA	NA	NA	NA	NA	NA	NA	A	G
chr15:62013943	A	G	45857	0	NA	NA	NA	NA	NA	NA	NA	G	A
chr15:62023783	T	C	45857	0.00078505	-1.62507	7.14579	0.369565	-	-0.227416	0.374089	0.543241	C	T
chr15:62023788	C	T	45857	4.36E-05	-0.0221602	0.711648	0.00069005	-	-0.0311392	1.18541	0.979043	T	C
chr15:62023837	G	A	45857	6.54E-05	0.00992975	0.800321	0.0001232	#ERROR!	0.0124072	1.11781	0.991144	A	G
chr15:62034998	G	A	45857	0.00019626	-1.14887	2.06815	0.638198	-	-0.555503	0.695358	0.424364	A	G
chr15:62035002	C	A	45857	0	NA	NA	NA	NA	NA	NA	NA	A	C
chr15:62044253	T	A	45857	0	NA	NA	NA	NA	NA	NA	NA	A	T
chr15:62044256	C	T	45857	7.63E-05	-0.329097	0.712495	0.152008	-	-0.461894	1.1847	0.696624	T	C

chr15:89316763	C	A	45857	0.0785594	-6.89384	755.697	0.0628891	-	-0.0091225	0.0363769	0.801987	A	C
chr15:89317460	G	A	45857	5.45E-05	-0.592115	0.495038	0.708228	-	-1.1961	1.42128	0.400033	A	G
chr15:89318553	T	C	45857	0	NA	NA	NA	NA	NA	NA	NA	C	T
chr15:89318581	G	A	45857	1.09E-05	-0.0530017	0.050189	0.055972	-	-1.05604	4.46371	0.81298	A	G
chr15:89318587	G	A	45857	0.00016355	-0.783995	1.45237	0.423203	-	-0.539803	0.829776	0.515343	A	G
chr15:89318595	T	C	45857	0.0403973	17.6718	403.557	0.773851	#ERROR!	0.0437901	0.0497792	0.379028	C	T
chr15:89318598	C	T	45857	0	NA	NA	NA	NA	NA	NA	NA	T	C
chr15:89318599	G	A	45857	1.09E-05	-0.0744259	0.0688796	0.0804187	-	-1.08052	3.81026	0.776731	A	G
chr15:89318617	C	T	45857	1.09E-05	0.76383	0.180345	3.23511	#ERROR!	4.23538	2.35477	0.0720753	T	C
chr15:89318641	G	A	45857	1.09E-05	-0.0742153	0.0686995	0.080174	-	-1.08029	3.81525	0.777062	A	G
chr15:89318677	T	C	45857	0.00011994	-1.25296	1.17402	1.3372	-	-1.06724	0.922918	0.247528	C	T
chr15:89318736	C	T	45857	0	NA	NA	NA	NA	NA	NA	NA	T	C
chr15:89318737	G	A	45857	1.09E-05	0.615432	0.236602	1.60082	#ERROR!	2.60113	2.05585	0.205787	A	G
chr15:89318962	C	T	45857	0	NA	NA	NA	NA	NA	NA	NA	T	C
chr15:89318986	G	A	45857	1.09E-05	-0.263089	0.193779	0.357189	-	-1.35767	2.27168	0.550072	A	G
chr15:89318989	G	C	45857	0	NA	NA	NA	NA	NA	NA	NA	C	G
chr15:89319053	C	G	45857	1.09E-05	-0.168319	0.13997	0.202411	-	-1.20254	2.6729	0.652782	G	C
chr15:89319065	G	A	45857	3.27E-05	-0.524627	0.429731	0.64048	-	-1.22083	1.52546	0.423537	A	G
chr15:89319073	A	G	45857	0.00095951	-4.87977	9.9397	2.39566	-	-0.490937	0.317185	0.121673	G	A
chr15:89319234	G	A	45857	7.63E-05	0.00992905	0.673548	0.00014637	#ERROR!	0.0147414	1.21847	0.990347	A	G
chr15:89320832	C	T	45857	0	NA	NA	NA	NA	NA	NA	NA	T	C
chr15:89320850	A	C	45857	8.72E-05	-0.907478	1.01972	0.807591	-	-0.889929	0.990284	0.368833	C	A
chr15:89320856	C	T	45857	0	NA	NA	NA	NA	NA	NA	NA	T	C
chr15:89320857	G	A	45857	0	NA	NA	NA	NA	NA	NA	NA	A	G
chr15:89320917	C	T	45857	0	NA	NA	NA	NA	NA	NA	NA	T	C
chr15:89320953	G	A	45857	0	NA	NA	NA	NA	NA	NA	NA	A	G
chr15:89321217	G	A	45857	0.00015265	-1.42693	1.40765	1.44647	-	-1.01369	0.842854	0.229096	A	G
chr15:89321223	T	C	45857	0	NA	NA	NA	NA	NA	NA	NA	C	T
chr15:89321242	C	A	45857	0	NA	NA	NA	NA	NA	NA	NA	A	C
chr15:89321780	G	A	45857	0.00011994	-0.70458	1.35856	0.365412	-	-0.518624	0.857948	0.545516	A	G
chr15:89321792	C	T	45857	0.00044704	2.46656	4.72954	1.28637	#ERROR!	0.521522	0.459823	0.256718	T	C
chr15:89321842	T	C	45857	0.0103692	0.330502	106.092	0.00102959	#ERROR!	0.00311523	0.0970863	0.974402	C	T
chr15:89322748	C	T	45857	0	NA	NA	NA	NA	NA	NA	NA	T	C
chr15:89322799	C	T	45857	0	NA	NA	NA	NA	NA	NA	NA	T	C
chr15:89322800	G	A	45857	3.27E-05	0.00623204	0.377495	0.00010288	#ERROR!	0.0165089	1.62759	0.991907	A	G
chr15:89323423	A	G	45857	0	NA	NA	NA	NA	NA	NA	NA	G	A
chr15:89323426	C	G	45857	0.00053427	0.610122	5.27542	0.0705628	#ERROR!	0.115654	0.435383	0.790519	G	C
chr15:89323445	C	T	45857	0	NA	NA	NA	NA	NA	NA	NA	T	C
chr15:89323451	T	C	45857	3.27E-05	-0.266554	0.241737	0.293919	-	-1.10266	2.0339	0.58772	C	T
chr15:89323460	C	G	45857	0.00203895	-1.0033	20.3278	0.0495184	-	-0.0493557	0.221796	0.823903	G	C
chr15:89323462	T	C	45857	0.00037072	-0.104225	4.19371	0.00259026	-	-0.0248527	0.488316	0.95941	C	T
chr15:89323504	C	T	45857	0	NA	NA	NA	NA	NA	NA	NA	T	C
chr15:89324193	C	T	45857	0.00017446	1.98405	1.66151	2.36919	#ERROR!	1.19412	0.775797	0.123751	T	C
chr15:89325456	G	C	45857	1.09E-05	-0.221114	0.172139	0.284023	-	-1.28451	2.41024	0.594076	C	G
chr15:89325501	T	G	45857	0	NA	NA	NA	NA	NA	NA	NA	G	T

chr15:89325520	G	A	45857	0	NA	NA	NA	NA	NA	NA	NA	A	G
chr15:89325562	G	A	45857	0	NA	NA	NA	NA	NA	NA	NA	A	G
chr15:89325591	A	G	45857	4.36E-05	-0.430921	0.379003	0.489951	-	-1.13699	1.62435	0.483949	G	A
chr15:89325639	G	A	45857	0.00227882	1.37426	23.193	0.0814286	#ERROR!	0.0592529	0.207645	0.775371	A	G
chr15:89326688	G	A	45857	3.27E-05	-0.525371	0.400509	0.689159	-	-1.31176	1.58013	0.406451	A	G
chr15:89326947	C	A	45857	0.00795953	-6.56037	81.183	0.530141	-	-0.0808097	0.110986	0.466547	A	C
chr15:89327004	T	G	45857	0.0003271	0.840658	3.39055	0.208434	#ERROR!	0.247941	0.543081	0.647998	G	T
chr15:89327006	C	G	45857	3.27E-05	-0.413024	0.352684	0.483688	-	-1.17109	1.68386	0.486756	G	C
chr15:89327198	T	C	45857	0.00080686	3.60398	8.10551	1.60245	#ERROR!	0.444633	0.351245	0.205557	C	T
chr15:89327201	C	T	45857	0.00161371	0.590726	15.3609	0.0227173	#ERROR!	0.0384565	0.255148	0.880195	T	C
chr15:89327300	A	G	45857	1.09E-05	-0.210326	0.166039	0.266426	-	-1.26673	2.45411	0.60574	G	A
chr15:89328532	G	C	45857	0.00243147	-2.61682	24.6055	0.278301	-	-0.106351	0.201597	0.597817	C	G
chr15:89328699	G	A	45857	0	NA	NA	NA	NA	NA	NA	NA	A	G
chr15:89328795	T	C	45857	0	NA	NA	NA	NA	NA	NA	NA	C	T
chr15:89328996	G	A	45857	0.00059969	0.423724	7.41805	0.0242034	#ERROR!	0.0571207	0.36716	0.876368	A	G
chr15:89330081	C	G	45857	3.27E-05	0.242628	0.431808	0.136329	#ERROR!	0.561887	1.52179	0.711958	G	C
chr15:89330106	T	A	45857	0.00105764	3.01679	10.9098	0.834206	#ERROR!	0.276521	0.302755	0.361059	A	T
chr15:89330133	C	G	45857	0.00435048	14.7178	45.2779	4.78407	#ERROR!	0.325054	0.148613	0.0287242	G	C
chr15:89330184	G	A	45857	0.00237695	0.93266	24.0199	0.0362138	#ERROR!	0.0388286	0.204039	0.849075	A	G
chr15:89330257	G	A	45857	1.09E-05	-0.173954	0.143646	0.210655	-	-1.21099	2.63847	0.646254	A	G
chr15:89330258	C	G	45857	0.00068692	0.433291	7.29635	0.0257308	#ERROR!	0.0593846	0.370209	0.87256	G	C
chr15:89333177	C	T	45857	0	NA	NA	NA	NA	NA	NA	NA	T	C
chr15:89333327	G	A	45857	1.09E-05	-0.149687	0.127259	0.176068	-	-1.17624	2.80321	0.674774	A	G
chr15:89333347	G	C	45857	0	NA	NA	NA	NA	NA	NA	NA	C	G
chr15:89333357	T	C	45857	9.81E-05	0.798959	0.965574	0.661094	#ERROR!	0.827445	1.01767	0.416174	C	T
chr15:89333364	A	G	45857	0.0003162	2.45823	3.41014	1.77204	#ERROR!	0.72086	0.541519	0.183129	G	A
chr15:89333427	G	A	45857	0	NA	NA	NA	NA	NA	NA	NA	A	G
chr15:89333627	T	C	45857	0.00292213	-7.91214	30.5976	2.04597	-	-0.258587	0.180782	0.152609	C	T
chr15:89333723	C	T	45857	0.00070873	-1.37365	7.64427	0.246841	-	-0.179697	0.361686	0.619308	T	C
chr16:46661807	T	G	45857	0	NA	NA	NA	NA	NA	NA	NA	G	T
chr16:46662405	A	C	45857	1.09E-05	-0.0692021	0.0644062	0.0743551	-	-1.07446	3.94036	0.785098	C	A
chr16:46674332	T	C	45857	0.00013084	-1.30026	1.09724	1.54084	-	-1.18503	0.954661	0.214493	C	T
chr16:46674380	C	T	45857	0	NA	NA	NA	NA	NA	NA	NA	T	C
chr16:46682118	G	A	45857	0	NA	NA	NA	NA	NA	NA	NA	A	G
chr16:46683521	A	T	45857	0.00026168	3.5838	3.22404	3.98371	#ERROR!	1.11159	0.556929	0.0459424	T	A
chr17:46024061	C	T	45857	1.09E-05	-0.113904	0.100921	0.128558	-	-1.12865	3.14782	0.719932	T	C
chr20:5069196	G	T	45857	0.00288942	-3.61908	29.1332	0.449581	-	-0.124225	0.18527	0.502534	T	G
chr20:5069288	C	T	45857	0.00139564	5.1446	13.86	1.90959	#ERROR!	0.371183	0.268608	0.167009	T	C
chr20:5069330	C	T	45857	0	NA	NA	NA	NA	NA	NA	NA	T	C
chr20:5100832	G	A	45857	0.00196262	-3.1823	20.3104	0.498611	-	-0.156683	0.221891	0.480111	A	G
chr20:5106224	T	C	45857	0.00099222	-5.91409	10.4193	3.35689	-	-0.567609	0.3098	0.0669242	C	T
chr20:5109429	A	G	45857	0.00150468	-2.66307	15.304	0.463405	-	-0.174011	0.255622	0.496037	G	A
chr20:5109435	C	T	45857	0.00016355	1.58588	1.82686	1.3767	#ERROR!	0.868095	0.739857	0.240664	T	C
chr21:32631012	C	A	45857	0	NA	NA	NA	NA	NA	NA	NA	A	C
chr21:32631144	C	T	45857	0	NA	NA	NA	NA	NA	NA	NA	T	C

chr21:32631764	G	A	45857	0	NA	NA	NA	NA	NA	NA	NA	A	G
chr21:32639773	G	A	45857	1.09E-05	-0.162395	0.135967	0.193959	-	-1.19437	2.71196	0.659641	A	G
chr21:32641904	C	G	45857	0	NA	NA	NA	NA	NA	NA	NA	G	C
chr21:32643457	C	T	45857	0	NA	NA	NA	NA	NA	NA	NA	T	C
chr21:32656885	A	G	45857	1.09E-05	-0.069985	0.0650822	0.0752573	-	-1.07533	3.91985	0.783831	G	A
chr21:32657057	G	A	45857	0	NA	NA	NA	NA	NA	NA	NA	A	G
chr21:32664963	C	A	45857	0.00087228	-3.19254	10.2501	0.994356	-	-0.311463	0.312345	0.31868	A	C
chr21:32665968	A	G	45857	0.00154829	-8.67834	16.1594	4.66066	-	-0.537045	0.248764	0.0308613	G	A
chr21:32666479	C	T	45857	4.36E-05	-0.435578	0.368616	0.514704	-	-1.18166	1.64707	0.47311	T	C
chr21:32673463	C	T	45857	0.0003053	-0.88885	3.02053	0.261562	-	-0.29427	0.575385	0.609049	T	C
chr21:32673511	T	C	45857	6.54E-05	-1.17304	0.920798	1.49437	-	-1.27393	1.04212	0.22154	C	T
chr21:32688306	C	T	45857	0	NA	NA	NA	NA	NA	NA	NA	T	C
chr21:32688348	C	T	45857	2.18E-05	-0.3893	0.313061	0.484105	-	-1.24353	1.78725	0.486569	T	C
chr21:32688349	G	A	45857	0	NA	NA	NA	NA	NA	NA	NA	A	G
chr21:32701973	G	A	45857	1.09E-05	-0.26619	0.1953	0.36281	-	-1.36298	2.26281	0.54695	A	G
chr21:32726855	T	C	45857	0	NA	NA	NA	NA	NA	NA	NA	C	T
chr21:32727961	C	T	45857	0	NA	NA	NA	NA	NA	NA	NA	T	C
chr22:32475067	C	T	45857	1.09E-05	0.932553	0.0628889	13.8284	#ERROR!	14.8286	3.98761	0.00020028	T	C
chr22:32475369	G	A	45857	0	NA	NA	NA	NA	NA	NA	NA	A	G
chr22:32475378	G	A	45857	0.0988399	4.48871	925.078	0.0217803	#ERROR!	0.00485224	0.0328784	0.882673	A	G
chr22:32479132	G	C	45857	4.36E-05	0.534478	0.401198	0.712034	#ERROR!	1.3322	1.57878	0.398769	C	G
chr22:32479135	T	G	45857	0.0003053	2.37381	2.96012	1.90363	#ERROR!	0.80193	0.581226	0.167673	G	T
chr22:32483895	A	T	45857	0	NA	NA	NA	NA	NA	NA	NA	T	A
chr22:32484066	A	G	45857	3.27E-05	0.492051	0.357179	0.67785	#ERROR!	1.3776	1.67324	0.410328	G	A
chr22:32487760	A	G	45857	0	NA	NA	NA	NA	NA	NA	NA	G	A
chr22:32493191	G	A	45857	0.00014175	0.228293	1.43318	0.036365	#ERROR!	0.159291	0.835312	0.848764	A	G
chr22:32498414	G	A	45857	0.00099222	3.19556	10.9458	0.932924	#ERROR!	0.291944	0.302257	0.334104	A	G
chr22:32498453	C	T	45857	1.09E-05	-0.207461	0.164386	0.261824	-	-1.26204	2.46643	0.60887	T	C
chr22:32498466	A	G	45857	2.18E-05	-0.404477	0.312579	0.523393	-	-1.294	1.78863	0.469398	G	A
chr22:32498513	C	T	45857	0.00011994	1.18409	0.892796	1.57043	#ERROR!	1.32627	1.05834	0.210145	T	C
chr22:38112165	G	C	45857	1.09E-05	-0.167432	0.139376	0.201135	-	-1.20129	2.67858	0.653806	C	G
chr22:38112534	C	G	45857	1.09E-05	0.890734	0.0973051	8.15381	#ERROR!	9.15403	3.20577	0.00429709	G	C
chr22:38112541	G	A	45857	0	NA	NA	NA	NA	NA	NA	NA	A	G
chr22:38112547	G	A	45857	1.09E-05	-0.0739991	0.0685108	0.079927	-	-1.08011	3.8205	0.777396	A	G
chr22:38112558	C	T	45857	2.18E-05	0.847666	0.140516	5.11355	#ERROR!	6.03251	2.6677	0.0237397	T	C
chr22:38112571	C	A	45857	5.45E-05	0.568279	0.546812	0.59059	#ERROR!	1.03926	1.35232	0.442191	A	C
chr22:38113561	G	A	45857	2.18E-05	-0.390415	0.313146	0.48675	-	-1.24675	1.78701	0.485381	A	G
chr22:38113621	C	T	45857	0.00050156	-0.187279	5.20698	0.00673586	-	-0.035967	0.438235	0.934589	T	C
chr22:38115658	G	A	45857	1.09E-05	-0.21677	0.169751	0.276812	-	-1.27698	2.42713	0.598799	A	G
chr22:38115667	G	A	45857	1.09E-05	-0.1482	0.126209	0.174022	-	-1.17424	2.81485	0.676562	A	G
chr22:38116105	C	T	45857	9.81E-05	0.126139	1.33661	0.0119041	#ERROR!	0.0943725	0.864962	0.913118	T	C
chr22:38116119	C	A	45857	4.36E-05	-0.678436	0.553222	0.831992	-	-1.22634	1.34447	0.361697	A	C
chr22:38116155	C	T	45857	0	NA	NA	NA	NA	NA	NA	NA	T	C
chr22:38120886	C	T	45857	0.00194082	-1.55102	20.7189	0.11611	-	-0.0748602	0.219693	0.733293	T	C
chr22:38120889	G	A	45857	2.18E-05	-0.140211	0.12954	0.15176	-	-1.08237	2.77842	0.696859	A	G

chr22:38123197	G	A	45857	0	NA	NA	NA	NA	NA	NA	NA	A	G
chr22:38126371	G	A	45857	0.00038162	-1.19715	3.79322	0.377821	-	-0.315602	0.513448	0.538772	A	G
chr22:38126374	C	T	45857	0.0003053	-0.37299	3.12266	0.0445523	-	-0.119446	0.565898	0.832829	T	C
chr22:38126390	T	C	45857	0.00046885	1.58016	4.31834	0.578206	#ERROR!	0.365917	0.481217	0.447016	C	T
chr22:38126417	G	A	45857	0.00019626	-2.52719	2.06548	3.09212	-	-1.22354	0.695809	0.0786725	A	G
chr22:38126451	T	C	45857	0	NA	NA	NA	NA	NA	NA	NA	C	T
chr22:38128349	G	A	45857	0.00037072	-3.75328	4.42742	3.18179	-	-0.847735	0.475253	0.0744633	A	G
chr22:38132850	G	A	45857	2.18E-05	0.135967	0.357602	0.0516971	#ERROR!	0.380218	1.67224	0.820136	A	G
chr22:38132881	C	T	45857	0.0122228	-4.72222	129.105	0.172723	-	-0.0365766	0.0880092	0.677703	T	C
chr22:38132917	C	T	45857	4.36E-05	0.7606	0.406222	1.42413	#ERROR!	1.87238	1.56898	0.232725	T	C
chr22:38132922	C	T	45857	1.09E-05	-0.14277	0.12237	0.166572	-	-1.16671	2.85866	0.683176	T	C
chr22:38132952	G	A	45857	0.00050156	3.40011	4.93359	2.34328	#ERROR!	0.689176	0.450213	0.125824	A	G
chr22:38133007	G	A	45857	0.00020717	-2.0763	2.2231	1.9392	-	-0.933969	0.670688	0.163755	A	G
chr22:38133010	C	T	45857	1.09E-05	-0.10761	0.0960201	0.1206	-	-1.12071	3.22715	0.728385	T	C
chr22:38135029	G	A	45857	7.63E-05	-0.126715	0.864666	0.0185699	-	-0.146548	1.07541	0.891607	A	G
chr22:38140006	G	A	45857	0.00021807	0.297151	1.91541	0.0460992	#ERROR!	0.155137	0.722552	0.829996	A	G
chr22:38140069	C	T	45857	8.72E-05	-1.2042	0.962493	1.50659	-	-1.25112	1.0193	0.21966	T	C
chr22:38143150	G	A	45857	0.00010904	-0.47265	1.19608	0.186776	-	-0.395167	0.914367	0.665614	A	G
chr22:38143219	C	G	45857	0.00016355	-0.887703	1.57024	0.501846	-	-0.565331	0.798027	0.47869	G	C
chr22:38145447	C	T	45857	0.00054517	-3.03686	5.35412	1.72251	-	-0.567201	0.432171	0.18937	T	C
chr22:38145597	G	T	45857	0	NA	NA	NA	NA	NA	NA	NA	T	G
chr22:38145625	C	T	45857	0	NA	NA	NA	NA	NA	NA	NA	T	C
chr22:38169318	G	A	45857	6.54E-05	0.55517	0.516652	0.59656	#ERROR!	1.07455	1.39124	0.439894	A	G
chr22:38169326	G	A	45857	0.00087228	-0.450606	9.37452	0.0216593	-	-0.0480671	0.326607	0.882997	A	G
chr22:38169336	C	T	45857	0.00050156	-0.104805	4.63432	0.00237018	-	-0.0226151	0.464523	0.961171	T	C

Supplementary Table 6: AMP-PD summary statistics

Location	REF	ALT	N_INFORMATIV	AF	U	V	STAT	DIRECTION	EFFECT	SE	PVALUE	minorAllele	majorAllele
chr1:7985019	G	A	4007	0.00074869	1.21194	1.23887	1.18561	#ERROR!	0.97827	0.898438	0.276217	A	G
chr1:16985990	C	T	4007	0.00074869	-1.08424	1.31297	0.895357	-	-0.825794	0.872717	0.34403	T	C
chr1:16986065	C	T	4007	0.00037435	0.0300306	0.648305	0.00139107	#ERROR!	0.0463217	1.24197	0.970248	T	C
chr1:16986091	G	A	4007	0.00411779	-1.46934	7.54414	0.286178	-	-0.194766	0.364079	0.59268	A	G
chr1:16986097	G	A	4007	0.0504118	-1.67883	84.7755	0.0332463	-	-0.0198033	0.108609	0.855319	A	G
chr1:16986101	T	A	4007	0.00549039	-1.41294	9.16515	0.217825	-	-0.154165	0.330317	0.640702	A	T
chr1:16986246	G	A	4007	0.00012478	0.604212	0.238637	1.52982	#ERROR!	2.53193	2.04706	0.216139	A	G
chr1:16986248	T	C	4007	0.484901	-18.5438	422.854	0.813223	-	-0.0438541	0.0486301	0.367169	C	T
chr1:16986291	C	T	4007	0.00024956	0.214196	0.448084	0.102391	#ERROR!	0.478026	1.49389	0.748979	T	C
chr1:16986335	G	A	4007	0.00037435	-0.923799	0.573829	1.48721	-	-1.60989	1.32011	0.22265	A	G
chr1:16986554	G	A	4007	0.00049913	-1.23018	0.831186	1.82072	-	-1.48003	1.09686	0.177228	A	G
chr1:16988161	T	A	4007	0.00124782	1.13831	2.13863	0.60588	#ERROR!	0.532263	0.683806	0.436343	A	T
chr1:16988226	C	T	4007	0.00024956	0.157612	0.468235	0.0530533	#ERROR!	0.336608	1.4614	0.817833	T	C
chr1:16990276	G	C	4007	0.00074869	-1.09628	1.31397	0.914647	-	-0.834322	0.872383	0.338885	C	G
chr1:16996298	G	C	4007	0.00124782	0.0152336	2.24439	0.0001034	#ERROR!	0.00678744	0.6675	0.991887	C	G
chr1:17000272	C	T	4007	0.0193412	-1.37974	31.6666	0.0601164	-	-0.0435708	0.177705	0.806312	T	C
chr1:17000494	G	A	4007	0.00099825	2.64014	1.84066	3.78685	#ERROR!	1.43434	0.737077	0.0516567	A	G
chr1:17005517	C	T	4007	0.00037435	0.0231533	0.587336	0.00091273	#ERROR!	0.0394209	1.30484	0.975898	T	C
chr1:20638080	C	T	4007	0.00049913	-0.298655	0.798691	0.111676	-	-0.373931	1.11895	0.738244	T	C
chr1:20644515	C	G	4007	0.00024956	0.51641	0.363623	0.733393	#ERROR!	1.42018	1.65834	0.391786	G	C
chr1:20644665	A	T	4007	0.00099825	0.0044328	1.80985	1.09E-05	#ERROR!	0.00244926	0.743325	0.997371	T	A
chr1:20645615	G	A	4007	0.00162216	0.91455	2.61367	0.320011	#ERROR!	0.34991	0.61855	0.571601	A	G
chr1:20648528	G	A	4007	0.00049913	0.132616	0.98113	0.0179252	#ERROR!	0.135166	1.00957	0.893494	A	G
chr1:20648612	G	A	4007	0.00099825	-0.221852	1.88186	0.026154	-	-0.11789	0.728965	0.871525	A	G
chr1:20649109	C	T	4007	0.00012478	-0.159637	0.133969	0.190223	-	-1.1916	2.73211	0.662731	T	C
chr1:65366050	A	T	4007	0.00024956	0.115132	0.486767	0.0272316	#ERROR!	0.236524	1.43331	0.868928	T	A
chr1:65385740	G	A	4007	0.00099825	1.22855	1.62995	0.926001	#ERROR!	0.753736	0.783273	0.335904	A	G
chr1:65386873	G	A	4007	0.00012478	0.547637	0.247407	1.2122	#ERROR!	2.21351	2.01045	0.270897	A	G
chr1:65386886	T	C	4007	0.00024956	1.04266	0.497085	2.18704	#ERROR!	2.09755	1.41835	0.139177	C	T
chr1:65392454	T	A	4007	0.00087347	-0.894107	1.21092	0.660181	-	-0.738369	0.908745	0.416496	A	T
chr1:65392779	A	C	4007	0.00087347	-0.942417	1.61025	0.551559	-	-0.58526	0.788048	0.457681	C	A
chr1:155235196	G	A	4007	0.00012478	0.477749	0.249336	0.915408	#ERROR!	1.91609	2.00266	0.338684	A	G
chr1:155235772	C	A	4007	0.00012478	0.593588	0.240122	1.46736	#ERROR!	2.47203	2.04072	0.225762	A	C
chr1:155235843	T	C	4007	0.00686299	10.2326	11.1585	9.38348	#ERROR!	0.917022	0.299363	0.00218949	C	T
chr1:155236246	G	A	4007	0.00860993	4.59119	15.2782	1.37968	#ERROR!	0.300507	0.255837	0.240155	A	G
chr1:155236376	C	T	4007	0.0133516	17.4039	23.5885	12.8409	#ERROR!	0.737814	0.205897	0.00033913	T	C
chr1:155237423	G	A	4007	0.00024956	-0.792767	0.463083	1.35716	-	-1.71193	1.4695	0.24403	A	G
chr1:155237438	C	T	4007	0.00024956	1.20076	0.478133	3.01551	#ERROR!	2.51134	1.44619	0.0824717	T	C
chr1:155237453	C	T	4007	0.00037435	0.699434	0.718364	0.681003	#ERROR!	0.973649	1.17985	0.409242	T	C
chr1:155237458	A	C	4007	0.00024956	-0.48555	0.353316	0.667275	-	-1.37427	1.68236	0.414003	C	A
chr1:175386035	T	C	4007	0.00049913	-0.85889	0.985166	0.748799	-	-0.871822	1.0075	0.386857	C	T
chr1:175403578	T	G	4007	0.00573996	-2.54273	9.97503	0.648168	-	-0.25491	0.316623	0.420769	G	T
chr1:175406219	T	C	4007	0.00673821	0.259344	10.8877	0.00617753	#ERROR!	0.0238198	0.303062	0.937353	C	T
chr2:74530221	T	C	4007	0.00311954	0.217546	5.21378	0.00907714	#ERROR!	0.0417252	0.437949	0.924097	C	T
chr2:232747717	G	T	4007	0.00037435	0.768818	0.716701	0.824725	#ERROR!	1.07272	1.18122	0.363802	T	G
chr2:232747740	A	G	4007	0.00087347	-0.167349	1.39245	0.0201126	-	-0.120183	0.847441	0.887223	G	A
chr2:232790836	G	C	4007	0.00012478	0.461944	0.248268	0.859523	#ERROR!	1.86067	2.00696	0.353872	C	G
chr2:232791124	T	A	4007	0.00037435	0.233234	0.531191	0.102408	#ERROR!	0.439078	1.37206	0.748959	A	T
chr2:232791432	C	T	4007	0.00012478	-0.0978034	0.0880968	0.10858	-	-1.11018	3.36915	0.741767	T	C

chr2:232794835	A	C	4007	0.00112303	-0.687569	1.63668	0.288847	-	-0.420099	0.78166	0.59096	C	A
chr2:232794843	C	A	4007	0.0207138	-0.226714	33.632	0.00152829	-	-0.006741	0.172434	0.968816	A	C
chr2:232839964	G	A	4007	0.0013726	-0.0557632	2.09837	0.00148188	-	-0.0265745	0.690333	0.969293	A	G
chr2:232844373	C	G	4007	0.00124782	-3.22014	2.38494	4.34784	-	-1.3502	0.647532	0.0370559	G	C
chr2:232847399	A	G	4007	0.00174694	-0.473959	3.17056	0.0708509	-	-0.149487	0.561606	0.790102	G	A
chr3:132450732	A	T	4007	0.00012478	-0.543358	0.247852	1.19119	-	-2.19227	2.00865	0.27509	T	A
chr3:132454135	C	T	4007	0.00012478	0.790911	0.165122	3.78835	#ERROR!	4.78985	2.46092	0.0516105	T	C
chr3:132456751	C	T	4007	0.00024956	1.01656	0.498811	2.07171	#ERROR!	2.03796	1.4159	0.150053	T	C
chr3:132466351	A	C	4007	0.00012478	-0.500066	0.249525	1.00217	-	-2.00407	2.0019	0.316787	C	A
chr3:132475009	C	A	4007	0.00973297	-5.22727	16.4863	1.6574	-	-0.317067	0.246285	0.197955	A	C
chr3:132478139	G	A	4007	0.00262041	0.0538829	4.04226	0.00071825	#ERROR!	0.0133299	0.497379	0.978619	A	G
chr3:132484650	C	T	4007	0.00099825	-0.541236	1.52288	0.192357	-	-0.355404	0.810341	0.660962	T	C
chr3:132492497	A	G	4007	0.00124782	-0.414332	2.13287	0.0804883	-	-0.19426	0.684728	0.776637	G	A
chr3:132494190	A	G	4007	0.0047417	-0.770191	8.11542	0.0730948	-	-0.0949047	0.35103	0.786883	G	A
chr3:132499168	G	A	4007	0.00037435	1.01887	0.62456	1.66212	#ERROR!	1.63134	1.26536	0.197317	A	G
chr3:132499231	C	T	4007	0.0164712	-5.6987	29.0064	1.11959	-	-0.196463	0.185675	0.290008	T	C
chr3:132499777	G	A	4007	0.0117295	1.68436	19.4491	0.145871	#ERROR!	0.0866034	0.226752	0.702513	A	G
chr3:132499779	G	T	4007	0.468181	-38.3305	436.287	3.36756	-	-0.087856	0.0478755	0.0664921	T	G
chr3:132502295	C	T	4007	0.0316945	2.33425	51.7841	0.10522	#ERROR!	0.0450765	0.138964	0.745654	T	C
chr3:132502299	G	A	4007	0.00286998	0.213185	5.03932	0.00901866	#ERROR!	0.0423044	0.445465	0.924341	A	G
chr3:132507256	A	G	4007	0.0657599	7.71095	105.542	0.563364	#ERROR!	0.0730603	0.0973389	0.452908	G	A
chr3:132511169	G	C	4007	0.00149738	2.17528	2.67799	1.76693	#ERROR!	0.812278	0.611076	0.183763	C	G
chr3:132522838	C	T	4007	0.00262041	1.31872	4.78315	0.363574	#ERROR!	0.275702	0.457239	0.546528	T	C
chr3:132522950	T	G	4007	0.00062391	-0.979885	1.17974	0.813886	-	-0.830593	0.920676	0.366974	G	T
chr3:132523636	G	C	4007	0.00149738	-0.840314	2.78302	0.253727	-	-0.301943	0.599434	0.614463	C	G
chr3:132525718	G	T	4007	0.00174694	-1.11483	3.12325	0.397937	-	-0.356947	0.565844	0.528157	T	G
chr3:132528316	T	G	4007	0.00386823	-0.918524	6.91972	0.121925	-	-0.13274	0.380151	0.726956	G	T
chr3:132538225	G	A	4007	0.00386823	-0.936032	6.65183	0.131716	-	-0.140718	0.38773	0.716659	A	G
chr3:184315839	T	C	4007	0.00074869	-1.07872	1.30442	0.892072	-	-0.826971	0.875569	0.344916	C	T
chr3:184319745	G	A	4007	0.00262041	2.56099	4.81237	1.36288	#ERROR!	0.532169	0.455848	0.243039	A	G
chr3:184320694	G	A	4007	0.00024956	-0.758683	0.464361	1.23955	-	-1.63382	1.46748	0.265557	A	G
chr3:184321516	A	G	4007	0.00037435	-0.934966	0.556362	1.57121	-	-1.6805	1.34067	0.210031	G	A
chr3:184321599	G	C	4007	0.00024956	-0.797734	0.471808	1.34881	-	-1.6908	1.45585	0.245486	C	G
chr3:184322040	C	T	4007	0.00212129	0.946974	3.56177	0.251774	#ERROR!	0.265872	0.529867	0.615829	T	C
chr3:184322551	C	T	4007	0.00024956	-0.494257	0.36048	0.677679	-	-1.37111	1.66556	0.410387	T	C
chr3:184322583	G	C	4007	0.00149738	-1.71287	2.55669	1.14754	-	-0.669954	0.625404	0.284064	C	G
chr3:184322631	C	T	4007	0.00012478	-0.224382	0.173793	0.289696	-	-1.29108	2.39874	0.590416	T	C
chr3:184323209	G	T	4007	0.00012478	-0.173177	0.142938	0.209813	-	-1.21155	2.645	0.646914	T	G
chr3:184323412	G	C	4007	0.00024956	0.29458	0.455203	0.190634	#ERROR!	0.64714	1.48217	0.662389	C	G
chr3:184323468	G	C	4007	0.00174694	-0.185582	3.13633	0.0109812	-	-0.0591717	0.564663	0.916542	C	G
chr3:184323921	A	G	4007	0.00074869	-2.53783	1.34667	4.78259	-	-1.88452	0.861725	0.0287489	G	A
chr3:184327230	G	A	4007	0.00012478	-0.224382	0.173793	0.289696	-	-1.29108	2.39874	0.590416	A	G
chr3:184327401	G	A	4007	0.00024956	1.03562	0.498668	2.15073	#ERROR!	2.07677	1.4161	0.142502	A	G
chr3:184327433	C	T	4007	0.00012478	0.559806	0.245991	1.27396	#ERROR!	2.27572	2.01623	0.259025	T	C
chr3:184327609	C	G	4007	0.00661343	-5.1413	11.0771	2.38626	-	-0.464136	0.30046	0.122406	G	C
chr3:184327622	T	C	4007	0.0212129	7.09416	37.0333	1.35897	#ERROR!	0.191562	0.164325	0.243716	C	T
chr3:184328741	T	C	4007	0.00124782	1.91305	1.8985	1.92771	#ERROR!	1.00766	0.725763	0.165009	C	T
chr3:184331762	C	T	4007	0.00024956	0.228779	0.462045	0.113278	#ERROR!	0.495143	1.47115	0.736442	T	C
chr3:195867623	C	T	4007	0.0153481	-4.91541	27.9153	0.86552	-	-0.176083	0.189269	0.352198	T	C
chr3:195868341	C	T	4007	0.00124782	0.533661	2.10351	0.13539	#ERROR!	0.253701	0.68949	0.712907	T	C
chr4:41261759	A	C	4007	0.00124782	-0.178635	2.35748	0.0135359	-	-0.0757738	0.651292	0.90738	C	A

chr4:41261921	C	T	4007	0.00049913	1.3342	0.959758	1.85474	#ERROR!	1.39015	1.02075	0.173233	T	C
chr4:89828156	A	C	4007	0.00024956	1.29674	0.453463	3.70819	#ERROR!	2.85963	1.48501	0.054146	C	A
chr5:1394741	C	G	4007	0.00049913	-0.640848	0.912731	0.449953	-	-0.702121	1.04672	0.502357	G	C
chr5:1403013	G	A	4007	0.00049913	0.639894	0.787772	0.519775	#ERROR!	0.812283	1.12668	0.470938	A	G
chr5:1432618	G	A	4007	0.00062391	-0.900543	1.12636	0.719996	-	-0.799513	0.942238	0.396145	A	G
chr5:1443128	C	T	4007	0.00124782	0.342161	2.85954	0.0409416	#ERROR!	0.119656	0.59136	0.839651	T	C
chr5:122450987	G	A	4007	0.00049913	0.61791	0.870039	0.438846	#ERROR!	0.71021	1.07209	0.50768	A	G
chr5:176621218	G	T	4007	0.00062391	1.27687	1.06149	1.53596	#ERROR!	1.20291	0.970603	0.21522	T	G
chr6:161350187	G	A	4007	0.00212129	-0.823564	3.85384	0.175995	-	-0.213699	0.509393	0.674838	A	G
chr6:161350208	C	T	4007	0.00037435	-0.498275	0.7444	0.333528	-	-0.669365	1.15904	0.563589	T	C
chr6:161360169	G	A	4007	0.00237085	-1.0888	3.58762	0.330437	-	-0.303487	0.527955	0.565402	A	G
chr6:161569358	C	G	4007	0.00049913	0.41291	0.947355	0.179969	#ERROR!	0.435856	1.02741	0.6714	G	C
chr6:161785820	G	A	4007	0.0033691	-3.49765	5.8644	2.08607	-	-0.59642	0.412941	0.148648	A	G
chr6:161785877	G	A	4007	0.00124782	0.764197	2.02005	0.2891	#ERROR!	0.378305	0.703588	0.590798	A	G
chr6:161973335	C	T	4007	0.00024956	-0.576022	0.33595	0.987651	-	-1.71461	1.72529	0.320317	T	C
chr6:162443371	G	A	4007	0.00049913	-0.464874	0.879441	0.245733	-	-0.528601	1.06634	0.620096	A	G
chr6:162443383	C	T	4007	0.00012478	-0.50307	0.249373	1.01486	-	-2.01734	2.00251	0.31374	T	C
chr11:94447275	C	T	4007	0.00087347	0.988958	1.32903	0.735901	#ERROR!	0.744117	0.867425	0.390977	T	C
chr12:40235634	T	C	4007	0.00249563	1.13895	3.642	0.356183	#ERROR!	0.312728	0.523999	0.550634	C	T
chr12:40240594	G	C	4007	0.00012478	0.514218	0.249513	1.05974	#ERROR!	2.06088	2.00195	0.303274	C	G
chr12:40249843	C	G	4007	0.00037435	-1.54685	0.745097	3.21133	-	-2.07604	1.15849	0.0731301	G	C
chr12:40251273	G	A	4007	0.00099825	-0.208791	1.82837	0.0238429	-	-0.114195	0.73955	0.877285	A	G
chr12:40251346	C	T	4007	0.00012478	-0.347366	0.226505	0.532717	-	-1.53359	2.10117	0.465467	T	C
chr12:40263806	A	G	4007	0.00062391	0.971878	1.17599	0.803194	#ERROR!	0.826436	0.922144	0.37014	G	A
chr12:40278179	A	G	4007	0.00012478	-0.501576	0.249765	1.00726	-	-2.00819	2.00094	0.31556	G	A
chr12:40284011	G	T	4007	0.00037435	0.843107	0.659905	1.07717	#ERROR!	1.27762	1.231	0.299332	T	G
chr12:40293624	G	C	4007	0.00037435	1.02413	0.622591	1.68465	#ERROR!	1.64495	1.26736	0.194307	C	G
chr12:40304000	G	A	4007	0.00012478	0.573473	0.244466	1.34527	#ERROR!	2.34582	2.02251	0.246107	A	G
chr12:40304040	G	C	4007	0.00024956	0.0341847	0.498298	0.00234517	#ERROR!	0.0686029	1.41663	0.961376	C	G
chr12:40308481	G	A	4007	0.00062391	2.24364	1.10158	4.56972	#ERROR!	2.03674	0.952778	0.0325419	A	G
chr12:40320103	A	G	4007	0.00037435	-0.301157	0.700285	0.129513	-	-0.43005	1.19499	0.718938	G	A
chr12:40322386	G	T	4007	0.00024956	1.3399	0.435039	4.1268	#ERROR!	3.07994	1.51613	0.0422091	T	G
chr12:40323256	T	C	4007	0.00012478	-0.470477	0.248881	0.889375	-	-1.89037	2.00449	0.345647	C	T
chr12:40335031	G	A	4007	0.00012478	0.474534	0.24903	0.904239	#ERROR!	1.90553	2.00389	0.341648	A	G
chr12:40340400	G	A	4007	0.00349389	13.8512	5.93653	32.3177	#ERROR!	2.33321	0.410425	1.31E-08	A	G
chr12:40351585	G	A	4007	0.00012478	-0.37799	0.234787	0.608537	-	-1.60993	2.06378	0.435339	A	G
chr12:40351723	A	G	4007	0.00012478	-0.430488	0.244962	0.756526	-	-1.75737	2.02046	0.384418	G	A
chr12:40363440	C	T	4007	0.00012478	0.63173	0.232357	1.71754	#ERROR!	2.71879	2.07454	0.19001	T	C
chr12:40363526	G	A	4007	0.00012478	0.676395	0.218512	2.09376	#ERROR!	3.09546	2.13925	0.147902	A	G
chr14:22873373	C	T	4007	0.00012478	0.530682	0.24876	1.13211	#ERROR!	2.13331	2.00498	0.287326	T	C
chr14:22875363	A	G	4007	0.00062391	0.449992	0.999278	0.202639	#ERROR!	0.450317	1.00036	0.652599	G	A
chr14:22875639	C	T	4007	0.00049913	-1.10087	0.750434	1.61494	-	-1.46697	1.15437	0.203798	T	C
chr14:22875648	G	A	4007	0.00012478	-0.150609	0.12776	0.177545	-	-1.17885	2.79771	0.673491	A	G
chr14:22875924	G	A	4007	0.00099825	-0.209868	1.84348	0.023892	-	-0.113843	0.736513	0.87716	A	G
chr14:22876113	G	A	4007	0.00012478	-0.568976	0.24464	1.3233	-	-2.32577	2.02179	0.25	A	G
chr14:22876722	G	C	4007	0.00012478	0.462105	0.248266	0.860132	#ERROR!	1.86133	2.00697	0.353702	C	G
chr14:22876816	G	A	4007	0.00112303	-0.948907	1.8193	0.494928	-	-0.521577	0.741391	0.481737	A	G
chr14:22877070	G	A	4007	0.00598952	2.4551	10.8037	0.557916	#ERROR!	0.227247	0.304239	0.455101	A	G
chr14:22877108	G	A	4007	0.00037435	1.15707	0.559969	2.39087	#ERROR!	2.06631	1.33634	0.122046	A	G
chr14:22877421	A	C	4007	0.00037435	-0.337468	0.69933	0.162848	-	-0.482559	1.1958	0.686548	C	A
chr15:50586433	G	A	4007	0.105316	0.184018	163.686	0.00020688	#ERROR!	0.00112422	0.0781618	0.988524	A	G

chr15:61854543	T	C	4007	0.00561517	0.908236	9.57591	0.0861425	#ERROR!	0.0948459	0.323154	0.76914	C	T
chr15:61854888	C	A	4007	0.00112303	1.54551	2.0495	1.16546	#ERROR!	0.754093	0.698516	0.280336	A	C
chr15:61856407	C	T	4007	0.00037435	-0.416368	0.740075	0.234249	-	-0.562602	1.16242	0.628391	T	C
chr15:61856408	G	A	4007	0.00012478	-0.301249	0.210208	0.43172	-	-1.4331	2.1811	0.511146	A	G
chr15:61868675	C	T	4007	0.00037435	-0.0724703	0.68616	0.00765411	-	-0.105617	1.20722	0.930284	T	C
chr15:61868702	C	T	4007	0.00074869	0.85116	1.23571	0.58628	#ERROR!	0.688802	0.899584	0.443861	T	C
chr15:61880871	G	A	4007	0.00012478	-0.461382	0.248186	0.857718	-	-1.85902	2.0073	0.354378	A	G
chr15:61882609	A	G	4007	0.00037435	-0.179797	0.669318	0.0482985	-	-0.268628	1.22232	0.826051	G	A
chr15:61910283	T	C	4007	0.481532	-45.0231	433.014	4.68133	-	-0.103976	0.0480561	0.0304922	C	T
chr15:61911844	G	A	4007	0.00361867	-3.31484	6.21325	1.7685	-	-0.533511	0.401181	0.183568	A	G
chr15:61915655	T	C	4007	0.00049913	0.647068	0.863512	0.484876	#ERROR!	0.749344	1.07613	0.486222	C	T
chr15:61915712	A	G	4007	0.00786124	1.86232	14.3238	0.24213	#ERROR!	0.130015	0.264223	0.622672	G	A
chr15:61916007	G	C	4007	0.00037435	-0.325468	0.732786	0.144557	-	-0.444152	1.16818	0.703792	C	G
chr15:61920099	C	T	4007	0.00798602	0.412236	12.955	0.0131176	#ERROR!	0.0318206	0.277832	0.908816	T	C
chr15:61920268	C	T	4007	0.00224607	-2.89458	3.7074	2.25996	-	-0.780756	0.519356	0.132757	T	C
chr15:61920571	T	C	4007	0.00511605	0.297423	8.40339	0.0105268	#ERROR!	0.0353932	0.344963	0.91828	C	T
chr15:61922408	C	T	4007	0.0783629	7.60662	126.756	0.456471	#ERROR!	0.0600097	0.0888209	0.499278	T	C
chr15:61922557	A	G	4007	0.00099825	1.2955	1.61916	1.03653	#ERROR!	0.800105	0.785879	0.308629	G	A
chr15:61927138	G	A	4007	0.0139755	3.3388	23.5571	0.473216	#ERROR!	0.141732	0.206034	0.491512	A	G
chr15:61931104	C	G	4007	0.0175942	9.12759	31.9899	2.60435	#ERROR!	0.285328	0.176805	0.106571	G	C
chr15:61934224	T	G	4007	0.00873471	0.602931	14.0967	0.0257879	#ERROR!	0.0427709	0.266343	0.872419	G	T
chr15:61940651	A	G	4007	0.00012478	-0.298512	0.209184	0.425987	-	-1.42703	2.18643	0.513965	G	A
chr15:61945803	G	A	4007	0.00349389	-1.33097	5.25642	0.337011	-	-0.253208	0.436169	0.561559	A	G
chr15:61950998	T	C	4007	0.0786124	8.17909	126.992	0.526786	#ERROR!	0.0644064	0.0887385	0.467961	C	T
chr15:61954471	T	G	4007	0.0013726	-0.737442	2.64366	0.205708	-	-0.278948	0.615032	0.650153	G	T
chr15:61961592	T	C	4007	0.0783629	6.68596	127.231	0.351345	#ERROR!	0.0525497	0.088655	0.553353	C	T
chr15:61962390	T	G	4007	0.00024956	1.32214	0.436326	4.0063	#ERROR!	3.03016	1.51389	0.0453305	G	T
chr15:61962790	T	C	4007	0.0197155	1.04065	33.7685	0.0320699	#ERROR!	0.0308172	0.172085	0.857874	C	T
chr15:61963904	C	T	4007	0.00024956	1.26913	0.452255	3.56144	#ERROR!	2.80621	1.48699	0.0591364	T	C
chr15:61966088	C	T	4007	0.00024956	-0.168557	0.153932	0.184571	-	-1.09501	2.5488	0.667474	T	C
chr15:61967438	T	C	4007	0.408286	33.747	426.644	2.66934	#ERROR!	0.0790987	0.0484136	0.102298	C	T
chr15:61969413	T	C	4007	0.0047417	0.508029	8.81004	0.0292953	#ERROR!	0.0576647	0.336908	0.864099	C	T
chr15:61978646	T	C	4007	0.00112303	-1.27331	1.98603	0.816366	-	-0.641135	0.70959	0.366245	C	T
chr15:61984885	A	T	4007	0.00012478	0.780449	0.171047	3.56101	#ERROR!	4.56277	2.41792	0.0591517	T	A
chr15:62008671	T	C	4007	0.00062391	-0.210416	1.1788	0.0375594	-	-0.1785	0.921043	0.84633	C	T
chr15:62010533	G	A	4007	0.00012478	-0.511583	0.249241	1.05006	-	-2.05256	2.00304	0.305494	A	G
chr15:62012166	T	C	4007	0.00012478	-0.482637	0.249397	0.934007	-	-1.93522	2.00242	0.333824	C	T
chr15:62023783	T	C	4007	0.00112303	-1.85597	1.70411	2.02137	-	-1.08912	0.76604	0.155099	C	T
chr15:62023836	C	T	4007	0.0173446	1.96195	29.6564	0.129795	#ERROR!	0.0661561	0.183629	0.718644	T	C
chr15:62034998	G	A	4007	0.00012478	-0.50394	0.249652	1.01724	-	-2.01857	2.00139	0.313175	A	G
chr15:89318587	G	A	4007	0.00024956	-0.335549	0.278342	0.404513	-	-1.20553	1.89544	0.524768	A	G
chr15:89318595	T	C	4007	0.0424258	7.73715	69.5215	0.86108	#ERROR!	0.111292	0.119934	0.353437	C	T
chr15:89318641	G	A	4007	0.00024956	0.584955	0.280722	1.2189	#ERROR!	2.08375	1.88739	0.269576	A	G
chr15:89319073	A	G	4007	0.00062391	1.49868	0.979521	2.29299	#ERROR!	1.53001	1.0104	0.129959	G	A
chr15:89319234	G	A	4007	0.00024956	-0.963553	0.484647	1.91569	-	-1.98815	1.43644	0.166332	A	G
chr15:89321217	G	A	4007	0.00012478	0.513617	0.249451	1.05753	#ERROR!	2.05899	2.0022	0.303779	A	G
chr15:89321780	G	A	4007	0.00037435	-1.4897	0.739784	2.99981	-	-2.0137	1.16265	0.0832744	A	G
chr15:89321792	C	T	4007	0.00012478	-0.394092	0.238338	0.651631	-	-1.6535	2.04835	0.41953	T	C
chr15:89321842	T	C	4007	0.00836037	-2.18932	15.1581	0.316208	-	-0.144432	0.256849	0.573895	C	T
chr15:89322799	C	T	4007	0.00012478	-0.462583	0.24813	0.862384	-	-1.86428	2.00752	0.353072	T	C
chr15:89323460	C	G	4007	0.00174694	-1.72383	3.27069	0.908547	-	-0.527053	0.552943	0.3405	G	C

chr15:89323462	T	C	4007	0.00049913	0.448943	0.856285	0.235377	#ERROR!	0.524291	1.08066	0.627565	C	T
chr15:89324193	C	T	4007	0.00037435	-0.862926	0.586045	1.27062	-	-1.47246	1.30627	0.25965	T	C
chr15:89325591	A	G	4007	0.00012478	-0.367223	0.232053	0.581129	-	-1.5825	2.0759	0.44587	G	A
chr15:89325639	G	A	4007	0.0033691	0.324781	5.69588	0.0185191	#ERROR!	0.0570203	0.419005	0.891754	A	G
chr15:89326947	C	A	4007	0.00686299	3.6547	11.4123	1.17039	#ERROR!	0.320243	0.296015	0.279321	A	C
chr15:89327004	T	G	4007	0.00024956	1.16851	0.479015	2.85045	#ERROR!	2.4394	1.44486	0.0913483	G	T
chr15:89327198	T	C	4007	0.00024956	0.143855	0.472063	0.043838	#ERROR!	0.304737	1.45546	0.834155	C	T
chr15:89327201	C	T	4007	0.00099825	-1.87731	1.70315	2.06928	-	-1.10226	0.766256	0.150293	T	C
chr15:89328532	G	C	4007	0.00149738	-1.02336	2.71902	0.385166	-	-0.376373	0.606448	0.534851	C	G
chr15:89328996	G	A	4007	0.00099825	-2.1296	1.85247	2.4482	-	-1.1496	0.734725	0.11766	A	G
chr15:89330081	C	G	4007	0.00012478	-0.62188	0.234671	1.64799	-	-2.65001	2.06429	0.199233	G	C
chr15:89330106	T	A	4007	0.00074869	1.08292	1.1892	0.986141	#ERROR!	0.910631	0.917007	0.320687	A	T
chr15:89330133	C	G	4007	0.00573996	3.1476	9.56439	1.03586	#ERROR!	0.329096	0.323349	0.308786	G	C
chr15:89330184	G	A	4007	0.00349389	-0.0749838	5.93052	0.00094807	-	-0.0126437	0.410633	0.975436	A	G
chr15:89330258	C	G	4007	0.00087347	0.481139	1.56106	0.148293	#ERROR!	0.308213	0.800369	0.700172	G	C
chr15:89333364	A	G	4007	0.00074869	0.392347	1.10486	0.139326	#ERROR!	0.355109	0.951362	0.708952	G	A
chr15:89333621	T	C	4007	0.00037435	0.778912	0.695137	0.872784	#ERROR!	1.12052	1.1994	0.350186	C	T
chr15:89333627	T	C	4007	0.00449214	3.86724	7.71003	1.93975	#ERROR!	0.501586	0.36014	0.163695	C	T
chr15:89333723	C	T	4007	0.00049913	-1.03545	0.985041	1.08845	-	-1.05118	1.00756	0.296815	T	C
chr20:5069196	G	T	4007	0.00224607	5.25968	3.86769	7.15264	#ERROR!	1.3599	0.50848	0.00748538	T	G
chr20:5069288	C	T	4007	0.00024956	0.212975	0.447029	0.101467	#ERROR!	0.476424	1.49566	0.750077	T	C
chr20:5100832	G	A	4007	0.00249563	0.127656	4.43704	0.00367273	#ERROR!	0.0287705	0.474737	0.951675	A	G
chr20:5106224	T	C	4007	0.00049913	-0.245743	0.835985	0.0722375	-	-0.293956	1.09371	0.788106	C	T
chr20:5109429	A	G	4007	0.00162216	-2.85761	2.4946	3.27346	-	-1.14552	0.63314	0.0704091	G	A
chr20:5109435	C	T	4007	0.00012478	0.47318	0.248684	0.900338	#ERROR!	1.90274	2.00528	0.342691	T	C
chr21:32639773	G	A	4007	0.00012478	-0.0750515	0.0693013	0.0812789	-	-1.08297	3.79865	0.775572	A	G
chr21:32664963	C	A	4007	0.00012478	-0.341982	0.224483	0.520981	-	-1.52342	2.11061	0.470423	A	C
chr21:32665968	A	G	4007	0.00149738	0.102962	2.75577	0.00384693	#ERROR!	0.0373625	0.602391	0.950544	G	A
chr21:32673463	C	T	4007	0.00037435	-0.920115	0.620819	1.3637	-	-1.4821	1.26916	0.242897	T	C
chr21:32673511	T	C	4007	0.00012478	0.599382	0.239738	1.49855	#ERROR!	2.50015	2.04236	0.220895	C	T
chr22:32475378	G	A	4007	0.101947	12.0205	156.336	0.924238	#ERROR!	0.0768887	0.079978	0.336365	A	G
chr22:32479135	T	G	4007	0.00024956	-0.981058	0.498572	1.93046	-	-1.96774	1.41624	0.164708	G	T
chr22:32498414	G	A	4007	0.0013726	3.19521	2.28309	4.47174	#ERROR!	1.39951	0.661819	0.03446	A	G
chr22:38113621	C	T	4007	0.00112303	0.466358	2.11141	0.103007	#ERROR!	0.220875	0.688198	0.748251	T	C
chr22:38116105	C	T	4007	0.00037435	0.601675	0.730745	0.495402	#ERROR!	0.823371	1.16981	0.481528	T	C
chr22:38116119	C	A	4007	0.00012478	-0.110538	0.0981904	0.124439	-	-1.12575	3.19128	0.724269	A	C
chr22:38120886	C	T	4007	0.00074869	-0.286535	1.36275	0.0602473	-	-0.210262	0.856626	0.806105	T	C
chr22:38126371	G	A	4007	0.00037435	0.745122	0.666252	0.833328	#ERROR!	1.11838	1.22513	0.361312	A	G
chr22:38126374	C	T	4007	0.00062391	0.0863056	1.14429	0.00650942	#ERROR!	0.075423	0.93483	0.935696	T	C
chr22:38126390	T	C	4007	0.00012478	0.639192	0.230388	1.77339	#ERROR!	2.77442	2.08339	0.182963	C	T
chr22:38126417	G	A	4007	0.00024956	0.395115	0.417675	0.373774	#ERROR!	0.945987	1.54732	0.540955	A	G
chr22:38132881	C	T	4007	0.014849	6.60545	24.9396	1.74951	#ERROR!	0.264858	0.200242	0.185939	T	C
chr22:38132917	C	T	4007	0.00012478	0.791945	0.164481	3.81307	#ERROR!	4.81482	2.46571	0.0508541	T	C
chr22:38132952	G	A	4007	0.00037435	0.763097	0.712862	0.816872	#ERROR!	1.07047	1.1844	0.366096	A	G
chr22:38133007	G	A	4007	0.00024956	0.224236	0.473431	0.106207	#ERROR!	0.473639	1.45335	0.744505	A	G
chr22:38133010	C	T	4007	0.00024956	-0.656627	0.380215	1.13399	-	-1.72699	1.62176	0.286926	T	C
chr22:38140006	G	A	4007	0.00012478	-0.391364	0.237068	0.646084	-	-1.65085	2.05383	0.421516	A	G
chr22:38140069	C	T	4007	0.00024956	0.251646	0.44632	0.141884	#ERROR!	0.563824	1.49685	0.706415	T	C
chr22:38143150	G	A	4007	0.00012478	-0.160792	0.134673	0.191978	-	-1.19395	2.72496	0.661276	A	G
chr22:38143219	C	G	4007	0.00012478	-0.506526	0.249682	1.02758	-	-2.02868	2.00127	0.310728	G	C
chr22:38145447	C	T	4007	0.00124782	-0.303055	2.35485	0.0390013	-	-0.128694	0.651656	0.843446	T	C

chr22:38169255	C	T	4007	0.00024956	0.178046	0.483094	0.0656193	#ERROR!	0.368553	1.43875	0.797825	T	C
chr22:38169326	G	A	4007	0.00062391	0.499532	1.23826	0.201519	#ERROR!	0.403415	0.898658	0.653498	A	G
chr22:38169336	C	T	4007	0.00074869	-1.62611	1.36584	1.93597	-	-1.19055	0.855656	0.164106	T	C

Supplementary Table 7: Meta-analysis summary statistics

MarkerName	Allele1	Allele2	Freq1	FreqSE	MinFreq	MaxFreq	Effect	StdErr	P-value	Direction	HetISq	HetChiSq	HetDf	HetPVal
chr1:20638104	a	c	1	0	1	1	1.0083	18.4693	0.9565	??+	0	0	0	1
chr22:38112571	a	c	0.9999	0	0.9999	0.9999	-0.3509	0.6857	0.6088	?--	0	0.349	1	0.5548
chr3:195867623	t	c	0.9823	0.0011	0.9802	0.9847	-0.0111	0.0313	0.7225	#ERROR!	0	1.408	2	0.4945
chr1:155235727	c	g	1.00E-04	0	1.00E-04	1.00E-04	0.1939	0.4011	0.6289	?-+	0	0.086	1	0.7692
chr15:89318617	t	c	1	0	1	1	-4.0112	2.3039	0.08167	?-+	0	0.212	1	0.6452
chr2:232791124	a	t	0.9994	0	0.9994	0.9996	-0.1359	0.1863	0.4657	-+-	0	0.326	2	0.8496
chr1:155238258	a	g	1	0	1	1	2.0301	14.4002	0.8879	??+	0	0	0	1
chr6:161785877	a	g	0.9992	1.00E-04	0.9988	0.9992	-0.2652	0.164	0.1059	-+-	11.7	2.264	2	0.3223
chr12:40322386	t	g	0.9999	0	0.9998	0.9999	-0.9109	0.2097	1.40E-05	-?-	52.1	2.087	1	0.1486
chr15:89333621	t	c	1.00E-04	1.00E-04	1.00E-04	4.00E-04	-0.153	0.4397	0.7279	#ERROR!	23.2	1.302	1	0.2538
chr1:65386873	a	g	1	0	0.9999	1	-1.6232	0.785	0.03866	---	0	0.11	2	0.9465
chr1:175406252	a	t	1.00E-04	0	1.00E-04	1.00E-04	-1.0823	0.7973	0.1747	?--	0	0.014	1	0.9062
chr6:162443371	a	g	0.9996	1.00E-04	0.9994	0.9997	0.0896	0.2011	0.6559	#ERROR!	0	1.071	2	0.5854
chr12:40367045	a	g	0.9998	0	0.9998	0.9999	-0.0835	0.4123	0.8395	?--	0	0.017	1	0.8971
chr1:20637956	c	g	1	0	1	1	2.0134	5.779	0.7275	??+	0	0	0	1
chr1:16986292	a	g	0.9998	0	0.9998	0.9998	-0.2841	0.7248	0.6951	?-?	0	0	0	1
chr1:65392454	a	t	0.9993	1.00E-04	0.9991	0.9994	-0.0207	0.167	0.9012	#ERROR!	0	1.517	2	0.4683
chr1:16987187	a	g	0.9999	0	0.9999	0.9999	3.1861	2.817	0.258	??+	0	0	0	1
chr3:184328741	t	c	6.00E-04	2.00E-04	4.00E-04	0.0012	0.0198	0.2206	0.9283	#ERROR!	2.3	2.047	2	0.3594
chr12:40351723	a	g	2.00E-04	0	1.00E-04	2.00E-04	0.0455	0.2959	0.8778	-++	0	0.816	2	0.6648
chr15:89318595	t	c	0.0431	0.0012	0.0404	0.0437	0.0318	0.0202	0.1154	#ERROR!	0	0.553	2	0.7586
chr3:184322040	t	c	0.9988	3.00E-04	0.9979	0.9989	0.0993	0.1388	0.4745	--+	62.8	5.375	2	0.06805
chr12:40251346	t	c	0.9997	0	0.9997	0.9999	-0.0254	0.2786	0.9273	#ERROR!	0	0.845	2	0.6553
chr22:38132952	a	g	0.9995	0	0.9995	0.9996	-0.1515	0.2058	0.4617	--+	24.2	2.638	2	0.2675
chr15:61929659	c	g	1	0	1	1	-2.8964	1.4661	0.0482	??-	0	0	0	1
chr1:155237458	a	c	0	1.00E-04	0	2.00E-04	0.0197	0.5085	0.9691	-+-	0	1.662	2	0.4357
chr12:40257283	t	c	1	0	1	1	2.5301	89.3333	0.9774	??+	0	0	0	1
chr14:22875400	a	g	0.9998	1.00E-04	0.9997	1	1.5256	0.8216	0.06333	?-+	79.2	4.801	1	0.02844
chr3:132516425	a	g	1	0	1	1	-1.7335	0.6922	0.01227	??-	0	0	0	1
chr1:20633840	t	c	1	0	1	1	2.0089	13.0926	0.8781	??+	0	0	0	1
chr15:61983908	a	t	0.9999	0	0.9999	0.9999	0.1637	0.8557	0.8483	??+	0	0	0	1
chr1:20637894	a	g	1	0	1	1	1.4098	2.2635	0.5334	?++	0	0.042	1	0.8369
chr15:62008671	t	c	5.00E-04	2.00E-04	3.00E-04	7.00E-04	0.1534	0.2178	0.4812	-++	0	0.252	2	0.8818
chr15:89321776	t	c	1	0	1	1	1.013	9.0922	0.9113	??+	0	0	0	1
chr1:20644570	t	c	1	0	1	1	1.5458	3.2346	0.6327	?++	0	0.108	1	0.7425
chr6:161973403	a	t	1	0	1	1	-1.4995	0.5536	0.006756	?--	0	0.151	1	0.6976
chr6:162443438	t	c	1	0	1	1	0.9554	15.2135	0.9499	??+	0	0	0	1
chr3:184320694	a	g	0.9996	2.00E-04	0.9994	0.9998	0.1519	0.2531	0.5485	#ERROR!	2.5	2.052	2	0.3584
chr4:41261921	t	c	0.9995	0	0.9995	0.9995	-1.3901	1.0208	0.1732	-??	0	0	0	1
chr3:184321882	t	c	0.9999	0	0.9999	1	-0.481	0.5137	0.3491	?+-	0	0.355	1	0.5515
chr22:38120889	a	g	1	0	1	1	1.4634	2.4916	0.557	?++	0	0.096	1	0.7566
chr12:40284011	t	g	0.9993	1.00E-04	0.9991	0.9996	0.4385	0.2047	0.0322	-++	7	2.152	2	0.341
chr15:89330081	c	g	1.00E-04	0	0	1.00E-04	-0.6985	1.2019	0.5611	-+-	0	1.869	2	0.3928
chr1:155236384	a	g	1	0	1	1	-1.7026	0.5876	0.003759	?+-	71.8	3.547	1	0.05965
chr1:16986321	t	c	1	0	1	1	1.3671	2.2568	0.5447	?+?	0	0	0	1
chr3:132502299	a	g	0.9963	3.00E-04	0.9958	0.9971	0.0448	0.0687	0.5147	-++	0	0.08	2	0.961
chr3:184321599	c	g	0.9998	0	0.9998	0.9999	0.4717	0.3614	0.1918	#ERROR!	0	1.846	2	0.3973
chr15:61915890	a	g	0.9998	0	0.9998	0.9998	0.7765	0.6299	0.2176	??+	0	0	0	1
chr2:232794835	a	c	6.00E-04	2.00E-04	6.00E-04	0.0011	0.1876	0.2307	0.4161	--+	2	2.041	2	0.3604
chr3:132505400	a	t	0	0	0	0	-0.8469	3.8441	0.8256	?--	0	0.002	1	0.9651
chr1:20644665	a	t	0.001	2.00E-04	6.00E-04	0.0011	-0.0308	0.1432	0.8297	#ERROR!	0	0.089	2	0.9563

chr12:40323256	t	c	4.00E-04	1.00E-04	1.00E-04	4.00E-04	0.1448	0.2193	0.5092	--+	0	1.66	2	0.436
chr15:89330250	a	c	7.00E-04	0	7.00E-04	7.00E-04	-0.0306	0.2941	0.917	??-	0	0	0	1
chr15:89330184	a	g	0.9977	2.00E-04	0.9965	0.9977	0.0513	0.0846	0.5441	#ERROR!	0	0.256	2	0.8797
chr1:155235231	t	c	0	0	0	0	-1.0093	17.4341	0.9538	??-	0	0	0	1
chr4:41261759	a	c	0.0015	2.00E-04	0.0012	0.0018	0.0146	0.1175	0.9013	--+	0	1.293	2	0.5239
chr22:38133007	a	g	0.9998	0	0.9998	0.9999	0.6973	0.4605	0.13	-++	0	0.774	2	0.6791
chr15:61915712	a	g	0.01	3.00E-04	0.0079	0.0101	0.0592	0.0412	0.1505	#ERROR!	0	0.083	2	0.9593
chr12:40243556	a	t	0	0	0	0	2.2444	0.9274	0.01552	??+	0	0	0	1
chr5:1432618	a	g	0.9987	1.00E-04	0.9986	0.9994	0.0132	0.1226	0.9142	#ERROR!	0	0.724	2	0.6961
chr15:89318641	a	g	0.9998	1.00E-04	0.9998	1	-1.2954	1.6599	0.4352	-++	0	0.812	2	0.6664
chr20:5069196	t	g	0.9977	5.00E-04	0.9971	0.9981	0.0643	0.1152	0.5766	-++	75.9	8.284	2	0.01589
chr15:62007466	t	c	0	0	0	0	-0.8055	5.127	0.8752	??-	0	0	0	1
chr12:40320042	t	c	1	0	1	1	13.8973	11.2849	0.2181	??+	0	0	0	1
chr3:132522838	t	c	0.9973	4.00E-04	0.9967	0.9976	-0.047	0.0859	0.5838	--+	65.4	5.772	2	0.05579
chr15:89328795	t	c	0	0	0	0	-1.0123	6.676	0.8795	??-	0	0	0	1
chr16:46674332	t	c	1.00E-04	0	1.00E-04	2.00E-04	-0.8731	0.5053	0.08401	?--	0	0.148	1	0.7002
chr15:89325466	a	c	1	0	1	1	0.3311	2.5187	0.8954	??+	0	0	0	1
chr15:89323504	t	c	0.9999	0	0.9999	0.9999	-0.1052	0.5488	0.8479	??-	0	0	0	1
chr15:89327201	t	c	0.9987	2.00E-04	0.9984	0.999	0.0332	0.14	0.8127	#ERROR!	2	2.041	2	0.3605
chr3:132522861	a	g	1	0	1	1	0.7664	1.3017	0.556	??+	0	0	0	1
chr22:38169326	a	g	0.9994	2.00E-04	0.9991	0.9995	0.0197	0.1934	0.9187	-++	0	0.233	2	0.8899
chr1:155236444	a	t	1	0	1	1	1.0084	13.2739	0.9394	??+	0	0	0	1
chr1:20649095	a	g	0	0	0	0	7.365	2.9171	0.01158	?+-	0	0.409	1	0.5225
chr22:32498513	t	c	0.9998	0	0.9998	0.9999	0.181	0.4948	0.7145	?-+	61.5	2.596	1	0.1072
chr12:40305893	t	g	1	0	1	1	1.0014	45.1426	0.9823	??+	0	0	0	1
chr15:89320953	a	g	1	0	1	1	4.3908	15.2236	0.773	??+	0	0	0	1
chr22:38112165	c	g	1	0	0.9999	1	-0.1504	0.8333	0.8568	?+-	0	0.282	1	0.5954
chr12:40240535	t	g	1	0	1	1	1.0007	61.4356	0.987	??+	0	0	0	1
chr21:32641895	t	c	1	0	1	1	1.0117	15.556	0.9481	??+	0	0	0	1
chr1:20633615	a	g	1	0	1	1	1.0414	2.7147	0.7013	??+	0	0	0	1
chr12:40225159	a	g	1	0	1	1	1.0074	17.8847	0.9551	??+	0	0	0	1
chr2:232747740	a	g	6.00E-04	1.00E-04	5.00E-04	9.00E-04	-0.5189	0.2776	0.06159	---	0	0.562	2	0.7551
chr1:17000494	a	g	0.9992	1.00E-04	0.999	0.9999	-0.1577	0.142	0.2666	-+-	37	3.172	2	0.2047
chr1:20644515	c	g	1.00E-04	1.00E-04	0	2.00E-04	0.8317	0.7134	0.2437	#ERROR!	84.9	13.285	2	0.001304
chr3:132475009	a	c	0.9915	0.0016	0.9893	0.9928	0.0574	0.054	0.2875	#ERROR!	12.5	2.285	2	0.3191
chr22:38120886	t	c	0.9986	3.00E-04	0.9981	0.9993	-0.1113	0.1163	0.3387	#ERROR!	0	1.215	2	0.5447
chr14:22875924	a	g	0.999	2.00E-04	0.9989	0.9994	-0.0251	0.1501	0.8674	#ERROR!	0	0.037	2	0.9815
chr15:89330106	a	t	0.9992	2.00E-04	0.9989	0.9994	-0.0613	0.1773	0.7297	--+	0	1.936	2	0.3799
chr6:161360168	t	c	1	0	1	1	1.3181	1.8029	0.4647	?++	0	0.04	1	0.8422
chr15:89333347	c	g	1	0	1	1	11.7618	42.8445	0.7837	??+	0	0	0	1
chr12:40354486	a	t	1.00E-04	0	1.00E-04	1.00E-04	0.2286	0.4471	0.6092	?-+	15.5	1.183	1	0.2767
chr15:89321217	a	g	0.9999	0	0.9998	0.9999	0.7488	0.3767	0.0468	-++	4.1	2.085	2	0.3525
chr12:40251369	a	g	1	0	1	1	1.3639	2.9319	0.6418	?++	0	0.02	1	0.8872
chr3:184321315	a	g	1	0	1	1	1.3956	2.2036	0.5265	?++	0	0	1	0.9889
chr1:20648612	a	g	0.9998	2.00E-04	0.999	0.9999	0.422	0.1749	0.01584	#ERROR!	0	0.195	2	0.9071
chr15:61915725	a	g	1	0	1	1	-0.1113	0.7203	0.8772	?+-	0	0.483	1	0.4873
chr15:61961754	a	g	0.9998	0	0.9997	0.9998	0.4247	0.4116	0.3022	?++	0	0.031	1	0.8603
chr15:89333627	t	c	0.0034	3.00E-04	0.0029	0.0045	-0.0574	0.0754	0.4463	#ERROR!	45.5	3.672	2	0.1594
chr1:16986335	a	g	0.9993	3.00E-04	0.9989	0.9996	0.1176	0.194	0.5445	#ERROR!	0	1.817	2	0.4031
chr1:16986101	a	t	0.9941	6.00E-04	0.9931	0.9945	0.0023	0.0574	0.9678	#ERROR!	0	0.62	2	0.7335
chr1:20637908	t	c	1	0	1	1	-0.5122	0.8499	0.5468	?+-	0	0.572	1	0.4494
chr15:61977193	t	g	1	0	0.9999	1	1.9113	2.1674	0.3779	?++	0	0.356	1	0.5506
chr15:89327300	a	g	0	0	0	0	-1.5237	2.27	0.5021	?--	0	0.076	1	0.7829

chr12:40293629	a	g	1	0	1	1	0.1349	3.0749	0.965	??+	0	0	0	1
chr2:232847564	a	c	0.0028	0	0.0028	0.0028	-0.174	0.1306	0.1827	??-	0	0	0	1
chr15:89326947	a	c	0.9925	3.00E-04	0.992	0.9931	0.0309	0.0487	0.5254	-++	0	1.609	2	0.4472
chr15:61984938	t	c	0	0	0	0	1.088	8.7933	0.9015	??+	0	0	0	1
chr21:32666479	t	c	1	0	1	1	-1.78	0.9202	0.05307	?+-	78.7	4.701	1	0.03015
chr15:89318599	a	g	1	0	1	1	1.4744	3.4118	0.6656	?++	0	0.054	1	0.8164
chr11:94447275	t	c	0.9997	2.00E-04	0.9991	0.9999	0.1795	0.2992	0.5486	-++	0	1.307	2	0.5203
chr20:5109435	t	c	0.9999	0	0.9998	0.9999	-0.5907	0.6145	0.3364	--+	0	1.781	2	0.4105
chr1:155235843	t	c	0.0032	7.00E-04	0.0014	0.0069	0.8155	0.0507	3.31E-58	#ERROR!	0	0.168	2	0.9195
chr12:40351680	c	g	0.9992	0	0.9992	0.9992	0.5511	0.5085	0.2785	??+	0	0	0	1
chr1:155236367	a	g	1	0	1	1	1.0117	12.4413	0.9352	??+	0	0	0	1
chr22:38140106	a	g	1	0	1	1	0.36	1.0238	0.7251	??+	0	0	0	1
chr15:61961728	t	c	1.00E-04	0	1.00E-04	1.00E-04	-0.0495	1.1079	0.9643	?-?	0	0	0	1
chr22:38169336	t	c	0.9995	1.00E-04	0.9993	0.9995	-0.1128	0.198	0.5688	#ERROR!	26	2.702	2	0.259
chr15:89318581	a	g	1	0	1	1	-0.9972	0.9938	0.3157	?+-	0	0.223	1	0.637
chr6:162054109	c	g	1	0	1	1	1.0135	14.5222	0.9444	??+	0	0	0	1
chr1:16985990	t	c	0.9995	1.00E-04	0.9993	0.9999	-0.1097	0.1564	0.4831	#ERROR!	49	3.92	2	0.1408
chr22:38115658	a	g	1	0	1	1	1.5378	2.309	0.5054	?++	0	0.122	1	0.7273
chr1:65384226	a	g	1	0	1	1	-0.3912	1.0818	0.7176	?--	0	0.039	1	0.8425
chr15:89320890	a	g	1	0	1	1	1.002	37.5844	0.9787	??+	0	0	0	1
chr15:89319065	a	g	1	0	0.9999	1	-0.0163	0.6564	0.9801	?+-	0	0.807	1	0.3689
chr15:61868702	t	c	0.9994	2.00E-04	0.9991	0.9995	-0.1913	0.1843	0.2993	---	0	0.783	2	0.6761
chr2:232847399	a	g	0.0021	1.00E-04	0.0017	0.0021	0.0346	0.1019	0.7345	-+-	34.5	3.052	2	0.2174
chr4:89828156	a	c	2.00E-04	0	2.00E-04	3.00E-04	0.7022	0.2847	0.01365	#ERROR!	9.1	2.199	2	0.333
chr16:46683521	a	t	2.00E-04	0	2.00E-04	3.00E-04	0.2959	0.3419	0.3867	?+-	70.9	3.442	1	0.06357
chr15:61950998	t	c	0.0771	0.0013	0.0741	0.0786	0.0168	0.0153	0.2721	#ERROR!	0	1.066	2	0.5867
chr6:161350139	t	c	1	0	1	1	2.0106	11.0557	0.8557	??+	0	0	0	1
chr12:40225558	t	c	1	0	1	1	1.0005	72.3472	0.989	??+	0	0	0	1
chr15:61983868	t	g	1	0	1	1	8.8134	20.1501	0.6618	??+	0	0	0	1
chr2:74530221	t	c	0.0035	5.00E-04	0.0031	0.0043	-0.1007	0.0773	0.1929	#ERROR!	0	0.46	2	0.7945
chr21:32665968	a	g	0.0014	1.00E-04	0.0014	0.0015	-0.0953	0.1125	0.3969	#ERROR!	49.6	3.965	2	0.1377
chr6:161569357	a	g	1	0	1	1	-2	374563.324	1	??-	0	0	0	1
chr21:32673463	t	c	0.9997	1.00E-04	0.9996	0.9999	0.6922	0.5056	0.171	#ERROR!	27	2.74	2	0.2541
chr15:89318710	t	c	1	0	1	1	1.0098	9.5913	0.9162	??+	0	0	0	1
chr4:89828149	t	c	1	0	1	1	-3.637	1.3261	0.006094	??-	0	0	0	1
chr15:89328996	a	g	0.9994	1.00E-04	0.999	0.9995	0.1874	0.2113	0.3751	#ERROR!	7.4	2.159	2	0.3398
chr5:1443128	t	c	0.9994	3.00E-04	0.9988	0.9997	-0.0324	0.2305	0.8881	-+-	0	0.512	2	0.774
chr3:184328662	a	g	1.00E-04	0	1.00E-04	1.00E-04	-1.5618	0.8092	0.05358	?--	56.5	2.301	1	0.1293
chr1:20648534	t	c	0	0	0	0	-2.0166	14.209	0.8871	??-	0	0	0	1
chr1:65392779	a	c	3.00E-04	2.00E-04	1.00E-04	9.00E-04	-0.1539	0.2736	0.5738	--+	17.7	2.43	2	0.2967
chr15:89317460	a	g	0.9999	0	0.9999	0.9999	0.3787	0.7708	0.6232	?++	0	0.469	1	0.4936
chr3:132480451	a	g	1	0	0.9999	1	-0.1623	0.3363	0.6295	?+-	0	0.164	1	0.6858
chr6:161785839	a	t	0	0	0	0	-1.007	10.2001	0.9214	??-	0	0	0	1
chr3:132499779	t	g	0.5286	6.00E-04	0.5284	0.5318	0.0193	0.0091	0.03262	#ERROR!	52.9	2.124	1	0.145
chr12:40257348	t	g	0	0	0	0	-1.0179	12.6706	0.936	??-	0	0	0	1
chr3:184327434	a	g	1	0	1	1	1.9822	2.0805	0.3407	??+	0	0	0	1
chr6:161785844	a	g	0	0	0	0	-1.0035	14.7396	0.9457	??-	0	0	0	1
chr22:38113591	a	g	1	0	1	1	1.0209	7.7563	0.8953	??+	0	0	0	1
chr1:155236376	t	c	0.9876	0.0012	0.9851	0.9882	-0.3632	0.0342	2.63E-26	---	53.9	4.338	2	0.1143
chr3:184323921	a	g	7.00E-04	2.00E-04	5.00E-04	9.00E-04	-0.6243	0.2142	0.003556	---	12.3	2.279	2	0.3199
chr2:232844498	c	g	1	0	1	1	1.3153	17.4126	0.9398	??+	0	0	0	1
chr3:132525718	t	g	0.9989	1.00E-04	0.9983	0.9989	0.0903	0.1349	0.5035	#ERROR!	0	0.287	2	0.8663
chr1:155239639	a	c	0	0	0	0	1.914	1.731	0.2689	?+-	0	0.027	1	0.8706

chr1:155235813	t	g	0	0	0	0	8.1319	3.0325	0.007328	?+-	0	0.126	1	0.7227
chr1:20649034	t	c	0	0	0	0	-1.0357	8.8903	0.9073	??-	0	0	0	1
chr22:38115583	a	g	1	0	1	1	1.0218	6.2572	0.8703	??+	0	0	0	1
chr20:5106224	t	c	0.0013	2.00E-04	5.00E-04	0.0013	-0.1566	0.1325	0.2371	---	9	2.199	2	0.3331
chr12:40363440	t	c	0.9998	0	0.9998	0.9999	0.2493	0.4238	0.5563	-++	6.9	2.149	2	0.3415
chr1:175403578	t	g	0.0058	1.00E-04	0.0057	0.006	0.1313	0.0525	0.01245	--+	54.8	4.425	2	0.1094
chr12:40304040	c	g	0.9996	1.00E-04	0.9995	0.9998	0.1582	0.3352	0.6368	-+-	0	0.159	2	0.9236
chr15:61991076	c	g	1	0	1	1	2.324	15.8724	0.8836	??+	0	0	0	1
chr3:195868341	t	c	0.999	1.00E-04	0.9988	0.9991	-0.213	0.1385	0.1241	---	0	0.267	2	0.8752
chr2:232747717	t	g	0.9998	0	0.9996	1	-0.3786	0.2495	0.1291	-+-	0	0.8	2	0.6703
chr12:40284061	a	g	0	0	0	0	-1.0143	10.9581	0.9263	??-	0	0	0	1
chr1:16986248	t	c	0.5222	0.0071	0.4849	0.5236	0.0324	0.0091	0.0003886	-?+	60.7	2.546	1	0.1106
chr1:20644526	a	c	1	0	1	1	1.0003	72.2717	0.989	??+	0	0	0	1
chr12:40309174	a	g	1	0	1	1	1.0539	7.2883	0.885	??+	0	0	0	1
chr22:38126390	t	c	5.00E-04	0	1.00E-04	5.00E-04	0.0272	0.22	0.9016	#ERROR!	20.2	2.507	2	0.2855
chr15:61929546	a	t	1	0	1	1	-4.3661	1.3521	0.001242	??-	0	0	0	1
chr12:40295466	a	g	1	0	1	1	-1.2027	1.0358	0.2456	??-	0	0	0	1
chr1:16993627	a	c	0.0142	0	0.0142	0.0142	0.1403	0.1547	0.3646	??+	0	0	0	1
chr15:89318677	t	c	1.00E-04	0	0	1.00E-04	-0.548	0.6854	0.424	?-+	0	0.706	1	0.4009
chr1:17011732	c	g	0	0	0	0	-258.3631	476.8564	0.588	??-	0	0	0	1
chr1:155235798	a	g	0	0	0	0	-2.0177	11.1887	0.8569	??-	0	0	0	1
chr21:32673511	t	c	2.00E-04	0	1.00E-04	2.00E-04	-0.019	0.3774	0.9598	#ERROR!	33.8	3.023	2	0.2205
chr12:40323300	t	c	1	0	1	1	-2.032	1.253	0.1049	??-	0	0	0	1
chr15:61951827	t	c	0	0	0	0	1.0956	0.4935	0.02641	??+	0	0	0	1
chr15:89322799	t	c	1	0	0.9999	1	0.8203	0.7168	0.2525	#ERROR!	0	0.31	1	0.5777
chr22:38133010	t	c	0.9998	1.00E-04	0.9998	1	1.6421	1.4407	0.2544	#ERROR!	0	0.086	2	0.9578
chr1:155236441	t	c	0	0	0	0	-1.0015	43.6282	0.9817	??-	0	0	0	1
chr22:38145447	t	c	0.9993	2.00E-04	0.9988	0.9995	0.1544	0.1693	0.3619	#ERROR!	0	1.084	2	0.5816
chr1:155237423	a	g	0.9998	0	0.9998	0.9999	0.1798	0.3716	0.6285	#ERROR!	0	1.387	2	0.4999
chr14:22875772	a	c	1	0	1	1	1.3027	14.2843	0.9273	??+	0	0	0	1
chr2:232794778	t	c	0.9998	0	0.9998	0.9999	-0.2382	0.436	0.5849	?-+	78.8	4.725	1	0.02973
chr12:40278154	a	g	0	0	0	0	-2.0103	14.5687	0.8903	??-	0	0	0	1
chr3:184327609	c	g	0.005	4.00E-04	0.0046	0.0066	0.0312	0.0605	0.6063	-++	29.5	2.836	2	0.2422
chr15:61962790	t	c	0.0252	0.001	0.0197	0.0254	-0.0558	0.0298	0.06099	#ERROR!	0	0.261	1	0.6092
chr22:38116105	t	c	0.9999	1.00E-04	0.9996	0.9999	-0.1645	0.4045	0.6843	---	0	0.361	2	0.8349
chr12:40367012	a	t	1	0	1	1	2.0065	13.9922	0.886	??+	0	0	0	1
chr1:17005517	t	c	0.9998	1.00E-04	0.9996	1	-0.2014	0.3114	0.5178	-+-	0	0.627	2	0.7307
chr3:184315839	t	c	6.00E-04	0	6.00E-04	7.00E-04	-0.1479	0.2047	0.47	-+-	0	1.777	2	0.4113
chr20:5069205	t	c	1	0	1	1	315.9066	1079.1159	0.7697	??+	0	0	0	1
chr15:89325531	a	c	0	0	0	0	-1.9607	6.5676	0.7653	??-	0	0	0	1
chr1:16988226	t	c	0.9997	1.00E-04	0.9997	0.9999	-1.6162	0.5146	0.001686	--+	11	2.248	2	0.3249
chr15:89318989	c	g	1	0	1	1	1.0052	10.7515	0.9255	??+	0	0	0	1
chr1:20645615	a	g	0.9984	5.00E-04	0.9979	0.999	0.0044	0.143	0.9757	--+	0	0.826	2	0.6617
chr3:184320964	t	c	1	0	1	1	-0.1518	1.418	0.9147	??-	0	0	0	1
chr22:38112186	a	t	0	0	0	0	-2.1008	9.4627	0.8243	??-	0	0	0	1
chr12:40351585	a	g	0.9999	0	0.9999	0.9999	-0.4212	0.3289	0.2003	#ERROR!	53.3	4.282	2	0.1176
chr1:155238264	t	c	0.9999	0	0.9999	0.9999	6.3413	4.8684	0.1927	??+	0	0	0	1
chr15:62034998	a	g	0.9998	0	0.9998	0.9999	-0.1471	0.2785	0.5974	#ERROR!	22.1	2.567	2	0.277
chr15:89320832	t	c	1	0	1	1	3.0286	6.9958	0.6651	??+	0	0	0	1
chr22:38112558	t	c	1	0	1	1	-2.1192	0.9607	0.02739	?--	59.6	2.472	1	0.1159
chr15:89327198	t	c	6.00E-04	2.00E-04	2.00E-04	8.00E-04	-0.0805	0.2274	0.7235	#ERROR!	51.6	4.136	2	0.1264
chr3:184331523	c	g	0	0	0	0	3.9644	1.324	0.00275	??+	0	0	0	1
chr6:161386864	t	c	1	0	1	1	-0.9766	0.9122	0.2844	?+-	52.8	2.118	1	0.1455

chr1:20649224	t	c	0.9998	0	0.9998	0.9998	1.9702	2.2912	0.3898	??+	0	0	0	1
chr1:155238215	t	c	0	0	0	0	1.1944	0.4914	0.01506	?++	0	0.156	1	0.6927
chr15:89328699	a	g	1	0	1	1	3.0492	6.9969	0.663	??+	0	0	0	1
chr12:40322038	a	g	1	0	1	1	1.1555	2.0792	0.5784	?++	0	0	1	0.9948
chr12:40340404	t	c	0	0	0	0	3.0639	0.8598	0.0003658	??+	0	0	0	1
chr12:40293626	a	g	0.9998	0	0.9998	1	-0.6166	0.5077	0.2246	?+-	0	0.663	1	0.4155
chr1:65392613	a	c	1	0	1	1	7.8559	62.8723	0.9006	??+	0	0	0	1
chr14:22876078	c	g	0	0	0	0	-0.0017	1.1923	0.9989	??-	0	0	0	1
chr14:22876722	c	g	0.9998	0	0.9997	0.9999	-0.4615	0.2736	0.09159	---	0	0.584	2	0.7466
chr1:155237453	t	c	1	1.00E-04	0.9996	1	-1.3115	0.2852	4.26E-06	---	0	0.219	2	0.8961
chr12:40359345	t	c	0.9999	0	0.9999	1	0.0619	0.734	0.9328	?-+	75.6	4.1	1	0.04288
chr12:40315266	a	t	1	0	1	1	1.0004	84.7888	0.9906	??+	0	0	0	1
chr1:175406219	t	c	0.0051	3.00E-04	0.0049	0.0067	0.0744	0.0577	0.1975	#ERROR!	20	2.501	2	0.2864
chr1:16990276	c	g	0.9992	1.00E-04	0.9991	0.9993	-0.2509	0.178	0.1585	#ERROR!	50	3.998	2	0.1355
chr15:61868675	t	c	0.9999	1.00E-04	0.9996	1	0.0295	0.4213	0.9442	#ERROR!	0	0.394	2	0.8212
chr15:89326688	a	g	1	0	1	1	-0.2102	0.4985	0.6734	?+-	2.9	1.03	1	0.3101
chr3:132456751	t	c	0.9998	0	0.9998	0.9999	-0.6378	0.2976	0.03208	-+-	0	1.655	2	0.4371
chr22:32479132	c	g	0.9999	0	0.9999	1	-0.5452	0.6617	0.4099	?--	0	0.301	1	0.583
chr15:62012166	t	c	1.00E-04	0	0	1.00E-04	-0.197	1.1906	0.8686	-+-	0	1.289	2	0.525
chr1:20644551	a	g	1	0	1	1	1.1238	2.9823	0.7063	?++	0	0	1	0.9908
chr15:89323426	c	g	6.00E-04	0	5.00E-04	6.00E-04	0.0719	0.1826	0.6936	?++	0	0.012	1	0.9119
chr1:20649062	a	g	1	0	1	1	1.0074	19.151	0.958	??+	0	0	0	1
chr12:40294909	t	c	1	0	1	1	1.0403	22.3005	0.9628	??+	0	0	0	1
chr20:5111588	a	g	0.9999	0	0.9999	0.9999	1.2503	1.1712	0.2857	??+	0	0	0	1
chr1:65366050	a	t	7.00E-04	1.00E-04	2.00E-04	7.00E-04	0.2137	0.1567	0.1727	#ERROR!	0	0.263	2	0.8766
chr22:32475369	a	g	1	0	1	1	2.9257	5.1791	0.5721	??+	0	0	0	1
chr3:184327433	t	c	0.9999	0	0.9999	0.9999	-0.2641	0.4161	0.5257	-+-	0	1.095	2	0.5784
chr1:20638080	t	c	0.9999	1.00E-04	0.9995	0.9999	-0.5657	0.2552	0.02661	#ERROR!	45.8	3.692	2	0.1579
chr3:184323209	t	g	0.9998	1.00E-04	0.9996	0.9999	-0.1922	0.3277	0.5576	#ERROR!	0	0.312	2	0.8556
chr12:40249843	c	g	3.00E-04	1.00E-04	1.00E-04	4.00E-04	0.3607	0.3168	0.2548	-++	59.4	4.923	2	0.08532
chr15:89319318	a	c	6.00E-04	0	6.00E-04	6.00E-04	-0.2456	0.2997	0.4124	??-	0	0	0	1
chr14:22875844	a	g	0	0	0	0	-1.0956	3.546	0.7574	?-?	0	0	0	1
chr3:132457295	t	c	0	0	0	0	-1.2164	18.3256	0.9471	??-	0	0	0	1
chr3:184322835	c	g	0	0	0	0	1.5925	0.7461	0.03281	?-+	47.3	1.897	1	0.1684
chr6:162443384	a	g	1	0	1	1	-15.4471	4.1196	0.0001771	?-+	93.5	15.412	1	8.64E-05
chr15:62023788	t	c	1	0	1	1	0.1859	1.1765	0.8744	?++	12.6	1.144	1	0.2849
chr15:89320850	a	c	1.00E-04	0	0	1.00E-04	-0.0432	0.5853	0.9411	?-+	11	1.124	1	0.2892
chr1:16996298	c	g	0.9986	3.00E-04	0.9981	0.9988	-0.0636	0.1196	0.5945	-+-	0	1.963	2	0.3747
chr14:22877421	a	c	4.00E-04	2.00E-04	3.00E-04	7.00E-04	-0.1163	0.2249	0.6049	--+	25.8	2.695	2	0.2599
chr12:40310435	a	g	1	0	1	1	-3.762	0.5793	8.34E-11	?+-	0	0.988	1	0.3202
chr1:20644639	a	g	1	0	1	1	4.0204	15.7455	0.7985	??+	0	0	0	1
chr6:161973347	a	g	1	0	1	1	1.3948	10.7667	0.8969	??+	0	0	0	1
chr12:40293644	a	g	0	0	0	0	-1.0092	17.497	0.954	??-	0	0	0	1
chr15:89323445	t	c	1	0	1	1	-1.5472	0.8125	0.05688	??-	0	0	0	1
chr14:22877070	a	g	0.9917	5.00E-04	0.991	0.994	-0.0521	0.0458	0.2545	-+-	0	0.622	2	0.7328
chr22:38116155	t	c	1	0	1	1	1.0146	6.037	0.8665	??+	0	0	0	1
chr15:61983818	a	g	0	0	0	0	-1.0087	15.7547	0.9489	??-	0	0	0	1
chr12:40321114	a	g	0	0	0	0	-1.0153	10.8227	0.9253	??-	0	0	0	1
chr1:16997044	t	c	0.9998	1.00E-04	0.9997	0.9999	0.5457	0.5248	0.2985	?++	0	0.067	1	0.7954
chr2:232791059	c	g	0.9999	0	0.9999	1	0.1099	0.7047	0.8761	?-+	0	0.676	1	0.4108
chr22:32475067	t	c	1	0	1	1	-13.4881	3.8439	0.0004499	?-+	37.4	1.597	1	0.2063
chr22:38140069	t	c	0.9999	0	0.9998	0.9999	-0.0725	0.3447	0.8335	-+-	0	1.953	2	0.3767
chr1:20633766	t	c	1	0	1	1	0.5789	2.5949	0.8235	??+	0	0	0	1

chr15:89321773	t	g	1	0	1	1	1.0034	19.5642	0.9591	??+	0	0	0	1
chr12:40298433	c	g	1.00E-04	0	0	1.00E-04	-0.2073	1.0793	0.8477	?--	0	0.041	1	0.8392
chr3:184322631	t	c	0.9999	0	0.9999	0.9999	-0.121	0.6522	0.8528	#ERROR!	0	1.153	2	0.5619
chr6:161785793	c	g	0	0	0	0	-1.0138	14.3637	0.9437	??-	0	0	0	1
chr15:89322800	a	g	1	0	1	1	0.1792	1.5744	0.9094	?-+	0	0.225	1	0.6354
chr2:232844499	c	g	1	0	1	1	1.3153	17.4126	0.9398	??+	0	0	0	1
chr15:89322748	t	c	1	0	1	1	3.0313	6.2221	0.6261	??+	0	0	0	1
chr12:40363541	a	g	0.9999	0	0.9999	1	1.2117	1.1649	0.2983	?++	0	0.013	1	0.9108
chr14:22875639	t	c	0.9998	1.00E-04	0.9995	0.9999	-0.0634	0.4892	0.897	#ERROR!	71.2	6.949	2	0.03097
chr3:184322583	c	g	0.9985	1.00E-04	0.9983	0.9985	-0.0846	0.1126	0.4522	#ERROR!	11.1	2.25	2	0.3246
chr1:16986334	t	c	1	0	1	1	-0.5677	0.9929	0.5675	?-+	42.5	1.739	1	0.1873
chr1:155235772	a	c	1	0	0.9999	1	-1.4297	0.3784	0.0001581	-?-	0	0.27	1	0.6032
chr22:38140006	a	g	0.9997	0	0.9997	0.9999	-0.115	0.2349	0.6244	#ERROR!	0	0.75	2	0.6874
chrX:121049176	t	g	0.0229	0	0.0229	0.0229	0.0182	0.0237	0.443	??+	0	0	0	1
chr12:40322037	t	c	1	0	1	1	4.0454	10.7821	0.7075	??+	0	0	0	1
chr1:20644651	t	c	0.9998	0	0.9998	1	-0.2626	0.3621	0.4684	?--	95.2	20.906	1	4.82E-06
chr2:232811255	t	c	1	0	1	1	-0.0048	1.1319	0.9966	??-	0	0	0	1
chr3:132499777	a	g	0.9893	6.00E-04	0.9883	0.9896	-0.0477	0.0426	0.2626	---	0	0.662	2	0.7184
chr15:61910283	t	c	0.5241	0.0082	0.4815	0.5257	0.0076	0.0091	0.4049	-?+	82.1	5.585	1	0.01812
chr15:89325456	c	g	1	0	1	1	-0.3414	0.9369	0.7155	?+-	0	0.536	1	0.4641
chr15:89323460	c	g	0.0018	1.00E-04	0.0017	0.002	0.0135	0.0965	0.8891	--+	0	1.146	2	0.5637
chr14:22876113	a	g	0.9998	0	0.9998	0.9999	-0.4381	0.276	0.1124	#ERROR!	9.5	2.21	2	0.3313
chr3:132474976	a	g	0	0	0	0	0.581	0.6807	0.3934	?-+	0	0.509	1	0.4757
chr12:40278179	a	g	1.00E-04	0	1.00E-04	1.00E-04	-0.3481	0.4061	0.3914	---	0	1.227	2	0.5415
chr12:40251480	a	g	1	0	1	1	7.5454	60.9371	0.9015	??+	0	0	0	1
chr15:61941806	t	c	0	0	0	0	1.7761	0.625	0.004488	??+	0	0	0	1
chr15:89333177	t	c	1	0	1	1	-2.048	2.9581	0.4887	??-	0	0	0	1
chr15:61919368	c	g	1	0	1	1	2.7571	39.7987	0.9448	??+	0	0	0	1
chr21:32678644	a	c	1	0	1	1	1.0053	17.0612	0.953	??+	0	0	0	1
chr15:61962390	t	g	1.00E-04	1.00E-04	0	2.00E-04	1.8849	1.1079	0.08889	#ERROR!	58	4.757	2	0.0927
chr21:32639773	a	g	1	1.00E-04	0.9999	1	1.1568	2.2072	0.6002	#ERROR!	0	0.001	1	0.981
chr22:38143150	a	g	0.9999	0	0.9999	0.9999	0.456	0.4841	0.3462	#ERROR!	0	0.078	2	0.9617
chr5:1414780	a	g	1	0	1	1	0.2138	0.4248	0.6148	?++	0	0.195	1	0.659
chr1:155236399	t	g	1	0	1	1	2.0147	11.3938	0.8596	??+	0	0	0	1
chr21:32643457	t	c	1	0	1	1	289.6988	1156.6463	0.8022	??+	0	0	0	1
chr3:132522950	t	g	8.00E-04	1.00E-04	6.00E-04	9.00E-04	-0.1716	0.1748	0.3261	-+-	29.4	2.835	2	0.2424
chr1:20649054	a	g	1	0	1	1	2.0399	6.3052	0.7463	??+	0	0	0	1
chr15:89318986	a	g	1	0	1	1	1.436	2.1319	0.5006	?++	0	0.01	1	0.9204
chr22:38123197	a	g	1	0	1	1	1.0101	8.341	0.9036	??+	0	0	0	1
chr6:161785805	t	c	0.9999	1.00E-04	0.9999	1	-0.4164	0.6625	0.5297	?--	0	0.021	1	0.8851
chr14:22877184	a	g	1	0	1	1	-0.4737	1.0387	0.6484	?+-	0	0.583	1	0.4453
chr12:40335031	a	g	0.9999	1.00E-04	0.9998	0.9999	-0.0929	0.2926	0.7508	-+-	0	1.537	2	0.4637
chr15:61920571	t	c	0.0038	5.00E-04	0.0029	0.0051	0.068	0.0691	0.3253	#ERROR!	0	0.424	2	0.8091
chr6:161548861	t	c	1	0	1	1	2.0652	6.9857	0.7675	??+	0	0	0	1
chr1:20639934	a	g	1	0	1	1	1.0011	49.7781	0.984	??+	0	0	0	1
chr1:20644564	a	c	1	0	1	1	4.2014	8.9692	0.6395	??+	0	0	0	1
chr22:32498414	a	g	0.9992	2.00E-04	0.9986	0.9993	-0.2085	0.1681	0.215	---	48.9	3.911	2	0.1415
chr1:17000272	t	c	0.9831	7.00E-04	0.9807	0.9845	0.0385	0.0318	0.2267	#ERROR!	0	0.558	2	0.7565
chr22:38145597	t	g	1	0	1	1	-0.8619	2.3093	0.709	??-	0	0	0	1
chr1:155235819	t	c	1	0	1	1	3.1189	16.9108	0.8537	??+	0	0	0	1
chr12:40299125	a	g	0	0	0	0	-1.0111	7.209	0.8885	??-	0	0	0	1
chr1:155236269	t	c	1	0	1	1	2.4885	18.8954	0.8952	??+	0	0	0	1
chr15:61983936	t	c	0	0	0	0	-2.8022	3.0891	0.3643	??-	0	0	0	1

chr20:5069288	t	c	0.9989	3.00E-04	0.9986	0.9998	-0.0268	0.1657	0.8717	--+	29.9	2.853	2	0.2401
chr1:17000107	t	c	0.9999	0	0.9999	1	1.3035	1.246	0.2955	?++	0	0.089	1	0.7658
chr6:162443357	a	g	1	0	1	1	1.2445	2.0193	0.5377	?++	0	0.004	1	0.9524
chr3:132522832	a	g	1	0	1	1	-0.4586	1.525	0.7636	?-?	0	0	0	1
chr15:61931104	c	g	0.0195	0.0012	0.0176	0.0218	0.0237	0.0303	0.4357	#ERROR!	16.6	2.397	2	0.3016
chr15:61918208	t	c	3.00E-04	0	2.00E-04	3.00E-04	-0.0822	0.2686	0.7597	?-+	73.9	3.83	1	0.05036
chr1:155235765	t	g	0	0	0	0	-1.0029	31.0018	0.9742	??-	0	0	0	1
chr15:61984980	t	g	1	0	1	1	-1.8029	1.0387	0.08262	??-	0	0	0	1
chr1:20644549	a	g	0.9999	0	0.9998	0.9999	-0.9333	0.3734	0.01244	?+-	74.7	3.954	1	0.04675
chr1:16991787	a	g	1	0	1	1	3.0237	7.735	0.6959	??+	0	0	0	1
chr22:38112534	c	g	0	0	0	0	6.765	2.8425	0.01731	?+-	61.5	2.597	1	0.107
chr1:65386886	t	c	3.00E-04	0	2.00E-04	3.00E-04	0.0375	0.256	0.8837	#ERROR!	20.5	2.515	2	0.2844
chr1:155235197	c	g	1	0	1	1	1.0961	3.5391	0.7568	?+?	0	0	0	1
chr22:38112547	a	g	1	0	1	1	-3.0093	1.0378	0.003736	?+-	19.2	1.237	1	0.2661
chr15:62010533	a	g	0.9998	0	0.9998	1	0.032	0.4518	0.9435	#ERROR!	0	1.317	2	0.5177
chr15:61991096	t	c	0.0011	2.00E-04	0.001	0.0015	-0.2371	0.1399	0.09005	?--	0	0.015	1	0.9015
chr22:32498466	a	g	0	0	0	0	-1.3082	1.7856	0.4638	?--	0	0.019	1	0.8907
chr12:40308481	a	g	0.9996	2.00E-04	0.9993	0.9998	-0.4147	0.1985	0.0367	---	35.4	3.097	2	0.2125
chr1:65385740	a	g	0.9994	1.00E-04	0.999	0.9997	-0.1236	0.1992	0.5349	-+-	0	1.417	2	0.4923
chr15:89333357	t	c	1.00E-04	0	1.00E-04	1.00E-04	0.3001	0.6202	0.6285	?+-	0	0.427	1	0.5134
chr15:89320944	t	g	0	0	0	0	-1.0194	8.3498	0.9028	??-	0	0	0	1
chr3:184323468	c	g	0.9985	3.00E-04	0.9981	0.9987	-0.027	0.1193	0.8207	#ERROR!	8.1	2.177	2	0.3367
chr22:38113621	t	c	0.9993	1.00E-04	0.9989	0.9995	-0.1036	0.1845	0.5743	-+-	0	0.141	2	0.932
chr21:32701973	a	g	1	0	1	1	1.363	2.2628	0.5469	?+?	0	0	0	1
chr22:38132881	t	c	0.9871	5.00E-04	0.9852	0.9878	-0.0659	0.037	0.0749	-+-	18.8	2.463	2	0.2919
chr22:32498453	t	c	1	0	1	1	-0.8959	0.989	0.365	?+-	0	0.912	1	0.3396
chr12:40294866	t	g	1	0	1	1	1.3753	2.5467	0.5892	?++	0	0.073	1	0.7868
chr12:40323270	t	g	1	0	1	1	1.0043	13.3421	0.94	??+	0	0	0	1
chr2:232794852	t	c	1	0	1	1	1.2689	2.1885	0.562	?++	0	0.015	1	0.9037
chr21:32664963	a	c	0.9995	3.00E-04	0.9991	0.9999	-0.0503	0.1909	0.7922	#ERROR!	30.3	2.87	2	0.2381
chr1:17000495	t	c	1	0	0.9999	1	-3.2543	1.2522	0.009355	?--	92.4	13.177	1	0.0002834
chr2:74530433	c	g	0	0	0	0	1.1087	3.2807	0.7354	?+-	0	0.001	1	0.9705
chr12:40319998	t	c	0	0	0	0	-1.0057	17.1927	0.9534	??-	0	0	0	1
chr6:161548937	a	g	1	0	1	1	-1.3294	0.8999	0.1396	??-	0	0	0	1
chr15:61936716	a	c	0	0	0	0	-3.7122	8.4965	0.6622	??-	0	0	0	1
chr1:7965399	a	g	0.9999	0	0.9999	0.9999	-0.4169	0.5649	0.4605	??-	0	0	0	1
chr15:61977090	t	g	0	0	0	0	-232.4395	924.9281	0.8016	??-	0	0	0	1
chr15:89327006	c	g	1.00E-04	0	0	1.00E-04	-0.5974	0.8704	0.4925	?--	0	0.158	1	0.6906
chr22:38120802	t	c	1	0	1	1	-2.1372	1.0038	0.03325	??-	0	0	0	1
chr3:184327628	t	c	1	0	1	1	1.2632	1.4562	0.3857	?+?	0	0	0	1
chr15:89333723	t	c	0.9995	1.00E-04	0.9993	0.9995	0.1363	0.197	0.489	#ERROR!	0	0.928	2	0.6289
chr6:161569358	c	g	6.00E-04	3.00E-04	3.00E-04	9.00E-04	-0.3665	0.2281	0.108	#ERROR!	0	0.651	2	0.7222
chr15:89318736	t	c	1	0	1	1	3.028	8.6096	0.7251	??+	0	0	0	1
chr1:20648577	t	c	1	0	1	1	1.0096	9.4643	0.915	??+	0	0	0	1
chr1:155239633	a	g	1	0	1	1	-7.3931	1.6965	1.31E-05	?-+	0	0.306	1	0.5801
chr22:38115619	t	c	1	0	1	1	-2.7228	1.2573	0.03035	??-	0	0	0	1
chr14:22877106	a	g	1	0	1	1	1.2518	1.768	0.4789	?+?	0	0	0	1
chr1:155235196	a	g	0.9998	1.00E-04	0.9995	0.9999	-1.3347	0.1818	2.15E-13	---	0	0.814	2	0.6655
chr15:61856407	t	c	0.9996	1.00E-04	0.9995	0.9997	0.5909	0.2631	0.02473	#ERROR!	0	0.031	2	0.9847
chr22:32484066	a	g	1.00E-04	0	0	1.00E-04	0.2071	0.7335	0.7777	?+-	0	0.606	1	0.4364
chr12:40308507	t	c	1	0	1	1	-2.7031	1.1107	0.01495	??-	0	0	0	1
chr15:61882609	a	g	3.00E-04	1.00E-04	2.00E-04	4.00E-04	-0.6305	0.3169	0.04666	---	0	0.323	2	0.8507
chr12:40293552	a	c	0	0	0	0	-0.3988	2.4885	0.8727	??-	0	0	0	1

chr22:38132917	t	c	0.9999	0	0.9999	1	-0.8343	0.7157	0.2438	---	48.5	3.883	2	0.1435
chr3:132528316	t	g	0.0045	1.00E-04	0.0039	0.0047	-0.1108	0.0626	0.07663	---	0	0.239	2	0.8874
chr2:232819840	a	g	0.9999	0	0.9999	0.9999	0.8227	0.4691	0.07944	?++	0	0.387	1	0.5339
chr22:38169318	a	g	0.9999	0	0.9999	0.9999	0.1348	0.8132	0.8683	?-+	12.9	1.148	1	0.284
chr12:40263875	a	g	0	0	0	0	-4.0637	12.574	0.7466	??-	0	0	0	1
chr3:132450732	a	t	2.00E-04	0	1.00E-04	2.00E-04	0.4593	0.4157	0.2692	-++	38.9	3.271	2	0.1949
chr1:16986554	a	g	0.9994	1.00E-04	0.9993	0.9999	0.14	0.1733	0.4191	#ERROR!	0	1.712	2	0.4248
chr14:22875363	a	g	6.00E-04	0	6.00E-04	6.00E-04	0.2157	0.1938	0.2657	#ERROR!	0	0.057	1	0.811
chr15:89318676	a	g	0	0	0	0	-1.0069	8.4938	0.9056	??-	0	0	0	1
chr5:1394741	c	g	4.00E-04	1.00E-04	3.00E-04	5.00E-04	0.3905	0.2128	0.06654	-++	0	1.307	2	0.5201
chr15:89321223	t	c	0	0	0	0	-1.0056	9.1414	0.9124	??-	0	0	0	1
chr15:89330257	a	g	1	0	1	1	-1.9418	1.0046	0.05326	?+-	40.1	1.67	1	0.1963
chr1:20648528	a	g	0.9997	1.00E-04	0.9995	0.9998	-0.0257	0.3871	0.947	--+	37.8	3.214	2	0.2005
chr22:38112541	a	g	1	0	1	1	2.0142	8.0174	0.8016	??+	0	0	0	1
chr15:89320907	t	c	0	0	0	0	-1.0017	24.0871	0.9668	??-	0	0	0	1
chr14:22875648	a	g	0.9998	1.00E-04	0.9997	0.9999	0.033	0.438	0.9399	#ERROR!	0	0.226	2	0.8932
chr1:155236331	t	c	1	0	1	1	1.0012	47.3356	0.9831	??+	0	0	0	1
chr1:17004708	a	g	1	0	1	1	1.1469	1.8134	0.5271	?+?	0	0	0	1
chr3:132466351	a	c	4.00E-04	0	1.00E-04	4.00E-04	-0.0413	0.2186	0.8503	---	0	0.977	2	0.6136
chr3:132523168	a	c	1.00E-04	0	0	1.00E-04	-0.122	0.6607	0.8535	?--	0	0.23	1	0.6318
chr21:32656885	a	g	0	0	0	0	0.7098	0.9257	0.4432	?-+	0	0.22	1	0.6393
chr1:155238209	t	g	1	0	1	1	2.0121	11.798	0.8646	??+	0	0	0	1
chr15:61922557	a	g	0.001	2.00E-04	8.00E-04	0.0012	0.091	0.144	0.5272	#ERROR!	75.6	8.192	2	0.01664
chr15:89320998	t	c	1	0	1	1	1.0034	24.4712	0.9673	??+	0	0	0	1
chr12:40257264	a	g	0	0	0	0	-3.0209	16.0095	0.8503	??-	0	0	0	1
chr22:38132914	a	g	0	0	0	0	-1.0136	7.2849	0.8893	??-	0	0	0	1
chr3:184327694	a	g	0	0	0	0	-1.2675	1.5867	0.4244	?--	0	0.415	1	0.5193
chr16:46682118	a	g	1	0	1	1	1.0099	14.8247	0.9457	??+	0	0	0	1
chr1:155235195	t	c	1	0	1	1	-1.2765	1.0522	0.2251	??-	0	0	0	1
chr1:155235006	a	g	1	0	1	1	2.4268	6.4604	0.7072	??+	0	0	0	1
chr15:62008730	a	g	4.00E-04	2.00E-04	1.00E-04	6.00E-04	-0.0881	0.2991	0.7684	?-+	64.7	2.833	1	0.09235
chr3:184317393	t	c	1	0	1	1	0.8399	7.2896	0.9083	??+	0	0	0	1
chr22:38169423	t	g	1	0	1	1	2.0281	7.2346	0.7792	??+	0	0	0	1
chr12:40363410	a	g	4.00E-04	0	4.00E-04	4.00E-04	-0.5517	0.7162	0.4411	??-	0	0	0	1
chr4:41263258	t	c	0	0	0	0	1.9221	6.3723	0.7629	??+	0	0	0	1
chr15:89316763	a	c	0.92	7.00E-04	0.9196	0.9214	0.0085	0.0155	0.5824	?++	0	0	1	0.985
chr15:61922408	t	c	0.9217	0.0015	0.921	0.925	-0.0153	0.0153	0.3156	-+-	0	0.801	2	0.6699
chr6:161360187	t	c	0	0	0	0	-1.0159	13.4152	0.9396	??-	0	0	0	1
chr6:162262681	t	c	1	0	1	1	1.2476	2.0655	0.5458	?++	0	0.015	1	0.9031
chr15:62013943	a	g	0	0	0	0	0.5555	1.6751	0.7402	??+	0	0	0	1
chr12:40263861	a	g	1.00E-04	0	0	1.00E-04	0.1955	0.8294	0.8136	?+-	0	0.211	1	0.646
chr15:89321780	a	g	0.9998	1.00E-04	0.9996	1	1.0457	0.6899	0.1296	#ERROR!	0	1.071	2	0.5855
chr15:89318553	t	c	0	0	0	0	-2.0189	7.3601	0.7838	??-	0	0	0	1
chr22:38132850	a	g	1	0	1	1	-0.8467	0.8902	0.3416	?--	0	0.109	1	0.7418
chr17:46010389	t	c	1	0	1	1	1.0076	4.6318	0.8278	??+	0	0	0	1
chr15:62035002	a	c	1	0	1	1	4.4843	21.2982	0.8332	??+	0	0	0	1
chr1:155235823	t	c	1	0	0.9999	1	-1.0836	1.6382	0.5083	?-+	7.2	1.078	1	0.2992
chr1:16987157	t	c	0.9998	0	0.9998	1	-0.4094	0.3617	0.2577	?+-	0	0.098	1	0.7541
chr15:61922680	t	g	0.9999	0	0.9999	0.9999	0.2437	0.5471	0.656	?+-	0	0.765	1	0.3817
chr1:16986065	t	c	0.9998	1.00E-04	0.9996	0.9998	0.1111	0.3464	0.7484	-+-	8.6	2.189	2	0.3347
chr1:20644626	c	g	0	0	0	0	-1.9449	19.7909	0.9217	??-	0	0	0	1
chr22:38123244	a	t	0	0	0	0	-1.0088	9.2563	0.9132	??-	0	0	0	1
chr15:61915865	c	g	1	0	1	1	51.3025	346.2031	0.8822	??+	0	0	0	1

chr21:32726855	t	c	0	0	0	0	-0.3548	3.2139	0.9121	??-	0	0	0	1
chr15:61940726	a	g	1	0	1	1	0.7642	3.7288	0.8376	?++	0	0.007	1	0.9324
chr3:184322551	t	c	0.9999	1.00E-04	0.9998	1	0.6865	0.9579	0.4736	#ERROR!	0	0.252	1	0.6154
chr3:184323412	c	g	0.9997	1.00E-04	0.9996	0.9998	-0.1903	0.282	0.4997	---	0	0.159	2	0.9236
chr3:132516758	a	g	1	0	1	1	3.6261	5.893	0.5383	??+	0	0	0	1
chr1:16997107	a	g	0.9999	0	0.9999	0.9999	-1.2117	0.4258	0.004436	??-	0	0	0	1
chr12:40351579	t	c	1	0	1	1	3.0164	15.5502	0.8462	??+	0	0	0	1
chr15:89333327	a	g	1	0	1	1	-0.4201	0.9793	0.668	?+-	0	0.369	1	0.5434
chr22:38126451	t	c	0	0	0	0	0.6159	1.0472	0.5565	??+	0	0	0	1
chr1:16986291	t	c	0.9998	0	0.9998	0.9999	0.3255	0.5913	0.582	-++	0	0.846	2	0.6552
chr15:61978646	t	c	8.00E-04	1.00E-04	0	0.0011	-0.2008	0.1438	0.1625	---	0	0.454	2	0.7968
chr15:89320856	t	c	1	0	1	1	1.0103	8.6477	0.907	??+	0	0	0	1
chr1:155238214	a	c	0	0	0	0	-4.035	23.9945	0.8665	??-	0	0	0	1
chr1:17000429	t	c	0	0	0	0	-5.9513	26.5081	0.8224	??-	0	0	0	1
chr6:161785778	a	c	0	0	0	0	-1.0049	21.7842	0.9632	??-	0	0	0	1
chr12:40310515	a	g	0	0	0	0	-1.2259	3.1269	0.695	?--	0	0.019	1	0.8895
chr15:61922527	a	g	0.9996	1.00E-04	0.9996	0.9998	0.3994	0.2984	0.1807	?++	0	0.108	1	0.7425
chr3:132499231	t	c	0.9814	5.00E-04	0.9812	0.9835	0.0463	0.0308	0.1334	#ERROR!	0	0.689	2	0.7087
chr15:61966088	t	c	0.9999	1.00E-04	0.9998	0.9999	-0.1215	0.8164	0.8817	#ERROR!	0	0.254	1	0.6144
chr15:89323462	t	c	3.00E-04	1.00E-04	3.00E-04	5.00E-04	-0.0402	0.2885	0.8892	#ERROR!	0	0.317	2	0.8535
chr12:40232383	a	g	0	0	0	0	-1.3899	2.5682	0.5884	?--	0	0.145	1	0.7032
chr1:155238144	a	g	0	0	0	0	-1.079	6.3329	0.8647	??-	0	0	0	1
chr1:20644627	t	c	1	0	1	1	3.0584	16.3996	0.8521	??+	0	0	0	1
chr1:155238246	a	g	1	0	1	1	-1.4708	2.1458	0.4931	?-?	0	0	0	1
chr15:89330133	c	g	0.0046	2.00E-04	0.0044	0.0057	0.0434	0.0635	0.4942	#ERROR!	64.4	5.613	2	0.0604
chr1:16992115	t	c	1	0	1	1	0.7871	16.424	0.9618	??+	0	0	0	1
chr6:161785825	t	c	1.00E-04	0	0	1.00E-04	0.6828	0.9905	0.4906	?+-	0	0.136	1	0.7123
chr15:61940651	a	g	1.00E-04	0	0	1.00E-04	-0.7947	1.6731	0.6348	-?+	0	0.202	1	0.6532
chr2:232794843	a	c	0.9806	3.00E-04	0.9793	0.9807	0.0272	0.0332	0.4126	#ERROR!	0	0.015	1	0.9036
chr1:16990191	t	c	1	0	1	1	0.9838	1.5439	0.524	?++	0	0.008	1	0.9285
chr12:40340380	t	c	0	0	0	0	-1.0061	13.68	0.9414	??-	0	0	0	1
chr21:32631012	a	c	1	0	1	1	-1.1777	1.0707	0.2714	??-	0	0	0	1
chr1:155237444	a	g	0	0	0	0	-0.5033	10.2471	0.9608	??-	0	0	0	1
chr12:40309225	a	c	0	0	0	0	-1.0028	31.4954	0.9746	??-	0	0	0	1
chr1:155239736	a	g	1	0	1	1	1.2187	2.6078	0.6402	?+?	0	0	0	1
chr3:184323541	t	c	1	0	1	1	-0.391	1.0132	0.6996	??-	0	0	0	1
chr1:7965425	c	g	1	0	1	1	1.0046	24.7863	0.9677	??+	0	0	0	1
chr15:89328532	c	g	0.9986	6.00E-04	0.9976	0.999	0.0971	0.103	0.3459	#ERROR!	0	0.229	2	0.8919
chr12:40302860	a	g	1	0	1	1	1.5104	10.5293	0.8859	??+	0	0	0	1
chr15:62023836	t	c	0.9779	8.00E-04	0.9778	0.9827	0.029	0.0313	0.3549	-?+	0	0.276	1	0.5991
chr12:40232385	t	c	0	0	0	0	-1.3903	2.5682	0.5883	?--	0	0.145	1	0.7032
chr1:20649134	a	g	1	0	0.9999	1	0.0352	0.756	0.9629	?++	0	0.001	1	0.9806
chr1:7984981	t	c	0	0	0	0	-1.004	24.2849	0.967	??-	0	0	0	1
chr2:74532831	a	g	1	0	1	1	4.0914	3.755	0.2759	??+	0	0	0	1
chr14:22877128	t	g	0.9999	0	0.9999	0.9999	0.4884	2.5293	0.8469	??+	0	0	0	1
chr3:132503289	a	g	1	0	1	1	-0.4757	3.219	0.8825	??-	0	0	0	1
chr15:61934224	t	g	0.0081	1.00E-04	0.0081	0.0087	-0.0258	0.0515	0.6173	#ERROR!	0	0.069	1	0.7931
chr5:1403013	a	g	0.9985	5.00E-04	0.998	0.9995	-0.3927	0.1408	0.005296	---	63.9	5.534	2	0.06286
chr12:40323255	a	g	0	0	0	0	-1.0044	17.8501	0.9551	??-	0	0	0	1
chr2:232794782	c	g	0.9997	1.00E-04	0.9996	0.9999	-0.3973	0.3477	0.2533	?--	0	0.086	1	0.7687
chr6:161360129	t	g	1	0	1	1	2.0235	7.653	0.7915	??+	0	0	0	1
chr15:89318737	a	g	1	0	1	1	-2.0087	1.9446	0.3016	?-+	0	0.789	1	0.3745
chr15:61854543	t	c	0.0055	1.00E-04	0.0051	0.0056	-0.0619	0.0573	0.2794	#ERROR!	0	0.634	2	0.7282

chr15:89319073	a	g	8.00E-04	1.00E-04	6.00E-04	0.001	-0.0055	0.1519	0.9712	#ERROR!	59.9	4.992	2	0.08242
chr12:40340400	a	g	0.9993	4.00E-04	0.9965	0.9995	-2.4648	0.0588	0.00E+00	---	19.1	2.472	2	0.2905
chr15:89318962	t	c	1	0	1	1	3.0287	8.2286	0.7128	??+	0	0	0	1
chr3:132454135	t	c	1	0	0.9999	1	-0.6657	0.7986	0.4046	-+-	40.1	3.339	2	0.1884
chr15:89325591	a	g	1.00E-04	0	0	1.00E-04	-0.7827	0.8011	0.3286	---	0	0.304	2	0.859
chr15:62044256	t	c	0.9999	0	0.9999	0.9999	-0.0925	0.7742	0.9049	?+-	0	0.382	1	0.5364
chr15:89321777	a	g	1	0	1	1	4.0562	6.8335	0.5528	??+	0	0	0	1
chr22:38132979	a	t	0	0	0	0	-1.006	18.4437	0.9565	??-	0	0	0	1
chr21:32657057	a	g	1	0	1	1	33.9605	126.9081	0.789	??+	0	0	0	1
chr1:175386035	t	c	8.00E-04	3.00E-04	5.00E-04	0.0012	-0.2244	0.1956	0.2513	---	0	0.954	2	0.6207
chr1:155235829	a	c	1	0	1	1	1.0116	6.9784	0.8847	??+	0	0	0	1
chr15:61967438	t	c	0.5796	0.033	0.4083	0.586	-0.0098	0.0092	0.284	#ERROR!	71.4	3.499	1	0.06142
chr15:89323451	t	c	2.00E-04	0	0	2.00E-04	0.3344	0.4234	0.4296	?-+	0	0.522	1	0.47
chr15:89330258	c	g	6.00E-04	1.00E-04	5.00E-04	9.00E-04	0.1244	0.1754	0.478	#ERROR!	0	0.085	2	0.9583
chr2:232832882	a	g	0.9998	1.00E-04	0.9997	0.9999	-0.4367	0.3433	0.2034	?+-	3.8	1.039	1	0.308
chr17:46024061	t	c	1	0	1	1	-1.4102	0.7317	0.05395	?+-	0	0.688	1	0.407
chr1:155236426	a	g	1	0	1	1	2.0351	13.0021	0.8756	??+	0	0	0	1
chr1:155236277	a	g	1	0	1	1	1.1592	2.7071	0.6685	?++	0	0	1	0.9874
chr1:16986208	t	c	1	0	1	1	306.324	991.8091	0.7574	??+	0	0	0	1
chr12:40274666	a	t	0	0	0	0	-2.0389	6.447	0.7518	??-	0	0	0	1
chr15:61910215	a	g	1	0	1	1	2.0202	11.0427	0.8548	??+	0	0	0	1
chr3:132502298	t	c	1	0	1	1	0.7091	1.0047	0.4803	?++	0	0.204	1	0.6515
chr1:155238570	c	g	1.00E-04	0	1.00E-04	1.00E-04	1.1863	0.2163	4.14E-08	?++	0	0.182	1	0.6695
chr3:132528231	a	g	0.9999	0	0.9999	1	1.2734	1.2764	0.3184	?++	0	0.094	1	0.7593
chr15:62010558	c	g	1	0	1	1	-1.3985	0.5809	0.01607	?+-	8.4	1.092	1	0.2961
chr12:40263806	a	g	2.00E-04	1.00E-04	1.00E-04	6.00E-04	-0.2449	0.287	0.3936	#ERROR!	33.1	1.495	1	0.2215
chr1:155236409	c	g	0	0	0	0	4.614	1.7052	0.006812	?+-	0	0.113	1	0.7363
chr1:155235708	c	g	1	0	1	1	-0.3209	0.4911	0.5134	??-	0	0	0	1
chr3:132496652	a	g	1	0	1	1	3.0501	5.8787	0.6039	??+	0	0	0	1
chr1:155240048	t	c	1	0	1	1	2.0143	11.86	0.8651	??+	0	0	0	1
chr12:40240594	c	g	0.9999	0	0.9998	0.9999	-0.1104	0.581	0.8493	--+	71.9	7.109	2	0.02859
chr15:61890400	t	c	0.9998	0	0.9998	0.9999	0.7835	0.4393	0.07454	?-+	0	0.94	1	0.3322
chr15:89319234	a	g	0.9998	0	0.9998	0.9999	0.6128	0.4543	0.1773	#ERROR!	0	1.198	2	0.5493
chr15:89319053	c	g	1.00E-04	0	0	1.00E-04	0.6705	0.5827	0.2499	?-+	0	0.516	1	0.4728
chr3:184325887	a	g	0	0	0	0	-1.0147	6.7922	0.8812	??-	0	0	0	1
chr5:176621218	t	g	0.9995	1.00E-04	0.9994	0.9998	-0.2231	0.3125	0.4753	---	0	1.163	2	0.5592
chr12:40293624	c	g	0.9999	1.00E-04	0.9996	0.9999	0.2377	0.4086	0.5608	-++	37.2	3.185	2	0.2034
chr2:74530208	t	c	1	0	1	1	0.4112	2.6617	0.8772	??+	0	0	0	1
chr15:61916007	c	g	0.9998	1.00E-04	0.9996	0.9999	0.1506	0.3894	0.6989	#ERROR!	2.7	2.055	2	0.3578
chr22:32479135	t	g	2.00E-04	1.00E-04	2.00E-04	3.00E-04	-0.0906	0.3693	0.8061	-+-	58.9	4.867	2	0.08775
chr15:89325562	a	g	1	0	1	1	-0.2215	0.7257	0.7601	??-	0	0	0	1
chr6:161785820	a	g	0.9973	5.00E-04	0.9961	0.9975	-0.2617	0.0639	4.20E-05	#ERROR!	79.6	9.825	2	0.007353
chr22:38113561	a	g	1	0	1	1	-1.3514	0.9292	0.1459	?+-	65.5	2.897	1	0.08873
chr12:40363526	a	g	1	0	0.9999	1	-0.6093	0.6788	0.3694	-?-	33.4	1.502	1	0.2204
chr5:122450987	a	g	0.9998	1.00E-04	0.9995	1	0.1174	0.3654	0.7481	-++	0	1.023	2	0.5996
chr20:5100832	a	g	0.9979	1.00E-04	0.9975	0.998	0.1481	0.1249	0.2358	-++	0	0.15	2	0.9278
chr22:38128349	a	g	0.9997	1.00E-04	0.9996	0.9999	0.4487	0.3718	0.2275	?+-	45	1.817	1	0.1777
chr22:38135029	a	g	0.9999	0	0.9999	1	0.0953	0.7406	0.8976	?++	0	0.004	1	0.9476
chr15:61962754	a	t	1	0	1	1	1.0065	12.1544	0.934	??+	0	0	0	1
chr15:89323423	a	g	0	0	0	0	-2.0818	6.4583	0.7472	??-	0	0	0	1
chr1:7961737	a	t	0.9999	0	0.9999	0.9999	1.0354	0.9674	0.2845	??+	0	0	0	1
chr6:162443429	t	c	1	0	1	1	12.4801	17.6616	0.4798	??+	0	0	0	1
chr1:20644662	a	g	0.9999	0	0.9999	1	-0.7266	0.9011	0.4201	?-+	84.2	6.338	1	0.01182

chr3:184327401	a	g	0.9997	0	0.9997	0.9998	-0.2867	0.3001	0.3394	--+	32.1	2.945	2	0.2294
chr1:7985019	a	g	0.9994	1.00E-04	0.9993	0.9996	-0.3631	0.189	0.05466	---	0	0.533	2	0.7659
chr3:132484650	t	c	0.9994	1.00E-04	0.999	0.9994	-0.0645	0.1815	0.7221	#ERROR!	0	0.459	2	0.7948
chr12:40305946	a	t	1	0	0.9999	1	0.0358	0.8385	0.966	?-+	0	0.926	1	0.336
chr6:161350125	t	g	1.00E-04	0	1.00E-04	1.00E-04	0.305	0.673	0.6505	?-+	0	0.747	1	0.3874
chr1:155237438	t	c	0.9998	1.00E-04	0.9997	0.9998	-0.9062	0.2394	0.0001534	---	5.9	2.125	2	0.3456
chr12:40302866	a	g	0	0	0	0	0.0041	1.1692	0.9972	?-+	0	0.39	1	0.5321
chr1:65385756	a	g	1	0	0.9999	1	1.0389	1.7487	0.5525	?++	0	0.028	1	0.8675
chr15:61936649	c	g	0	0	0	0	0.8751	0.8824	0.3213	?++	21.4	1.273	1	0.2592
chr1:20649217	t	c	0.9999	0	0.9999	0.9999	-0.0056	0.5754	0.9922	?+-	0	0.003	1	0.9547
chr15:89320857	a	g	1	0	1	1	2.0154	7.5463	0.7894	??+	0	0	0	1
chr22:38120867	t	g	0	0	0	0	1.6161	0.7115	0.02313	??+	0	0	0	1
chr3:184327230	a	g	0.9999	1.00E-04	0.9999	1	1.3286	1.6466	0.4197	#ERROR!	0	0	1	0.9828
chr1:20648601	a	g	1	0	1	1	3.0137	13.0794	0.8178	??+	0	0	0	1
chr6:162443383	t	c	0.9998	0	0.9998	1	1.2097	0.6818	0.07604	#ERROR!	0	0.278	2	0.8702
chr15:61984004	a	g	1	0	1	1	1.0997	3.4858	0.7524	?+?	0	0	0	1
chr6:161973335	t	c	0.9999	1.00E-04	0.9998	0.9999	0.1602	0.6947	0.8176	#ERROR!	15.9	2.377	2	0.3047
chr1:20639911	t	c	1	0	1	1	1.0007	62.4851	0.9872	??+	0	0	0	1
chr12:40235634	t	c	0.0024	1.00E-04	0.0022	0.0025	0.0165	0.0857	0.8474	#ERROR!	0	1.069	2	0.5859
chr6:161360169	a	g	0.9984	2.00E-04	0.9976	0.9985	-0.1324	0.1134	0.2428	#ERROR!	0	1.052	2	0.591
chr1:20645675	a	g	1	0	1	1	3.0334	6.5724	0.6444	??+	0	0	0	1
chr14:22873373	t	c	0.9999	0	0.9999	1	-2.1633	1.0986	0.04895	--?	0	0	1	0.9857
chr15:61880871	a	g	0.9998	0	0.9998	0.9999	-0.0679	0.3615	0.8509	#ERROR!	0	1.14	2	0.5656
chr15:50586433	a	g	0.8946	3.00E-04	0.8939	0.8947	-0.0138	0.0134	0.3039	---	0	0.113	2	0.9449
chr22:32493191	a	g	0.9999	0	0.9999	0.9999	-0.2841	0.4577	0.5348	?--	0	0.032	1	0.8582
chr3:132494190	a	g	0.0064	9.00E-04	0.0047	0.0076	-0.0699	0.0671	0.2976	-+-	0	1.482	2	0.4767
chr15:61911844	a	g	0.9967	2.00E-04	0.9964	0.997	-0.0585	0.074	0.4294	#ERROR!	48.4	3.879	2	0.1438
chr12:40304004	a	g	0	0	0	0	-1.0116	11.7554	0.9314	??-	0	0	0	1
chr3:184321516	a	g	3.00E-04	0	3.00E-04	4.00E-04	0.1957	0.25	0.4337	-?+	50.7	2.029	1	0.1543
chr15:62008659	a	g	1	0	1	1	2.0393	11.7127	0.8618	??+	0	0	0	1
chr3:132478139	a	g	0.9972	4.00E-04	0.9966	0.9974	-0.0375	0.088	0.6701	--+	28.1	2.782	2	0.2489
chr3:132502295	t	c	0.9711	9.00E-04	0.9683	0.9727	0.0373	0.0253	0.1406	-++	0	0.396	2	0.8203
chr6:162443314	a	t	0	0	0	0	0.4559	0.5769	0.4294	?-+	0	0.07	1	0.7913
chr15:61917535	t	c	1	0	1	1	14.1204	47.4719	0.7661	??+	0	0	0	1
chr15:61854888	a	c	0.9986	2.00E-04	0.9982	0.9989	-0.0011	0.1255	0.993	-++	0	1.206	2	0.5473
chr15:61915655	t	c	7.00E-04	0	5.00E-04	7.00E-04	-0.0552	0.1961	0.7783	#ERROR!	0	0.578	1	0.447
chr12:40240543	t	c	0.9998	0	0.9998	0.9998	0.111	0.3308	0.7371	??+	0	0	0	1
chr15:89319031	a	g	0	0	0	0	-1.0103	6.612	0.8786	??-	0	0	0	1
chr1:155238629	t	c	1	0	1	1	1.3999	1.5686	0.3722	?++	0	0.011	1	0.9157
chr1:16988161	a	t	0.9982	1.00E-04	0.9981	0.9988	0.0082	0.1251	0.9476	--+	61.2	5.153	2	0.07602
chr12:40367050	a	g	0	0	0	0	-1.0045	24.9028	0.9678	??-	0	0	0	1
chr15:61984885	a	t	3.00E-04	1.00E-04	1.00E-04	4.00E-04	0.0382	0.3282	0.9073	#ERROR!	63.2	5.437	2	0.06597
chr1:16992042	a	g	0	0	0	0	0.2191	0.8293	0.7916	??+	0	0	0	1
chr16:46671766	t	c	0	0	0	0	-1.0048	24.0369	0.9667	??-	0	0	0	1
chr3:184327622	t	c	0.0239	0.0012	0.0212	0.0262	-0.0239	0.0275	0.3839	#ERROR!	0	1.801	2	0.4063
chr2:74531688	a	g	1	0	1	1	2.0577	6.5341	0.7528	??+	0	0	0	1
chr2:232844373	c	g	0.002	1.00E-04	0.0012	0.002	-0.1218	0.1153	0.2909	--+	66.6	5.985	2	0.05016
chr12:40298346	a	g	1	0	1	1	1.0127	4.9847	0.839	??+	0	0	0	1
chr15:89320917	t	c	1	0	1	1	1.0105	7.5103	0.893	??+	0	0	0	1
chr15:89321842	t	c	0.0098	4.00E-04	0.0084	0.0104	-0.0286	0.0434	0.5106	-+-	0	0.316	2	0.8537
chr20:5069330	t	c	0.9999	0	0.9999	0.9999	4.3065	4.7921	0.3688	??+	0	0	0	1
chr22:38169255	t	c	0.9998	0	0.9998	0.9998	0.322	0.4349	0.4591	-?+	0	0.254	1	0.6146
chr1:155238251	t	g	1	0	1	1	1.159	2.9077	0.6902	?+?	0	0	0	1

chr15:89333427	a	g	0.9999	0	0.9999	0.9999	-0.9448	0.7566	0.2118	??-	0	0	0	1
chr1:155239716	c	g	0	0	0	0	2.3258	1.0473	0.02636	??+	0	0	0	1
chr14:22877108	a	g	0.9999	1.00E-04	0.9996	0.9999	-0.8901	0.3306	0.007091	---	0	1.56	2	0.4584
chr12:40320103	a	g	2.00E-04	1.00E-04	1.00E-04	4.00E-04	0.1429	0.3771	0.7048	--+	25.2	2.673	2	0.2627
chr15:62023837	a	g	0.9999	0	0.9999	0.9999	-0.1624	0.6478	0.802	?--	0	0.027	1	0.8692
chr1:155235777	a	t	1	0	1	1	-2.0605	1.0492	0.04955	??-	0	0	0	1
chr22:32475378	a	g	0.9003	5.00E-04	0.8981	0.9012	0.0137	0.0139	0.3252	--+	0	1.844	2	0.3977
chr22:38126417	a	g	0.9998	0	0.9998	0.9998	0.4335	0.3976	0.2755	-++	15.8	2.374	2	0.3051
chr15:61927138	a	g	0.9868	5.00E-04	0.9859	0.987	0.047	0.0375	0.2103	-++	0	0.988	2	0.6102
chr12:40310561	a	g	1	0	1	1	3.0279	12.3969	0.807	??+	0	0	0	1
chr15:89325639	a	g	0.9975	2.00E-04	0.9966	0.9977	0.0307	0.0849	0.7176	--+	0	0.293	2	0.8639
chr1:16986091	a	g	0.9946	9.00E-04	0.9933	0.9959	0.111	0.0666	0.09528	#ERROR!	0	1.142	2	0.5649
chr1:16986246	a	g	0.9999	0	0.9999	0.9999	-0.8716	1.0536	0.4081	--?	0	0.895	1	0.3442
chr6:162262627	a	g	1	0	1	1	3.0624	11.9294	0.7974	??+	0	0	0	1
chr1:155237370	a	g	1	0	1	1	1.1618	1.7228	0.5001	?++	0	0.004	1	0.947
chr1:155235780	a	g	1	0	1	1	-5.2675	3.1789	0.09752	??-	0	0	0	1
chr2:232790836	c	g	0.9998	0	0.9998	0.9999	-0.1109	0.3461	0.7485	-+-	0	0.813	2	0.666
chr15:89321792	t	c	0.9996	1.00E-04	0.9996	0.9999	-0.1062	0.3261	0.7446	#ERROR!	4.4	2.092	2	0.3514
chr1:155236246	a	g	0.993	3.00E-04	0.9914	0.9931	-0.3887	0.0424	4.87E-20	---	0	0.393	2	0.8218
chr1:20637888	t	c	1	0	1	1	1.3496	1.7882	0.4504	?++	0	0.064	1	0.801
chr12:40364843	a	g	1	0	1	1	2.032	12.539	0.8713	??+	0	0	0	1
chr3:132507256	a	g	0.0611	0.0031	0.0542	0.0658	0.0085	0.0173	0.625	#ERROR!	0	0.838	2	0.6577
chr15:61945803	a	g	0.9971	1.00E-04	0.9965	0.9971	0.009	0.0807	0.9108	#ERROR!	0	0.642	2	0.7256
chr22:38115667	a	g	1	0	1	1	1.1742	2.8148	0.6766	?+?	0	0	0	1
chr12:40251273	a	g	0.999	2.00E-04	0.9989	0.9998	-0.3183	0.1307	0.01492	#ERROR!	0	0.836	2	0.6584
chr15:61954471	t	g	0.001	1.00E-04	9.00E-04	0.0014	-0.0769	0.1472	0.6016	---	0	0.115	2	0.9443
chr15:61880845	a	g	1	0	1	1	-0.5955	1.2151	0.6241	?-+	0	0.243	1	0.6218
chrX:155260942	t	g	1	0	1	1	1.013	8.0068	0.8993	??+	0	0	0	1
chr1:17005754	a	g	0.9998	0	0.9998	0.9998	-0.4535	0.6619	0.4933	?-?	0	0	0	1
chr1:20649109	t	c	0.9999	0	0.9999	1	1.2789	1.3834	0.3552	#ERROR!	0	0.238	2	0.8878
chr3:184321987	a	c	0.9986	0	0.9986	0.9986	0.3329	0.2184	0.1275	??+	0	0	0	1
chr16:46662405	a	c	0	0	0	0	-1.1275	3.936	0.7745	?--	0	0.083	1	0.7737
chr15:61963904	t	c	0.9996	0	0.9995	0.9998	-0.0909	0.2101	0.6654	-+-	55.1	4.454	2	0.1079
chr15:61920268	t	c	0.9979	2.00E-04	0.9976	0.998	0.1301	0.0994	0.1908	#ERROR!	0	1.968	2	0.3737
chr15:62023783	t	c	7.00E-04	1.00E-04	6.00E-04	0.0011	-0.1163	0.1745	0.505	---	0	1.951	2	0.3769
chr15:89333267	a	g	0.9995	0	0.9995	0.9995	0.2421	0.3077	0.4315	??+	0	0	0	1
chr3:132511169	c	g	0.9984	2.00E-04	0.998	0.9985	-0.15	0.1065	0.1591	---	21.6	2.551	2	0.2793
chr6:161350208	t	c	0.9998	0	0.9996	0.9998	-0.6149	0.1902	0.001228	#ERROR!	0	1.418	2	0.4922
chr15:89318587	a	g	0.9998	0	0.9998	0.9998	0.0078	0.3236	0.9809	#ERROR!	0	0.967	2	0.6166
chr12:40274671	t	c	0	0	0	0	-1.0184	12.1836	0.9334	??-	0	0	0	1
chr15:61984953	a	g	0.9999	0	0.9999	0.9999	0.7418	0.7298	0.3094	?++	0	0.121	1	0.7275
chr1:155239948	a	g	1	0	1	1	2.0218	9.6506	0.8341	??+	0	0	0	1
chr1:65401812	t	c	1	0	1	1	-9.4672	3.3512	0.004727	?-+	65.4	2.888	1	0.08924
chr15:89324193	t	c	0.9997	1.00E-04	0.9996	0.9998	0.0815	0.3702	0.8258	#ERROR!	52.1	4.174	2	0.124
chr15:61969413	t	c	0.0049	3.00E-04	0.0042	0.005	-0.0851	0.0641	0.1844	#ERROR!	0	1.684	2	0.4309
chr1:155235814	t	c	1	0	1	1	1.172	2.827	0.6785	?+?	0	0	0	1
chr15:89327004	t	g	2.00E-04	1.00E-04	1.00E-04	3.00E-04	0.4359	0.3403	0.2002	#ERROR!	3.1	2.064	2	0.3562
chr12:40304000	a	g	0.9999	0	0.9999	0.9999	-0.6874	0.4292	0.1093	---	4.2	2.087	2	0.3523
chr22:38126371	a	g	0.9997	1.00E-04	0.9996	0.9998	0.2182	0.3481	0.5307	-++	0	1.298	2	0.5226
chr21:32688348	t	c	1	0	1	1	1.3066	1.7509	0.4555	?++	0	0.031	1	0.8604
chr12:40364975	a	c	1	0	1	1	1.2284	2.1352	0.5651	?++	0	0.056	1	0.813
chr1:155239933	t	c	1	0	1	1	1.0706	3.9645	0.7871	?++	0	0	1	0.9981
chr12:40299255	t	c	1.00E-04	0	1.00E-04	1.00E-04	-5.0384	13.4722	0.7084	??-	0	0	0	1

chr15:61961592	t	c	0.0784	8.00E-04	0.0766	0.0788	0.0142	0.0153	0.3517	#ERROR!	0	0.87	2	0.6473
chr22:38116200	a	g	1	0	1	1	1.0006	41.5475	0.9808	??+	0	0	0	1
chr12:40251361	a	g	0	0	0	0	-1.0012	47.6553	0.9832	??-	0	0	0	1
chr12:40299212	a	g	0.9999	0	0.9999	1	-0.0253	0.7021	0.9712	?+-	0	0.007	1	0.9332
chr3:132538225	a	g	0.9965	2.00E-04	0.9961	0.9966	0.1337	0.0769	0.082	#ERROR!	0	0.001	2	0.9997
chr15:89333364	a	g	4.00E-04	1.00E-04	3.00E-04	7.00E-04	0.215	0.2382	0.3667	#ERROR!	0	1.164	2	0.5588
chr21:32688306	t	c	1	0	1	1	3.4156	22.8546	0.8812	??+	0	0	0	1
chr3:132523636	c	g	0.9989	1.00E-04	0.9985	0.999	-0.0828	0.1259	0.5107	#ERROR!	64.7	5.665	2	0.05887
chr15:61920099	t	c	0.9928	5.00E-04	0.992	0.9938	0.0429	0.0496	0.3867	-++	0	0.405	2	0.8167
chr3:184331762	t	c	0.9998	0	0.9998	0.9998	0.331	0.4282	0.4395	--+	0	1.296	2	0.5232
chr2:232839964	a	g	0.9993	2.00E-04	0.9986	0.9994	0.0282	0.2089	0.8926	#ERROR!	0	0.217	2	0.8974
chr3:132492416	a	g	1	0	1	1	1.2736	2.5814	0.6217	?++	0	0.008	1	0.9296
chr6:161350211	t	c	1	0	1	1	0.1535	1.016	0.8799	??+	0	0	0	1
chr1:16986097	a	g	0.9414	0.0019	0.9385	0.9496	0.0096	0.0177	0.5877	#ERROR!	0	0.252	2	0.8814
chr2:232791432	t	c	0.9999	0	0.9999	0.9999	0.2001	0.4755	0.6739	#ERROR!	0	0.706	2	0.7024
chr1:7984971	a	g	1	0	1	1	1.0124	8.0065	0.8994	??+	0	0	0	1
chr14:22877045	t	c	1	0	0.9998	1	1.3497	3.095	0.6628	?++	0	0.109	1	0.741
chr3:184321497	t	c	1	0	1	1	-4.7625	2.4246	0.0495	?-+	0	0.248	1	0.6187
chr1:65386820	a	g	0.9999	0	0.9998	0.9999	-0.4713	0.6864	0.4923	?--	0	0.02	1	0.8868
chr15:61907289	t	c	0	0	0	0	-31.5342	41.3794	0.446	??-	0	0	0	1
chr15:61890334	a	g	1	0	1	1	1.2696	2.4461	0.6037	?+?	0	0	0	1
chr1:155241085	t	c	1	0	1	1	1.0017	40.6027	0.9803	??+	0	0	0	1
chr22:38135061	a	c	0	0	0	0	-1.0005	72.5719	0.989	??-	0	0	0	1
chr14:22876816	a	g	0.9992	1.00E-04	0.9989	1	0.1993	0.1562	0.202	#ERROR!	0	0.27	2	0.8737
chr14:22876765	t	g	0.9999	0	0.9999	1	-0.6382	0.3887	0.1006	?--	88	8.358	1	0.003839
chr6:161350187	a	g	0.9975	7.00E-04	0.9963	0.9979	0.054	0.0763	0.4797	#ERROR!	0	1.751	2	0.4167
chr22:38115588	t	g	0	0	0	0	-2.0244	8.7899	0.8178	??-	0	0	0	1
chr22:38116119	a	c	0.9998	0	0.9998	1	-0.7834	0.3941	0.04684	#ERROR!	30.7	2.884	2	0.2364
chr1:155238596	a	c	1	0	1	1	5.8218	17.5244	0.7397	??+	0	0	0	1
chr22:38143219	c	g	2.00E-04	0	1.00E-04	2.00E-04	0.0889	0.3142	0.7772	--+	4	2.083	2	0.3529
chr1:155236276	t	c	1	0	1	1	-2.6054	1.2536	0.03768	??-	0	0	0	1
chr15:61856408	a	g	0.9994	2.00E-04	0.9991	0.9999	-0.1508	0.222	0.497	#ERROR!	0	1.724	2	0.4224
chr3:132492497	a	g	5.00E-04	3.00E-04	3.00E-04	0.0012	0.0194	0.2525	0.9387	-+-	1.9	2.039	2	0.3607
chr22:38126374	t	c	0.9995	1.00E-04	0.9994	0.9997	0.1753	0.2221	0.4301	-++	0	0.095	2	0.9536
chr15:61983969	a	g	1	0	1	1	3.0102	14.8268	0.8391	??+	0	0	0	1
chr20:5109429	a	g	0.0014	1.00E-04	0.0013	0.0016	-0.1395	0.1603	0.3841	--+	32.9	2.98	2	0.2254
chr22:38132922	t	c	1	0	1	1	1.4803	2.6067	0.5701	?++	0	0.071	1	0.7893
chr6:161973401	t	c	1	0	1	1	1.4965	3.5853	0.6764	?++	0	0.019	1	0.8894
chr1:155237576	a	t	0	0	0	0	0.8201	0.9789	0.4022	?-+	0	0.959	1	0.3273
chr15:89321242	a	c	1	0	1	1	1.008	14.2965	0.9438	??+	0	0	0	1
chr12:40356126	a	t	0	0	0	0	1.8633	1.3934	0.1811	??+	0	0	0	1
chr3:132499168	a	g	0.9998	0	0.9996	0.9998	-0.0952	0.365	0.7941	--+	45.9	3.695	2	0.1576
chr3:132467277	a	g	0.9998	0	0.9998	0.9998	-0.0342	0.3117	0.9127	?+-	40.1	1.67	1	0.1963
chr3:184319745	a	g	0.4096	0.4885	0.0025	0.9974	-0.0395	0.0905	0.6626	---	0	1.218	2	0.5439
chr22:38169240	t	c	1.00E-04	0	1.00E-04	1.00E-04	-0.7182	0.8046	0.372	??-	0	0	0	1

Supplementary Table 8: Meta-analysis with annotations, statistics, penetrance, power

NOTE: Variants with wide confidence intervals indicate a strong involvement in dominantlly inherited PD but their statistics should be interpreted with caution.

MarkerName	avsnp150	VariantName	Ref	Alt	Gene	P-value	OR	L95	U95	CLNDN	PD_known	CLNSIG	QC_pass_23anc	Genotyped_vs_	ImputationR2	max_credible_A	max_credible_A	Penetrance_L95	Penetrance_est	Penetrance_U95	Power_at_alpha005_OR2
chr1:20638104	rs74315360	PINK1_A217D	C	A	PINK1	0.9565	2.740937458	5.21E-16	1.44E+16	Parkinson_disea	PD_known	Pathogenic	FALSE	G	NA	NA	0	NA	NA	NA	0.050529558
chr22:38112571	rs772143897	PLA2G6_D737Y	C	A	PLA2G6	0.6088	1.420345284	0.370436959	5.445948839	.	Not_PD_known	.	FALSE	I	0.383233339	NA	1.67E-06	NA	NA	NA	0.272770382
chr3:195867623	rs112384084	TNK2_R877H	C	T	TNK2	0.7225	1.011161834	0.950993549	1.075136898	Parkinson_disea	PD_known	Conflicting_interpretations_of_pathogenicity	TRUE	I	0.947827697	0.003056978	0.000295	0.026465823	0.028950159	0.031665168	1
chr1:155235727	rs1064651	GBA1_D448H	C	G	GBA1	0.6289	1.213974879	0.553078132	2.664605456	.	Not_PD_known	.	TRUE	I	0.648898244	1.11E-05	1.67E-06	0.016079901	0.045236159	0.12725096	0.353915975
chr15:89318617	rs56047213	POLG_E1136K	C	T	POLG	0.08167	55.21308653	0.603841218	5048.487639	not_provided	Not_PD_known	Likely_pathogenic	FALSE	G	NA	0	0	0.02406471	0.26903056	1	0.053712059
chr2:232791124	rs148277228	GIGYF2_D349E	T	A	GIGYF2	0.4657	1.145567331	0.795131316	1.650450036	.	Not_PD_known	.	TRUE	I	0.620631397	0.000195378	1.00E-05	0.005463566	0.015354851	0.043146488	0.96133828
chr1:155238258	rs374591570	GBA1_L213F	G	A	GBA1	0.8879	7.614847805	4.21E-12	1.38E+13	Gaucher_disease	Not_PD_known	Conflicting_interpretations_of_pathogenicity	FALSE	G	NA	NA	0	0	0	1	0.053906761
chr6:161785877	rs150562946	PRKN_R256C	G	A	PRKN	0.1059	1.303691688	0.945312234	1.797937186	Neoplasm_of_ov	PD_known	Uncertain_significance	TRUE	I	0.641049981	NA	1.33E-05	NA	NA	NA	0.993708479
chr12:40322386	rs111910483	LRRK2_L1795F	G	T	LRRK2	1.40E-05	2.48655943	1.648536624	3.750585646	Parkinson_disea	PD_known	Uncertain_significance	TRUE	I	0.849218488	5.18E-06	1.67E-06	0.022229774	0.096589956	0.419552636	0.300880431
chr15:89333621	rs201016638	POLG_Q45R	T	C	POLG	0.7279	1.165324979	0.492228618	2.758844686	Seizures|Progres	Not_PD_known	Benign/Likely_benign	TRUE	I	0.911483765	7.10E-06	1.67E-06	0.003414024	0.070461751	1	0.317798678
chr1:65386873	rs144323705	DNAJC6_V296M	G	A	DNAJC6	0.03866	5.069286105	1.088281667	23.61306121	.	Not_PD_known	.	TRUE	G	0.407384396	NA	0	NA	NA	NA	0.077535794
chr1:175406252	rs150331590	TNR_C155S	A	T	TNR	0.1747	2.951460109	0.61853098	14.08355775	Parkinson_disea	PD_known	Uncertain_significance	TRUE	G	0.397012055	0.000105573	1.67E-06	0.000760413	0.004736074	0.029495285	0.311684242
chr6:162443371	rs148990138	PRKN_P37L	G	A	PRKN	0.6559	1.093736702	0.73745073	1.622155791	Parkinson_disea	PD_known	Conflicting_interpretations_of_pathogenicity	TRUE	I	0.505675018	NA	6.67E-06	NA	NA	NA	0.821938061
chr12:40367045	rs146428335	LRRK2_R2477Q	G	A	LRRK2	0.8395	1.087085215	0.484514461	2.43904849	Parkinson_disea	PD_known	Uncertain_significance	TRUE	I	0.639142752	4.56E-05	3.33E-06	0.005250703	0.021932051	0.09159495	0.402108395
chr1:20637956	rs768091663	PINK1_A168P	G	C	PINK1	0.7275	7.488735813	9.02E-05	621716.8233	PINK1-Related_F	PD_known	Likely_pathogenic	TRUE	G	NA	NA	0	NA	NA	NA	0.090271839
chr1:16986292	rs201610681	ATP13A2_A1057V	G	A	ATP13A2	0.6951	1.328565781	0.320937782	5.499779497	Kufor-Rakeb_syr	Not_PD_known	Uncertain_significance	FALSE	I	NA	NA	3.33E-06	NA	NA	NA	0.344825568
chr1:65392454	rs145329294	DNAJC6_C441S	T	A	DNAJC6	0.9012	1.020915731	0.735930201	1.416260575	Parkinson_disea	PD_known	Conflicting_interpretations_of_pathogenicity	TRUE	I	0.819304228	NA	1.17E-05	NA	NA	NA	0.979681835
chr1:16987187	rs148201608	ATP13A2_T937M	G	A	ATP13A2	0.258	24.19388705	0.096789187	6047.619485	Kufor-Rakeb_syr	Not_PD_known	Conflicting_interpretations_of_pathogenicity	FALSE	I	0.311943769	NA	1.67E-06	NA	NA	NA	0.218528123
chr3:184328741	rs144059151	EIF4G1_M1356T	T	C	EIF4G1	0.9283	1.01999732	0.661942887	1.571728548	.	Not_PD_known	.	TRUE	I	0.689064145	0.000144086	1.00E-05	0.009618593	0.020820848	0.045062545	0.887157546
chr12:40351723	rs35658131	LRRK2_Y2189C	A	G	LRRK2	0.8778	1.046551005	0.585983293	1.869113025	Parkinson_disea	PD_known	Uncertain_significance	TRUE	I	0.966629863	5.70E-05	3.33E-06	0.003616845	0.017546505	0.085110244	0.635652369
chr15:89318595	rs2307441	POLG_E1143G	T	C	POLG	0.1154	1.032311022	0.992238279	1.074002152	Association_with	Not_PD_known	Benign	TRUE	I	0.973517239	0.008237358	0.000718333	0.024802532	0.026161302	0.02759276	1
chr3:184322040	rs112545306	EIF4G1_P486S	C	T	EIF4G1	0.4745	1.104397569	0.841349608	1.449687477	.	Not_PD_known	.	TRUE	I	0.910621524	0.000250035	2.00E-05	0.01419033	0.023996631	0.040573129	0.999193846
chr12:40251346	rs141262110	LRRK2_T358M	C	T	LRRK2	0.9273	1.025725329	0.59413067	1.770843523	Parkinson_disea	PD_known	Conflicting_interpretations_of_pathogenicity	TRUE	I	0.741862357	7.86E-05	5.00E-06	0.005429893	0.019073879	0.066991112	0.746671996
chr22:38132952	rs149653983	PLA2G6_T319M	G	A	PLA2G6	0.4617	1.163578302	0.777347341	1.741711064	Parkinson_disea	PD_known	Uncertain_significance	TRUE	I	0.793139219	NA	8.33E-06	NA	NA	NA	0.92094843
chr15:61929659	rs149882066	VPS13C_A2043G	G	C	VPS13C	0.0482	18.10883607	1.023106922	320.5236293	not_provided	Not_PD_known	Likely_benign	FALSE	I	0.310837477	NA	0	NA	NA	NA	0.090997926
chr1:155237458	rs367968666	GBA1_H294Q	A	C	GBA1	0.9691	1.019895326	0.376453781	2.763118685	.	Not_PD_known	.	TRUE	I	0.592274368	0	0	0.068930823	0.47775124	1	0.050035682
chr12:40257283	rs568593066	LRRK2_H442Y	C	T	LRRK2	0.9774	12.55476155	1.14E-75	1.38E+77	.	Not_PD_known	.	FALSE	I	0.76905781	NA	0	0	NA	1	0.051889805
chr14:22875400	rs201144028	LRP10_R151H	G	A	LRP10	0.06333	4.597901483	0.918754804	23.01016328	.	Not_PD_known	.	FALSE	I	0.448353797	1.03E-05	3.33E-06	0.003038112	0.097491673	1	0.702909033
chr3:132516425	rs142901736	DNAJC13_R1830H	G	A	DNAJC13	0.01227	5.660430786	1.457595266	21.98173761	.	Not_PD_known	.	TRUE	I	0.641307473	NA	NA	NA	NA	NA	0.108737208
chr1:20633840	rs575668171	PINK1_R98W	C	T	PINK1	0.8781	7.455112212	5.34E-11	1.04E+12	Parkinson_disea	PD_known	Likely_pathogenic	FALSE	G	NA	NA	0	NA	NA	NA	0.079765556
chr15:61983908	rs557609723	VPS13C_K609I	T	A	VPS13C	0.8483	1.177860904	0.220144308	6.302031237	.	Not_PD_known	.	TRUE	I	0.626975238	NA	1.67E-06	NA	NA	NA	0.227224783
chr1:20637894	rs138050841	PINK1_R147H	G	A	PINK1	0.5334	4.095136295	0.048477282	345.9381494	.	Not_PD_known	.	FALSE	G	NA	NA	0	NA	NA	NA	0.138543914
chr15:62008671	rs150098264	VPS13C_T368A	T	C	VPS13C	0.4812	1.165791202	0.760721465	1.786552884	not_specified	Not_PD_known	Uncertain_significance	TRUE	I	0.757018685	NA	8.33E-06	NA	NA	NA	0.774086939
chr15:89321776	rs796052888	POLG_R853Q	C	T	POLG	0.9113	2.753850186	5.02E-08	151139226.5	Progressive_scle	Not_PD_known	Pathogenic/Likely_pathogenic	TRUE	G	NA	NA	0	0	0	1	0.051482399
chr1:20644570	rs146691996	PINK1_P286L	C	T	PINK1	0.6327	4.691723505	0.008279141	2658.762319	not_provided	Not_PD_known	Uncertain_significance	TRUE	G	NA	NA	0	NA	NA	NA	0.106749819
chr6:161973403	rs137853060	PRKN_K211N	T	A	PRKN	0.006756	4.479448786	1.513528985	13.2574015	Parkinson_disea	PD_known	Pathogenic	TRUE	G	NA	NA	0	NA	NA	NA	0.110478515
chr6:162443438	rs532703934	PRKN_V15M	C	T	PRKN	0.9499	2.599710259	2.92E-13	2.32E+13	.	Not_PD_known	.	FALSE	G	NA	NA	0	NA	NA	NA	0.051930521
chr3:184320694	rs34838305	EIF4G1_R201H	G	A	EIF4G1	0.5485	1.164043826	0.708804168	1.911667695	.	Not_PD_known	.	TRUE	I	0.659146845	0.000237096	6.67E-06	0.002748309	0.008435404	0.025886702	0.694572298
chr4:41261921	rs139583787	UCHL1_R153W	C	T	UCHL1	0.1732	4.015251558	0.542988032	29.6917135	Parkinson_disea	PD_known	Uncertain_significance	FALSE	I	0.366497189	9.50E-06	8.33E-06	0.015802077	0.26319758	1	0.638478422
chr3:184321882	rs145998921	EIF4G1_A433V	C	T	EIF4G1	0.3491	1.617691285	0.591051574	4.427574867	.	Not_PD_known	.	TRUE	I	0.731602073	1.54E-05	1.67E-06	0.001951074	0.032497224	0.541170131	0.230325789
chr22:38120889	rs370691849	PLA2G6_R538C	G	A	PLA2G6	0.557	4.320624707	0.032707986	570.7412759	Iron_accumulatic	Not_PD_known	Pathogenic	TRUE	G	NA	NA	0	NA	NA	NA	0.121776928
chr12:40284011	rs35173587	LRRK2_R793M	G	T	LRRK2	0.0322	1.550379904	1.037991919	2.315699959	Parkinson_disea	PD_known	Conflicting_interpretations_of_pathogenicity	TRUE	I	0.814714134	0.000142987	1.17E-05	0.015584185	0.024477684	0.038444203	0.983153161
chr15:89330081	rs141367015	POLG_Q285H	C	G	POLG	0.5611	2.010734343	0.190667539	21.20472427	Progressive_scle	Not_PD_known	Uncertain_significance	TRUE	G	NA	5.78E-05	1.67E-06	0.00134246	0.008643716	0.055649952	0.181095322
chr1:155236384	rs76539814	GBA1_T362I	G	A	GBA1	0.003759	5.488198173	1.734820587	17.36220991	Gaucher_disease	Not_PD_known	Pathogenic	FALSE	G	NA	0	0	0.024879733	0.091829895	0.338915555	0.086189529
chr1:16986321	rs533548757	ATP13A2_R1148H	C	T	ATP13A2	0.5447	3.923954711	0.04706489	327.1530159	.	Not_PD_known	.	FALSE	I	NA	NA	0	NA	NA	NA	NA
chr3:132502299	rs139620588	DNAJC13_R1516H	G	A	DNAJC13	0.5147	1.045818675	0.914066457	1.196561468	.	Not_PD_known	.	TRUE	I	0.937204778	0.000963136	6.17E-05	0.013763633	0.019208079	0.026801877	1
chr3:184321599	rs191357892	EIF4G1_E339Q	G	C	EIF4G1	0.1918	1.602716497	0.789272219	3.254517399	.	Not_PD_known	.	TRUE	I	0.669144094	8.56E-05	3.33E-06	0.002835098	0.011688697	0.048186952	0.471072304
chr15:61915890	rs35236516	VPS13C_R2730C	G	A	VPS13C	0.2176	2.173850459	0.632481695	7.4715614	not_provided	Not_PD_known	Benign	TRUE	I	0.80210501	NA	NA	NA	NA	NA	0.491222246
chr2:232794835	rs116074753	GIGYF2_N457T	A	C	GIGYF2	0.4161	1.206350878	0.767534384	1.896048531	Parkinson_disea	PD_known	risk_factor	TRUE	G	0.484703124	0.0001426	1.00E-05	0.013139901	0.02103785	0.033679402	0.9725334
chr3:132505400	rs193294938	DNAJC13_E1661D	A	T	DNAJC13	0.8256	2.332405177	0.00124635	4364.836454	.	Not_PD_known	.	FALSE	I	0.378421098	NA	0	0	0	1	0.0556136
chr1:20644665	rs139226733	PINK1_M318L	A	T	PINK1	0.8297	1.031279227	0.778900476	1.365433554	Parkinson_disea	PD_known	Uncertain_significance	TRUE	I	0.683014154	NA	1.67E-05	NA	NA	NA	0.99914329
chr12:40323256	rs35602796	LRRK2_M1869T	T	C	LRRK2	0.5092	1.155808385	0.751993198	1.776469557	Parkinson_disea	PD_known	Uncertain_significance	TRUE	I	0.913289666	9.30E-05	6.67E-06	0.009274916	0.021509242	0.049876268	0.887823842
chr15:89330250	rs202088074	POLG_V229G	A	C	POLG	0.917	1.031072992	0.579357238	1.834984437	.	Not_PD_known	.	FALSE	I	0.359030664	NA	NA	NA	NA	NA	0.986435862
chr15:89330184	rs113994094	POLG_T251I	G	A	POLG	0.5441	1.052638638	0.89179767	1.242488222	Abnormality_of_t	Not_PD_known	Conflicting_interpretations_of_pathogenicity	TRUE	I	0.999308944	0.000419501	3.83E-05	0.022077203	0.027413495	0.034037604	0.999999979
chr1:155235231	rs76071730	GBA1_H490R	T	C	GBA1	0.9538	2.743679767	3.96E-15	1.90E+15	.	Not_PD_known	.	FALSE	G	NA	NA	0	0	0	1	0.051069626
chr4:41261759	rs150601238	UCHL1_M124L	A	C	UCHL1	0.9013	1.014707101	0.805977059	1.277493557	Parkinson_disea	PD_known	Conflicting_interpretations_of_pathogenicity	TRUE	I	0.726760685	0.000462059	2.50E-05	0.009490554	0.016231677	0.027756561	0.999960757
chr22:38133007	rs367854265	PLA2G6_R301C	G	A	PLA2G6	0.13	2.008322909	0.814419247	4.952438096	not_provided	Not_PD_known	Uncertain_significance	TRUE	I	0.503189445	NA	3.33E-06	NA	NA	NA	0.428246003
chr15:61915712	rs72747885	VPS13C_I2789T	A	G	VPS13C	0.1505	1.060987417	0.978678585	1.150218587	not_provided	Not_PD_known	Benign	TRUE	I	0.964622259	NA	0.000166667	NA	NA	NA	1
chr12:40243556	rs28365216	LRRK2_N238I	A	T	LRRK2	0.01552	9.434753006	1.532186806	58.09641744	Parkinson_disea	PD_known	Uncertain_significance	FALSE	G	NA	0	0	0.056545092	0.606276409	1	0.054752867
chr5:1432618	rs71653633	SLC6A3_L167F	G	A	SLC6A3	0.9142	1.013287505	0.796844282	1.288522225	Tobacco_addictic	PD_known	Conflicting_interpretations_of_pathogenicity	TRUE	I	0.841640949	0.00033645	2.17E-05	0.010767349	0.019319341	0.034658217	0.999849142
chr15:89318641	rs755544706	POLG_R1128C	G	A	POLG	0.4352	3.652456665	0.141139855	94.51929561	not_provided	Not_PD_known	Uncertain_significance	TRUE	G	NA	2.18E-05	3.33E-06	0.006043711	0.04595765	0.349443744	0.071924389
chr20:5069196	rs570474631	TMEM230_P126T	G	T	TMEM230	0.5766	1.066412274	0.850873338	1.336550445	.	Not_PD_known	.	TRUE	I	0.543975353	0.000568867	3.83E-05	0.01359082	0.02021563	0.030064871	0.999999291
chr15:62007466	rs371570548	VPS13C_I378V	T	C	VPS13C	0.8752	2.237815127	9.67E-05	51762.43644	not_provided	Not_PD_known	Likely_benign	TRUE	I	0.763122022	NA	0	NA	NA	NA	0.054775938
chr12:40320042	rs542505115	LRRK2_R1628C	C	T	LRRK2	0.2181	1085227.283	0.000268918	4.38E+15	.	Not_PD_known	.	FALSE	I	0.390015721	NA	0	0	NA	1	0.1795555
chr3:132522838	rs145242123	DNAJC13_T1895M	C	T	DNAJC13	0.5838	1.048122009	0.885711502	1.240313289	not_provided	Not_PD_known	Likely_benign	TRUE	I	0.92733115	0.000504211	4.50E-05	0.018907369	0.026774502	0.037908984	0.999999995
chr15:89328795	rs796052916	POLG_N354D	T	C	POLG	0.8795	2.751923165	5.71E-06	1325446.577	not_provided	Not_PD_known	Likely_pathogenic	TRUE	G	NA	NA	0	0	0	1	0.054370068
chr16:46674332	rs754978683	VPS35_N381S	T	C	VPS35	0.08401	2.394321758	0.88932903	6.446181887	.	Not_PD_known	.	TRUE	I	0.902914703	Inf	1.67E-06	0	0	0.054539284	0.497172404
chr15:89325466	rs781130302	POLG_V645L	C	A	POLG	0.8954	1.392499035	0.009996183	193.9794062	.	Not_PD_known	.	FALSE	I	0.385139436	NA	NA	NA	NA	NA	0.11665718
chr15:89323504	rs185645212	POLG_R722H	C	T	POLG	0.8479	1.110932775	0.378913247	3.257135083	Progressive_scle	Not_PD_known	Benign/Likely_benign	TRUE	I	0.662673652	NA	1.67E-06	0	NA	1	0.337338966
chr15:89327201	rs113994095	POLG_A467T	C	T	POLG	0.8127	1.03375727	0.785684474	1.360156817	POLG-related_cc	Not_PD_known	Pathogenic	TRUE	I	0.7051301	0.000198104	2.17E-05	0.022887875	0.032811044	0.047032703	0.999655031
chr3:132522861	rs150102948	DNAJC13_V1903I	G	A	DNAJC13	0.556	2.152005074	0.167808472	27.59768797	.	Not_PD_known	.	TRUE	I	0.621577322	NA	0	0	NA	1	0.142391971
chr22:38169326	rs147948449	PLA2G6_S34L	G	A	PLA2G6	0.9187	1.019895326	0.698120189	1.489981941	Neurodegenerati	PD_known	Uncertain_significance	TRUE	I	0.726187348	NA	1.00E-05	NA	NA	NA	0.932095525
chr1:155236444	rs77714449	GBA1_K342I	T	A	GBA1	0.9394	2.741211566	1.38E-11	5.46E+11	.	Not_PD_known	.	FALSE	G	NA	NA	0	9.22E-17	0	1	0.052574194
chr1:20649095	rs747400197	PINK1_N451S	A	G	PINK1	0.01158	1579.715427	5.193895533	480468.0443	.	Not_PD_known	.	FALSE	G	NA	NA	0	NA	NA	NA	0.107626319
chr22:32498513	rs374363283	FBXO7_R518W	C	T	FBXO7	0.7145	1.198415179	0.454386101	3.160745761	Parkinson_disea	PD_known	Uncertain_significance	TRUE	I	0.589326799	NA	3.33E-06	NA	NA	NA	0.554691401
chr12:40305893	rs56320539	LRRK2_D1296Y	G	T	LRRK2	0.9823	2.722090088	1.02E-38	7.26E+38	.	Not_PD_known	.	FALSE	G	NA	NA	0	0	0	1	0.05100776

chr15:89320953	rs121918048	POLG_H932Y	G	A	POLG	0.773	80.70495713	8.88E-12	7.34E+14	Progressive_scle	Not_PD_known	Pathogenic	FALSE	G	NA	NA	0	0	0	1	0.067582221
chr22:38112165	rs140758033	PLA2G6_P806R	G	C	PLA2G6	0.8568	1.162299069	0.226985759	5.951647072	PLA2G6-associa	Not_PD_known	Conflicting_interpretations_of_pathogenicity	TRUE	I	0.64381671	NA	0	NA	NA	NA	0.200125636
chr12:40240535	rs111655870	LRRK2_L208F	G	T	LRRK2	0.987	2.720185292	1.38E-52	5.37E+52	.	Not_PD_known	.	FALSE	I	1.000000358	NA	0	0	NA	1	0.050658085
chr21:32641895	rs765344810	NA	C	T	SYNJ1	0.9481	2.750272506	1.58E-13	4.80E+13	.	Not_PD_known	.	FALSE	G	NA	NA	0	1.19E-16	0	1	0.050545906
chr1:20633615	rs551542832	PINK1_G23S	G	A	PINK1	0.7013	2.833180692	0.013850804	579.526853	Parkinson_disea	PD_known	Conflicting_interpretations_of_pathogenicity	TRUE	I	0.743851006	NA	0	NA	NA	NA	0.080701735
chr12:40225159	rs281865040	LRRK2_E10K	G	A	LRRK2	0.9551	2.738471724	1.64E-15	4.58E+15	Parkinson_disea	PD_known	Uncertain_significance	FALSE	G	NA	NA	0	0	0	1	0.053241235
chr2:232747740	rs72554080	GIGYF2_N56S	A	G	GIGYF2	0.06159	1.680178437	0.97511877	2.895031524	Parkinson_disea	PD_known	risk_factor	TRUE	I	0.742126703	0.000114101	1.00E-05	0.016008513	0.026292409	0.043180141	0.949406264
chr1:17000494	rs145515028	ATP13A2_A244V	G	A	ATP13A2	0.2666	1.170814898	0.886370716	1.546539725	Kufor-Rakeb_syr	Not_PD_known	Conflicting_interpretations_of_pathogenicity	TRUE	I	0.822054029	NA	1.33E-05	NA	NA	NA	0.991015393
chr1:20644515	rs372280083	PINK1_L268V	C	G	PINK1	0.2437	2.297220698	0.567471926	9.29953129	Parkinson_disea	PD_known	Uncertain_significance	TRUE	I	0.745930791	NA	1.67E-06	NA	NA	NA	0.280830776
chr3:132475009	rs149121829	DNAJC13_S790Y	C	A	DNAJC13	0.2875	1.059079357	0.9527145	1.177319212	not_provided	Not_PD_known	Likely_benign	TRUE	I	0.695607781	0.00209859	0.000141667	0.01650556	0.020251688	0.024844064	1
chr22:38120886	rs143826762	PLA2G6_G539S	C	T	PLA2G6	0.3387	1.117730176	0.889898382	1.403891467	Iron_accumulatic	PD_known	Conflicting_interpretations_of_pathogenicity	TRUE	I	0.756161094	NA	2.33E-05	NA	NA	NA	0.999759631
chr14:22875924	rs138170865	LRP10_G326S	G	A	LRP10	0.8674	1.025417657	0.764069901	1.376158608	.	Not_PD_known	.	TRUE	I	0.632698894	0.000232721	1.67E-05	0.009299607	0.021484988	0.04962906	0.99936738
chr15:89330106	rs138929605	POLG_H277L	T	A	POLG	0.7297	1.063217832	0.75110637	1.505022728	Progressive_scle	Not_PD_known	Conflicting_interpretations_of_pathogenicity	TRUE	I	0.744691551	0.000103	1.33E-05	0.02565275	0.038834772	0.058785862	0.970016439
chr6:161360168	rs766915327	PRKN_R402H	C	T	PRKN	0.4647	3.736315629	0.109089787	127.9684821	Parkinson_disea	PD_known	Uncertain_significance	FALSE	G	NA	NA	0	NA	NA	NA	0.136874835
chr15:89333347	rs115109291	POLG_D136E	G	C	POLG	0.7837	128258.1104	4.35E-32	3.78E+41	Progressive_scle	Not_PD_known	Conflicting_interpretations_of_pathogenicity	FALSE	I	0.306628048	NA	0	0	NA	1	0.069267908
chr12:40354486	rs200762374	LRRK2_K2255M	A	T	LRRK2	0.6092	1.256839203	0.523239492	3.018970865	.	Not_PD_known	.	TRUE	I	0.671317637	Inf	1.67E-06	0	0	0.175307329	0.435373238
chr15:89321217	rs375935084	POLG_P881L	G	A	POLG	0.0468	2.11446114	1.010522981	4.42438816	Progressive_scle	Not_PD_known	Conflicting_interpretations_of_pathogenicity	TRUE	I	0.596869469	9.79E-06	1.67E-06	0.016700823	0.051057903	0.156082194	0.378279233
chr12:40251369	rs113065049	LRRK2_V366M	G	A	LRRK2	0.6418	3.911418125	0.012492535	1224.666695	Parkinson_disea	PD_known	Uncertain_significance	TRUE	G	NA	NA	0	0	0	0.177147799	0.085254914
chr3:184321315	rs147855566	EIF4G1_R244Q	G	A	EIF4G1	0.5265	4.037396284	0.053747614	303.2798571	.	Not_PD_known	.	FALSE	I	0.950605273	NA	0	0	0	1	0.052000349
chr1:20648612	rs45478900	PINK1_G411S	G	A	PINK1	0.01584	1.525008525	1.082416455	2.148573213	Parkinson_disea	PD_known	Conflicting_interpretations_of_pathogenicity	TRUE	I	0.728206813	NA	3.33E-06	NA	NA	NA	0.451664268
chr15:61915725	rs145670690	VPS13C_R2785C	G	A	VPS13C	0.8772	1.117730176	0.27239883	4.586366049	not_provided	Not_PD_known	Benign	TRUE	I	0.814133108	NA	0	NA	NA	NA	0.167893817
chr15:61961754	rs116290654	VPS13C_P1248L	G	A	VPS13C	0.3022	1.529131611	0.682470484	3.426145951	.	Not_PD_known	.	FALSE	I	0.45980683	NA	3.33E-06	NA	NA	NA	0.53081741
chr15:89333627	rs28567406	POLG_Q43R	T	C	POLG	0.4463	1.059079357	0.913580303	1.22775095	Seizures|Progres	Not_PD_known	Benign	TRUE	I	0.857893169	0.000897248	5.67E-05	0.012837046	0.018946819	0.02796005	1
chr1:16986335	rs377703085	ATP13A2_R1043C	G	A	ATP13A2	0.5445	1.124794104	0.769018694	1.645163876	Kufor-Rakeb_syr	Not_PD_known	Conflicting_interpretations_of_pathogenicity	TRUE	I	0.69316715	NA	1.17E-05	NA	NA	NA	0.904006672
chr1:16986101	rs41273151	ATP13A2_T1121S	T	A	ATP13A2	0.9678	1.002302647	0.895651404	1.121653572	Kufor-Rakeb_syr	Not_PD_known	Benign/Likely_benign	TRUE	I	0.877379298	NA	9.83E-05	NA	NA	NA	1
chr1:20637908	rs45608139	PINK1_R152W	C	T	PINK1	0.5468	1.668958869	0.315497664	8.828666642	Parkinson_disea	PD_known	Uncertain_significance	TRUE	I	0.57090354	NA	0	NA	NA	NA	0.107550905
chr15:61977193	rs115290371	VPS13C_T766N	G	T	VPS13C	0.3779	6.761873523	0.096635994	473.1459958	not_provided	Not_PD_known	Benign	TRUE	I	0.974546611	NA	0	NA	NA	NA	0.300606275
chr15:89327300	rs775538075	POLG_Y434H	A	G	POLG	0.5021	4.589173764	0.053637866	392.6426851	not_provided	Not_PD_known	Likely_pathogenic	TRUE	G	NA	NA	0	0	0	0.146837671	0.089978023
chr12:40293629	rs201810995	LRRK2_C925Y	G	A	LRRK2	0.965	1.144422336	0.002761713	474.235484	.	Not_PD_known	.	FALSE	I	0.432576895	NA	NA	NA	NA	NA	0.06909856
chr2:232847564	rs143181034	GIGYF2_Q1226P	A	C	GIGYF2	0.1827	1.190055566	0.921294069	1.53722063	.	Not_PD_known	.	FALSE	I	0.479182035	NA	NA	NA	NA	NA	1
chr15:89326947	rs61752783	POLG_G517V	C	A	POLG	0.5254	1.03138236	0.937487364	1.134681506	Seizures|Toe_wa	Not_PD_known	Conflicting_interpretations_of_pathogenicity	TRUE	I	0.984716177	0.001315057	0.000125	0.024870399	0.028515879	0.032693099	1
chr15:61984938	rs551048142	VPS13C_K547R	T	C	VPS13C	0.9015	2.968331469	9.72E-08	90681869.51	.	Not_PD_known	.	TRUE	I	0.503913999	NA	NA	NA	NA	NA	0.064938185
chr21:32666479	rs373820739	SYNJ1_V675I	C	T	SYNJ1	0.05307	5.929856419	0.976684116	36.00263031	Epileptic_enceph	PD_known	Conflicting_interpretations_of_pathogenicity	FALSE	I	0.349877506	NA	0	0	0	0.240700408	0.137046698
chr15:89318599	rs2307442	POLG_R1142W	G	A	POLG	0.6656	4.368413938	0.005446795	3503.535919	Progressive_scle	Not_PD_known	Uncertain_significance	TRUE	G	NA	NA	0	1.13E-17	0	0.258904931	0.071904458
chr11:94447275	rs139461096	MRE11_R576Q	C	T	MRE11	0.5486	1.196618904	0.665689456	2.150998171	Depression|Dem	PD_known	Conflicting_interpretations_of_pathogenicity	TRUE	I	0.795584857	6.41E-05	5.00E-06	0.005514952	0.023397868	0.099252453	0.727624454
chr20:5109435	rs149904653	TMEM230_R62H	C	T	TMEM230	0.3364	1.805251649	0.541333359	6.020197102	.	Not_PD_known	.	FALSE	I	0.305997491	1.25E-05	1.67E-06	0.00951182	0.039874493	0.167131055	0.368261579
chr1:155235843	rs76763715	GBA1_N409S	T	C	GBA1	3.31E-58	2.260305543	2.046493826	2.496455685	Thrombocytopen	PD_known	Pathogenic/Likely_pathogenic|_risk_factor	FALSE	G	1.000000119	0.0003298	5.33E-05	0.043765751	0.048514292	0.053775065	1
chr12:40351680	rs72547981	LRRK2_D2175H	G	C	LRRK2	0.2785	1.735160645	0.640465515	4.700928301	Parkinson_disea	PD_known	Uncertain_significance	FALSE	G	NA	0.000920261	1.33E-05	0.001587569	0.004346591	0.011898622	0.992728201
chr1:155236367	rs374306700	GBA1_R368C	G	A	GBA1	0.9352	2.750272506	7.07E-11	1.07E+11	Gaucher_disease	Not_PD_known	Conflicting_interpretations_of_pathogenicity	FALSE	G	NA	NA	0	0	0	1	0.051946398
chr22:38140106	rs587784359	PLA2G6_H225Y	G	A	PLA2G6	0.7251	1.433329415	0.19269474	10.66159466	Iron_accumulatic	Not_PD_known	Pathogenic/Likely_pathogenic	FALSE	G	NA	NA	0	NA	NA	NA	0.135935272
chr15:61961728	rs533261054	VPS13C_I1257V	T	C	VPS13C	0.9643	1.050745592	0.119793722	9.216395338	.	Not_PD_known	.	FALSE	I	NA	NA	1.67E-06	NA	NA	NA	NA
chr22:38169336	rs150024227	PLA2G6_D31N	C	T	PLA2G6	0.5688	1.119408029	0.759359473	1.650172784	Neurodegenerati	PD_known	Uncertain_significance	TRUE	I	0.644730151	NA	8.33E-06	NA	NA	NA	0.95416274
chr15:89318581	rs149099318	POLG_R1148C	G	A	POLG	0.3157	2.710681285	0.386490496	19.01157492	Progressive_scle	Not_PD_known	Uncertain_significance	TRUE	G	NA	0	0	0.003068668	0.02110048	0.145076742	0.107855682
chr6:162054109	rs72480421	PRKN_H200Q	G	C	PRKN	0.9444	2.755227455	1.20E-12	6.33E+12	.	Not_PD_known	.	FALSE	G	NA	NA	0	NA	NA	NA	0.050991255
chr1:16985990	rs76298930	ATP13A2_V1158M	C	T	ATP13A2	0.4831	1.115943237	0.821318749	1.51625579	Kufor-Rakeb_syr	Not_PD_known	Benign	TRUE	I	0.969541907	NA	8.33E-06	NA	NA	NA	0.935253998
chr22:38115658	rs587784339	PLA2G6_R635X	G	A	PLA2G6	0.5054	4.654339452	0.050396175	429.8515859	Iron_accumulatic	Not_PD_known	Pathogenic	TRUE	G	NA	NA	0	NA	NA	NA	0.194707077
chr1:65384226	rs186061249	DNAJC6_A177T	G	A	DNAJC6	0.7176	1.478754235	0.177439069	12.32374637	.	Not_PD_known	.	TRUE	I	0.607365966	NA	0	NA	NA	NA	0.117132896
chr15:89320890	rs11546842	POLG_R953C	G	A	POLG	0.9787	2.723723832	2.77E-32	2.68E+32	Progressive_scle	Not_PD_known	Conflicting_interpretations_of_pathogenicity	FALSE	G	NA	NA	0	0	0	1	0.05100697
chr15:89319065	rs181860632	POLG_R1047W	G	A	POLG	0.9801	1.01643357	0.280763107	3.679747001	Seizures|Progres	Not_PD_known	Conflicting_interpretations_of_pathogenicity	TRUE	G	0.431543291	0	0	0.004385797	0.018162413	0.075207587	0.206463722
chr15:61868702	rs116228685	VPS13C_R3607H	C	T	VPS13C	0.2993	1.210822644	0.843725563	1.737640225	.	Not_PD_known	.	TRUE	I	0.815254271	NA	1.00E-05	NA	NA	NA	0.920767982
chr2:232847399	rs72554081	GIGYF2_H1171R	A	G	GIGYF2	0.7345	1.035205544	0.847788572	1.264053979	.	Not_PD_known	.	TRUE	I	0.793560803	0.000429279	3.50E-05	0.015753293	0.0244596	0.037971503	0.999999888
chr4:89828156	rs201106962	SNCA_H50Q	A	C	SNCA	0.01365	2.01818784	1.155101247	3.526168956	Lewy_body_dem	PD_known	Uncertain_significance	TRUE	I	0.567412972	2.05E-05	3.33E-06	0.016750218	0.048739092	0.141796283	0.635652365
chr16:46683521	rs145147781	VPS35_M30K	A	T	VPS35	0.3867	1.344335717	0.687822822	2.627476816	Parkinson_disea	PD_known	Uncertain_significance	TRUE	I	0.649241269	1.90E-05	3.33E-06	0.016786646	0.052639516	0.165040405	0.587310539
chr15:61950998	rs11629598	VPS13C_I1495V	T	C	VPS13C	0.2721	1.016941914	0.986898581	1.047899831	not_provided	Not_PD_known	Benign	TRUE	I	0.999026895	NA	0.001285	NA	NA	NA	1
chr6:161350139	rs137853056	PRKN_W453X	C	T	PRKN	0.8557	7.467796681	2.90E-09	19230665436	Parkinson_disea	PD_known	Likely_pathogenic	FALSE	G	NA	NA	0	NA	NA	NA	0.07389728
chr12:40225558	rs72546335	LRRK2_S52F	C	T	LRRK2	0.989	2.719641309	7.10E-62	1.04E+62	Parkinson_disea	PD_known	Uncertain_significance	FALSE	G	NA	NA	0	0	0	1	0.050976214
chr15:61983868	rs116803472	VPS13C_D622E	G	T	VPS13C	0.6618	6723.741168	4.74E-14	9.54E+20	not_provided	Not_PD_known	Likely_benign	FALSE	I	0.445127696	NA	0	NA	NA	NA	0.05639457
chr2:74530221	rs150047108	HTRA2_L72P	T	C	HTRA2	0.1929	1.105944809	0.950461142	1.286863677	Parkinson_disea	PD_known	Conflicting_interpretations_of_pathogenicity	TRUE	I	0.901501954	0.000861088	5.83E-05	0.014676182	0.020323129	0.028138341	1
chr21:32665968	rs147929290	SYNJ1_I746T	A	G	SYNJ1	0.3969	1.099988802	0.882320421	1.371355957	Parkinson_disea	PD_known	Conflicting_interpretations_of_pathogenicity	TRUE	I	0.950167537	0.000288669	2.33E-05	0.01716884	0.02424926	0.034246905	0.999951153
chr6:161569357	rs137853055	PRKN_Q311X	G	A	PRKN	1	7.389056099	0	Inf	Parkinson_disea	PD_known	Pathogenic	FALSE	G	NA	NA	0	NA	NA	NA	NA
chr21:32673463	rs145712835	SYNJ1_V574I	C	T	SYNJ1	0.171	1.998106536	0.741725537	5.382624067	Parkinson_disea	PD_known	Uncertain_significance	TRUE	I	0.641366303	0.000159576	5.00E-06	0.002212142	0.009399914	0.039936063	0.338368315
chr15:89318710	rs753410045	POLG_A1105T	C	T	POLG	0.9162	2.74505195	1.88E-08	400709787	.	Not_PD_known	.	TRUE	G	NA	NA	0	0	0	1	0.051319015
chr4:89828149	rs104893877	SNCA_A53T	C	T	SNCA	0.006094	37.97773246	2.823123793	510.8908671	Lewy_body_dem	PD_known	Pathogenic	TRUE	G	NA	0	0	0.044291973	0.587418697	1	0.051416387
chr15:89328996	rs2307437	POLG_P324S	G	A	POLG	0.3751	1.206109632	0.797121632	1.824941625	Progressive_scle	Not_PD_known	Conflicting_interpretations_of_pathogenicity	TRUE	I	0.581538796	0.000145339	1.00E-05	0.008983699	0.020641422	0.047419228	0.953772427
chr5:1443128	rs201800694	SLC6A3_V24M	C	T	SLC6A3	0.8881	1.032930595	0.657454315	1.622843731	Tobacco_addictic	PD_known	Uncertain_significance	TRUE	I	0.520162463	0.000156323	1.00E-05	0.008955615	0.019191018	0.041117909	0.787893776
chr3:184328662	rs112809828	EIF4G1_M1330V	A	G	EIF4G1	0.05358	4.767394837	0.976059238	23.2855268	.	Not_PD_known	.	FALSE	I	0.332434237	Inf	1.67E-06	0	0	0.044348548	0.338025268
chr1:20648534	rs763416852	PINK1_F385L	T	C	PINK1	0.8871	7.512738151	6.04E-12	9.35E+12	not_provided	Not_PD_known	Likely_pathogenic	FALSE	G	NA	NA	0	NA	NA	NA	0.054552648
chr1:65392779	rs199937139	DNAJC6_H549P	A	C	DNAJC6	0.5738	1.166374244	0.682252128	1.994026577	not_provided	Not_PD_known	Uncertain_significance	TRUE	I	0.707299769	NA	5.00E-06	NA	NA	NA	0.767374933
chr15:89317460	rs369544574	POLG_R1187W	G	A	POLG	0.6232	1.460384855	0.322365914	6.615848113	Progressive_scle	Not_PD_known	Conflicting_interpretations_of_pathogenicity	FALSE	I	0.409295917	Inf	1.67E-06	0	0	0.175311936	0.258366256
chr3:132480451	rs202174230	DNAJC13_R952Q	G	A	DNAJC13	0.6295	1.176213052	0.60844546	2.273789903	.	Not_PD_known	.	TRUE	I	0.818664432	0	0	0.032423256	0.430010836	1	0.173807547
chr6:161785839	rs377554392	PRKN_C268X	A	T	PRKN	0.9214	2.737376555	5.69E-09	1317755833	not_provided	Not_PD_known	Pathogenic	FALSE	G	NA	NA	0	NA	NA	NA	0.066936356
chr3:132499779	rs3762672	DNAJC13_A1463S	G	T	DNAJC13	0.03262	1.019487449	1.001465072	1.037834157	not_specified|not	Not_PD_known	Benign	TRUE	I	1.000000119	0.112123629	0.007856667	0.02067988	0.021021439	0.021367084	1

chr12:40257348	rs79656150	LRRK2_C463W	T	G	LRRK2	0.936	2.767377166	4.54E-11	1.69E+11	.	Not_PD_known	.	FALSE	G	NA	NA	0	0	0	1	0.052639219
chr3:184327434	rs34086109	EIF4G1_R1217H	G	A	EIF4G1	0.3407	7.25869456	0.122998884	428.3668694	.	Not_PD_known	.	TRUE	I	0.844895422	NA	0	0	NA	1	0.158215238
chr6:161785844	rs114696251	PRKN_Y267H	A	G	PRKN	0.9457	2.727812484	7.75E-13	9.60E+12	Parkinson_disea	PD_known	Uncertain_significance	FALSE	G	0.476515204	NA	0	NA	NA	NA	0.07257032
chr22:38113591	rs587784346	PLA2G6_Q700X	G	A	PLA2G6	0.8953	2.775691763	6.94E-07	11108798.39	Iron_accumulatio	Not_PD_known	Pathogenic	TRUE	G	NA	NA	0	NA	NA	NA	0.053198333
chr1:155236376	rs2230288	GBA1_E365K	C	T	GBA1	2.63E-26	1.437923415	1.344696047	1.537614209	.	Not_PD_known	.	FALSE	G	0.999916971	0.002176292	0.000206667	0.026675755	0.028488824	0.030423311	1
chr3:184323921	rs62287499	EIF4G1_I807V	A	G	EIF4G1	0.003556	1.866938643	1.226872195	2.840931525	.	Not_PD_known	.	TRUE	I	0.683925271	0.000354619	1.17E-05	0.004173549	0.00986976	0.023336628	0.945694146
chr2:232844498	rs566316792	GIGYF2_G1077R	G	C	GIGYF2	0.9398	3.725868578	5.61E-15	2.47E+15	.	Not_PD_known	.	FALSE	I	0.804408073	NA	NA	NA	NA	NA	0.051378646
chr3:132525718	rs138693725	DNAJC13_A2057S	G	T	DNAJC13	0.5035	1.094502585	0.840209511	1.425758567	.	Not_PD_known	.	TRUE	I	0.810351133	0.000313468	1.83E-05	0.009342649	0.017545641	0.032945712	0.999321112
chr1:155239639	rs794727708	GBA1_L144R	A	C	GBA1	0.2689	6.780155251	0.227920134	201.6956746	Gaucher_disease	Not_PD_known	Conflicting_interpretations_of_pathogenicity	FALSE	G	NA	0	0	0.120793467	1	1	0.05072928
chr1:155235813	rs77284004	GBA1_D419A	T	G	GBA1	0.007328	3401.255816	8.919144197	1297046.092	.	Not_PD_known	.	FALSE	G	NA	0	0	0.014502977	0.179262656	1	0.055284449
chr1:20649034	rs74315361	PINK1_Y431H	T	C	PINK1	0.9073	2.8170775	7.62E-08	104081832.6	Parkinson_disea	PD_known	risk_factor	TRUE	G	NA	NA	0	NA	NA	NA	0.051545587
chr22:38115583	rs587784341	PLA2G6_P660S	G	A	PLA2G6	0.8703	2.77819101	1.31E-05	588841.3079	Iron_accumulatio	Not_PD_known	Likely_pathogenic	TRUE	G	NA	NA	0	NA	NA	NA	0.053481212
chr20:5106224	rs148033002	TMEM230_I62M	T	C	TMEM230	0.2371	1.169527709	0.902036765	1.516340703	.	Not_PD_known	.	TRUE	I	0.613953412	0.000565708	2.17E-05	0.005184075	0.011490024	0.025462499	0.999913837
chr12:40363440	rs113511708	LRRK2_T2356I	C	T	LRRK2	0.5563	1.283126913	0.559144132	2.944526432	Parkinson_disea	PD_known	Conflicting_interpretations_of_pathogenicity	TRUE	I	0.69224751	2.94E-05	3.33E-06	0.010403904	0.034018768	0.111219451	0.568009725
chr1:175403578	rs61731112	TNR_N180H	T	G	TNR	0.01245	1.140309823	1.028807125	1.263897246	Parkinson_disea	PD_known	Conflicting_interpretations_of_pathogenicity	TRUE	I	0.959826946	0.00102554	9.67E-05	0.024140616	0.028277789	0.033121337	1
chr12:40304040	rs60185966	LRRK2_S1228T	G	C	LRRK2	0.6368	1.171400451	0.607263791	2.259609476	Parkinson_disea	PD_known	Uncertain_significance	TRUE	G	0.550128639	8.11E-05	6.67E-06	0.012530286	0.024655627	0.048507739	0.78108995
chr15:61991076	rs140027316	VPS13C_T501S	G	C	VPS13C	0.8836	10.21645852	3.15E-13	3.31E+14	.	Not_PD_known	.	FALSE	I	0.541032016	NA	0	NA	NA	NA	0.052321215
chr3:195868341	rs201407161	TNK2_V638M	C	T	TNK2	0.1241	1.237384651	0.943215968	1.623298191	Parkinson_disea	PD_known	Conflicting_interpretations_of_pathogenicity	TRUE	I	0.677645087	0.000168464	1.67E-05	0.01560745	0.029679919	0.056431797	0.998107638
chr2:232747717	rs147623346	GIGYF2_M48I	G	T	GIGYF2	0.1291	1.460238823	0.895457964	2.381236761	.	Not_PD_known	.	TRUE	I	0.806977868	1.14E-05	3.33E-06	0.006598248	0.087730366	1	0.572350935
chr12:40284061	rs72546337	LRRK2_I810V	A	G	LRRK2	0.9263	2.757432519	1.30E-09	5864458574	Parkinson_disea	PD_known	Uncertain_significance	FALSE	G	NA	NA	0	0	0	1	0.054286611
chr1:16986248	rs3170740	ATP13A2_A1072T	C	T	ATP13A2	0.0003886	1.032930595	1.014670572	1.051519226	Kufor-Rakeb_syr	Not_PD_known	Benign	TRUE	I	0.983309686	NA	0.007963333	NA	NA	NA	1
chr1:20644526	rs28940284	PINK1_H271Q	C	A	PINK1	0.989	2.719097435	8.23E-62	8.98E+61	Parkinson_disea	PD_known	Pathogenic	FALSE	G	NA	NA	0	NA	NA	NA	0.05185299
chr12:40309174	rs113589830	LRRK2_D1420N	G	A	LRRK2	0.885	2.868817718	1.79E-06	4588060.758	Parkinson_disea	PD_known	Uncertain_significance	TRUE	G	NA	NA	0	0	0	1	0.051150961
chr22:38126390	rs201801144	PLA2G6_M470V	T	C	PLA2G6	0.9016	1.027573297	0.667644121	1.581541493	Infantile_neuroax	Not_PD_known	Uncertain_significance	TRUE	I	0.716191053	NA	8.33E-06	NA	NA	NA	0.947007339
chr15:61929546	rs191668294	VPS13C_I2081F	T	A	VPS13C	0.001242	78.73596188	5.562145972	1114.561129	.	Not_PD_known	.	TRUE	I	0.577219069	NA	0	NA	NA	NA	0.052514471
chr12:40295466	rs75148313	LRRK2_S973N	G	A	LRRK2	0.2456	3.329093351	0.437154762	25.35226309	Parkinson_disea	PD_known	Uncertain_significance	TRUE	G	NA	0	0	0.006113419	0.044573397	0.324960909	0.078692012
chr1:16993627	rs74058364	NA	A	C	ATP13A2	0.3646	1.150618933	0.849665956	1.558169913	Kufor-Rakeb_syr	Not_PD_known	Uncertain_significance	FALSE	I	0.335068256	NA	NA	NA	NA	NA	1
chr15:89318677	rs201144044	POLG_M1116V	T	C	POLG	0.424	1.729789976	0.451407861	6.62853623	Progressive_scle	Not_PD_known	Uncertain_significance	TRUE	G	NA	2.03E-05	1.67E-06	0.005816929	0.02459523	0.103985085	0.143038569
chr1:17011732	rs549839037	ATP13A2_A3P	C	G	ATP13A2	0.588	1.61E+112	1.98E-294	Inf	Kufor-Rakeb_syr	Not_PD_known	Conflicting_interpretations_of_pathogenicity	FALSE	I	0.686631024	NA	0	NA	NA	NA	0.067346591
chr1:155235798	rs772548282	GBA1_L424P	A	G	GBA1	0.8569	7.52100671	2.25E-09	25135625012	Gaucher_disease	Not_PD_known	Likely_pathogenic	FALSE	G	NA	NA	0	0	0	1	0.069321361
chr21:32673511	rs148901211	SYNJ1_S558G	T	C	SYNJ1	0.9598	1.019181649	0.486409703	2.135506809	Parkinson_disea	PD_known	Uncertain_significance	TRUE	I	0.622689605	6.84E-05	3.33E-06	0.001183046	0.014622088	0.180695544	0.600006171
chr12:40323300	rs71653641	LRRK2_L1884F	C	T	LRRK2	0.1049	7.62932977	0.654502417	88.93270855	.	Not_PD_known	.	TRUE	G	NA	0	0	0.025080102	0.332620508	1	0.066548904
chr15:61951827	rs8026956	VPS13C_T1485A	T	C	VPS13C	0.02641	2.990976731	1.136939496	7.868441403	not_provided	Not_PD_known	Benign	TRUE	I	0.942186952	NA	0	NA	NA	NA	0.15339721
chr15:89322799	rs191490663	POLG_R790H	C	T	POLG	0.2525	2.27118109	0.557313144	9.255592839	Seizures|Progres	Not_PD_known	Uncertain_significance	TRUE	I	0.672494054	NA	0	0	0	1	0.139373387
chr22:38133010	rs528966598	PLA2G6_A300T	C	T	PLA2G6	0.2544	5.166006743	0.306765398	86.99685773	Neurodegenerati	PD_known	Conflicting_interpretations_of_pathogenicity	FALSE	I	0.320729524	NA	3.33E-06	NA	NA	NA	0.111972549
chr1:155236441	rs77321207	GBA1_Y343C	T	C	GBA1	0.9817	2.722362311	1.99E-37	3.73E+37	.	Not_PD_known	.	FALSE	G	NA	NA	0	0	0	1	0.05318671
chr22:38145447	rs141825182	PLA2G6_R139H	C	T	PLA2G6	0.3619	1.166957576	0.837421291	1.626170721	Neurodegenerati	PD_known	Conflicting_interpretations_of_pathogenicity	TRUE	I	0.762986779	NA	1.17E-05	NA	NA	NA	0.981875318
chr1:155237423	rs199628072	GBA1_T306I	G	A	GBA1	0.6285	1.196977944	0.577795083	2.479696069	.	Not_PD_known	.	TRUE	I	0.710893631	0.000136779	3.33E-06	0.00073835	0.007311044	0.072381432	0.499053308
chr14:22875772	rs201377432	LRP10_T275N	C	A	LRP10	0.9273	3.679217155	2.55E-12	5.31E+12	.	Not_PD_known	.	FALSE	I	0.575367987	NA	NA	NA	NA	NA	0.052262724
chr2:232794778	rs142481025	GIGYF2_S438L	C	T	GIGYF2	0.5849	1.26896296	0.539906123	2.982494408	.	Not_PD_known	.	TRUE	I	0.615765393	1.90E-05	3.33E-06	0.007647017	0.052636923	0.362259161	0.520632392
chr12:40278154	rs199566791	LRRK2_M712V	A	G	LRRK2	0.8903	7.465556678	2.96E-12	1.88E+13	Parkinson_disea	PD_known	Uncertain_significance	FALSE	G	NA	NA	0	0	0	1	0.07729137
chr3:184327609	rs35629949	EIF4G1_P1230A	C	G	EIF4G1	0.6063	1.031691822	0.916328825	1.161578667	not_provided	Not_PD_known	Likely_benign	TRUE	I	0.853231668	0.001250031	8.33E-05	0.01470368	0.019999496	0.027198345	1
chr15:61962790	rs3784635	VPS13C_I1132V	T	C	VPS13C	0.06099	1.057386185	0.997395398	1.120985266	not_provided	Not_PD_known	Benign	TRUE	I	1.000000119	NA	0.00042	NA	NA	NA	1
chr22:38116105	rs139579057	PLA2G6_V617I	C	T	PLA2G6	0.6843	1.17880357	0.533487307	2.604706498	.	Not_PD_known	.	TRUE	I	0.720932007	NA	1.67E-06	NA	NA	NA	0.364558281
chr12:40367012	rs281865057	LRRK2_L2466H	T	A	LRRK2	0.886	7.437241396	9.14E-12	6.05E+12	Parkinson_disea	PD_known	Uncertain_significance	FALSE	G	NA	NA	0	0	0	0.342092379	0.101607775
chr1:17005517	rs56379718	ATP13A2_G49S	C	T	ATP13A2	0.5178	1.22311392	0.664351435	2.251831759	Kufor-Rakeb_syr	Not_PD_known	Benign/Likely_benign	TRUE	I	0.999579966	NA	3.33E-06	NA	NA	NA	0.555236957
chr3:184315839	rs759151568	EIF4G1_S15P	T	C	EIF4G1	0.47	1.159396951	0.776225662	1.731714572	.	Not_PD_known	.	TRUE	I	0.708743334	0.000129517	1.00E-05	0.010552507	0.023162964	0.05083477	0.967357528
chr20:5069205	rs553953692	TMEM230_V123M	C	T	TMEM230	0.7697	1.57E+137	0	Inf	.	Not_PD_known	.	FALSE	I	0.449733466	NA	NA	NA	NA	NA	0.096828043
chr15:89325531	rs758438414	POLG_L623W	A	C	POLG	0.7653	7.104298334	1.82E-05	2766783.466	Progressive_scle	Not_PD_known	Conflicting_interpretations_of_pathogenicity	TRUE	G	0.387184262	NA	0	0	0	1	0.053040782
chr1:16988226	rs564643512	ATP13A2_R880H	C	T	ATP13A2	0.001686	5.033925011	1.835990284	13.80203438	Kufor-Rakeb_syr	Not_PD_known	Uncertain_significance	FALSE	I	0.497981548	NA	5.00E-06	NA	NA	NA	0.295171522
chr15:89318989	rs530757118	POLG_T1072S	G	C	POLG	0.9255	2.732453709	1.93E-09	3876273148	Progressive_scle	Not_PD_known	Uncertain_significance	FALSE	G	NA	NA	0	0	0	0.839297401	0.055745651
chr1:20645615	rs55831733	PINK1_A339T	G	A	PINK1	0.9757	1.004409694	0.758903994	1.329336572	Parkinson_disea	PD_known	Conflicting_interpretations_of_pathogenicity	TRUE	I	0.56177485	NA	2.67E-05	NA	NA	NA	0.998797301
chr3:184320964	rs752416317	EIF4G1_T223M	C	T	EIF4G1	0.9147	1.163927428	0.072260329	18.74786726	.	Not_PD_known	.	FALSE	I	0.343666732	NA	0	0	NA	1	0.188702159
chr22:38112186	rs587784354	PLA2G6_L799H	A	T	PLA2G6	0.8243	8.172705462	7.20E-08	927211265.6	Iron_accumulatio	Not_PD_known	Likely_pathogenic	TRUE	G	NA	NA	0	NA	NA	NA	0.050425772
chr12:40351585	rs201271001	LRRK2_R2143H	G	A	LRRK2	0.2003	1.523789006	0.799759677	2.903288326	Parkinson_disea	PD_known	Uncertain_significance	TRUE	I	0.593727291	1.87E-05	1.67E-06	0.004109235	0.026668692	0.1730542	0.201077791
chr1:155238264	rs188760929	GBA1_V211I	C	T	GBA1	0.1927	567.5336257	0.040731074	7907830.27	.	Not_PD_known	.	FALSE	I	0.361517966	NA	1.67E-06	0	NA	1	0.206849666
chr15:62034998	rs151147947	VPS13C_A81V	G	A	VPS13C	0.5974	1.158469804	0.671151758	1.999625674	.	Not_PD_known	.	TRUE	I	0.790020704	NA	3.33E-06	NA	NA	NA	0.597680512
chr15:89320832	rs200309005	POLG_R972Q	C	T	POLG	0.6651	20.66827674	2.29E-05	18631569.78	Progressive_scle	Not_PD_known	Uncertain_significance	TRUE	G	NA	NA	0	0	0	0.174057361	0.079772198
chr22:38112558	rs121908686	PLA2G6_R741Q	C	T	PLA2G6	0.02739	8.324475243	1.266463031	54.71686609	Neurodegenerati	PD_known	Pathogenic/Likely_pathogenic	TRUE	G	NA	NA	0	NA	NA	NA	0.079594815
chr15:89327198	rs145843073	POLG_N468D	T	C	POLG	0.7235	1.083828847	0.694055049	1.692495387	Intellectual_disat	Not_PD_known	Conflicting_interpretations_of_pathogenicity	TRUE	I	0.644990563	0.000128223	1.00E-05	0.011192731	0.023396716	0.048899473	0.946531514
chr3:184331523	rs545768471	EIF4G1_L1439V	C	G	EIF4G1	0.00275	52.68864672	3.932832877	705.8762932	.	Not_PD_known	.	TRUE	I	0.619143724	NA	0	0	NA	1	0.053334356
chr6:161386864	rs761213043	PRKN_R366Q	C	T	PRKN	0.2844	2.655412474	0.444274795	15.87129291	.	Not_PD_known	.	TRUE	G	NA	NA	0	NA	NA	NA	0.108787665
chr1:20649224	rs542258150	PINK1_A494V	C	T	PINK1	0.3898	7.172110767	0.080415205	639.6697324	Parkinson_disea	PD_known	Conflicting_interpretations_of_pathogenicity	FALSE	I	0.315431327	NA	3.33E-06	NA	NA	NA	0.534617659
chr1:155238215	rs364897	GBA1_N227S	T	C	GBA1	0.01506	3.30157623	1.260181805	8.649867475	Gaucher_disease	Not_PD_known	Pathogenic/Likely_pathogenic	FALSE	G	NA	0	0	0.01875473	0.050428275	0.135584616	0.138927267
chr15:89328699	rs199759055	POLG_R386C	G	A	POLG	0.663	21.0984589	2.34E-05	19060410.63	Progressive_scle	Not_PD_known	Conflicting_interpretations_of_pathogenicity	TRUE	G	NA	NA	0	0	0	0.130421596	0.102220093
chr12:40322038	rs72547979	LRRK2_R1725Q	G	A	LRRK2	0.5784	3.175610826	0.053948143	186.9295859	Parkinson_disea	PD_known	Uncertain_significance	FALSE	G	NA	NA	0	0	0	1	0.052632612
chr12:40340404	rs35870237	LRRK2_I2020T	T	C	LRRK2	0.0003658	21.41089704	3.969705855	115.4812293	Parkinson_disea	PD_known	Pathogenic	FALSE	G	NA	0	0	0.057140615	0.356520131	1	0.053934632
chr12:40293626	rs200795874	LRRK2_R924H	G	A	LRRK2	0.2246	1.852618419	0.68489345	5.01128315	not_provided	Not_PD_known	Uncertain_significance	FALSE	I	0.326654881	Inf	3.33E-06	0	0	1	0.582653885
chr1:65392613	rs377495314	DNAJC6_P494T	C	A	DNAJC6	0.9006	2580.916894	7.83E-51	8.51E+56	.	Not_PD_known	.	FALSE	I	0.710841417	NA	0	NA	NA	NA	0.062842168
chr14:22876078	rs1062656	LRP10_P377R	C	G	LRP10	0.9989	1.001701446	0.096790348	10.36679593	.	Not_PD_known	.	TRUE	I	0.671139896	NA	0	0	NA	1	0.150750201
chr14:22876722	rs142130715	LRP10_E486D	G	C	LRP10	0.09159	1.586451879	0.927969883	2.712188843	.	Not_PD_known	.	TRUE	I	0.675455928	2.93E-05	3.33E-06	0.010455916	0.034117365	0.111306191	0.6416356
chr1:155237453	rs78973108	GBA1_R296Q	C	T	GBA1	4.26E-06	3.711737144	2.122316116	6.491489427	Lewy_body_dem	PD_known	Pathogenic	TRUE	I	0.804317772	0	0	0.181638583	0.859859804	1	0.101704204
chr12:40359345	rs200002022	LRRK2_T2310M	C	T	LRRK2	0.9328	1.063855954	0.252400037	4.484109836	Parkinson_disea	PD_known	Conflicting_interpretations_of_pathogenicity	TRUE	I	0.700865805	5.00E-07	1.67E-06	0.025894722	1	1	0.341965962
chr12:40315266	rs721710	LRRK2_V1598E	T	A	LRRK2	0.9906	2.719369359	1.82E-72	4.06E+72	Parkinson_disea	PD_known	Uncertain_significance	FALSE	G	NA	NA	0	0	0	1	0.050554745
chr1:175406219	rs147204644	TNR_T166A	T	C	TNR	0.1975	1.077237614	0.962046974	1.2062206	Parkinson_disea	PD_known	Conflicting_interpretations_of_pathogenicity	TRUE	I	0.87587136	0.001384418	8.50E-05	0.013596378	0.018419293	0.024948999	1

chr1:16990276	rs200924194	ATP13A2_Q750E	G	C	ATP13A2	0.1585	1.28518156	0.906667037	1.821717978	Kufor-Rakeb_syr	Not_PD_known	Uncertain_significance	TRUE	I	0.589521766	NA	1.33E-05	NA	NA	NA	0.982417986
chr15:61868675	rs77673743	VPS13C_G3616D	C	T	VPS13C	0.9442	1.029939435	0.451018013	2.351957594	not_provided	Not_PD_known	Benign	TRUE	I	0.971663952	NA	1.67E-06	NA	NA	NA	0.398858747
chr15:89326688	rs2307447	POLG_R546C	G	A	POLG	0.6734	1.23392482	0.464469214	3.278086933	Seizures|Progres	Not_PD_known	Benign	TRUE	I	0.801049888	NA	0	0	0	1	0.183910699
chr3:132456751	rs147898644	DNAJC13_A423V	C	T	DNAJC13	0.03208	1.892313207	1.0560167	3.390902126	.	Not_PD_known	.	TRUE	I	0.698938608	2.66E-05	3.33E-06	0.006194824	0.037599654	0.22817559	0.530095094
chr22:32479132	rs139135860	FBXO7_D92H	G	C	FBXO7;FBXO7	0.4099	1.724953339	0.471549122	6.309976801	.	Not_PD_known	.	TRUE	G	NA	2.71E-05	1.67E-06	0.005418442	0.018465872	0.06292133	0.451564863
chr15:62012166	rs769205655	NA	T	C	VPS13C	0.8686	1.217744041	0.118058384	12.56073904	not_provided	Not_PD_known	Uncertain_significance	FALSE	G	NA	NA	1.67E-06	NA	NA	NA	0.156914063
chr1:20644551	rs772510148	PINK1_A280T	G	A	PINK1	0.7063	3.076522806	0.008901745	1063.273887	Parkinson_disea	PD_known	Uncertain_significance	FALSE	G	NA	NA	0	NA	NA	NA	0.070314933
chr15:89323426	rs113994097	POLG_W748S	C	G	POLG	0.6936	1.074547884	0.751265621	1.536943955	.	Not_PD_known	.	TRUE	G	0.417372018	0.000152475	1.00E-05	0.013752787	0.019675315	0.028146663	0.959992583
chr1:20649062	rs45467995	PINK1_G440E	G	A	PINK1	0.958	2.738471724	1.37E-16	5.48E+16	.	Not_PD_known	.	FALSE	G	NA	NA	0	NA	NA	NA	0.050296196
chr12:40294909	rs373486170	LRRK2_S958L	C	T	LRRK2	0.9628	2.830065907	2.95E-19	2.72E+19	.	Not_PD_known	.	FALSE	I	0.88652873	NA	0	0	NA	1	0.050024884
chr20:5111588	rs191127079	TMEM230_S29L	G	A	TMEM230	0.2857	3.491390217	0.351603204	34.66921093	.	Not_PD_known	.	FALSE	I	0.426888585	NA	NA	NA	NA	NA	0.372855484
chr1:65366050	rs61757223	DNAJC6_M76L	A	T	DNAJC6	0.1727	1.238251124	0.91079995	1.683427679	Parkinson_disea	PD_known	Uncertain_significance	TRUE	I	0.823803127	NA	1.17E-05	NA	NA	NA	0.98739111
chr22:32475369	rs199636063	FBXO7_R3Q	G	A	FBXO7	0.5721	18.64727457	0.000727908	477698.9494	.	Not_PD_known	.	TRUE	I	0.578762949	NA	0	NA	NA	NA	0.088949917
chr3:184327433	rs201711322	EIF4G1_R1217C	C	T	EIF4G1	0.5257	1.302258416	0.576110383	2.943666756	.	Not_PD_known	.	TRUE	I	0.57272768	1.14E-05	1.67E-06	0.00467846	0.043866263	0.411233837	0.4445427
chr1:20638080	rs34677717	PINK1_P209L	C	T	PINK1	0.02661	1.760679827	1.067701279	2.903427687	Parkinson_disea	PD_known	Uncertain_significance	TRUE	I	0.774448752	NA	1.67E-06	NA	NA	NA	0.212047638
chr3:184323209	rs112019125	EIF4G1_G686C	G	T	EIF4G1	0.5576	1.211912875	0.637569493	2.3036435	.	Not_PD_known	.	TRUE	I	0.722962618	5.05E-05	3.33E-06	0.006762743	0.019812711	0.058035717	0.481151372
chr12:40249843	rs200437744	LRRK2_L286V	C	G	LRRK2	0.2548	1.434333096	0.770875806	2.66879751	Parkinson_disea	PD_known	Conflicting_interpretations_of_pathogenicity	TRUE	I	0.525641084	6.08E-05	5.00E-06	0.009197558	0.024674165	0.06618245	0.46406818
chr15:89319318	rs201204229	POLG_V1005G	A	C	POLG	0.4124	1.278388116	0.710481763	2.300236629	not_provided	Not_PD_known	Uncertain_significance	FALSE	I	0.462970287	NA	NA	NA	NA	NA	0.968449426
chr14:22875844	rs556418686	LRP10_N299S	A	G	LRP10	0.7574	2.990976731	0.002866797	3120.53581	.	Not_PD_known	.	FALSE	I	NA	NA	0	0	0	1	NA
chr3:132457295	rs149087994	DNAJC13_V459A	T	C	DNAJC13	0.9471	3.37501578	8.50E-16	1.34E+16	.	Not_PD_known	.	FALSE	I	0.533691585	NA	0	0	NA	1	0.051807417
chr3:184322835	rs538505681	EIF4G1_L604V	C	G	EIF4G1	0.03281	4.916023638	1.138992386	21.2181299	.	Not_PD_known	.	TRUE	I	0.685688734	NA	0	0	0	0.366976761	0.076465723
chr6:162443384	rs770591350	PRKN_R33X	G	A	PRKN	0.0001771	5111993.482	1591.900716	16415896482	not_provided	Not_PD_known	Pathogenic	TRUE	G	NA	NA	0	NA	NA	NA	0.055872641
chr15:62023788	rs201028815	VPS13C_R169H	C	T	VPS13C	0.8744	1.204301824	0.120026827	12.0834893	.	Not_PD_known	.	FALSE	I	0.519678175	NA	0	NA	NA	NA	0.119532054
chr15:89320850	rs142347031	POLG_L966R	A	C	POLG	0.9411	1.044146703	0.331546237	3.288356842	Progressive_scle	Not_PD_known	Pathogenic/Likely_pathogenic	TRUE	G	0.449167311	1.79E-05	1.67E-06	0.00835714	0.027952672	0.093487172	0.182887167
chr1:16996298	rs149372969	ATP13A2_L432V	G	C	ATP13A2	0.5945	1.065666047	0.842976667	1.347183343	Kufor-Rakeb_syr	Not_PD_known	Conflicting_interpretations_of_pathogenicity	TRUE	I	0.69581902	NA	2.33E-05	NA	NA	NA	0.999732437
chr14:22877421	rs149685154	LRP10_H679P	A	C	LRP10	0.6049	1.123332821	0.722885816	1.745609887	.	Not_PD_known	.	TRUE	I	0.650684774	0.000185629	6.67E-06	0.004029437	0.01077417	0.028804057	0.753405785
chr12:40310435	rs34995376	LRRK2_R1441H	G	A	LRRK2	8.34E-11	43.03440878	13.82629204	133.9448302	Parkinson_disea	PD_known	Pathogenic	TRUE	G	NA	0	0	0.211980649	0.776426592	1	0.05565986
chr1:20644639	rs74315355	PINK1_G309D	G	A	PINK1	0.7985	55.72339073	2.20E-12	1.41E+15	Parkinson_disea	PD_known	Pathogenic	FALSE	G	NA	NA	0	NA	NA	NA	0.107910315
chr6:161973347	rs571490973	PRKN_A230V	G	A	PRKN	0.8969	4.034167658	2.76E-09	5895952880	Parkinson_disea	PD_known	Uncertain_significance	FALSE	I	0.73036617	NA	0	NA	NA	NA	0.05206312
chr12:40293644	rs281865045	LRRK2_Q930R	A	G	LRRK2	0.954	2.743405413	3.50E-15	2.15E+15	Parkinson_disea	PD_known	Uncertain_significance	FALSE	G	NA	NA	0	0	0	1	0.052585745
chr15:89323445	rs147827654	POLG_V742M	C	T	POLG	0.05688	4.698296518	0.955710726	23.09693674	Progressive_scle	Not_PD_known	Uncertain_significance	TRUE	G	0.342877388	0	0	0.018889533	0.091587102	0.444028119	0.081251508
chr14:22877070	rs142153001	LRP10_R562H	G	A	LRP10	0.2545	1.053481085	0.963032615	1.152424518	.	Not_PD_known	.	TRUE	I	0.926040828	0.001956213	0.000138333	0.016982571	0.021214463	0.026496656	1
chr22:38116155	rs149712244	PLA2G6_R600Q	C	T	PLA2G6	0.8665	2.758259873	2.00E-05	379693.6415	Iron_accumulatic	Not_PD_known	Pathogenic/Likely_pathogenic	TRUE	G	NA	NA	0	NA	NA	NA	0.05420977
chr15:61983818	rs775829630	NA	A	G	VPS13C	0.9489	2.742034053	1.07E-13	7.06E+13	.	Not_PD_known	.	FALSE	G	NA	NA	0	NA	NA	NA	0.0525898
chr12:40321114	rs35801418	LRRK2_Y1699C	A	G	LRRK2	0.9253	2.760191331	1.69E-09	4502019901	Parkinson_disea	PD_known	Pathogenic	FALSE	G	NA	NA	0	0	0	1	0.051338875
chr1:16997044	rs113105667	ATP13A2_V386I	C	T	ATP13A2	0.2985	1.725816031	0.616986584	4.827399901	Kufor-Rakeb_syr	Not_PD_known	Uncertain_significance	TRUE	I	0.563865483	NA	3.33E-06	NA	NA	NA	0.263836403
chr2:232791059	rs374338771	GIGYF2_V328L	G	C	GIGYF2	0.8761	1.116166448	0.280463412	4.442032314	.	Not_PD_known	.	TRUE	I	0.622499228	1.54E-05	1.67E-06	0.001951074	0.032497224	0.541170131	0.296225196
chr22:32475067	rs121918305	FBXO7_T22M	C	T	FBXO7	0.0004499	720787.7571	385.3130035	1348345335	Parkinson_disea	PD_known	Pathogenic	FALSE	G	NA	NA	0	NA	NA	NA	0.076655668
chr22:38140069	rs147066967	PLA2G6_R237H	C	T	PLA2G6	0.8335	1.075192806	0.547106389	2.113006891	.	Not_PD_known	.	TRUE	I	0.684845328	NA	1.67E-06	NA	NA	NA	0.476760411
chr1:20633766	rs202048763	PINK1_S73L	C	T	PINK1	0.8235	1.784074869	0.011030358	288.560278	Parkinson_disea	PD_known	Uncertain_significance	TRUE	I	0.851205885	NA	0	NA	NA	NA	0.073733409
chr15:89321773	rs796052889	POLG_A854D	G	T	POLG	0.9591	2.727539716	6.06E-17	1.23E+17	not_provided	Not_PD_known	Likely_pathogenic	FALSE	G	NA	NA	0	0	0	1	0.050644553
chr12:40298433	rs76535406	LRRK2_S1096C	C	G	LRRK2	0.8477	1.230351622	0.148357853	10.20347144	Parkinson_disea	PD_known	Uncertain_significance	TRUE	G	NA	4.83E-05	1.67E-06	0.00148095	0.010347166	0.072285637	0.234074872
chr3:184322631	rs145521479	EIF4G1_R566C	C	T	EIF4G1	0.8528	1.128624912	0.314329965	4.052410949	.	Not_PD_known	.	TRUE	I	0.510260046	Inf	1.67E-06	0	0	0.1225326	0.232116288
chr6:161785793	rs751037529	PRKN_G284R	C	G	PRKN	0.9437	2.756054147	1.64E-12	4.64E+12	not_provided	Not_PD_known	Pathogenic	FALSE	G	NA	NA	0	NA	NA	NA	0.052584023
chr15:89322800	rs775168496	POLG_R790C	G	A	POLG	0.9094	1.196259972	0.054659951	26.18073932	not_provided	Not_PD_known	Uncertain_significance	TRUE	G	NA	0	0	0.003367769	0.023395113	0.16250662	0.100412892
chr2:232844499	rs538735187	GIGYF2_G1077A	G	C	GIGYF2	0.9398	3.725868578	5.61E-15	2.47E+15	.	Not_PD_known	.	FALSE	I	0.804408073	NA	NA	NA	NA	NA	0.051307902
chr15:89322748	rs796052887	POLG_R807H	C	T	POLG	0.6261	20.72415649	0.000104741	4100485.749	Progressive_scle	Not_PD_known	Pathogenic	FALSE	G	NA	NA	0	0	0	0.100529739	0.112767743
chr12:40363541	rs79546190	LRRK2_V2390M	G	A	LRRK2	0.2983	3.359190425	0.34249302	32.94712488	Parkinson_disea	PD_known	Uncertain_significance	FALSE	G	NA	Inf	1.67E-06	0	0	0.285741547	0.078886737
chr14:22875639	rs35043211	LRP10_R231W	C	T	LRP10	0.897	1.065452935	0.408431118	2.779391448	.	Not_PD_known	.	TRUE	I	0.680545211	2.85E-05	3.33E-06	0.007413796	0.035093011	0.166085259	0.439651622
chr3:184322583	rs111924994	EIF4G1_A550P	G	C	EIF4G1	0.4522	1.088281667	0.872758844	1.357026623	not_provided	Not_PD_known	Likely_benign	TRUE	I	0.843979657	0.000357965	2.50E-05	0.012537418	0.020951767	0.035007705	0.999966526
chr1:16986334	rs151181674	ATP13A2_R1043H	C	T	ATP13A2	0.5675	1.764204711	0.251985433	12.35158012	Kufor-Rakeb_syr	Not_PD_known	Benign/Likely_benign	TRUE	I	0.565776944	NA	0	NA	NA	NA	0.189062848
chr1:155235772	rs80356769	GBA1_V433L	C	A	GBA1	0.0001581	4.17744577	1.989803719	8.770238491	Lewy_body_dem	PD_known	Pathogenic/Likely_pathogenic	TRUE	G	NA	0	0	0.04905274	0.109452871	0.244211989	0.125728477
chr22:38140006	rs147924368	PLA2G6_S258L	G	A	PLA2G6	0.6244	1.121873438	0.70793429	1.777848633	PLA2G6-associa	Not_PD_known	Conflicting_interpretations_of_pathogenicity	TRUE	I	0.813010931	NA	5.00E-06	NA	NA	NA	0.757296083
chrX:121049176	rs9697983	GLUD2_S498A	T	G	GLUD2	0.443	1.018366629	0.972143356	1.066787718	Parkinson_disea	PD_known	Pathogenic	TRUE	I	0.979696095	0.005725	0.000381667	0.015418279	0.02	0.025931005	1
chr12:40322037	rs11564176	LRRK2_R1725X	C	T	LRRK2	0.7075	57.13403508	3.79E-08	86060467793	Parkinson_disea	PD_known	Uncertain_significance	FALSE	G	NA	NA	0	0	0	0.091837281	0.140167046
chr1:20644651	rs74315359	PINK1_T313M	C	T	PINK1	0.4684	1.300306492	0.639469725	2.644061019	Parkinson_disea	PD_known	Pathogenic	FALSE	G	NA	NA	3.33E-06	NA	NA	NA	0.541362119
chr2:232811255	rs74560358	GIGYF2_A637V	C	T	GIGYF2	0.9966	1.004811538	0.109292883	9.237986956	.	Not_PD_known	.	TRUE	I	0.846997917	NA	0	0	NA	1	0.140779425
chr3:132499777	rs61748103	DNAJC13_R1462H	G	A	DNAJC13	0.2626	1.048855951	0.9648371	1.140191237	not_specified|not	Not_PD_known	Benign	TRUE	I	0.824703813	0.002402246	0.000178333	0.018405204	0.022270828	0.026944026	1
chr15:61910283	rs10851704	VPS13C_S2913N	C	T	VPS13C	0.4049	1.007628953	0.98981621	1.025762255	not_provided	Not_PD_known	Benign	TRUE	I	0.999265552	NA	0.007931667	NA	NA	NA	1
chr15:89325456	rs796052906	POLG_P648R	G	C	POLG	0.7155	1.406915895	0.224265648	8.826194961	Progressive_scle	Not_PD_known	Pathogenic/Likely_pathogenic	FALSE	G	NA	0	0	0.003038637	0.020874352	0.143387118	0.104399487
chr15:89323460	rs121918054	POLG_G737R	C	G	POLG	0.8891	1.013591536	0.83891994	1.224631522	Seizures|Progres	Not_PD_known	Pathogenic/Likely_pathogenic	TRUE	I	0.979261816	0.000323669	3.00E-05	0.020958223	0.027806144	0.036888618	0.999998393
chr14:22876113	rs754181235	LRP10_D389N	G	A	LRP10	0.1124	1.549759876	0.90225328	2.66195283	.	Not_PD_known	.	TRUE	I	0.705324352	2.74E-05	3.33E-06	0.008933355	0.036555219	0.14955971	0.672166044
chr3:132474976	rs137948208	DNAJC13_K779R	A	G	DNAJC13	0.3934	1.787825362	0.47087058	6.788106251	not_provided	Not_PD_known	Likely_benign	TRUE	I	0.763221204	NA	0	0	0	1	0.137404483
chr12:40278179	rs71653639	LRRK2_N720S	A	G	LRRK2	0.3914	1.41637388	0.638996693	3.139476292	.	Not_PD_known	.	TRUE	I	0.609462321	2.04E-05	1.67E-06	0.004331203	0.024546425	0.139098259	0.278623248
chr12:40251480	rs202170315	LRRK2_A373T	G	A	LRRK2	0.9015	1892.019394	2.55E-49	1.40E+55	.	Not_PD_known	.	FALSE	I	0.351182252	NA	0	0	NA	1	0.051901042
chr15:61941806	rs73430435	VPS13C_I1804V	T	C	VPS13C	0.004488	5.906775017	1.735160645	20.10764317	not_provided	Not_PD_known	Benign	TRUE	I	0.552050889	NA	0	NA	NA	NA	0.143353696
chr15:89333177	rs3176162	POLG_R193Q	C	T	POLG	0.4887	7.75238083	0.023520662	2555.175017	Seizures|Progres	Not_PD_known	Uncertain_significance	FALSE	I	0.398368984	NA	0	0	NA	1	0.056561891
chr15:61919368	rs568860952	VPS13C_A2520G	G	C	VPS13C	0.9448	15.75408978	2.09E-33	1.19E+35	.	Not_PD_known	.	FALSE	I	0.461085111	NA	NA	NA	NA	NA	0.051089632
chr21:32678644	rs748818427	NA	C	A	SYNJ1	0.953	2.732726968	8.20E-15	9.11E+14	.	Not_PD_known	.	FALSE	G	NA	NA	0	0	0	1	0.051939141
chr15:61962390	rs147306617	VPS13C_K1195T	T	G	VPS13C	0.08889	6.58569584	0.750824007	57.76505453	not_provided	Not_PD_known	Benign	FALSE	I	0.431152999	NA	1.67E-06	NA	NA	NA	0.24483907
chr21:32639773	rs760863245	SYNJ1_P1238S	G	A	SYNJ1	0.6002	3.179741805	0.042032506	240.5461589	not_provided	Not_PD_known	Uncertain_significance	FALSE	I	NA	NA	0	0	0	0.614327251	NA
chr22:38143150	rs185396488	PLA2G6_P25L	G	A	PLA2G6	0.3462	1.577750345	0.610891447	4.07485841	Infantile_neuroax	Not_PD_known	Likely_benign	TRUE	I	0.678933024	NA	1.67E-06	NA	NA	NA	0.460384512
chr5:1414780	rs577802449	SLC6A3_T356M	G	A	SLC6A3	0.6148	1.238374955	0.53858605	2.847404849	Parkinsonism-dy	PD_known	Uncertain_significance	TRUE	I	0.85860759	NA	0	8.94E-17	0	1	0.167948083
chr1:155236399	rs78188205	GBA1_A357D	G	T	GBA1	0.8596	7.4984775	1.50E-09	37460317139	.	Not_PD_known	.	FALSE	G	NA	NA	0	4.66E-17	0	1	0.065729564
chr21:32643457	rs142964720	SYNJ1_G1183D	C	T	SYNJ1	0.8022	6.53E+125	0	Inf	Parkinson_disea	PD_known	Uncertain_significance	FALSE	I	0.561128676	NA	0	0	NA	1	0.052235667
chr3:132522950	rs147575919	DNAJC13_D1932E	T	G	DNAJC13	0.3261	1.187202857	0.842814831	1.672313505	.	Not_PD_known	.	TRUE	I	0.939847648	0.000190432	1.33E-05	0.010401324	0.021004828	0.042411152	0.987182645
chr1:20649054	rs74315356	PINK1_W437X	G	A	PINK1	0.7463	7.689840176	3.30E-05	1790654.941	Parkinson_disea	PD_known	Pathogenic	TRUE	G	NA	NA	0	NA	NA	NA	0.06974287
chr15:89318986	rs267606959	POLG_P1073L	G	A	POLG	0.5006	4.203846754	0.064407577	274.3827419	Progressive_scle	Not_PD_known	Pathogenic	TRUE	G	NA	NA	0	0	0	0.174048317	0.089758391
chr22:38123197	rs587784331	PLA2G6_L497F	G	A	PLA2G6	0.9036	2.745875589	2.18E-07	34568727.44	Iron_accumulatic	Not_PD_known	Likely_pathogenic	TRUE	G	NA	NA	0	NA	NA	NA	0.052321909
chr6:161785805	rs72480422	PRKN_D280N	C	T	PRKN	0.5297	1.516492345	0.413912782	5.556119873	Parkinson_disea	PD_known	Uncertain_significance	TRUE	G	NA	NA	1.67E-06	NA	NA	NA	0.107395958
chr14:22877184	rs139012932	LRP10_R600H	G	A	LRP10	0.6484	1.605925137	0.209684345	12.29941869	.	Not_PD_known	.	TRUE	I	0.524148226	NA	0	0	0	0.682653786	0.129366295

chr12:40335031	rs77428810	LRRK2_R1941H	G	A	LRRK2	0.7508	1.097351995	0.618414707	1.947206926	Parkinson_disea	PD_known	Uncertain_significance	TRUE	I	0.677721858	8.54E-06	1.67E-06	0.018898623	0.058547088	0.181356939	0.336498048
chr15:61920571	rs139993005	VPS13C_H2380R	T	C	VPS13C	0.3253	1.070365308	0.934787545	1.225606716	not_provided	Not_PD_known	Benign	TRUE	I	0.87374264	NA	6.33E-05	NA	NA	NA	1
chr6:161548861	rs201300874	PRKN_G359D	C	T	PRKN	0.7675	7.886875115	8.92E-06	6970322.142	.	Not_PD_known	.	TRUE	G	NA	NA	0	NA	NA	NA	0.055537255
chr1:20639934	rs573931674	PINK1_E240K	G	A	PINK1	0.984	2.721273584	1.16E-42	6.41E+42	.	Not_PD_known	.	FALSE	G	NA	NA	0	NA	NA	NA	0.051259385
chr1:20644564	rs113092523	PINK1_S284Y	C	A	PINK1	0.6395	66.77975729	1.55E-06	2879930503	not_specified	Not_PD_known	Uncertain_significance	TRUE	I	0.543890774	NA	0	NA	NA	NA	0.091068961
chr22:32498414	rs141286570	FBXO7_V485I	G	A	FBXO7	0.215	1.23182893	0.886055225	1.712537177	Parkinson_disea	PD_known	Uncertain_significance	TRUE	I	0.641458869	NA	1.33E-05	NA	NA	NA	0.987233278
chr1:17000272	rs56367069	ATP13A2_R289Q	C	T	ATP13A2	0.2267	1.039250728	0.976453645	1.106086379	Kufor-Rakeb_syr	Not_PD_known	Benign/Likely_benign	TRUE	I	0.999649882	NA	0.000281667	NA	NA	NA	1
chr22:38145597	rs142715413	PLA2G6_S89Y	G	T	PLA2G6	0.709	2.367654967	0.025621383	218.7934207	PLA2G6-associa	Not_PD_known	Conflicting_interpretations_of_pathogenicity	FALSE	I	0.311267853	NA	0	NA	NA	NA	0.092633077
chr1:155235819	rs754743440	GBA1_W417X	C	T	GBA1	0.8537	22.62148232	9.12E-14	5.61E+15	Gaucher_disease	Not_PD_known	Likely_pathogenic	FALSE	G	NA	NA	0	0	0	1	0.052607361
chr12:40299125	rs34805604	LRRK2_I1122V	A	G	LRRK2	0.8885	2.748622838	2.01E-06	3763048.658	Parkinson_disea	PD_known	Pathogenic	TRUE	G	NA	NA	0	0	0	1	0.053055995
chr1:155236269	rs149487315	GBA1_M400I	C	T	GBA1	0.8952	12.04319777	9.92E-16	1.46E+17	Hepatoblastoma	Not_PD_known	Uncertain_significance	FALSE	I	0.654259861	NA	0	0	NA	1	0.053168581
chr15:61983936	rs116507802	VPS13C_I600V	T	C	VPS13C	0.3643	16.48086482	0.038679869	7022.229225	.	Not_PD_known	.	TRUE	I	0.521835089	NA	0	NA	NA	NA	0.161126472
chr20:5069288	rs186368464	TMEM230_R95H	C	T	TMEM230	0.8717	1.02716235	0.742322124	1.421300077	.	Not_PD_known	.	TRUE	I	0.583716929	0.000239153	1.83E-05	0.013038649	0.022997847	0.040557596	0.996919474
chr1:17000107	rs150519745	ATP13A2_G310R	C	T	ATP13A2	0.2955	3.682161707	0.320247867	42.33694038	Kufor-Rakeb_syr	Not_PD_known	Likely_pathogenic	TRUE	G	NA	NA	1.67E-06	NA	NA	NA	0.109088874
chr6:162443357	rs577232474	PRKN_R42C	G	A	PRKN	0.5377	3.471198766	0.06631574	181.6947352	not_provided	Not_PD_known	Likely_benign	FALSE	G	0.322159559	NA	0	NA	NA	NA	0.069159988
chr3:132522832	rs139626514	DNAJC13_R1893Q	G	A	DNAJC13	0.7636	1.581857833	0.079627163	31.42488202	.	Not_PD_known	.	FALSE	I	NA	0	0	0.002359842	0.048744555	1	NA
chr15:61931104	rs78071599	VPS13C_E2008D	C	G	VPS13C	0.4357	1.023983077	0.964941308	1.086637428	.	Not_PD_known	.	TRUE	I	0.939700842	NA	0.000325	NA	NA	NA	1
chr15:61918208	rs201120398	VPS13C_N2563S	T	C	VPS13C	0.7597	1.085672923	0.641301227	1.83795952	.	Not_PD_known	.	TRUE	I	0.706382751	NA	5.00E-06	NA	NA	NA	0.675658814
chr1:155235765	rs75385858	GBA1_N435T	T	G	GBA1	0.9742	2.726176287	1.11E-26	6.68E+26	.	Not_PD_known	.	FALSE	G	NA	NA	0	0	0	1	0.051547699
chr15:61984980	rs35435766	VPS13C_T533N	G	T	VPS13C	0.08262	6.067216905	0.792191598	46.46744686	not_provided	Not_PD_known	Likely_benign	TRUE	I	0.901482821	NA	0	NA	NA	NA	0.064484126
chr1:20644549	rs74315358	PINK1_R279H	G	A	PINK1	0.01244	2.542886873	1.223157952	5.286540171	Parkinson_disea	PD_known	Pathogenic	TRUE	G	NA	NA	1.67E-06	NA	NA	NA	0.499089743
chr1:16991787	rs201883464	ATP13A2_T728M	G	A	ATP13A2	0.6959	20.5672499	5.36E-06	78948002.7	Kufor-Rakeb_syr	Not_PD_known	Conflicting_interpretations_of_pathogenicity	TRUE	G	NA	NA	0	NA	NA	NA	0.087272953
chr22:38112534	rs587784351	PLA2G6_W749S	C	G	PLA2G6	0.01731	866.9662078	3.299265936	227817.4661	Iron_accumulatic	Not_PD_known	Likely_pathogenic	TRUE	G	NA	NA	0	NA	NA	NA	0.078922126
chr1:65386886	rs201840876	DNAJC6_M300T	T	C	DNAJC6	0.8837	1.038211997	0.628600097	1.714737487	not_provided	Not_PD_known	Uncertain_significance	TRUE	I	0.703989565	NA	5.00E-06	NA	NA	NA	0.801879866
chr1:155235197	.	GBA1_N501K	G	C	GBA1	0.7568	2.992472593	0.002907284	3080.157466	Gaucher_disease	Not_PD_known	Likely_pathogenic	FALSE	G	NA	NA	0	0	0	1	0.05589892
chr22:38112547	rs587784350	PLA2G6_R745W	G	A	PLA2G6	0.003736	20.27320371	2.651729318	154.994247	Iron_accumulatic	Not_PD_known	Pathogenic	TRUE	G	NA	NA	0	NA	NA	NA	0.054553566
chr15:62010533	rs529383497	VPS13C_T317M	G	A	VPS13C	0.9435	1.032517505	0.425909669	2.503095085	.	Not_PD_known	.	TRUE	I	0.56936872	NA	3.33E-06	NA	NA	NA	0.499342355
chr15:61991096	rs143639809	NA	T	C	VPS13C	0.09005	1.267567868	0.963575918	1.667464151	not_provided	Not_PD_known	Benign	TRUE	I	0.753814876	NA	1.83E-05	NA	NA	NA	0.998483124
chr22:32498466	rs144538200	FBXO7_N502S	A	G	FBXO7	0.4638	3.6995086	0.111740507	122.4834599	Parkinson_disea	PD_known	Uncertain_significance	FALSE	I	0.574417293	NA	0	NA	NA	NA	0.07424746
chr12:40308481	rs72546338	LRRK2_R1325Q	G	A	LRRK2	0.0367	1.513916498	1.025971532	2.233924714	Parkinson_disea	PD_known	Conflicting_interpretations_of_pathogenicity	TRUE	I	0.792934299	4.36E-05	6.67E-06	0.029056233	0.045888514	0.072467439	0.651488059
chr1:65385740	rs146050826	DNAJC6_A220T	G	A	DNAJC6	0.5349	1.131563155	0.765801715	1.672019204	not_provided	Not_PD_known	Uncertain_significance	TRUE	I	0.702057421	NA	1.00E-05	NA	NA	NA	0.973226462
chr15:89333357	rs540905618	POLG_D133G	T	C	POLG	0.6285	1.3499938	0.40031962	4.552570416	Progressive_scle	Not_PD_known	Uncertain_significance	TRUE	I	0.502586424	1.80E-05	1.67E-06	0.003700613	0.027854764	0.209623676	0.307954532
chr15:89320944	rs760431841	POLG_T935P	T	G	POLG	0.9028	2.771531346	2.16E-07	35498748.49	.	Not_PD_known	.	TRUE	G	NA	NA	0	0	0	1	0.052561017
chr3:184323468	rs111396765	EIF4G1_A718P	G	C	EIF4G1	0.8207	1.027367803	0.813159501	1.298004389	not_provided	Not_PD_known	Likely_benign	TRUE	I	0.826265156	0.000307776	2.50E-05	0.015208859	0.024368346	0.039037852	0.999860756
chr22:38113621	rs141777179	PLA2G6_V690I	C	T	PLA2G6	0.5743	1.109156703	0.77257978	1.592364471	Iron_accumulatic	Not_PD_known	Uncertain_significance	TRUE	I	0.75681603	NA	1.17E-05	NA	NA	NA	0.988911866
chr21:32701973	rs372488661	SYNJ1_R106W	G	A	SYNJ1	0.5469	3.907899432	0.046324329	329.6686305	Parkinson_disea	PD_known	Uncertain_significance	FALSE	I	NA	NA	0	0	0	1	NA
chr22:38132881	rs11570680	PLA2G6_A343T	C	T	PLA2G6	0.0749	1.0681199	0.993401864	1.148457801	PLA2G6-associa	Not_PD_known	Benign/Likely_benign	TRUE	I	0.898242235	NA	0.000215	NA	NA	NA	1
chr22:32498453	rs121918304	FBXO7_R498X	C	T	FBXO7	0.365	2.449539383	0.352558046	17.01916394	Parkinson_disea	PD_known	Pathogenic	TRUE	G	NA	NA	0	NA	NA	NA	0.106563166
chr12:40294866	rs17519916	LRRK2_D944Y	G	T	LRRK2	0.5892	3.956263424	0.026883784	582.2104615	Parkinson_disea	PD_known	Uncertain_significance	TRUE	G	NA	NA	0	0	0	0.174100618	0.078239256
chr12:40323270	rs281865054	LRRK2_E1874X	G	T	LRRK2	0.94	2.729995607	1.20E-11	6.21E+11	Parkinson_disea	PD_known	Pathogenic	FALSE	G	NA	NA	0	0	0	1	0.056543884
chr2:232794852	rs115337999	GIGYF2_R463W	C	T	GIGYF2	0.562	3.556937778	0.048773897	259.3970761	.	Not_PD_known	.	TRUE	G	NA	NA	0	0	0	0.488232262	0.056907437
chr21:32664963	rs145978776	SYNJ1_D791Y	C	A	SYNJ1	0.7922	1.051586525	0.723348611	1.528770779	Parkinson_disea	PD_known	Uncertain_significance	TRUE	I	0.78400594	0.000163837	8.33E-06	0.006899781	0.015259103	0.033740629	0.811128034
chr1:17000495	rs199661793	ATP13A2_A244T	C	T	ATP13A2	0.009355	25.90147715	2.225514222	301.4523619	Kufor-Rakeb_syr	Not_PD_known	Uncertain_significance	FALSE	I	0.475601226	NA	0	NA	NA	NA	0.220833217
chr2:74530433	rs387906942	HTRA2_P143A	C	G	HTRA2	0.7354	3.030416291	0.004885557	1879.708518	Parkinson_disea	PD_known	risk_factor	FALSE	G	NA	0	0	0.052088097	1	1	0.050203754
chr12:40319998	rs281865051	LRRK2_V1613A	T	C	LRRK2	0.9534	2.733820277	6.34E-15	1.18E+15	Parkinson_disea	PD_known	Uncertain_significance	FALSE	G	NA	NA	0	9.23E-17	0	1	0.051333554
chr6:161548937	rs199657839	PRKN_R334C	G	A	PRKN	0.1396	3.778775442	0.647650552	22.04760546	Parkinson_disea	PD_known	Conflicting_interpretations_of_pathogenicity	TRUE	I	0.597688556	NA	0	NA	NA	NA	0.120614848
chr15:61936716	rs114590764	VPS13C_M1879R	A	C	VPS13C	0.6622	40.94378384	2.40E-06	699124922.3	.	Not_PD_known	.	TRUE	I	0.891901672	NA	0	NA	NA	NA	0.103364224
chr1:7965399	rs114601558	PARK7_A56T	G	A	PARK7	0.4605	1.51725078	0.501423613	4.591028165	not_provided	Not_PD_known	Benign/Likely_benign	TRUE	I	0.854736984	NA	1.67E-06	0	NA	1	0.228468966
chr15:61977090	rs76341351	VPS13C_R800S	T	G	VPS13C	0.8016	8.86E+100	0	Inf	not_provided	Not_PD_known	Benign	FALSE	I	0.371590465	NA	0	NA	NA	NA	0.062374345
chr15:89327006	rs121918052	POLG_Q497H	C	G	POLG	0.4925	1.817387445	0.330025947	10.00799226	.	Not_PD_known	.	FALSE	G	NA	6.43E-05	1.67E-06	0.001211275	0.007774444	0.049893999	0.280005736
chr22:38120802	rs587784337	PLA2G6_E567K	C	T	PLA2G6	0.03325	8.47567249	1.185010932	60.62140206	Iron_accumulatic	Not_PD_known	Pathogenic	TRUE	G	NA	NA	0	NA	NA	NA	0.054377879
chr3:184327628	rs199929867	EIF4G1_A1236V	C	T	EIF4G1	0.3857	3.536720906	0.203731567	61.39644902	.	Not_PD_known	.	FALSE	I	NA	NA	0	0	0	0.240700408	NA
chr15:89333723	rs765472726	POLG_G11D	C	T	POLG	0.489	1.14602565	0.77894098	1.686103086	.	Not_PD_known	.	TRUE	I	0.8457973	0.000168129	8.33E-06	0.006263647	0.014869554	0.03529385	0.913190322
chr6:161569358	rs72480423	PRKN_E310D	C	G	PRKN	0.108	1.442676401	0.922584784	2.255960896	Parkinson_disea	PD_known	Uncertain_significance	TRUE	I	0.723047495	NA	1.00E-05	NA	NA	NA	0.817850264
chr15:89318736	rs368435864	POLG_R1096H	C	T	POLG	0.7251	20.65587949	9.69E-07	440233576.6	Seizures|Progres	Not_PD_known	Conflicting_interpretations_of_pathogenicity	TRUE	G	0.448620319	NA	0	8.09E-18	0	0.167870478	0.083648073
chr1:20648577	rs119451946	PINK1_P399L	C	T	PINK1	0.915	2.744502994	2.41E-08	312347841.1	Parkinson_disea	PD_known	Uncertain_significance	TRUE	G	NA	NA	0	NA	NA	NA	0.051489442
chr1:155239633	rs758447515	GBA1_S146L	G	A	GBA1	1.31E-05	1624.734993	58.43762815	45172.32954	Gaucher_disease	Not_PD_known	Conflicting_interpretations_of_pathogenicity	FALSE	G	NA	0	0	0.194931919	1	1	0.050654458
chr22:38115619	rs794729212	PLA2G6_G648R	C	T	PLA2G6	0.03035	15.22288671	1.29497579	178.9502797	.	Not_PD_known	.	TRUE	G	NA	NA	0	NA	NA	NA	0.051832007
chr14:22877106	rs200149859	LRP10_R574Q	G	A	LRP10	0.4789	3.496631233	0.109319554	111.8411991	.	Not_PD_known	.	FALSE	I	NA	NA	0	0	0	0.682599965	0.241912794
chr1:155235196	rs80356771	GBA1_R502C	G	A	GBA1	2.15E-13	3.798856119	2.660122036	5.425054796	Lewy_body_dem	PD_known	Pathogenic	FALSE	G	NA	1.46E-05	3.33E-06	0.048324794	0.068304611	0.096539342	0.596000392
chr15:61856407	rs146072191	VPS13C_R3652Q	C	T	VPS13C	0.02473	1.805612736	1.078125624	3.023986518	.	Not_PD_known	.	TRUE	I	0.761567056	NA	6.67E-06	NA	NA	NA	0.826470623
chr22:32484066	rs548204763	FBXO7_N196S	A	G	FBXO7	0.7777	1.230105576	0.29212894	5.179766614	.	Not_PD_known	.	TRUE	I	0.547830403	NA	1.67E-06	NA	NA	NA	0.312305875
chr12:40308507	rs201177073	LRRK2_R1334X	C	T	LRRK2	0.01495	14.92593046	1.692366762	131.6401416	.	Not_PD_known	.	TRUE	G	NA	0	0	0.009698748	0.0983917	0.998007738	0.076191421
chr15:61882609	rs142572498	VPS13C_L3204S	A	G	VPS13C	0.04666	1.878549619	1.009420092	3.49601588	.	Not_PD_known	.	TRUE	I	0.668867409	NA	5.00E-06	NA	NA	NA	0.63836101
chr12:40293552	rs148113070	LRRK2_E899D	A	C	LRRK2	0.8727	1.490035582	0.01134861	195.6368237	.	Not_PD_known	.	TRUE	I	0.709278047	NA	0	0	NA	1	0.106522456
chr22:38132917	rs199935023	PLA2G6_D331N	C	T	PLA2G6	0.2438	2.303201243	0.566390222	9.36586784	PLA2G6-associa	Not_PD_known	Conflicting_interpretations_of_pathogenicity	FALSE	I	0.328313321	NA	1.67E-06	NA	NA	NA	0.331730469
chr3:132528316	rs140537885	DNAJC13_L2170W	T	G	DNAJC13	0.07663	1.11717145	0.988174478	1.263007776	.	Not_PD_known	.	TRUE	I	0.979786277	0.00082867	7.50E-05	0.022653935	0.027151953	0.032540469	1
chr2:232819840	rs200601366	GIGYF2_R795H	G	A	GIGYF2	0.07944	2.276638471	0.907795633	5.709525955	.	Not_PD_known	.	TRUE	I	0.818051696	Inf	1.67E-06	0	0	0.049278045	0.440604793
chr22:38169318	rs200075782	PLA2G6_R37X	G	A	PLA2G6	0.8683	1.1443079	0.23245189	5.633168092	Iron_accumulatic	Not_PD_known	Pathogenic	TRUE	G	NA	NA	1.67E-06	NA	NA	NA	0.230693428
chr12:40263875	rs79996249	LRRK2_K544E	A	G	LRRK2	0.7466	58.18921335	1.15E-09	2.94E+12	Parkinson_disea	PD_known	Uncertain_significance	FALSE	G	NA	NA	0	0	0	0.416628389	0.085468354
chr3:132450732	rs149480465	DNAJC13_N141I	A	T	DNAJC13	0.2692	1.582965521	0.700842567	3.575381916	.	Not_PD_known	.	FALSE	I	0.466368824	2.61E-05	3.33E-06	0.011445686	0.0383792	0.128670928	0.46760373
chr1:16986554	rs201756175	ATP13A2_P1105L	G	A	ATP13A2	0.4191	1.150273799	0.819002617	1.615537955	Kufor-Rakeb_syr	Not_PD_known	Conflicting_interpretations_of_pathogenicity	TRUE	I	0.703794599	NA	1.00E-05	NA	NA	NA	0.978771643
chr14:22875363	rs28534929	LRP10_M139V	A	G	LRP10	0.2657	1.240730104	0.848616417	1.814024758	not_provided	Not_PD_known	Benign	TRUE	I	0.825330555	0.000127729	1.00E-05	0.002192636	0.02348725	0.251448744	0.971951912
chr15:89318676	rs764036283	POLG_M1116T	A	G	POLG	0.9056	2.737102831	1.61E-07	46490009.59	not_provided	Not_PD_known	Conflicting_interpretations_of_pathogenicity	TRUE	G	NA	NA	0	0	0	1	0.055009869
chr5:1394741	rs200712598	SLC6A3_K619N	C	G	SLC6A3	0.06654	1.477719469	0.973762349	2.242492566	Parkinsonism-dy	PD_known	Uncertain_significance	TRUE	I	0.69238615	8.29E-05	6.67E-06	0.009896299	0.024126445	0.058809065	0.775727161
chr15:89321223	rs368587966	POLG_Q879R	T	C	POLG	0.9124	2.733546909	4.52E-08	165212651.7	Progressive_scle	Not_PD_known	Conflicting_interpretations_of_pathogenicity	TRUE	G	0.301898956	NA	0	3.81E-17	0	1	0.052367059
chr15:89330257	rs121918056	POLG_R227W	G	A	POLG	0.05326	6.971288002	0.973151018	49.93968614	Abnormality_of_	Not_PD_known	Pathogenic	TRUE	G	NA	0	0	0.005883307	0.05337816	0.484233952	0.085411207
chr1:20648528	rs45515602	PINK1_A383T	G	A	PINK1	0.947	1.026033092	0.480457742	2.191126949	Parkinson_disea	PD_known	Conflicting_interpretations_of_pathogenicity	TRUE	I	0.699574769	NA	5.00E-06	NA	NA	NA	0.695760892

chr22:38112541	rs121908687	PLA2G6_R747W	G	A	PLA2G6	0.8016	7.494729199	1.12E-06	50038536.67	Neurodegenerati	PD_known	Pathogenic/Likely_pathogenic	TRUE	G	NA	NA	0	NA	NA	NA	0.065711065
chr15:89320907	rs796052891	POLG_K947R	T	C	POLG	0.9668	2.722906838	8.54E-21	8.68E+20	not_provided	Not_PD_known	Pathogenic	FALSE	G	NA	NA	0	0	0	1	0.052549686
chr14:22875648	rs371755191	LRP10_V234M	G	A	LRP10	0.9399	1.033550539	0.43802469	2.438736311	.	Not_PD_known	.	FALSE	I	0.430078834	5.24E-05	3.33E-06	0.00542944	0.019072288	0.066985527	0.379249547
chr1:155236331	rs781306264	GBA1_A380T	C	T	GBA1	0.9831	2.721545725	1.39E-40	5.34E+40	.	Not_PD_known	.	FALSE	G	NA	NA	0	0	0	1	0.05252546
chr1:17004708	rs376169767	ATP13A2_A154V	G	A	ATP13A2	0.5271	3.148417671	0.09005236	110.0752255	Kufor-Rakeb_syr	Not_PD_known	Uncertain_significance	FALSE	I	NA	NA	0	NA	NA	NA	0.936808247
chr3:132466351	rs199541720	DNAJC13_D674A	A	C	DNAJC13	0.8503	1.042164708	0.678985165	1.599603842	.	Not_PD_known	.	TRUE	I	0.908252597	0.000104213	6.67E-06	0.00658658	0.01919149	0.055909794	0.868341097
chr3:132523168	rs61731474	DNAJC13_N1952T	A	C	DNAJC13	0.8535	1.129754102	0.3094459	4.124612187	not_provided	Not_PD_known	Benign	TRUE	I	0.79691714	Inf	1.67E-06	0	0	0.61434238	0.253205877
chr21:32656885	rs114053718	SYNJ1_I905T	A	G	SYNJ1	0.4432	2.033584501	0.331352671	12.48055708	Parkinson_disea	PD_known	Uncertain_significance	TRUE	I	0.600566804	NA	0	0	0	1	0.112470612
chr1:155238209	rs75636769	GBA1_A229E	G	T	GBA1	0.8646	7.479006782	6.78E-10	82509559805	.	Not_PD_known	.	FALSE	G	NA	NA	0	0	0	1	0.087724966
chr15:61922557	rs141890160	VPS13C_I2272T	A	G	VPS13C	0.5272	1.095269005	0.82593434	1.452432882	.	Not_PD_known	.	TRUE	I	0.662195742	NA	1.67E-05	NA	NA	NA	0.99480579
chr15:89320998	rs796052890	POLG_G917R	C	T	POLG	0.9673	2.727539716	4.03E-21	1.85E+21	not_provided	Not_PD_known	Likely_pathogenic	FALSE	G	NA	NA	0	0	0	1	0.050633326
chr12:40257264	rs71653637	LRRK2_I435M	A	G	LRRK2	0.8503	20.50974215	4.84E-13	8.70E+14	.	Not_PD_known	.	FALSE	G	NA	NA	0	0	0	1	0.063816546
chr22:38132914	rs587784364	PLA2G6_C332R	A	G	PLA2G6	0.8893	2.755502992	1.73E-06	4377568.538	Iron_accumulatic	Not_PD_known	Likely_pathogenic	TRUE	G	NA	NA	0	NA	NA	NA	0.05284612
chr3:184327694	rs73053766	EIF4G1_N1258S	A	G	EIF4G1	0.4244	3.551961549	0.158431651	79.63327236	.	Not_PD_known	.	FALSE	I	0.412814617	NA	0	0	0	0.240724114	0.06280519
chr16:46682118	rs186122975	VPS35_R54W	G	A	VPS35	0.9457	2.745326469	6.60E-13	1.14E+13	.	Not_PD_known	.	FALSE	G	NA	NA	0	0	0	1	0.050329984
chr1:155235195	rs80356772	GBA1_R502H	C	T	GBA1	0.2251	3.58407349	0.455749483	28.18562229	Gaucher_disease	Not_PD_known	Conflicting_interpretations_of_pathogenicity	FALSE	G	NA	0	0	0.007341981	0.056631819	0.436794132	0.069699836
chr1:155235006	rs146519305	GBA1_R534C	G	A	GBA1	0.7072	11.32259175	3.59E-05	3573957.203	.	Not_PD_known	.	TRUE	I	0.782674909	NA	NA	NA	NA	NA	0.066315785
chr15:62008730	rs138433394	VPS13C_V348A	A	G	VPS13C	0.7684	1.092097326	0.607662287	1.962729291	.	Not_PD_known	.	TRUE	I	0.580149412	NA	6.67E-06	NA	NA	NA	0.314741995
chr3:184317393	rs201980563	EIF4G1_P74S	C	T	EIF4G1	0.9083	2.316135352	1.44E-06	3713613.686	.	Not_PD_known	.	TRUE	G	NA	NA	0	0	0	1	0.054210046
chr22:38169423	rs587784356	PLA2G6_Q2K	G	T	PLA2G6	0.7792	7.599633329	5.28E-06	10939777.32	Iron_accumulatic	Not_PD_known	Likely_pathogenic	TRUE	G	NA	NA	0	NA	NA	NA	0.077194118
chr12:40363410	rs75695264	LRRK2_Y2346C	A	G	LRRK2	0.4411	1.736202054	0.426538776	7.067112637	.	Not_PD_known	.	FALSE	G	NA	0.000347142	6.67E-06	0.001454361	0.005761331	0.02281772	0.863839958
chr4:41263258	rs564599222	UCHL1_F165L	T	C	UCHL1	0.7629	6.835297533	2.57E-05	1815384.774	.	Not_PD_known	.	FALSE	I	0.390688241	NA	0	0	NA	1	0.051513779
chr15:89316763	rs3087374	POLG_Q1236H	C	A	POLG	0.5824	1.008536228	0.978357631	1.039645719	Seizures|Progres	Not_PD_known	Benign/Likely_benign	TRUE	I	1.000000119	0.016438809	0.001333333	0.02343776	0.024332663	0.025260273	1
chr15:61922408	rs12907567	VPS13C_V2322M	C	T	VPS13C	0.3156	1.015417644	0.985419342	1.046329159	not_provided	Not_PD_known	Benign	TRUE	I	1.000000119	NA	0.001305	NA	NA	NA	1
chr6:161360187	rs539917500	PRKN_R396G	T	C	PRKN	0.9396	2.761847942	1.05E-11	7.25E+11	.	Not_PD_known	.	FALSE	G	NA	NA	0	NA	NA	NA	0.051200442
chr6:162262681	rs747891099	PRKN_D86N	C	T	PRKN	0.5458	3.481976179	0.060762649	199.5330726	.	Not_PD_known	.	FALSE	G	NA	NA	0	NA	NA	NA	0.081921729
chr15:62013943	rs116305045	VPS13C_I245T	A	G	VPS13C	0.7402	1.742812173	0.065369728	46.46484476	.	Not_PD_known	.	FALSE	I	0.30547002	NA	0	NA	NA	NA	0.167047197
chr12:40263861	rs55968234	LRRK2_K539R	A	G	LRRK2	0.8136	1.215918794	0.23927925	6.178799519	.	Not_PD_known	.	FALSE	G	0.449575275	1.69E-05	1.67E-06	0.00819976	0.029565622	0.106591432	0.192829128
chr15:89321780	rs144500145	POLG_R852C	G	A	POLG	0.1296	2.845389599	0.736015574	11.000096	.	Not_PD_known	.	FALSE	G	0.637592673	6.92E-06	3.33E-06	0.015192583	0.144475514	1	0.050839567
chr15:89318553	rs548076633	POLG_N1157S	T	C	POLG	0.7838	7.530037335	4.09E-06	13862473.51	Progressive_scle	Not_PD_known	Likely_pathogenic	TRUE	G	NA	NA	0	1.28E-17	0	0.307679827	0.068992598
chr22:38132850	rs587784326	PLA2G6_P353L	G	A	PLA2G6	0.3416	2.331938743	0.407346135	13.34967447	Iron_accumulatic	Not_PD_known	Conflicting_interpretations_of_pathogenicity	TRUE	G	NA	NA	0	NA	NA	NA	0.074275884
chr17:46010389	rs63751273	MAPT_P301L	C	T	MAPT	0.8278	2.739019474	0.000312556	24002.85355	Frontotemporal_(	PD_known	Pathogenic	TRUE	G	NA	NA	0	0	0	0.33066821	0.065485672
chr15:62035002	rs116290723	VPS13C_V80F	C	A	VPS13C	0.8332	88.61489867	6.58E-17	1.19E+20	.	Not_PD_known	.	FALSE	I	0.408132941	NA	0	NA	NA	NA	0.053519811
chr1:155235823	rs121908311	GBA1_G416S	C	T	GBA1	0.5083	2.955299502	0.119161925	73.29350526	Thrombocytopen	Not_PD_known	Pathogenic	FALSE	G	NA	0	0	0.000756555	0.004711527	0.029339733	0.248080883
chr1:16987157	rs145548316	ATP13A2_R947Q	C	T	ATP13A2	0.2577	1.505913966	0.741165005	3.059746288	Kufor-Rakeb_syr	Not_PD_known	Uncertain_significance	FALSE	G	NA	NA	3.33E-06	NA	NA	NA	0.541600203
chr15:61922680	rs371685718	VPS13C_T2231K	G	T	VPS13C	0.656	1.275961485	0.436653196	3.728537255	.	Not_PD_known	.	TRUE	I	0.554982662	NA	1.67E-06	NA	NA	NA	0.334763045
chr1:16986065	rs542270701	ATP13A2_V1133I	C	T	ATP13A2	0.7484	1.117506652	0.566746027	2.203493378	.	Not_PD_known	.	TRUE	I	0.519249678	NA	3.33E-06	NA	NA	NA	0.564728545
chr1:20644626	rs112600292	PINK1_P305A	C	G	PINK1	0.9217	6.992932527	9.96E-17	4.91E+17	.	Not_PD_known	.	FALSE	I	0.552645683	NA	0	NA	NA	NA	0.050965554
chr22:38123244	rs587784330	PLA2G6_L481Q	A	T	PLA2G6	0.9132	2.74230827	3.62E-08	207605042.8	Iron_accumulatic	Not_PD_known	Pathogenic	TRUE	G	NA	NA	0	NA	NA	NA	0.052547652
chr15:61915865	rs532971259	VPS13C_P2738R	G	C	VPS13C	0.8822	1.91E+22	3.86E-273	Inf	.	Not_PD_known	.	FALSE	I	0.381515145	NA	0	NA	NA	NA	0.05074394
chr21:32726855	rs565807712	SYNJ1_D53G	T	C	SYNJ1	0.9121	1.425895447	0.002620359	775.9157929	Parkinson_disea	PD_known	Uncertain_significance	FALSE	I	0.425793111	NA	0	0	NA	1	0.084583975
chr15:61940726	rs77555508	VPS13C_S1841F	G	A	VPS13C	0.8376	2.147275867	0.001438365	3205.578974	.	Not_PD_known	.	FALSE	I	0.35916853	NA	0	NA	NA	NA	0.067659143
chr3:184322551	rs561916314	EIF4G1_P539L	C	T	EIF4G1	0.4736	1.986749726	0.303922058	12.98745641	.	Not_PD_known	.	TRUE	I	0.763089299	Inf	1.67E-06	0	0	0.61434238	0.117768949
chr3:184323412	rs373584582	EIF4G1_G699A	G	C	EIF4G1	0.4997	1.209612427	0.695989991	2.102274807	.	Not_PD_known	.	TRUE	I	0.632680774	6.20E-05	5.00E-06	0.008540448	0.02420148	0.068569918	0.593568759
chr3:132516758	rs113742727	DNAJC13_R1872Q	G	A	DNAJC13	0.5383	37.56602307	0.000361886	3899582.289	.	Not_PD_known	.	TRUE	I	0.751726568	NA	0	1.70E-16	NA	1	0.155200425
chr1:16997107	rs142616130	ATP13A2_R365W	G	A	ATP13A2	0.004436	3.359190425	1.458096765	7.738965327	Kufor-Rakeb_syr	Not_PD_known	Uncertain_significance	FALSE	G	NA	NA	1.67E-06	NA	NA	NA	0.212655582
chr12:40351579	rs111691891	LRRK2_T2141M	C	T	LRRK2	0.8462	20.41765566	1.18E-12	3.52E+14	Parkinson_disea	PD_known	Uncertain_significance	FALSE	G	NA	NA	0	0	0	0.390481249	0.079801806
chr15:89333327	rs796052899	POLG_A143V	G	A	POLG	0.668	1.522113759	0.223280154	10.37633778	Progressive_scle	Not_PD_known	Conflicting_interpretations_of_pathogenicity	TRUE	G	NA	0	0	0.003201518	0.022122441	0.152852659	0.105818735
chr22:38126451	rs797045888	NA	T	C	PLA2G6	0.5565	1.85132204	0.237731831	14.41705672	Neurodegenerati	Not_PD_known	Pathogenic	TRUE	G	NA	NA	0	NA	NA	NA	0.08521805
chr1:16986291	rs544885605	ATP13A2_R1158H	C	T	ATP13A2	0.582	1.384722834	0.434548377	4.412529024	Kufor-Rakeb_syr	Not_PD_known	Uncertain_significance	FALSE	I	0.332303643	NA	3.33E-06	NA	NA	NA	0.268600399
chr15:61978646	rs140060073	VPS13C_Q757R	T	C	VPS13C	0.1625	1.222380271	0.922149427	1.620359438	.	Not_PD_known	.	FALSE	I	0.8930884	NA	1.33E-05	NA	NA	NA	0.990254585
chr15:89320856	rs767339769	POLG_R964H	C	T	POLG	0.907	2.746424819	1.20E-07	63072284.52	.	Not_PD_known	.	TRUE	G	NA	NA	0	0	0	1	0.053700361
chr1:155238214	rs381418	GBA1_N227K	A	C	GBA1	0.8665	56.54292024	2.13E-19	1.50E+22	Gaucher_disease	Not_PD_known	Pathogenic/Likely_pathogenic	FALSE	G	NA	NA	0	0	0	0.917025825	0.070278753
chr1:17000429	rs561097876	ATP13A2_I266V	T	C	ATP13A2	0.8224	384.2525428	1.05E-20	1.41E+25	Kufor-Rakeb_syr	Not_PD_known	Uncertain_significance	FALSE	I	0.451225489	NA	NA	NA	NA	NA	0.052808908
chr6:161785778	rs55961220	PRKN_C289G	A	C	PRKN	0.9632	2.731634096	7.82E-19	9.54E+18	.	Not_PD_known	.	FALSE	G	NA	NA	0	NA	NA	NA	0.050506132
chr12:40310515	rs281865050	LRRK2_K1468E	A	G	LRRK2	0.695	3.407231211	0.007425584	1563.409043	Parkinson_disea	PD_known	Uncertain_significance	FALSE	G	NA	NA	0	0	0	0.878975354	0.066865647
chr15:61922527	rs138540886	VPS13C_S2282F	G	A	VPS13C	0.1807	1.490929871	0.830718741	2.67584174	.	Not_PD_known	.	TRUE	I	0.633691132	NA	6.67E-06	NA	NA	NA	0.878925938
chr3:132499231	rs61748102	DNAJC13_A1421V	C	T	DNAJC13	0.1334	1.04738858	0.986030492	1.112564822	.	Not_PD_known	.	TRUE	I	0.999875128	0.003620027	0.00031	0.023473976	0.025690418	0.028113892	1
chr15:61966088	rs747812804	VPS13C_V1016I	C	T	VPS13C	0.8817	1.129189366	0.227946574	5.59371698	.	Not_PD_known	.	TRUE	I	0.592843115	NA	1.67E-06	NA	NA	NA	0.215815637
chr15:89323462	rs138457939	POLG_N736S	T	C	POLG	0.8892	1.041018957	0.59140158	1.832461234	Intellectual_disat	Not_PD_known	Conflicting_interpretations_of_pathogenicity	TRUE	I	0.597379088	6.41E-05	5.00E-06	0.008305422	0.023394188	0.065884721	0.713726688
chr12:40232383	rs144883021	LRRK2_Q116R	A	G	LRRK2	0.5884	4.014448588	0.026153507	616.200255	not_provided	Not_PD_known	Benign	FALSE	I	0.677628756	NA	0	0	0	1	0.087450493
chr1:155238144	rs121908300	GBA1_Y251H	A	G	GBA1	0.8647	2.941736343	1.20E-05	723231.0346	Gaucher_disease	Not_PD_known	Uncertain_significance	FALSE	G	NA	NA	0	0	0	1	0.050739575
chr1:20644627	rs7349186	PINK1_P305L	C	T	PINK1	0.8521	21.29346036	2.34E-13	1.94E+15	.	Not_PD_known	.	FALSE	G	NA	NA	0	NA	NA	NA	0.073051867
chr1:155238246	.	GBA1_P217S	G	A	GBA1	0.4931	4.352715921	0.064896084	291.9457508	.	Not_PD_known	.	FALSE	G	NA	0	0	0.02619157	0.541038598	1	0.051942956
chr15:89330133	rs61752784	POLG_G268A	C	G	POLG	0.4942	1.044355554	0.922138361	1.182771011	Seizures|Progres	Not_PD_known	Conflicting_interpretations_of_pathogenicity	TRUE	I	0.966621935	0.0007232	7.67E-05	0.026870801	0.031803112	0.037637769	1
chr1:16992115	rs143834546	ATP13A2_A669T	C	T	ATP13A2	0.9618	2.197015833	2.30E-14	2.10E+14	Kufor-Rakeb_syr	Not_PD_known	Uncertain_significance	FALSE	I	0.442866325	NA	0	NA	NA	NA	0.0510714
chr6:161785825	rs373750972	PRKN_N273S	T	C	PRKN	0.4906	1.979412335	0.284057101	13.79325908	.	Not_PD_known	.	FALSE	G	NA	NA	1.67E-06	NA	NA	NA	0.169344171
chr15:61940651	rs142341124	VPS13C_M1866T	A	G	VPS13C	0.6348	2.213776764	0.083360892	58.79024854	.	Not_PD_known	.	TRUE	I	0.576423228	NA	1.67E-06	NA	NA	NA	0.134561456
chr2:232794843	rs2289912	GIGYF2_P460T	C	A	GIGYF2	0.4126	1.027573297	0.962836176	1.096663074	not_provided	Not_PD_known	Benign	TRUE	I	0.998170555	0.004507498	0.000323333	0.01938454	0.021519699	0.023887762	1
chr1:16990191	rs137955309	ATP13A2_R778Q	C	T	ATP13A2	0.524	2.674600437	0.129737254	55.13826832	Kufor-Rakeb_syr	Not_PD_known	Benign/Likely_benign	FALSE	I	0.321387738	NA	0	NA	NA	NA	0.139353535
chr12:40340380	rs34015634	LRRK2_I2012T	T	C	LRRK2	0.9414	2.734914024	6.20E-12	1.21E+12	Parkinson_disea	PD_known	Uncertain_significance	FALSE	G	NA	NA	0	0	0	1	0.05055757
chr21:32631012	rs536572904	SYNJ1_D1608Y	C	A	SYNJ1	0.2714	3.246897743	0.398171684	26.47688266	.	Not_PD_known	.	TRUE	I	0.77662605	NA	0	0	NA	1	0.097007885
chr1:155237444	rs794727908	GBA1_I299T	A	G	GBA1	0.9608	1.654171038	3.13E-09	873148323.2	Gaucher_disease	Not_PD_known	Likely_pathogenic	FALSE	G	NA	NA	0	0	0	1	0.050292415
chr12:40309225	rs74163686	LRRK2_N1437H	A	C	LRRK2	0.9746	2.725903683	4.23E-27	1.76E+27	Parkinson_disea	PD_known	Pathogenic	FALSE	G	NA	NA	0	0	0	1	0.05016652
chr1:155239736	.	GBA1_Q112X	G	A	GBA1	0.6402	3.382787251	0.020392502	561.14986	Gaucher_disease	Not_PD_known	Pathogenic	FALSE	G	NA	NA	0	0	0	1	0.054555996
chr3:184323541	rs147678593	EIF4G1_T742M	C	T	EIF4G1	0.6996	1.478458513	0.202934502	10.77115793	.	Not_PD_known	.	TRUE	I	0.590655506	NA	0	1.70E-16	NA	1	0.162684208
chr1:7965425	rs74315353	PARK7_E64D	G	C	PARK7	0.9677	2.730814728	2.18E-21	3.43E+21	Parkinson_disea	PD_known	Pathogenic	FALSE	G	NA	NA	0	0	0	1	0.051245065
chr15:89328532	rs145289229	POLG_L392V	G	C	POLG	0.3459	1.101970565	0.900522616	1.348482654	Seizures|Progres	Not_PD_known	Conflicting_interpretations_of_pathogenicity	TRUE	I	0.787577033	0.000179539	2.33E-05	0.02879952	0.038988805	0.052778837	0.998775636
chr12:40302860	rs533532047	LRRK2_E1190K	G	A	LRRK2	0.8859	4.528541849	4.93E-09	4156037777	.	Not_PD_known	.	FALSE	I	0.616674602	NA	NA	NA	NA	NA	0.053759321
chr15:62023836	rs12595158	VPS13C_R153H	C	T	VPS13C	0.3549	1.029424594	0.9681696	1.094555123	not_provided	Not_PD_known	Benign	TRUE	I	0.999431491	NA	0.000368333	NA	NA	NA	1

chr12:40232385	rs141221000	NA	T	C	LRRK2	0.5883	4.016054689	0.02616397	616.4467844	not_provided	Not_PD_known	Conflicting_interpretations_of_pathogenicity	FALSE	I	0.677628756	NA	0	0	0	1	0.051219445
chr1:20649134	rs764328076	PINK1_R464H	G	A	PINK1	0.9629	1.035826853	0.235378599	4.558346736	.	Not_PD_known	.	TRUE	G	NA	NA	0	NA	NA	NA	0.156805811
chr1:7984981	rs28938172	PARK7_L166P	T	C	PARK7	0.967	2.729176731	5.81E-21	1.28E+21	Parkinson_disea	PD_known	Pathogenic	FALSE	G	NA	NA	0	0	0	1	0.050977817
chr2:74532831	rs550171858	HTRA2_R408Q	G	A	HTRA2	0.2759	59.82358612	0.038067286	94014.0953	.	Not_PD_known	.	FALSE	I	0.361924201	NA	0	0	NA	1	0.151152111
chr14:22877128	rs570350751	LRP10_Q581H	G	T	LRP10	0.8469	1.629706602	0.011458448	231.7891217	.	Not_PD_known	.	FALSE	I	0.357765853	NA	1.67E-06	0	NA	1	0.228892953
chr3:132503289	rs566485027	DNAJC13_A1598T	G	A	DNAJC13	0.8825	1.609140201	0.002927695	884.4270197	.	Not_PD_known	.	FALSE	I	0.304683477	NA	0	0	NA	1	0.069623377
chr15:61934224	rs112236709	VPS13C_N1955H	T	G	VPS13C	0.6173	1.026135701	0.927613611	1.135121848	not_provided	Not_PD_known	Benign	TRUE	I	0.995925605	NA	0.000135	NA	NA	NA	1
chr5:1403013	rs28364997	SLC6A3_A559V	G	A	SLC6A3	0.005296	1.48097403	1.123818206	1.951636009	Parkinsonism-dy	PD_known	Uncertain_significance	TRUE	I	0.624082804	0.000120785	2.50E-05	0.044893144	0.062093684	0.085875361	0.999463461
chr12:40323255	rs281865052	LRRK2_M1869V	A	G	LRRK2	0.9551	2.73026862	1.75E-15	4.27E+15	Parkinson_disea	PD_known	Pathogenic	FALSE	G	NA	NA	0	0	0	1	0.051942543
chr2:232794782	rs146944691	GIGYF2_Q439H	G	C	GIGYF2	0.2533	1.487802204	0.752622127	2.941124526	.	Not_PD_known	.	TRUE	I	0.582800508	5.58E-05	5.00E-06	0.009491852	0.026896994	0.076202915	0.387474237
chr6:161360129	rs778125254	PRKN_T415N	G	T	PRKN	0.7915	7.564755297	2.31E-06	24726349.14	not_provided	Not_PD_known	Likely_pathogenic	TRUE	G	NA	NA	0	NA	NA	NA	0.065973846
chr15:89318737	rs201732356	POLG_R1096C	G	A	POLG	0.3016	7.453621339	0.164850546	337.0111447	Abnormality_of_t	Not_PD_known	Likely_pathogenic	TRUE	G	NA	0	0	0.002707609	0.018380821	0.124769037	0.124834884
chr15:61854543	rs115819951	VPS13C_I3726V	T	C	VPS13C	0.2794	1.063855954	0.950841402	1.190303123	not_provided	Not_PD_known	Benign	TRUE	I	0.976831734	NA	9.17E-05	NA	NA	NA	1
chr15:89319073	rs150233690	POLG_V1044A	A	G	POLG	0.9712	1.005515153	0.746601279	1.354217775	Seizures|EEG_al	Not_PD_known	Uncertain_significance	TRUE	I	0.81855154	0.000280573	1.33E-05	0.006696906	0.014256536	0.030344797	0.991846973
chr12:40340400	rs34637584	LRRK2_G2019S	G	A	LRRK2	0	11.76112969	10.48087324	13.19777164	Parkinson_disea	PD_known	Pathogenic|_risk_factor	FALSE	G	0.913208783	1.39E-05	1.17E-05	0.222253987	0.251536879	0.284662145	0.974037252
chr15:89318962	rs140079523	POLG_R1081Q	C	T	POLG	0.7128	20.67034367	2.05E-06	208772562.6	Secondary_micr	Not_PD_known	Conflicting_interpretations_of_pathogenicity	FALSE	G	NA	NA	0	0	0	0.151404985	0.090122885
chr3:132454135	rs147315244	DNAJC13_R304W	C	T	DNAJC13	0.4046	1.945852141	0.406750217	9.308761003	.	Not_PD_known	.	FALSE	G	0.316726089	0	0	0.010959239	0.03760087	0.128997288	0.143927675
chr15:89325591	rs367610201	POLG_M603T	A	G	POLG	0.3286	2.187370199	0.455000846	10.51555932	Progressive_scle	Not_PD_known	Conflicting_interpretations_of_pathogenicity	TRUE	G	NA	4.42E-05	1.67E-06	0.001723468	0.011302137	0.074111111	0.161770972
chr15:62044256	rs757475739	NA	C	T	VPS13C	0.9049	1.096913142	0.240524818	5.002471048	.	Not_PD_known	.	FALSE	G	NA	NA	1.67E-06	NA	NA	NA	0.262098779
chr15:89321777	rs121918053	POLG_R853W	G	A	POLG	0.5528	57.75442674	8.81E-05	37877203.56	Progressive_scle	Not_PD_known	Uncertain_significance	TRUE	G	NA	NA	0	0	0	0.078740624	0.117432891
chr22:38132979	rs121908682	PLA2G6_V310E	A	T	PLA2G6	0.9565	2.734640547	5.46E-16	1.37E+16	Infantile_neuroax	Not_PD_known	Pathogenic	FALSE	G	NA	NA	0	NA	NA	NA	0.050462116
chr21:32657057	rs370346211	SYNJ1_T881I	G	A	SYNJ1	0.789	5.61E+14	5.28E-94	5.96E+122	Parkinson_disea	PD_known	Uncertain_significance	FALSE	I	0.390580863	NA	0	0	NA	1	0.065068053
chr1:175386035	rs140481433	TNR_T592A	T	C	TNR	0.2513	1.251571548	0.853016831	1.836342828	Parkinson_disea	PD_known	Uncertain_significance	TRUE	I	0.675572693	0.000267873	1.33E-05	0.00751407	0.014932461	0.029670029	0.96537483
chr1:155235829	rs398123528	GBA1_V414L	C	A	GBA1	0.8847	2.749997493	3.16E-06	2395886.82	Gaucher_disease	Not_PD_known	Pathogenic/Likely_pathogenic	FALSE	G	NA	NA	0	0	0	1	0.054683003
chr15:61967438	rs3784634	VPS13C_R974K	C	T	VPS13C	0.284	1.009848177	0.99180179	1.028222928	not_provided	Not_PD_known	Benign	TRUE	I	1.000000119	NA	0.007006667	NA	NA	NA	1
chr15:89323451	rs78347903	POLG_N740D	T	C	POLG	0.4296	1.397101873	0.609288132	3.203564192	Seizures|Progres	Not_PD_known	Uncertain_significance	TRUE	I	0.516980886	Inf	3.33E-06	0	0	0.366967116	0.530244933
chr15:89330258	rs147282197	POLG_Q226H	C	G	POLG	0.478	1.132468768	0.803013302	1.59709124	Autistic_disorder|	Not_PD_known	Conflicting_interpretations_of_pathogenicity	TRUE	I	0.873447895	0.000136783	1.00E-05	0.010064603	0.021932592	0.047787433	0.937434239
chr2:232832882	rs375089299	GIGYF2_R852Q	G	A	GIGYF2	0.2034	1.54759173	0.789648002	3.033047835	.	Not_PD_known	.	FALSE	I	0.46460104	6.15E-05	3.33E-06	0.004247401	0.016250267	0.062160256	0.440283665
chr17:46024061	rs63750424	MAPT_R406W	C	T	MAPT	0.05395	4.096774677	0.976352099	17.19007186	Frontotemporal_(	PD_known	Pathogenic	FALSE	G	NA	0	0	0.011642087	0.053129819	0.242448173	0.085068233
chr1:155236426	rs78396650	GBA1_A348V	G	A	GBA1	0.8756	7.653017389	6.55E-11	8.94E+11	Gaucher_disease	Not_PD_known	Pathogenic	FALSE	G	NA	NA	0	0	0	1	0.054826468
chr1:155236277	rs121908309	GBA1_R398X	G	A	GBA1	0.6685	3.18738235	0.015816272	642.3388704	Gaucher_disease	Not_PD_known	Pathogenic	FALSE	G	NA	NA	0	0	0	1	0.052011084
chr1:16986208	rs56004722	ATP13A2_G1085E	C	T	ATP13A2	0.7574	1.08E+133	0	Inf	.	Not_PD_known	.	FALSE	I	0.511400521	NA	0	NA	NA	NA	NA
chr12:40274666	rs145906734	LRRK2_L580F	A	T	LRRK2	0.7518	7.68215418	2.50E-05	2362001.711	not_provided	Not_PD_known	Benign	TRUE	I	0.950775683	NA	0	0	NA	1	0.055895976
chr15:61910215	rs148074630	VPS13C_R2936X	G	A	VPS13C	0.8548	7.539832749	3.00E-09	18927694425	Parkinson_disea	PD_known	Likely_pathogenic	FALSE	G	NA	NA	0	NA	NA	NA	0.066920749
chr3:132502298	rs79734612	DNAJC13_R1516C	C	T	DNAJC13	0.4803	2.03216149	0.283622259	14.56049443	not_provided	Not_PD_known	Benign	TRUE	I	0.796964586	NA	0	0	0	0.366957472	0.175427678
chr1:155238570	rs147138516	GBA1_D179H	C	G	GBA1	4.14E-08	3.274941479	2.143311365	5.004052079	.	Not_PD_known	.	TRUE	I	0.865636766	1.18E-05	1.67E-06	0.017201421	0.04232247	0.104124268	0.412319211
chr3:132528231	rs181058582	DNAJC13_E2142K	G	A	DNAJC13	0.3184	3.572980066	0.292777015	43.60378681	.	Not_PD_known	.	TRUE	G	0.448379397	Inf	1.67E-06	0	0	0.078775992	0.123545678
chr15:62010558	rs114954486	VPS13C_P309A	G	C	VPS13C	0.01607	4.049121727	1.296847086	12.64249805	not_provided	Not_PD_known	Likely_benign	TRUE	I	0.997521043	NA	0	NA	NA	NA	0.139821355
chr12:40263806	rs35328937	LRRK2_R521G	A	G	LRRK2	0.3936	1.277493557	0.72787933	2.242115859	Parkinson_disea	PD_known	Conflicting_interpretations_of_pathogenicity	TRUE	I	0.940006793	3.47E-05	3.33E-06	0.009995946	0.028796801	0.082950363	0.470552209
chr1:155236409	rs398123526	GBA1_D354H	C	G	GBA1	0.006812	100.8868912	3.567296407	2853.187302	Gaucher_disease	Not_PD_known	Conflicting_interpretations_of_pathogenicity	FALSE	G	NA	0	0	0.090164957	0.717383487	1	0.051985408
chr1:155235708	rs121908295	GBA1_P454R	G	C	GBA1	0.5134	1.378367737	0.52641995	3.609091216	Gaucher_disease	Not_PD_known	Pathogenic	FALSE	G	NA	0	0	0.091871349	0.277482936	0.837903095	0.168007247
chr3:132496652	rs200244570	DNAJC13_R1382H	G	A	DNAJC13	0.6039	21.11745606	0.000209214	2131533.006	.	Not_PD_known	.	TRUE	G	0.365253896	NA	0	0	0	0.105116266	0.10851476
chr1:155240048	rs760930573	GBA1_G49S	C	T	GBA1	0.8651	7.495478709	6.02E-10	93375982273	Gaucher_disease	Not_PD_known	Uncertain_significance	FALSE	G	NA	NA	0	0	0	0.766390731	0.080749304
chr12:40240594	rs56108242	LRRK2_C228S	G	C	LRRK2	0.8493	1.116724671	0.357592932	3.4874123	Parkinson_disea	PD_known	Uncertain_significance	TRUE	I	0.897544622	8.61E-06	1.67E-06	0.019173211	0.058101701	0.176050196	0.518964251
chr15:61890400	rs149077892	VPS13C_D3036N	C	T	VPS13C	0.07454	2.189120796	0.925401113	5.178565047	.	Not_PD_known	.	TRUE	I	0.800053895	NA	3.33E-06	NA	NA	NA	0.592982573
chr15:89319234	rs551708243	POLG_A1033V	G	A	POLG	0.1773	1.845591828	0.757578588	4.496179337	Progressive_scle	Not_PD_known	Conflicting_interpretations_of_pathogenicity	TRUE	I	0.645188749	9.12E-05	3.33E-06	0.000980997	0.010966296	0.122569525	0.486821683
chr15:89319053	rs121918049	POLG_G1051R	C	G	POLG	0.2499	1.955214684	0.624008056	6.126306256	Intellectual_disab	Not_PD_known	Conflicting_interpretations_of_pathogenicity	TRUE	G	NA	2.07E-05	1.67E-06	0.007293515	0.024176739	0.080134881	0.209043823
chr3:184325887	rs199973292	EIF4G1_N1054S	A	G	EIF4G1	0.8812	2.758535713	4.56E-06	1668460.662	.	Not_PD_known	.	TRUE	G	NA	NA	0	0	0	1	0.051143261
chr5:176621218	rs104893937	SNCB_P123H	G	T	SNCB	0.4753	1.249945562	0.677463231	2.306197352	Lewy_body_dem	Not_PD_known	Likely_pathogenic	TRUE	I	0.620998323	9.13E-05	8.33E-06	0.01198617	0.027369231	0.062488272	0.934171925
chr12:40293624	rs58559150	LRRK2_Q923H	G	C	LRRK2	0.5608	1.268328637	0.56940917	2.825134573	Parkinson_disea	PD_known	Uncertain_significance	TRUE	I	0.865468502	1.72E-05	1.67E-06	0.00962596	0.029135234	0.088178676	0.388183688
chr2:74530208	rs146380692	HTRA2_P68S	C	T	HTRA2	0.8772	1.508627052	0.008182709	278.1420627	not_provided	Not_PD_known	Likely_benign	FALSE	I	0.464771777	NA	0	0	NA	1	0.081259286
chr15:61916007	rs139139141	VPS13C_H2691D	G	C	VPS13C	0.6989	1.162531552	0.541926985	2.49384077	.	Not_PD_known	.	TRUE	I	0.641811252	NA	3.33E-06	NA	NA	NA	0.449466607
chr22:32479135	rs143041875	FBXO7_S93A	T	G	FBXO7	0.8061	1.094830985	0.530875366	2.257883793	Parkinson_disea	PD_known	Uncertain_significance	TRUE	I	0.643031061	NA	3.33E-06	NA	NA	NA	0.549387052
chr15:89325562	rs147407423	POLG_H613Y	G	A	POLG	0.7601	1.247947248	0.300931685	5.175169023	Global_developm	Not_PD_known	Conflicting_interpretations_of_pathogenicity	TRUE	I	0.885116398	NA	0	0	NA	1	0.172029722
chr6:161785820	rs34424986	PRKN_R275W	G	A	PRKN	4.20E-05	1.299136743	1.146204444	1.47247403	See_cases|Neop	PD_known	Pathogenic	TRUE	I	1.000000119	NA	4.50E-05	NA	NA	NA	0.999999997
chr22:38113561	rs587784347	PLA2G6_R710C	G	A	PLA2G6	0.1459	3.862829708	0.625107277	23.87022819	Iron_accumulatio	Not_PD_known	Likely_pathogenic	TRUE	G	NA	NA	0	NA	NA	NA	0.062783122
chr12:40363526	rs34778348	LRRK2_G2385R	G	A	LRRK2	0.3694	1.839143548	0.486193785	6.956997586	Parkinson_disea	PD_known	Conflicting_interpretations_of_pathogenicity|_risk_factor	TRUE	I	0.611731946	0	0	0.083245011	1	1	0.120125035
chr5:122450987	rs143468222	SNCAIP_A714T	G	A	SNCAIP	0.7481	1.124569167	0.549479397	2.301552741	Parkinson_Disea	PD_known	Uncertain_significance	TRUE	I	0.777315617	2.85E-05	3.33E-06	0.004111006	0.035093011	0.299518439	0.424382063
chr20:5100832	rs143571424	TMEM230_R108C	G	A	TMEM230	0.2358	1.159628853	0.907824682	1.48127618	.	Not_PD_known	.	FALSE	I	0.443526745	0.00052717	3.50E-05	0.01240144	0.019917657	0.031984146	0.999999845
chr22:38128349	rs199636953	PLA2G6_A423V	G	A	PLA2G6	0.2275	1.566274704	0.75576258	3.246014707	Infantile_neuroax	Not_PD_known	Uncertain_significance	FALSE	I	0.486872762	NA	5.00E-06	NA	NA	NA	0.335299539
chr22:38135029	rs201190836	PLA2G6_R285C	G	A	PLA2G6	0.8976	1.099988802	0.257618364	4.696774516	.	Not_PD_known	.	TRUE	G	NA	NA	1.67E-06	NA	NA	NA	0.155880999
chr15:61962754	rs776169337	VPS13C_K1144X	T	A	VPS13C	0.934	2.736008209	1.23E-10	60694808247	.	Not_PD_known	.	FALSE	G	NA	NA	0	NA	NA	NA	0.062447184
chr15:89323423	rs202037973	POLG_F749S	A	G	POLG	0.7472	8.018889933	2.55E-05	2520752.434	Intellectual_disab	Not_PD_known	Conflicting_interpretations_of_pathogenicity	TRUE	G	0.364175946	NA	0	0	0	1	0.053694583
chr1:7961737	rs140230911	NA	T	A	PARK7	0.2845	2.816232503	0.422864281	18.75581804	Parkinson_disea	PD_known	Uncertain_significance	TRUE	I	0.676396728	NA	NA	NA	NA	NA	0.252465593
chr6:162443429	rs146288080	PRKN_D18N	C	T	PRKN	0.4798	263050.1559	2.43E-10	2.84E+20	not_provided	Not_PD_known	Likely_benign	FALSE	I	0.508517146	NA	0	NA	NA	NA	0.056522955
chr1:20644662	rs200949139	PINK1_V317I	G	A	PINK1	0.4201	2.068037314	0.353611651	12.09456285	not_provided	Not_PD_known	Uncertain_significance	TRUE	I	0.549178064	NA	1.67E-06	NA	NA	NA	0.318882245
chr3:184327401	rs112176450	EIF4G1_R1206H	G	A	EIF4G1	0.3394	1.332024546	0.739710785	2.398625824	Parkinson_disea	PD_known	risk_factor	TRUE	I	0.544064879	2.46E-05	5.00E-06	0.031939566	0.060986673	0.116441071	0.791689304
chr1:7985019	rs71653622	PARK7_A179T	G	A	PARK7	0.05466	1.43777963	0.992686872	2.08243941	Parkinson_disea	PD_known	Uncertain_significance	FALSE	I	0.644672453	8.83E-05	1.00E-05	0.013688095	0.03395596	0.084220822	0.976730487
chr3:132484650	rs202127368	DNAJC13_T1082I	C	T	DNAJC13	0.7221	1.066625578	0.747336296	1.52232687	.	Not_PD_known	.	TRUE	I	0.789919794	0.000180313	1.00E-05	0.007189045	0.016637698	0.038498669	0.9602397
chr12:40305946	rs183902574	LRRK2_C1313X	T	A	LRRK2	0.966	1.036448536	0.200355899	5.361586928	not_specified	Not_PD_known	Uncertain_significance	TRUE	G	NA	0	0	0.006399565	0.020984055	0.068801399	0.211075556
chr6:161350125	rs182893847	PRKN_M458L	T	G	PRKN	0.6505	1.356625003	0.362735993	5.07374904	Neoplasm_of_ov	Not_PD_known	Conflicting_interpretations_of_pathogenicity	TRUE	G	NA	NA	1.67E-06	NA	NA	NA	0.252329649
chr1:155237438	rs140955685	GBA1_R301H	C	T	GBA1	0.0001534	2.474900022	1.548018924	3.956754031	not_provided	Not_PD_known	Uncertain_significance	TRUE	I	0.638045669	9.67E-06	3.33E-06	0.046890562	0.103416785	0.228053413	0.489801286
chr12:40302866	rs281865047	LRRK2_I1192V	A	G	LRRK2	0.9972	1.004108416	0.101516696	9.931703359	Parkinson_disea	PD_known	Uncertain_significance	TRUE	I	0.794680834	NA	0	0	0	1	0.090197428
chr1:65385756	rs368061386	DNAJC6_R225H	G	A	DNAJC6	0.5525	2.826106587	0.09176246	87.03862625	.	Not_PD_known	.	FALSE	I	0.386579186	NA	0	NA	NA	NA	0.248437618
chr15:61936649	rs538854121	VPS13C_E1901D	C	G	VPS13C	0.3213	2.399115193	0.425536735	13.525868	.	Not_PD_known	.	TRUE	I	0.50409317	NA	0	NA	NA	NA	0.186371259
chr1:20649217	rs34208370	PINK1_R492X	C	T	PINK1	0.9922	1.005615709	0.325567977	3.106149947	Parkinson_disea	PD_known	Pathogenic	TRUE	I	0.528490782	NA	1.67E-06	NA	NA	NA	0.262726241

chr15:89320857	rs201477273	POLG_R964C	G	A	POLG	0.7894	7.503728272	2.83E-06	19898362.5	Seizures|Spinoce	Not_PD_known	Conflicting_interpretations_of_pathogenicity	TRUE	G	NA	NA	0	0	0	0.328494183	0.07130755
chr22:38120867	rs121908681	PLA2G6_K545T	T	G	PLA2G6	0.02313	5.033421643	1.248022127	20.30038802	Microcephaly|Glc	PD_known	Pathogenic	TRUE	G	NA	NA	0	NA	NA	NA	0.065749324
chr3:184327230	rs375604272	EIF4G1_R1149Q	G	A	EIF4G1	0.4197	3.775753631	0.149757793	95.19581683	.	Not_PD_known	.	FALSE	I	NA	Inf	1.67E-06	0	0	0.61434238	0.241433122
chr1:20648601	rs556540177	PINK1_R407Q	G	A	PINK1	0.8178	20.36260234	1.50E-10	2.77E+12	Parkinson_disea	PD_known	Uncertain_significance	FALSE	G	NA	NA	0	NA	NA	NA	0.114718678
chr6:162443383	rs147757966	PRKN_R33Q	C	T	PRKN	0.07604	3.352478758	0.881061366	12.75633486	Parkinson_disea	PD_known	Pathogenic/Likely_pathogenic	TRUE	G	NA	NA	3.33E-06	NA	NA	NA	0.103558124
chr15:61984004	rs200452364	VPS13C_A577V	G	A	VPS13C	0.7524	3.003264909	0.003239073	2784.623626	.	Not_PD_known	.	FALSE	I	NA	NA	0	NA	NA	NA	NA
chr6:161973335	rs144032774	PRKN_R234Q	C	T	PRKN	0.8176	1.173745597	0.300769226	4.580517578	not_provided	Not_PD_known	Uncertain_significance	TRUE	I	0.550825596	NA	1.67E-06	NA	NA	NA	0.432490732
chr1:20639911	rs144071530	PINK1_A232V	C	T	PINK1	0.9872	2.720185292	1.76E-53	4.20E+53	Parkinson_disea	PD_known	Uncertain_significance	FALSE	G	0.489761978	NA	0	NA	NA	NA	0.050123156
chr12:40235634	rs33995463	LRRK2_L119P	T	C	LRRK2	0.8474	1.016636877	0.859441946	1.202583308	Parkinson_disea	PD_known	Conflicting_interpretations_of_pathogenicity	TRUE	I	0.999605775	0.000467634	4.00E-05	0.020627264	0.025661088	0.031921461	0.999999997
chr6:161360169	rs55830907	PRKN_R402C	G	A	PRKN	0.2428	1.141564854	0.914055488	1.425701538	Parkinson_disea	PD_known	Conflicting_interpretations_of_pathogenicity	TRUE	I	0.768148124	NA	2.67E-05	NA	NA	NA	0.999988989
chr1:20645675	rs76753586	PINK1_A359T	G	A	PINK1	0.6444	20.76772295	5.28E-05	8164483.299	Parkinson_disea	PD_known	Uncertain_significance	TRUE	G	NA	NA	0	NA	NA	NA	0.10296342
chr14:22873373	rs2273837	LRP10_R48W	C	T	LRP10	0.04895	8.699799679	1.01009461	74.93012406	.	Not_PD_known	.	FALSE	G	0.405358285	1.07E-05	1.67E-06	0.015459084	0.046758656	0.141411473	0.558278721
chr15:61880871	rs140034886	VPS13C_T3287I	G	A	VPS13C	0.8509	1.070258277	0.526955065	2.173720031	.	Not_PD_known	.	TRUE	I	0.811202109	NA	3.33E-06	NA	NA	NA	0.493989156
chr15:50586433	rs8042919	TRPM7_T1482I	G	A	TRPM7	0.3039	1.01389566	0.987613354	1.040877388	Amyotrophic_late	PD_known	Conflicting_interpretations_of_pathogenicity	TRUE	I	0.986967027	0.018597568	0.001756667	0.027207764	0.028337039	0.029510389	1
chr22:32493191	rs762037477	FBXO7_V352I	G	A	FBXO7	0.5348	1.328565781	0.541727593	3.25825573	Parkinson_disea	PD_known	Uncertain_significance	TRUE	I	0.555329204	NA	1.67E-06	NA	NA	NA	0.345619299
chr3:132494190	rs61748101	DNAJC13_E1291G	A	G	DNAJC13	0.2976	1.072400936	0.940243871	1.22313349	Parkinson_disea	PD_known	Conflicting_interpretations_of_pathogenicity	TRUE	G	0.9742378	0.001617734	0.000106667	0.017382487	0.019780759	0.022508119	1
chr15:61911844	rs115869241	VPS13C_S2904L	G	A	VPS13C	0.4294	1.060244986	0.917098864	1.225734186	not_provided	Not_PD_known	Likely_benign	TRUE	I	0.884274602	NA	5.50E-05	NA	NA	NA	1
chr12:40304004	rs72546324	LRRK2_H1216R	A	G	LRRK2	0.9314	2.749997493	2.71E-10	27908133316	Parkinson_disea	PD_known	Uncertain_significance	FALSE	G	NA	NA	0	0	0	1	0.053702603
chr3:184321516	rs16858632	EIF4G1_Y311C	A	G	EIF4G1	0.4337	1.216162002	0.745052942	1.985160962	not_provided	Not_PD_known	Benign	TRUE	I	0.95537883	0.000112606	5.00E-06	0.001744473	0.013320739	0.101683513	0.721207427
chr15:62008659	rs767007361	VPS13C_R372X	G	A	VPS13C	0.8618	7.685227656	8.23E-10	71731226085	.	Not_PD_known	.	FALSE	G	NA	NA	0	NA	NA	NA	0.070284289
chr3:132478139	rs141952333	DNAJC13_R903K	G	A	DNAJC13	0.6701	1.038211997	0.873733386	1.233653387	.	Not_PD_known	.	TRUE	I	0.815282881	0.000593248	4.67E-05	0.016595548	0.023598894	0.033552287	0.999999999
chr3:132502295	rs55825559	DNAJC13_P1515S	C	T	DNAJC13	0.1406	1.038004375	0.987787189	1.090774506	not_specified|not	Not_PD_known	Benign/Likely_benign	TRUE	I	0.980295181	0.005419375	0.000481667	0.024819928	0.026663593	0.028641918	1
chr6:162443314	rs137853059	PRKN_V56E	A	T	PRKN	0.4294	1.577592578	0.50924604	4.887221785	Parkinson_disea	PD_known	Conflicting_interpretations_of_pathogenicity	TRUE	G	NA	NA	0	NA	NA	NA	0.166805475
chr15:61917535	rs114277501	VPS13C_V2621I	C	T	VPS13C	0.7661	1356475.026	5.29E-35	3.48E+46	.	Not_PD_known	.	FALSE	I	0.353398651	NA	0	NA	NA	NA	0.066333825
chr15:61854888	rs62007358	VPS13C_D3715Y	C	A	VPS13C	0.993	1.001100605	0.782798468	1.280281531	.	Not_PD_known	.	TRUE	I	0.788080812	NA	2.33E-05	NA	NA	NA	0.999819345
chr15:61915655	rs34060567	VPS13C_K2808R	T	C	VPS13C	0.7783	1.056751944	0.719530761	1.55201797	not_provided	Not_PD_known	Benign	TRUE	I	0.90321815	NA	1.17E-05	NA	NA	NA	0.981560189
chr12:40240543	rs112794616	LRRK2_A211V	C	T	LRRK2	0.7371	1.117394907	0.584284066	2.136925257	Parkinson_disea	PD_known	Conflicting_interpretations_of_pathogenicity	TRUE	I	0.521142423	0.000147012	3.33E-06	0.000513003	0.006802152	0.090180509	0.495221648
chr15:89319031	rs796052911	POLG_F1058S	A	G	POLG	0.8786	2.746424819	6.46E-06	1166851.904	Progressive_scle	Not_PD_known	Uncertain_significance	TRUE	G	NA	NA	0	0	0	1	0.0560814
chr1:155238629	rs79653797	GBA1_R159Q	C	T	GBA1	0.3722	4.054794467	0.187391363	87.73807889	Gaucher_disease	Not_PD_known	Pathogenic/Likely_pathogenic	FALSE	G	NA	NA	0	0	0	0.289760269	0.071903577
chr1:16988161	rs55708915	ATP13A2_I902F	T	A	ATP13A2	0.9476	1.008233712	0.788994444	1.28839338	Kufor-Rakeb_syr	Not_PD_known	Conflicting_interpretations_of_pathogenicity	FALSE	I	0.596549869	NA	3.00E-05	NA	NA	NA	0.999998647
chr12:40367050	rs55633591	LRRK2_N2479D	A	G	LRRK2	0.9678	2.730541661	1.73E-21	4.30E+21	Parkinson_disea	PD_known	Uncertain_significance	FALSE	G	NA	NA	0	0	0	1	0.053707352
chr15:61984885	rs141515062	VPS13C_S565T	A	T	VPS13C	0.9073	1.038939	0.546035111	1.97678542	.	Not_PD_known	.	TRUE	I	0.678934157	NA	5.00E-06	NA	NA	NA	0.598343837
chr1:16992042	rs61734958	ATP13A2_V693A	A	G	ATP13A2	0.7916	1.244955766	0.245041425	6.325113632	Kufor-Rakeb_syr	Not_PD_known	Benign/Likely_benign	TRUE	I	0.620706797	NA	0	NA	NA	NA	0.191576355
chr16:46671766	rs797044948	VPS35_Q488R	T	C	VPS35	0.9667	2.731360946	9.46E-21	7.89E+20	Inborn_genetic_c	Not_PD_known	Likely_pathogenic	FALSE	G	NA	NA	0	0	0	1	0.050172391
chr3:184327622	rs2230570	EIF4G1_L1234P	T	C	EIF4G1	0.3839	1.024187894	0.970445534	1.080906456	not_specified|not	Not_PD_known	Benign	TRUE	I	0.997038424	0.004220475	0.000398333	0.026242933	0.028314346	0.030546819	1
chr2:74531688	rs201615648	HTRA2_R344H	G	A	HTRA2	0.7528	7.827944816	2.15E-05	2854867.528	.	Not_PD_known	.	TRUE	G	NA	NA	0	1.89E-17	0	0.539375422	0.06452478
chr2:232844373	rs144086186	GIGYF2_S1035C	C	G	GIGYF2	0.2909	1.129528174	0.901055883	1.41593204	.	Not_PD_known	.	TRUE	I	0.554613769	0.000821667	3.33E-05	0.007050016	0.012170387	0.021006277	0.999999569
chr12:40298346	rs111341148	LRRK2_R1067Q	G	A	LRRK2	0.839	2.753024154	0.000157307	48180.61304	Parkinson_disea	PD_known	Uncertain_significance	TRUE	G	NA	NA	0	0	0	0.792100899	0.056598054
chr15:89320917	rs768653086	POLG_E944K	C	T	POLG	0.893	2.746974159	1.11E-06	6788143.192	Progressive_scle	Not_PD_known	Uncertain_significance	TRUE	G	NA	NA	0	3.53E-17	0	1	0.053522676
chr15:89321842	rs41549716	POLG_Y831C	T	C	POLG	0.5106	1.029012907	0.945100508	1.120375615	Seizures|Progres	Not_PD_known	Conflicting_interpretations_of_pathogenicity	TRUE	I	0.967782915	0.001748109	0.000163333	0.024385003	0.028030292	0.032217467	1
chr20:5069330	rs141618836	TMEM230_R81Q	C	T	TMEM230	0.3688	74.18040265	0.006182602	890034.9406	.	Not_PD_known	.	FALSE	I	0.353610843	NA	1.67E-06	0	NA	1	0.361182939
chr22:38169255	rs11570605	PLA2G6_V58I	C	T	PLA2G6	0.4591	1.379884776	0.588367221	3.236213586	Infantile_neuroax	Not_PD_known	Benign	TRUE	I	0.99817574	NA	3.33E-06	NA	NA	NA	0.496264801
chr1:155238251	.	GBA1_A215D	G	T	GBA1	0.6902	3.186744937	0.010672425	951.5497774	not_provided	Not_PD_known	Uncertain_significance	FALSE	G	NA	NA	0	0	0	1	0.051323332
chr15:89333427	rs139599587	POLG_H110Y	G	A	POLG	0.2118	2.572298867	0.583835508	11.33319466	Seizures|Progres	Not_PD_known	Conflicting_interpretations_of_pathogenicity	FALSE	I	0.410079569	NA	1.67E-06	0	NA	1	0.206467695
chr1:155239716	rs121908312	GBA1_K118N	C	G	GBA1	0.02636	10.2348647	1.314021129	79.71900388	Gaucher_disease	Not_PD_known	Pathogenic	FALSE	G	NA	0	0	0.012352452	0.098781589	0.78986882	0.064335533
chr14:22877108	rs146141715	LRP10_A575T	G	A	LRP10	0.007091	2.435373176	1.273952153	4.655624227	.	Not_PD_known	.	TRUE	I	0.760359526	8.14E-06	1.67E-06	0.016160483	0.061408232	0.233307821	0.420514303
chr12:40320103	rs200143418	LRRK2_Q1648R	A	G	LRRK2	0.7048	1.153614434	0.550892273	2.415764983	not_provided	Not_PD_known	Uncertain_significance	TRUE	I	0.534813344	0.000119687	3.33E-06	0.001498863	0.008355147	0.046566834	0.319778854
chr15:62023837	rs180757384	VPS13C_R153C	G	A	VPS13C	0.802	1.176330679	0.330453938	4.187433431	.	Not_PD_known	.	FALSE	I	0.329761326	NA	1.67E-06	NA	NA	NA	0.258863134
chr1:155235777	rs77738682	GBA1_N431I	T	A	GBA1	0.04955	7.849893776	1.004076286	61.37066793	.	Not_PD_known	.	FALSE	G	NA	0	0	0.015330931	0.132774214	1	0.105878949
chr22:32475378	rs9621461	FBXO7_G6E	G	A	FBXO7	0.3252	1.013794275	0.986547307	1.041793763	not_provided	Not_PD_known	Benign	TRUE	I	0.99396044	NA	0.001661667	NA	NA	NA	1
chr22:38126417	rs76718524	PLA2G6_R461W	G	A	PLA2G6	0.2755	1.542647352	0.707656834	3.362874116	Infantile_neuroax	Not_PD_known	Uncertain_significance	TRUE	G	0.495183051	NA	3.33E-06	NA	NA	NA	0.514329278
chr15:61927138	rs75341202	VPS13C_L2157F	G	A	VPS13C	0.2103	1.048122009	0.973848044	1.128060741	not_provided	Not_PD_known	Benign	TRUE	I	0.984036624	NA	0.00022	NA	NA	NA	1
chr12:40310561	rs113431708	LRRK2_R1483Q	G	A	LRRK2	0.807	20.65381401	5.79E-10	7.37E+11	Parkinson_disea	PD_known	Uncertain_significance	FALSE	G	NA	NA	0	0	0	0.286454365	0.093475667
chr15:89325639	rs113994096	POLG_P587L	G	A	POLG	0.7176	1.031176105	0.873101032	1.217870693	Abnormality_of_t	Not_PD_known	Conflicting_interpretations_of_pathogenicity	TRUE	I	0.999281406	0.000477709	4.17E-05	0.021166018	0.026166581	0.032346633	0.999999997
chr1:16986091	rs189334432	ATP13A2_P1124L	G	A	ATP13A2	0.09528	1.117394907	0.980653591	1.273203289	Kufor-Rakeb_syr	Not_PD_known	Conflicting_interpretations_of_pathogenicity	TRUE	I	0.692980587	NA	9.00E-05	NA	NA	NA	1
chr1:16986246	rs372995036	ATP13A2_P1173L	G	A	ATP13A2	0.4081	2.390732968	0.30317169	18.85269737	Kufor-Rakeb_syr	Not_PD_known	Uncertain_significance	FALSE	I	0.376375973	NA	1.67E-06	NA	NA	NA	0.583692879
chr6:162262627	rs769099303	PRKN_R104W	G	A	PRKN	0.7974	21.37880477	1.50E-09	3.05E+11	not_provided	Not_PD_known	Uncertain_significance	FALSE	G	NA	NA	0	NA	NA	NA	0.093717391
chr1:155237370	rs765633380	GBA1_R324C	G	A	GBA1	0.5001	3.195680327	0.10916574	93.54924667	Gaucher_disease	Not_PD_known	Pathogenic	FALSE	G	NA	NA	0	0	0	1	0.064713269
chr1:155235780	rs76910485	GBA1_P430L	G	A	GBA1	0.09752	193.9305296	0.381690961	98532.72446	Gaucher_disease	Not_PD_known	Pathogenic	TRUE	G	NA	0	0	0.051825686	1	1	0.052635372
chr2:232790836	rs371141455	GIGYF2_S284T	G	C	GIGYF2	0.7485	1.117283173	0.566965967	2.201757709	.	Not_PD_known	.	TRUE	I	0.671529591	4.18E-05	3.33E-06	0.004544251	0.023926464	0.125957816	0.575764528
chr15:89321792	rs113994098	POLG_G848S	C	T	POLG	0.7446	1.112044263	0.586867622	2.107191462	POLG-_Related_	Not_PD_known	Pathogenic	TRUE	I	0.545777917	4.25E-05	6.67E-06	0.027261889	0.047043723	0.081174884	0.823341966
chr1:155236246	rs75548401	GBA1_T408M	G	A	GBA1	4.87E-20	1.475061966	1.357433793	1.602883188	Parkinson_disea	PD_known	Conflicting_interpretations_of_pathogenicity	TRUE	I	0.850529432	0.000995218	0.000116667	0.028865143	0.035168176	0.042842054	1
chr1:20637888	rs45604240	PINK1_T145M	C	T	PINK1	0.4504	3.855882869	0.115871669	128.3129241	Parkinson_disea	PD_known	Conflicting_interpretations_of_pathogenicity	FALSE	G	NA	NA	0	NA	NA	NA	0.130656463
chr12:40364843	rs78964014	LRRK2_E2395K	G	A	LRRK2	0.8713	7.62932977	1.62E-10	3.60E+11	Parkinson_disea	PD_known	Uncertain_significance	FALSE	G	NA	NA	0	0	0	1	0.066531555
chr3:132507256	rs79953286	DNAJC13_Y1673C	A	G	DNAJC13	0.625	1.008536228	0.974912067	1.043320067	not_specified|not	Not_PD_known	Benign	TRUE	I	0.971063852	0.014280782	0.001018333	0.019616295	0.021392386	0.023325545	1
chr15:61945803	rs114089496	VPS13C_A1687V	G	A	VPS13C	0.9108	1.009040622	0.861420938	1.181957545	not_provided	Not_PD_known	Benign	TRUE	I	0.866328061	NA	4.83E-05	NA	NA	NA	1
chr22:38115667	rs121908683	PLA2G6_R632W	G	A	PLA2G6	0.6766	3.235553465	0.012999973	805.2944605	Neurodegenerati	Not_PD_known	Pathogenic	FALSE	G	NA	NA	0	NA	NA	NA	0.065080589
chr12:40251273	rs78501232	LRRK2_E334K	G	A	LRRK2	0.01492	1.374788636	1.064098541	1.77619245	Parkinson_disea	PD_known	Conflicting_interpretations_of_pathogenicity	TRUE	I	0.776489913	0.000229101	1.67E-05	0.012067697	0.021824475	0.039465441	0.999214085
chr15:61954471	rs140338178	VPS13C_I1417L	T	G	VPS13C	0.6016	1.079934078	0.809278637	1.441107622	not_provided	Not_PD_known	Likely_benign	TRUE	I	0.78455317	NA	1.67E-05	NA	NA	NA	0.998319461
chr15:61880845	rs377118536	VPS13C_R3296W	G	A	VPS13C	0.6241	1.813937687	0.167613256	19.63072616	.	Not_PD_known	.	FALSE	I	0.318716466	NA	0	NA	NA	NA	0.119600298
chrX:155260942	rs587777874	RAB39B_T168K	G	T	RAB39B	0.8993	2.753850186	4.21E-07	18008024.1	Early-onset_park	PD_known	Pathogenic	TRUE	G	NA	NA	0	NA	NA	NA	0.050374261
chr1:17005754	rs151117874	ATP13A2_T12M	G	A	ATP13A2	0.4933	1.573810895	0.430062815	5.759346423	Kufor-Rakeb_syr	Not_PD_known	Uncertain_significance	FALSE	I	NA	NA	3.33E-06	NA	NA	NA	0.241613054
chr1:20649109	rs45539432	PINK1_Q456X	C	T	PINK1	0.3552	3.592685597	0.23869612	54.07456892	Parkinson_disea	PD_known	Pathogenic	TRUE	G	NA	NA	1.67E-06	NA	NA	NA	0.261995309
chr3:184321987	rs200221361	EIF4G1_A468E	C	A	EIF4G1	0.1275	1.395007791	0.90922381	2.140338513	.	Not_PD_known	.	FALSE	I	0.414601833	NA	NA	NA	NA	NA	0.999936209
chr16:46662405	rs569735490	VPS35_F635L	A	C	VPS35	0.7745	3.087927025	0.001378086	6919.231586	.	Not_PD_known	.	FALSE	I	0.364269316	NA	0	0	0	1	0.053859665

chr15:61963904	rs200815172	VPS13C_A1088T	C	T	VPS13C	0.6654	1.095159484	0.725498699	1.65317222	.	Not_PD_known	.	TRUE	I	0.819589555	NA	6.67E-06	NA	NA	NA	0.872200377
chr15:61920268	rs143926369	VPS13C_V2426I	C	T	VPS13C	0.1908	1.138942272	0.93732613	1.383925464	not_provided	Not_PD_known	Likely_benign	TRUE	I	0.896753132	NA	3.50E-05	NA	NA	NA	0.999999802
chr15:62023783	rs150832196	VPS13C_K171E	T	C	VPS13C	0.505	1.123332821	0.797941494	1.581414975	not_provided	Not_PD_known	Likely_benign	TRUE	I	0.798568368	NA	1.17E-05	NA	NA	NA	0.977671252
chr15:89333267	rs752892262	POLG_P163L	G	A	POLG	0.4315	1.273921579	0.696984574	2.328424829	Progressive_scle	Not_PD_known	Uncertain_significance	FALSE	G	NA	0.000179765	8.33E-06	0.007457299	0.013907007	0.025931787	0.947927292
chr3:132511169	rs142160751	DNAJC13_E1740Q	G	C	DNAJC13	0.1591	1.161834243	0.942951905	1.431524557	.	Not_PD_known	.	TRUE	I	0.897105753	0.000323418	2.67E-05	0.015595653	0.02473579	0.039226398	0.999975765
chr6:161350208	rs191486604	PRKN_G430D	C	T	PRKN	0.001228	1.849471643	1.27393177	2.685030266	Parkinson_disea	PD_known	Pathogenic/Likely_pathogenic	TRUE	I	0.621124446	NA	3.33E-06	NA	NA	NA	0.632696691
chr15:89318587	rs2307440	POLG_R1146C	G	A	POLG	0.9809	1.007830499	0.534482655	1.900384055	Progressive_scle	Not_PD_known	Conflicting_interpretations_of_pathogenicity	TRUE	I	0.769532561	5.58E-05	3.33E-06	0.006715896	0.017914608	0.047784269	0.562158132
chr12:40274671	rs79299560	LRRK2_L582P	T	C	LRRK2	0.9334	2.7687612	1.18E-10	65039199871	.	Not_PD_known	.	FALSE	G	NA	NA	0	0	0	1	0.051012714
chr15:61984953	rs35942317	VPS13C_T542M	G	A	VPS13C	0.3094	2.099711596	0.502274749	8.777643697	not_provided	Not_PD_known	Benign	TRUE	I	0.804283381	NA	1.67E-06	NA	NA	NA	0.286055846
chr1:155239948	rs1141811	GBA1_T82I	G	A	GBA1	0.8341	7.551906138	4.61E-08	1238263708	.	Not_PD_known	.	FALSE	G	NA	NA	0	0	0	1	0.068571458
chr1:65401812	rs143504255	DNAJC6_S663L	C	T	DNAJC6	0.004727	12928.63632	18.15322081	9207712.444	not_provided	Not_PD_known	Likely_benign	FALSE	I	0.374326289	NA	0	NA	NA	NA	0.076160751
chr15:89324193	rs2307450	POLG_E662K	C	T	POLG	0.8258	1.084913218	0.525139152	2.241380566	Seizures|Progres	Not_PD_known	Benign	TRUE	I	0.516129494	6.41E-05	5.00E-06	0.005514356	0.02339534	0.099241734	0.852306394
chr15:61969413	rs146460562	VPS13C_T933A	T	C	VPS13C	0.1844	1.088825944	0.960274594	1.234586381	not_provided	Not_PD_known	Benign/Likely_benign	TRUE	I	0.877734661	NA	8.17E-05	NA	NA	NA	1
chr1:155235814	.	GBA1_D419N	C	T	GBA1	0.6785	3.228443072	0.012664911	822.9702074	Gaucher_disease	Not_PD_known	Likely_pathogenic	FALSE	G	NA	NA	0	0	0	1	0.052554233
chr15:89327004	rs769637557	POLG_K498T	T	G	POLG	0.2002	1.546354152	0.79366962	3.012854596	Progressive_scle	Not_PD_known	Uncertain_significance	TRUE	G	NA	2.24E-05	3.33E-06	0.023468382	0.044617373	0.08481841	0.375432474
chr12:40304000	rs143710836	LRRK2_A1215T	G	A	LRRK2	0.1093	1.988538606	0.857416055	4.611863476	Parkinson_disea	PD_known	Uncertain_significance	FALSE	I	0.4802531	1.14E-05	1.67E-06	0.008644954	0.043866263	0.22255073	0.424668453
chr22:38126371	rs146684391	PLA2G6_T476I	G	A	PLA2G6	0.5307	1.24383581	0.62871577	2.460774161	PLA2G6-associa	Not_PD_known	Uncertain_significance	TRUE	I	0.72605443	NA	5.00E-06	NA	NA	NA	0.490259587
chr21:32688348	rs532075408	SYNJ1_R309H	C	T	SYNJ1	0.4555	3.693594119	0.119413383	114.2471404	Parkinson_disea	PD_known	Uncertain_significance	FALSE	I	0.35716033	NA	0	0	0	0.61434238	0.056946296
chr12:40364975	rs72547983	LRRK2_L2439I	C	A	LRRK2	0.5651	3.415759946	0.051995817	224.391434	.	Not_PD_known	.	FALSE	G	NA	NA	0	0	0	0.106341146	0.134549679
chr1:155239933	rs78769774	GBA1_R87Q	C	T	GBA1	0.7871	2.917129253	0.001231133	6912.039326	.	Not_PD_known	.	FALSE	G	NA	NA	0	0	0	1	0.051941898
chr12:40299255	rs281865046	LRRK2_L1165P	T	C	LRRK2	0.7084	154.2230606	5.25E-10	4.53E+13	Parkinson_disea	PD_known	Uncertain_significance	FALSE	G	NA	Inf	1.67E-06	0	0	0.094059774	0.386083723
chr15:61961592	rs2303405	VPS13C_Y1302C	T	C	VPS13C	0.3517	1.014301299	0.984335977	1.04517883	not_provided	Not_PD_known	Benign	TRUE	I	1.000000119	NA	0.001306667	NA	NA	NA	1
chr22:38116200	rs587784338	PLA2G6_T585I	G	A	PLA2G6	0.9808	2.719913287	1.17E-35	6.32E+35	Iron_accumulatio	Not_PD_known	Pathogenic	FALSE	G	NA	NA	0	NA	NA	NA	0.051338713
chr12:40251361	rs72546336	LRRK2_N363S	A	G	LRRK2	0.9832	2.721545725	7.41E-41	1.00E+41	Parkinson_disea	PD_known	Uncertain_significance	FALSE	G	NA	NA	0	1.19E-16	0	1	0.052600639
chr12:40299212	rs74985840	LRRK2_A1151T	G	A	LRRK2	0.9712	1.025622761	0.259028807	4.060946197	Parkinson_disea	PD_known	Uncertain_significance	TRUE	G	NA	2.99E-05	1.67E-06	0.004872155	0.016700206	0.057236374	0.405872771
chr3:132538225	rs138367039	DNAJC13_M2225I	G	A	DNAJC13	0.082	1.143049853	0.983120089	1.328996306	not_provided	Not_PD_known	Benign	TRUE	I	0.885704458	0.00105278	5.83E-05	0.011609965	0.016622659	0.023795809	1
chr15:89333364	rs562847013	POLG_Y131H	A	G	POLG	0.3667	1.239861897	0.777344232	1.977576292	Intellectual_disat	Not_PD_known	Conflicting_interpretations_of_pathogenicity	TRUE	I	0.788402557	4.37E-05	6.67E-06	0.017452742	0.045773492	0.120031398	0.825570826
chr21:32688306	rs565013600	SYNJ1_R323K	C	T	SYNJ1	0.8812	30.43520507	1.07E-18	8.66E+20	Parkinson_disea	PD_known	Conflicting_interpretations_of_pathogenicity	FALSE	I	0.320003688	NA	0	0	NA	1	0.054918496
chr3:132523636	rs10935014	DNAJC13_V1995L	G	C	DNAJC13	0.5107	1.086324522	0.848772577	1.390361799	not_provided	Not_PD_known	Likely_benign	TRUE	I	0.824127674	0.000419555	1.83E-05	0.006213306	0.013109135	0.027653861	0.999506735
chr15:61920099	rs115481870	VPS13C_R2482H	C	T	VPS13C	0.3867	1.043833506	0.947132765	1.150407238	not_provided	Not_PD_known	Benign	TRUE	I	0.990095437	NA	0.00012	NA	NA	NA	1
chr3:184331762	rs141379472	EIF4G1_T1478M	C	T	EIF4G1	0.4395	1.392359792	0.601534132	3.22286914	.	Not_PD_known	.	TRUE	I	0.649378896	5.13E-05	3.33E-06	0.004796528	0.019496117	0.079231836	0.516312814
chr2:232839964	rs146430802	GIGYF2_R961Q	G	A	GIGYF2	0.8926	1.028601384	0.683011215	1.549053346	.	Not_PD_known	.	FALSE	I	0.494030625	0.000164571	1.17E-05	0.010293753	0.021267444	0.043932638	0.970368012
chr3:132492416	rs201816934	DNAJC13_R1209H	G	A	DNAJC13	0.6217	3.573694734	0.022687436	562.9236465	.	Not_PD_known	.	TRUE	G	NA	NA	0	1.61E-17	0	0.426730756	0.063358905
chr6:161350211	rs760223151	PRKN_G429E	C	T	PRKN	0.8799	1.165907787	0.159157659	8.540845435	.	Not_PD_known	.	TRUE	G	NA	NA	0	NA	NA	NA	0.163967076
chr1:16986097	rs15786	ATP13A2_P1122L	G	A	ATP13A2	0.5877	1.009646228	0.975220188	1.045287534	Kufor-Rakeb_syr	Not_PD_known	Benign	TRUE	I	0.98444587	NA	0.000976667	NA	NA	NA	1
chr2:232791432	rs34845648	GIGYF2_P423L	C	T	GIGYF2	0.6739	1.221524905	0.481003852	3.102102167	.	Not_PD_known	.	TRUE	I	0.998509109	2.44E-05	1.67E-06	0.005538306	0.020471077	0.075660589	0.443604688
chr1:7984971	rs74315354	PARK7_E163K	G	A	PARK7	0.8994	2.752198371	4.21E-07	17986643.27	.	Not_PD_known	.	TRUE	G	NA	NA	0	0	0	1	0.052656267
chr14:22877045	rs201213246	LRP10_R554X	C	T	LRP10	0.6628	3.856268477	0.008946436	1662.204479	.	Not_PD_known	.	FALSE	G	NA	NA	0	0	0	0.023811542	0.539484784
chr3:184321497	rs116508885	EIF4G1_R305C	C	T	EIF4G1	0.0495	117.0381558	1.010337062	13557.78229	.	Not_PD_known	.	FALSE	I	0.50679934	0	0	0.002359904	0.048745837	1	0.053746197
chr1:65386820	rs761817101	DNAJC6_R278H	G	A	DNAJC6	0.4923	1.602075538	0.41726073	6.15118041	.	Not_PD_known	.	FALSE	I	0.417897224	NA	1.67E-06	NA	NA	NA	0.529021164
chr15:61907289	rs116802310	VPS13C_N3027S	T	C	VPS13C	0.446	4.96E+13	2.97E-22	8.28E+48	.	Not_PD_known	.	FALSE	I	0.576050401	NA	0	NA	NA	NA	0.074094515
chr15:61890334	rs114345245	VPS13C_R3058C	G	A	VPS13C	0.6037	3.559428506	0.029458994	430.0734465	.	Not_PD_known	.	FALSE	I	NA	NA	0	NA	NA	NA	0.241612905
chr1:155241085	rs80356759	NA	C	T	GBA1	0.9803	2.722906838	7.47E-35	9.93E+34	.	Not_PD_known	.	FALSE	G	NA	NA	0	0	0	1	0.051143311
chr22:38135061	rs587784362	PLA2G6_M274R	A	C	PLA2G6	0.989	2.719641309	4.57E-62	1.62E+62	Iron_accumulatio	Not_PD_known	Pathogenic	FALSE	G	NA	NA	0	NA	NA	NA	0.050127047
chr14:22876816	rs74357167	LRP10_D518N	G	A	LRP10	0.202	1.220548075	0.898658665	1.657734647	.	Not_PD_known	.	FALSE	I	0.867262363	0.000182372	1.33E-05	0.003048122	0.021933132	0.157797208	0.992336113
chr14:22876765	rs116010131	LRP10_A501S	G	T	LRP10	0.1006	1.893070284	0.883687311	4.055410843	.	Not_PD_known	.	TRUE	I	0.761851072	5.13E-06	1.67E-06	0.003038112	0.097491673	1	0.424837441
chr6:161350187	rs149953814	PRKN_P437L	G	A	PRKN	0.4797	1.055484602	0.908874735	1.225743992	Parkinson_disea	PD_known	Conflicting_interpretations_of_pathogenicity	TRUE	I	0.938734055	NA	4.17E-05	NA	NA	NA	0.999999913
chr22:38115588	rs587784340	PLA2G6_N658T	T	G	PLA2G6	0.8178	7.571566641	2.50E-07	229773362.4	Iron_accumulatio	Not_PD_known	Likely_pathogenic	TRUE	G	NA	NA	0	NA	NA	NA	0.063692692
chr22:38116119	rs200117092	PLA2G6_R612L	C	A	PLA2G6	0.04684	2.188901894	1.011024325	4.739046713	not_provided	Not_PD_known	Uncertain_significance	FALSE	I	0.444100082	NA	3.33E-06	NA	NA	NA	0.510059163
chr1:155238596	rs80356763	GBA1_R170L	C	A	GBA1	0.7397	337.5791495	4.09E-13	2.79E+17	Gaucher_disease	Not_PD_known	Likely_pathogenic	FALSE	G	NA	NA	0	0	0	1	0.066578657
chr22:38143219	rs150190277	PLA2G6_G2A	C	G	PLA2G6	0.7772	1.092971354	0.590413583	2.023304367	Iron_accumulatio	Not_PD_known	Conflicting_interpretations_of_pathogenicity	TRUE	I	0.932412028	NA	3.33E-06	NA	NA	NA	0.509202798
chr1:155236276	rs74979486	GBA1_R398Q	C	T	GBA1	0.03768	13.53663888	1.159911837	157.9780343	.	Not_PD_known	.	TRUE	G	NA	0	0	0.022426591	0.277201164	1	0.063741996
chr15:61856408	rs138846118	VPS13C_R3652X	G	A	VPS13C	0.497	1.162764082	0.752525798	1.796643137	Frontotemporal_(	Not_PD_known	Uncertain_significance	TRUE	I	0.890146554	NA	1.00E-05	NA	NA	NA	0.859038903
chr3:132492497	rs201263331	DNAJC13_Y1236C	A	G	DNAJC13	0.9387	1.019589403	0.621574192	1.672467365	.	Not_PD_known	.	TRUE	I	0.793126345	8.65E-05	8.33E-06	0.016095741	0.028898052	0.051878985	0.925413284
chr22:38126374	rs139184008	PLA2G6_R475Q	C	T	PLA2G6	0.4301	1.191603644	0.771039249	1.841565454	Infantile_neuroax	Not_PD_known	Uncertain_significance	TRUE	I	0.659831405	NA	8.33E-06	NA	NA	NA	0.939422467
chr15:61983969	rs202117436	VPS13C_Q589X	G	A	VPS13C	0.8391	20.29145781	4.86E-12	8.48E+13	.	Not_PD_known	.	FALSE	G	NA	NA	0	NA	NA	NA	0.107627881
chr20:5109429	rs141394228	TMEM230_M2del	A	G	TMEM230	0.3841	1.149698806	0.839718972	1.5741068	.	Not_PD_known	.	FALSE	I	0.347718	0.000589559	2.33E-05	0.00634008	0.011873283	0.022231938	0.999855914
chr22:38132922	rs587784363	PLA2G6_R329H	C	T	PLA2G6	0.5701	4.394263762	0.026547173	727.3676077	Iron_accumulatio	Not_PD_known	Pathogenic/Likely_pathogenic	TRUE	G	NA	NA	0	NA	NA	NA	0.106411926
chr6:161973401	rs137853058	PRKN_C212Y	C	T	PRKN	0.6764	4.466030577	0.003963261	5032.579728	Parkinson_disea	PD_known	Pathogenic	TRUE	G	NA	NA	0	NA	NA	NA	0.063704999
chr1:155237576	rs74500255	GBA1_F255Y	A	T	GBA1	0.4022	2.270726899	0.333356097	15.46754565	Parkinson_disea	PD_known	Pathogenic/Likely_pathogenic	FALSE	G	NA	0	0	0.007634126	0.075597381	0.748520526	0.065599341
chr15:89321242	rs121918047	POLG_E873X	C	A	POLG	0.9438	2.740115301	1.85E-12	4.05E+12	Progressive_scle	Not_PD_known	Pathogenic	FALSE	G	NA	NA	0	0	0	1	0.051561477
chr12:40356126	rs12581902	LRRK2_N2261I	A	T	LRRK2	0.1811	6.444970119	0.419889373	98.92519911	Parkinson_disea	PD_known	Uncertain_significance	TRUE	G	NA	0	0	0.028485533	0.414952558	1	0.064751727
chr3:132499168	rs147104839	DNAJC13_R1400Q	G	A	DNAJC13	0.7941	1.099878809	0.537836859	2.249257136	not_provided	Not_PD_known	Uncertain_significance	TRUE	I	0.632574022	2.28E-05	3.33E-06	0.013684174	0.043864103	0.140582205	0.637552718
chr3:132467277	rs200653977	DNAJC13_M724I	G	A	DNAJC13	0.9127	1.034791544	0.561731107	1.906238636	.	Not_PD_known	.	TRUE	I	0.783015013	0.000174378	3.33E-06	0.000632122	0.005734654	0.052014989	0.545607522
chr3:184319745	rs13319149	EIF4G1_T161A	A	G	EIF4G1	0.6626	1.040290499	0.87120323	1.24219503	not_specified	Not_PD_known	Benign	TRUE	I	0.600733519	0.098293928	0.006826667	0.015087282	0.020835468	0.028769078	0.999999997
chr22:38169240	rs11570606	PLA2G6_R63G	T	C	PLA2G6	0.372	2.050738557	0.423663403	9.926579922	Infantile_neuroax	Not_PD_known	Benign	TRUE	I	0.816279769	NA	NA	NA	NA	NA	0.246411476

Supplementary Table 9: All meta-analysis variants with gnomAD and meta-analysis frequencies

MarkerName	Ref	Alt	Gene	MAF_meta	MAF_case	MAF_ctrls	AF	AF_popmax	AF_male	AF_female	AF_raw	AF_afr	AF_sas	AF_amr	AF_eas	AF_nfe	AF_fin	AF_asj	AF_oth	non_topmed_AF	non_neuro_AF_p	non_cancer_AF_	controls_AF_popmax
chr1:20638104	C	A	PINK1	0	0	1.44E-06	.	.	.	.	.	.	.	.	.	.	.	.	.	.	.	.	.
chr22:38112571	C	A	PLA2G6	1.00E-04	0.00025621	7.88E-05	.	.	.	.	.	.	.	.	.	.	.	.	.	.	.	.	.
chr3:195867623	C	T	TNK2	0.0177	0.02483233	0.01715523	0.012	0.025	0.0118	0.0122	0.012	0.0037	.	0.0083	0.025	0.017	0.0066	0	0.0111	0.0257	0.025	.	0.0295
chr1:155235727	C	G	GBA1	1.00E-04	0.00014643	6.47E-05	0.0002	0.0006	0.0002	0.0001	0.0004	0.0003	.	0	0.0006	0.0001	0	0	0	0.0007	0.0006	.	0.0011
chr15:89318617	C	T	POLG	0	4.47E-05	3.32E-06	.	.	.	.	.	.	.	.	.	.	.	.	.	.	.	.	.
chr2:232791124	T	A	GIGYF2	0.0006	0.00075627	0.00098505	0.0006	0.001	0.0004	0.0008	0.0006	0	.	0	0	0.001	0	0.0069	0	0.0013	0.0011	.	0.0024
chr1:155238258	G	A	GBA1	0	0	4.87E-06	3.19E-05	0.0001	5.72E-05	0	3.18E-05	0.0001	.	0	0	0	0	0	0	0.0001	0.0003	.	.
chr6:161785877	G	A	PRKN	0.0008	0.0012661	0.00058416	0.0005	0.0009	0.0005	0.0005	0.0005	0	.	0	0	0.0009	0.0003	0	0.0009	0.001	0.0007	.	0.0007
chr12:40322386	G	T	LRRK2	1.00E-04	0.00040845	8.46E-05	.	.	.	.	.	.	.	.	.	.	.	.	.	.	.	.	.
chr15:89333621	T	C	POLG	1.00E-04	0.00137836	0.00039124	0.0031	0.0109	0.0032	0.003	0.0032	0.0109	.	0.0012	0	0	0	0	0.0018	0.0107	0.0118	.	0.0113
chr1:65386873	G	A	DNAJC6	0	0.00012599	1.83E-05	3.18E-05	.	5.72E-05	0	3.18E-05	0	.	0	0	0	0.0003	0	0	.	.	.	.
chr1:175406252	A	T	TNR	1.00E-04	4.20E-05	0.00017737	6.37E-05	0.0001	0.0001	0	6.37E-05	0	.	0	0	0.0001	0	0	0	9.04E-05	7.35E-05	.	.
chr6:162443371	G	A	PRKN	0.0004	0.00057912	0.00026848	0.0002	0.0002	5.73E-05	0.0003	0.0002	0.0002	.	0	0	0.0002	0	0	0	0.0002	0.0003	.	0.0004
chr12:40367045	G	A	LRRK2	0.0002	0.00043211	0.00039404	9.58E-05	0.0001	0.0001	7.21E-05	9.55E-05	0	.	0	0	0.0001	0	0	0.0009	.	0.0001	.	.
chr1:20637956	G	C	PINK1	0	0	2.83E-05	.	.	.	.	.	.	.	.	.	.	.	.	.	.	.	.	.
chr1:16986292	G	A	ATP13A2	0.0002	0.00032411	0.00034477	0.0001	0.0006	0.0002	7.20E-05	0.0001	0.0001	.	0	0.0006	0.0001	0	0	0	0.0007	0.0006	.	0.0002
chr1:65392454	T	A	DNAJC6	0.0007	0.00129632	0.00115746	0.0022	0.006	0.0025	0.0019	0.0022	0.006	.	0.0047	0	0.0006	0	0.0034	0.0037	0.0061	0.0056	.	0.0051
chr1:16987187	G	A	ATP13A2	1.00E-04	0	0	0.0006	0.0023	0.0006	0.0006	0.0006	0.0023	.	0	0	0	0	0	0	0.0023	0.0024	.	0.0023
chr3:184328741	T	C	EIF4G1	6.00E-04	0.0015127	0.00145306	0.0004	0.0005	0.0005	0.0002	0.0004	0.0001	.	0	0	0.0005	0.0006	0	0.0009	0.0007	0.0004	.	0.0005
chr12:40351723	A	G	LRRK2	2.00E-04	0.00032408	0.00036939	0.0004	0.0012	0.0005	0.0002	0.0004	0.0006	.	0.0012	0	0.0003	0	0	0.0009	0.0012	0.0018	.	0.0041
chr15:89318595	T	C	POLG	0.0431	0.05569777	0.04258027	0.0294	0.0434	0.029	0.03	0.0295	0.006	.	0.0236	0	0.0434	0.0368	0.0621	0.034	0.0452	0.0437	.	0.0466
chr3:184322040	C	T	EIF4G1	0.0012	0.00345722	0.00288142	0.001	0.0035	0.001	0.0009	0.001	0.0001	.	0.0035	0	0.0015	0.0006	0	0.0009	0.0037	0.0018	.	0.0041
chr12:40251346	C	T	LRRK2	0.0003	0.00054019	0.00056642	0.0001	0.0002	0.0001	0.0001	0.0001	0.0002	.	0	0	0.0001	0	0	0	0.0002	0.0001	.	.
chr22:38132952	G	A	PLA2G6	0.0005	0.00129632	0.00091117	0.0004	0.001	0.0006	0.0002	0.0004	0.001	.	0	0	0.0003	0	0	0.0009	0.0011	0.0012	.	0.0008
chr15:61929659	G	C	VPS13C	0	0	0	0.0001	0.0026	0.0001	0.0001	0.0001	0	.	0	0.0026	0	0	0	0	0.0026	0.0026	.	0.0044
chr1:155237458	A	C	GBA1	0	7.11E-05	2.98E-06	6.37E-05	0.0001	5.73E-05	7.19E-05	6.37E-05	0	.	0	0	0.0001	0	0	0	9.02E-05	7.35E-05	.	.
chr12:40257283	C	T	LRRK2	0	0	0	0.0001	0.0005	5.73E-05	0.0002	0.0001	0.0005	.	0	0	0	0	0	0	0.0005	0.0006	.	.
chr14:22875400	G	A	LRP10	0.0002	0.00012811	2.63E-05	6.37E-05	0.0001	0	0.0001	6.37E-05	0.0001	.	0	0	6.49E-05	0	0	0	0.0001	0.0003	.	0.0002
chr3:132516425	G	A	DNAJC13	0	NA	NA	0.0003	.	0.0003	0.0001	0.0003	0	.	0	0	0	0.0023	0	0	.	.	.	.
chr1:20633840	C	T	PINK1	0	0	2.14E-05	.	.	.	.	.	.	.	.	.	.	.	.	.	.	.	.	.
chr15:61983908	T	A	VPS13C	1.00E-04	0	0	3.19E-05	6.48E-05	5.73E-05	0	3.18E-05	0	.	0	0	6.48E-05	0	0	0	9.02E-05	7.34E-05	.	0.0002
chr1:20637894	G	A	PINK1	0	0	6.07E-05	3.19E-05	6.48E-05	5.72E-05	0	3.18E-05	0	.	0	0	6.48E-05	0	0	0	9.02E-05	.	.	.
chr15:62008671	T	C	VPS13C	5.00E-04	0.00172842	0.00140377	0.0004	0.0005	0.0005	0.0003	0.0004	0	.	0	0	0.0005	0.0012	0	0	0.0006	0.0005	.	0.0005
chr15:89321776	C	T	POLG	0	0	1.69E-06	3.19E-05	6.49E-05	5.73E-05	0	3.18E-05	0	.	0	0	6.49E-05	0	0	0	.	7.35E-05	.	.
chr1:20644570	C	T	PINK1	0	0	4.21E-05	.	.	.	.	.	.	.	.	.	.	.	.	.	.	.	.	.
chr6:161973403	T	A	PRKN	0	0.00017928	4.38E-05	.	.	.	.	.	.	.	.	.	.	.	.	.	.	.	.	.
chr6:162443438	C	T	PRKN	0	0	4.83E-06	.	.	.	.	.	.	.	.	.	.	.	.	.	.	.	.	.
chr3:184320694	G	A	EIF4G1	0.0004	0.00054013	0.00128063	0.0002	0.0004	0.0003	0	0.0002	0	.	0	0	0.0004	0	0	0	0.0003	0.0004	.	0.0002
chr4:41261921	C	T	UCHL1	0.0005	0.00032408	2.46E-05	0.0002	0.0002	0.0002	0.0001	0.0002	0.0002	.	0	0	6.48E-05	0	0.0034	0.0009	0.0002	0.0003	.	0.0002
chr3:184321882	C	T	EIF4G1	1.00E-04	0.00012811	7.88E-05	9.56E-05	0.0012	5.72E-05	0.0001	9.55E-05	0	.	0.0012	0	0.0001	0	0	0	0.0012	0.0018	.	0.0002
chr22:38120889	G	A	PLA2G6	0	0	4.95E-05	3.19E-05	6.49E-05	0	7.19E-05	3.18E-05	0	.	0	0	6.49E-05	0	0	0	.	7.35E-05	.	.
chr12:40284011	G	T	LRRK2	0.0007	0.00071728	0.00058607	0.0014	0.001	0.0011	0.0019	0.0014	0.0001	.	0	0	0.001	0.0063	0	0.0064	0.0011	0.001	.	0.0018
chr15:89330081	C	G	POLG	1.00E-04	4.20E-05	9.72E-05	3.19E-05	6.48E-05	5.72E-05	0	3.18E-05	0	.	0	0	6.48E-05	0	0	0	9.02E-05	7.34E-05	.	.
chr1:155236384	G	A	GBA1	0	0.00011233	2.45E-05	.	.	.	.	.	.	.	.	.	.	.	.	.	.	.	.	.
chr1:16986321	C	T	ATP13A2	0	0	2.63E-05	0.0001	0.0005	0.0001	0.0001	0.0001	0.0005	.	0	0	0	0	0	0	0.0005	0.0003	.	.
chr3:132502299	G	A	DNAJC13	0.0037	0.00799568	0.00832533	0.0022	0.0037	0.0021	0.0023	0.0022	0.0006	.	0	0	0.0037	0.0012	0.0069	0	0.0032	0.0038	.	0.0021
chr3:184321599	G	C	EIF4G1	0.0002	8.18E-05	0.00013989	6.37E-05	0.0001	5.72E-05	7.18E-05	6.37E-05	0	.	0	0	0.0001	0	0	0	0.0002	0.0001	.	.
chr15:61915890	G	A	VPS13C	0.0002	NA	NA	0.0102	0.0362	0.0111	0.0091	0.0102	0.0362	.	0.0036	0	0	0	0	0.0028	0.0356	0.037	.	0.0374
chr2:232794835	A	C	GIGYF2	6.00E-04	0.00130608	0.00124164	0.0003	0.0006	0.0004	0.0002	0.0003	0.0001	.	0	0	0.0006	0	0	0	0.0006	0.0006	.	0.0005
chr3:132505400	A	T	DNAJC13	0	0	2.64E-05	0.0005	0.002	0.0005	0.0006	0.0005	0.002	.	0	0	0	0	0	0	0.002	0.0024	.	0.0016
chr1:20644665	A	T	PINK1	0.001	0.00110071	0.00072286	0.0011	0.0016	0.0011	0.001	0.0011	0	.	0.0012	0	0.0016	0.0017	0.0034	0.0009	0.0017	0.0018	.	0.0041
chr12:40323256	T	C	LRRK2	4.00E-04	0.00040046	0.00037237	0.0001	0.0003	0.0002	7.19E-05	0.0001	0	.	0	0	0.0003	0	0	0	0.0003	0.0002	.	.
chr15:89330250	A	C	POLG	7.00E-04	NA	NA	.	.	.	.	.	.	.	.	.	.	.	.	.	.	.	.	.
chr15:89330184	G	A	POLG	0.0023	0.00319605	0.00233174	0.0014	0.0023	0.0019	0.0009	0.0014	0.0002	.	0	0	0.0023	0.0012	0.0034	0.0028	0.0019	0.0022	.	0.0011
chr1:155235231	T	C	GBA1	0	0	1.44E-06	.	.	.	.	.	.	.	.	.	.	.	.	.	.	.	.	.
chr4:41261759	A	C	UCHL1	0.0015	0.00291829	0.00359579	0.0008	0.0014	0.0005	0.0012	0.0008	0.0001	.	0	0	0.0014	0.0003	0	0.0009	0.0013	0.0014	.	0.0009
chr22:38133007	G	A	PLA2G6	0.0002	0.00032408	0.00044328	6.37E-05	6.49E-05	0	0.0001	6.37E-05	0	.	0	0	6.49E-05	0	0	0.0009	9.02E-05	7.35E-05	.	.
chr15:61915712	A	G	VPS13C	0.01	0.02063303	0.01923598	0.0067	0.0108	0.0064	0.007	0.0067	0.0021	.	0.0035	0	0.0108	0.0043	0.0069	0.0055	0.0116	0.011	.	0.0127
chr12:40243556	A	T	LRRK2	0	0.00012415	4.10E-06	.	.	.	.	.	.	.	.	.	.	.	.	.	.	.	.	.
chr5:1432618	G	A	SLC6A3	0.0013	0.00259291	0.00268427	0.0011	0.0019	0.001	0.0013	0.0011	0	.	0	0	0.0019	0.0012	0	0.0018	0.0019	0.0018	.	0.0011
chr15:89318641	G	A	POLG	0.0002	4.20E-05	1.83E-05	3.19E-05	0.0006	5.73E-05	0	3.18E-05	0	.	0	0.0006	0	0	0	0	0.0007	0.0006	.	0.0011
chr20:5069196	G	T	TMEM230	0.0023	0.0057254	0.00566433	0.0015	0.0024	0.0015	0.0014	0.0015	0.0007	.	0.0024	0	0.0019	0.002	0.0069	0	0.0025	0.0036	.	0.0024
chr15:62007466	T	C	VPS13C	0	0	0	.	.	.	.	.	.	.	.	.	.	.	.	.	.	.	.	.
chr12:40320042	C	T	LRRK2	0	0	0	.	.	.	.	.	.	.	.	.	.	.	.	.	.	.	.	.
chr3:132522838	C	T	DNAJC13	0.0027	0.00821	0.0061327	0.0021	0.0036	0.0022	0.002	0.0021	0.0006	.	0.0012	0	0.0036	0.0003	0	0.0028	0.0041	0.0038	.	0.0051
chr15:89328795	T	C	POLG	0	0	4.16E-06	.	.	.	.	.	.	.	.	.	.	.	.	.	.	.	.	.
chr16:46674332	T	C	VPS35	1.00E-04	0	0.00031537	3.19E-05	0.0001	5.72E-05	0	3.18E-05	0.0001	.	0	0	0	0	0	0	0.0001	.	.	.
chr15:89325466	C	A	POLG	0	NA	NA	.	.	.	.	.	.	.	.	.	.	.	.	.	.	.	.	.
chr15:89323504	C	T	POLG	1.00E-04	0	0	0.0014	0.0004	0.0013	0.0016	0.0014	0.0001	.	0	0	0.0004	0.0101	0	0.0018	0.0005	0.0004	.	0.0011
chr15:89327201	C	T	POLG	0.0013	0.00156842	0.00095603	0.0004	0.0012	0.0006	0.0002	0.0004	0	.	0.0012	0	0.0007	0.0006	0	0	0.0012	0.0018	.	0.0002
chr3:132522861	G	A	DNAJC13	0	0	0	.	.	.	.	.	.	.	.	.	.	.	.	.	.	.	.	.
chr22:38169326	G	A	PLA2G6	0.0006	0.00194447	0.00165	0.0004	0.0006	5.73E-05	0.0007	0.0004	0	.	0	0	0.0006	0	0	0.0009	0.0009	0.0006	.	0.0005
chr1:155236444	T	A	GBA1	0	0	3.23E-06	.	.	.	.	.	.	.	.	.	.	.	.	.	.	.	.	.
chr1:20649095	A	G	PINK1	0	8.22E-05	3.79E-05	.	.	.	.	.	.	.	.	.	.	.	.	.	.	.	.	.
chr22:32498513	C	T	FBXO7	0.0002	0.00038432	0.00021024	0.0003	0.0003	0.0003	0.0003	0.0003	0	.	0	0	0.0003	0.0012	0	0	0.0005	0.0004	.	0.0009
chr12:40305893	G	T	LRRK2	0	0	1.44E-06	3.19E-05	0.0006	0	7.20E-05	3.18E-05	0	.	0	0.0006	0	0	0	0	0.0007	0.0006	.	.
chr15:89320953	G	A	POLG	0	0	6.87E-06	.	.	.	.	.	.	.	.	.	.	.	.	.	.	.	.	.
chr22:38112165	G	C	PLA2G6	0	0	2.63E-05	0.0004	0.0071	0.0005	0.0002	0.0004	0	.	0	0.0071	0	0	0	0	0.0072	0.0071	.	0.0066
chr12:40240535	G	T	LRRK2	0	0	0	6.37E-05	0.0002	5.73E-05	7.18E-05	6.37E-05	0.0002	.	0	0	0	0	0	0	0.0002	0.0003	.	0.0004
chr21:32641895	C	T	SYNJ1	0	0	1.61E-06	.	.	.	.	.	.	.	.	.	.	.	.	.	.	.	.	.
chr1:20633615	G	A	PINK1	0	0	0	0.0012	0.0041	0.0015	0.0008	0.0011	0.0041	.	0.0014	0	0	0	0	0	0.0041	0.0046	.	0.0045
chr12:40225159	G	A	LRRK2	0	0	3.22E-06	.	.	.	.	.	.	.	.	.	.	.	.	.	.	.	.	.
chr2:232747740	A	G	GIGYF2	6.00E-04	0.00059722	0.00045429	0.0003	0.0004	0.0003	0.0003	0.0003	0.0003	.	0	0	0.0004	0	0	0	0.0005	0.0004	.	0.0008
chr1:17000494	G	A	ATP13A2	0.0008	0.00075618	0.00022164	0.0004	0.0012	0.0006	0.0002	0.0004	0	.	0.0012	0.0006	0.0003	0	0.0207	0.0009	0.0012	0.0018	.	0.0041
chr1:20644515	C	G	PINK1	1.00E-04	0.00017083	7.15E-05	3.19E-05	0.0012	5.72E-05	0	3.18E-05	0	.	0.0012	0	0	0	0	0	0.0012	0.0018	.	.
chr3:132475009	C	A	DNAJC13	0.0085	0.02150189	0.02123467	0.0045	0.0071	0.0045	0.0045	0.0045	0.0023	.	0.0071	0	0.0068	0	0.0034	0.0074	0.0075	0.0069	.	0.0053
chr22:38120886	C	T	PLA2G6	0.0014	0.00334882	0.00376801	0.0009	0.0017	0.001	0.0008	0.0009	0.0001	.	0	0	0.0017	0.0006	0	0	0.0014	0.0017	.	0.0009

chr14:22875924	G	A	LRP10	0.001	0.00129632	0.00120672	0.0017	0.0012	0.0015	0.002	0.0017	0.0001	.	0	0	0.0012	0.0086	0.0034	0.0028	0.0013	0.0013	.	0.002
chr15:89330106	T	A	POLG	0.0008	0.00120422	0.00062017	0.0002	0.0003	0.0002	7.19E-05	0.0002	0	.	0	0	0.0003	0.0003	0	0	0.0002	0.0002	.	.
chr6:161360168	C	T	PRKN	0	0	6.35E-05	.	.	.	.	.	.	.	.	.	.	.	.	.	.	.	.	.
chr15:89333347	G	C	POLG	0	0	0	0.0008	0.0028	0.001	0.0004	0.0008	0.0028	.	0	0	0	0	0	0	0.0028	0.0024	.	0.0016
chr12:40354486	A	T	LRRK2	1.00E-04	0	0.00013141	.	.	.	.	.	.	.	.	.	.	.	.	.	.	.	.	.
chr15:89321217	G	A	POLG	1.00E-04	0.00016253	6.37E-05	6.37E-05	0.0001	0.0001	0	6.37E-05	0.0001	.	0	0	6.48E-05	0	0	0	0.0001	7.35E-05	.	0.0002
chr12:40251369	G	A	LRRK2	0	0	2.50E-05	3.19E-05	6.49E-05	5.73E-05	0	3.18E-05	0	.	0	0	6.49E-05	0	0	0	.	7.35E-05	.	.
chr3:184321315	G	A	EIF4G1	0	0	2.63E-05	.	.	.	.	.	.	.	.	.	.	.	.	.	.	.	.	.
chr1:20648612	G	A	PINK1	0.0002	0.00063778	0.00019622	0.003	0.0031	0.0026	0.0034	0.003	0	.	0	0.0006	0.0031	0.0112	0	0.0055	0.0037	0.0032	.	0.0062
chr15:61915725	G	A	VPS13C	0	0	7.88E-05	0.0002	0.0006	0.0002	0.0002	0.0002	0	.	0	0.0006	0.0001	0	0	0.0037	0.0007	0.0006	.	0.0002
chr15:61961754	G	A	VPS13C	0.0002	0.00038432	0.00052562	3.19E-05	6.49E-05	0	7.19E-05	3.18E-05	0	.	0	0	6.49E-05	0	0	0	9.04E-05	7.35E-05	.	.
chr15:89333627	T	C	POLG	0.0034	0.00583784	0.00616234	0.0131	0.0403	0.0133	0.0129	0.0133	0.0403	.	0.0035	0.0058	0.0025	0.0014	0.0034	0.0046	0.0407	0.0386	.	0.0414
chr1:16986335	G	A	ATP13A2	0.0007	0.00162057	0.00219174	0.0004	0.0008	0.0004	0.0005	0.0004	0.0002	.	0	0	0.0008	0	0	0	0.0008	0.0008	.	0.0013
chr1:16986101	T	A	ATP13A2	0.0059	0.0128593	0.01379616	0.0038	0.0056	0.0037	0.004	0.0036	0.001	.	0.0049	0	0.0056	0.0035	0.0141	0.0039	0.0054	0.0054	.	0.0041
chr1:20637908	C	T	PINK1	0	0	4.15E-07	.	.	.	.	.	.	.	.	.	.	.	.	.	.	.	.	.
chr15:61977193	G	T	VPS13C	0	0	2.63E-05	0.01	0.0353	0.0095	0.0105	0.0099	0.0353	.	0.0024	0	0	0	0	0.0019	0.035	0.0385	.	0.0394
chr15:89327300	A	G	POLG	0	0	2.95E-05	.	.	.	.	.	.	.	.	.	.	.	.	.	.	.	.	.
chr12:40293629	G	A	LRRK2	0	NA	NA	9.57E-05	0.0013	0.0002	0	9.55E-05	0	.	0	0.0013	0	0	0	0.0009	0.0013	0.0013	.	0.0011
chr2:232847564	A	C	GIGYF2	0.0028	NA	NA	.	.	.	.	.	.	.	.	.	.	.	.	.	.	.	.	.
chr15:89326947	C	A	POLG	0.0075	0.01072964	0.00752538	0.0043	0.0065	0.0037	0.005	0.0043	0.0013	.	0.0035	0	0.0065	0.0043	0	0.0037	0.0065	0.0067	.	0.006
chr15:61984938	T	C	VPS13C	0	NA	NA	0.0001	0.0024	0.0002	7.18E-05	0.0001	0.0002	.	0.0024	0	0	0	0	0	0.0025	0.0018	.	0.0041
chr21:32666479	C	T	SYNJ1	0	0	0.00010512	3.19E-05	0.0001	5.72E-05	0	3.18E-05	0.0001	.	0	0	0	0	0	0	0.0001	.	.	.
chr15:89318599	G	A	POLG	0	0	1.79E-05	0.0002	0.0006	0.0001	0.0002	0.0002	0.0006	.	0	0	0	0	0	0	0.0006	0.0003	.	0.0004
chr11:94447275	C	T	MRE11	0.0003	0.00043215	0.00036939	9.56E-05	0.0012	5.73E-05	0.0001	9.55E-05	0	.	0.0012	0	6.48E-05	0	0.0034	0	0.0012	0.0018	.	0.0041
chr20:5109435	C	T	TMEM230	1.00E-04	0.00054013	0.00027092	3.18E-05	6.48E-05	0	7.18E-05	3.18E-05	0	.	0	0	6.48E-05	0	0	0	9.01E-05	7.34E-05	.	.
chr1:155235843	T	C	GBA1	0.0032	0.01420653	0.00585664	0.0016	0.0024	0.0013	0.0021	0.0016	0.0001	.	0.0024	0	0.0018	0.002	0.0278	0.0046	0.0025	0.0036	.	0.0041
chr12:40351680	G	C	LRRK2	0.0008	0.00033344	0.00153428	.	.	.	.	.	.	.	.	.	.	.	.	.	.	.	.	.
chr1:155236367	G	A	GBA1	0	0	4.56E-06	.	.	.	.	.	.	.	.	.	.	.	.	.	.	.	.	.
chr22:38140106	G	A	PLA2G6	0	6.89E-05	6.15E-05	.	.	.	.	.	.	.	.	.	.	.	.	.	.	.	.	.
chr15:61961728	T	C	VPS13C	1.00E-04	0.00012811	0.00010512	3.19E-05	0.0001	0	7.19E-05	3.18E-05	0.0001	.	0	0	0	0	0	0	0.0001	0.0003	.	.
chr22:38169336	C	T	PLA2G6	0.0005	0.00086421	0.00108358	0.0001	0.0012	0	0.0003	0.0001	0.0001	.	0.0012	0	0.0001	0	0	0	0.0012	0.0018	.	.
chr15:89318581	G	A	POLG	0	4.47E-05	4.24E-05	9.55E-05	0.0001	5.72E-05	0.0001	9.55E-05	0.0001	.	0	0	0.0001	0	0	0	0.0001	0.0001	.	.
chr6:162054109	G	C	PRKN	0	0	1.44E-06	.	.	.	.	.	.	.	.	.	.	.	.	.	.	.	.	.
chr1:16985990	C	T	ATP13A2	0.0005	0.00043211	0.00027089	0.0037	0.0124	0.0037	0.0037	0.0037	0.0124	.	0.0024	0	0.0001	0	0.0034	0.0018	0.0125	0.0151	.	0.016
chr22:38115658	G	A	PLA2G6	0	0	9.62E-05	.	.	.	.	.	.	.	.	.	.	.	.	.	.	.	.	.
chr1:65384226	G	A	DNAJC6	0	4.48E-05	0	.	.	.	.	.	.	.	.	.	.	.	.	.	.	.	.	.
chr15:89320890	G	A	POLG	0	0	1.44E-06	9.56E-05	0.0003	0.0001	7.18E-05	9.55E-05	0.0003	.	0	0	0	0	0	0	0.0004	0.0006	.	0.0008
chr15:89319065	G	A	POLG	0	8.94E-05	9.85E-05	9.57E-05	0.0024	0	0.0002	9.55E-05	0	.	0.0024	0	0	0	0	0.0009	0.0025	0.0036	.	0.0041
chr15:61868702	C	T	VPS13C	0.0006	0.0020525	0.00174855	0.0003	0.0005	0.0003	0.0002	0.0003	0.0002	.	0	0	0.0005	0	0	0	0.0003	0.0005	.	0.0004
chr2:232847399	A	G	GIGYF2	0.0021	0.00496975	0.00406364	0.0023	0.0034	0.0019	0.0028	0.0023	0.0008	.	0	0	0.0034	0.0029	0	0.0037	0.0036	0.0034	.	0.0051
chr4:89828156	A	C	SNCA	2.00E-04	0.00108026	0.00044328	9.57E-05	0.0001	0.0001	7.19E-05	9.55E-05	0.0001	.	0	0	0.0001	0	0	0	0.0002	7.35E-05	.	0.0002
chr16:46683521	A	T	VPS35	2.00E-04	0.00097224	0.00036939	6.37E-05	0.0001	5.72E-05	7.18E-05	6.37E-05	0	.	0	0	0.0001	0	0	0	.	0.0001	.	.
chr15:61950998	T	C	VPS13C	0.0771	0.12219261	0.10470651	0.0578	0.0846	0.0572	0.0585	0.0576	0.023	.	0.059	0.0365	0.0846	0.0364	0.0172	0.0655	0.0861	0.0829	.	0.0909
chr6:161350139	C	T	PRKN	0	0	1.95E-05	.	.	.	.	.	.	.	.	.	.	.	.	.	.	.	.	.
chr12:40225558	C	T	LRRK2	0	0	1.36E-06	.	.	.	.	.	.	.	.	.	.	.	.	.	.	.	.	.
chr15:61983868	G	T	VPS13C	0	0	0	0.0003	0.0038	0.0003	0.0003	0.0003	0.0003	.	0	0.0038	0	0	0	0.0009	0.0039	0.0038	.	0.0033
chr2:74530221	T	C	HTRA2	0.0035	0.00853408	0.00839839	0.0016	0.0025	0.0017	0.0016	0.0016	0.0013	.	0	0	0.0025	0	0	0.0018	0.0023	0.0025	.	0.0024
chr21:32665968	A	G	SYNJ1	0.0014	0.00165314	0.00136345	0.0009	0.0017	0.0007	0.0011	0.0009	0.0002	.	0	0	0.0017	0	0	0	0.0013	0.0017	.	0.0009
chr6:161569357	G	A	PRKN	0	0	0	.	.	.	.	.	.	.	.	.	.	.	.	.	.	.	.	.
chr21:32673463	C	T	SYNJ1	0.0003	0.00032408	0.00068954	0.0001	0.0002	0.0002	7.18E-05	0.0001	0.0001	.	0	0	0.0002	0	0	0	0.0003	0.0003	.	0.0004
chr15:89318710	C	T	POLG	0	0	2.53E-06	.	.	.	.	.	.	.	.	.	.	.	.	.	.	.	.	.
chr4:89828149	C	T	SNCA	0	4.92E-05	1.68E-06	.	.	.	.	.	.	.	.	.	.	.	.	.	.	.	.	.
chr15:89328996	G	A	POLG	0.0006	0.00129632	0.00125603	0.0004	0.0006	0.0004	0.0003	0.0004	0.0002	.	0	0	0.0006	0	0	0	0.0005	0.0006	.	0.0004
chr5:1443128	C	T	SLC6A3	0.0006	0.00151237	0.00157612	0.0002	0.0006	0.0001	0.0003	0.0002	0.0002	.	0	0.0006	0.0002	0	0	0	0.0007	0.0006	.	0.0011
chr3:184328662	A	G	EIF4G1	1.00E-04	0	0.00032014	0.0004	0.0038	0.0004	0.0003	0.0004	0.0001	.	0	0.0038	0.0002	0	0	0.0009	0.0039	0.0038	.	0.0022
chr1:20648534	T	C	PINK1	0	0	1.06E-05	.	.	.	.	.	.	.	.	.	.	.	.	.	.	.	.	.
chr1:65392779	A	C	DNAJC6	3.00E-04	0.00032408	0.00039402	0.0003	0.0006	0.0004	0.0002	0.0003	0	.	0	0	0.0006	0	0	0	0.0009	0.0007	.	0.0016
chr15:89317460	G	A	POLG	1.00E-04	0	0.0001314	6.37E-05	6.48E-05	5.72E-05	7.18E-05	6.37E-05	0	.	0	0	6.48E-05	0	0	0.0009	.	7.34E-05	.	.
chr3:132480451	G	A	DNAJC13	0	4.48E-05	2.08E-06	0.0004	0.0003	0.0005	0.0004	0.0004	0	.	0	0	0.0003	0.0023	0	0.0009	0.0005	0.0004	.	0.0007
chr6:161785839	A	T	PRKN	0	0	9.13E-06	.	.	.	.	.	.	.	.	.	.	.	.	.	.	.	.	.
chr3:132499779	G	T	DNAJC13	0.4714	0.49589463	0.47179894	0.6117	0.91	0.6205	0.6007	0.6115	0.8978	.	0.5448	0.91	0.4642	0.4709	0.5034	0.5148	0.9104	0.91	.	0.9125
chr12:40257348	T	G	LRRK2	0	0	1.44E-06	.	.	.	.	.	.	.	.	.	.	.	.	.	.	.	.	.
chr3:184327434	G	A	EIF4G1	0	0	0	0.0039	0.0139	0.0039	0.0038	0.0039	0.0139	.	0	0	0	0	0	0	0.0139	0.0151	.	0.0132
chr6:161785844	A	G	PRKN	0	0	1.06E-05	0.0005	0.0018	0.0005	0.0005	0.0005	0.0018	.	0	0	0	0	0	0	0.0019	0.0027	.	0.0031
chr22:38113591	G	A	PLA2G6	0	0	2.96E-06	.	.	.	.	.	.	.	.	.	.	.	.	.	.	.	.	.
chr1:155236376	C	T	GBA1	0.0124	0.0343975	0.02414806	0.0129	0.0141	0.0115	0.0147	0.0129	0.0025	.	0.0035	0	0.0141	0.04	0.0069	0.0193	0.0138	0.0139	.	0.0134
chr3:184323921	A	G	EIF4G1	7.00E-04	0.00097234	0.00197035	0.0005	0.0012	0.0005	0.0006	0.0005	0.0003	.	0.0012	0.0006	0.0007	0.0003	0	0	0.0012	0.0018	.	0.0041
chr2:232844498	G	C	GIGYF2	0	NA	NA	3.19E-05	0.0001	5.73E-05	0	3.18E-05	0.0001	.	0	0	0	0	0	0	0.0001	.	.	.
chr3:132525718	G	T	DNAJC13	0.0011	0.00216053	0.00246275	0.0005	0.0012	0.0004	0.0006	0.0005	0.0001	.	0.0012	0	0.0008	0	0	0.0018	0.0012	0.0018	.	0.0041
chr1:155239639	A	C	GBA1	0	7.49E-05	1.32E-06	.	.	.	.	.	.	.	.	.	.	.	.	.	.	.	.	.
chr1:155235813	T	G	GBA1	0	8.26E-05	9.22E-06	.	.	.	.	.	.	.	.	.	.	.	.	.	.	.	.	.
chr1:20649034	T	C	PINK1	0	0	2.89E-06	.	.	.	.	.	.	.	.	.	.	.	.	.	.	.	.	.
chr22:38115583	G	A	PLA2G6	0	0	3.80E-06	.	.	.	.	.	.	.	.	.	.	.	.	.	.	.	.	.
chr20:5106224	T	C	TMEM230	0.0013	0.00118842	0.00206861	0.001	0.0011	0.0006	0.0015	0.001	0.0001	.	0	0	0.0011	0.0035	0	0.0018	0.0009	0.001	.	0.0005
chr12:40363440	C	T	LRRK2	0.0002	0.00026482	0.00015569	0.0003	0.0006	0.0005	0.0001	0.0003	0	.	0	0	0.0006	0.0003	0	0	0.0006	0.0005	.	0.0002
chr1:175403578	T	G	TNR	0.0058	0.00802256	0.00567411	0.0034	0.0057	0.0033	0.0036	0.0034	0.001	.	0	0	0.0057	0.0017	0	0.0037	0.0048	0.0056	.	0.0038
chr12:40304040	G	C	LRRK2	0.0004	0.00080936	0.00065653	0.0002	0.0003	0.0002	7.18E-05	0.0002	0	.	0	0	0.0003	0	0	0	0.0003	0.0002	.	.
chr15:61991076	G	C	VPS13C	0	0	0	0.0004	0.0013	0.0003	0.0004	0.0004	0.0013	.	0	0	0	0	0	0	0.0013	0.0009	.	0.0012
chr3:195868341	C	T	TNK2	0.001	0.00248514	0.00167463	0.0008	0.0014	0.0009	0.0007	0.0008	0.0003	.	0	0	0.0014	0	0.0069	0	0.0011	0.0015	.	0.0017
chr2:232747717	G	T	GIGYF2	0.0002	0.00021605	4.93E-05	.	.	.	.	.	.	.	.	.	.	.	.	.	.	.	.	.
chr12:40284061	A	G	LRRK2	0	0	6.84E-06	.	.	.	.	.	.	.	.	.	.	.	.	.	.	.	.	.
chr1:16986248	C	T	ATP13A2	0.4778	0.59473931	0.47216056	0.4139	0.531	0.4136	0.4143	0.4131	0.187	.	0.455	0.2865	0.531	0.4827	0.5034	0.4704	0.5306	0.5325	.	0.5292
chr1:20644526	C	A	PINK1	0	0	3.18E-06	.	.	.	.	.	.	.	.	.	.	.	.	.	.	.	.	.
chr12:40309174	G	A	LRRK2	0	0	1.27E-06	.	.	.	.	.	.	.	.	.	.	.	.	.	.	.	.	.
chr22:38126390	T	C	PLA2G6	5.00E-04	0.00108026	0.00083729	0.0004	0.0007	0.0002	0.0006	0.0004	0	.	0	0	0.0007	0.0003	0	0	0.0009	0.0004	.	0.0002
chr15:61929546	T	A	VPS13C	0	0	0	0.0003	0.0051	0.0002	0.0003	0.0003	0	.	0	0.0051	0	0	0	0	0.0053	0.0051	.	0.0044
chr12:40295466	G	A	LRRK2	0	4.37E-05	1.96E-05	.	.	.	.	.	.	.	.	.	.	.	.	.	.	.	.	.
chr1:16993627	A	C	ATP13A2	0.0142	NA	NA	6.39E-05	0.0001	5.74E-05	7.20E-05	0.0002	0	.	0	0	0.0001	0	0	0	0.0002	0.0001	.	0.0004
chr15:89318677	T	C	POLG	1.00E-04	8.95E-05	7.27E-05	.	.	.	.	.	.	.	.	.	.	.	.	.	.	.	.	.

chr1:17011732	C	G	ATP13A2	0	0	0	0.0009	0.0034	0.0009	0.001	0.001	0.0034	.	0	0	0	0	0	0	0.0033	0.0039	.	0.0032
chr1:155235798	A	G	GBA1	0	0	2.27E-05	.	.	.	.	.	.	.	.	.	.	.	.	.	.	.	.	.
chr21:32673511	T	C	SYNJ1	2.00E-04	0.00010803	0.00014776	6.37E-05	0.0001	5.72E-05	7.18E-05	6.37E-05	0	.	0	0	0.0001	0	0	0	0.0002	0.0001	.	0.0002
chr12:40323300	C	T	LRRK2	0	0.00011979	7.20E-06	.	.	.	.	.	.	.	.	.	.	.	.	.	.	.	.	.
chr15:61951827	T	C	VPS13C	0	0	0	0.0083	0.0297	0.0082	0.0085	0.0084	0.0297	.	0	0	6.49E-05	0	0	0.0018	0.0295	0.034	.	0.0362
chr15:89322799	C	T	POLG	0	0	4.16E-07	6.37E-05	0.0012	0	0.0001	6.37E-05	0	.	0.0012	0	0	0	0	0.0009	0.0012	0.0018	.	.
chr22:38133010	C	T	PLA2G6	0.0002	0	7.39E-05	6.37E-05	6.48E-05	5.72E-05	7.18E-05	6.37E-05	0	.	0	0	6.48E-05	0	0	0.0009	9.02E-05	7.35E-05	.	.
chr1:155236441	T	C	GBA1	0	0	1.44E-06	.	.	.	.	.	.	.	.	.	.	.	.	.	.	.	.	.
chr22:38145447	C	T	PLA2G6	0.0007	0.00097245	0.00125603	0.0004	0.0007	0.0005	0.0004	0.0004	0.0002	.	0	0	0.0007	0	0	0	0.0008	0.0007	.	0.0005
chr1:155237423	G	A	GBA1	0.0002	0.00010803	0.00029552	0.0002	0.0003	0.0002	0.0001	0.0002	0.0001	.	0	0	0.0003	0	0	0	0.0002	0.0003	.	0.0002
chr14:22875772	C	A	LRP10	0	NA	NA	.	.	.	.	.	.	.	.	.	.	.	.	.	.	.	.	.
chr2:232794778	C	T	GIGYF2	0.0002	0.00032408	0.00012314	0.0002	0.0003	0.0002	0.0001	0.0002	0.0002	.	0	0	0.0003	0	0	0	0.0003	0.0003	.	0.0004
chr12:40278154	A	G	LRRK2	0	0	1.77E-05	.	.	.	.	.	.	.	.	.	.	.	.	.	.	.	.	.
chr3:184327609	C	G	EIF4G1	0.005	0.00951043	0.00951067	0.0026	0.0037	0.0027	0.0026	0.0026	0.0013	.	0.0024	0	0.0037	0.0012	0.0103	0.0055	0.0025	0.0036	.	0.0041
chr15:61962790	T	C	VPS13C	0.0252	0.02608567	0.02521096	0.0512	0.197	0.051	0.0515	0.0512	0.0081	.	0.1344	0.197	0.0409	0.1128	0.031	0.0792	0.2	0.197	.	0.1943
chr22:38116105	C	T	PLA2G6	1.00E-04	0.00043211	0.00019701	0.0002	0.0003	5.72E-05	0.0003	0.0002	0.0001	.	0	0	0.0003	0	0	0	0.0003	0.0002	.	.
chr12:40367012	T	A	LRRK2	0	0	2.88E-05	.	.	.	.	.	.	.	.	.	.	.	.	.	.	.	.	.
chr1:17005517	C	T	ATP13A2	0.0002	0.00017343	0.00019859	0.0066	0.0233	0.0064	0.007	0.0066	0.0233	.	0.0012	0	0.0003	0	0	0	0.0235	0.0207	.	0.0206
chr3:184315839	T	C	EIF4G1	6.00E-04	0.00156041	0.00134733	0.0003	0.0006	0.0003	0.0004	0.0003	0	.	0	0	0.0006	0	0	0.0009	0.0005	0.0004	.	.
chr20:5069205	C	T	TMEM230	0	NA	NA	3.19E-05	0.0001	5.72E-05	0	3.18E-05	0.0001	.	0	0	0	0	0	0	0.0001	0.0003	.	0.0004
chr15:89325531	A	C	POLG	0	0	4.23E-06	.	.	.	.	.	.	.	.	.	.	.	.	.	.	.	.	.
chr1:16988226	C	T	ATP13A2	0.0003	0.00118829	0.00039402	9.56E-05	0.0002	0.0001	7.18E-05	9.55E-05	0	.	0	0	0.0002	0	0	0	0.0002	0.0002	.	.
chr15:89318989	G	C	POLG	0	0	6.65E-06	.	.	.	.	.	.	.	.	.	.	.	.	.	.	.	.	.
chr1:20645615	G	A	PINK1	0.0016	0.00203454	0.0009432	0.0005	0.0006	0.0007	0.0003	0.0005	0.0005	.	0	0.0006	0.0006	0	0	0.0009	0.0007	0.0007	.	0.0004
chr3:184320964	C	T	EIF4G1	0	0	0	.	.	.	.	.	.	.	.	.	.	.	.	.	.	.	.	.
chr22:38112186	A	T	PLA2G6	0	0	8.47E-07	.	.	.	.	.	.	.	.	.	.	.	.	.	.	.	.	.
chr12:40351585	G	A	LRRK2	1.00E-04	9.26E-05	6.95E-05	6.37E-05	0.0006	5.73E-05	7.18E-05	6.37E-05	0.0001	.	0	0.0006	0	0	0	0	0.0007	0.0006	.	.
chr1:155238264	C	T	GBA1	1.00E-04	0	0	3.19E-05	0.0001	5.73E-05	0	3.18E-05	0.0001	.	0	0	0	0	0	0	0.0001	.	.	.
chr15:62034998	G	A	VPS13C	0.0002	0.00021605	0.00041866	9.58E-05	0.0001	0	0.0002	9.55E-05	0.0001	.	0	0	6.51E-05	0	0	0.0009	0.0001	0.0003	.	.
chr15:89320832	C	T	POLG	0	0	2.54E-05	.	.	.	.	.	.	.	.	.	.	.	.	.	.	.	.	.
chr22:38112558	C	T	PLA2G6	0	7.60E-05	1.70E-05	.	.	.	.	.	.	.	.	.	.	.	.	.	.	.	.	.
chr15:89327198	T	C	POLG	6.00E-04	0.00172861	0.00147765	0.0006	0.0024	0.0008	0.0004	0.0006	0.0003	.	0.0024	0	0.0008	0.0003	0	0.0009	0.0012	0.0007	.	0.0005
chr3:184331523	C	G	EIF4G1	0	0	0	.	.	.	.	.	.	.	.	.	.	.	.	.	.	.	.	.
chr6:161386864	C	T	PRKN	0	8.22E-05	4.25E-05	.	.	.	.	.	.	.	.	.	.	.	.	.	.	.	.	.
chr1:20649224	C	T	PINK1	0.0002	0	0	3.19E-05	.	0	7.18E-05	3.18E-05	0	.	0	0	0	0	0	0.0009	.	.	.	.
chr1:155238215	T	C	GBA1	0	0.00016342	6.48E-05	9.61E-05	0.0002	0.0001	7.22E-05	9.55E-05	0.0002	.	0	0	0	0.0003	0	0	0.0002	.	.	.
chr15:89328699	G	A	POLG	0	0	3.28E-05	.	.	.	.	.	.	.	.	.	.	.	.	.	.	.	.	.
chr12:40322038	G	A	LRRK2	0	0	4.09E-06	3.20E-05	0.0006	5.75E-05	0	3.18E-05	0	.	0	0.0006	0	0	0	0	0.0007	0.0006	.	0.0011
chr12:40340404	T	C	LRRK2	0	7.56E-05	4.24E-06	.	.	.	.	.	.	.	.	.	.	.	.	.	.	.	.	.
chr12:40293626	G	A	LRRK2	0.0002	0	2.63E-05	.	.	.	.	.	.	.	.	.	.	.	.	.	.	.	.	.
chr1:65392613	C	A	DNAJC6	0	0	0	.	.	.	.	.	.	.	.	.	.	.	.	.	.	.	.	.
chr14:22876078	C	G	LRP10	0	0	0	0.0023	0.0077	0.0021	0.0025	0.0023	0.0077	.	0.0035	0	0	0	0	0.0009	0.0075	0.0089	.	0.0089
chr14:22876722	G	C	LRP10	0.0002	0.00075618	0.00044328	0.0001	0.0001	5.73E-05	0.0002	0.0001	0.0001	.	0	0	0.0001	0.0003	0	0	0.0001	0.0001	.	.
chr1:155237453	C	T	GBA1	0	0.00014219	3.31E-06	9.56E-05	0.0001	0.0002	0	9.55E-05	0.0001	.	0	0	0.0001	0	0	0	0.0001	0.0003	.	0.0004
chr12:40359345	C	T	LRRK2	1.00E-04	0.00038432	0	6.41E-05	0.0001	5.76E-05	7.22E-05	6.37E-05	0	.	0	0	0.0001	0	0	0	9.07E-05	0.0001	.	.
chr12:40315266	T	A	LRRK2	0	0	1.62E-06	.	.	.	.	.	.	.	.	.	.	.	.	.	.	.	.	.
chr1:175406219	T	C	TNR	0.0051	0.00950735	0.01032325	0.0036	0.0106	0.0038	0.0033	0.0036	0.0013	.	0.0106	0	0.0051	0.0029	0.0103	0.0018	0.0099	0.0072	.	0.0041
chr1:16990276	G	C	ATP13A2	0.0008	0.00083442	0.00049565	0.0003	0.0006	0.0004	0.0002	0.0003	0.0001	.	0	0	0.0006	0	0	0	0.0006	0.0004	.	0.0004
chr15:61868675	C	T	VPS13C	1.00E-04	0.00010803	9.85E-05	0.0038	0.0186	0.004	0.0034	0.0038	0.0096	.	0.0024	0.0186	6.48E-05	0	0	0.0018	0.0178	0.0186	.	0.0219
chr15:89326688	G	A	POLG	0	0	1.25E-06	0.0054	0.0189	0.0053	0.0056	0.0054	0.0189	.	0.0035	0	0.0001	0	0	0.0009	0.0189	0.0198	.	0.0206
chr3:132456751	C	T	DNAJC13	0.0002	0.00032408	0.00017238	0.0002	0.0003	0.0002	0.0001	0.0002	0.0003	.	0	0	0.0001	0	0	0	0.0002	0.0006	.	0.0004
chr22:32479132	G	C	FBXO7;FBXO7	1.00E-04	0.00024675	0.00026725	0.0004	0.0006	0.0005	0.0004	0.0004	0	.	0	0	0.0006	0.0003	0	0.0018	0.0008	0.0006	.	0.0011
chr15:62012166	T	C	VPS13C	1.00E-04	7.61E-05	7.15E-05	3.20E-05	6.52E-05	5.76E-05	0	3.18E-05	0	.	0	0	6.52E-05	0	0	0	.	7.39E-05	.	.
chr1:20644551	G	A	PINK1	0	0	1.37E-05	9.56E-05	0.0019	0.0002	0	9.55E-05	0	.	0	0.0019	0	0	0	0	0.002	0.0019	.	0.0011
chr15:89323426	C	G	POLG	6.00E-04	0.00112202	0.00114054	0.0011	0.0006	0.0013	0.0009	0.0011	0.0001	.	0	0	0.0006	0.0075	0	0	0.0005	0.0007	.	0.0007
chr1:20649062	G	A	PINK1	0	0	6.54E-07	.	.	.	.	.	.	.	.	.	.	.	.	.	.	.	.	.
chr12:40294909	C	T	LRRK2	0	0	0	0.0001	0.0012	5.74E-05	0.0002	0.0001	0.0003	.	0.0012	0	0	0	0	0	0.0013	0.0018	.	.
chr20:5111588	G	A	TMEM230	1.00E-04	NA	NA	0.0022	0.0071	0.0023	0.0021	0.0018	0.0071	.	0	0	0	0	0	0.0027	0.007	0.007	.	0.0074
chr1:65366050	A	T	DNAJC6	7.00E-04	0.00140434	0.00115752	0.0004	0.0006	0.0004	0.0003	0.0004	0.0002	.	0	0	0.0006	0	0	0	0.0005	0.0005	.	0.0007
chr22:32475369	G	A	FBXO7	0	0	0	0.002	0.0072	0.0022	0.0018	0.002	0.0072	.	0	0	0	0	0	0	0.0074	0.0044	.	0.0066
chr3:184327433	C	T	EIF4G1	1.00E-04	0.00021605	9.85E-05	9.56E-05	6.49E-05	5.73E-05	0.0001	9.55E-05	0	.	0	0	6.49E-05	0.0006	0	0	.	7.35E-05	.	.
chr1:20638080	C	T	PINK1	1.00E-04	0.00015127	9.52E-05	0.0002	0.0003	0.0002	0.0003	0.0002	0.0003	.	0	0	0.0002	0.0003	0	0	0.0004	0.0003	.	0.0002
chr3:184323209	G	T	EIF4G1	0.0002	0.00075627	0.00076342	9.56E-05	0.0002	0.0001	7.18E-05	9.55E-05	0	.	0	0	0.0002	0	0	0	9.02E-05	0.0002	.	.
chr12:40249843	C	G	LRRK2	3.00E-04	0.00097224	0.00078806	0.0002	0.0004	0.0002	0.0002	0.0002	0	.	0	0	0.0004	0	0	0	0.0003	0.0003	.	.
chr15:89319318	A	C	POLG	6.00E-04	NA	NA	0.0002	0.0004	0.0002	0.0001	0.0004	0	.	0	0	0.0004	0	0	0	0.0006	0.0004	.	0.0009
chr14:22875844	A	G	LRP10	0	0	2.63E-05	.	.	.	.	.	.	.	.	.	.	.	.	.	.	.	.	.
chr3:132457295	T	C	DNAJC13	0	0	0	0.0002	0.0007	0.0002	0.0001	0.0002	0.0007	.	0	0	0	0	0	0	0.0007	0.0009	.	0.0012
chr3:184322835	C	G	EIF4G1	0	0	7.88E-05	.	.	.	.	.	.	.	.	.	.	.	.	.	.	.	.	.
chr6:162443384	G	A	PRKN	0	3.74E-05	7.60E-06	3.19E-05	0.0001	5.72E-05	0	3.18E-05	0.0001	.	0	0	0	0	0	0	0.0001	.	.	.
chr15:62023788	C	T	VPS13C	0	0.00012811	7.88E-05	.	.	.	.	.	.	.	.	.	.	.	.	.	.	.	.	.
chr15:89320850	A	C	POLG	1.00E-04	0.00013416	9.60E-05	.	.	.	.	.	.	.	.	.	.	.	.	.	.	.	.	.
chr1:16996298	G	C	ATP13A2	0.0014	0.00356487	0.00374338	0.0009	0.0015	0.0009	0.0009	0.0009	0.0001	.	0	0	0.0015	0.0009	0	0.0009	0.0014	0.0015	.	0.0022
chr14:22877421	A	C	LRP10	4.00E-04	0.00075618	0.0014037	9.56E-05	0.0002	5.73E-05	0.0001	9.55E-05	0	.	0	0	0.0002	0	0	0	0.0002	0.0001	.	.
chr12:40310435	G	A	LRRK2	0	0.00017498	4.51E-06	3.20E-05	.	0	7.21E-05	3.18E-05	0	.	0	0	0	0	0.0034	0	.	.	.	.
chr1:20644639	G	A	PINK1	0	0	4.13E-05	.	.	.	.	.	.	.	.	.	.	.	.	.	.	.	.	.
chr6:161973347	G	A	PRKN	0	0	0	.	.	.	.	.	.	.	.	.	.	.	.	.	.	.	.	.
chr12:40293644	A	G	LRRK2	0	0	1.61E-06	.	.	.	.	.	.	.	.	.	.	.	.	.	.	.	.	.
chr15:89323445	C	T	POLG	0	8.94E-05	1.95E-05	.	.	.	.	.	.	.	.	.	.	.	.	.	.	.	.	.
chr14:22877070	G	A	LRP10	0.0083	0.0184785	0.01742066	0.0078	0.0095	0.0083	0.0072	0.0078	0.0016	.	0.0012	0	0.0095	0.0173	0.0034	0.0193	0.0094	0.0092	.	0.0096
chr22:38116155	C	T	PLA2G6	0	0	4.99E-06	6.37E-05	0.0002	0.0001	0	6.37E-05	0.0002	.	0	0	0	0	0	0	0.0002	0.0003	.	0.0004
chr15:61983818	A	G	VPS13C	0	0	3.22E-06	.	.	.	.	.	.	.	.	.	.	.	.	.	.	.	.	.
chr12:40321114	A	G	LRRK2	0	0	9.84E-07	.	.	.	.	.	.	.	.	.	.	.	.	.	.	.	.	.
chr1:16997044	C	T	ATP13A2	0.0002	0.00038437	0.00060445	0.0002	0.0003	0.0002	0.0001	0.0002	0.0001	.	0	0	0.0003	0	0	0	0.0003	0.0001	.	.
chr2:232791059	G	C	GIGYF2	1.00E-04	0.00012811	7.88E-05	.	.	.	.	.	.	.	.	.	.	.	.	.	.	.	.	.
chr22:32475067	C	T	FBXO7	0	6.23E-05	2.24E-05	.	.	.	.	.	.	.	.	.	.	.	.	.	.	.	.	.
chr22:38140069	C	T	PLA2G6	1.00E-04	0.00010803	0.00022164	0.0003	0.0006	0.0004	7.19E-05	0.0003	0.0002	.	0	0.0006	0.0003	0	0	0	0.0007	0.0006	.	0.0005
chr1:20633766	C	T	PINK1	0	0	0	0.0006	0.002	0.0007	0.0004	0.0006	0.002	.	0.0012	0	0	0	0	0	0.0019	0.0036	.	0.0031
chr15:89321773	G	T	POLG	0	0	1.27E-06	.	.	.	.	.	.	.	.	.	.	.	.	.	.	.	.	.
chr12:40298433	C	G	LRRK2	1.00E-04	6.20E-05	0.00011991	.	.	.	.	.	.	.	.	.	.	.	.	.	.	.	.	.

chr3:184322631	C	T	EIF4G1	1.00E-04	0	0.00014776	3.18E-05	0.0001	5.72E-05	0	3.18E-05	0.0001	.	0	0	0	0	0	0	0.0001	.	.	.
chr6:161785793	C	G	PRKN	0	0	1.52E-06	.	.	.	.	.	.	.	.	.	.	.	.	.	.	.	.	.
chr15:89322800	G	A	POLG	0	4.47E-05	3.82E-05	.	.	.	.	.	.	.	.	.	.	.	.	.	.	.	.	.
chr2:232844499	G	C	GIGYF2	0	NA	NA	3.19E-05	0.0001	5.73E-05	0	3.18E-05	0.0001	.	0	0	0	0	0	0	0.0001	.	.	.
chr15:89322748	C	T	POLG	0	0	4.16E-05	3.19E-05	.	5.73E-05	0	3.18E-05	0	.	0	0	0	0.0003	0	0	.	.	.	.
chr12:40363541	G	A	LRRK2	1.00E-04	0	2.60E-05	.	.	.	.	.	.	.	.	.	.	.	.	.	.	.	.	.
chr14:22875639	C	T	LRP10	0.0002	0.00043211	0.00024626	0.0064	0.0225	0.0076	0.0049	0.0064	0.0225	.	0.0012	0	0.0002	0	0	0	0.0221	0.0242	.	0.0237
chr3:184322583	G	C	EIF4G1	0.0015	0.00345722	0.00330017	0.0007	0.0035	0.0008	0.0006	0.0008	0.0006	.	0.0035	0	0.0009	0	0	0.0009	0.0025	0.0054	.	0.0002
chr1:16986334	C	T	ATP13A2	0	0.00012811	2.63E-05	0.0054	0.0191	0.0059	0.0048	0.0054	0.0191	.	0.0012	0	0	0	0	0.0018	0.0192	0.0175	.	0.0172
chr1:155235772	C	A	GBA1	0	0.00026254	4.80E-05	.	.	.	.	.	.	.	.	.	.	.	.	.	.	.	.	.
chr22:38140006	G	A	PLA2G6	0.0003	0.00032408	0.00044327	0.0003	0.0006	0.0003	0.0004	0.0003	0	.	0	0.0006	0.0005	0.0006	0	0	0.0007	0.0006	.	0.0011
chrX:121049176	T	G	GLUD2	0.0229	0.02243504	0.02243504	0.0353	0.0555	0.0331	0.0365	0.0361	0.0555	.	0.0112	0	0.0254	0.0472	0.0221	0.0456	0.0562	0.06	.	0.0595
chr12:40322037	C	T	LRRK2	0	0	6.82E-05	.	.	.	.	.	.	.	.	.	.	.	.	.	.	.	.	.
chr1:20644651	C	T	PINK1	0.0002	0.0003062	0.00035368	3.18E-05	0.0001	0	7.18E-05	3.18E-05	0.0001	.	0	0	0	0	0	0	0.0001	.	.	.
chr2:232811255	C	T	GIGYF2	0	0	0	0.0023	0.0079	0.0026	0.0019	0.0023	0.0079	.	0	0	0	0	0	0.0037	0.008	0.008	.	0.0097
chr3:132499777	G	A	DNAJC13	0.0107	0.02551351	0.02291205	0.0081	0.0124	0.0086	0.0074	0.0081	0.0011	.	0.0059	0	0.0124	0.0072	0.0241	0.0138	0.0136	0.0123	.	0.0154
chr15:61910283	C	T	VPS13C	0.4759	0.52836039	0.47743485	0.5538	0.673	0.5427	0.5676	0.5533	0.4817	.	0.673	0.4807	0.5644	0.6923	0.4552	0.5821	0.67	0.6395	.	0.6295
chr15:89325456	G	C	POLG	0	4.48E-05	4.29E-05	.	.	.	.	.	.	.	.	.	.	.	.	.	.	.	.	.
chr15:89323460	C	G	POLG	0.0018	0.00250578	0.00180232	0.0012	0.0019	0.0011	0.0014	0.0012	0.0009	.	0.0012	0	0.0019	0	0	0	0.0017	0.0021	.	0.0012
chr14:22876113	G	A	LRP10	0.0002	0.00054013	0.00029552	6.37E-05	0.0001	5.72E-05	7.18E-05	6.37E-05	0	.	0	0	0.0001	0	0	0	9.02E-05	0.0001	.	.
chr3:132474976	A	G	DNAJC13	0	0	2.63E-05	0.0008	0.0029	0.0009	0.0006	0.0008	0.0029	.	0	0	0	0	0	0	0.0028	0.0038	.	0.0039
chr12:40278179	A	G	LRRK2	1.00E-04	8.57E-05	6.98E-05	3.18E-05	6.48E-05	0	7.18E-05	3.18E-05	0	.	0	0	6.48E-05	0	0	0	9.02E-05	7.34E-05	.	.
chr12:40251480	G	A	LRRK2	0	0	0	.	.	.	.	.	.	.	.	.	.	.	.	.	.	.	.	.
chr15:61941806	T	C	VPS13C	0	0	0	0.0029	0.0101	0.0027	0.0031	0.0029	0.0101	.	0	0	0	0	0	0.0018	0.0101	0.0106	.	0.0105
chr15:89333177	C	T	POLG	0	0	0	0.0006	0.0023	0.0005	0.0009	0.0006	0.0023	.	0	0	0	0	0	0	0.0023	0.003	.	0.0035
chr15:61919368	G	C	VPS13C	0	NA	NA	.	.	.	.	.	.	.	.	.	.	.	.	.	.	.	.	.
chr21:32678644	C	A	SYNJ1	0	0	3.22E-06	.	.	.	.	.	.	.	.	.	.	.	.	.	.	.	.	.
chr15:61962390	T	G	VPS13C	1.00E-04	0.00032408	2.46E-05	0.0009	0.0213	0.0006	0.0013	0.0009	0.0006	.	0.0213	0	0	0	0	0.0046	0.0223	0.0181	.	0.0122
chr21:32639773	G	A	SYNJ1	0	0	4.93E-05	.	.	.	.	.	.	.	.	.	.	.	.	.	.	.	.	.
chr22:38143150	G	A	PLA2G6	1.00E-04	0.00010803	0.00024626	0.0002	0.0035	0.0002	0.0002	0.0002	0.0001	.	0.0035	0	0.0001	0	0	0	0.0025	0.0054	.	0.0081
chr5:1414780	G	A	SLC6A3	0	0	8.31E-07	6.39E-05	0.0001	0	0.0001	6.37E-05	0	.	0	0	0.0001	0	0	0	0.0002	0.0001	.	0.0002
chr1:155236399	G	T	GBA1	0	0	1.13E-05	.	.	.	.	.	.	.	.	.	.	.	.	.	.	.	.	.
chr21:32643457	C	T	SYNJ1	0	0	0	0.0005	0.0016	0.0005	0.0004	0.0005	0.0016	.	0	0	0	0	0	0.0009	0.0016	0.0003	.	0.0004
chr3:132522950	T	G	DNAJC13	8.00E-04	0.00183645	0.0017486	0.0004	0.0007	0.0005	0.0004	0.0004	0.0001	.	0	0	0.0007	0.0003	0	0.0009	0.0004	0.0007	.	0.0002
chr1:20649054	G	A	PINK1	0	0	1.42E-05	.	.	.	.	.	.	.	.	.	.	.	.	.	.	.	.	.
chr15:89318986	G	A	POLG	0	0	2.54E-05	.	.	.	.	.	.	.	.	.	.	.	.	.	.	.	.	.
chr22:38123197	G	A	PLA2G6	0	0	2.49E-06	.	.	.	.	.	.	.	.	.	.	.	.	.	.	.	.	.
chr6:161785805	C	T	PRKN	1.00E-04	0.00024737	5.32E-05	9.55E-05	0.0002	0.0002	0	9.55E-05	0.0002	.	0	0	6.48E-05	0	0	0	0.0002	0.0003	.	0.0004
chr14:22877184	G	A	LRP10	0	0	5.26E-05	0.002	0.007	0.0021	0.0019	0.002	0.007	.	0	0	6.49E-05	0	0	0	0.0071	0.0068	.	0.007
chr12:40335031	G	A	LRRK2	1.00E-04	0.00025945	8.86E-05	3.19E-05	0.0006	0	7.19E-05	3.18E-05	0	.	0	0.0006	0	0	0	0	0.0007	0.0006	.	0.0011
chr15:61920571	T	C	VPS13C	0.0038	0.00713051	0.00598463	0.003	0.0047	0.003	0.0029	0.003	0.0006	.	0.0047	0	0.0045	0.0023	0.0034	0.0055	0.0051	0.0072	.	0.0062
chr6:161548861	C	T	PRKN	0	0	7.62E-06	.	.	.	.	.	.	.	.	.	.	.	.	.	.	.	.	.
chr1:20639934	G	A	PINK1	0	0	1.52E-06	.	.	.	.	.	.	.	.	.	.	.	.	.	.	.	.	.
chr1:20644564	C	A	PINK1	0	0	0	0.0006	0.0022	0.0008	0.0004	0.0006	0.0022	.	0	0	0	0	0	0	0.0022	0.0012	.	0.0016
chr22:32498414	G	A	FBXO7	0.0008	0.00280869	0.00187174	0.0004	0.0009	0.0005	0.0004	0.0004	0	.	0	0	0.0009	0	0	0	0.0008	0.0009	.	0.0005
chr1:17000272	C	T	ATP13A2	0.0169	0.02093607	0.01708981	0.012	0.0196	0.0127	0.0111	0.012	0.0019	.	0.0071	0.0006	0.0196	0.0089	0.0241	0.013	0.0219	0.0194	.	0.0252
chr22:38145597	G	T	PLA2G6	0	0	0	.	.	.	.	.	.	.	.	.	.	.	.	.	.	.	.	.
chr1:155235819	C	T	GBA1	0	0	1.61E-06	.	.	.	.	.	.	.	.	.	.	.	.	.	.	.	.	.
chr12:40299125	A	G	LRRK2	0	0	3.93E-06	.	.	.	.	.	.	.	.	.	.	.	.	.	.	.	.	.
chr1:155236269	C	T	GBA1	0	0	0	0.0009	0.0031	0.0008	0.0009	0.0009	0.0031	.	0	0	0	0	0	0	0.0032	0.0021	.	0.0023
chr15:61983936	T	C	VPS13C	0	0	0	0.0006	0.0023	0.0006	0.0006	0.0006	0.0023	.	0	0	0	0	0	0	0.0023	0.0024	.	0.0023
chr20:5069288	C	T	TMEM230	0.0011	0.00291671	0.00253651	0.0008	0.0012	0.0007	0.0009	0.0008	0.0003	.	0.0012	0	0.0012	0	0	0.0009	0.0014	0.0018	.	0.0041
chr1:17000107	C	T	ATP13A2	1.00E-04	0	4.40E-05	3.19E-05	6.50E-05	5.74E-05	0	3.18E-05	0	.	0	0	6.50E-05	0	0	0	9.05E-05	7.37E-05	.	.
chr6:162443357	G	A	PRKN	0	0	1.97E-05	.	.	.	.	.	.	.	.	.	.	.	.	.	.	.	.	.
chr3:132522832	G	A	DNAJC13	0	0.00012811	5.26E-05	.	.	.	.	.	.	.	.	.	.	.	.	.	.	.	.	.
chr15:61931104	C	G	VPS13C	0.0195	0.04206315	0.04326045	0.0141	0.0194	0.0136	0.0148	0.0142	0.0048	.	0.0118	0	0.0194	0.0225	0	0.0129	0.0187	0.0202	.	0.021
chr15:61918208	T	C	VPS13C	3.00E-04	0.00012811	0.00078875	9.57E-05	0.0012	0.0002	0	9.55E-05	0.0001	.	0.0012	0	6.49E-05	0	0	0	0.0013	0.0018	.	0.0004
chr1:155235765	T	G	GBA1	0	0	1.45E-06	.	.	.	.	.	.	.	.	.	.	.	.	.	.	.	.	.
chr15:61984980	G	T	VPS13C	0	0	0	0.0014	0.0051	0.0013	0.0017	0.0014	0.0051	.	0	0	0	0	0	0.0009	0.005	0.0065	.	0.0066
chr1:20644549	G	A	PINK1	1.00E-04	0.0004949	0.00029634	3.19E-05	6.48E-05	0	7.18E-05	3.18E-05	0	.	0	0	6.48E-05	0	0	0	9.02E-05	7.35E-05	.	.
chr1:16991787	G	A	ATP13A2	0	0	2.37E-05	0.0001	0.0026	0.0002	0	0.0001	0	.	0	0.0026	0	0	0	0	0.0026	0.0026	.	0.0033
chr22:38112534	C	G	PLA2G6	0	4.48E-05	1.96E-05	.	.	.	.	.	.	.	.	.	.	.	.	.	.	.	.	.
chr1:65386886	T	C	DNAJC6	3.00E-04	0.00086421	0.00054178	6.37E-05	0.0001	0.0001	0	6.37E-05	0	.	0	0	0.0001	0	0	0	9.02E-05	0.0001	.	0.0002
chr1:155235197	G	C	GBA1	0	0	9.11E-06	.	.	.	.	.	.	.	.	.	.	.	.	.	.	.	.	.
chr22:38112547	G	A	PLA2G6	0	4.48E-05	5.41E-06	.	.	.	.	.	.	.	.	.	.	.	.	.	.	.	.	.
chr15:62010533	G	A	VPS13C	0.0002	0	4.93E-05	6.37E-05	0.0001	0.0001	0	6.37E-05	0	.	0	0	0.0001	0	0	0	9.02E-05	7.35E-05	.	.
chr15:61991096	T	C	VPS13C	0.0011	0.0015152	0.00095401	0.0025	0.0021	0.0024	0.0026	0.0025	0.0002	.	0	0	0.0021	0.0119	0	0.0019	0.0026	0.0021	.	0.004
chr22:32498466	A	G	FBXO7	0	0	5.26E-05	0.0001	0.0005	5.72E-05	0.0002	0.0001	0.0005	.	0	0	0	0	0	0	0.0005	0.0006	.	0.0004
chr12:40308481	G	A	LRRK2	0.0004	0.00075605	0.00032952	0.0003	0.0003	0.0003	0.0003	0.0003	0.0002	.	0	0	0.0003	0	0	0.0018	0.0002	0.0004	.	0.0002
chr1:65385740	G	A	DNAJC6	0.0006	0.00086421	0.00068957	0.0004	0.0008	0.0003	0.0005	0.0004	0	.	0	0	0.0008	0	0	0	0.0008	0.0009	.	0.0013
chr15:89333357	T	C	POLG	1.00E-04	0.00025621	0.00018396	0.0001	0.0003	5.73E-05	0.0002	0.0001	0	.	0	0	0.0003	0	0	0	0.0003	0.0003	.	.
chr15:89320944	T	G	POLG	0	0	1.69E-06	.	.	.	.	.	.	.	.	.	.	.	.	.	.	.	.	.
chr3:184323468	G	C	EIF4G1	0.0015	0.00432105	0.00354645	0.0007	0.0012	0.0003	0.0011	0.0007	0.0002	.	0	0	0.0012	0	0	0	0.0014	0.0011	.	0.0009
chr22:38113621	C	T	PLA2G6	0.0007	0.00086984	0.00053828	0.001	0.0018	0.001	0.0009	0.001	0	.	0	0	0.0018	0.0003	0	0.0018	0.0024	0.0018	.	0.0038
chr21:32701973	G	A	SYNJ1	0	0	2.63E-05	.	.	.	.	.	.	.	.	.	.	.	.	.	.	.	.	.
chr22:38132881	C	T	PLA2G6	0.0129	0.02583225	0.02467523	0.0084	0.0134	0.0086	0.0082	0.0084	0.0018	.	0.0059	0	0.0134	0.0066	0.0207	0.0074	0.0128	0.0134	.	0.0125
chr22:32498453	C	T	FBXO7	0	6.19E-05	3.69E-05	.	.	.	.	.	.	.	.	.	.	.	.	.	.	.	.	.
chr12:40294866	G	T	LRRK2	0	0	1.87E-05	3.20E-05	6.52E-05	5.75E-05	0	3.18E-05	0	.	0	0	6.52E-05	0	0	0	.	7.39E-05	.	.
chr12:40323270	G	T	LRRK2	0	0	6.45E-06	.	.	.	.	.	.	.	.	.	.	.	.	.	.	.	.	.
chr2:232794852	C	T	GIGYF2	0	0	1.04E-05	.	.	.	.	.	.	.	.	.	.	.	.	.	.	.	.	.
chr21:32664963	C	A	SYNJ1	0.0005	0.00129646	0.00169926	9.55E-05	0.0001	0.0001	7.18E-05	9.55E-05	0.0001	.	0	0	0.0001	0	0	0	0.0001	0.0001	.	.
chr1:17000495	C	T	ATP13A2	0	0.00025621	0	6.38E-05	0.0013	0	0.0001	6.37E-05	0	.	0	0.0013	0	0	0	0	0.0013	0.0013	.	0.0022
chr2:74530433	C	G	HTRA2	0	4.47E-05	8.31E-07	6.37E-05	0.0006	5.73E-05	7.19E-05	6.37E-05	0.0001	.	0	0.0006	0	0	0	0	0.0007	0.0006	.	.
chr12:40319998	T	C	LRRK2	0	0	3.22E-06	.	.	.	.	.	.	.	.	.	.	.	.	.	.	.	.	.
chr6:161548937	G	A	PRKN	0	0	0	0.0001	0.0013	0.0001	0.0001	0.0001	0	.	0	0.0013	6.49E-05	0	0	0.0009	0.0013	0.0013	.	0.0022
chr15:61936716	A	C	VPS13C	0	0	0	0.0019	0.0067	0.0022	0.0014	0.0019	0.0067	.	0	0	0	0	0	0.0009	0.0066	0.008	.	0.0089
chr1:7965399	G	A	PARK7	1.00E-04	0	0	0.0045	0.0158	0.0047	0.0042	0.0045	0.0158	.	0	0	6.48E-05	0	0	0.0009	0.0158	0.0171	.	0.0179
chr15:61977090	T	G	VPS13C	0	0	0	0.0007	0.0148	0.0009	0.0006	0.0007	0	.	0	0.0148	0	0	0	0	0.0152	0.0148	.	0.012
chr15:89327006	C	G	POLG	1.00E-04	5.75E-05	0.00014795	.	.	.	.	.	.	.	.	.	.	.	.	.	.	.	.	.

chr22:38120802	C	T	PLA2G6	0	6.89E-05	4.65E-06	.	.	.	.	.	.	.	.	.	.	.	.	.	.	.	.	.
chr3:184327628	C	T	EIF4G1	0	0	0.00010512	3.19E-05	.	0	7.18E-05	3.18E-05	0	.	0	0	0	0.0003	0	0	.	.	.	.
chr15:89333723	C	T	POLG	0.0005	0.00108026	0.00145299	0.0002	0.0002	0.0001	0.0002	0.0002	0.0001	.	0	0	0.0002	0	0	0.0009	0.0001	0.0003	.	0.0004
chr6:161569358	C	G	PRKN	6.00E-04	0.00083061	0.00050778	0.0002	0.0004	0.0003	0.0001	0.0002	0.0001	.	0	0	0.0004	0	0	0	0.0004	0.0004	.	0.0004
chr15:89318736	C	T	POLG	0	0	2.62E-05	6.37E-05	0.0002	0.0001	0	6.37E-05	0.0002	.	0	0	0	0	0	0	0.0002	.	.	.
chr1:20648577	C	T	PINK1	0	0	2.58E-06	.	.	.	.	.	.	.	.	.	.	.	.	.	.	.	.	.
chr1:155239633	G	A	GBA1	0	0.00024691	1.52E-06	.	.	.	.	.	.	.	.	.	.	.	.	.	.	.	.	.
chr22:38115619	C	T	PLA2G6	0	6.87E-05	1.69E-06	.	.	.	.	.	.	.	.	.	.	.	.	.	.	.	.	.
chr14:22877106	G	A	LRP10	0	0	5.26E-05	9.57E-05	0.0013	0.0001	7.19E-05	9.55E-05	0	.	0	0.0013	6.50E-05	0	0	0	0.0013	0.0013	.	0.0022
chr1:155235196	G	A	GBA1	0.0002	0.00140087	0.00041018	3.19E-05	0.0001	5.72E-05	0	3.18E-05	0.0001	.	0	0	0	0	0	0	0.0001	.	.	.
chr15:61856407	C	T	VPS13C	0.0004	0.00064816	0.00113284	0.0003	0.0005	0.0002	0.0004	0.0003	0.0002	.	0	0	0.0005	0	0	0	0.0005	0.0004	.	.
chr22:32484066	A	G	FBXO7	1.00E-04	0.00012811	5.26E-05	9.55E-05	0.0002	5.72E-05	0.0001	9.55E-05	0.0002	.	0	0	6.48E-05	0	0	0	0.0002	7.34E-05	.	.
chr12:40308507	C	T	LRRK2	0	8.23E-05	1.67E-05	.	.	.	.	.	.	.	.	.	.	.	.	.	.	.	.	.
chr15:61882609	A	G	VPS13C	3.00E-04	0.00032408	0.0009358	9.55E-05	0.0001	0.0001	7.18E-05	9.55E-05	0.0001	.	0	0	0.0001	0	0	0	0.0002	0.0003	.	0.0004
chr12:40293552	A	C	LRRK2	0	0	0	0.0003	0.001	5.73E-05	0.0006	0.0003	0.001	.	0	0	0	0	0	0	0.0011	0.0009	.	0.0008
chr22:38132917	C	T	PLA2G6	1.00E-04	0.00032408	4.93E-05	3.19E-05	6.48E-05	0	7.18E-05	3.18E-05	0	.	0	0	6.48E-05	0	0	0	9.02E-05	7.34E-05	.	.
chr3:132528316	T	G	DNAJC13	0.0045	0.00609738	0.0044913	0.0035	0.0058	0.0037	0.0033	0.0035	0.0011	.	0	0	0.0058	0.0014	0.0034	0.0037	0.0059	0.0062	.	0.0069
chr2:232819840	G	A	GIGYF2	1.00E-04	0	0.00034386	0.0002	0.0003	0.0002	0.0002	0.0002	0	.	0	0	0.0003	0	0	0	0.0002	0.0004	.	0.0002
chr22:38169318	G	A	PLA2G6	1.00E-04	0.00011237	0.00012369	.	.	.	.	.	.	.	.	.	.	.	.	.	.	.	.	.
chr12:40263875	A	G	LRRK2	0	0	3.31E-05	.	.	.	.	.	.	.	.	.	.	.	.	.	.	.	.	.
chr3:132450732	A	T	DNAJC13	2.00E-04	0.00075618	0.00039406	0.0001	0.0002	0.0001	0.0001	0.0001	0.0001	.	0	0	0.0002	0	0	0	0.0003	0.0003	.	0.0004
chr1:16986554	G	A	ATP13A2	0.0006	0.00010803	0.00034477	0.0002	0.0001	0.0003	0.0001	0.0002	0.0001	.	0	0	6.50E-05	0	0.0103	0.0018	0.0001	.	.	0.0004
chr14:22875363	A	G	LRP10	6.00E-04	0.00137836	0.00117371	0.0196	0.0679	0.0193	0.02	0.0197	0.0679	.	0.0059	0	0.0007	0	0	0.0074	0.0683	0.0681	.	0.0681
chr15:89318676	A	G	POLG	0	0	5.91E-06	3.19E-05	0.0006	5.72E-05	0	3.18E-05	0	.	0	0.0006	0	0	0	0	0.0007	0.0006	.	0.0011
chr5:1394741	C	G	SLC6A3	4.00E-04	0.00118829	0.00098505	0.0003	0.0006	0.0003	0.0003	0.0003	0.0001	.	0	0	0.0006	0	0	0	0.0008	0.0006	.	0.0011
chr15:89321223	T	C	POLG	0	0	3.74E-06	9.56E-05	0.0003	0	0.0002	9.55E-05	0.0003	.	0	0	0	0	0	0	0.0004	0.0006	.	0.0008
chr15:89330257	G	A	POLG	0	6.19E-05	2.32E-05	.	.	.	.	.	.	.	.	.	.	.	.	.	.	.	.	.
chr1:20648528	G	A	PINK1	0.0003	0.00061033	0.00022003	0.0004	0.0009	0.0003	0.0005	0.0004	0.0009	.	0	0	0.0001	0	0	0.0018	0.0009	0.0009	.	0.0004
chr22:38112541	G	A	PLA2G6	0	0	9.56E-06	.	.	.	.	.	.	.	.	.	.	.	.	.	.	.	.	.
chr15:89320907	T	C	POLG	0	0	2.12E-06	.	.	.	.	.	.	.	.	.	.	.	.	.	.	.	.	.
chr14:22875648	G	A	LRP10	0.0002	0.00054013	0.0005664	0.0001	0.0003	0.0002	7.18E-05	0.0001	0	.	0	0	0.0003	0	0	0	0.0002	0.0002	.	0.0002
chr1:155236331	C	T	GBA1	0	0	1.61E-06	.	.	.	.	.	.	.	.	.	.	.	.	.	.	.	.	.
chr1:17004708	G	A	ATP13A2	0	0	7.39E-05	0.0003	0.0005	0.0002	0.0003	0.0003	0	.	0	0	0.0005	0	0	0	0.0007	0.0004	.	0.0009
chr3:132466351	A	C	DNAJC13	4.00E-04	0.00075618	0.00078804	0.0007	0.0008	0.0009	0.0005	0.0007	0.0001	.	0	0	0.0008	0.0023	0	0.0009	0.0007	0.0009	.	0.0011
chr3:132523168	A	C	DNAJC13	1.00E-04	0	4.93E-05	0.004	0.0139	0.0045	0.0034	0.004	0.0139	.	0	0	0.0001	0	0	0.0018	0.014	0.0139	.	0.0148
chr21:32656885	A	G	SYNJ1	0	0	4.15E-07	0.0003	0.0012	0.0005	0.0001	0.0003	0.0009	.	0.0012	0	0	0	0	0.0009	0.0012	0.0018	.	0.0012
chr1:155238209	G	T	GBA1	0	0	1.77E-05	.	.	.	.	.	.	.	.	.	.	.	.	.	.	.	.	.
chr15:61922557	A	G	VPS13C	0.001	0.00194447	0.00256113	0.0005	0.001	0.0005	0.0006	0.0005	0.0002	.	0	0	0.001	0	0	0	0.0008	0.001	.	0.0008
chr15:89320998	C	T	POLG	0	0	8.45E-07	.	.	.	.	.	.	.	.	.	.	.	.	.	.	.	.	.
chr12:40257264	A	G	LRRK2	0	0	1.30E-05	.	.	.	.	.	.	.	.	.	.	.	.	.	.	.	.	.
chr22:38132914	A	G	PLA2G6	0	0	4.24E-06	.	.	.	.	.	.	.	.	.	.	.	.	.	.	.	.	.
chr3:184327694	A	G	EIF4G1	0	0	0.00010514	0.001	0.0032	0.0012	0.0006	0.001	0.0032	.	0	0.0006	0	0	0	0.0009	0.0033	0.0035	.	0.0031
chr16:46682118	G	A	VPS35	0	0	8.31E-07	.	.	.	.	.	.	.	.	.	.	.	.	.	.	.	.	.
chr1:155235195	C	T	GBA1	0	3.74E-05	1.32E-05	.	.	.	.	.	.	.	.	.	.	.	.	.	.	.	.	.
chr1:155235006	G	A	GBA1	0	NA	NA	0.0025	0.009	0.0028	0.0021	0.0025	0.009	.	0	0	0	0	0	0	0.009	0.0079	.	0.0092
chr15:62008730	A	G	VPS13C	4.00E-04	0.00089675	0.0012615	0.0001	0.0003	0.0001	0.0001	0.0001	0	.	0	0	0.0003	0	0	0	0.0003	0.0001	.	.
chr3:184317393	C	T	EIF4G1	0	0	5.91E-06	.	.	.	.	.	.	.	.	.	.	.	.	.	.	.	.	.
chr22:38169423	G	T	PLA2G6	0	0	1.40E-05	.	.	.	.	.	.	.	.	.	.	.	.	.	.	.	.	.
chr12:40363410	A	G	LRRK2	4.00E-04	0.00024528	0.00085146	.	.	.	.	.	.	.	.	.	.	.	.	.	.	.	.	.
chr4:41263258	T	C	UCHL1	0	0	0	.	.	.	.	.	.	.	.	.	.	.	.	.	.	.	.	.
chr15:89316763	C	A	POLG	0.08	0.09848217	0.08094648	0.0679	0.0896	0.0632	0.0737	0.068	0.0127	.	0.0401	0	0.0896	0.1422	0.0417	0.0893	0.0921	0.0884	.	0.0984
chr15:61922408	C	T	VPS13C	0.0783	0.09811217	0.07924864	0.0575	0.084	0.0569	0.0583	0.0575	0.023	.	0.0579	0.0366	0.084	0.0363	0.0172	0.0664	0.0856	0.0824	.	0.0911
chr6:161360187	T	C	PRKN	0	0	1.52E-06	6.37E-05	0.0013	5.72E-05	7.18E-05	6.37E-05	0	.	0	0.0013	0	0	0	0	0.0013	0.0013	.	0.0011
chr6:162262681	C	T	PRKN	0	0	3.19E-05	.	.	.	.	.	.	.	.	.	.	.	.	.	.	.	.	.
chr15:62013943	A	G	VPS13C	0	0	0	3.19E-05	0.0001	5.73E-05	0	3.18E-05	0.0001	.	0	0	0	0	0	0	0.0001	0.0003	.	.
chr12:40263861	A	G	LRRK2	1.00E-04	0.00018608	0.00012588	9.55E-05	0.0002	0.0002	0	9.55E-05	0	.	0	0	0.0002	0	0	0	.	0.0002	.	.
chr15:89321780	G	A	POLG	0.0002	4.20E-05	5.82E-06	3.19E-05	6.48E-05	5.73E-05	0	3.18E-05	0	.	0	0	6.48E-05	0	0	0	9.03E-05	7.35E-05	.	0.0002
chr15:89318553	T	C	POLG	0	0	1.54E-05	.	.	.	.	.	.	.	.	.	.	.	.	.	.	.	.	.
chr22:38132850	G	A	PLA2G6	0	8.97E-05	1.88E-05	3.19E-05	6.48E-05	5.73E-05	0	3.18E-05	0	.	0	0	6.48E-05	0	0	0	.	7.34E-05	.	.
chr17:46010389	C	T	MAPT	0	0	1.37E-05	.	.	.	.	.	.	.	.	.	.	.	.	.	.	.	.	.
chr15:62035002	C	A	VPS13C	0	0	0	0.0004	0.0015	0.0005	0.0004	0.0004	0.0015	.	0	0	0	0	0	0	0.0015	0.0009	.	0.0016
chr1:155235823	C	T	GBA1	0	3.27E-05	0.00013884	3.19E-05	6.48E-05	5.72E-05	0	3.18E-05	0	.	0	0	6.48E-05	0	0	0	9.02E-05	.	.	.
chr1:16987157	C	T	ATP13A2	0.0002	0.00036124	0.00033177	0.0005	0.0017	0.0006	0.0004	0.0005	0.0017	.	0	0	6.49E-05	0	0	0	0.0018	0.0009	.	0.0008
chr15:61922680	G	T	VPS13C	1.00E-04	0.00010803	0.00017238	6.38E-05	0.0001	0.0001	0	6.37E-05	0.0001	.	0	0	6.49E-05	0	0	0	0.0001	0.0003	.	0.0004
chr1:16986065	C	T	ATP13A2	0.0002	0.00021605	0.00051716	0.0002	0.0003	0.0002	0.0002	0.0002	0.0003	.	0	0	0.0002	0	0	0	0.0004	0.0006	.	0.0004
chr1:20644626	C	G	PINK1	0	0	0	0.0001	0.0005	0.0002	7.18E-05	0.0001	0.0005	.	0	0	0	0	0	0	0.0005	0.0006	.	0.0008
chr22:38123244	A	T	PLA2G6	0	0	3.39E-06	.	.	.	.	.	.	.	.	.	.	.	.	.	.	.	.	.
chr15:61915865	G	C	VPS13C	0	0	0	0.0001	0.0005	0.0001	0.0001	0.0001	0.0005	.	0	0	0	0	0	0	0.0005	0.0006	.	0.0008
chr21:32726855	T	C	SYNJ1	0	0	0	3.19E-05	6.49E-05	0	7.19E-05	3.18E-05	0	.	0	0	6.49E-05	0	0	0	.	7.35E-05	.	.
chr15:61940726	G	A	VPS13C	0	0	2.63E-05	0.0007	0.0128	0.0009	0.0004	0.0007	0.0001	.	0	0.0128	0	0	0	0	0.0132	0.0128	.	0.0109
chr3:184322551	C	T	EIF4G1	1.00E-04	0	4.93E-05	.	.	.	.	.	.	.	.	.	.	.	.	.	.	.	.	.
chr3:184323412	G	C	EIF4G1	0.0003	0.00086421	0.00071418	0.0002	0.0003	0.0003	7.18E-05	0.0002	0.0001	.	0	0	0.0003	0	0	0	0.0003	0.0003	.	.
chr3:132516758	G	A	DNAJC13	0	0	0	0.0026	0.0092	0.0027	0.0024	0.0026	0.0092	.	0	0	6.48E-05	0	0	0	0.0092	0.0083	.	0.0097
chr1:16997107	G	A	ATP13A2	1.00E-04	0.00026936	0.00011403	6.37E-05	0.0002	5.73E-05	7.18E-05	6.37E-05	0.0002	.	0	0	0	0	0	0	0.0002	0.0003	.	0.0004
chr12:40351579	C	T	LRRK2	0	0	2.60E-05	3.19E-05	0.0001	0	7.18E-05	3.18E-05	0.0001	.	0	0	0	0	0	0	0.0001	.	.	.
chr15:89333327	G	A	POLG	0	4.49E-05	4.06E-05	6.37E-05	0.0001	5.73E-05	7.18E-05	6.37E-05	0	.	0	0	0.0001	0	0	0	0.0002	0.0001	.	.
chr22:38126451	T	C	PLA2G6	0	4.48E-05	2.71E-05	.	.	.	.	.	.	.	.	.	.	.	.	.	.	.	.	.
chr1:16986291	C	T	ATP13A2	0.0002	0.00032411	0.00041866	9.58E-05	0.0024	5.74E-05	0.0001	9.55E-05	0	.	0.0024	0	6.50E-05	0	0	0	0.0025	0.0036	.	.
chr15:61978646	T	C	VPS13C	8.00E-04	0.00021605	0.00022164	0.0001	0.0012	0.0001	0.0001	0.0001	0	.	0.0012	0	6.48E-05	0	0.0034	0.0009	0.0012	0.0018	.	.
chr15:89320856	C	T	POLG	0	0	4.16E-06	.	.	.	.	.	.	.	.	.	.	.	.	.	.	.	.	.
chr1:155238214	A	C	GBA1	0	0	2.56E-05	3.20E-05	.	0	7.21E-05	3.18E-05	0	.	0	0	0	0.0003	0	0	.	.	.	.
chr1:17000429	T	C	ATP13A2	0	NA	NA	.	.	.	.	.	.	.	.	.	.	.	.	.	.	.	.	.
chr6:161785778	A	C	PRKN	0	0	6.69E-07	.	.	.	.	.	.	.	.	.	.	.	.	.	.	.	.	.
chr12:40310515	A	G	LRRK2	0	0	1.37E-05	.	.	.	.	.	.	.	.	.	.	.	.	.	.	.	.	.
chr15:61922527	G	A	VPS13C	0.0004	0.00012811	0.00042052	0.0001	0.0001	0.0001	0.0001	0.0001	0	.	0	0	0.0001	0.0006	0	0	0.0002	0.0001	.	.
chr3:132499231	C	T	DNAJC13	0.0186	0.02426586	0.01889098	0.0229	0.0277	0.0227	0.0231	0.0229	0.0277	.	0.0177	0	0.0193	0.04	0.0138	0.0202	0.0272	0.0289	.	0.0292
chr15:61966088	C	T	VPS13C	1.00E-04	0	4.93E-05	.	.	.	.	.	.	.	.	.	.	.	.	.	.	.	.	.
chr15:89323462	T	C	POLG	3.00E-04	0.00086421	0.00073883	0.0005	0.001	0.0003	0.0009	0.0005	0.001	.	0	0	0.0004	0	0	0.0018	0.0011	0.0018	.	0.0008
chr12:40232383	A	G	LRRK2	0	0	2.63E-05	0.001	0.0034	0.001	0.001	0.001	0.0034	.	0.0012	0	0	0	0	0	0.0035	0.003	.	0.0031

chr1:155238144	A	G	GBA1	0	0	3.27E-07	.	.	.	.	.	.	.	.	.	.	.	.	.	.	.	.	.
chr1:20644627	C	T	PINK1	0	0	1.29E-05	.	.	.	.	.	.	.	.	.	.	.	.	.	.	.	.	.
chr1:155238246	G	A	GBA1	0	8.23E-05	3.04E-06	.	.	.	.	.	.	.	.	.	.	.	.	.	.	.	.	.
chr15:89330133	C	G	POLG	0.0046	0.00717541	0.00451239	0.003	0.0083	0.0024	0.0037	0.003	0.0008	.	0.0083	0	0.004	0.0046	0	0.0028	0.0062	0.009	.	0.0035
chr1:16992115	C	T	ATP13A2	0	0	0	0.0003	0.0009	0.0002	0.0004	0.0003	0.0009	.	0	0	0	0	0	0	0.0009	0.0006	.	0.0008
chr6:161785825	T	C	PRKN	1.00E-04	0.00016464	8.70E-05	3.18E-05	6.48E-05	0	7.18E-05	3.18E-05	0	.	0	0	6.48E-05	0	0	0	9.02E-05	7.34E-05	.	.
chr15:61940651	A	G	VPS13C	1.00E-04	0	2.46E-05	0.0009	0.0033	0.001	0.0008	0.0009	0.0033	.	0	0	0	0	0	0	0.0034	0.003	.	0.0031
chr2:232794843	C	A	GIGYF2	0.0194	0.02086171	0.01938848	0.0351	0.2143	0.0355	0.0345	0.0352	0.0079	.	0.0684	0.2143	0.0216	0.0718	0.031	0.045	0.2117	0.2143	.	0.2303
chr1:16990191	C	T	ATP13A2	0	0	7.88E-05	0.0011	0.004	0.0011	0.0012	0.0011	0.004	.	0	0	6.49E-05	0	0	0	0.004	0.003	.	0.0019
chr12:40340380	T	C	LRRK2	0	0	1.29E-06	3.19E-05	0.0006	5.72E-05	0	3.18E-05	0	.	0	0.0006	0	0	0	0	0.0007	0.0006	.	0.0011
chr21:32631012	C	A	SYNJ1	0	0	0	6.37E-05	0.0002	5.72E-05	7.18E-05	6.37E-05	0.0002	.	0	0	0	0	0	0	0.0002	0.0006	.	0.0008
chr1:155237444	A	G	GBA1	0	0	8.31E-07	.	.	.	.	.	.	.	.	.	.	.	.	.	.	.	.	.
chr12:40309225	A	C	LRRK2	0	0	4.22E-07	.	.	.	.	.	.	.	.	.	.	.	.	.	.	.	.	.
chr1:155239736	G	A	GBA1	0	0	7.59E-06	.	.	.	.	.	.	.	.	.	.	.	.	.	.	.	.	.
chr3:184323541	C	T	EIF4G1	0	0	0	0.0007	0.0022	0.0009	0.0004	0.0007	0.0022	.	0	0	0.0001	0	0	0	0.0021	0.0015	.	0.0016
chr1:7965425	G	C	PARK7	0	0	1.62E-06	3.19E-05	0.0006	5.72E-05	0	3.18E-05	0	.	0	0.0006	0	0	0	0	0.0007	0.0006	.	0.0011
chr15:89328532	G	C	POLG	0.0014	0.00227314	0.00116605	0.0021	0.0024	0.0019	0.0023	0.0021	0.0007	.	0.0024	0	0.0016	0.0092	0	0.0009	0.0025	0.0018	.	0.0015
chr12:40302860	G	A	LRRK2	0	NA	NA	.	.	.	.	.	.	.	.	.	.	.	.	.	.	.	.	.
chr15:62023836	C	T	VPS13C	0.0221	0.02313678	0.02209955	0.0623	0.1938	0.0621	0.0625	0.0623	0.0576	.	0.1312	0.1938	0.0365	0.1099	0.0345	0.0773	0.1967	0.1938	.	0.19
chr12:40232385	T	C	LRRK2	0	0	1.52E-06	0.001	0.0034	0.001	0.001	0.001	0.0034	.	0.0012	0	0	0	0	0	0.0035	0.003	.	0.0031
chr1:20649134	G	A	PINK1	0	8.95E-05	7.73E-05	.	.	.	.	.	.	.	.	.	.	.	.	.	.	.	.	.
chr1:7984981	T	C	PARK7	0	0	1.00E-06	.	.	.	.	.	.	.	.	.	.	.	.	.	.	.	.	.
chr2:74532831	G	A	HTRA2	0	0	0	9.56E-05	0.0003	5.73E-05	0.0001	9.55E-05	0.0003	.	0	0	0	0	0	0	0.0004	0.0003	.	0.0008
chr14:22877128	G	T	LRP10	1.00E-04	0	0	0.0002	0.0045	0.0003	7.18E-05	0.0003	0	.	0	0.0045	0	0	0	0	0.0046	0.0045	.	0.0066
chr3:132503289	G	A	DNAJC13	0	0	0	.	.	.	.	.	.	.	.	.	.	.	.	.	.	.	.	.
chr15:61934224	T	G	VPS13C	0.0081	0.00883619	0.00807773	0.0181	0.0571	0.0189	0.0171	0.0181	0.0333	.	0.0083	0.0571	0.0069	0.0124	0.0103	0.0267	0.0565	0.0571	.	0.0557
chr5:1403013	G	A	SLC6A3	0.0015	0.00334126	0.0010762	0.0005	0.0008	0.0007	0.0001	0.0005	0.0001	.	0	0	0.0008	0	0	0.0009	0.0005	0.0009	.	0.0004
chr12:40323255	A	G	LRRK2	0	0	3.23E-06	.	.	.	.	.	.	.	.	.	.	.	.	.	.	.	.	.
chr2:232794782	G	C	GIGYF2	0.0003	0.00102498	0.00076216	6.37E-05	0.0001	5.72E-05	7.18E-05	6.37E-05	0	.	0	0	0.0001	0	0	0	.	0.0001	.	.
chr6:161360129	G	T	PRKN	0	0	1.14E-05	.	.	.	.	.	.	.	.	.	.	.	.	.	.	.	.	.
chr15:89318737	G	A	POLG	0	4.47E-05	4.87E-05	3.19E-05	0.0001	5.73E-05	0	3.18E-05	0.0001	.	0	0	0	0	0	0	0.0001	0.0003	.	0.0004
chr15:61854543	T	C	VPS13C	0.0055	0.00994057	0.01046979	0.0029	0.0043	0.0033	0.0025	0.003	0.0011	.	0.0024	0	0.0043	0.002	0.0103	0.0028	0.0034	0.0044	.	0.0081
chr15:89319073	A	G	POLG	8.00E-04	0.00140434	0.0019701	0.0001	0.0001	0.0002	7.18E-05	0.0001	0.0001	.	0	0	0.0001	0	0	0.0009	0.0001	0.0001	.	.
chr12:40340400	G	A	LRRK2	0.0007	0.01370622	0.0010898	0.0002	0.0003	0.0002	0.0002	0.0002	0.0001	.	0	0	0.0003	0	0.0034	0	0.0002	0.0003	.	.
chr15:89318962	C	T	POLG	0	0	2.88E-05	.	.	.	.	.	.	.	.	.	.	.	.	.	.	.	.	.
chr3:132454135	C	T	DNAJC13	0	0.00012641	6.72E-05	6.54E-05	0.0001	0	0.0001	6.37E-05	0.0001	.	0	0	0	0	0	0.0009	0.0001	0.0003	.	0.0004
chr15:89325591	A	G	POLG	1.00E-04	4.20E-05	7.43E-05	3.19E-05	6.48E-05	5.73E-05	0	3.18E-05	0	.	0	0	6.48E-05	0	0	0	9.02E-05	7.35E-05	.	.
chr15:62044256	C	T	VPS13C	1.00E-04	0.00017923	0.00014635	6.38E-05	0.0001	5.74E-05	7.20E-05	6.37E-05	0	.	0	0	0.0001	0	0	0	.	0.0001	.	.
chr15:89321777	G	A	POLG	0	0	5.02E-05	6.37E-05	0.0001	0	0.0001	6.37E-05	0	.	0	0	0.0001	0	0	0	0.0002	7.35E-05	.	.
chr22:38132979	A	T	PLA2G6	0	0	6.55E-07	.	.	.	.	.	.	.	.	.	.	.	.	.	.	.	.	.
chr21:32657057	G	A	SYNJ1	0	0	0	.	.	.	.	.	.	.	.	.	.	.	.	.	.	.	.	.
chr1:175386035	T	C	TNR	8.00E-04	0.00172842	0.00231499	0.0003	0.0012	0.0003	0.0002	0.0003	0.0002	.	0.0012	0	0.0004	0	0	0	0.0012	0.0004	.	0.0004
chr1:155235829	C	A	GBA1	0	0	4.69E-06	.	.	.	.	.	.	.	.	.	.	.	.	.	.	.	.	.
chr15:61967438	C	T	VPS13C	0.4204	0.44488621	0.42071709	0.656	0.8715	0.6688	0.64	0.655	0.8715	.	0.5571	0.8458	0.5684	0.465	0.6034	0.5915	0.8715	0.8718	.	0.8694
chr15:89323451	T	C	POLG	2.00E-04	0	7.88E-05	3.19E-05	6.48E-05	0	7.18E-05	3.18E-05	0	.	0	0	6.48E-05	0	0	0	.	7.35E-05	.	.
chr15:89330258	C	G	POLG	6.00E-04	0.00151237	0.00137911	0.0004	0.0007	0.0005	0.0002	0.0004	0.0001	.	0	0	0.0007	0	0	0	0.0004	0.0007	.	0.0004
chr2:232832882	G	A	GIGYF2	0.0002	0.00051249	0.00063075	9.56E-05	0.0002	0.0002	0	9.55E-05	0	.	0	0	0.0002	0	0	0	9.02E-05	0.0002	.	.
chr17:46024061	C	T	MAPT	0	6.52E-05	2.45E-05	.	.	.	.	.	.	.	.	.	.	.	.	.	.	.	.	.
chr1:155236426	G	A	GBA1	0	0	5.75E-06	.	.	.	.	.	.	.	.	.	.	.	.	.	.	.	.	.
chr1:155236277	G	A	GBA1	0	0	2.08E-06	.	.	.	.	.	.	.	.	.	.	.	.	.	.	.	.	.
chr1:16986208	C	T	ATP13A2	0	0	0	.	.	.	.	.	.	.	.	.	.	.	.	.	.	.	.	.
chr12:40274666	A	T	LRRK2	0	0	0	0.0014	0.0051	0.0017	0.0011	0.0014	0.0051	.	0	0	0	0	0	0.0009	0.0051	0.0065	.	0.007
chr15:61910215	G	A	VPS13C	0	0	9.13E-06	.	.	.	.	.	.	.	.	.	.	.	.	.	.	.	.	.
chr3:132502298	C	T	DNAJC13	0	0	7.88E-05	0.0042	0.0144	0.004	0.0045	0.0042	0.0144	.	0.0047	0	6.54E-05	0	0	0.0009	0.014	0.0149	.	0.0149
chr1:155238570	C	G	GBA1	1.00E-04	0.00018625	8.80E-05	6.39E-05	0.0001	5.74E-05	7.21E-05	6.37E-05	0	.	0	0	0.0001	0	0	0	0.0002	7.36E-05	.	0.0002
chr3:132528231	G	A	DNAJC13	1.00E-04	0	5.20E-05	0.0001	0.0002	0.0001	0.0001	0.0001	0	.	0	0	0.0002	0.0003	0	0	0.0003	0.0002	.	0.0005
chr15:62010558	G	C	VPS13C	0	0	2.63E-05	0.0008	0.0029	0.0009	0.0007	0.0008	0.0029	.	0	0	0	0	0	0	0.0029	0.0027	.	0.0035
chr12:40263806	A	G	LRRK2	2.00E-04	0.00025961	0.00018031	6.37E-05	.	0.0001	0	6.37E-05	0	.	0	0	0	0	0.0069	0	.	.	.	.
chr1:155236409	C	G	GBA1	0	8.95E-05	2.49E-06	.	.	.	.	.	.	.	.	.	.	.	.	.	.	.	.	.
chr1:155235708	G	C	GBA1	0	0.00071848	5.18E-05	.	.	.	.	.	.	.	.	.	.	.	.	.	.	.	.	.
chr3:132496652	G	A	DNAJC13	0	0	3.99E-05	0.0002	0.0026	0.0003	7.18E-05	0.0002	0.0002	.	0	0.0026	6.48E-05	0	0	0	0.0026	0.0026	.	0.0022
chr1:155240048	C	T	GBA1	0	0	1.52E-05	.	.	.	.	.	.	.	.	.	.	.	.	.	.	.	.	.
chr12:40240594	G	C	LRRK2	1.00E-04	0.00027009	9.30E-05	6.37E-05	0.0001	0.0001	0	6.37E-05	0	.	0	0	0.0001	0	0	0	0.0002	7.35E-05	.	.
chr15:61890400	C	T	VPS13C	0.0002	0.00010803	0.00012313	3.19E-05	0.0001	0	7.19E-05	3.18E-05	0.0001	.	0	0	0	0	0	0	0.0001	0.0003	.	.
chr15:89319234	G	A	POLG	0.0002	0.00010803	0.00019702	9.56E-05	0.0012	0.0002	0	9.55E-05	0	.	0.0012	0	6.49E-05	0	0.0034	0	0.0012	7.35E-05	.	.
chr15:89319053	C	G	POLG	1.00E-04	0.0001342	0.00011101	.	.	.	.	.	.	.	.	.	.	.	.	.	.	.	.	.
chr3:184325887	A	G	EIF4G1	0	0	2.96E-06	.	.	.	.	.	.	.	.	.	.	.	.	.	.	.	.	.
chr5:176621218	G	T	SNCB	0.0005	0.00043122	0.00031512	0.0004	0.0012	0.0003	0.0004	0.0004	0	.	0.0012	0	0.0007	0	0	0	0.0012	0.0008	.	0.0009
chr12:40293624	G	C	LRRK2	1.00E-04	0.00012921	8.87E-05	6.39E-05	0.0001	0	0.0001	6.37E-05	0	.	0	0	0.0001	0	0	0	0.0002	7.36E-05	.	0.0004
chr2:74530208	C	T	HTRA2	0	0	0	0.0011	0.0038	0.0014	0.0007	0.0011	0.0038	.	0	0	0	0	0	0.0009	0.0039	0.0044	.	0.0039
chr15:61916007	G	C	VPS13C	0.0002	0.00010803	0.00029552	.	.	.	.	.	.	.	.	.	.	.	.	.	.	.	.	.
chr22:32479135	T	G	FBXO7	2.00E-04	0.00075618	0.00056643	0.0002	0.0012	0	0.0004	0.0002	0	.	0.0012	0	0.0002	0	0	0.0018	0.0012	0.0018	.	0.0041
chr15:89325562	G	A	POLG	0	0	0	0.0016	0.0055	0.0017	0.0016	0.0016	0.0055	.	0.0012	0	0.0001	0	0	0	0.0056	0.0044	.	0.0058
chr6:161785820	G	A	PRKN	0.0027	0.00404714	0.00276527	0.0023	0.0035	0.0024	0.0021	0.0023	0.0006	.	0.0035	0	0.0033	0.002	0	0.0046	0.0027	0.0036	.	0.0013
chr22:38113561	G	A	PLA2G6	0	4.47E-05	9.15E-06	.	.	.	.	.	.	.	.	.	.	.	.	.	.	.	.	.
chr12:40363526	G	A	LRRK2	0	2.92E-05	0	0.0014	0.029	0.0019	0.0009	0.0015	0	.	0	0.029	0	0	0	0	0.0291	0.029	.	0.0275
chr5:122450987	G	A	SNCAIP	0.0002	0.00021605	0.00012313	9.56E-05	0.0012	0.0001	7.18E-05	9.55E-05	0.0002	.	0.0012	0	0	0	0	0	0.0012	.	.	.
chr20:5100832	G	A	TMEM230	0.0021	0.00399914	0.00401567	0.0029	0.0031	0.003	0.0027	0.0029	0.0006	.	0	0	0.0031	0.0092	0	0.0055	0.0033	0.0034	.	0.0049
chr22:38128349	G	A	PLA2G6	0.0003	0.00021605	0.00078808	6.37E-05	0.0001	5.73E-05	7.19E-05	6.37E-05	0	.	0	0	0.0001	0	0	0	9.03E-05	0.0001	.	.
chr22:38135029	G	A	PLA2G6	1.00E-04	8.95E-05	7.28E-05	3.19E-05	0.0001	5.74E-05	0	3.18E-05	0.0001	.	0	0	0	0	0	0	0.0001	.	.	.
chr15:61962754	T	A	VPS13C	0	0	6.50E-06	.	.	.	.	.	.	.	.	.	.	.	.	.	.	.	.	.
chr15:89323423	A	G	POLG	0	0	5.40E-06	0.0002	0.0008	0.0003	0.0001	0.0002	0.0008	.	0	0	0	0	0	0	0.0008	0.0003	.	0.0004
chr1:7961737	T	A	PARK7	1.00E-04	NA	NA	3.20E-05	6.50E-05	5.75E-05	0	3.18E-05	0	.	0	0	6.50E-05	0	0	0	.	7.37E-05	.	.
chr6:162443429	C	T	PRKN	0	0	0	0.0005	0.0024	0.0005	0.0006	0.0005	0.0017	.	0.0024	0	0	0	0	0	0.0025	0.0036	.	0.0019
chr1:20644662	G	A	PINK1	1.00E-04	0.00011375	5.49E-05	.	.	.	.	.	.	.	.	.	.	.	.	.	.	.	.	.
chr3:184327401	G	A	EIF4G1	0.0003	0.0005197	0.00017043	0.0002	0.0003	0.0002	0.0001	0.0002	0.0003	.	0	0	0.0001	0	0	0	0.0004	0.0003	.	0.0004
chr1:7985019	G	A	PARK7	0.0006	0.00129632	0.00076353	0.0008	0.0013	0.001	0.0005	0.0008	0.0001	.	0.0012	0	0.0013	0.0003	0	0.0009	0.0013	0.0018	.	0.0016
chr3:132484650	C	T	DNAJC13	0.0006	0.00118829	0.00142843	0.0004	0.0007	0.0005	0.0003	0.0004	0.0001	.	0	0	0.0007	0	0	0	0.0008	0.0006	.	0.0005
chr12:40305946	T	A	LRRK2	0	0.00011294	0.00010764	.	.	.	.	.	.	.	.	.	.	.	.	.	.	.	.	.

chr6:161350125	T	G	PRKN	1.00E-04	0.00024683	0.00015807	0.0002	0.0026	0.0002	0.0001	0.0002	0	.	0	0.0026	0.0001	0	0	0	0.0026	0.0026	.	0.0022
chr1:155237438	C	T	GBA1	0.0002	0.00077581	0.00015003	0.0003	0.0005	0.0004	0.0001	0.0003	0.0002	.	0	0	0.0005	0	0	0	0.0003	0.0006	.	0.0004
chr12:40302866	A	G	LRRK2	0	0	1.52E-06	3.19E-05	0.0012	5.73E-05	0	3.18E-05	0	.	0.0012	0	0	0	0	0	0.0012	.	.	.
chr1:65385756	G	A	DNAJC6	0	0	5.26E-05	6.37E-05	0.0001	5.73E-05	7.18E-05	6.37E-05	0.0001	.	0	0	0	0	0	0.0009	0.0001	.	.	.
chr15:61936649	C	G	VPS13C	0	0.00012811	2.63E-05	6.37E-05	0.0012	0.0001	0	6.37E-05	0	.	0.0012	0	6.48E-05	0	0	0	0.0012	7.34E-05	.	.
chr1:20649217	C	T	PINK1	1.00E-04	7.20E-05	5.45E-05	3.18E-05	0.0001	5.72E-05	0	3.18E-05	0.0001	.	0	0	0	0	0	0	0.0001	.	.	.
chr15:89320857	G	A	POLG	0	0	1.45E-05	0.0006	0.0115	0.0007	0.0004	0.0006	0	.	0	0.0115	0	0	0	0	0.0112	0.0115	.	0.0109
chr22:38120867	T	G	PLA2G6	0	8.77E-05	1.08E-05	.	.	.	.	.	.	.	.	.	.	.	.	.	.	.	.	.
chr3:184327230	G	A	EIF4G1	1.00E-04	0	4.93E-05	6.37E-05	0.0001	5.72E-05	7.18E-05	6.37E-05	0.0001	.	0	0	6.48E-05	0	0	0	0.0001	0.0003	.	0.0004
chr1:20648601	G	A	PINK1	0	0	4.10E-05	.	.	.	.	.	.	.	.	.	.	.	.	.	.	.	.	.
chr6:162443383	C	T	PRKN	0.0002	4.20E-05	4.48E-05	6.37E-05	0.0012	0	0.0001	6.37E-05	0	.	0.0012	0	6.48E-05	0	0	0	0.0012	0.0018	.	0.0041
chr15:61984004	G	A	VPS13C	0	0	2.63E-05	3.18E-05	0.0001	5.72E-05	0	3.18E-05	0.0001	.	0	0	0	0	0	0	0.0001	0.0003	.	.
chr6:161973335	C	T	PRKN	1.00E-04	3.70E-05	5.67E-05	0.0002	0.0003	0.0001	0.0002	0.0002	0.0001	.	0	0	0.0003	0	0	0	0.0003	0.0002	.	0.0004
chr1:20639911	C	T	PINK1	0	0	4.16E-07	0.0002	0.0007	0.0001	0.0003	0.0002	0.0007	.	0	0	0	0	0	0	0.0007	0.0006	.	.
chr12:40235634	T	C	LRRK2	0.0024	0.00311163	0.00242517	0.0013	0.002	0.0014	0.0013	0.0013	0.0009	.	0	0	0.002	0	0	0.0028	0.0018	0.0021	.	0.0018
chr6:161360169	G	A	PRKN	0.0016	0.00334918	0.00302986	0.0013	0.0024	0.0015	0.0011	0.0014	0.0011	.	0.0024	0	0.0013	0.002	0	0.0028	0.0025	0.0018	.	0.0016
chr1:20645675	G	A	PINK1	0	0	4.03E-05	0.0006	0.0022	0.0005	0.0007	0.0006	0.0022	.	0	0	0	0	0	0	0.0022	0.0027	.	0.0031
chr14:22873373	C	T	LRP10	1.00E-04	0.00052759	0.00022566	0.0006	0.0096	0.0005	0.0007	0.0006	0	.	0	0.0096	0.0001	0	0	0.0018	0.0099	0.0096	.	0.0109
chr15:61880871	G	A	VPS13C	0.0002	0.00032408	0.00034478	6.38E-05	0.0001	5.73E-05	7.19E-05	6.37E-05	0	.	0	0	0.0001	0	0	0	0.0002	0.0001	.	0.0002
chr15:50586433	G	A	TRPM7	0.1054	0.15580039	0.10996237	0.0723	0.1049	0.0743	0.0699	0.0724	0.0216	.	0.0743	0.0577	0.1049	0.0581	0.0828	0.0795	0.0983	0.1055	.	0.0848
chr22:32493191	G	A	FBXO7	1.00E-04	0.00025628	0.00028909	.	.	.	.	.	.	.	.	.	.	.	.	.	.	.	.	.
chr3:132494190	A	G	DNAJC13	0.0064	0.01162105	0.01174986	0.0027	0.0047	0.0028	0.0026	0.0027	0.0006	.	0.0024	0	0.0047	0.0009	0	0.0018	0.0033	0.0049	.	0.0041
chr15:61911844	G	A	VPS13C	0.0033	0.00658961	0.00588597	0.002	0.0035	0.0023	0.0017	0.002	0.0006	.	0.0035	0	0.0032	0.0009	0	0.0018	0.0037	0.0054	.	0.0041
chr12:40304004	A	G	LRRK2	0	0	4.31E-06	.	.	.	.	.	.	.	.	.	.	.	.	.	.	.	.	.
chr3:184321516	A	G	EIF4G1	3.00E-04	0.00021046	0.00031599	0.0161	0.0569	0.0163	0.016	0.0162	0.0569	.	0.0071	0	0.0001	0	0	0.0037	0.057	0.057	.	0.0582
chr15:62008659	G	A	VPS13C	0	0	7.59E-06	.	.	.	.	.	.	.	.	.	.	.	.	.	.	.	.	.
chr3:132478139	G	A	DNAJC13	0.0028	0.00767236	0.0065023	0.0016	0.0083	0.0014	0.0019	0.0016	0.0001	.	0.0083	0	0.0025	0.0009	0	0	0.0086	0.0054	.	0.0041
chr3:132502295	C	T	DNAJC13	0.0289	0.0383855	0.02879244	0.0161	0.0262	0.0164	0.0157	0.0161	0.0047	.	0.019	0	0.0262	0.0065	0.0278	0.0122	0.0257	0.0264	.	0.0234
chr6:162443314	A	T	PRKN	0	9.78E-05	7.46E-05	6.37E-05	0.0001	0.0001	0	6.37E-05	0	.	0	0	0.0001	0	0	0	0.0002	0.0001	.	0.0002
chr15:61917535	C	T	VPS13C	0	0	0	0.0009	0.0173	0.001	0.0007	0.0009	0	.	0	0.0173	0	0	0	0	0.0158	0.0173	.	0.0197
chr15:61854888	C	A	VPS13C	0.0014	0.00334882	0.00344785	0.0009	0.0016	0.0009	0.0009	0.0009	0.0002	.	0.0012	0	0.0016	0	0	0	0.0016	0.0018	.	0.0041
chr15:61915655	T	C	VPS13C	7.00E-04	0.00137836	0.00078247	0.027	0.0952	0.0277	0.0262	0.0272	0.0952	.	0.0047	0	0.0004	0.0003	0.0034	0.0074	0.0953	0.0966	.	0.0983
chr12:40240543	C	T	LRRK2	0.0002	3.33E-05	9.78E-05	0.0002	0.0019	0.0003	7.18E-05	0.0002	0.0001	.	0	0.0019	0.0001	0	0	0	0.002	0.0019	.	0.0004
chr15:89319031	A	G	POLG	0	0	7.18E-06	.	.	.	.	.	.	.	.	.	.	.	.	.	.	.	.	.
chr1:155238629	C	T	GBA1	0	0	1.62E-05	.	.	.	.	.	.	.	.	.	.	.	.	.	.	.	.	.
chr1:16988161	T	A	ATP13A2	0.0018	0.00442956	0.00344777	0.0005	0.0009	0.0004	0.0006	0.0005	0.0002	.	0	0	0.0009	0	0	0	0.0011	0.001	.	0.0007
chr12:40367050	A	G	LRRK2	0	0	1.44E-06	.	.	.	.	.	.	.	.	.	.	.	.	.	.	.	.	.
chr15:61984885	A	T	VPS13C	3.00E-04	0.00086421	0.00066493	0.0002	0.0004	0.0001	0.0004	0.0002	0.0001	.	0	0	0.0004	0	0	0	0.0004	0.0004	.	0.0004
chr1:16992042	A	G	ATP13A2	0	0	0	0.0049	0.0176	0.0049	0.0048	0.0049	0.0176	.	0	0	0	0	0	0	0.0177	0.018	.	0.0175
chr16:46671766	T	C	VPS35	0	0	4.22E-07	.	.	.	.	.	.	.	.	.	.	.	.	.	.	.	.	.
chr3:184327622	T	C	EIF4G1	0.0239	0.03440358	0.02430117	0.0397	0.0844	0.0394	0.04	0.0397	0.0844	.	0.0142	0	0.0226	0.0345	0.0034	0.0257	0.0846	0.081	.	0.0863
chr2:74531688	G	A	HTRA2	0	0	9.56E-06	.	.	.	.	.	.	.	.	.	.	.	.	.	.	.	.	.
chr2:232844373	C	G	GIGYF2	0.002	0.00259263	0.00426056	0.0012	0.0021	0.001	0.0014	0.0012	0.0003	.	0	0	0.0021	0.0006	0	0.0009	0.0018	0.0022	.	0.0018
chr12:40298346	G	A	LRRK2	0	0	7.71E-06	3.19E-05	6.49E-05	0	7.19E-05	3.18E-05	0	.	0	0	6.49E-05	0	0	0	9.04E-05	.	.	.
chr15:89320917	C	T	POLG	0	0	4.17E-06	3.19E-05	0.0006	0	7.18E-05	3.18E-05	0	.	0	0.0006	0	0	0	0	0.0007	0.0006	.	.
chr15:89321842	T	C	POLG	0.0098	0.01414766	0.01009455	0.0092	0.0134	0.0084	0.0102	0.0092	0.0014	.	0	0	0.0134	0.017	0	0.0111	0.0151	0.0136	.	0.0184
chr20:5069330	C	T	TMEM230	1.00E-04	0	0	0.0018	0.0063	0.0021	0.0015	0.0018	0.0063	.	0	0	0.0001	0	0	0.0009	0.0064	0.0065	.	0.0054
chr22:38169255	C	T	PLA2G6	0.0002	0.00068918	0.00039124	0.0134	0.0477	0.0138	0.0129	0.0134	0.0477	.	0.0012	0	6.49E-05	0	0	0.0018	0.0479	0.0443	.	0.0471
chr1:155238251	G	T	GBA1	0	0	1.52E-06	.	.	.	.	.	.	.	.	.	.	.	.	.	.	.	.	.
chr15:89333427	G	A	POLG	1.00E-04	0	0	0.0002	0.0008	0.0001	0.0004	0.0002	0.0008	.	0	0	0	0	0	0	0.0008	0.0009	.	0.0008
chr1:155239716	C	G	GBA1	0	5.29E-05	1.07E-05	.	.	.	.	.	.	.	.	.	.	.	.	.	.	.	.	.
chr14:22877108	G	A	LRP10	1.00E-04	0.00075618	0.00024628	0.0004	0.0035	0.0002	0.0006	0.0004	0.0005	.	0.0035	0	0.0003	0	0	0.0009	0.0037	0.0036	.	0.0041
chr12:40320103	A	G	LRRK2	2.00E-04	0.00021608	0.00051723	3.19E-05	6.48E-05	0	7.19E-05	3.18E-05	0	.	0	0	6.48E-05	0	0	0	9.02E-05	.	.	.
chr15:62023837	G	A	VPS13C	1.00E-04	0.00012811	0.00013141	0.0004	0.009	0.0006	0.0002	0.0004	0	.	0	0.009	0	0	0	0	0.0086	0.009	.	0.0077
chr1:155235777	T	A	GBA1	0	0.00025954	3.91E-05	.	.	.	.	.	.	.	.	.	.	.	.	.	.	.	.	.
chr22:32475378	G	A	FBXO7	0.0997	0.19872142	0.19886827	0.0821	0.1038	0.0825	0.0815	0.0822	0.0367	.	0.1038	0.0418	0.1036	0.1094	0.0862	0.0919	0.1049	0.1065	.	0.1027
chr22:38126417	G	A	PLA2G6	0.0002	0.00021005	0.0003373	0.0002	0.0003	0.0001	0.0003	0.0002	0.0002	.	0	0	0.0003	0	0	0	0.0004	0.0003	.	0.0004
chr15:61927138	G	A	VPS13C	0.0132	0.01869538	0.01324778	0.0079	0.0142	0.0088	0.0068	0.0079	0.0022	.	0.0142	0	0.0118	0.0078	0.0103	0.0037	0.0149	0.0163	.	0.0244
chr12:40310561	G	A	LRRK2	0	0	3.34E-05	3.20E-05	6.51E-05	0	7.21E-05	3.18E-05	0	.	0	0	6.51E-05	0	0	0	9.06E-05	.	.	.
chr15:89325639	G	A	POLG	0.0025	0.00330468	0.00252588	0.0015	0.0023	0.002	0.0009	0.0015	0.0005	.	0	0	0.0023	0.0012	0.0034	0.0028	0.0019	0.0022	.	0.0012
chr1:16986091	G	A	ATP13A2	0.0054	0.01091656	0.01338328	0.0022	0.0047	0.0021	0.0022	0.0022	0.001	.	0.0047	0	0.0032	0.0006	0.0034	0.0018	0.0049	0.0054	.	0.0081
chr1:16986246	G	A	ATP13A2	1.00E-04	0.00021605	0.00012313	9.59E-05	0.0001	5.74E-05	0.0001	9.55E-05	0	.	0	0	0.0001	0.0003	0	0	0.0002	7.37E-05	.	.
chr6:162262627	G	A	PRKN	0	0	2.59E-05	.	.	.	.	.	.	.	.	.	.	.	.	.	.	.	.	.
chr1:155237370	G	A	GBA1	0	0	1.22E-05	.	.	.	.	.	.	.	.	.	.	.	.	.	.	.	.	.
chr1:155235780	G	A	GBA1	0	0.00011962	1.44E-06	.	.	.	.	.	.	.	.	.	.	.	.	.	.	.	.	.
chr2:232790836	G	C	GIGYF2	0.0002	0.00032408	0.0002709	6.37E-05	0.0001	5.72E-05	7.18E-05	6.37E-05	0.0001	.	0	0	6.48E-05	0	0	0	0.0001	7.34E-05	.	.
chr15:89321792	C	T	POLG	0.0004	0.00052962	0.00022516	0.0004	0.0006	0.0004	0.0003	0.0004	0.0001	.	0	0	0.0006	0	0	0	0.0004	0.0007	.	.
chr1:155236246	G	A	GBA1	0.007	0.01553736	0.00883603	0.0076	0.0115	0.007	0.0084	0.0076	0.0006	.	0.0047	0	0.0115	0.0095	0.0034	0.0165	0.012	0.0117	.	0.0143
chr1:20637888	C	T	PINK1	0	0	5.73E-05	.	.	.	.	.	.	.	.	.	.	.	.	.	.	.	.	.
chr12:40364843	G	A	LRRK2	0	0	6.82E-06	.	.	.	.	.	.	.	.	.	.	.	.	.	.	.	.	.
chr3:132507256	A	G	DNAJC13	0.0611	0.11665404	0.10906127	0.0459	0.0645	0.0447	0.0474	0.046	0.0105	.	0.0342	0	0.0645	0.0676	0.0828	0.0617	0.0656	0.065	.	0.0696
chr15:61945803	G	A	VPS13C	0.0029	0.0054019	0.00632896	0.0015	0.0035	0.0015	0.0015	0.0016	0.0007	.	0.0035	0	0.0021	0.0012	0	0.0028	0.0037	0.0036	.	0.0041
chr22:38115667	G	A	PLA2G6	0	0	8.92E-06	3.19E-05	0.0001	5.73E-05	0	3.18E-05	0.0001	.	0	0	0	0	0	0	0.0001	.	.	0.0004
chr12:40251273	G	A	LRRK2	0.001	0.00082283	0.00075404	0.0049	0.0039	0.0039	0.0062	0.005	0.0002	.	0	0	0.0039	0.0245	0.0034	0.0065	0.0052	0.0036	.	0.0078
chr15:61954471	T	G	VPS13C	0.001	0.00194447	0.00184706	0.001	0.0008	0.001	0.001	0.001	0.0002	.	0	0	0.0008	0.0037	0	0.0028	0.0011	0.001	.	0.0011
chr15:61880845	G	A	VPS13C	0	0.00012816	2.63E-05	3.20E-05	0.0001	0	7.22E-05	3.18E-05	0.0001	.	0	0	0	0	0	0	0.0001	0.0003	.	0.0004
chrX:155260942	G	T	RAB39B	0	0	2.12E-06	.	.	.	.	.	.	.	.	.	.	.	.	.	.	.	.	.
chr1:17005754	G	A	ATP13A2	0.0002	0.00043211	0.00039402	3.19E-05	6.49E-05	0	7.18E-05	3.18E-05	0	.	0	0	6.49E-05	0	0	0	.	7.35E-05	.	.
chr1:20649109	C	T	PINK1	1.00E-04	0	0.00014465	3.19E-05	6.48E-05	0	7.18E-05	3.18E-05	0	.	0	0	6.48E-05	0	0	0	9.02E-05	7.34E-05	.	.
chr3:184321987	C	A	EIF4G1	0.0014	NA	NA	.	.	.	.	.	.	.	.	.	.	.	.	.	.	.	.	.
chr16:46662405	A	C	VPS35	0	0	2.63E-05	.	.	.	.	.	.	.	.	.	.	.	.	.	.	.	.	.
chr15:61963904	C	T	VPS13C	0.0004	0.0008643	0.00098515	6.38E-05	0.0001	0	0.0001	6.37E-05	0.0001	.	0	0	6.50E-05	0	0	0	0.0001	0.0003	.	0.0004
chr15:61920268	C	T	VPS13C	0.0021	0.00432105	0.00490075	0.0012	0.002	0.0011	0.0012	0.0012	0.0005	.	0.0012	0	0.002	0	0	0.0009	0.0016	0.0021	.	0.0004
chr15:62023783	T	C	VPS13C	7.00E-04	0.00108038	0.00174855	0.0013	0.0008	0.0014	0.0013	0.0013	0	.	0	0	0.0008	0.0066	0	0.0055	0.0005	0.001	.	0.0009
chr15:89333267	G	A	POLG	0.0005	0.00070831	0.00101863	.	.	.	.	.	.	.	.	.	.	.	.	.	.	.	.	.
chr3:132511169	G	C	DNAJC13	0.0016	0.00453907	0.00367004	0.0012	0.0016	0.0013	0.0012	0.0012	0.0003	.	0	0	0.0016	0.0032	0	0.0009	0.0018	0.0015	.	0.0015
chr6:161350208	C	T	PRKN	0.0002	0.00049586	0.00013126	0.0001	0.0002	5.72E-05	0.0002	0.0001	0.0001	.	0	0	0.0002	0	0	0	0.0001	0.0002	.	.
chr15:89318587	G	A	POLG	0.0002	0.00014068	0.00015705	0.0002	0.0004	0.0002	0.0001	0.0002	0	.	0	0	0.0004	0	0	0	0.0005	0.0004	.	0.0005
chr12:40274671	T	C	LRRK2	0	0	2.88E-06	.	.	.	.	.	.	.	.	.	.	.	.	.	.	.	.	.

chr15:61984953	G	A	VPS13C	1.00E-04	0	0.00012314	0.0057	0.0201	0.0054	0.0061	0.0057	0.0201	.	0.0012	0	0.0001	0	0	0.0009	0.0199	0.0207	.	0.0225
chr1:155239948	G	A	GBA1	0	0	1.45E-05	.	.	.	.	.	.	.	.	.	.	.	.	.	.	.	.	.
chr1:65401812	C	T	DNAJC6	0	0.00012811	2.63E-05	0.0009	0.0031	0.0013	0.0004	0.0009	0.0031	.	0	0	6.48E-05	0	0	0	0.0032	0.0035	.	0.0043
chr15:89324193	C	T	POLG	0.0003	0.00043211	0.00036939	0.0015	0.0436	0.0016	0.0014	0.0015	0.0002	.	0.0436	0	0.0003	0	0.0034	0.0028	0.0432	0.0379	.	0.0203
chr15:61969413	T	C	VPS13C	0.0049	0.00725422	0.00886551	0.0055	0.009	0.005	0.0063	0.0055	0.0013	.	0	0	0.009	0.0058	0	0.0046	0.0106	0.009	.	0.0142
chr1:155235814	C	T	GBA1	0	0	4.55E-06	.	.	.	.	.	.	.	.	.	.	.	.	.	.	.	.	.
chr15:89327004	T	G	POLG	2.00E-04	0.00050429	0.00022605	3.19E-05	0.0001	0	7.18E-05	3.18E-05	0.0001	.	0	0	0	0	0	0	0.0001	.	.	.
chr12:40304000	G	A	LRRK2	1.00E-04	0.00043211	0.00019701	0.0002	0.0003	0.0002	0.0001	0.0002	0.0001	.	0	0	0.0003	0	0	0	0.0004	0.0003	.	0.0002
chr22:38126371	G	A	PLA2G6	0.0003	0.00075635	0.00076366	0.0002	0.0024	0.0002	0.0001	0.0002	0.0001	.	0.0024	0	0.0001	0	0	0	0.0025	0.0036	.	0.0004
chr21:32688348	C	T	SYNJ1	0	0	4.93E-05	9.55E-05	0.0013	0.0002	0	9.55E-05	0	.	0	0.0013	6.48E-05	0	0	0	0.0013	0.0013	.	0.0011
chr12:40364975	C	A	LRRK2	0	0	6.00E-05	.	.	.	.	.	.	.	.	.	.	.	.	.	.	.	.	.
chr1:155239933	C	T	GBA1	0	0	4.55E-06	.	.	.	.	.	.	.	.	.	.	.	.	.	.	.	.	.
chr12:40299255	T	C	LRRK2	1.00E-04	0	0.00022549	.	.	.	.	.	.	.	.	.	.	.	.	.	.	.	.	.
chr15:61961592	T	C	VPS13C	0.0784	0.09875963	0.07938395	0.0596	0.0829	0.059	0.0603	0.0598	0.0323	.	0.059	0.0372	0.0829	0.036	0.0172	0.0671	0.0844	0.0811	.	0.0896
chr22:38116200	G	A	PLA2G6	0	0	1.27E-06	.	.	.	.	.	.	.	.	.	.	.	.	.	.	.	.	.
chr12:40251361	A	G	LRRK2	0	0	1.61E-06	.	.	.	.	.	.	.	.	.	.	.	.	.	.	.	.	.
chr12:40299212	G	A	LRRK2	1.00E-04	0.00018553	0.00022219	3.19E-05	6.49E-05	5.73E-05	0	3.18E-05	0	.	0	0	6.49E-05	0	0	0	9.03E-05	7.35E-05	.	.
chr3:132538225	G	A	DNAJC13	0.0035	0.00659174	0.00793103	0.0047	0.0531	0.0042	0.0053	0.0047	0.0022	.	0.0531	0.0006	0.0036	0.0066	0	0.0046	0.0556	0.0505	.	0.0691
chr15:89333364	A	G	POLG	4.00E-04	0.00129632	0.0005664	0.0002	0.0004	0.0002	0.0002	0.0002	0	.	0	0	0.0004	0	0	0	0.0005	0.0004	.	0.0005
chr21:32688306	C	T	SYNJ1	0	0	0	.	.	.	.	.	.	.	.	.	.	.	.	.	.	.	.	.
chr3:132523636	G	C	DNAJC13	0.0011	0.00140434	0.00214254	0.0004	0.0008	0.0003	0.0006	0.0004	0.0002	.	0	0	0.0008	0	0	0	0.001	0.0005	.	0.0007
chr15:61920099	C	T	VPS13C	0.0072	0.01242303	0.01268848	0.005	0.006	0.0055	0.0045	0.005	0.0014	.	0.0048	0.0006	0.006	0.0087	0.0103	0.0138	0.005	0.0063	.	0.0124
chr3:184331762	C	T	EIF4G1	0.0002	0.00043211	0.00044327	0.0002	0.0024	0.0002	7.18E-05	0.0002	0.0001	.	0.0024	0	0.0001	0	0	0	0.0025	0.0036	.	0.0041
chr2:232839964	G	A	GIGYF2	0.0007	0.00172842	0.00162542	0.0006	0.0012	0.0006	0.0006	0.0006	0.0002	.	0.0012	0	0.001	0	0	0.0009	0.0012	0.001	.	0.0011
chr3:132492416	G	A	DNAJC13	0	0	1.16E-05	.	.	.	.	.	.	.	.	.	.	.	.	.	.	.	.	.
chr6:161350211	C	T	PRKN	0	4.47E-05	7.98E-05	.	.	.	.	.	.	.	.	.	.	.	.	.	.	.	.	.
chr1:16986097	G	A	ATP13A2	0.0586	0.07807375	0.05874141	0.0466	0.0628	0.043	0.0513	0.0467	0.0095	.	0.0248	0	0.0628	0.0927	0.0207	0.0588	0.0653	0.0625	.	0.0699
chr2:232791432	C	T	GIGYF2	1.00E-04	0.00010303	0.00010066	0.0126	0.0446	0.0137	0.0111	0.0125	0.0446	.	0.0024	0	6.49E-05	0	0	0.0028	0.0441	0.0505	.	0.0514
chr1:7984971	G	A	PARK7	0	0	1.99E-06	.	.	.	.	.	.	.	.	.	.	.	.	.	.	.	.	.
chr14:22877045	C	T	LRP10	0	0	0.00030726	6.37E-05	0.0012	0	0.0001	6.37E-05	0	.	0.0012	0	6.49E-05	0	0	0	0.0012	0.0018	.	.
chr3:184321497	C	T	EIF4G1	0	0.00012811	5.26E-05	0.0007	0.0024	0.0007	0.0006	0.0007	0.0024	.	0	0	0	0	0	0	0.0025	0.0015	.	0.0023
chr1:65386820	G	A	DNAJC6	1.00E-04	0.00038432	0.00021028	6.37E-05	0.0013	0.0001	0	6.37E-05	0	.	0	0.0013	0	0	0	0	0.0013	0.0013	.	0.0011
chr15:61907289	T	C	VPS13C	0	0	0	0.0014	0.0049	0.0014	0.0014	0.0014	0.0049	.	0	0	6.48E-05	0	0	0.0009	0.0049	0.0041	.	0.0039
chr15:61890334	G	A	VPS13C	0	0	2.63E-05	0.0001	0.0019	0.0002	0	0.0001	0	.	0	0.0019	6.49E-05	0	0	0	0.002	0.0019	.	0.0022
chr1:155241085	C	T	GBA1	0	0	4.22E-07	.	.	.	.	.	.	.	.	.	.	.	.	.	.	.	.	.
chr22:38135061	A	C	PLA2G6	0	0	4.22E-07	.	.	.	.	.	.	.	.	.	.	.	.	.	.	.	.	.
chr14:22876816	G	A	LRP10	0.0008	0.00021605	0.00019701	0.0026	0.0341	0.0028	0.0024	0.0026	0	.	0	0.0341	0.0008	0.0049	0	0	0.0336	0.0341	.	0.0274
chr14:22876765	G	T	LRP10	1.00E-04	0.00012811	2.63E-05	0.0101	0.0357	0.0097	0.0106	0.0102	0.0357	.	0.0012	0	0.0002	0	0	0.0028	0.0362	0.0371	.	0.0383
chr6:161350187	G	A	PRKN	0.0025	0.00370466	0.00243288	0.0014	0.0024	0.0015	0.0014	0.0014	0.0007	.	0.0012	0	0.0024	0.0003	0	0	0.002	0.0024	.	0.0007
chr22:38115588	T	G	PLA2G6	0	0	5.51E-06	.	.	.	.	.	.	.	.	.	.	.	.	.	.	.	.	.
chr22:38116119	C	A	PLA2G6	0.0002	0	0.00012313	9.56E-05	0.0001	0.0001	7.18E-05	9.55E-05	0	.	0	0	0.0001	0.0003	0	0	9.02E-05	0.0001	.	0.0002
chr1:155238596	C	A	GBA1	0	0	7.22E-06	.	.	.	.	.	.	.	.	.	.	.	.	.	.	.	.	.
chr22:38143219	C	G	PLA2G6	2.00E-04	0.00010803	0.00036939	0.0044	0.0154	0.0039	0.0049	0.0044	0.0154	.	0.0012	0	0.0001	0	0	0	0.0154	0.0162	.	0.0152
chr1:155236276	C	T	GBA1	0	0.00011957	8.63E-06	.	.	.	.	.	.	.	.	.	.	.	.	.	.	.	.	.
chr15:61856408	G	A	VPS13C	0.0006	0.00156678	0.00059352	0.0002	0.0012	0.0002	0.0001	0.0002	0.0002	.	0.0012	0	0.0002	0	0	0	0.0012	0.0018	.	0.0041
chr3:132492497	A	G	DNAJC13	5.00E-04	0.00057833	0.00040026	0.0002	0.0003	0.0002	7.19E-05	0.0002	0	.	0	0	0.0003	0	0	0	0.0003	0.0003	.	.
chr22:38126374	C	T	PLA2G6	0.0005	0.00075618	0.00064039	0.0003	0.0004	0.0003	0.0002	0.0003	0	.	0	0	0.0004	0	0.0034	0.0009	0.0005	0.0004	.	0.0004
chr15:61983969	G	A	VPS13C	0	0	4.03E-05	.	.	.	.	.	.	.	.	.	.	.	.	.	.	.	.	.
chr20:5109429	A	G	TMEM230	0.0014	0.00194447	0.00327538	0.0009	0.0047	0.0007	0.001	0.0009	0	.	0.0047	0	0.0014	0	0.0034	0.0009	0.0049	0.0054	.	0.0081
chr22:38132922	C	T	PLA2G6	0	0	4.37E-05	3.19E-05	6.48E-05	0	7.19E-05	3.18E-05	0	.	0	0	6.48E-05	0	0	0	.	7.34E-05	.	.
chr6:161973401	C	T	PRKN	0	0	1.10E-05	.	.	.	.	.	.	.	.	.	.	.	.	.	.	.	.	.
chr1:155237576	A	T	GBA1	0	6.19E-05	1.64E-05	3.19E-05	6.49E-05	5.73E-05	0	3.18E-05	0	.	0	0	6.49E-05	0	0	0	9.03E-05	7.35E-05	.	.
chr15:89321242	C	A	POLG	0	0	2.76E-06	.	.	.	.	.	.	.	.	.	.	.	.	.	.	.	.	.
chr12:40356126	A	T	LRRK2	0	0.00012011	5.79E-06	.	.	.	.	.	.	.	.	.	.	.	.	.	.	.	.	.
chr3:132499168	G	A	DNAJC13	0.0002	0.00086421	0.00039404	0.0002	0.0003	0.0002	0.0003	0.0002	0	.	0	0	0.0003	0	0	0.0018	0.0003	0.0004	.	0.0002
chr3:132467277	G	A	DNAJC13	0.0002	0.00012811	0.00044678	0.0002	0.0003	0.0002	7.19E-05	0.0002	0	.	0	0	0.0003	0	0	0	0.0004	0.0004	.	0.0004
chr3:184319745	A	G	EIF4G1	0.4096	0.00875203	0.00840108	0.9976	1	0.9977	0.9976	0.9976	0.999	.	0.9976	1	0.9969	0.996	1	0.9982	1	1	.	1
chr22:38169240	T	C	PLA2G6	1.00E-04	NA	NA	0.0032	0.0115	0.0036	0.0027	0.0032	0.0115	.	0	0	0	0	0	0.0009	0.0112	0.01	.	0.0093

Supplementary Table 10: Meta-analysis variants and their counts, and MAC counts

Note: Numbers may not be 100% accurate but are estimates, because of dosage information for imputed variants in 23andMe data

MarkerName	rsID	VariantName	Ref	Alt	Genotyped_vs_I	TOTAL_n_cases	MAF_case	TOTAL_MAC_ca	23andMe_MAC_	UKB_MAC_case	AMP_MAC_case	TOTAL_n_ctrls	MAF_ctrls	TOTAL_MAC_ctr	23andMe_MAC_	UKB_MAC_ctrls	AMP_MAC_ctrls
chr1:20638104	rs74315360	PINK1_A217D	C	A	G	8361	0	0	0	NA	NA	695243	1.44E-06	1	1	NA	NA
chr22:38112571	rs772143897	PLA2G6_D737Y	C	A	I	7806	0.0002562	2	0	2	NA	38050	7.88E-05	3	0	3	NA
chr3:195867623	rs112384084	TNK2_R877H	C	T	I	23809	0.0248323	591.2330183	244.2330183	301	46	2408731	0.017155231	41322.33711	39729.33711	1516	77
chr1:155235727	rs1064651	GBA1_D448H	C	G	I	30583	0.0001464	4.47812531	1.47812531	3	NA	3092117	6.47E-05	200.1780011	198.1780011	2	NA
chr15:89318617	rs56047213	POLG_E1136K	C	T	G	22366	4.47E-05	1	0	1	NA	2406855	3.32E-06	8	8	0	NA
chr2:232791124	rs148277228	GIGYF2_D349E	T	A	I	9256	0.0007563	7	0	6	1	40607	0.000985052	40	0	38	2
chr1:155238258	rs374591570	GBA1_L213F	G	A	G	4322	0	0	0	NA	NA	616579	4.87E-06	3	3	NA	NA
chr6:161785877	rs150562946	PRKN_R256C	G	A	I	13611	0.0012661	17.23290529	2.232905294	11	4	661455	0.000584162	386.3967046	318.3967046	62	6
chr12:40322386	rs111910483	LRRK2_L1795F	G	T	I	5801	0.0004084	2.369410042	0.369410042	NA	2	623489	8.46E-05	52.73077832	52.73077832	NA	0
chr15:89333621	rs201016638	POLG_Q45R	T	C	I	1451	0.0013784	2	0	NA	2	2556	0.000391236	1	0	NA	1
chr1:65386873	rs144323705	DNAJC6_V296M	G	A	G	23811	0.000126	3	1	1	1	2407523	1.83E-05	44	41	3	0
chr1:175406252	rs150331590	TNR_C155S	A	T	G	23808	4.20E-05	1	1	0	0	2407350	0.000177373	427	422	5	0
chr6:162443371	rs148990138	PRKN_P37L	G	A	I	13607	0.0005791	7.880076801	0.880076801	6	1	661286	0.000268485	177.5451503	125.5451503	49	3
chr12:40367045	rs146428335	LRRK2_R2477Q	G	A	I	9257	0.0004321	4	0	4	0	40605	0.00039404	16	0	16	0
chr1:20637956	rs768091663	PINK1_A168P	G	C	G	14559	0	0	0	NA	NA	2368338	2.83E-05	67	67	NA	NA
chr1:16986292	rs201610681	ATP13A2_A1057	G	A	I	9256	0.0003241	3	NA	3	0	40607	0.000344768	14	NA	14	0
chr1:65392454	rs145329294	DNAJC6_C441S	T	A	I	9257	0.0012963	12	0	11	1	40606	0.001157464	47	0	41	6
chr1:16987187	rs148201608	ATP13A2_T937M	G	A	I	7806	0	0	0	0	NA	38051	0	0	0	0	NA
chr3:184328741	rs144059151	EIF4G1_M1356T	T	C	I	9255	0.0015127	14	0	9	5	40604	0.001453059	59	0	54	5
chr12:40351723	rs35658131	LRRK2_Y2189C	A	G	I	9257	0.0003241	3	0	3	0	40607	0.000369394	15	0	14	1
chr15:89318595	rs2307441	POLG_E1143G	T	C	I	29909	0.0556978	1665.864531	866.8645315	665	134	2484259	0.042580272	105780.4235	102534.4235	3040	206
chr3:184322040	rs112545306	EIF4G1_P486S	C	T	I	9256	0.0034572	32	0	25	7	40605	0.002881419	117	0	107	10
chr12:40251346	rs141262110	LRRK2_T358M	C	T	I	9256	0.0005402	5	0	5	0	40606	0.000566419	23	0	22	1
chr22:38132952	rs149653983	PLA2G6_T319M	G	A	I	9257	0.0012963	12	0	10	2	40607	0.000911173	37	0	36	1
chr15:61929659	rs149882066	VPS13C_A2043	G	C	I	7806	0	0	0	0	NA	38050	0	0	0	0	NA
chr1:155237458	rs367968666	GBA1_H294Q	A	C	I	28136	7.11E-05	2	0	2	0	3024452	2.98E-06	9	0	7	2
chr12:40257283	rs568593066	LRRK2_H442Y	C	T	I	7730	0	0	0	0	NA	37621	0	0	0	0	NA
chr14:22875400	rs201144028	LRP10_R151H	G	A	I	7806	0.0001281	1	0	1	NA	38051	2.63E-05	1	0	1	NA
chr3:132516425	rs142901736	DNAJC13_R183	G	A	I	0	NA	0	NA	NA	NA	0	NA	0	NA	NA	NA
chr1:20633840	rs575668171	PINK1_R98W	C	T	G	12134	0	0	0	0	NA	655511	2.14E-05	14	14	0	NA
chr15:61983908	rs557609723	VPS13C_K609I	T	A	I	7806	0	0	0	0	NA	38051	0	0	0	0	NA
chr1:20637894	rs138050841	PINK1_R147H	G	A	G	12159	0	0	0	0	NA	658925	6.07E-05	40	39	1	NA
chr15:62008671	rs150098264	VPS13C_T368A	T	C	I	9257	0.0017284	16	0	14	2	40605	0.001403768	57	0	54	3
chr15:89321776	rs796052888	POLG_R853Q	C	T	G	14529	0	0	0	NA	NA	2365117	1.69E-06	4	4	NA	NA
chr1:20644570	rs146691996	PINK1_P286L	C	T	G	22329	0	0	0	0	NA	2401308	4.21E-05	101	100	1	NA
chr6:161973403	rs137853060	PRKN_K211N	T	A	G	22312	0.0001793	4	3	1	NA	2399965	4.38E-05	105	104	1	NA
chr6:162443438	rs532703934	PRKN_V15M	C	T	G	4355	0	0	0	NA	NA	620988	4.83E-06	3	3	NA	NA
chr3:184320694	rs34838305	EIF4G1_R201H	G	A	I	9257	0.0005401	5	0	5	0	40605	0.00128063	52	0	50	2
chr4:41261921	rs139583787	UCHL1_R153W	C	T	I	9257	0.0003241	3	0	0	3	40607	2.46E-05	1	0	0	1
chr3:184321882	rs145998921	EIF4G1_A433V	C	T	I	7806	0.0001281	1	0	1	NA	38051	7.88E-05	3	0	3	NA
chr22:38120889	rs370691849	PLA2G6_R538C	G	A	G	22340	0	0	0	0	NA	2402847	4.95E-05	119	117	2	NA
chr12:40284011	rs35173587	LRRK2_R793M	G	T	I	32118	0.0007173	23.03762587	13.03762587	8	2	3097308	0.000586069	1815.236825	1743.236825	71	1
chr15:89330081	rs141367015	POLG_Q285H	C	G	G	23807	4.20E-05	1	0	1	0	2407637	9.72E-05	234	231	2	1
chr1:155236384	rs76539814	GBA1_T362I	G	A	G	26706	0.0001123	3	3	0	NA	3024638	2.45E-05	74	72	2	NA
chr1:16986321	rs533548757	ATP13A2_R1148	C	T	I	7806	0	0	NA	0	NA	38051	2.63E-05	1	NA	1	NA
chr3:132502299	rs139620588	DNAJC13_R151	G	A	I	9255	0.0079957	74	0	65	9	40599	0.008325328	338	0	324	14
chr3:184321599	rs191357892	EIF4G1_E339Q	G	C	I	23790	8.18E-05	1.944934224	1.944934224	0	0	2404668	0.000139886	336.3794912	316.3794912	18	2
chr15:61915890	rs35236516	VPS13C_R2730	G	A	I	0	NA	0	NA	NA	NA	0	NA	0	NA	NA	NA
chr2:232794835	rs116074753	GIGYF2_N457T	A	C	G	17610	0.0013061	23	12	9	2	734510	0.001241644	912	863	42	7
chr3:132505400	rs193294938	DNAJC13_E166	A	T	I	7762	0	0	0	0	NA	37893	2.64E-05	1	0	1	NA
chr1:20644665	rs139226733	PINK1_M318L	A	T	I	13592	0.0011007	14.96086636	2.960866359	9	3	659108	0.000722864	476.4456205	422.4456205	49	5
chr12:40323256	rs35602796	LRRK2_M1869T	T	C	I	17590	0.0004005	7.044177115	3.044177115	4	0	732916	0.000372365	272.9125581	252.9125581	19	1
chr15:89330250	rs202088074	POLG_V229G	A	C	I	0	NA	0	NA	NA	NA	0	NA	0	NA	NA	NA
chr15:89330184	rs113994094	POLG_T251I	G	A	I	31999	0.0031961	102.2704532	52.2704532	40	10	3080793	0.002331736	7183.595639	6987.595639	178	18
chr1:155235231	rs76071730	GBA1_H490R	T	C	G	8347	0	0	0	NA	NA	692919	1.44E-06	1	1	NA	NA
chr4:41261759	rs150601238	UCHL1_M124L	A	C	I	9252	0.0029183	27	0	23	4	40603	0.003595793	146	0	140	6
chr22:38133007	rs367854265	PLA2G6_R301C	G	A	I	9257	0.0003241	3	0	2	1	40606	0.000443284	18	0	17	1
chr15:61915712	rs72747885	VPS13C_I2789T	A	G	I	9257	0.020633	191	0	165	26	40601	0.019235979	781	0	744	37
chr12:40243556	rs28365216	LRRK2_N238I	A	T	G	16109	0.0001242	2	2	0	NA	732488	4.10E-06	3	3	0	NA
chr5:1432618	rs71653633	SLC6A3_L167F	G	A	I	9256	0.0025929	24	0	23	1	40607	0.002684266	109	0	105	4
chr15:89318641	rs755544706	POLG_R1128C	G	A	G	23804	4.20E-05	1	0	0	1	2406747	1.83E-05	44	42	1	1

chr20:5069196	rs570474631	TMEM230_P126	G	T	I	9257	0.0057254	53	0	41	12	40605	0.005664327	230	0	224	6
chr15:62007466	rs371570548	VPS13C_I378V	T	C	I	7806	0	0	0	0	NA	38051	0	0	0	0	NA
chr12:40320042	rs542505115	LRRK2_R1628C	C	T	I	7805	0	0	0	0	NA	38041	0	0	0	0	NA
chr3:132522838	rs145242123	DNAJC13_T189	C	T	I	9257	0.00821	76	0	66	10	40602	0.006132703	249	0	238	11
chr15:89328795	rs796052916	POLG_N354D	T	C	G	22327	0	0	0	0	NA	2401016	4.16E-06	10	10	0	NA
chr16:46674332	rs754978683	VPS35_N381S	T	C	I	7806	0	0	0	0	NA	38050	0.000315375	12	0	12	NA
chr15:89325466	rs781130302	POLG_V645L	C	A	I	0	NA	0	NA	NA	NA	0	NA	0	NA	NA	NA
chr15:89323504	rs185645212	POLG_R722H	C	T	I	7803	0	0	0	0	NA	38038	0	0	0	0	NA
chr15:89327201	rs113994095	POLG_A467T	C	T	I	23804	0.0015684	37.33478584	13.33478584	23	1	2407605	0.000956035	2301.753998	2169.753998	125	7
chr3:132522861	rs150102948	DNAJC13_V190	G	A	I	7806	0	0	0	0	NA	38051	0	0	0	0	NA
chr22:38169326	rs147948449	PLA2G6_S34L	G	A	I	9257	0.0019445	18	0	15	3	40606	0.001650002	67	0	65	2
chr1:155236444	rs77714449	GBA1_K342I	T	A	G	4347	0	0	0	NA	NA	620132	3.23E-06	2	2	NA	NA
chr1:20649095	rs747400197	PINK1_N451S	A	G	G	12159	8.22E-05	1	0	1	NA	658817	3.79E-05	25	24	1	NA
chr22:32498513	rs374363283	FBXO7_R518W	C	T	I	7806	0.0003843	3	0	3	NA	38051	0.000210244	8	0	8	NA
chr12:40305893	rs56320539	LRRK2_D1296Y	G	T	G	8354	0	0	0	NA	NA	693658	1.44E-06	1	1	NA	NA
chr15:89320953	rs121918048	POLG_H932Y	G	A	G	16131	0	0	0	0	NA	727676	6.87E-06	5	5	0	NA
chr22:38112165	rs140758033	PLA2G6_P806R	G	C	I	7806	0	0	0	0	NA	38051	2.63E-05	1	0	1	NA
chr12:40240535	rs111655870	LRRK2_L208F	G	T	I	7806	0	0	0	0	NA	38051	0	0	0	0	NA
chr21:32641895	rs765344810	NA	C	T	G	4352	0	0	0	NA	NA	620568	1.61E-06	1	1	NA	NA
chr1:20633615	rs551542832	PINK1_G23S	G	A	I	7806	0	0	0	0	NA	38050	0	0	0	0	NA
chr12:40225159	rs281865040	LRRK2_E10K	G	A	G	4354	0	0	0	NA	NA	620952	3.22E-06	2	2	NA	NA
chr2:232747740	rs72554080	GIGYF2_N56S	A	G	I	32104	0.0005972	19.17322127	10.17322127	7	2	3096409	0.000454292	1406.675358	1360.675358	41	5
chr1:17000494	rs145515028	ATP13A2_A244	G	A	I	9257	0.0007562	7	0	1	6	40607	0.000221637	9	0	7	2
chr1:20644515	rs372280083	PINK1_L268V	C	G	I	13607	0.0001708	2.324479894	0.324479894	1	1	661266	7.15E-05	47.29686587	46.29686587	0	1
chr3:132475009	rs149121829	DNAJC13_S790	C	A	I	9255	0.0215019	199	0	175	24	40594	0.021234665	862	0	808	54
chr22:38120886	rs143826762	PLA2G6_G539S	C	T	I	9257	0.0033488	31	0	29	2	40605	0.003768009	153	0	149	4
chr14:22875924	rs138170865	LRP10_G326S	G	A	I	9257	0.0012963	12	0	9	3	40606	0.001206718	49	0	44	5
chr15:89330106	rs138929605	POLG_H277L	T	A	I	23807	0.0012042	28.6688054	8.668805404	17	3	2408259	0.000620175	1493.541713	1410.541713	80	3
chr6:161360168	rs766915327	PRKN_R402H	C	T	G	12083	0	0	0	0	NA	645221	6.35E-05	41	38	3	NA
chr15:89333347	rs115109291	POLG_D136E	G	C	I	7806	0	0	0	0	NA	38051	0	0	0	0	NA
chr12:40354486	rs200762374	LRRK2_K2255M	A	T	I	7806	0	0	0	0	NA	38050	0.000131406	5	0	5	NA
chr15:89321217	rs375935084	POLG_P881L	G	A	I	23798	0.0001625	3.867907894	0.867907894	2	1	2407109	6.37E-05	153.2492791	141.2492791	12	0
chr12:40251369	rs113065049	LRRK2_V366M	G	A	G	22324	0	0	0	0	NA	2399784	2.50E-05	60	59	1	NA
chr3:184321315	rs147855566	EIF4G1_R244Q	G	A	I	7806	0	0	0	0	NA	38051	2.63E-05	1	0	1	NA
chr1:20648612	rs45478900	PINK1_G411S	G	A	I	17580	0.0006378	11.21217306	1.212173061	7	3	733165	0.000196225	143.8652365	100.8652365	38	5
chr15:61915725	rs145670690	VPS13C_R2785	G	A	I	7806	0	0	0	0	NA	38051	7.88E-05	3	0	3	NA
chr15:61961754	rs116290654	VPS13C_P1248	G	A	I	7806	0.0003843	3	0	3	NA	38050	0.000525624	20	0	20	NA
chr15:89333627	rs28567406	POLG_Q43R	T	C	I	9250	0.0058378	54	0	37	17	40569	0.006162341	250	0	231	19
chr1:16986335	rs377703085	ATP13A2_R104	G	A	I	9256	0.0016206	15	0	15	0	40607	0.00219174	89	0	86	3
chr1:16986101	rs41273151	ATP13A2_T1121	T	A	I	9254	0.0128593	119	0	105	14	40591	0.013796162	560	0	530	30
chr1:20637908	rs45608139	PINK1_R152W	C	T	I	22367	0	0	0	0	NA	2406844	4.15E-07	1	0	1	NA
chr15:61977193	rs115290371	VPS13C_T766N	G	T	I	7800	0	0	0	0	NA	38027	2.63E-05	1	0	1	NA
chr15:89327300	rs775538075	POLG_Y434H	A	G	G	22361	0	0	0	0	NA	2406058	2.95E-05	71	70	1	NA
chr12:40293629	rs201810995	LRRK2_C925Y	G	A	I	0	NA	0	NA	NA	NA	0	NA	0	NA	NA	NA
chr2:232847564	rs143181034	GIGYF2_Q1226	A	C	I	0	NA	0	NA	NA	NA	0	NA	0	NA	NA	NA
chr15:89326947	rs61752783	POLG_G517V	C	A	I	23812	0.0107296	255.4940797	107.4940797	125	23	2408163	0.007525376	18122.3319	17485.3319	605	32
chr15:61984938	rs551048142	VPS13C_K547R	T	C	I	0	NA	0	NA	NA	NA	0	NA	0	NA	NA	NA
chr21:32666479	rs373820739	SYNJ1_V675I	C	T	I	7806	0	0	0	0	NA	38051	0.000105122	4	0	4	NA
chr15:89318599	rs2307442	POLG_R1142W	G	A	G	22365	0	0	0	0	NA	2406360	1.79E-05	43	42	1	NA
chr11:94447275	rs139461096	MRE11_R576Q	C	T	I	9256	0.0004322	4	0	1	3	40607	0.000369394	15	0	11	4
chr20:5109435	rs149904653	TMEM230_R62	C	T	I	9257	0.0005401	5	0	4	1	40603	0.000270916	11	0	11	0
chr1:155235843	rs76763715	GBA1_N409S	T	C	G	34280	0.0142065	487	423	36	28	3105877	0.005856639	18190	18072	91	27
chr12:40351680	rs72547981	LRRK2_D2175H	G	C	G	11996	0.0003334	4	4	0	NA	635477	0.001534281	975	975	0	NA
chr1:155236367	rs374306700	GBA1_R368C	G	A	G	12148	0	0	0	0	NA	657459	4.56E-06	3	3	0	NA
chr22:38140106	rs587784359	PLA2G6_H225Y	G	A	G	14508	6.89E-05	1	1	NA	NA	2358579	6.15E-05	145	145	NA	NA
chr15:61961728	rs533261054	VPS13C_I1257V	T	C	I	7806	0.0001281	1	NA	1	NA	38051	0.000105122	4	NA	4	NA
chr22:38169336	rs150024227	PLA2G6_D31N	C	T	I	9257	0.0008642	8	0	7	1	40606	0.001083584	44	0	39	5
chr15:89318581	rs149099318	POLG_R1148C	G	A	G	22366	4.47E-05	1	1	0	NA	2406860	4.24E-05	102	101	1	NA
chr6:162054109	rs72480421	PRKN_H200Q	G	C	G	8349	0	0	0	NA	NA	694231	1.44E-06	1	1	NA	NA
chr1:16985990	rs76298930	ATP13A2_V1158	C	T	I	9257	0.0004321	4	0	3	1	40607	0.000270889	11	0	6	5
chr22:38115658	rs587784339	PLA2G6_R635X	G	A	G	21941	0	0	0	0	NA	2328526	9.62E-05	224	223	1	NA
chr1:65384226	rs186061249	DNAJC6_A177T	G	A	I	22334	4.48E-05	1	0	1	NA	2403510	0	0	0	0	NA
chr15:89320890	rs11546842	POLG_R953C	G	A	G	8366	0	0	0	NA	NA	695595	1.44E-06	1	1	NA	NA
chr15:89319065	rs181860632	POLG_R1047W	G	A	G	22360	8.94E-05	2	2	0	NA	2406211	9.85E-05	237	234	3	NA

chr15:61868702	rs116228685	VPS13C_R3607	C	T	I	9257	0.0020525	19	0	16	3	40605	0.001748553	71	0	68	3
chr2:232847399	rs72554081	GIGYF2_H1171	A	G	I	9256	0.0049697	46	0	41	5	40604	0.004063639	165	0	156	9
chr4:89828156	rs201106962	SNCA_H50Q	A	C	I	9257	0.0010803	10	0	8	2	40606	0.000443284	18	0	18	0
chr16:46683521	rs145147781	VPS35_M30K	A	T	I	9257	0.0009722	9	0	9	0	40607	0.000369394	15	0	15	0
chr15:61950998	rs11629598	VPS13C_I1495V	T	C	I	14944	0.1221926	1826.046323	442.0463233	1142	242	106040	0.104706513	11103.07861	5060.078614	5655	388
chr6:161350139	rs137853056	PRKN_W453X	C	T	G	4317	0	0	0	NA	NA	616383	1.95E-05	12	12	NA	NA
chr12:40225558	rs72546335	LRRK2_S52F	C	T	G	16168	0	0	0	0	NA	733363	1.36E-06	1	1	0	NA
chr15:61983868	rs116803472	VPS13C_D622E	G	T	I	7806	0	0	0	0	NA	38051	0	0	0	0	NA
chr2:74530221	rs150047108	HTRA2_L72P	T	C	I	9257	0.0085341	79	0	70	9	40603	0.008398394	341	0	325	16
chr21:32665968	rs147929290	SYNJ1_I746T	A	G	I	23803	0.0016531	39.34959179	19.34959179	15	5	2407943	0.001363453	3283.116133	3149.116133	127	7
chr6:161569357	rs137853055	PRKN_Q311X	G	A	G	14560	0	0	0	NA	NA	2369001	0	0	0	NA	NA
chr21:32673463	rs145712835	SYNJ1_V574I	C	T	I	9257	0.0003241	3	0	3	0	40607	0.000689536	28	0	25	3
chr15:89318710	rs753410045	POLG_A1105T	C	T	G	14558	0	0	0	NA	NA	2368636	2.53E-06	6	6	NA	NA
chr4:89828149	rs104893877	SNCA_A53T	C	T	G	20313	4.92E-05	1	1	NA	0	2983059	1.68E-06	5	5	NA	0
chr15:89328996	rs2307437	POLG_P324S	G	A	I	9257	0.0012963	12	0	11	1	40604	0.001256034	51	0	44	7
chr5:1443128	rs201800694	SLC6A3_V24M	C	T	I	9257	0.0015124	14	0	9	5	40606	0.001576122	64	0	59	5
chr3:184328662	rs112809828	EIF4G1_M1330	A	G	I	9257	0	0	0	0	0	40607	0.000320142	13	0	13	0
chr1:20648534	rs763416852	PINK1_F385L	T	C	G	12161	0	0	0	0	NA	659018	1.06E-05	7	7	0	NA
chr1:65392779	rs199937139	DNAJC6_H549P	A	C	I	9257	0.0003241	3	0	1	2	40607	0.000394021	16	0	11	5
chr15:89317460	rs369544574	POLG_R1187W	G	A	I	7806	0	0	0	0	NA	38051	0.000131403	5	0	5	NA
chr3:132480451	rs202174230	DNAJC13_R952	G	A	I	22333	4.48E-05	1	0	1	NA	2400858	2.08E-06	5	0	5	NA
chr6:161785839	rs377554392	PRKN_C268X	A	T	G	12149	0	0	0	0	NA	657004	9.13E-06	6	6	0	NA
chr3:132499779	rs3762672	DNAJC13_A146	G	T	I	26169	0.4958946	12977.06659	11652.06659	NA	1325	3063765	0.471798945	1445481.095	1443054.095	NA	2427
chr12:40257348	rs79656150	LRRK2_C463W	T	G	G	8358	0	0	0	NA	NA	694333	1.44E-06	1	1	NA	NA
chr3:184327434	rs34086109	EIF4G1_R1217	G	A	I	7806	0	0	0	0	NA	38051	0	0	0	0	NA
chr6:161785844	rs114696251	PRKN_Y267H	A	G	G	12156	0	0	0	0	NA	658635	1.06E-05	7	7	0	NA
chr22:38113591	rs587784346	PLA2G6_Q700X	G	A	G	14549	0	0	0	NA	NA	2366416	2.96E-06	7	7	NA	NA
chr1:155236376	rs2230288	GBA1_E365K	C	T	G	31950	0.0343975	1099	743	297	59	3095859	0.024148064	74759	73640	1071	48
chr3:184323921	rs62287499	EIF4G1_I807V	A	G	I	9256	0.0009723	9	0	9	0	40602	0.001970346	80	0	74	6
chr2:232844498	rs566316792	GIGYF2_G1077	G	C	I	0	NA	0	NA	NA	NA	0	NA	0	NA	NA	NA
chr3:132525718	rs138693725	DNAJC13_A205	G	T	I	9257	0.0021605	20	0	16	4	40605	0.002462751	100	0	90	10
chr1:155239639	rs794727708	GBA1_L144R	A	C	G	26695	7.49E-05	2	0	2	NA	3023638	1.32E-06	4	4	0	NA
chr1:155235813	rs77284004	GBA1_D419A	T	G	G	12102	8.26E-05	1	0	1	NA	650831	9.22E-06	6	5	1	NA
chr1:20649034	rs74315361	PINK1_Y431H	T	C	G	8270	0	0	0	NA	NA	691549	2.89E-06	2	2	NA	NA
chr22:38115583	rs587784341	PLA2G6_P660S	G	A	G	14541	0	0	0	NA	NA	2366583	3.80E-06	9	9	NA	NA
chr20:5106224	rs148033002	TMEM230_I62M	T	C	I	9256	0.0011884	11	0	10	1	40607	0.002068609	84	0	81	3
chr12:40363440	rs113511708	LRRK2_T2356I	C	T	I	13598	0.0002648	3.601009288	0.601009288	2	1	659958	0.00015569	102.7488375	85.74883751	17	0
chr1:175403578	rs61731112	TNR_N180H	T	G	I	23812	0.0080226	191.033198	81.03319797	95	15	2408848	0.005674107	13668.06123	13184.06123	453	31
chr12:40304040	rs60185966	LRRK2_S1228T	G	C	G	13591	0.0008094	11	3	7	1	658004	0.000656531	432	392	39	1
chr15:61991076	rs140027316	VPS13C_T501S	G	C	I	7806	0	0	0	0	NA	38044	0	0	0	0	NA
chr3:195868341	rs201407161	TNK2_V638M	C	T	I	9255	0.0024851	23	0	19	4	40606	0.001674629	68	0	62	6
chr2:232747717	rs147623346	GIGYF2_M48I	G	T	I	9257	0.0002161	2	0	0	2	40606	4.93E-05	2	0	1	1
chr12:40284061	rs72546337	LRRK2_I810V	A	G	G	16151	0	0	0	0	NA	731165	6.84E-06	5	5	0	NA
chr1:16986248	rs3170740	ATP13A2_A1072	C	T	I	5422	0.5947393	3224.676537	1865.676537	NA	1359	567887	0.472160562	268133.8451	265606.8451	NA	2527
chr1:20644526	rs28940284	PINK1_H271Q	C	A	G	6672	0	0	0	NA	NA	629121	3.18E-06	2	2	NA	NA
chr12:40309174	rs113589830	LRRK2_D1420N	G	A	G	14557	0	0	0	NA	NA	2368676	1.27E-06	3	3	NA	NA
chr22:38126390	rs201801144	PLA2G6_M470V	T	C	I	9257	0.0010803	10	0	9	1	40607	0.000837294	34	0	34	0
chr15:61929546	rs191668294	VPS13C_I2081F	T	A	I	7806	0	0	0	0	NA	38051	0	0	0	0	NA
chr12:40295466	rs75148313	LRRK2_S973N	G	A	G	22862	4.37E-05	1	1	NA	NA	3057111	1.96E-05	60	60	NA	NA
chr1:16993627	rs74058364	NA	A	C	I	0	NA	0	NA	NA	NA	0	NA	0	NA	NA	NA
chr15:89318677	rs201144044	POLG_M1116V	T	C	G	22356	8.95E-05	2	1	1	NA	2405598	7.27E-05	175	165	10	NA
chr1:17011732	rs549839037	ATP13A2_A3P	C	G	I	7806	0	0	0	0	NA	38051	0	0	0	0	NA
chr1:155235798	rs772548282	GBA1_L424P	A	G	G	4311	0	0	0	NA	NA	615508	2.27E-05	14	14	NA	NA
chr21:32673511	rs148901211	SYNJ1_S558G	T	C	I	9257	0.000108	1	0	0	1	40607	0.000147758	6	0	6	0
chr12:40323300	rs71653641	LRRK2_L1884F	C	T	G	8348	0.0001198	1	1	NA	NA	694179	7.20E-06	5	5	NA	NA
chr15:61951827	rs8026956	VPS13C_T1485	T	C	I	7806	0	0	0	0	NA	38051	0	0	0	0	NA
chr15:89322799	rs191490663	POLG_R790H	C	T	I	23793	0	0	0	0	0	2406632	4.16E-07	1	0	0	1
chr22:38133010	rs528966598	PLA2G6_A300T	C	T	I	9257	0	0	0	0	0	40607	7.39E-05	3	0	1	2
chr1:155236441	rs77321207	GBA1_Y343C	T	C	G	8356	0	0	0	NA	NA	695422	1.44E-06	1	1	NA	NA
chr22:38145447	rs141825182	PLA2G6_R139H	C	T	I	9255	0.0009724	9	0	5	4	40604	0.001256034	51	0	45	6
chr1:155237423	rs199628072	GBA1_T306I	G	A	I	9257	0.000108	1	0	1	0	40607	0.000295516	12	0	10	2
chr14:22875772	rs201377432	LRP10_T275N	C	A	I	0	NA	0	NA	NA	NA	0	NA	0	NA	NA	NA
chr2:232794778	rs142481025	GIGYF2_S438L	C	T	I	9257	0.0003241	3	0	3	0	40605	0.000123138	5	0	5	0
chr12:40278154	rs199566791	LRRK2_M712V	A	G	G	4354	0	0	0	NA	NA	620810	1.77E-05	11	11	NA	NA

chr3:184327609	rs35629949	EIF4G1_P1230A	C	G	I	9253	0.0095104	88	0	74	14	40586	0.009510669	386	0	347	39
chr15:61962790	rs3784635	VPS13C_I1132V	T	C	I	26460	0.0260857	690.2268751	630.2268751	NA	60	3064158	0.025210965	77250.3796	77152.3796	NA	98
chr22:38116105	rs139579057	PLA2G6_V617I	C	T	I	9257	0.0004321	4	0	2	2	40607	0.00019701	8	0	7	1
chr12:40367012	rs281865057	LRRK2_L2466H	T	A	G	12159	0	0	0	0	NA	658670	2.88E-05	19	19	0	NA
chr1:17005517	rs56379718	ATP13A2_G49S	C	T	I	32119	0.0001734	5.570479437	4.570479437	0	1	3099174	0.000198587	615.4566595	611.4566595	2	2
chr3:184315839	rs759151568	EIF4G1_S15P	T	C	I	8972	0.0015604	14	0	13	1	39337	0.001347332	53	0	48	5
chr20:5069205	rs553953692	TMEM230_V123	C	T	I	0	NA	0	NA	NA	NA	0	NA	0	NA	NA	NA
chr15:89325531	rs758438414	POLG_L623W	A	C	G	14538	0	0	0	NA	NA	2365596	4.23E-06	10	10	NA	NA
chr1:16988226	rs564643512	ATP13A2_R880I	C	T	I	9257	0.0011883	11	0	10	1	40607	0.000394021	16	0	15	1
chr15:89318989	rs530757118	POLG_T1072S	G	C	G	22356	0	0	0	0	NA	2405549	6.65E-06	16	16	0	NA
chr1:20645615	rs55831733	PINK1_A339T	G	A	I	27895	0.0020345	56.75347428	16.75347428	35	5	2986766	0.000943203	2817.125747	2648.125747	161	8
chr3:184320964	rs752416317	EIF4G1_T223M	C	T	I	7806	0	0	0	0	NA	38051	0	0	0	0	NA
chr22:38112186	rs587784354	PLA2G6_L799H	A	T	G	14502	0	0	0	NA	NA	2360669	8.47E-07	2	2	NA	NA
chr12:40351585	rs201271001	LRRK2_R2143H	G	A	I	13544	9.26E-05	1.254590262	0.254590262	1	0	652703	6.95E-05	45.34180997	36.34180997	8	1
chr1:155238264	rs188760929	GBA1_V211I	C	T	I	7805	0	0	0	0	NA	38048	0	0	0	0	NA
chr15:62034998	rs151147947	VPS13C_A81V	G	A	I	9257	0.0002161	2	0	2	0	40606	0.000418657	17	0	16	1
chr15:89320832	rs200309005	POLG_R972Q	C	T	G	22355	0	0	0	0	NA	2405511	2.54E-05	61	61	0	NA
chr22:38112558	rs121908686	PLA2G6_R741Q	C	T	G	26299	7.60E-05	2	1	1	NA	2475038	1.70E-05	42	41	1	NA
chr15:89327198	rs145843073	POLG_N468D	T	C	I	9256	0.0017286	16	0	15	1	40605	0.001477651	60	0	59	1
chr3:184331523	rs545768471	EIF4G1_L1439V	C	G	I	7806	0	0	0	0	NA	38051	0	0	0	0	NA
chr6:161386864	rs761213043	PRKN_R366Q	C	T	G	12161	8.22E-05	1	1	0	NA	658979	4.25E-05	28	25	3	NA
chr1:20649224	rs542258150	PINK1_A494V	C	T	I	7806	0	0	0	0	NA	38051	0	0	0	0	NA
chr1:155238215	rs364897	GBA1_N227S	T	C	G	30596	0.0001634	5	4	1	NA	3085807	6.48E-05	200	198	2	NA
chr15:89328699	rs199759055	POLG_R386C	G	A	G	22357	0	0	0	0	NA	2406175	3.28E-05	79	79	0	NA
chr12:40322038	rs72547979	LRRK2_R1725Q	G	A	G	16163	0	0	0	0	NA	732741	4.09E-06	3	1	2	NA
chr12:40340404	rs35870237	LRRK2_I2020T	T	C	G	26459	7.56E-05	2	2	NA	0	3065779	4.24E-06	13	13	NA	0
chr12:40293626	rs200795874	LRRK2_R924H	G	A	I	7806	0	0	0	0	NA	38051	2.63E-05	1	0	1	NA
chr1:65392613	rs377495314	DNAJC6_P494T	C	A	I	7806	0	0	0	0	NA	38051	0	0	0	0	NA
chr14:22876078	rs1062656	LRP10_P377R	C	G	I	7806	0	0	0	0	NA	38051	0	0	0	0	NA
chr14:22876722	rs142130715	LRP10_E486D	G	C	I	9257	0.0007562	7	0	6	1	40606	0.000443284	18	0	18	0
chr1:155237453	rs78973108	GBA1_R296Q	C	T	I	28132	0.0001422	4	0	2	2	3023697	3.31E-06	10	0	9	1
chr12:40359345	rs200002022	LRRK2_T2310M	C	T	I	7806	0.0003843	3	0	3	NA	38050	0	0	0	0	NA
chr12:40315266	rs721710	LRRK2_V1598E	T	A	G	4343	0	0	0	NA	NA	618985	1.62E-06	1	1	NA	NA
chr1:175406219	rs147204644	TNR_T166A	T	C	I	9256	0.0095073	88	0	70	18	40588	0.010323248	419	0	383	36
chr1:16990276	rs200924194	ATP13A2_Q750I	G	C	I	23801	0.0008344	19.86014688	6.860146883	12	1	2407112	0.000495652	1193.088738	1116.088738	72	5
chr15:61868675	rs77673743	VPS13C_G3616	C	T	I	9257	0.000108	1	0	0	1	40607	9.85E-05	4	0	2	2
chr15:89326688	rs2307447	POLG_R546C	G	A	I	22358	0	0	0	0	NA	2405148	1.25E-06	3	0	3	NA
chr3:132456751	rs147898644	DNAJC13_A423	C	T	I	9257	0.0003241	3	0	1	2	40607	0.000172384	7	0	7	0
chr22:32479132	rs139135860	FBXO7_D92H	G	C	G	12158	0.0002468	3	2	1	NA	658557	0.000267251	176	173	3	NA
chr15:62012166	rs769205655	NA	T	C	G	13133	7.61E-05	1	0	1	0	657779	7.15E-05	47	44	2	1
chr1:20644551	rs772510148	PINK1_A280T	G	A	G	12152	0	0	0	0	NA	658013	1.37E-05	9	8	1	NA
chr15:89323426	rs113994097	POLG_W748S	C	G	G	32085	0.001122	36	26	10	0	3097668	0.001140535	3533	3494	39	0
chr1:20649062	rs45467995	PINK1_G440E	G	A	G	22868	0	0	0	NA	NA	3058069	6.54E-07	2	2	NA	NA
chr12:40294909	rs373486170	LRRK2_S958L	C	T	I	7517	0	0	0	0	NA	36495	0	0	0	0	NA
chr20:5111588	rs191127079	TMEM230_S29L	G	A	I	0	NA	0	NA	NA	NA	0	NA	0	NA	NA	NA
chr1:65366050	rs61757223	DNAJC6_M76L	A	T	I	9257	0.0014043	13	0	12	1	40604	0.001157521	47	0	46	1
chr22:32475369	rs199636063	FBXO7_R3Q	G	A	I	7806	0	0	0	0	NA	38051	0	0	0	0	NA
chr3:184327433	rs201711322	EIF4G1_R1217C	C	T	I	9257	0.0002161	2	0	1	1	40607	9.85E-05	4	0	4	0
chr1:20638080	rs34677717	PINK1_P209L	C	T	I	17379	0.0001513	2.629007277	0.629007277	1	1	724059	9.52E-05	68.92985487	52.92985487	13	3
chr3:184323209	rs112019125	EIF4G1_G686C	G	T	I	9256	0.0007563	7	0	7	0	40607	0.000763415	31	0	30	1
chr12:40249843	rs200437744	LRRK2_L286V	C	G	I	9257	0.0009722	9	0	9	0	40606	0.000788061	32	0	29	3
chr15:89319318	rs201204229	POLG_V1005G	A	C	I	0	NA	0	NA	NA	NA	0	NA	0	NA	NA	NA
chr14:22875844	rs556418686	LRP10_N299S	A	G	I	7806	0	0	NA	0	NA	38051	2.63E-05	1	NA	1	NA
chr3:132457295	rs149087994	DNAJC13_V459	T	C	I	22348	0	0	0	0	NA	2404656	0	0	0	0	NA
chr3:184322835	rs538505681	EIF4G1_L604V	C	G	I	7806	0	0	0	0	NA	38051	7.88E-05	3	0	3	NA
chr6:162443384	rs770591350	PRKN_R33X	G	A	G	26706	3.74E-05	1	0	1	NA	3025589	7.60E-06	23	23	0	NA
chr15:62023788	rs201028815	VPS13C_R169H	C	T	I	7806	0.0001281	1	0	1	NA	38050	7.88E-05	3	0	3	NA
chr15:89320850	rs142347031	POLG_L966R	A	C	G	22362	0.0001342	3	2	1	NA	2406549	9.60E-05	231	224	7	NA
chr1:16996298	rs149372969	ATP13A2_L432V	G	C	I	9257	0.0035649	33	0	29	4	40605	0.003743381	152	0	146	6
chr14:22877421	rs149685154	LRP10_H679P	A	C	I	9257	0.0007562	7	0	6	1	40607	0.001403699	57	0	55	2
chr12:40310435	rs34995376	LRRK2_R1441H	G	A	G	34289	0.000175	6	6	0	0	3106004	4.51E-06	14	13	1	0
chr1:20644639	rs74315355	PINK1_G309D	G	A	G	4265	0	0	0	NA	NA	605576	4.13E-05	25	25	NA	NA
chr6:161973347	rs571490973	PRKN_A230V	G	A	I	7805	0	0	0	0	NA	38050	0	0	0	0	NA
chr12:40293644	rs281865045	LRRK2_Q930R	A	G	G	5798	0	0	0	NA	0	621892	1.61E-06	1	1	NA	0

chr15:89323445	rs147827654	POLG_V742M	C	T	G	22365	8.94E-05	2	2	0	NA	2406806	1.95E-05	47	47	0	NA
chr14:22877070	rs142153001	LRP10_R562H	G	A	I	9254	0.0184785	171	0	149	22	40584	0.017420658	707	0	681	26
chr22:38116155	rs149712244	PLA2G6_R600Q	C	T	G	22353	0	0	0	0	NA	2405354	4.99E-06	12	12	0	NA
chr15:61983818	rs775829630	NA	A	G	G	4354	0	0	0	NA	NA	620460	3.22E-06	2	2	NA	NA
chr12:40321114	rs35801418	LRRK2_Y1699C	A	G	G	24937	0	0	0	NA	NA	3049030	9.84E-07	3	3	NA	NA
chr1:16997044	rs113105667	ATP13A2_V386I	C	T	I	7805	0.0003844	3	0	3	NA	38051	0.000604452	23	0	23	NA
chr2:232791059	rs374338771	GIGYF2_V328L	G	C	I	7806	0.0001281	1	0	1	NA	38051	7.88E-05	3	0	3	NA
chr22:32475067	rs121918305	FBXO7_T22M	C	T	G	16061	6.23E-05	1	0	1	NA	715351	2.24E-05	16	16	0	NA
chr22:38140069	rs147066967	PLA2G6_R237H	C	T	I	9257	0.000108	1	0	0	1	40606	0.000221642	9	0	8	1
chr1:20633766	rs202048763	PINK1_S73L	C	T	I	7806	0	0	0	0	NA	38051	0	0	0	0	NA
chr15:89321773	rs796052889	POLG_A854D	G	T	G	14548	0	0	0	NA	NA	2366426	1.27E-06	3	3	NA	NA
chr12:40298433	rs76535406	LRRK2_S1096C	C	G	G	16120	6.20E-05	1	1	0	NA	725564	0.000119907	87	86	1	NA
chr3:184322631	rs145521479	EIF4G1_R566C	C	T	I	9257	0	0	0	0	0	40607	0.000147758	6	0	5	1
chr6:161785793	rs751037529	PRKN_G284R	C	G	G	12157	0	0	0	0	NA	658231	1.52E-06	1	1	0	NA
chr15:89322800	rs775168496	POLG_R790C	G	A	G	22362	4.47E-05	1	0	1	NA	2406543	3.82E-05	92	90	2	NA
chr2:232844499	rs538735187	GIGYF2_G1077A	G	C	I	0	NA	0	NA	NA	NA	0	NA	0	NA	NA	NA
chr15:89322748	rs796052887	POLG_R807H	C	T	G	22361	0	0	0	0	NA	2406097	4.16E-05	100	100	0	NA
chr12:40363541	rs79546190	LRRK2_V2390M	G	A	G	16160	0	0	0	0	NA	731180	2.60E-05	19	13	6	NA
chr14:22875639	rs35043211	LRP10_R231W	C	T	I	9257	0.0004321	4	0	4	0	40607	0.000246263	10	0	6	4
chr3:184322583	rs111924994	EIF4G1_A550P	G	C	I	9256	0.0034572	32	0	29	3	40604	0.003300167	134	0	125	9
chr1:16986334	rs151181674	ATP13A2_R1043	C	T	I	7806	0.0001281	1	0	1	NA	38051	2.63E-05	1	0	1	NA
chr1:155235772	rs80356769	GBA1_V433L	C	A	G	34280	0.0002625	9	8	0	1	3105859	4.80E-05	149	149	0	0
chr22:38140006	rs147924368	PLA2G6_S258L	G	A	I	9257	0.0003241	3	0	3	0	40607	0.000443273	18	0	17	1
chrX:121049176	rs9697983	GLUD2_S498A	T	G	I	3839	0.022435	86.12812077	86.12812077	NA	NA	66770	0.022435041	1497.987659	1497.987659	NA	NA
chr12:40322037	rs11564176	LRRK2_R1725X	C	T	G	16165	0	0	0	0	NA	732986	6.82E-05	50	50	0	NA
chr1:20644651	rs74315359	PINK1_T313M	C	T	G	29393	0.0003062	9	8	1	NA	2937705	0.000353677	1039	1039	0	NA
chr2:232811255	rs74560358	GIGYF2_A637V	C	T	I	7790	0	0	0	0	NA	37987	0	0	0	0	NA
chr3:132499777	rs61748103	DNAJC13_R146	G	A	I	9250	0.0255135	236	0	202	34	40590	0.022912047	930	0	870	60
chr15:61910283	rs10851704	VPS13C_S2913I	C	T	I	11967	0.5283604	6322.888806	5000.888806	NA	1322	701291	0.477434848	334820.7618	332283.7618	NA	2537
chr15:89325456	rs796052906	POLG_P648R	G	C	G	22308	4.48E-05	1	1	0	NA	2398175	4.29E-05	103	102	1	NA
chr15:89323460	rs121918054	POLG_G737R	C	G	I	23803	0.0025058	59.64510428	25.64510428	30	4	2406963	0.001802322	4338.121832	4171.121832	157	10
chr14:22876113	rs754181235	LRP10_D389N	G	A	I	9257	0.0005401	5	0	5	0	40607	0.000295516	12	0	11	1
chr3:132474976	rs137948208	DNAJC13_K779	A	G	I	7806	0	0	0	0	NA	38050	2.63E-05	1	0	1	NA
chr12:40278179	rs71653639	LRRK2_N720S	A	G	I	17605	8.57E-05	1.508779145	0.508779145	1	0	734645	6.98E-05	51.29900102	42.29900102	8	1
chr12:40251480	rs202170315	LRRK2_A373T	G	A	I	7806	0	0	0	0	NA	38050	0	0	0	0	NA
chr15:61941806	rs73430435	VPS13C_I1804V	T	C	I	7806	0	0	0	0	NA	38051	0	0	0	0	NA
chr15:89333177	rs3176162	POLG_R193Q	C	T	I	7806	0	0	0	0	NA	38051	0	0	0	0	NA
chr15:61919368	rs568860952	VPS13C_A2520	G	C	I	0	NA	0	NA	NA	NA	0	NA	0	NA	NA	NA
chr21:32678644	rs748818427	NA	C	A	G	4354	0	0	0	NA	NA	620832	3.22E-06	2	2	NA	NA
chr15:61962390	rs147306617	VPS13C_K1195T	T	G	I	9257	0.0003241	3	0	1	2	40607	2.46E-05	1	0	1	0
chr21:32639773	rs760863245	SYNJ1_P1238S	G	A	I	9257	0	0	NA	0	0	40606	4.93E-05	2	NA	1	1
chr22:38143150	rs185396488	PLA2G6_P25L	G	A	I	9257	0.000108	1	0	1	0	40607	0.000246263	10	0	9	1
chr5:1414780	rs577802449	SLC6A3_T356M	G	A	I	22363	0	0	0	0	NA	2406728	8.31E-07	2	0	2	NA
chr1:155236399	rs78188205	GBA1_A357D	G	T	G	4352	0	0	0	NA	NA	620720	1.13E-05	7	7	NA	NA
chr21:32643457	rs142964720	SYNJ1_G1183D	C	T	I	7804	0	0	0	0	NA	38036	0	0	0	0	NA
chr3:132522950	rs147575919	DNAJC13_D193	T	G	I	9257	0.0018364	17	0	16	1	40604	0.001748596	71	0	67	4
chr1:20649054	rs74315356	PINK1_W437X	G	A	G	30669	0	0	0	0	NA	3095997	1.42E-05	44	44	0	NA
chr15:89318986	rs267606959	POLG_P1073L	G	A	G	22316	0	0	0	0	NA	2401190	2.54E-05	61	60	1	NA
chr22:38123197	rs587784331	PLA2G6_L497F	G	A	G	22365	0	0	0	0	NA	2406036	2.49E-06	6	6	0	NA
chr6:161785805	rs72480422	PRKN_D280N	C	T	G	16170	0.0002474	4	1	3	NA	733263	5.32E-05	39	30	9	NA
chr14:22877184	rs139012932	LRP10_R600H	G	A	I	7806	0	0	0	0	NA	38051	5.26E-05	2	0	2	NA
chr12:40335031	rs77428810	LRRK2_R1941H	G	A	I	17595	0.0002595	4.565084485	0.565084485	3	1	731022	8.86E-05	64.79100143	46.79100143	18	0
chr15:61920571	rs139993005	VPS13C_H2380I	T	C	I	9256	0.0071305	66	0	52	14	40604	0.005984632	243	0	216	27
chr6:161548861	rs201300874	PRKN_G359D	C	T	G	26679	0	0	0	0	NA	3019815	7.62E-06	23	23	0	NA
chr1:20639934	rs573931674	PINK1_E240K	G	A	G	12157	0	0	0	0	NA	658348	1.52E-06	1	1	0	NA
chr1:20644564	rs113092523	PINK1_S284Y	C	A	I	7806	0	0	0	0	NA	38051	0	0	0	0	NA
chr22:32498414	rs141286570	FBXO7_V485I	G	A	I	9257	0.0028087	26	0	19	7	40604	0.001871737	76	0	72	4
chr1:17000272	rs56367069	ATP13A2_R289	C	T	I	32083	0.0209361	671.6919151	385.6919151	234	52	3093070	0.017089812	52859.98566	51568.98566	1188	103
chr22:38145597	rs142715413	PLA2G6_S89Y	G	T	I	7806	0	0	0	0	NA	38051	0	0	0	0	NA
chr1:155235819	rs754743440	GBA1_W417X	C	T	G	4348	0	0	0	NA	NA	620245	1.61E-06	1	1	NA	NA
chr12:40299125	rs34805604	LRRK2_I1122V	A	G	G	24857	0	0	0	NA	NA	3055382	3.93E-06	12	12	NA	NA
chr1:155236269	rs149487315	GBA1_M400I	C	T	I	7806	0	0	0	0	NA	38051	0	0	0	0	NA
chr15:61983936	rs116507802	VPS13C_I600V	T	C	I	7806	0	0	0	0	NA	38051	0	0	0	0	NA
chr20:5069288	rs186368464	TMEM230_R95H	C	T	I	9257	0.0029167	27	0	26	1	40607	0.002536508	103	0	102	1

chr1:17000107	rs150519745	ATP13A2_G310	C	T	G	22361	0	0	0	0	NA	2406394	4.40E-05	106	101	5	NA
chr6:162443357	rs577232474	PRKN_R42C	G	A	G	12160	0	0	0	0	NA	658930	1.97E-05	13	11	2	NA
chr3:132522832	rs139626514	DNAJC13_R189	G	A	I	7806	0.0001281	1	NA	1	NA	38050	5.26E-05	2	NA	2	NA
chr15:61931104	rs78071599	VPS13C_E2008	C	G	I	9248	0.0420631	389	0	325	64	40522	0.043260451	1753	0	1676	77
chr15:61918208	rs201120398	VPS13C_N2563	T	C	I	7806	0.0001281	1	0	1	NA	38035	0.000788747	30	0	30	NA
chr1:155235765	rs75385858	GBA1_N435T	T	G	G	8339	0	0	0	NA	NA	691781	1.45E-06	1	1	NA	NA
chr15:61984980	rs35435766	VPS13C_T533N	G	T	I	7802	0	0	0	0	NA	38032	0	0	0	0	NA
chr1:20644549	rs74315358	PINK1_R279H	G	A	G	16165	0.0004949	8	8	0	NA	732266	0.00029634	217	210	7	NA
chr1:16991787	rs201883464	ATP13A2_T728	G	A	G	23806	0	0	0	0	0	2408068	2.37E-05	57	57	0	0
chr22:38112534	rs587784351	PLA2G6_W749S	C	G	G	22340	4.48E-05	1	0	1	NA	2401943	1.96E-05	47	47	0	NA
chr1:65386886	rs201840876	DNAJC6_M300T	T	C	I	9257	0.0008642	8	0	6	2	40607	0.000541779	22	0	22	0
chr1:155235197	.	GBA1_N501K	G	C	G	12159	0	0	0	0	NA	658669	9.11E-06	6	5	1	NA
chr22:38112547	rs587784350	PLA2G6_R745W	G	A	G	22338	4.48E-05	1	1	0	NA	2403604	5.41E-06	13	12	1	NA
chr15:62010533	rs529383497	VPS13C_T317M	G	A	I	9257	0	0	0	0	0	40607	4.93E-05	2	0	1	1
chr15:61991096	rs143639809	NA	T	C	I	13598	0.0015152	20.6036874	3.603687397	17	0	659789	0.000954011	629.4458512	513.4458512	116	0
chr22:32498466	rs144538200	FBXO7_N502S	A	G	I	7806	0	0	0	0	NA	38051	5.26E-05	2	0	2	NA
chr12:40308481	rs72546338	LRRK2_R1325Q	G	A	I	32045	0.0007561	24.22775472	7.227754718	13	4	3088236	0.000329518	1017.628777	966.6287768	50	1
chr1:65385740	rs146050826	DNAJC6_A220T	G	A	I	9257	0.0008642	8	0	4	4	40605	0.00068957	28	0	24	4
chr15:89333357	rs540905618	POLG_D133G	T	C	I	7806	0.0002562	2	0	2	NA	38051	0.000183964	7	0	7	NA
chr15:89320944	rs760431841	POLG_T935P	T	G	G	14548	0	0	0	NA	NA	2365969	1.69E-06	4	4	NA	NA
chr3:184323468	rs111396765	EIF4G1_A718P	G	C	I	9257	0.0043211	40	0	35	5	40604	0.003546449	144	0	135	9
chr22:38113621	rs141777179	PLA2G6_V690I	C	T	I	23810	0.0008698	20.71082012	7.710820117	9	4	2408515	0.000538278	1296.450041	1254.450041	37	5
chr21:32701973	rs372488661	SYNJ1_R106W	G	A	I	7806	0	0	NA	0	NA	38045	2.63E-05	1	NA	1	NA
chr22:38132881	rs11570680	PLA2G6_A343T	C	T	I	9252	0.0258323	239	0	190	49	40567	0.024675229	1001	0	931	70
chr22:32498453	rs121918304	FBXO7_R498X	C	T	G	16165	6.19E-05	1	1	0	NA	731620	3.69E-05	27	26	1	NA
chr12:40294866	rs17519916	LRRK2_D944Y	G	T	G	30423	0	0	0	0	NA	3093641	1.87E-05	58	56	2	NA
chr12:40323270	rs281865054	LRRK2_E1874X	G	T	G	4351	0	0	0	NA	NA	619739	6.45E-06	4	4	NA	NA
chr2:232794852	rs115337999	GIGYF2_R463W	C	T	G	22359	0	0	0	0	NA	2406365	1.04E-05	25	23	2	NA
chr21:32664963	rs145978776	SYNJ1_D791Y	C	A	I	9256	0.0012965	12	0	12	0	40606	0.001699256	69	0	68	1
chr1:17000495	rs199661793	ATP13A2_A244T	C	T	I	7806	0.0002562	2	0	2	NA	38051	0	0	0	0	NA
chr2:74530433	rs387906942	HTRA2_P143A	C	G	G	22360	4.47E-05	1	0	1	NA	2405908	8.31E-07	2	2	0	NA
chr12:40319998	rs281865051	LRRK2_V1613A	T	C	G	4352	0	0	0	NA	NA	620848	3.22E-06	2	2	NA	NA
chr6:161548937	rs199657839	PRKN_R334C	G	A	I	26690	0	0	0	0	NA	3021301	0	0	0	0	NA
chr15:61936716	rs114590764	VPS13C_M1879	A	C	I	7806	0	0	0	0	NA	38051	0	0	0	0	NA
chr1:7965399	rs114601558	PARK7_A56T	G	A	I	9257	0	0	0	0	0	40607	0	0	0	0	0
chr15:61977090	rs76341351	VPS13C_R800S	T	G	I	7796	0	0	0	0	NA	38031	0	0	0	0	NA
chr15:89327006	rs121918052	POLG_Q497H	C	G	G	17388	5.75E-05	1	1	0	NA	1662739	0.000147949	246	243	3	NA
chr22:38120802	rs587784337	PLA2G6_E567K	C	T	G	14522	6.89E-05	1	1	NA	NA	2363884	4.65E-06	11	11	NA	NA
chr3:184327628	rs199929867	EIF4G1_A1236V	C	T	I	7806	0	0	NA	0	NA	38051	0.000105122	4	NA	4	NA
chr15:89333723	rs765472726	POLG_G11D	C	T	I	9257	0.0010803	10	0	9	1	40606	0.001452987	59	0	56	3
chr6:161569358	rs72480423	PRKN_E310D	C	G	I	17603	0.0008306	14.62116646	3.621166464	9	2	735437	0.000507778	373.4387898	301.4387898	70	2
chr15:89318736	rs368435864	POLG_R1096H	C	T	G	22366	0	0	0	0	NA	2406823	2.62E-05	63	63	0	NA
chr1:20648577	rs119451946	PINK1_P399L	C	T	G	30660	0	0	0	0	NA	3095483	2.58E-06	8	8	0	NA
chr1:155239633	rs758447515	GBA1_S146L	G	A	G	12150	0.0002469	3	0	3	NA	657504	1.52E-06	1	1	0	NA
chr22:38115619	rs794729212	PLA2G6_G648R	C	T	G	14558	6.87E-05	1	1	NA	NA	2367793	1.69E-06	4	4	NA	NA
chr14:22877106	rs200149859	LRP10_R574Q	G	A	I	7806	0	0	NA	0	NA	38048	5.26E-05	2	NA	2	NA
chr1:155235196	rs80356771	GBA1_R502C	G	A	G	32123	0.0014009	45	28	16	1	3098626	0.000410182	1271	1241	30	0
chr15:61856407	rs146072191	VPS13C_R3652	C	T	I	9257	0.0006482	6	0	5	1	40606	0.001132838	46	0	44	2
chr22:32484066	rs548204763	FBXO7_N196S	A	G	I	7806	0.0001281	1	0	1	NA	38051	5.26E-05	2	0	2	NA
chr12:40308507	rs201177073	LRRK2_R1334X	C	T	G	12154	8.23E-05	1	1	0	NA	657719	1.67E-05	11	11	0	NA
chr15:61882609	rs142572498	VPS13C_L3204	A	G	I	9257	0.0003241	3	0	2	1	40607	0.000935799	38	0	36	2
chr12:40293552	rs148113070	LRRK2_E899D	A	C	I	7789	0	0	0	0	NA	37972	0	0	0	0	NA
chr22:38132917	rs199935023	PLA2G6_D331N	C	T	I	9257	0.0003241	3	0	2	1	40607	4.93E-05	2	0	2	0
chr3:132528316	rs140537885	DNAJC13_L217	T	G	I	23807	0.0060974	145.1603246	64.16032455	70	11	2407100	0.004491301	10811.01105	10434.01105	357	20
chr2:232819840	rs200601366	GIGYF2_R795H	G	A	I	7756	0	0	0	0	NA	37806	0.000343861	13	0	13	NA
chr22:38169318	rs200075782	PLA2G6_R37X	G	A	G	26697	0.0001124	3	1	2	NA	3023779	0.000123686	374	370	4	NA
chr12:40263875	rs79996249	LRRK2_K544E	A	G	G	8352	0	0	0	NA	NA	694331	3.31E-05	23	23	NA	NA
chr3:132450732	rs149480465	DNAJC13_N141	A	T	I	9257	0.0007562	7	0	7	0	40603	0.00039406	16	0	15	1
chr1:16986554	rs201756175	ATP13A2_P1105	G	A	I	9257	0.000108	1	0	1	0	40607	0.000344768	14	0	10	4
chr14:22875363	rs28534929	LRP10_M139V	A	G	I	1451	0.0013784	2	0	NA	2	2556	0.001173709	3	0	NA	3
chr15:89318676	rs764036283	POLG_M1116T	A	G	G	14561	0	0	0	NA	NA	2368794	5.91E-06	14	14	NA	NA
chr5:1394741	rs200712598	SLC6A3_K619N	C	G	I	9257	0.0011883	11	0	10	1	40607	0.000985052	40	0	37	3
chr15:89321223	rs368587966	POLG_Q879R	T	C	G	22354	0	0	0	0	NA	2404940	3.74E-06	9	9	0	NA
chr15:89330257	rs121918056	POLG_R227W	G	A	G	16167	6.19E-05	1	1	0	NA	733520	2.32E-05	17	16	1	NA

chr1:20648528	rs45515602	PINK1_A383T	G	A	I	17623	0.0006103	10.75579275	1.755792754	7	2	736122	0.000220031	161.9694235	145.9694235	14	2
chr22:38112541	rs121908687	PLA2G6_R747W	G	A	G	22346	0	0	0	0	NA	2405180	9.56E-06	23	23	0	NA
chr15:89320907	rs796052891	POLG_K947R	T	C	G	14521	0	0	0	NA	NA	2362133	2.12E-06	5	5	NA	NA
chr14:22875648	rs371755191	LRP10_V234M	G	A	I	9257	0.0005401	5	0	5	0	40607	0.000566405	23	0	22	1
chr1:155236331	rs781306264	GBA1_A380T	C	T	G	4353	0	0	0	NA	NA	620571	1.61E-06	1	1	NA	NA
chr1:17004708	rs376169767	ATP13A2_A154	G	A	I	9257	0	0	NA	0	0	40607	7.39E-05	3	NA	3	0
chr3:132466351	rs199541720	DNAJC13_D674	A	C	I	9257	0.0007562	7	0	7	0	40607	0.000788041	32	0	31	1
chr3:132523168	rs61731474	DNAJC13_N195	A	C	I	9257	0	0	0	0	0	40607	4.93E-05	2	0	2	0
chr21:32656885	rs114053718	SYNJ1_I905T	A	G	I	23810	0	0	0	0	0	2408337	4.15E-07	1	0	1	0
chr1:155238209	rs75636769	GBA1_A229E	G	T	G	4354	0	0	0	NA	NA	620623	1.77E-05	11	11	NA	NA
chr15:61922557	rs141890160	VPS13C_I2272T	A	G	I	9257	0.0019445	18	0	14	4	40607	0.002561135	104	0	100	4
chr15:89320998	rs796052890	POLG_G917R	C	T	G	14553	0	0	0	NA	NA	2367702	8.45E-07	2	2	NA	NA
chr12:40257264	rs71653637	LRRK2_I435M	A	G	G	8345	0	0	0	NA	NA	691752	1.30E-05	9	9	NA	NA
chr22:38132914	rs587784364	PLA2G6_C332R	A	G	G	14504	0	0	0	NA	NA	2359608	4.24E-06	10	10	NA	NA
chr3:184327694	rs73053766	EIF4G1_N1258S	A	G	I	7804	0	0	0	0	NA	38045	0.000105139	4	0	4	NA
chr16:46682118	rs186122975	VPS35_R54W	G	A	G	22360	0	0	0	0	NA	2405930	8.31E-07	2	2	0	NA
chr1:155235195	rs80356772	GBA1_R502H	C	T	G	26707	3.74E-05	1	1	0	NA	3024932	1.32E-05	40	40	0	NA
chr1:155235006	rs146519305	GBA1_R534C	G	A	I	0	NA	0	NA	NA	NA	0	NA	0	NA	NA	NA
chr15:62008730	rs138433394	VPS13C_V348A	A	G	I	7806	0.0008967	7	0	7	NA	38050	0.001261498	48	0	48	NA
chr3:184317393	rs201980563	EIF4G1_P74S	C	T	G	14546	0	0	0	NA	NA	2367342	5.91E-06	14	14	NA	NA
chr22:38169423	rs587784356	PLA2G6_Q2K	G	T	G	14495	0	0	0	NA	NA	2358356	1.40E-05	33	33	NA	NA
chr12:40363410	rs75695264	LRRK2_Y2346C	A	G	G	8154	0.0002453	2	2	NA	NA	656516	0.000851464	559	559	NA	NA
chr4:41263258	rs564599222	UCHL1_F165L	T	C	I	7806	0	0	0	0	NA	38051	0	0	0	0	NA
chr15:89316763	rs3087374	POLG_Q1236H	C	A	I	32823	0.0984822	3232.480239	2002.480239	1230	NA	3103116	0.080946477	251186.3092	245211.3092	5975	NA
chr15:61922408	rs12907567	VPS13C_V2322I	C	T	I	34237	0.0981122	3359.066234	1959.066234	1159	241	3103929	0.079248642	245982.1569	239872.1569	5723	387
chr6:161360187	rs539917500	PRKN_R396G	T	C	G	12156	0	0	0	0	NA	658626	1.52E-06	1	1	0	NA
chr6:162262681	rs747891099	PRKN_D86N	C	T	G	12157	0	0	0	0	NA	658690	3.19E-05	21	18	3	NA
chr15:62013943	rs116305045	VPS13C_I245T	A	G	I	7805	0	0	0	0	NA	38038	0	0	0	0	NA
chr12:40263861	rs55968234	LRRK2_K539R	A	G	G	16122	0.0001861	3	0	3	NA	730874	0.000125877	92	82	10	NA
chr15:89321780	rs144500145	POLG_R852C	G	A	G	23792	4.20E-05	1	0	1	0	2406153	5.82E-06	14	1	10	3
chr15:89318553	rs548076633	POLG_N1157S	T	C	G	22335	0	0	0	0	NA	2401375	1.54E-05	37	37	0	NA
chr22:38132850	rs587784326	PLA2G6_P353L	G	A	G	22286	8.97E-05	2	1	1	NA	2389045	1.88E-05	45	44	1	NA
chr17:46010389	rs63751273	MAPT_P301L	C	T	G	22864	0	0	0	NA	NA	3058136	1.37E-05	42	42	NA	NA
chr15:62035002	rs116290723	VPS13C_V80F	C	A	I	7806	0	0	0	0	NA	38051	0	0	0	0	NA
chr1:155235823	rs121908311	GBA1_G416S	C	T	G	30575	3.27E-05	1	0	1	NA	3082776	0.000138836	428	426	2	NA
chr1:16987157	rs145548316	ATP13A2_R947	C	T	G	22146	0.0003612	8	8	0	NA	2369144	0.000331765	786	785	1	NA
chr15:61922680	rs371685718	VPS13C_T2231	G	T	I	9257	0.000108	1	0	1	0	40607	0.000172384	7	0	7	0
chr1:16986065	rs542270701	ATP13A2_V1133	C	T	I	9257	0.0002161	2	0	1	1	40606	0.000517165	21	0	19	2
chr1:20644626	rs112600292	PINK1_P305A	C	G	I	22350	0	0	0	0	NA	2403797	0	0	0	0	NA
chr22:38123244	rs587784330	PLA2G6_L481Q	A	T	G	14494	0	0	0	NA	NA	2359249	3.39E-06	8	8	NA	NA
chr15:61915865	rs532971259	VPS13C_P2738	G	C	I	7806	0	0	0	0	NA	38051	0	0	0	0	NA
chr21:32726855	rs565807712	SYNJ1_D53G	T	C	I	7806	0	0	0	0	NA	38051	0	0	0	0	NA
chr15:61940726	rs77555508	VPS13C_S1841	G	A	I	7806	0	0	0	0	NA	38051	2.63E-05	1	0	1	NA
chr3:184322551	rs561916314	EIF4G1_P539L	C	T	I	9257	0	0	0	0	0	40607	4.93E-05	2	0	0	2
chr3:184323412	rs373584582	EIF4G1_G699A	G	C	I	9257	0.0008642	8	0	7	1	40606	0.00071418	29	0	28	1
chr3:132516758	rs113742727	DNAJC13_R187	G	A	I	22365	0	0	0	0	NA	2406566	0	0	0	0	NA
chr1:16997107	rs142616130	ATP13A2_R365	G	A	G	22275	0.0002694	6	6	0	NA	2394146	0.000114028	273	273	0	NA
chr12:40351579	rs111691891	LRRK2_T2141M	C	T	G	12136	0	0	0	0	NA	654813	2.60E-05	17	17	0	NA
chr15:89333327	rs796052899	POLG_A143V	G	A	G	22274	4.49E-05	1	1	0	NA	2389863	4.06E-05	97	96	1	NA
chr22:38126451	rs797045888	NA	T	C	G	22334	4.48E-05	1	1	0	NA	2402170	2.71E-05	65	65	0	NA
chr1:16986291	rs544885605	ATP13A2_R115	C	T	I	9256	0.0003241	3	0	2	1	40606	0.000418657	17	0	16	1
chr15:61978646	rs140060073	VPS13C_Q757R	T	C	I	9257	0.0002161	2	0	0	2	40607	0.000221637	9	0	2	7
chr15:89320856	rs767339769	POLG_R964H	C	T	G	22357	0	0	0	0	NA	2404720	4.16E-06	10	10	0	NA
chr1:155238214	rs381418	GBA1_N227K	A	C	G	6219	0	0	0	NA	NA	351591	2.56E-05	9	9	NA	NA
chr1:17000429	rs561097876	ATP13A2_I266V	T	C	I	0	NA	0	NA	NA	NA	0	NA	0	NA	NA	NA
chr6:161785778	rs55961220	PRKN_C289G	A	C	G	18902	0	0	0	NA	NA	2987442	6.69E-07	2	2	NA	NA
chr12:40310515	rs281865050	LRRK2_K1468E	A	G	G	12160	0	0	0	0	NA	658840	1.37E-05	9	8	1	NA
chr15:61922527	rs138540886	VPS13C_S2282	G	A	I	7806	0.0001281	1	0	1	NA	38048	0.000420521	16	0	16	NA
chr3:132499231	rs61748102	DNAJC13_A142	C	T	I	23799	0.0242659	577.5032176	270.5032176	264	43	2407466	0.018890982	45479.39626	44018.39626	1372	89
chr15:61966088	rs747812804	VPS13C_V1016	C	T	I	9256	0	0	0	0	0	40593	4.93E-05	2	0	0	2
chr15:89323462	rs138457939	POLG_N736S	T	C	I	9257	0.0008642	8	0	6	2	40605	0.000738825	30	0	28	2
chr12:40232383	rs144883021	LRRK2_Q116R	A	G	I	7799	0	0	0	0	NA	38000	2.63E-05	1	0	1	NA
chr1:155238144	rs121908300	GBA1_Y251H	A	G	G	22852	0	0	0	NA	NA	3056863	3.27E-07	1	1	NA	NA
chr1:20644627	rs7349186	PINK1_P305L	C	T	G	8361	0	0	0	NA	NA	695399	1.29E-05	9	9	NA	NA

chr1:155238246	.	GBA1_P217S	G	A	G	12151	8.23E-05	1	0	1	NA	657416	3.04E-06	2	2	0	NA
chr15:89330133	rs61752784	POLG_G268A	C	G	I	23763	0.0071754	170.5092548	64.50925478	87	19	2403533	0.004512395	10845.6892	10506.6892	312	27
chr1:16992115	rs143834516	ATP13A2_A669T	C	T	I	7806	0	0	0	0	NA	38051	0	0	0	0	NA
chr6:161785825	rs373750972	PRKN_N273S	T	C	G	12148	0.0001646	2	0	2	NA	655399	8.70E-05	57	52	5	NA
chr15:61940651	rs142341124	VPS13C_M1866	A	G	I	9257	0	0	0	0	0	40607	2.46E-05	1	0	0	1
chr2:232794843	rs2289912	GIGYF2_P460T	C	A	I	19972	0.0208617	416.6500667	358.6500667	NA	58	2440892	0.019388477	47325.17883	47217.17883	NA	108
chr1:16990191	rs137955309	ATP13A2_R778	C	T	I	7806	0	0	0	0	NA	38050	7.88E-05	3	0	3	NA
chr12:40340380	rs34015634	LRRK2_I2012T	T	C	G	30674	0	0	0	0	NA	3095860	1.29E-06	4	4	0	NA
chr21:32631012	rs536572904	SYNJ1_D1608Y	C	A	I	7806	0	0	0	0	NA	38051	0	0	0	0	NA
chr1:155237444	rs794727908	GBA1_I299T	A	G	G	22359	0	0	0	0	NA	2405896	8.31E-07	2	2	0	NA
chr12:40309225	rs74163686	LRRK2_N1437H	A	C	G	14554	0	0	0	NA	NA	2366953	4.22E-07	1	1	NA	NA
chr1:155239736	.	GBA1_Q112X	G	A	G	12160	0	0	0	0	NA	658881	7.59E-06	5	4	1	NA
chr3:184323541	rs147678593	EIF4G1_T742M	C	T	I	22365	0	0	0	0	NA	2406564	0	0	0	0	NA
chr1:7965425	rs74315353	PARK7_E64D	G	C	G	4337	0	0	0	NA	NA	619088	1.62E-06	1	1	NA	NA
chr15:89328532	rs145289229	POLG_L392V	G	C	I	23745	0.0022731	53.97568508	15.97568508	34	4	2399721	0.001166047	2798.18753	2601.18753	189	8
chr12:40302860	rs533532047	LRRK2_E1190K	G	A	I	0	NA	0	NA	NA	NA	0	NA	0	NA	NA	NA
chr15:62023836	rs12595158	VPS13C_R153H	C	T	I	19966	0.0231368	461.9488766	408.9488766	NA	53	2439765	0.022099546	53917.69768	53831.69768	NA	86
chr12:40232385	rs141221000	NA	T	C	I	12150	0	0	0	0	NA	658649	1.52E-06	1	0	1	NA
chr1:20649134	rs764328076	PINK1_R464H	G	A	G	22358	8.95E-05	2	1	1	NA	2405567	7.73E-05	186	182	4	NA
chr1:7984981	rs28938172	PARK7_L166P	T	C	G	18902	0	0	0	NA	NA	2987018	1.00E-06	3	3	NA	NA
chr2:74532831	rs550171858	HTRA2_R408Q	G	A	I	7806	0	0	0	0	NA	38051	0	0	0	0	NA
chr14:22877128	rs570350751	LRP10_Q581H	G	T	I	7806	0	0	0	0	NA	38051	0	0	0	0	NA
chr3:132503289	rs566485027	DNAJC13_A159	G	A	I	7806	0	0	0	0	NA	38051	0	0	0	0	NA
chr15:61934224	rs112236709	VPS13C_N1955	T	G	I	15984	0.0088362	141.2376733	117.2376733	NA	24	2365716	0.008077726	19109.60558	19063.60558	NA	46
chr5:1403013	rs28364997	SLC6A3_A559V	G	A	I	17602	0.0033413	58.81289262	7.81289262	49	2	735234	0.0010762	791.2590208	650.2590208	139	2
chr12:40323255	rs281865052	LRRK2_M1869V	A	G	G	4340	0	0	0	NA	NA	619285	3.23E-06	2	2	NA	NA
chr2:232794782	rs146944691	GIGYF2_Q439H	G	C	I	7805	0.001025	8	0	8	NA	38050	0.000762155	29	0	29	NA
chr6:161360129	rs778125254	PRKN_T415N	G	T	G	18903	0	0	0	NA	NA	2987432	1.14E-05	34	34	NA	NA
chr15:89318737	rs201732356	POLG_R1096C	G	A	G	22353	4.47E-05	1	0	1	NA	2403569	4.87E-05	117	117	0	NA
chr15:61854543	rs115819951	VPS13C_I3726V	T	C	I	9255	0.0099406	92	0	75	17	40593	0.010469785	425	0	397	28
chr15:89319073	rs150233690	POLG_V1044A	A	G	I	9257	0.0014043	13	0	10	3	40607	0.001970104	80	0	78	2
chr12:40340400	rs34637584	LRRK2_G2019S	G	A	G	34291	0.0137062	470	426	20	24	3106080	0.001089798	3385	3359	22	4
chr15:89318962	rs140079523	POLG_R1081Q	C	T	G	22310	0	0	0	0	NA	2397531	2.88E-05	69	69	0	NA
chr3:132454135	rs147315244	DNAJC13_R304	C	T	G	23732	0.0001264	3	1	1	1	2394456	6.72E-05	161	158	3	0
chr15:89325591	rs367610201	POLG_M603T	A	G	G	23811	4.20E-05	1	1	0	0	2408581	7.43E-05	179	174	4	1
chr15:62044256	rs757475739	NA	C	T	G	11159	0.0001792	2	1	1	NA	642280	0.000146354	94	88	6	NA
chr15:89321777	rs121918053	POLG_R853W	G	A	G	22773	0	0	0	NA	NA	3048442	5.02E-05	153	153	NA	NA
chr22:38132979	rs121908682	PLA2G6_V310E	A	T	G	22825	0	0	0	NA	NA	3051517	6.55E-07	2	2	NA	NA
chr21:32657057	rs370346211	SYNJ1_T881I	G	A	I	7806	0	0	0	0	NA	38051	0	0	0	0	NA
chr1:175386035	rs140481433	TNR_T592A	T	C	I	9257	0.0017284	16	0	15	1	40605	0.002314986	94	0	91	3
chr1:155235829	rs398123528	GBA1_V414L	C	A	G	18902	0	0	0	NA	NA	2987087	4.69E-06	14	14	NA	NA
chr15:61967438	rs3784634	VPS13C_R974K	C	T	I	25933	0.4448862	11537.23403	10292.23403	NA	1245	3004178	0.420717094	1263909.039	1261882.039	NA	2027
chr15:89323451	rs78347903	POLG_N740D	T	C	I	7806	0	0	0	0	NA	38050	7.88E-05	3	0	3	NA
chr15:89330258	rs147282197	POLG_Q226H	C	G	I	9257	0.0015124	14	0	11	3	40606	0.001379107	56	0	52	4
chr2:232832882	rs375089299	GIGYF2_R852Q	G	A	I	7805	0.0005125	4	0	4	NA	38050	0.000630749	24	0	24	NA
chr17:46024061	rs63750424	MAPT_R406W	C	T	G	30669	6.52E-05	2	2	0	NA	3095933	2.45E-05	76	75	1	NA
chr1:155236426	rs78396650	GBA1_A348V	G	A	G	8363	0	0	0	NA	NA	695147	5.75E-06	4	4	NA	NA
chr1:155236277	rs121908309	GBA1_R398X	G	A	G	22356	0	0	0	0	NA	2405470	2.08E-06	5	4	1	NA
chr1:16986208	rs56004722	ATP13A2_G108	C	T	I	4010	0	0	0	NA	NA	74605	0	0	0	NA	NA
chr12:40274666	rs145906734	LRRK2_L580F	A	T	I	7805	0	0	0	0	NA	38051	0	0	0	0	NA
chr15:61910215	rs148074630	VPS13C_R2936	G	A	G	12140	0	0	0	0	NA	657186	9.13E-06	6	6	0	NA
chr3:132502298	rs79734612	DNAJC13_R151	C	T	I	7806	0	0	0	0	NA	38049	7.88E-05	3	0	3	NA
chr1:155238570	rs147138516	GBA1_D179H	C	G	I	32089	0.0001862	5.976421864	1.976421864	4	0	3095742	8.80E-05	272.4638047	264.4638047	8	0
chr3:132528231	rs181058582	DNAJC13_E214	G	A	G	22355	0	0	0	0	NA	2405215	5.20E-05	125	119	6	NA
chr15:62010558	rs114954486	VPS13C_P309A	G	C	I	7806	0	0	0	0	NA	38051	2.63E-05	1	0	1	NA
chr12:40263806	rs35328937	LRRK2_R521G	A	G	I	17590	0.0002596	4.56661532	1.56661532	0	3	732121	0.000180308	132.0070754	130.0070754	0	2
chr1:155236409	rs398123526	GBA1_D354H	C	G	G	22358	8.95E-05	2	0	2	NA	2405889	2.49E-06	6	5	1	NA
chr1:155235708	rs121908295	GBA1_P454R	G	C	G	8351	0.0007185	6	6	NA	NA	695178	5.18E-05	36	36	NA	NA
chr3:132496652	rs200244570	DNAJC13_R138	G	A	G	22355	0	0	0	0	NA	2404897	3.99E-05	96	96	0	NA
chr1:155240048	rs760930573	GBA1_G49S	C	T	G	12160	0	0	0	0	NA	659000	1.52E-05	10	10	0	NA
chr12:40240594	rs56108242	LRRK2_C228S	G	C	I	17574	0.0002701	4.746487862	0.746487862	3	1	732484	9.30E-05	68.09904807	62.09904807	6	0
chr15:61890400	rs149077892	VPS13C_D3036	C	T	I	9257	0.000108	1	0	1	0	40607	0.000123131	5	0	5	0
chr15:89319234	rs551708243	POLG_A1033V	G	A	I	9257	0.000108	1	0	1	0	40606	0.000197015	8	0	6	2
chr15:89319053	rs121918049	POLG_G1051R	C	G	G	22355	0.0001342	3	3	0	NA	2405096	0.000111014	267	266	1	NA

chr3:184325887	rs199973292	EIF4G1_N1054S	A	G	G	14546	0	0	0	NA	NA	2366806	2.96E-06	7	7	NA	NA
chr5:176621218	rs104893937	SNCB_P123H	G	T	I	17613	0.0004312	7.595151992	2.595151992	2	3	734664	0.000315116	231.504427	215.504427	14	2
chr12:40293624	rs58559150	LRRK2_Q923H	G	C	I	28107	0.0001292	3.63158122	1.63158122	0	2	3020985	8.87E-05	267.9423283	257.9423283	9	1
chr2:74530208	rs146380692	HTRA2_P68S	C	T	I	7806	0	0	0	0	NA	38051	0	0	0	0	NA
chr15:61916007	rs139139141	VPS13C_H2691I	G	C	I	9257	0.000108	1	0	0	1	40606	0.000295523	12	0	10	2
chr22:32479135	rs143041875	FBXO7_S93A	T	G	I	9257	0.0007562	7	0	7	0	40605	0.000566433	23	0	21	2
chr15:89325562	rs147407423	POLG_H613Y	G	A	I	7806	0	0	0	0	NA	38050	0	0	0	0	NA
chr6:161785820	rs34424986	PRKN_R275W	G	A	I	34155	0.0040471	138.230008	67.23000801	64	7	3085539	0.002765271	8532.35248	8221.35248	291	20
chr22:38113561	rs587784347	PLA2G6_R710C	G	A	G	22351	4.47E-05	1	1	0	NA	2403860	9.15E-06	22	20	2	NA
chr12:40363526	rs34778348	LRRK2_G2385R	G	A	I	34291	2.92E-05	1	0	0	1	3105978	0	0	0	0	0
chr5:122450987	rs143468222	SNCAIP_A714T	G	A	I	9257	0.0002161	2	0	0	2	40607	0.000123131	5	0	3	2
chr20:5100832	rs143571424	TMEM230_R108	G	A	I	9252	0.0039991	37	0	29	8	40591	0.004015668	163	0	151	12
chr22:38128349	rs199636953	PLA2G6_A423V	G	A	I	9257	0.0002161	2	0	2	0	40605	0.00078808	32	0	32	0
chr22:38135029	rs201190836	PLA2G6_R285C	G	A	G	22354	8.95E-05	2	1	1	NA	2405311	7.28E-05	175	169	6	NA
chr15:61962754	rs776169337	VPS13C_K1144	T	A	G	4308	0	0	0	NA	NA	615000	6.50E-06	4	4	NA	NA
chr15:89323423	rs202037973	POLG_F749S	A	G	G	22364	0	0	0	0	NA	2406538	5.40E-06	13	13	0	NA
chr1:7961737	rs140230911	NA	T	A	I	0	NA	0	NA	NA	NA	0	NA	0	NA	NA	NA
chr6:162443429	rs146288080	PRKN_D18N	C	T	I	12159	0	0	0	0	NA	658846	0	0	0	0	NA
chr1:20644662	rs200949139	PINK1_V317I	G	A	I	26710	0.0001137	3.038166212	1.038166212	2	NA	3025498	5.49E-05	166.0640909	164.0640909	2	NA
chr3:184327401	rs112176450	EIF4G1_R1206H	G	A	I	23814	0.0005197	12.37614895	2.376148954	8	2	2409008	0.000170431	410.5693046	386.5693046	24	0
chr1:7985019	rs71653622	PARK7_A179T	G	A	I	9257	0.0012963	12	0	9	3	40601	0.000763528	31	0	28	3
chr3:132484650	rs202127368	DNAJC13_T108	C	T	I	9257	0.0011883	11	0	9	2	40604	0.001428431	58	0	52	6
chr12:40305946	rs183902574	LRRK2_C1313X	T	A	G	26563	0.0001129	3	1	2	NA	3000667	0.000107643	323	322	1	NA
chr6:161350125	rs182893847	PRKN_M458L	T	G	G	12154	0.0002468	3	1	2	NA	657918	0.000158074	104	93	11	NA
chr1:155237438	rs140955685	GBA1_R301H	C	T	I	13605	0.0007758	10.55484451	0.554844513	8	2	660766	0.000150035	99.13795276	79.13795276	20	0
chr12:40302866	rs281865047	LRRK2_I1192V	A	G	I	12081	0	0	0	0	NA	656190	1.52E-06	1	0	1	NA
chr1:65385756	rs368061386	DNAJC6_R225H	G	A	I	7806	0	0	0	0	NA	38051	5.26E-05	2	0	2	NA
chr15:61936649	rs538854121	VPS13C_E1901I	C	G	I	7806	0.0001281	1	0	1	NA	38051	2.63E-05	1	0	1	NA
chr1:20649217	rs34208370	PINK1_R492X	C	T	I	30615	7.20E-05	2.205434624	1.205434624	1	NA	3090238	5.45E-05	168.3052694	161.3052694	7	NA
chr15:89320857	rs201477273	POLG_R964C	G	A	G	22364	0	0	0	0	NA	2406730	1.45E-05	35	35	0	NA
chr22:38120867	rs121908681	PLA2G6_K545T	T	G	G	22796	8.77E-05	2	2	NA	NA	3049377	1.08E-05	33	33	NA	NA
chr3:184327230	rs375604272	EIF4G1_R1149C	G	A	I	9257	0	0	NA	0	0	40607	4.93E-05	2	NA	1	1
chr1:20648601	rs556540177	PINK1_R407Q	G	A	G	12159	0	0	0	0	NA	658809	4.10E-05	27	27	0	NA
chr6:162443383	rs147757966	PRKN_R33Q	C	T	G	23810	4.20E-05	1	0	1	0	2409054	4.48E-05	108	89	18	1
chr15:61984004	rs200452364	VPS13C_A577V	G	A	I	7806	0	0	NA	0	NA	38051	2.63E-05	1	NA	1	NA
chr6:161973335	rs144032774	PRKN_R234Q	C	T	I	28161	3.70E-05	1.041280706	1.041280706	0	0	3028102	5.67E-05	171.5589264	164.5589264	5	2
chr1:20639911	rs144071530	PINK1_A232V	C	T	G	22358	0	0	0	0	NA	2405589	4.16E-07	1	1	0	NA
chr12:40235634	rs33995463	LRRK2_L119P	T	C	I	32073	0.0031116	99.79918826	54.79918826	38	7	3095408	0.002425171	7506.893627	7328.893627	165	13
chr6:161360169	rs55830907	PRKN_R402C	G	A	I	9256	0.0033492	31	0	26	5	40596	0.003029855	123	0	109	14
chr1:20645675	rs76753586	PINK1_A359T	G	A	G	22360	0	0	0	0	NA	2405987	4.03E-05	97	97	0	NA
chr14:22873373	rs2273837	LRP10_R48W	C	T	G	13268	0.0005276	7	4	2	1	115216	0.000225663	26	24	2	0
chr15:61880871	rs140034886	VPS13C_T3287I	G	A	I	9257	0.0003241	3	0	3	0	40606	0.000344777	14	0	13	1
chr15:50586433	rs8042919	TRPM7_T1482I	G	A	I	19734	0.1558004	3074.564988	1094.564988	1681	299	739880	0.109962369	81358.95774	72764.95774	8049	545
chr22:32493191	rs762037477	FBXO7_V352I	G	A	I	7804	0.0002563	2	0	2	NA	38050	0.000289093	11	0	11	NA
chr3:132494190	rs61748101	DNAJC13_E129	A	G	G	23750	0.0116211	276	144	119	13	2397221	0.011749855	28167	27568	574	25
chr15:61911844	rs115869241	VPS13C_S2904I	G	A	I	9257	0.0065896	61	0	54	7	40605	0.005885975	239	0	217	22
chr12:40304004	rs72546324	LRRK2_H1216R	A	G	G	8354	0	0	0	NA	NA	695352	4.31E-06	3	3	NA	NA
chr3:184321516	rs16858632	EIF4G1_Y311C	A	G	I	5461	0.0002105	1.149320733	1.149320733	NA	0	77190	0.000315988	24.39112309	21.39112309	NA	3
chr15:62008659	rs767007361	VPS13C_R372X	G	A	G	12157	0	0	0	0	NA	658928	7.59E-06	5	5	0	NA
chr3:132478139	rs141952333	DNAJC13_R903	G	A	I	9254	0.0076724	71	0	64	7	40601	0.006502303	264	0	250	14
chr3:132502295	rs55825559	DNAJC13_P151	C	T	I	23789	0.0383855	913.1525612	412.1525612	413	88	2407822	0.028792441	69327.07216	67066.07216	2095	166
chr6:162443314	rs137853059	PRKN_V56E	A	T	G	30662	9.78E-05	3	3	0	NA	3095804	7.46E-05	231	230	1	NA
chr15:61917535	rs114277501	VPS13C_V2621I	C	T	I	7806	0	0	0	0	NA	38051	0	0	0	0	NA
chr15:61854888	rs62007358	VPS13C_D3715	C	A	I	9257	0.0033488	31	0	26	5	40605	0.003447851	140	0	136	4
chr15:61915655	rs34060567	VPS13C_K2808I	T	C	I	1451	0.0013784	2	0	NA	2	2556	0.000782473	2	0	NA	2
chr12:40240543	rs112794616	LRRK2_A211V	C	T	I	13597	3.33E-05	0.452351623	0.452351623	0	0	660168	9.78E-05	64.57600839	64.57600839	0	0
chr15:89319031	rs796052911	POLG_F1058S	A	G	G	14560	0	0	0	NA	NA	2368718	7.18E-06	17	17	NA	NA
chr1:155238629	rs79653797	GBA1_R159Q	C	T	G	22362	0	0	0	0	NA	2406454	1.62E-05	39	37	2	NA
chr1:16988161	rs55708915	ATP13A2_I902F	T	A	I	9256	0.0044296	41	0	36	5	40606	0.003447766	140	0	135	5
chr12:40367050	rs55633591	LRRK2_N2479D	A	G	G	8361	0	0	0	NA	NA	695088	1.44E-06	1	1	NA	NA
chr15:61984885	rs141515062	VPS13C_S565T	A	T	I	9257	0.0008642	8	0	7	1	40606	0.000664926	27	0	27	0
chr1:16992042	rs61734958	ATP13A2_V693	A	G	I	7806	0	0	0	0	NA	38051	0	0	0	0	NA
chr16:46671766	rs797044948	VPS35_Q488R	T	C	G	14547	0	0	0	NA	NA	2367228	4.22E-07	1	1	NA	NA
chr3:184327622	rs2230570	EIF4G1_L1234P	T	C	I	23805	0.0344036	818.9772482	346.9772482	399	73	2409539	0.024301166	58554.60804	56450.60804	2007	97

chr2:74531688	rs201615648	HTRA2_R344H	G	A	G	22366	0	0	0	0	NA	2406819	9.56E-06	23	23	0	NA
chr2:232844373	rs144086186	GIGYF2_S1035(	C	G	I	9257	0.0025926	24	0	23	1	40605	0.004260559	173	0	164	9
chr12:40298346	rs111341148	LRRK2_R1067Q	G	A	G	18871	0	0	0	NA	NA	2982265	7.7E-06	23	23	NA	NA
chr15:89320917	rs768653086	POLG_E944K	C	T	G	22338	0	0	0	0	NA	2400934	4.17E-06	10	10	0	NA
chr15:89321842	rs41549716	POLG_Y831C	T	C	I	19778	0.0141477	279.8124845	99.81248446	155	25	742259	0.010094553	7492.772624	6654.772624	796	42
chr20:5069330	rs141618836	TMEM230_R81(	C	T	I	7806	0	0	0	0	NA	38051	0	0	0	0	NA
chr22:38169255	rs11570605	PLA2G6_V58I	C	T	I	1451	0.0006892	1	0	NA	1	2556	0.000391236	1	0	NA	1
chr1:155238251	.	GBA1_A215D	G	T	G	12146	0	0	0	0	NA	657674	1.52E-06	1	0	1	NA
chr15:89333427	rs139599587	POLG_H110Y	G	A	I	7806	0	0	0	0	NA	38051	0	0	0	0	NA
chr1:155239716	rs121908312	GBA1_K118N	C	G	G	18895	5.29E-05	1	1	NA	NA	2986365	1.07E-05	32	32	NA	NA
chr14:22877108	rs146141715	LRP10_A575T	G	A	I	9257	0.0007562	7	0	5	2	40604	0.000246281	10	0	9	1
chr12:40320103	rs200143418	LRRK2_Q1648R	A	G	I	9256	0.0002161	2	0	1	1	40601	0.000517229	21	0	19	2
chr15:62023837	rs180757384	VPS13C_R153C	G	A	I	7806	0.0001281	1	0	1	NA	38050	0.000131406	5	0	5	NA
chr1:155235777	rs77738682	GBA1_N431I	T	A	G	3853	0.0002595	1	1	NA	NA	537158	3.91E-05	21	21	NA	NA
chr22:32475378	rs9621461	FBXO7_G6E	G	A	I	9229	0.1987214	1834	0	1525	309	40469	0.19886827	8048	0	7540	508
chr22:38126417	rs76718524	PLA2G6_R461W	G	A	G	23804	0.00021	5	4	0	1	2407339	0.000337302	812	793	18	1
chr15:61927138	rs75341202	VPS13C_L2157F	G	A	I	23810	0.0186954	445.1369562	189.1369562	213	43	2409573	0.013247781	31921.49557	30771.49557	1081	69
chr12:40310561	rs113431708	LRRK2_R1483Q	G	A	G	12160	0	0	0	0	NA	658830	3.34E-05	22	22	0	NA
chr15:89325639	rs113994096	POLG_P587L	G	A	I	32086	0.0033047	106.0339863	57.03398627	39	10	3091846	0.002525879	7809.628489	7622.628489	170	17
chr1:16986091	rs189334432	ATP13A2_P1124	G	A	I	9252	0.0109166	101	0	89	12	40573	0.013383284	543	0	522	21
chr1:16986246	rs372995036	ATP13A2_P1173	G	A	I	9257	0.0002161	2	0	1	1	40607	0.000123131	5	0	5	0
chr6:162262627	rs769099303	PRKN_R104W	G	A	G	12143	0	0	0	0	NA	656470	2.59E-05	17	17	0	NA
chr1:155237370	rs765633380	GBA1_R324C	G	A	G	12124	0	0	0	0	NA	653876	1.22E-05	8	5	3	NA
chr1:155235780	rs76910485	GBA1_P430L	G	A	G	8360	0.0001196	1	1	NA	NA	695195	1.44E-06	1	1	NA	NA
chr2:232790836	rs371141455	GIGYF2_S284T	G	C	I	9257	0.0003241	3	0	2	1	40606	0.000270896	11	0	11	0
chr15:89321792	rs113994098	POLG_G848S	C	T	I	32057	0.0005296	16.97793122	4.977931225	12	0	3086206	0.000225159	694.8884895	664.8884895	29	1
chr1:155236246	rs75548401	GBA1_T408M	G	A	I	13243	0.0155374	205.7612408	23.76124075	150	32	114594	0.008836033	1012.556385	440.5563848	535	37
chr1:20637888	rs45604240	PINK1_T145M	C	T	G	16158	0	0	0	0	NA	733418	5.73E-05	42	40	2	NA
chr12:40364843	rs78964014	LRRK2_E2395K	G	A	G	16150	0	0	0	0	NA	732792	6.82E-06	5	5	0	NA
chr3:132507256	rs79953286	DNAJC13_Y167	A	G	I	9241	0.116654	1078	0	878	200	40491	0.109061273	4416	0	4089	327
chr15:61945803	rs114089496	VPS13C_A1687	G	A	I	9256	0.0054019	50	0	43	7	40607	0.006328958	257	0	236	21
chr22:38115667	rs121908683	PLA2G6_R632W	G	A	G	11805	0	0	0	0	NA	112060	8.92E-06	1	0	1	NA
chr12:40251273	rs78501232	LRRK2_E334K	G	A	I	17608	0.0008228	14.48834972	6.488349717	5	3	735799	0.000754041	554.8227762	539.8227762	10	5
chr15:61954471	rs140338178	VPS13C_I1417L	T	G	I	9257	0.0019445	18	0	14	4	40605	0.001847063	75	0	68	7
chr15:61880845	rs377118536	VPS13C_R3296	G	A	I	7803	0.0001282	1	0	1	NA	38043	2.63E-05	1	0	1	NA
chrX:155260942	rs587777874	RAB39B_T168K	G	T	G	14513	0	0	0	NA	NA	2361856	2.12E-06	5	5	NA	NA
chr1:17005754	rs151117874	ATP13A2_T12M	G	A	I	9257	0.0004321	4	NA	4	0	40607	0.000394021	16	NA	16	0
chr1:20649109	rs45539432	PINK1_Q456X	C	T	G	28161	0	0	0	0	0	3028022	0.000144649	438	433	4	1
chr3:184321987	rs200221361	EIF4G1_A468E	C	A	I	0	NA	0	NA	NA	NA	0	NA	0	NA	NA	NA
chr16:46662405	rs569735490	VPS35_F635L	A	C	I	7806	0	0	0	0	NA	38051	2.63E-05	1	0	1	NA
chr15:61963904	rs200815172	VPS13C_A1088	C	T	I	9256	0.0008643	8	0	6	2	40603	0.000985149	40	0	40	0
chr15:61920268	rs143926369	VPS13C_V2426I	C	T	I	9257	0.0043211	40	0	37	3	40606	0.004900754	199	0	184	15
chr15:62023783	rs150832196	VPS13C_K171E	T	C	I	9256	0.0010804	10	0	9	1	40605	0.001748553	71	0	63	8
chr15:89333267	rs752892262	POLG_P163L	G	A	G	15530	0.0007083	11	11	NA	0	2288362	0.001018633	2331	2331	NA	0
chr3:132511169	rs142160751	DNAJC13_E174(	G	C	I	9253	0.0045391	42	0	35	7	40599	0.003670041	149	0	144	5
chr6:161350208	rs191486604	PRKN_G430D	C	T	I	23814	0.0004959	11.80834171	1.808341713	9	1	2408615	0.000131265	316.1660285	294.1660285	20	2
chr15:89318587	rs2307440	POLG_R1146C	G	A	I	32107	0.0001407	4.516763805	3.516763805	1	0	3097333	0.000157054	486.4500376	470.4500376	14	2
chr12:40274671	rs79299560	LRRK2_L582P	T	C	G	8351	0	0	0	NA	NA	694563	2.88E-06	2	2	NA	NA
chr15:61984953	rs35942317	VPS13C_T542M	G	A	I	9257	0	0	0	0	0	40604	0.000123141	5	0	5	0
chr1:155239948	rs1141811	GBA1_T82I	G	A	G	8293	0	0	0	NA	NA	687966	1.45E-05	10	10	NA	NA
chr1:65401812	rs143504255	DNAJC6_S663L	C	T	I	7806	0.0001281	1	0	1	NA	38051	2.63E-05	1	0	1	NA
chr15:89324193	rs2307450	POLG_E662K	C	T	I	9257	0.0004321	4	0	4	0	40607	0.000369394	15	0	12	3
chr15:61969413	rs146460562	VPS13C_T933A	T	C	I	9236	0.0072542	67	0	52	15	40494	0.008865511	359	0	336	23
chr1:155235814	.	GBA1_D419N	C	T	G	12158	0	0	0	0	NA	658707	4.55E-06	3	2	1	NA
chr15:89327004	rs769637557	POLG_K498T	T	G	G	23796	0.0005043	12	5	5	2	2406554	0.000226049	544	519	25	0
chr12:40304000	rs143710836	LRRK2_A1215T	G	A	I	9257	0.0004321	4	0	3	1	40607	0.00019701	8	0	8	0
chr22:38126371	rs146684391	PLA2G6_T476I	G	A	I	9255	0.0007563	7	0	5	2	40594	0.00076366	31	0	30	1
chr21:32688348	rs532075408	SYNJ1_R309H	C	T	I	9257	0	0	0	0	0	40607	4.93E-05	2	0	2	0
chr12:40364975	rs72547983	LRRK2_L2439I	C	A	G	16165	0	0	0	0	NA	733492	6.00E-05	44	42	2	NA
chr1:155239933	rs78769774	GBA1_R87Q	C	T	G	12161	0	0	0	0	NA	658939	4.55E-06	3	2	1	NA
chr12:40299255	rs281865046	LRRK2_L1165P	T	C	G	4280	0	0	0	NA	NA	607578	0.000225485	137	137	NA	NA
chr15:61961592	rs2303405	VPS13C_Y1302(	T	C	I	34240	0.0987596	3381.529834	1959.529834	1182	240	3102238	0.079383947	246267.8974	240035.8974	5844	388
chr22:38116200	rs587784338	PLA2G6_T585I	G	A	G	14535	0	0	0	NA	NA	2365630	1.27E-06	3	3	NA	NA
chr12:40251361	rs72546336	LRRK2_N363S	A	G	G	4352	0	0	0	NA	NA	620465	1.61E-06	1	1	NA	NA

chr12:40299212	rs74985840	LRRK2_A1151T	G	A	G	16170	0.0001855	3	2	1	NA	733615	0.000222187	163	161	2	NA
chr3:132538225	rs138367039	DNAJC13_M222	G	A	I	9254	0.0065917	61	0	51	10	40600	0.007931034	322	0	301	21
chr15:89333364	rs562847013	POLG_Y131H	A	G	I	9257	0.0012963	12	0	10	2	40607	0.000566405	23	0	19	4
chr21:32688306	rs565013600	SYNJ1_R323K	C	T	I	7806	0	0	0	0	NA	38051	0	0	0	0	NA
chr3:132523636	rs10935014	DNAJC13_V199	G	C	I	9257	0.0014043	13	0	9	4	40606	0.002142541	87	0	79	8
chr15:61920099	rs115481870	VPS13C_R2482	C	T	I	9257	0.012423	115	0	93	22	40588	0.012688479	515	0	473	42
chr3:184331762	rs141379472	EIF4G1_T1478M	C	T	I	9257	0.0004321	4	0	3	1	40607	0.000443273	18	0	17	1
chr2:232839964	rs146430802	GIGYF2_R961Q	G	A	I	9257	0.0017284	16	0	13	3	40605	0.001625416	66	0	58	8
chr3:132492416	rs201816934	DNAJC13_R120	G	A	G	22367	0	0	0	0	NA	2406993	1.16E-05	28	27	1	NA
chr6:161350211	rs760223151	PRKN_G429E	C	T	G	22361	4.47E-05	1	1	0	NA	2405890	7.98E-05	192	192	0	NA
chr1:16986097	rs15786	ATP13A2_P1122	G	A	I	27749	0.0780738	2166.468522	1067.468522	946	153	2478117	0.058741413	145568.0943	140621.0943	4696	251
chr2:232791432	rs34845648	GIGYF2_P423L	C	T	I	23807	0.000103	2.452830754	1.452830754	1	0	2407625	0.000100659	242.3489034	236.3489034	5	1
chr1:7984971	rs74315354	PARK7_E163K	G	A	G	26663	0	0	0	0	NA	3016947	1.99E-06	6	6	0	NA
chr14:22877045	rs201213246	LRP10_R554X	C	T	G	12067	0	0	0	0	NA	644400	0.000307263	198	197	1	NA
chr3:184321497	rs116508885	EIF4G1_R305C	C	T	I	7806	0.0001281	1	0	1	NA	38051	5.26E-05	2	0	2	NA
chr1:65386820	rs761817101	DNAJC6_R278H	G	A	I	7806	0.0003843	3	0	3	NA	38045	0.000210277	8	0	8	NA
chr15:61907289	rs116802310	VPS13C_N3027	T	C	I	7806	0	0	0	0	NA	38051	0	0	0	0	NA
chr15:61890334	rs114345245	VPS13C_R3058	G	A	I	7806	0	0	NA	0	NA	38050	2.63E-05	1	NA	1	NA
chr1:155241085	rs80356759	NA	C	T	G	14558	0	0	0	NA	NA	2367999	4.22E-07	1	1	NA	NA
chr22:38135061	rs587784362	PLA2G6_M274R	A	C	G	14559	0	0	0	NA	NA	2368829	4.22E-07	1	1	NA	NA
chr14:22876816	rs74357167	LRP10_D518N	G	A	I	9257	0.0002161	2	0	0	2	40607	0.00019701	8	0	1	7
chr14:22876765	rs116010131	LRP10_A501S	G	T	I	7806	0.0001281	1	0	1	NA	38051	2.63E-05	1	0	1	NA
chr6:161350187	rs149953814	PRKN_P437L	G	A	I	23775	0.0037047	88.07839302	34.07839302	48	6	2402894	0.002432878	5845.947417	5543.947417	291	11
chr22:38115588	rs587784340	PLA2G6_N658T	T	G	G	14505	0	0	0	NA	NA	2361345	5.51E-06	13	13	NA	NA
chr22:38116119	rs200117092	PLA2G6_R612L	C	A	I	9257	0	0	0	0	0	40607	0.000123131	5	0	4	1
chr1:155238596	rs80356763	GBA1_R170L	C	A	G	8312	0	0	0	NA	NA	692989	7.22E-06	5	5	NA	NA
chr22:38143219	rs150190277	PLA2G6_G2A	C	G	I	9257	0.000108	1	0	1	0	40607	0.000369394	15	0	14	1
chr1:155236276	rs74979486	GBA1_R398Q	C	T	G	8363	0.0001196	1	1	NA	NA	695470	8.63E-06	6	6	NA	NA
chr15:61856408	rs138846118	VPS13C_R3652	G	A	I	13611	0.0015668	21.32541886	2.325418856	19	0	661464	0.000593522	392.5932994	331.5932994	60	1
chr3:132492497	rs201263331	DNAJC13_Y123	A	G	I	23816	0.0005783	13.77356323	5.773563226	5	3	2408775	0.000400257	964.1290078	939.1290078	18	7
chr22:38126374	rs139184008	PLA2G6_R475Q	C	T	I	9257	0.0007562	7	0	5	2	40600	0.000640394	26	0	23	3
chr15:61983969	rs202117436	VPS13C_Q589X	G	A	G	4349	0	0	0	NA	NA	620735	4.03E-05	25	25	NA	NA
chr20:5109429	rs141394228	TMEM230_M2de	A	G	I	9257	0.0019445	18	0	17	1	40606	0.003275378	133	0	121	12
chr22:38132922	rs587784363	PLA2G6_R329H	C	T	G	22341	0	0	0	0	NA	2403578	4.37E-05	105	104	1	NA
chr6:161973401	rs137853058	PRKN_C212Y	C	T	G	30664	0	0	0	0	NA	3095033	1.10E-05	34	33	1	NA
chr1:155237576	rs74500255	GBA1_F255Y	A	T	G	16165	6.19E-05	1	1	0	NA	733219	1.64E-05	12	10	2	NA
chr15:89321242	rs121918047	POLG_E873X	C	A	G	16110	0	0	0	0	NA	725941	2.76E-06	2	2	0	NA
chr12:40356126	rs12581902	LRRK2_N2261I	A	T	G	8326	0.0001201	1	1	NA	NA	690979	5.79E-06	4	4	NA	NA
chr3:132499168	rs147104839	DNAJC13_R140	G	A	I	9257	0.0008642	8	0	6	2	40605	0.00039404	16	0	15	1
chr3:132467277	rs200653977	DNAJC13_M724	G	A	I	7806	0.0001281	1	0	1	NA	38050	0.000446781	17	0	17	NA
chr3:184319745	rs13319149	EIF4G1_T161A	A	G	I	9255	0.008752	81	0	70	11	40590	0.008401084	341	0	331	10
chr22:38169240	rs11570606	PLA2G6_R63G	T	C	I	0	NA	0	NA	NA	NA	0	NA	0	NA	NA	NA
