# Supplementary material for: Analysis of rare Parkinson’s disease variants in millions of people
Source: NPJ Parkinsons Dis. 2024 Jan 8;10:11. doi: 10.1038/s41531-023-00608-8 (PMC10774311; doi:10.1038/s41531-023-00608-8)
Supplement: Supplementary file 2 — reporting summary [file 41531_2023_608_MOESM2_ESM.pdf]

nature portfolio

Corresponding author(s): Vanessa Pitz

Last updated by author(s): Nov 21, 2023

Reporting Summary

Nature Portfolio wishes to improve the reproducibility of the work that we publish. This form provides structure for consistency and transparency in reporting. For further information on Nature Portfolio policies, see our Editorial Policies and the Editorial Policy Checklist.

Statistics

For all statistical analyses, confirm that the following items are present in the figure legend, table legend, main text, or Methods section.

n/a	Confirmed	
☐	☒	The exact sample size (*n*) for each experimental group/condition, given as a discrete number and unit of measurement
☒	☐	A statement on whether measurements were taken from distinct samples or whether the same sample was measured repeatedly
☒	☐	The statistical test(s) used AND whether they are one- or two-sided<br>*Only common tests should be described solely by name; describe more complex techniques in the Methods section.*
☐	☒	A description of all covariates tested
☐	☒	A description of any assumptions or corrections, such as tests of normality and adjustment for multiple comparisons
☐	☒	A full description of the statistical parameters including central tendency (e.g. means) or other basic estimates (e.g. regression coefficient) AND variation (e.g. standard deviation) or associated estimates of uncertainty (e.g. confidence intervals)
☒	☐	For null hypothesis testing, the test statistic (e.g. *F*, *t*, *r*) with confidence intervals, effect sizes, degrees of freedom and *P* value noted<br>*Give P values as exact values whenever suitable.*
☒	☐	For Bayesian analysis, information on the choice of priors and Markov chain Monte Carlo settings
☐	☒	For hierarchical and complex designs, identification of the appropriate level for tests and full reporting of outcomes
☐	☒	Estimates of effect sizes (e.g. Cohen's *d*, Pearson's *r*), indicating how they were calculated

Our web collection on statistics for biologists contains articles on many of the points above.

Software and code

Policy information about availability of computer code

Data collection	Used open-source accessible data sets AMP-PD and UKB, summary statistics from 23andMe as part of a collaboration
Data analysis	R3.6, bash, plink1.9, plink2.0, ANNOVAR (refGene, avsnp150, and clinvar_20220320), METAL,

For manuscripts utilizing custom algorithms or software that are central to the research but not yet described in published literature, software must be made available to editors and reviewers. We strongly encourage code deposition in a community repository (e.g. GitHub). See the Nature Portfolio guidelines for submitting code & software for further information.

Data

Policy information about availability of data

All manuscripts must include a data availability statement. This statement should provide the following information, where applicable:

- Accession codes, unique identifiers, or web links for publicly available datasets
- A description of any restrictions on data availability
- For clinical datasets or third party data, please ensure that the statement adheres to our policy

All AMP-PD (https://amp-pd.org/) and UK Biobank (https://www.ukbiobank.ac.uk/) data is available via application on their websites, and 23andMe summary statistics are available via application at https://research.23andme.com/dataset-access/. All code used for this analysis is available in the GitHub repository that can be found here: https://github.com/neurogenetics/23andme_rare_variants.

Research involving human participants, their data, or biological material

Policy information about studies with human participants or human data. See also policy information about sex, gender (identity/presentation), and sexual orientation and race, ethnicity and racism.

Reporting on sex and gender	We did not report on sex and geneder. The term sex was used appropriately and only used as a covariate in the analyses.
Reporting on race, ethnicity, or other socially relevant groupings	We did not report on race, ethnicity, or other socially relevant groupings. We used the TOWNSEND score as covariate in the UKB analysis.
Population characteristics	Our cohorts had similar age (SD) ranges: 23andMe 72.3 (±10.9), AMP-PD 61.3 (±10.2), and UKB 59.1 (±7.1) in cases, and 50.1 (±17.5), 70.7 (±13.2), and 64.1 (±2.8) in controls. 23andMe data was provided as summary statistics, AMP-PD data were derived from whole-genome sequencing, UKB data were derived from whole-exome sequencing.
Recruitment	Cohort recruitment for the AMP-PD study, utilizing sources like the UK Biobank (UKB), and 23andMe may introduce several biases. Here are some potential biases associated with this recruitment strategy:<br><br>1. Selection Bias: Participants in the UKB and 23andMe may not be representative of the broader population, potentially skewing the data towards individuals who have a higher interest in genetics or those who can afford genetic testing.<br><br>2. Ascertainment Bias: People who opt for genetic testing through 23andMe or enroll in the UKB may already have some awareness or concern about Parkinson's disease, leading to a biased sample of individuals predisposed to the condition or with a family history.<br><br>3. Socioeconomic Bias: Access to and affordability of genetic testing may limit participation to individuals from specific socioeconomic backgrounds, potentially excluding lower-income or disadvantaged populations and affecting the generalizability of the findings.<br><br>4. Health Bias: People opting for genetic testing or joining the UKB might already be more health-conscious, potentially biasing the sample towards individuals with a healthier lifestyle or better access to healthcare services.<br><br>5. Ethnic Bias: Genetic databases may be skewed towards certain ethnicities due to varying uptake and interest in genetic testing within different ethnic groups, leading to underrepresentation or overrepresentation of specific populations.<br><br>6. Age Bias: Genetic testing and participation in the UKB may attract certain age groups more than others, potentially skewing the age distribution and influencing the study's results, especially for a disease like Parkinson's which is more prevalent in older populations.<br><br>7. Geographic Bias: Participation may vary by geographic location due to differing levels of awareness, accessibility, and interest in genetic testing and research studies, impacting the generalizability of the findings to broader geographic regions.
Ethics oversight	Each contributing study abided by the ethics guidelines set out by their institutional review boards, and all participants gave written informed consent to participate in both their initial cohorts and subsequent studies. The research used was deemed ‘not human subjects research’ by the NIH Office of IRB Operations and stated that no IRB approval is required. Studies that are conducted on de-identified human genetics are waived ethical approval by the NIH Intramural IRB, as they are considered non-human subjects research. All authors and the public can access the statistical programming code used in this project for the analyses and results generation.

Note that full information on the approval of the study protocol must also be provided in the manuscript.

Field-specific reporting

Please select the one below that is the best fit for your research. If you are not sure, read the appropriate sections before making your selection.

☒ Life sciences ☐ Behavioural & social sciences ☐ Ecological, evolutionary & environmental sciences

For a reference copy of the document with all sections, see nature.com/documents/nr-reporting-summary-flat.pdf

Life sciences study design

All studies must disclose on these points even when the disclosure is negative.

Sample size	27,590 cases (+6,701 proxies), 3,106,080 controls
Data exclusions	We only used publicly available data sets. No data was excluded.
Replication	Findings were replicated in 2 different studies, meta-analysis was carefully assessed.

Randomization	Participants were already allocated in case and control groups. Covariates were age, sex, principal components 1-5 and socioeconomic factors like townsend score
Blinding	This was an association analysis and not a clinical trial. Blinding was not required. We worked with anonymized data.

Reporting for specific materials, systems and methods

We require information from authors about some types of materials, experimental systems and methods used in many studies. Here, indicate whether each material, system or method listed is relevant to your study. If you are not sure if a list item applies to your research, read the appropriate section before selecting a response.

Materials & experimental systems

n/a	Involved in the study
☒	☐ Antibodies
☒	☐ Eukaryotic cell lines
☒	☐ Palaeontology and archaeology
☒	☐ Animals and other organisms
☒	☐ Clinical data
☒	☐ Dual use research of concern
☒	☐ Plants

Methods

n/a	Involved in the study
☒	☐ ChIP-seq
☒	☐ Flow cytometry
☒	☐ MRI-based neuroimaging
